# Mathematical modelling of the influence of *ACE I/D* polymorphism on blood pressure and antihypertensive therapy

**DOI:** 10.1016/j.heliyon.2024.e29988

**Published:** 2024-04-23

**Authors:** Elena Kutumova, Anna Kovaleva, Ruslan Sharipov, Galina Lifshits, Fedor Kolpakov

**Affiliations:** a Department of Computational Biology, Sirius University of Science and Technology, Sirius, Krasnodar region, Russia; b Laboratory of Bioinformatics, Federal Research Center for Information and Computational Technologies, Novosibirsk, Russia; c Biosoft.Ru, Ltd., Novosibirsk, Russia; d Laboratory for Personalized Medicine, Center of New Medical Technologies, Institute of Chemical Biology and Fundamental Medicine SB RAS, Novosibirsk, Russia; e Specialized Educational Scientific Center, Novosibirsk State University, Novosibirsk, Russia

**Keywords:** Mathematical modelling, *ACE I/D* polymorphism, Antihypertensive therapy, Cardiovascular system, Renal system, Blood pressure regulation

## Abstract

The angiotensin-converting enzyme (ACE) gene (*ACE*) insertion/deletion (*I/D*) polymorphism raises the possibility of personalising ACE inhibitor therapy to optimise its efficiency and reduce side effects in genetically distinct subgroups. However, the extent of its influence among these subgroups is unknown. Therefore, we extended our computational model of blood pressure regulation to investigate the effect of the *ACE I/D* polymorphism on haemodynamic parameters in humans undergoing antihypertensive therapy. The model showed that the dependence of blood pressure on serum ACE activity is a function of saturation and therefore, the lack of association between *ACE I/D* and blood pressure levels may be due to high ACE activity in specific populations. Additionally, in an extended model simulating the effects of different classes of antihypertensive drugs, we explored the relationship between *ACE I/D* and the efficacy of inhibitors of the renin–angiotensin–aldosterone system. The model predicted that the response of cardiovascular and renal parameters to treatment directly depends on ACE activity. However, significant differences in parameter changes were observed only between groups with high and low ACE levels, while different *ACE I/D* genotypes within the same group had similar changes in absolute values. We conclude that a single genetic variant is responsible for only a small fraction of heredity in treatment success and its predictive value is limited.

## 1 Introduction

The human angiotensin-converting enzyme (ACE) gene (*ACE*) contains 26 exons interrupted by 25 introns and maps to chromosome 17q23 [[1], [2], [3]]. The *ACE* insertion/deletion (*I/D*) polymorphism (rs1799752, rs4340, rs13447447 or rs4646994), identified in 1990 by Rigat and colleagues [4], has received much attention [5]. It is characterised by the presence or absence of a 287-bp *Alu* repetitive sequence within intron 16 [3], resulting in three genotypes: *DD* and *II* homozygotes and *ID* heterozygotes [6,7]. The *ACE D* allele is associated with the risk of coronary heart disease [8] and myocardial infarction [9], which is also confirmed by meta-analysis data [10]. However, this association has been observed only in small studies [11]. Some experiments have shown an association between the *DD* genotype and a higher prevalence of left ventricular hypertrophy [[12], [13], [14]], which was not found in other studies [10]. The association of the *ACE I/D* polymorphism with other pathological conditions has also been discussed, including pregnancy-related complications [15], insulin-resistant polycystic ovary syndrome [16], susceptibility to SARS-CoV-2 infection [[17], [18], [19], [20], [21]], risk of glioma [22,23] and cerebral microbleed [24].

The relationship between *ACE I/D* polymorphism and the pathogenesis of essential hypertension is controversial. Results of a meta-analysis indicate that the *ACE D* allele is associated with hypertension susceptibility in Asian, Caucasian and mixed populations [25], as well as in the Chinese population [26], but this association was inconclusive in people of West African descent [27]. Heterogeneous data were also obtained for individual population groups. Barley et al. [28] revealed that in White people of European descent, there was no significant association between *ACE* genotype and high blood pressure, while Black people of Afro-Caribbean descent showed a positive association between the frequency of the *D* allele and increasing blood pressure. O'Donnell et al. [29] examined a sample of Framingham Heart Study participants and found consistent evidence for a genetic linkage between the *АСЕ* locus and hypertension and blood pressure in men but not in women. Ned et al. [30] reported that among non-Hispanic Black people, the *D* allele was linked with increased systolic blood pressure (SBP) in additive and dominant covariate-adjusted models and was also associated with increased diastolic blood pressure (DBP) in dominant models when participants taking ACE inhibitors were excluded from the analyses. However, significant genotype-sex interactions were detected only among Mexican Americans (positive associations with SBP and hypertension in women, but not in men), while this was not observed for non-Hispanic White persons and non-Hispanic Black persons. A multivariate regression analysis by Han et al. [31] demonstrated that the *ACE DD* genotype was independently associated with 24‐hour SBP, 24‐hour DBP, central SBP, and the central augmentation index in Chinese patients. Thus, it can be concluded that the influence of the *ACE* genotype on interindividual variation in blood pressure is dependent on factors including gender, age and body size [32]. However, ethnic predisposition cannot be ruled out. In addition to the above, it has been shown that *ACE I/D* polymorphism contributes to the development of hypertension in Swedes [33], North Indians [[34], [35], [36], [37]], Asian Indians [38], residents of Gujarat, Western India [39], Saudi subjects [40], Russians [41], an indigenous ethnic group of Mountain Shoria, Russia [42], Japanese [43], a Punjabi population from Faisalabad, Pakistan [44], the population of Burkina Faso, West Africa [45], the Ethiopian population, East Africa [46,47], and Han, Kazakh, Tibetan and Zhuang Chinese populations [26,48]. In contrast, no association between *ACE I/D* genotypes and hypertension has been established in Slovenians [49], Buryats [41], Thais [50], Romany subjects and Slovaks [51], a Cuban population, primarily of European and African ancestry, living in Havana [52], an Algerian population from Oran [53], and some Chinese minorities, including Mongolians, Uyghurs, Yugurs and Koreans [26].

Despite controversy regarding the relationship of *ACE I/D* variants with cardiovascular diseases, experimental studies agree that the *ACE* polymorphism affects the plasma ACE concentration and activity, which both increase with the number of *D* alleles [4,10,11,[54], [55], [56], [57], [58], [59], [60]]. A plausible mechanism for the lack of effect of *ACE I/D* on the cardiovascular system could be that subjects with the *DD* genotype, despite elevated plasma levels of ACE, do not necessarily produce increased amounts of angiotensin (Ang) II [11]. On the other hand, a number of experimental investigations noted that increased ACE activity can lead to higher levels of Ang II [55,61]. In the latter case, there is likely to be an association between the *ACE* genotype and cardiovascular risk.

Numerous studies confirm that genotype-based antihypertensive therapies are the most effective at treating hypertension and may help to avoid the occurrence of major adverse events and also decrease the costs of treatment [62]. It has been suggested that differences in circulating ACE might affect the therapeutic response of ACE inhibitors, thus explaining interindividual variability in cardiovascular or renal responses to equivalent doses of drugs. Several studies have examined the extent of the influence of the *ACE* genotype on the effectiveness of ACE inhibitors in various conditions, such as hypertension, diabetic nephropathy and coronary artery disease [5]. However, whether *ACE* genotyping helps predict the success of ACE inhibition remains unresolved [63].

To explore the association between *ACE I/D* polymorphism and the development of arterial hypertension *in silico*, we used a previously created mathematical model of the human cardiovascular and renal systems [64]. The original model incorporated the processes of blood circulation and the cardiac cycle, neurohumoral regulation, oxygen exchange between blood and tissue, the renin–angiotensin–aldosterone system (RAAS), renal microcirculation and sodium transport along the nephron, renal sympathetic nerve activity, and regulation of water and sodium balance. In a subsequent study [65], the model was improved to account for the effects of various antihypertensive agents, including RAAS blockers such as the direct renin inhibitor aliskiren, the ACE inhibitor enalapril, and the Ang II receptor blocker losartan, as well as drugs with other mechanisms of action, such as the β-blocker bisoprolol, the calcium channel blocker amlodipine and the thiazide diuretic hydrochlorothiazide.

Using this model, in the present study, we investigate why *ACE I/D* genotypes are associated with the development of hypertension in some ethnic groups but not in others and whether they are associated with the success of antihypertensive therapy with RAAS blockers. For computational analysis, we use the BioUML software, an extensible open-source platform that adapts a visual modelling approach to formally describe and simulate complex biological systems [66,67].

## 2 Results

### 2.1 Modelling the dependence of blood pressure on ACE activity

In humans, Ang I is converted to Ang II by ACE and chymase [68]. In our model [64], we defined this process according to Hallow et al. [69] as two first-order biochemical reactions with the following rate constants:

(1) $$c_{ACE}=54.1\;h^{–1},c_{chym}=1.1\;h^{–1}\text{.}$$

Ang II participates in blood pressure regulation through vasoconstriction and control of fluid-electrolyte balance [70,71]. Therefore, the first computational experiment was to analyse the dependence of arterial pressure on ACE activity ($c_{ACE}$).

**Fig. 1:**
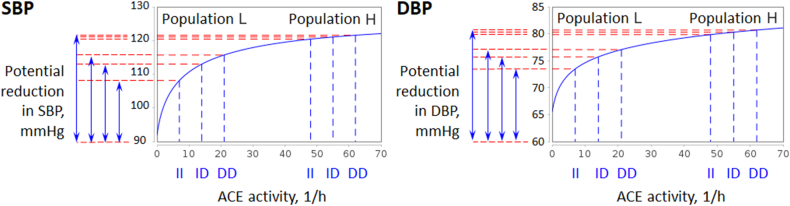
Simulated dependence of SBP and DBP on serum ACE activity (solid blue lines). Since ACE activity increases with the number of *D* alleles in the *ACE* gene, the modelled curves suggest that *ACE* polymorphism could be a marker of hypertension in population *L* with low ACE activity, while there is no such association for population *H* with high ACE activity. In addition, the potential reduction in blood pressure with ACE blockers is greater in population *H* than in population *L*.

We generated a virtual normotensive subject, i.e. we found an equilibrium parameterisation of the model with indicators of a healthy person (for more details, see Materials and Methods), and analysed the change in the equilibrium values of SBP and DBP while varying the parameter $c_{ACE}$. As can be seen from Fig. 1, the blood pressure-ACE activity curves were similar to the saturation functions. Since serum ACE activity for the three genotypes (*II*, *ID* and *DD*) increases with the number of *D* alleles [10,11,55,57,58,60], we invoked the following hypotheses.

Hypothesis 1 The lack of association between ACE I/D genotypes and blood pressure levels in a specific population is due to high ACE activity (population H in Fig. 1). In contrast, populations with such an association have low ACE activity (population L in Fig. 1).

Hypothesis 2 The potential reduction in blood pressure with ACE blockers is greater in populations without association between ACE I/D genotypes and blood pressure levels than in populations with it.

An interactive implementation of the simulation experiment shown in Fig. 1 is available online in the BioUML software (see Data Availability).

### 2.2 Generation and analysis of populations with different ACE activity

We generated two types of virtual hypertensive populations with low and high ACE activity (populations *L* and *H*, respectively) to study the relationship between *ACE I/D* polymorphism and the success of RAAS inhibitor therapy. The choice of $c_{ACE}$ parameter values in both cases and for different *ACE* genotypes was based on the following considerations.

The primary $c_{ACE}$ and $c_{chym}$ values (1) were taken from the renin–angiotensin system model developed by Lo et al. [72] in which a number of assumptions were made to estimate them:

1. The activity of ACE and chymase in converting Ang I to Ang II is assumed to be proportional to the concentration of the substrate Ang I.
2. ACE is assumed to be responsible for >95 % of the conversion of Ang I to Ang II.

To be precise, the rate constants (1) correspond to 98 % of the contribution of ACE to the formation of Ang II. However, some experimental studies have reported different results. For example, analysis of angiotensin metabolism in human kidney homogenates showed a 93 % contribution of ACE to Ang II [73], while in a group of orthotopic heart transplant recipients with normal left ventricle function, ACE mediated 89 % of the conversion of Ang I to Ang II across the myocardial circulation *in vivo* [74].

Therefore, we regard the 98 % contribution of ACE to Ang II as high ACE activity and the 89 % contribution as low ACE activity with the corresponding values of $c_{ACE}$:

$$c_{ACE}^{H}=54.1\;h^{–1},c_{ACE}^{L}=8.9\;h^{–1}\text{.}$$

Assuming that these constants are the means of the three *ACE I/D* variants, we took into account that plasma ACE activity is increased by an average of 28 % and 56 % in *ID* and *DD* genotypes compared to *ACE II* [11]. Thus, we obtained the following ACE activities for different cases:

$$c_{ACE}^{HII}=42.3\;h^{–1};\;c_{ACE}^{HID}=54.1\;h^{–1};\;c_{ACE}^{HDD}=65.9\;h^{–1}\text{;}$$

$$c_{ACE}^{LII}=7.0\;h^{–1};\;c_{ACE}^{LID}=8.9\;h^{–1};\;c_{ACE}^{LDD}=10.8\;h^{–1}\text{.}$$

Using this data, we generated three *L*-subpopulations (*LII*, *LID* and *LDD*, namely one for each *ACE* genotype) and three *H*-subpopulations (*HII*, *HID* and *HDD*) of 100 virtual patients with arterial hypertension (see the generation algorithm in Materials and Methods). The baseline characteristics of the subpopulations are given in Table 1. Distribution plots for all parameters are shown in Supplementary File 1, Figs. S1–S9, and are available in the Jupyter document on the web version of BioUML (see Data Availability). There were no significant differences between subpopulations in most parameters (Table 1 and Supplementary File 1, Table S1). However, with increasing ACE activity, there was a trend towards higher Ang II (*P* ≤ 0.001 for all subpopulations compared to each other), lower Ang I (*P* < 0.001 for any *H*-subpopulation *vs*. all other cases), and lower plasma renin activity (*P* < 0.001 for all *L*-*vs*. all *H*-subpopulations). In addition to the RAAS components, intraglomerular haemodynamic parameters were also sensitive to changes in the $c_{ACE}$ value. In *H*-subpopulations, the afferent arteriolar diameter was larger than in *L*-subpopulations (*P* < 0.001 for all pairs of such subpopulations). The glomerular hydrostatic pressure slightly increased along with the $c_{ACE}$ value (*P* < 0.001 for all *L*-subpopulations compared to *HDD*), while the afferent arteriolar resistance tended to decrease (*P* = 0.001 for *LII vs*. *HDD*). Note that for all subpopulations, we used the same generation algorithm that utilised a random number function for SBP, DBP, heart rate, stroke volume, body weight and body mass index with the same means and standard deviations (160 ± 10 mmHg, 100 ± 10 mmHg, 75 ± 10 beats/min, 70 ± 10 mL, 80 ± 20 kg and 29 ± 5 kg/m^2^, respectively). Thus, the resulting differences in subpopulations were associated with the maintenance of the same level of these parameters with variations in ACE activity.

**Table 1:** Baseline characteristics of virtual subpopulations (*n* = 100). Data are shown as mean ± SD.

Characteristics	*L-*subpopulations			*H-*subpopulations		
	LII	LID	LDD	HII	HID	HDD
General parameters						
Body mass index, kg/m^2^	28.6 ± 3.1	28.2 ± 3.1	28.4 ± 2.9	28.6 ± 3.0	28.2 ± 2.8	28.6 ± 3.4
Body weight, kg	81.2 ± 8.7	81.0 ± 8.5	81.7 ± 8.9	82.2 ± 9.8	81.0 ± 9.3	80.6 ± 9.4
**Systemic hemodynamics**						
Systolic blood pressure, mmHg	154.1 ± 6.9	154.9 ± 7.4	154.5 ± 6.5	153.7 ± 7.2	152.5 ± 6.8	154.2 ± 7.2
Diastolic blood pressure, mmHg	100.5 ± 5.6	101.0 ± 5.3	100.9 ± 5.1	100.1 ± 5.3	100.5 ± 5.8	100.5 ± 5.9
Heart rate, beats/min	77.2 ± 6.4	75.7 ± 7.6	75.2 ± 7.1	75.9 ± 7.1	76.6 ± 6.8	76.0 ± 7.4
Systemic arterial elasticity, mmHg/mL	0.9 ± 0.2	0.9 ± 0.2	0.9 ± 0.2	0.9 ± 0.2	0.9 ± 0.2	1.0 ± 0.2
Systemic vascular resistance, s∙mmHg/mL	1.4 ± 0.2	1.4 ± 0.2	1.4 ± 0.2	1.4 ± 0.2	1.4 ± 0.2	1.5 ± 0.2
**Pulmonary hemodynamics**						
Diastolic pulmonary arterial pressure, mmHg	11.3 ± 0.6	11.3 ± 0.5	11.2 ± 0.7	11.2 ± 0.6	11.1 ± 0.7	11.3 ± 0.6
Systolic pulmonary arterial pressure, mmHg	17.8 ± 1.5	17.6 ± 1.3	17.5 ± 1.4	17.4 ± 1.4	17.7 ± 1.6	17.8 ± 1.5
Pulmonary vascular resistance, s∙mmHg/mL	0.097 ± 0.012	0.100 ± 0.014	0.097 ± 0.015	0.098 ± 0.015	0.098 ± 0.013	0.100 ± 0.014
**Left ventricle (LV)**						
Stroke volume, mL	73.2 ± 6.9	72.5 ± 7.6	73.5 ± 7.8	72.8 ± 7.3	73.4 ± 7.9	71.8 ± 7.0
Ejection fraction, %	66.6 ± 5.6	66.4 ± 6.2	66.7 ± 6.5	66.3 ± 6.8	66.5 ± 6.2	65.5 ± 6.6
LV end-diastolic pressure, mmHg	8.1 ± 1.3	8.0 ± 1.2	8.1 ± 1.1	8.0 ± 1.0	8.0 ± 1.1	8.2 ± 1.0
LV peak systolic pressure, mmHg	174.0 ± 6.6	174.3 ± 6.9	173.8 ± 6.4	172.8 ± 6.7	172.6 ± 6.8	173.4 ± 7.0
LV end-diastolic volume, mL	110.6 ± 12.8	110.0 ± 14.2	111.0 ± 14.0	110.5 ± 13.3	111.2 ± 13.5	110.5 ± 13.4
LV end-systolic volume, mL	37.3 ± 9.4	37.5 ± 10.2	37.5 ± 10.5	37.7 ± 10.9	37.7 ± 10.1	38.6 ± 10.7
**Right ventricle (RV)**						
RV end-diastolic pressure, mmHg	5.4 ± 1.2	5.8 ± 1.1	5.2 ± 1.2	5.6 ± 1.1	5.6 ± 1.1	5.7 ± 1.0
RV peak systolic pressure, mmHg	20.2 ± 1.6	19.9 ± 1.4	19.9 ± 1.6	19.7 ± 1.5	20.1 ± 1.6	20.1 ± 1.7
RV end-diastolic volume, mL	116.8 ± 14.7	118.1 ± 14.0	117.4 ± 14.9	117.2 ± 14.3	119.2 ± 12.4	117.1 ± 13.1
RV end-systolic volume, mL	43.5 ± 12.2	45.5 ± 10.5	43.9 ± 11.4	44.4 ± 11.5	45.7 ± 9.7	45.2 ± 11.4
**Renal function**						
Glomerular filtration rate, mL/min	94.5 ± 19.2	94.8 ± 19.7	91.6 ± 19.7	92.2 ± 18.6	93.6 ± 18.1	95.0 ± 19.6
Renal blood flow, L/min	1.1 ± 0.1	1.1 ± 0.2	1.1 ± 0.2	1.1 ± 0.2	1.1 ± 0.2	1.1 ± 0.2
Renal vascular resistance, mmHg∙min/L	107.4 ± 14.3	106.0 ± 15.9	105.1 ± 15.3	104.7 ± 13.7	102.7 ± 12.8	102.0 ± 13.9
**Intraglomerular hemodynamics**						
Afferent arteriolar diameter, μm	12.0 ± 1.3^b^	12.2 ± 1.2^b^	12.1 ± 1.2^b^	13.3 ± 1.5^b^	13.7 ± 1.6^b^	13.7 ± 1.5^b^
Efferent arteriolar diameter, μm	18.3 ± 1.1	18.5 ± 1.0	18.3 ± 0.9	18.8 ± 0.9	18.7 ± 1.0	18.9 ± 0.8
Afferent arteriolar resistance, dyn∙s/cm^5^	5011.4 ± 814.3^d^	4936.5 ± 864.4	4886.3 ± 859.1	4773.4 ± 756.5	4612.7 ± 738.8	4586.3 ± 778.0
Efferent arteriolar resistance, dyn∙s/cm^5^	2484.9 ± 492.6	2489.1 ± 529.6	2418.6 ± 497.6	2558.7 ± 491.0	2580.7 ± 490.7	2528.0 ± 465.6
Glomerular hydrostatic pressure, mmHg	51.5 ± 2.8^d^	51.9 ± 3.0^d^	52.0 ± 3.0^d^	52.9 ± 3.3	53.7 ± 4.0	54.0 ± 3.6
**Biochemical parameters**						
Hematocrit, %	44.0 ± 4.2	44.3 ± 3.6	44.7 ± 4.0	43.8 ± 3.8	43.4 ± 4.1	43.7 ± 4.3
Hemoglobin, g/L	149.4 ± 11.7	146.8 ± 12.4	149.2 ± 12.2	147.2 ± 13.0	149.4 ± 11.9	149.3 ± 10.9
Sodium, mEq/L	143.6 ± 1.3	143.6 ± 1.2	143.7 ± 1.3	143.5 ± 1.4	143.4 ± 1.3	143.5 ± 1.4
Potassium, mEq/L	4.5 ± 0.5^d^	4.4 ± 0.5	4.4 ± 0.5	4.3 ± 0.5	4.3 ± 0.4	4.2 ± 0.4
Glucose, mmol/L	4.9 ± 0.6	5.0 ± 0.6	4.9 ± 0.6	5.0 ± 0.6	5.0 ± 0.6	5.0 ± 0.6
Total protein, g/L	75.2 ± 4.7	74.4 ± 5.5	74.4 ± 5.2	74.5 ± 5.3	74.2 ± 5.5	73.5 ± 5.7
Urea, mmol/L	4.4 ± 1.4	4.4 ± 1.3	4.5 ± 1.5	4.5 ± 1.5	4.4 ± 1.5	4.2 ± 1.5
**Renin–angiotensin–aldosterone system**						
Plasma renin activity, fmol/mL/min	68.1 ± 9.1^b^	64.1 ± 11.6^b^	60.7 ± 11.8^b^	33.2 ± 2.8^b^	31.3 ± 4.0^b^	30.3 ± 3.9^b^
Plasma angiotensin I, fmol/mL	23.3 ± 3.1^b^	21.7 ± 3.9^b^	20.3 ± 4.0^b^	9.4 ± 0.8^bc^	8.4 ± 1.1^bc^	7.7 ± 1.0^bc^
Plasma angiotensin II, fmol/mL	2.1 ± 0.3^ab^	2.5 ± 0.4^ab^	2.8 ± 0.5^ab^	4.7 ± 0.4^bc^	5.3 ± 0.7^bc^	5.9 ± 0.8^bc^
Plasma aldosterone, pg/mL	166.0 ± 59.3	172.0 ± 63.2	176.0 ± 52.1	193.2 ± 62.1	196.3 ± 64.2	188.0 ± 60.5

^a^*P* ≤ 0.001 for any *L*-subpopulation *vs*. all other *L*-subpopulations. ^b^*P* ≤ 0.001 for any *L*-subpopulation *vs*. all *H*-subpopulations. ^c^*P* ≤ 0.001 for any *H*-subpopulation *vs*. all other *H*-subpopulations. ^d^*P* ≤ 0.001 *vs. HDD*. **SD** = standard deviation.

### 2.3 Simulation of antihypertensive treatment

To analyse the antihypertensive efficacy of RAAS blockers in different genotypic subpopulations, we simulated a 4-week treatment of the created virtual subpopulations with enalapril (20 mg), losartan (100 mg) and aliskiren (300 mg). We also considered amlodipine (5 mg), bisoprolol (5 mg) and hydrochlorothiazide (12.5 mg) as additional drugs for combination therapy. For computational experiments at this stage, we used an extension of the primary model, including the pharmacodynamic functions of these drugs at the indicated dosages [65]. Note that in the current study, the bisoprolol and hydrochlorothiazide models were improved to better fit the clinical data (see Materials and Methods for details). As before, therapeutic parameters were estimated for the case of $c_{ACE}$ = 54.1 h^−1^. A detailed analysis of the predicted response of the cardiovascular and renal systems to antihypertensive therapy for this case, as well as confirmation of the predictions with experimental studies, was carried out previously [65]. Therefore, in the current research, we mainly focused on comparing simulation results obtained with different ACE activities.

**Fig. 2:**
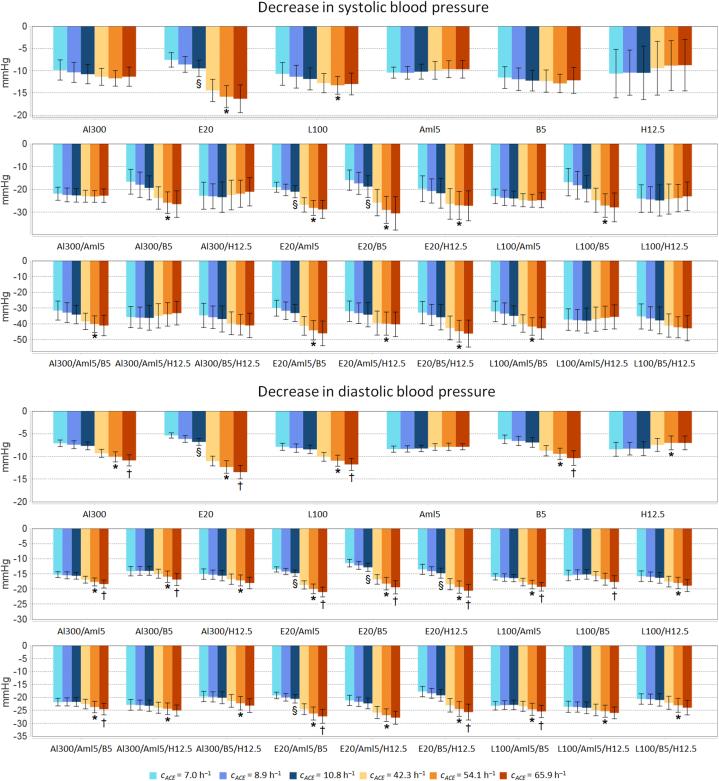
Simulated decrease in systolic and diastolic blood pressure from baseline to week 4 with aliskiren 300 mg (Al300), enalapril 20 mg (E20), losartan 100 mg (L100), amlodipine 5 mg (Aml5), bisoprolol 5 mg (B5) and hydrochlorothiazide 12.5 mg (H12.5), as well as double and triple combinations of these drugs in subpopulations (*n* = 100) with different ACE activity ($c_{ACE}$). *P < 10^−5^*vs*. *LID*-subpopulation ($c_{ACE}$ = 8.9 h^−1^). ^§^*P* < 10^−5^*vs*. *LII* ($c_{ACE}$ = 7.0 h^−1^). ^†^*P* < 10^−5^*vs*. *HII* ($c_{ACE}$ = 42.3 h^−1^). Data are mean ± SD.

Fig. 2 shows a simulated decrease in SBP and DBP with antihypertensive monotherapy and combination therapies. Figs. S10–S35 in Supplementary File 1 demonstrate similar plots for all the physiological characteristics in Table 1 that were significantly altered by the drugs. In addition, Tables S2–S57 (Supplementary File 1) contain numerical data for these characteristics, including values at baseline and after treatment, absolute parameter changes, and *P*-values calculated using the Kolmogorov–Smirnov test for endpoint *vs*. baseline values and parameter changes in subpopulations treated with the same regimens. An interactive script that calculates and visually presents all statistics is implemented online (see Data Availability).

#### 2.3.1 Systemic haemodynamics

All drugs produced a statistically significant (*P* < 10^−5^) drop in SBP and DBP in all subpopulations (Supplementary File 1, Tables S2 and S4). As can be seen from Fig. 2 and Tables S3 and S5 (Supplementary File 1), a statistically greater decrease in SBP for *HID vs*. *LID* was observed for all enalapril-based regimens, losartan alone, and combinations of aliskiren-bisoprolol, losartan-bisoprolol, aliskiren-amlodipine-bisoprolol, and losartan-amlodipine-bisoprolol. In addition, all therapies except amlodipine and the concomitant use of losartan and bisoprolol showed a clear difference in DBP reduction between *HID* and *LID*. RAAS blockers (aliskiren, enalapril and losartan) and bisoprolol, whose ability to suppress renin secretion [75] was reproduced in the model, as well as all double and triple combinations of drugs, were better at lowering DBP with high ACE activity, while hydrochlorothiazide monotherapy showed the opposite trend.

There were no significant differences in SBP reduction between *H*-subpopulations, whereas in population *L*, enalapril-based regimens without hydrochlorothiazide showed a statistically greater decrease in SBP for *LDD* compared to *LII* (*P* < 10^−4^). For DBP, statistical differences between *DD* and *II* variants were more frequently observed in population *H* than in population *L*.

Among single drugs, only bisoprolol led to a significant change (*P* < 10^−4^) in heart rate at all $c_{ACE}$ values (Supplementary File 1, Fig. S10 and Table S6), because its effect was modelled on the fact that selective β1-blockers have a negative chronotropic effect [76]. The model predicted a direct relationship between heart rate reduction with bisoprolol and $c_{ACE}$ values in population *H*, which also showed a significantly greater decrease in heart rate than in population *L* (Supplementary File 1, Table S7). Co-administration of a RAAS blocker and amlodipine increased heart rate, with aliskiren- and losartan-based regimens resulting in a smaller increase with greater ACE activity. Similar dynamics were also observed in dual combinations with hydrochlorothiazide, but the difference between *H*- and *L*-subpopulations for aliskiren- and losartan-based regimens was more pronounced. Interestingly, among dual combinations with bisoprolol, a statistically significant decrease in heart rate was observed only for the case of *LII* (*P* < 10^−4^), while it was practically absent at high levels of ACE activity. All triple drug combinations contributed to the increase in heart rate, which was a consequence of a strong decrease in blood pressure in the model. A significant difference between *HID* and *LID* was found for aliskiren-amlodipine-hydrochlorothiazide, enalapril-amlodipine-bisoprolol, losartan-amlodipine-bisoprolol and losartan-amlodipine-hydrochlorothiazide regimens (Supplementary File 1, Table S7).

In the current research, we extended the pharmacodynamic model of bisoprolol to take into account the decrease in systemic arterial elasticity during treatment (see Materials and Methods). As a result, all bisoprolol-based regimens reduced this variable (*P* < 0.005, Supplementary File 1, Fig. S11 and Table S8). However, there were no statistically significant differences between subpopulations (Supplementary File 1, Table S9).

All medications led to a decrease in systemic vascular resistance (Supplementary File 1, Fig. S12), although in the case of monotherapy, it was weak with averages in the range of 3–9% of the baseline (*P*-values from 0.00025 to 0.37, Supplementary File 1, Table S10). There was a statistically significantly smaller decrease for *HID vs*. *LID* for aliskiren or losartan in double combination with bisoprolol or in triple combination with bisoprolol and amlodipine (*P*_25_ in Supplementary File 1, Table S11). In contrast, enalapril (alone or in combination with amlodipine or hydrochlorothiazide) showed a statistically significantly greater decrease for *HID vs*. *LID*. In addition, enalapril was the only drug that demonstrated a significant difference between *L*-subpopulations (*P*_13_ in Supplementary File 1, Table S11).

#### 2.3.2 Pulmonary haemodynamics

Bisoprolol caused a small but statistically significant increase in diastolic and systolic pulmonary arterial pressure (Supplementary File 1, Figs. S13–S14 and Tables S12–S15). However, dependency appeared with the addition of RAAS blockers, which, in dual combinations with bisoprolol, prevented the increase in pulmonary arterial pressure in *H*-subpopulations, but enhanced it in *L*-subpopulations. Moreover, a significant difference between these two groups of populations remained in triple combinations with bisoprolol.

#### 2.3.3 Left ventricle

The model showed an increase in stroke volume with bisoprolol (Supplementary File 1, Fig. S15). There were no significant differences in this increase between *H*-subpopulations and between *L*-subpopulations. However, a statistically significant smaller increase in stroke volume was found in all *L*-subpopulations *vs*. all *H*-subpopulations (Supplementary File 1, Tables S16–S17). For other monotherapies, no statistically significant changes in stroke volume were observed. The difference in the dynamics for *H*- *vs*. *L*-subpopulations persisted with the co-use of bisoprolol and RAAS blockers, as well as with the subsequent addition of amlodipine, while the addition of hydrochlorothiazide reduced it.

Ejection fraction was significantly decreased in all subpopulations only with triple drug combinations (Supplementary File 1, Fig. S16 and Table S18). For amlodipine-bisoprolol-based regimens, there was a trend towards a higher decrease with increasing $c_{ACE}$ value (*P* < 0.001 for *LII vs*. *LDD*, and *P* < 10^−5^ for *LID vs*. *HID*, Supplementary File 1, Table S19).

The simulated changes in left ventricular end-diastolic pressure for all subpopulations and treatment regimens (Supplementary File 1, Fig. S17 and Tables S20–S21) were similar to those for diastolic pulmonary arterial pressure (Supplementary File 1, Fig. S13 and Tables S12–S13). At the same time, left ventricular peak systolic pressure was closely related to systolic blood pressure, namely, the plots showing the response of these variables to antihypertensive therapy were almost the same (Fig. 2 and Supplementary File 1, Fig. S18), as were the statistical significances of the changes (Supplementary File 1, Tables S2–S3 and S22–S23).

Another relationship demonstrated by the model was observed between left ventricular end-diastolic volume and stroke volume according to the Frank–Starling law [77]. Within physiologic limits, the heart pumps all the blood that returns to it by way of the veins [78]. As venous return increases, end-diastolic volume also increases and, due to the length-tension relationship in the ventricles, stroke volume increases accordingly [79]. Implementation of the Frank–Starling mechanism in the model [65] caused the same changes in end-diastolic volume dynamics (Supplementary File 1, Fig. S19 and Tables S24–S25) as described above for stroke volume. The modelling of bisoprolol in the present study included the negative inotropic effect of β1-blockers [76], which is characterised by a decrease in stroke volume at a given end-diastolic volume. Therefore, bisoprolol-based regimens induced an increase in end-systolic volume (Supplementary File 1, Fig. S20 and Table S26). However, statistically significant differences between subpopulations were observed only for the combination of enalapril, amlodipine and bisoprolol (Supplementary File 1, Table S27).

#### 2.3.4 Right ventricle

The simulated response of right ventricular end-diastolic pressure, peak systolic pressure, end-diastolic and end-systolic volumes to antihypertensive therapy in all subpopulations is presented in Supplementary File 1, Figs. S21–S24 and Tables S28–S35. The dynamics of changes in end-diastolic pressure and volume while varying the value of $c_{ACE}$ were the same as those for the left ventricle. The response of right ventricular peak systolic pressure to treatment was the same as for systolic pulmonary arterial pressure. In addition, end-systolic volume increased with bisoprolol-based regimens due to the negative inotropic effect of the drug. However, in contrast to the left ventricle, there was a statistically significantly greater increase in end-systolic volume for *LID vs*. *HID* during therapy with double and triple combinations of bisoprolol, RAAS blockers and amlodipine (*P* < 0.0005).

#### 2.3.5 Renal function and intraglomerular haemodynamics

As seen in Supplementary File 1, Fig. S25 and Table S36, the glomerular filtration rate remained unchanged with aliskiren, enalapril, losartan, and bisoprolol, but slightly increased with amlodipine-based regimens and slightly decreased with hydrochlorothiazide-based regimens (without amlodipine). However, these changes were not statistically different between subpopulations (Supplementary File 1, Table S37).

In our example, all drugs increased renal blood flow (Supplementary File 1, Fig. S26). However, in the case of single drugs and double combinations with bisoprolol, as well as all double combinations in patients with low ACE activity, this increase tended to be insignificant (Supplementary File 1, Table S38). For almost all drugs, the response of renal blood flow significantly depended on the $c_{ACE}$ value, namely, high ACE activity resulted in a greater increase in this parameter (Supplementary File 1, Table S39).

All antihypertensive regimens contributed to a decrease in afferent and efferent arteriolar resistance and, consequently, to a decrease in renal vascular resistance (Supplementary File 1, Figs. S27–S29 and Tables S40, S42 and S44). RAAS blockers and bisoprolol administered alone resulted in a statistically stronger decrease in these resistances in population *H*, while the opposite trend was observed for hydrochlorothiazide (Supplementary File 1, Tables S41, S43 and S45). Interestingly, in the baseline subpopulations, afferent arteriolar resistance tended to decrease with increasing $c_{ACE}$ values (Table 1). For amlodipine, there were no significant differences between subpopulations. In this regard, the addition of hydrochlorothiazide and amlodipine to RAAS blockers reduced the difference between populations *H* and *L*.

Glomerular hydrostatic pressure was statistically significantly reduced in all treatment regimens, except for monotherapy with amlodipine or hydrochlorothiazide. A direct relationship was observed between the magnitude of the reduction and the value of $c_{ACE}$ for enalapril-based regimens (Supplementary File 1, Fig. S30 and Tables S46–S47).

#### 2.3.6 Biochemical parameters

All blood counts listed in Table 1, except for sodium, are constant in the model. To account for the fact that thiazide diuretics reduce sodium reabsorption [80,81], we previously included the corresponding effect of hydrochlorothiazide in the model [65]. As a result, in the current study, we observed a slight decrease in circulating sodium levels with hydrochlorothiazide-based therapy (Supplementary File 1, Fig. S31 and Table S48). There were no statistically significant differences between subpopulations in these treatment regimens (Supplementary File 1, Table S49).

#### 2.3.7 Renin–angiotensin–aldosterone system

The simulated response of RAAS parameters, including plasma renin activity (PRA), Ang I, Ang II and aldosterone, to antihypertensive therapy was determined by the physiological targets of each class of drugs (see Materials and Methods). Taking cross-treatment effects into account, the model demonstrated the dynamics shown in Supplementary File 1, Figs. S32–S35 and Tables S50, S52, S54 and S56. Aliskiren and all combinations with it reduced PRA, Ang I, Ang II and aldosterone. With low ACE activity, bisoprolol eliminated the increase in PRA and Ang I when used together with enalapril and amlodipine, while other enalapril-based regimens led to a statistically significant increase in these parameters. All combinations with enalapril contributed to the reduction of Ang II and aldosterone. Losartan-based regimens induced a rise in PRA, Ang I, and Ang II and a fall in aldosterone.

All predicted changes in RAAS parameters were highly dependent on the $c_{ACE}$ value (Supplementary File 1, Tables S51, S53, S55 and S57). Moreover, the model showed a positive correlation between baseline levels of PRA, Ang I and Ang II and the DBP reduction for RAAS-acting agents (aliskiren, enalapril, losartan and bisoprolol), with a correlation coefficient that was directly related to ACE activity, i.e. increased along with the $c_{ACE}$ value (Supplementary File 1, Table S58). The same trend persisted for dual combinations of RAAS blockers with amlodipine. However, such a relationship was not observed for aldosterone. For systolic pressure, this trend was also absent (Supplementary File 1, Table S59).

## 3 Discussion

The association between *ACE I/D* polymorphism and cardiovascular disease (particularly hypertension) remains controversial, as does its ability to predict the therapeutic effect of ACE inhibitors. Therefore, the aim of our study was to determine why *ACE I/D* genotypes are associated with the development of hypertension in some ethnic groups but not others using a previously created computational model of the human cardiorenal system. Computational experiments suggested that, firstly, a possible reason for the lack of association between *ACE I/D* and blood pressure levels could be a sufficiently high ACE activity in certain populations, and secondly, the potential decrease in blood pressure with ACE inhibitors is greater in populations without this association than in populations with it. We have not been able to find experimental confirmation or refutation of these hypotheses in the scientific literature, so they require experimental verification. To our knowledge, our study is the first attempt to model the influence of genetic factors on blood pressure regulation.

We also examined the relationship between the *ACE I/D* polymorphism and the success of RAAS inhibitor therapy. To do this, we considered two types of virtual populations of hypertensive patients with low and high ACE activity. Since plasma ACE levels have been reported to increase with the number of *ACE D* alleles, we assigned a specific value for the ACE activity parameter in the model for each genetic variant in both population types. Summarising the above, we can formulate the following model-based conclusions.

1. An increase in ACE activity is accompanied by a rise in circulating Ang II. Under these conditions, maintaining blood pressure at the same level is achieved through the parameters of intraglomerular haemodynamics, namely, through the larger afferent arteriolar diameter, which entails a decrease in the afferent arteriolar resistance and an increase in the glomerular hydrostatic pressure.
2. In ethnic groups with high ACE activity, the association between different *ACE I/D* genotypes and blood pressure levels is erased; however, these groups are generally more susceptible to developing hypertension than the low ACE activity population in which the ACE DD variant becomes a risk factor.
3. The response of cardiovascular and renal parameters to treatment with RAAS blockers directly depends on ACE activity. However, significant variations in parameter changes are observed only with pronounced differences in ACE levels, while the *ACE I/D* polymorphisms within the same ethnic group lead to similar changes in absolute values.

Regarding the latter conclusion, as an example, we note that a statistically significant difference in the effect of the ACE inhibitor enalapril on SBP between *ACE II* and *DD* variants was observed only in the population with low ACE activity (Supplementary File 1, Table S3). However, this difference was about 2 mmHg, i.e. only 1.3 % of the baseline SBP. Thus, our results suggest that any single genetic variant explains only a small fraction of heritability in the success of treating hypertension, which is consistent with similar findings about the development of the disease [82,83]. Nevertheless, one should take into account the fact that the adjustment of the pharmacodynamic parameters of drugs using experimental data was carried out for the case of high ACE activity ($c_{ACE}$ = 54.1 h^−1^). It is possible that refitting these parameters with lower ACE activity (assuming such patients could be recruited for clinical trials) would result in a more significant difference in blood pressure reduction between various *ACE I/D* variants.

The correspondence of the simulated response of cardiovascular and renal parameters to single-drug treatment with experimental observations was carried out in our previous study on modelling antihypertensive therapy [65]. The dynamics of model parameters in the case of double and triple drug combinations in the present research were a consequence of cross-drug effects. However, some findings of the model should be discussed. The model predicted significant reductions in ejection fraction in all subpopulations receiving triple drug combinations (Supplementary File 1, Fig. S16). This finding is unexpected considering the use of RAAS inhibitors and β-blockers as anti-remodelling drugs [[84], [85], [86]]. To clarify this contradiction, we turn to the pathophysiological aspects of cardiac remodelling and the role of the Frank–Starling mechanism in heart failure. The Frank–Starling relationship is an intrinsic property of the myocardium by which increased ventricular volume results in enhanced performance during the subsequent contraction [77,87,88]. Changes in myocardial contractility (or inotropism) affect the Frank–Starling curves [79,89]. Namely, positive inotropic agents cause an increase in left ventricular stroke volume (SV) for a given end-diastolic volume (EDV) and therefore an increase in ejection fraction (EF), which is calculated as:

$$EF=\frac{SV}{EDV}∙100\%\text{.}$$

Negative inotropic agents have the opposite effect [79]. Heart failure is also often associated with impaired contractility [77].

In the current study, we considered virtual patients with uncomplicated essential hypertension. The model showed an increase in SV with bisoprolol (Supplementary File 1, Fig. S15), which is consistent with clinical data for this group of patients [90,91]. Consequently, the inclusion of the Frank–Starling law in the model led to an increase in EDV (Supplementary File 1, Fig. S19). Because bisoprolol has a negative inotropic effect, the SV/EDV ratio decreased, as did EF (although this decrease was only ∼2 % and was not statistically significant; Supplementary File 1, Fig. S16). With the use of other RAAS inhibitors, a slight decrease in EF of ∼1 % was also observed. Thus, with triple combinations of drugs (especially with the inclusion of bisoprolol), these effects were cumulative and the reduction in EF reached averages in the range of 6–10 %, becoming statistically significant in all subpopulations.

In patients with heart failure, bisoprolol reduces left ventricular EDV and practically does not change SV [92,93]. This also corresponds to the Frank–Starling law [65] but leads to bisoprolol improving EF in patients with reduced values of the parameter [92,93]. The ability of the model to simulate the appropriate Frank–Starling curves in patients with heart failure has been previously validated [65]. Therefore, the model is expected to show an improvement in EF for RAAS inhibitors in this group of patients. However, testing this hypothesis is beyond the scope of this study and requires confirmation.

It is also worth noting that the model showed a strong relationship between left ventricular end-diastolic pressure (Supplementary File 1, Fig. S17) and diastolic pulmonary arterial pressure (Supplementary File 1, Fig. S13). This result is consistent with the fact that these values correlate with each other and with pulmonary artery wedge pressure in patients with normal left ventricular function [94,95]. At the same time, changes in end-diastolic pressure and volume of the right ventricle with varying $c_{ACE}$ values were the same as those for the left ventricle (Supplementary File 1, Figs. S17, S19, S21 and S23). This is supported by the fact that left ventricular end-diastolic pressure is highly correlated with right ventricular end-diastolic pressure [[96], [97], [98]], which is also typical for ventricular end-diastolic volumes [99,100].

### 3.1 Limitations of the study

In the present study, we relied on the fact that *ACE I/D* genotypes affect plasma concentration and the activity of ACE and calculated the value of this activity ($c_{ACE}$) relative to the value of chymase activity ($c_{chym}$), which was taken from the model by Hallow et al. [69]. However, *ACE I/D* polymorphisms may also influence the circulating quantity of chymase [101]. This inference was not included in the model. In addition, we did not consider the inactivation of bradykinin by ACE in the model, although the inhibition of the bradykinin binding site on ACE is a promising target for developing antihypertensive drugs [102], since this peptide is a potent endothelium-dependent vasodilator [103].

Also, our model does not consider the long-term cumulative effect of antihypertensive treatment (which is a separate and large task). For drugs that affect the RAAS, treatment is modelled as an acute effect on target parameters. Thus, after activation of the treatment process, the virtual hypertensive patient leaves the equilibrium state and, after 1–2 weeks of model time, returns to a new equilibrium with lower blood pressure values. After this, the values of the model variables stop changing or oscillate with a constant period equal to the length of the cardiac cycle. In the future, it would be interesting to analyse the effect of the *ACE I/D* polymorphism on blood pressure in populations with different ACE activities, taking into account the cumulative effect of therapy and at different times after the start of treatment.

Finally, it should be noted that we investigated the influence of only one of the polymorphic loci that affect the main pathophysiological systems of blood pressure regulation in humans. Due to the polygenic nature of arterial hypertension, the development of this disease is difficult to interpret for individual SNPs. However, our research is an important step towards studying the multiple genetic factors that create a systemic environment for the occurrence of hypertension.

## 4 Conclusion

In the current research, we used a previously created cardiorenal model to examine the association of *ACE I/D* polymorphism with high blood pressure and evaluate the success of RAAS inhibition in the treatment of hypertension depending on *ACE* genotype. The modelled predictions obtained through computational experiments in virtual hypertensive patients with low and high ACE activity allow us to formulate a number of questions for experimental verification.

1. Do populations without an association between *ACE I/D* and blood pressure levels have higher ACE values and a greater susceptibility to developing hypertension compared to populations with it?
2. Do populations with higher ACE levels experience greater reductions in blood pressure when taking ACE inhibitors or not?
3. Is an increase in ACE activity associated with an increase in the diameter (and decreased resistance) of afferent arterioles, as well as with an increase in glomerular hydrostatic pressure?

Clinical studies designed to answer these questions could improve our understanding of the genetic influence of the *ACE I/D* polymorphism on human physiology and would be a natural extension of our theoretical research.

## 5 Materials and Methods

### 5.1 Mathematical model

We used a computational model of the human cardiovascular and renal systems developed by Kutumova et al. [64] and available in the BioModels database [104] with ID MODEL22021600011.^1^ The model is hybrid, i.e., discrete-continuous [105], and consists of a system of ordinary differential equations that includes several discrete events corresponding to instantaneous changes in the simulated dynamics (for example, the transition from systole to diastole). Lists of functions, equations, parameters and variables of the model are given in Supplementary File 2, Tables S1–S4, respectively. The rationale for all formulas and changes made to the baseline models is provided in a supplementary file to our primary study [64].

### 5.2 Equilibrium states

We considered a model to be in equilibrium if all its variables either did not change (e.g. systolic/diastolic blood pressure) or oscillated steadily with an amplitude equal to the duration of the cardiac cycle (e.g. systemic arterial pressure).

### 5.3 Numerical solution of the model

The model covers different time scales from fractions of a second (heart work) to days and weeks (regulation of water and sodium balance). Therefore, we used an agent-based co-simulation methodology [106] to speed up numerical calculations. Briefly, we divided the model equations and events into two sets (modules) representing the functioning of the cardiovascular system (with units in seconds) and the renal system (with units in minutes). In our interpretation, the combination of a module and a suitable numerical solver was an agent. The agents were simulated independently for a given time (several minutes per time step), after which the values of common variables were exchanged. Since the cardiovascular agent quickly reached equilibrium after each exchange, calculations were accelerated by stopping its subsequent simulation until the next exchange. To simulate agents, we used a version of the CVODE solver [107] ported to Java and adapted to the BioUML software interface. For more information, see our agent-based modelling approach [64].

### 5.4 Simulation of antihypertensive treatments

For computational experiments, we used RAAS blockers with different mechanisms of action, including the direct renin inhibitor aliskiren (300 mg), the ACE inhibitor enalapril (20 mg), and the angiotensin II receptor blocker losartan (100 mg). In addition, we considered the following representatives of other classes of antihypertensive medications to analyse their combined action with RAAS inhibitors: the β-blocker bisoprolol (5 mg), the calcium channel blocker amlodipine (5 mg) and the thiazide diuretic hydrochlorothiazide (12.5 mg). An extension of the primary model to account for the effects of these drugs was described previously [65]. Briefly, we identified target variables in the model for all listed medications. The pharmacodynamic effects of therapy were determined by multiplying each variable υ by the influence function:

(2) $$F_{\upsilon }=1\pm E_{\upsilon }∙d_{\upsilon }\text{,}$$

where $E_{\upsilon }$ is the amount of target stimulation (plus sign) or target inhibition (minus sign), and $d_{\upsilon }$ is the drug indicator equal to 1 or 0 depending on whether treatment with this drug is simulated or not. In the case of monotherapy, only one indicator in the model was equal to 1, while in combination therapy, several indicators took on non-zero values.

The pharmacodynamic functions of aliskiren, enalapril, losartan and amlodipine were taken unchanged from the study by Kutumova et al. [65]. We also retained the hydrochlorothiazide targets in the model but re-estimated the kinetic parameters in the influence functions to better fit clinical trials [108]. The pharmacodynamic model of bisoprolol was improved. For this drug, we previously considered a negative chronotropic effect due to blocking the access of catecholamines to their receptors [109] and suppression of the renal secretion of renin [75]. In this study, we additionally took into account the fact that bisoprolol has a negative inotropic effect [76] and reduces arterial stiffness as demonstrated, in particular, by a decrease in pulse wave velocity [[110], [111], [112], [113], [114], [115]]. A complete list of target points for all antihypertensive drugs, including actual $E_{\upsilon }$-values used in formula (2), is provided in Supplementary File 2, Table S5. An extended model comprising all pharmacodynamic functions is available online (see Data Availability).

The effect of each individual drug on the RAAS parameters corresponded to the following pharmacological properties.

• Direct renin inhibitors (aliskiren) provide a reduction in PRA, Ang I and Ang II [116].
• ACE inhibitors (enalapril) lead to an increase in PRA and Ang I and a decrease in Ang II [117].
• Angiotensin II receptor blockers (losartan) stimulate the growth of PRA, Ang I and Ang II [116].
• RAAS inhibitors (aliskiren, enalapril and losartan) can lead to a decrease in plasma aldosterone levels [118].
• Calcium antagonists (amlodipine) do not affect the RAAS parameters [116,[119], [120], [121]].
• β-blockers (bisoprolol) cause a decrease in PRA, Ang I, Ang II and aldosterone [122,123].
• Thiazide diuretics induce activation of the RAAS [124], increasing PRA, Ang I and Ang II [123], while aldosterone levels can increase [125] or not change significantly [126].

### 5.5 Virtual patient

We defined a virtual patient as a single equilibrium parameterisation of the model within physiological ranges. The problem of generating a virtual patient can be formulated as minimising the function of normalised distances between the desired ($X_{i}^{des}$) and simulated equilibrium ($X_{i}$) values of the model variables (blood pressure, heart rate, etc.):

(3) $$f_{dist}\left(X_{1},\ldots ,X_{n}\right)=\sum_{i=1}^{n}\frac{\omega_{\min}}{\omega_{i}}{\left(X_{i}-X_{i}^{des}\right)}^{2},\omega_{i}=X_{i}^{des},\omega_{\min}=\min\;\omega_{i},i=1,\ldots ,n\text{.}$$

A list of fitting parameters, including the limits of the search space in hypertensive and normotensive cases, is presented in Supplementary File 2, Table S6. In addition, we took into account a set of physiological constraints $Y_{j}^{\min}\leq Y_{j}\left(t\right)\leq Y_{j}^{\max}$ for j=1,…,m, which were imposed on the model variables (Supplementary File 2, Table S7), and determined the penalty function of the optimisation problem:

(4) $$f_{penalty}\left(Y_{1},\ldots ,Y_{m}\right)=\sum_{t}\left(\sum_{j=1}^{m}\max\;{\left(0,Y_{j}^{\min}-Y_{j}\left(t\right)\right)}^{2}+\sum_{j=1}^{m}\max\;{\left(0,Y_{j}\left(t\right)-Y_{j}^{\max}\right)}^{2}\right)\text{.}$$

To solve such an optimisation problem, we used a stochastic ranking evolution strategy [127] suitable for constrained global optimisation. To accelerate the calculations, we optimised each of the model agents separately and then combined the resultant equilibrium states.

In order to cut off decisions with unrealistic physiological behaviour, we tested each virtual patient for resistance to an increase in sodium load. The computational experiment consisted of instantly changing the sodium intake (parameter $\Phi_{sodin}$ in the model) from the patient's normal value in the range of 0.028–0.209 mEq/min to the elevated value of 0.243 mEq/min [128], and subsequently observing the dynamics of the model variables. In the case of convergence to a new equilibrium state with SBP increased by no more than 25 mmHg and slightly elevated pulmonary venous pressure, we included the virtual patient in further analyses. Other patients who demonstrated an acute increase in pulmonary venous pressure due to accumulation of blood in the pulmonary veins and, as a result, pulmonary oedema [129], were not taken into consideration.

### 5.6 Virtual hypertensive population

To generate a population of unique virtual hypertensive patients with varying values of SBP, DBP, heart rate (HR), stroke volume (SV), body weight (BW) and body mass index (BMI), we used a function that produces random numbers with the following means and standard deviations:

SBP: 160 ± 10 mmHg; DBP: 100 ± 10 mmHg; HR: 75 ± 10 beats/min;

SV: 70 ± 10 mL; BW: 80 ± 20 kg; BMI: 29 ± 5 kg/m^2^.

Body height (BH) in centimetres was calculated as

$$BH=100∙\sqrt{BW/BMI}\text{.}$$

We considered the following inclusion criteria: essential hypertension (SBP ≥140 mmHg and/or DBP ≥90 mmHg; SBP >130 mmHg; DBP >80 mmHg), BMI >22 kg/m^2^ and height 160–180 cm. Exclusion criteria: severe hypertension (SBP >179.5 mmHg or DBP >109.5 mmHg), abnormal heart rate (HR < 60 or HR > 90 beats/min) and severe obesity (BMI >36 kg/m^2^).

The population generation algorithm included the following steps.

1. Randomly generate SBP, DBP, HR, SV, BW and BMI values using means and standard deviations within the inclusion and exclusion criteria above; select the gender of the patient (male or female) with a probability of 0.5.
2 Substitute the value of BW into the model. Use BW, BH and gender to estimate ranges for baseline total body water (line 50 in Supplementary File 2, Table S6) and total blood volume (line 43 in Supplementary File 2, Table S7).
3. Generate a virtual patient by solving an optimisation problem with fitting parameters from Supplementary File 2, Table S6; objective function (3) defined for the selected values of SBP, DBP, HR and SV; and penalty function (4) determined by constraints from Supplementary File 2, Table S7.
4. If a solution to the optimisation problem is found, check it with the sodium loading test and include it in the final population if the test is successful.

### 5.7 Modelling platform

Here, we used the BioUML software (https://sirius-web.org/bioumlweb/; https://ict.biouml.org/), an open-source Java-based integrated environment for systems biology [66,67] that supports a wide range of bioinformatic formats and mathematical methods for analysing computational models of biological systems, including visual modelling tools.

## Funding

Supported by the 10.13039/501100012190Ministry of Science and Higher Education of the Russian Federation (Agreement 075-10-2021-093, Project CMB-RND-2123).

## Data availability

The data presented in this study can be found in the online repository at https://gitlab.sirius-web.org/virtual-patient/ace-polymorphism-in-cardiorenal-modeling.

## CRediT authorship contribution statement

**Elena Kutumova:** Writing – original draft, Visualization, Validation, Software, Methodology, Investigation, Formal analysis, Data curation. **Anna Kovaleva:** Writing – review & editing. **Ruslan Sharipov:** Writing – review & editing. **Galina Lifshits:** Writing – review & editing, Supervision. **Fedor Kolpakov:** Writing – review & editing, Supervision, Software, Resources, Project administration, Methodology, Funding acquisition.

## Declaration of competing interest

Authors Elena Kutumova, Ruslan Sharipov, and Fedor Kolpakov were employed by Biosoft.Ru, Ltd. The other authors declare that the research was conducted in the absence of any commercial or financial relationships that could be construed as a potential conflict of interest.

## Footnotes

1 https://www.ebi.ac.uk/biomodels/MODEL2202160001.
