# Supplementary material for: Mathematical modelling of the influence of ACE I/D polymorphism on blood pressure and antihypertensive therapy
Source: Heliyon. 2024 Apr 23;10(9):e29988. doi: 10.1016/j.heliyon.2024.e29988 (PMC11068647; doi:10.1016/j.heliyon.2024.e29988)
Supplement: Multimedia component 1 [file mmc1.pdf]

Supplementary File 1 – Model Analysis

Mathematical modeling of the influence of *ACE I/D* polymorphism on blood pressure and antihypertensive therapy

Elena Kutumova*, Anna Kovaleva, Ruslan Sharipov, Galina Lifshits, Fedor Kolpakov

*** Corresponding Author:** elena.kutumova@biouml.org

Figure S1. Distribution of general parameters in baseline virtual hypertensive subpopulations (n = 100)

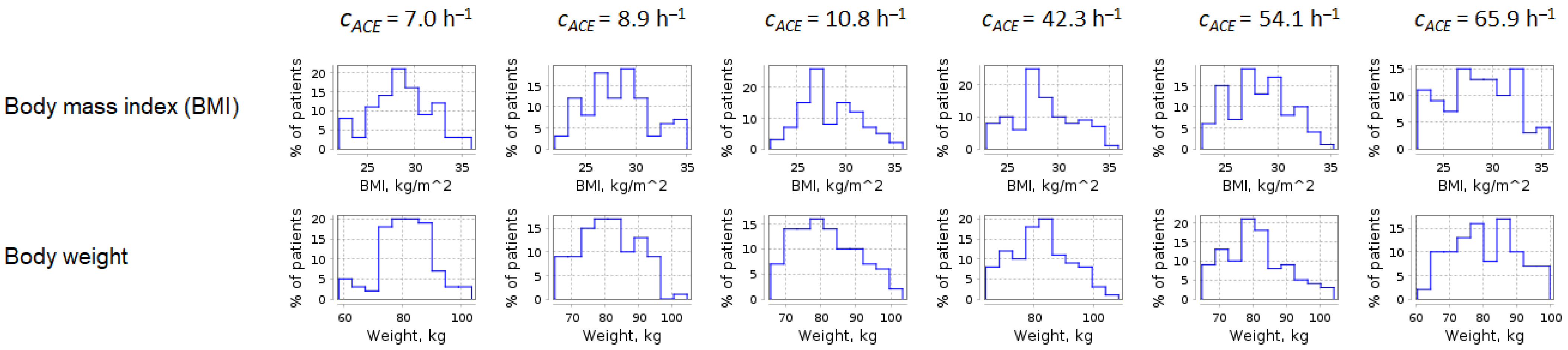

Figure S2. Distribution of systemic hemodynamic parameters in baseline virtual hypertensive subpopulations (n = 100)

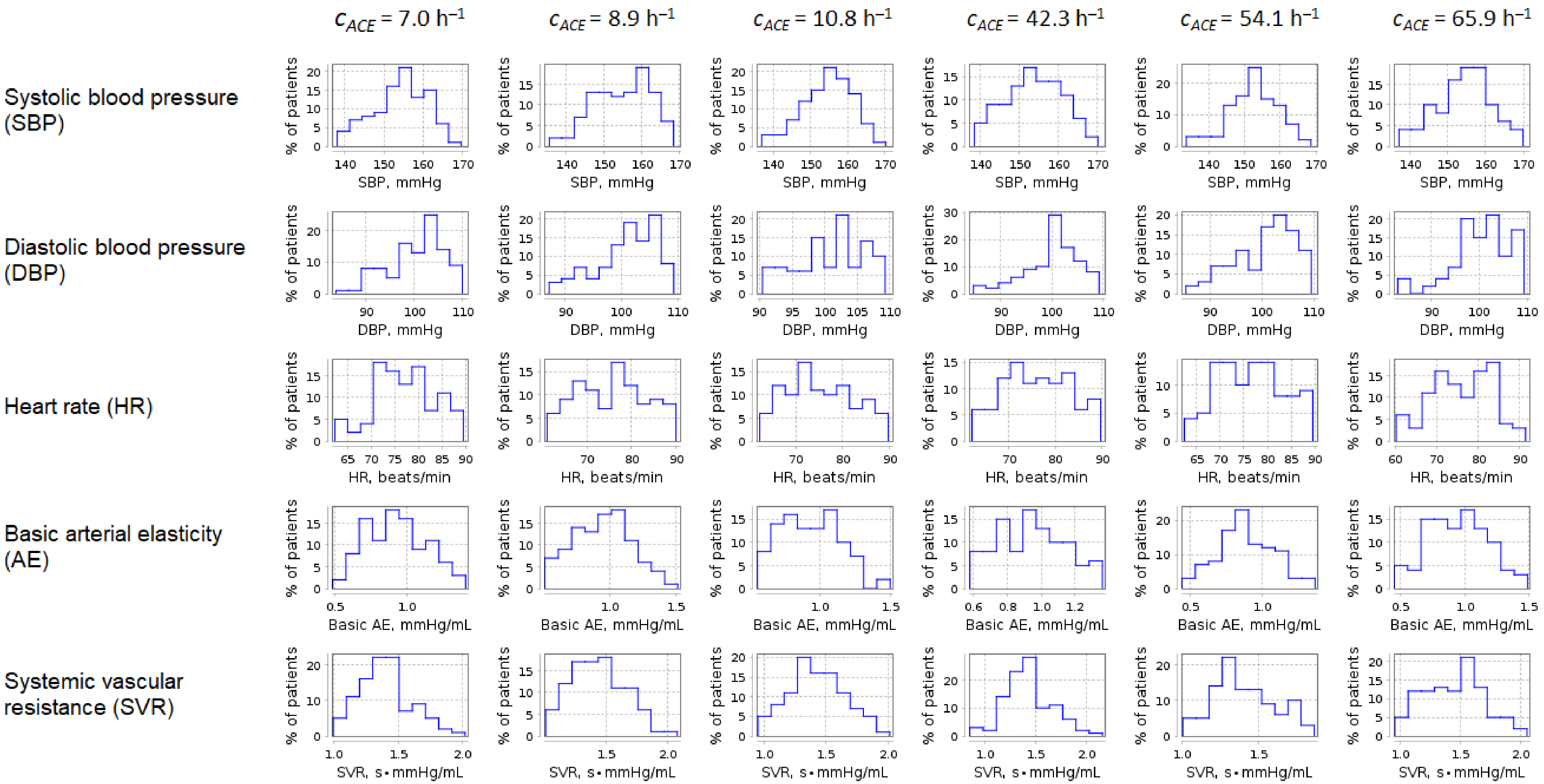

Figure S3. Distribution of pulmonary hemodynamic parameters in baseline virtual hypertensive subpopulations (n = 100)

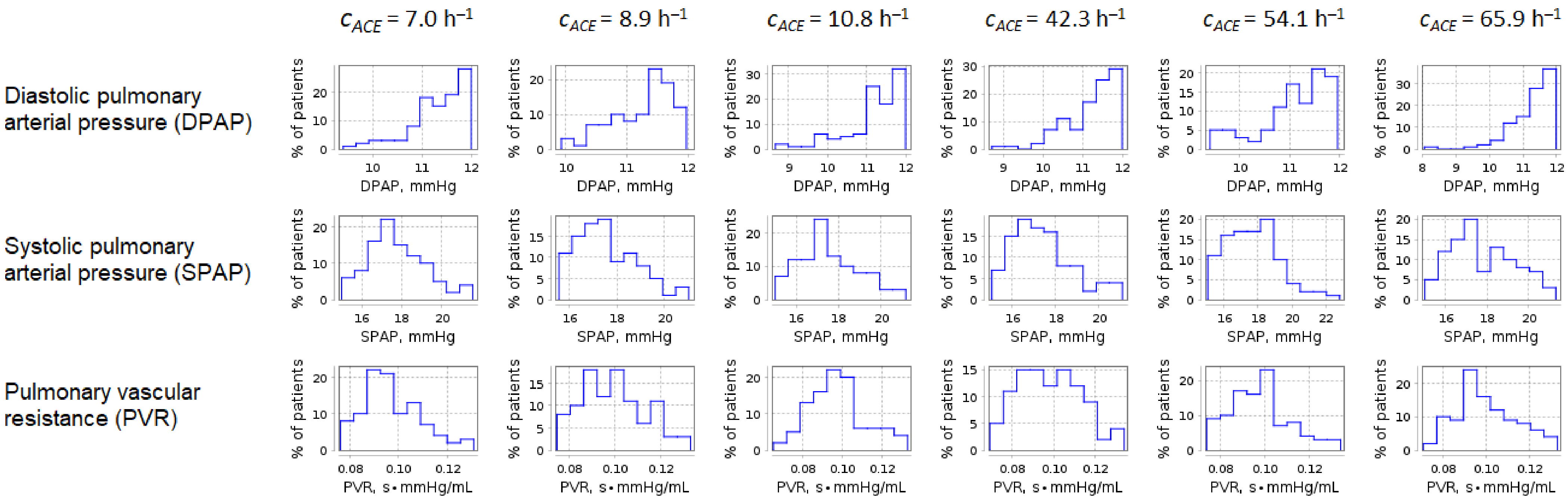

Figure S4. Distribution of left ventricular parameters in baseline virtual hypertensive subpopulations (n = 100)

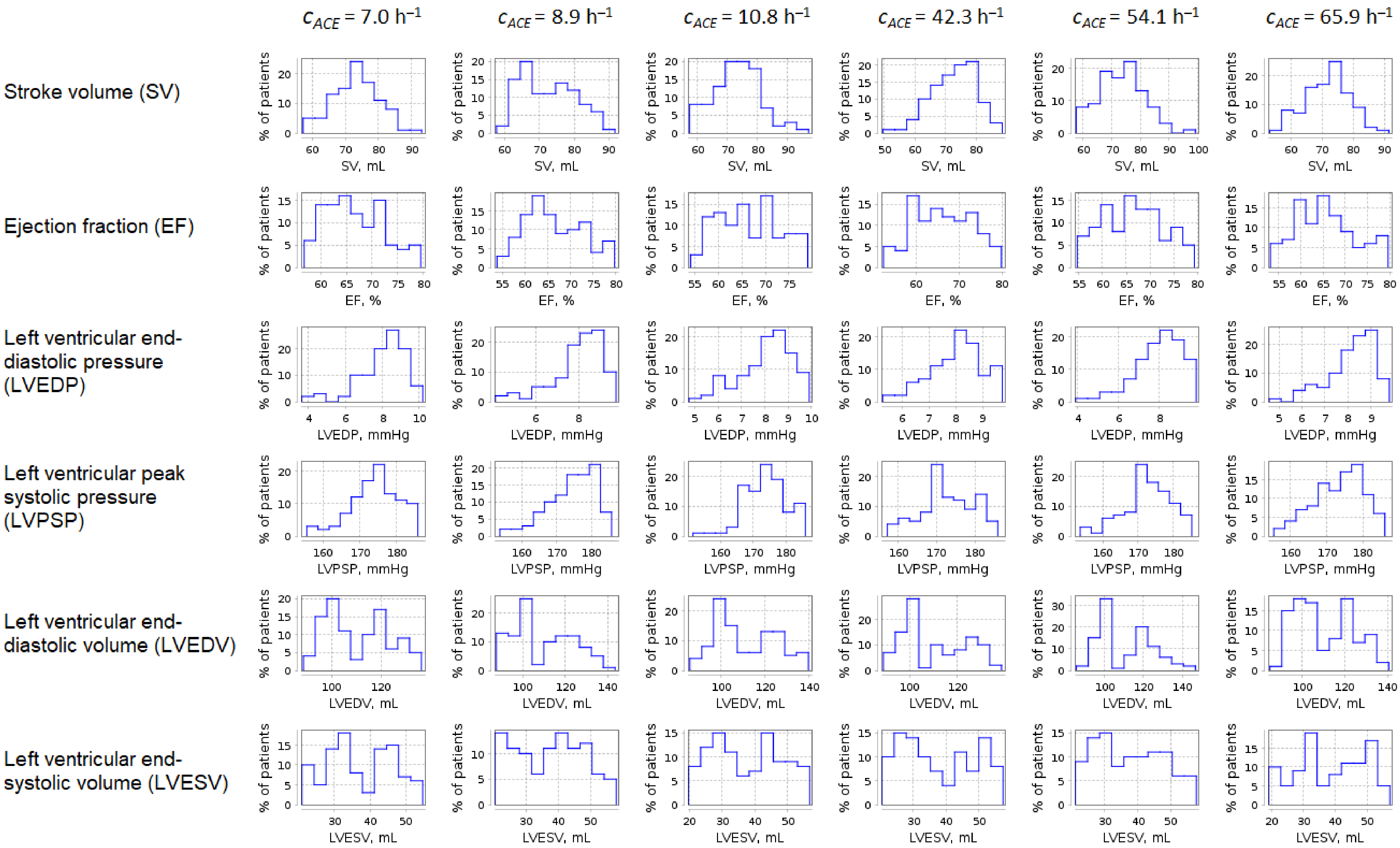

Figure S5. Distribution of right ventricular parameters in baseline virtual hypertensive subpopulations (n = 100)

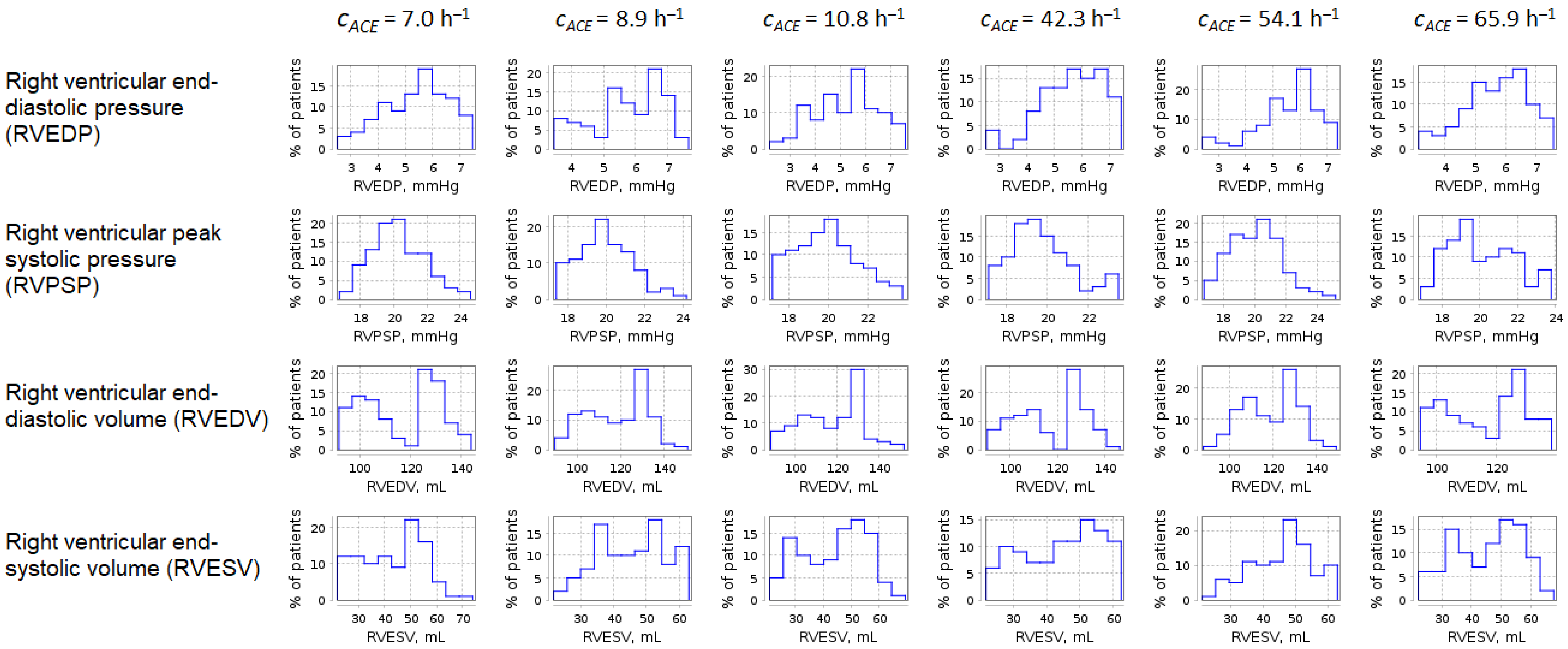

Figure S6. Distribution of renal function parameters in baseline virtual hypertensive subpopulations (n = 100)

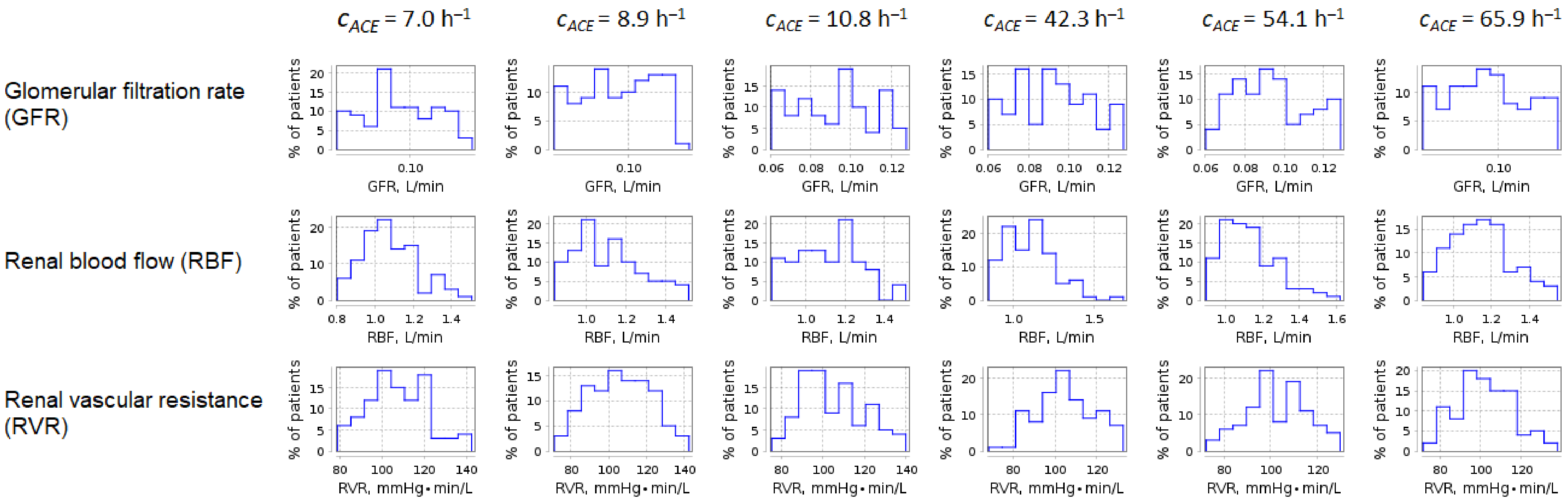

Figure S7. Distribution of intraglomerular hemodynamic parameters in baseline virtual hypertensive subpopulations (n = 100)

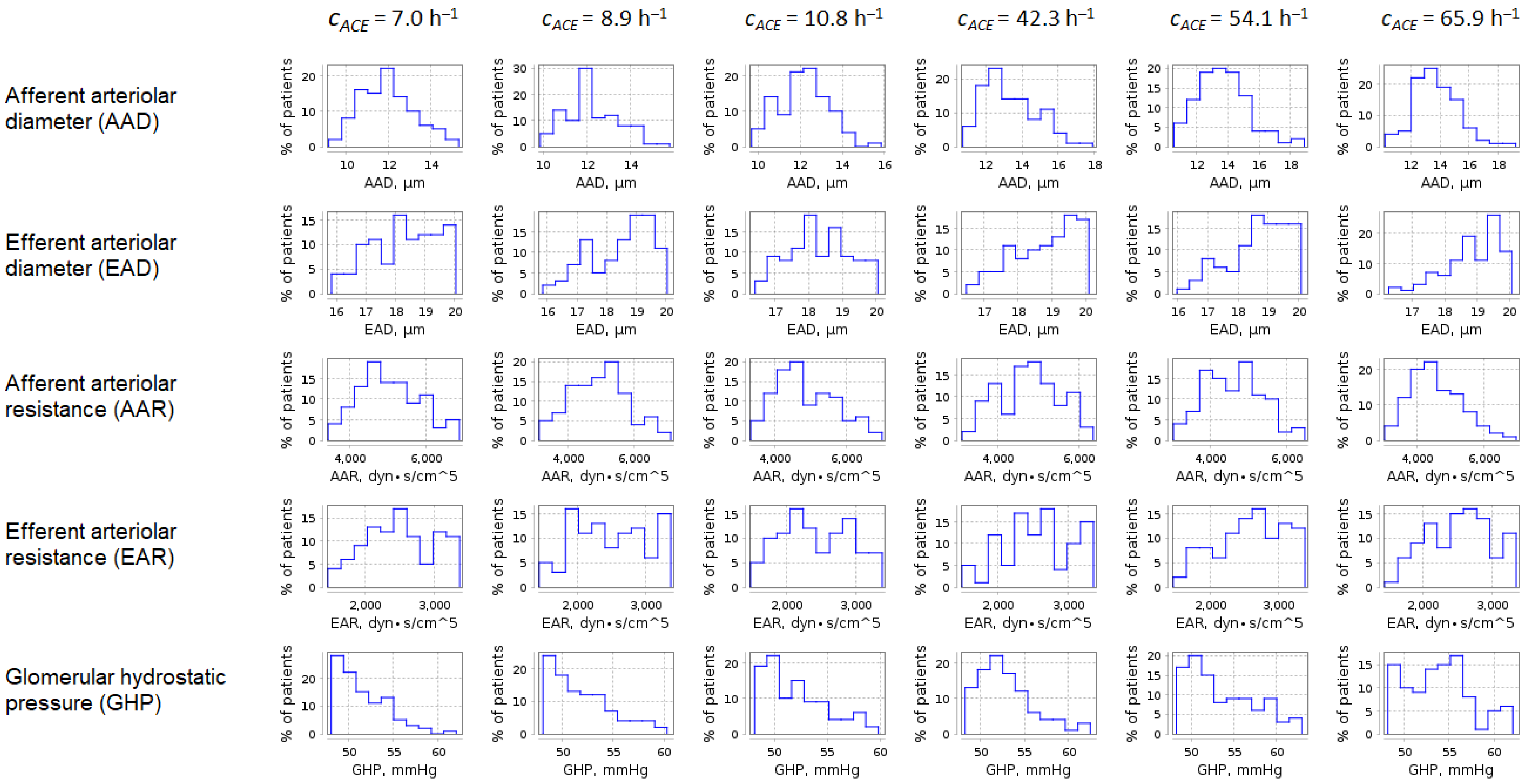

Figure S8. Distribution of blood parameters in baseline virtual hypertensive subpopulations (n = 100)

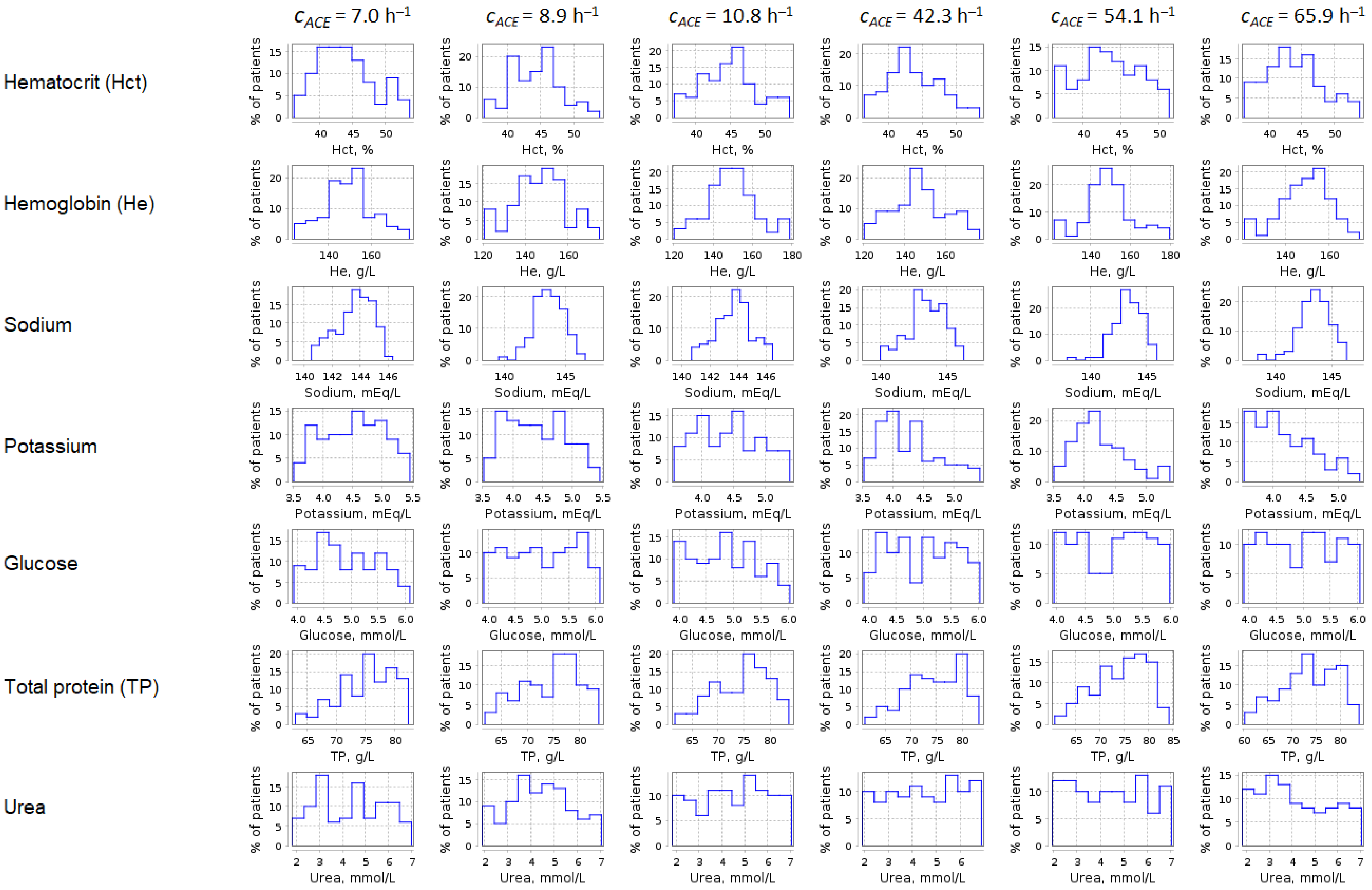

Figure S9. Distribution of renin-angiotensin-aldosterone system parameters in baseline virtual hypertensive subpopulations (n = 100)

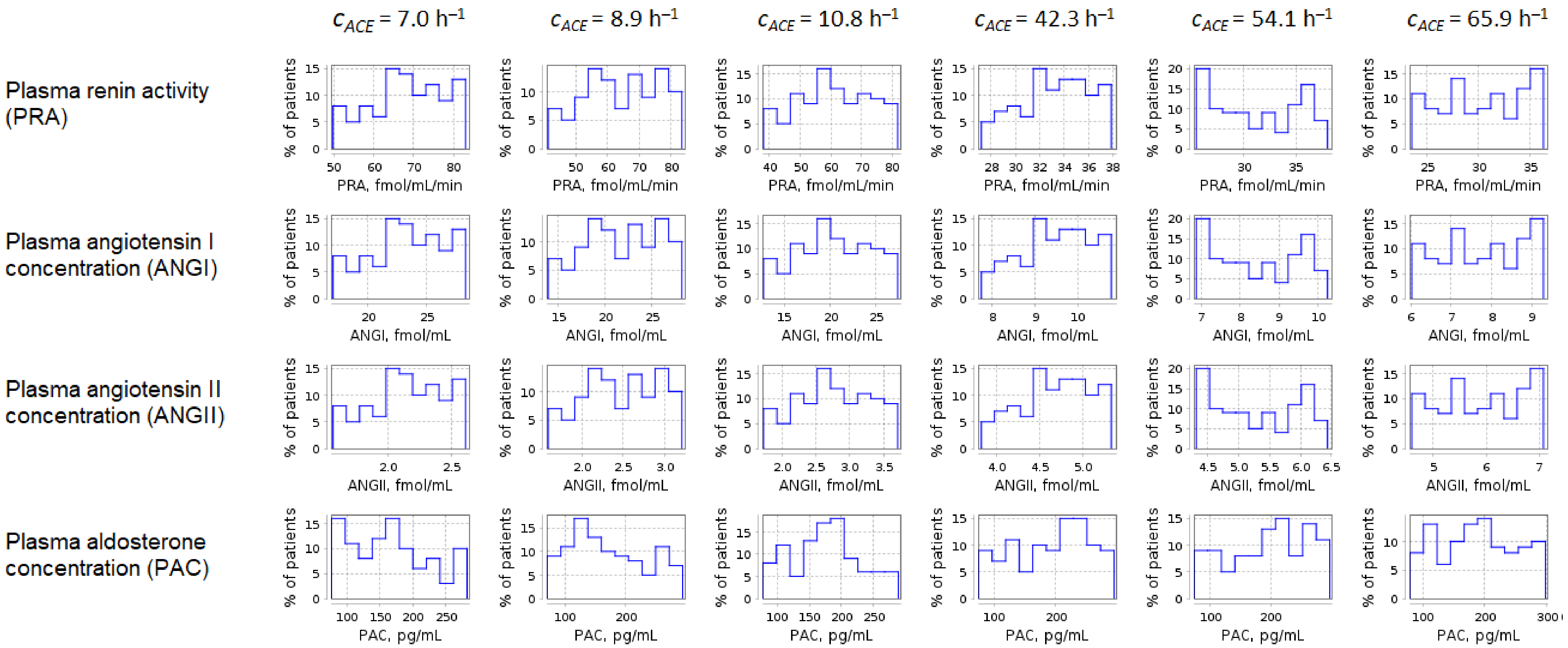

Table S1. *P*-values calculated using the Kolmogorov-Smirnov test for different cases of ACE activity: 7.0 h^{-1} (*LII*-subpopulation, case 1), 8.9 h^{-1} (*LID*, case 2), 10.8 h^{-1} (*LDD*, case 3), 42.3 h^{-1} (*HII*, case 4), 54.1 h^{-1} (*HID*, case 5), and 65.9 h^{-1} (*HDD*, case 6); *P*-value for case *i vs*. case *j* is denoted P_{ij}. Size of subpopulations: n = 100.

Characteristics	***L*-subpopulation *vs.* *L*-subpopulation**			***L*-subpopulation *vs.* *H*-subpopulation**									***H*-subpopulation *vs.* *H*-subpopulation**		
	P_{12}	P_{13}	P_{23}	P_{14}	P_{15}	P_{16}	P_{24}	P_{25}	P_{26}	P_{34}	P_{35}	P_{36}	P_{45}	P_{46}	P_{56}
Body mass index	0.281	0.211	0.699	0.813	0.367	0.813	0.211	0.813	0.367	0.367	0.468	0.367	0.155	0.699	0.367
Body weight	0.581	0.813	0.994	0.468	0.581	0.367	0.468	0.967	0.813	0.581	0.699	0.813	0.367	0.367	0.468
Systolic blood pressure	0.367	0.967	0.367	0.813	0.111	1.000	0.468	0.024	0.367	0.813	0.054	0.699	0.468	0.906	0.111
Diastolic blood pressure	0.581	0.813	0.967	0.367	0.906	0.813	0.155	0.813	0.581	0.281	0.906	0.906	0.281	0.581	0.699
Heart rate	0.024	0.037	0.699	0.111	0.281	0.281	0.813	0.468	0.468	0.699	0.468	0.468	0.906	0.967	0.699
Basic systemic arterial elasticity	0.699	0.906	0.813	0.699	0.468	0.468	0.967	0.078	0.906	0.813	0.367	0.906	0.078	0.699	0.037
Systemic vascular resistance	0.211	0.281	0.813	0.581	0.906	0.006	0.906	0.468	0.281	0.813	0.699	0.211	0.468	0.078	0.078
Diastolic pulmonary arterial pressure	0.468	0.813	0.367	0.367	0.211	0.581	0.581	0.155	0.699	0.967	0.281	0.699	0.468	0.967	0.111
Systolic pulmonary arterial pressure	0.699	0.581	0.906	0.037	0.906	0.813	0.468	0.367	0.281	0.468	0.699	0.281	0.111	0.024	0.906
Pulmonary vascular resistance	0.281	0.906	0.468	0.155	0.813	0.367	0.699	0.699	0.967	0.367	0.813	0.699	0.281	0.211	0.581
Stroke volume	0.155	0.581	0.211	0.699	0.111	0.468	0.367	0.367	0.468	0.994	0.813	0.281	0.699	0.367	0.024
Ejection fraction	0.813	0.581	0.813	0.281	0.468	0.281	0.581	0.813	0.581	0.994	0.967	0.211	0.813	0.367	0.281
Left ventricular end-diastolic pressure	0.699	0.906	1.000	0.468	0.813	0.468	0.699	0.967	0.468	0.581	0.906	0.468	0.906	0.155	0.211
Left ventricular peak systolic pressure	0.581	0.906	0.468	0.078	0.211	0.813	0.054	0.078	0.581	0.581	0.699	0.468	0.906	0.468	0.468
Left ventricular end-diastolic volume	0.581	0.813	0.699	0.967	0.906	1.000	0.906	0.468	0.813	0.994	0.813	0.813	0.699	0.906	0.699
Left ventricular end-systolic volume	0.468	0.699	0.813	0.367	0.813	0.155	0.581	0.906	0.581	0.813	0.906	0.699	0.813	0.581	0.699
Right ventricular end-diastolic pressure	0.054	0.699	0.006	0.468	0.281	0.155	0.581	0.111	0.211	0.054	0.037	0.024	0.813	0.994	0.906
Right ventricular peak systolic pressure	0.468	0.281	0.906	0.078	0.967	0.581	0.155	0.468	0.111	0.468	0.211	0.581	0.078	0.155	0.699
Right ventricular end-diastolic volume	0.468	0.581	0.906	0.906	0.111	0.699	0.813	0.581	0.813	0.967	0.211	0.581	0.281	0.906	0.281
Right ventricular end-systolic volume	0.155	0.994	0.468	0.906	0.155	0.581	0.367	0.813	0.699	0.994	0.367	0.581	0.367	0.813	0.468
Glomerular filtration rate	0.906	0.367	0.211	0.581	0.699	0.994	0.367	0.367	0.906	0.813	0.367	0.468	0.906	0.699	0.581
Renal blood flow	0.367	0.078	0.581	0.211	0.111	0.010	0.581	0.367	0.155	0.367	0.581	0.699	0.367	0.281	0.581
Renal vascular resistance	0.281	0.078	0.581	0.468	0.211	0.024	0.581	0.155	0.111	0.581	0.367	0.367	0.468	0.078	0.468
Afferent arteriolar diameter	0.281	0.367	0.813	SS	SS	SS	SS	SS	SS	SS	SS	SS	0.111	0.024	0.813
Efferent arteriolar diameter	0.111	0.468	0.054	0.010	0.016	SS	0.111	0.581	0.037	0.004	0.024	SS	0.699	0.281	0.281
Afferent arteriolar resistance	0.699	0.281	0.468	0.155	0.016	0.001	0.281	0.024	0.002	0.581	0.211	0.155	0.155	0.037	0.699
Efferent arteriolar resistance	0.813	0.281	0.699	0.581	0.367	0.699	0.155	0.078	0.468	0.054	0.016	0.078	0.906	0.581	0.699
Glomerular hydrostatic pressure	0.967	0.813	1.000	0.010	0.001	SS	0.037	0.010	SS	0.037	0.016	SS	0.078	0.010	0.155
Hematocrit	0.468	0.281	0.967	0.906	0.699	0.967	0.367	0.281	0.281	0.078	0.111	0.111	0.813	0.906	0.813
Hemoglobin	0.281	1.000	0.367	0.367	0.468	0.906	0.813	0.054	0.111	0.468	0.813	0.906	0.211	0.155	0.367
Plasma sodium	0.699	0.813	0.813	0.367	0.281	0.699	0.699	0.468	0.699	0.281	0.211	0.211	0.906	0.994	0.906
Plasma potassium	0.281	0.211	0.906	SS	SS	SS	0.054	0.024	0.024	0.024	0.010	0.024	0.699	0.281	0.155
Plasma glucose	0.468	0.468	0.155	0.813	0.581	0.813	0.967	0.967	0.967	0.211	0.211	0.367	0.967	0.994	0.967
Plasma total protein	0.367	0.581	0.967	0.468	0.367	0.078	0.906	1.000	0.367	0.906	0.994	0.281	0.906	0.813	0.699
Plasma urea	0.468	0.581	0.367	0.699	0.967	0.211	0.281	0.468	0.111	0.994	0.813	0.111	0.906	0.211	0.468
Plasma renin activity	0.024	SS	0.367	SS	SS	SS	SS	SS	SS	SS	SS	SS	SS	SS	0.078
Plasma angiotensin I	0.010	SS	0.281	SS	SS	SS	SS	SS	SS	SS	SS	SS	SS	SS	SS
Plasma angiotensin II	SS	SS	0.001	SS	SS	SS	SS	SS	SS	SS	SS	SS	SS	SS	SS
Plasma aldosterone concentration	0.813	0.281	0.078	0.001	SS	0.037	0.024	0.006	0.078	0.002	0.001	0.111	0.906	0.581	0.581

SS = statistically significant (P < 0.001)

Table S2. Simulated response of <u>systolic blood pressure</u> to antihypertensive therapy in virtual hypertensive subpopulations (*n* = 100) with different ACE activity, including *P*-values (Kolmogorov-Smirnov test) for endpoint *vs*. baseline; data are presented as mean ± SD in mmHg

**Regimens**	***LII*** **(c_{ACE} = 7.0 h^{-1})**			***LID*** **(c_{ACE} = 8.9 h^{-1})**			***LDD*** **(c_{ACE} = 10.8 h^{-1})**			***HII*** **(c_{ACE} = 42.3 h^{-1})**			***HID*** **(c_{ACE} = 54.1 h^{-1})**			***HDD*** **(c_{ACE} = 65.9 h^{-1})**		
	**Value**	**Change**	***P***	**Value**	**Change**	***P***	**Value**	**Change**	***P***	**Value**	**Change**	***P***	**Value**	**Change**	***P***	**Value**	**Change**	***P***
Baseline	154.1 ± 6.9	–	–	154.9 ± 7.4	–	–	154.5 ± 6.5	–	–	153.7 ± 7.2	–	–	152.5 ± 6.8	–	–	154.2 ± 7.2	–	–
Al300	144.2 ± 6.7	-9.8 ± 2.3	SS	144.5 ± 7.6	-10.4 ± 2.3	SS	143.7 ± 6.9	-10.8 ± 2.2	SS	142.4 ± 7.7	-11.4 ± 1.9	SS	140.8 ± 6.9	-11.7 ± 1.7	SS	142.8 ± 7.6	-11.3 ± 2.1	SS
E20	146.5 ± 6.6	-7.6 ± 1.6	SS	146.3 ± 7.5	-8.6 ± 1.8	SS	145.1 ± 6.8	-9.5 ± 1.8	SS	139.3 ± 7.9	-14.5 ± 2.5	SS	136.6 ± 7.0	-15.9 ± 2.5	SS	137.9 ± 7.8	-16.3 ± 3.1	SS
L100	143.3 ± 6.8	-10.7 ± 2.6	SS	143.5 ± 7.8	-11.4 ± 2.6	SS	142.7 ± 7.0	-11.9 ± 2.5	SS	140.9 ± 7.8	-12.8 ± 2.2	SS	139.2 ± 7.0	-13.3 ± 2.0	SS	141.2 ± 7.7	-13.0 ± 2.4	SS
Aml5	143.6 ± 6.6	-10.4 ± 1.2	SS	144.4 ± 7.2	-10.5 ± 1.4	SS	144.3 ± 6.3	-10.2 ± 1.7	SS	143.8 ± 7.2	-9.9 ± 2.0	SS	142.8 ± 6.1	-9.7 ± 1.9	SS	144.5 ± 6.9	-9.7 ± 2.0	SS
B5	142.5 ± 7.0	-11.5 ± 2.5	SS	143.0 ± 7.9	-11.9 ± 2.5	SS	142.3 ± 6.9	-12.2 ± 2.3	SS	141.4 ± 8.1	-12.3 ± 2.5	SS	139.6 ± 7.1	-12.9 ± 2.1	SS	142.0 ± 8.2	-12.2 ± 2.9	SS
H12.5	143.4 ± 8.0	-10.7 ± 5.4	SS	144.4 ± 8.9	-10.4 ± 5.1	SS	144.0 ± 8.5	-10.5 ± 6.0	SS	144.3 ± 9.6	-9.5 ± 6.0	SS	143.6 ± 8.0	-8.9 ± 5.6	SS	145.4 ± 8.1	-8.8 ± 5.8	SS
Al300<br>Aml5	132.2 ± 6.6	-21.9 ± 2.9	SS	132.5 ± 7.8	-22.4 ± 3.0	SS	131.9 ± 6.9	-22.6 ± 2.9	SS	130.8 ± 7.7	-23.0 ± 2.6	SS	129.4 ± 6.5	-23.1 ± 2.6	SS	131.5 ± 7.5	-22.7 ± 3.0	SS
Al300<br>B5	137.5 ± 9.0	-16.6 ± 5.4	SS	137.0 ± 9.8	-17.9 ± 5.5	SS	135.2 ± 8.8	-19.3 ± 5.3	SS	130.0 ± 9.2	-23.7 ± 4.9	SS	126.7 ± 8.0	-25.8 ± 4.7	SS	127.8 ± 9.1	-26.4 ± 5.8	SS
Al300<br>H12.5	131.3 ± 7.7	-22.8 ± 5.9	SS	131.9 ± 9.0	-23.0 ± 5.5	SS	131.2 ± 9.0	-23.4 ± 6.7	SS	131.3 ± 10.1	-22.4 ± 6.5	SS	130.6 ± 8.2	-21.9 ± 5.9	SS	133.2 ± 8.6	-21.0 ± 6.2	SS
E20<br>Aml5	135.0 ± 6.5	-19.0 ± 2.2	SS	134.7 ± 7.6	-20.2 ± 2.5	SS	133.5 ± 6.8	-21.0 ± 2.6	SS	127.0 ± 7.9	-26.8 ± 3.2	SS	124.4 ± 6.7	-28.1 ± 3.3	SS	125.4 ± 7.6	-28.7 ± 3.9	SS
E20<br>B5	138.1 ± 8.1	-15.9 ± 4.4	SS	137.5 ± 9.2	-17.4 ± 4.8	SS	135.8 ± 8.4	-18.8 ± 4.7	SS	128.0 ± 9.8	-25.8 ± 5.8	SS	123.5 ± 8.7	-29.0 ± 5.9	SS	123.7 ± 10.1	-30.5 ± 7.3	SS
E20<br>H12.5	134.4 ± 7.7	-19.7 ± 5.6	SS	134.2 ± 8.9	-20.7 ± 5.3	SS	132.9 ± 8.9	-21.7 ± 6.5	SS	127.4 ± 10.2	-26.3 ± 6.7	SS	125.5 ± 8.2	-27.0 ± 6.1	SS	127.0 ± 8.7	-27.1 ± 6.5	SS
L100<br>Aml5	131.1 ± 6.8	-23.0 ± 3.2	SS	131.2 ± 7.9	-23.7 ± 3.4	SS	130.6 ± 7.1	-24.0 ± 3.2	SS	129.0 ± 7.8	-24.7 ± 2.8	SS	127.6 ± 6.6	-24.9 ± 2.8	SS	129.5 ± 7.5	-24.7 ± 3.2	SS
L100<br>B5	137.2 ± 9.5	-16.8 ± 6.0	SS	136.7 ± 10.3	-18.2 ± 6.1	SS	134.8 ± 9.2	-19.7 ± 5.7	SS	129.0 ± 9.4	-24.7 ± 5.3	SS	125.5 ± 8.2	-27.0 ± 5.1	SS	126.3 ± 9.4	-27.8 ± 6.3	SS
L100<br>H12.5	130.1 ± 7.8	-24.0 ± 6.0	SS	130.5 ± 9.1	-24.4 ± 5.6	SS	129.7 ± 9.1	-24.8 ± 6.8	SS	129.5 ± 10.1	-24.2 ± 6.6	SS	128.7 ± 8.2	-23.8 ± 6.0	SS	131.2 ± 8.7	-23.0 ± 6.3	SS
Al300<br>Aml5/B5	122.4 ± 9.0	-31.6 ± 6.0	SS	121.9 ± 10.1	-33.0 ± 6.3	SS	120.3 ± 9.0	-34.2 ± 5.9	SS	115.2 ± 8.9	-38.5 ± 5.2	SS	112.3 ± 7.6	-40.2 ± 5.2	SS	113.1 ± 8.9	-41.1 ± 6.5	SS
Al300<br>Aml5/H12.5	118.3 ± 7.9	-35.8 ± 6.7	SS	118.8 ± 9.6	-36.1 ± 6.7	SS	118.3 ± 9.7	-36.3 ± 7.8	SS	118.7 ± 10.6	-35.0 ± 7.7	SS	118.4 ± 8.8	-34.1 ± 7.4	SS	120.9 ± 9.0	-33.3 ± 7.5	SS
Al300<br>B5/H12.5	119.4 ± 8.9	-34.7 ± 7.7	SS	119.0 ± 10.1	-35.9 ± 7.1	SS	117.5 ± 10.1	-37.0 ± 8.3	SS	114.0 ± 10.1	-39.7 ± 7.2	SS	111.9 ± 7.9	-40.6 ± 6.5	SS	113.1 ± 9.0	-41.1 ± 7.7	SS
E20<br>Aml5/B5	123.9 ± 8.0	-30.1 ± 5.0	SS	123.1 ± 9.5	-31.8 ± 5.6	SS	121.3 ± 8.5	-33.2 ± 5.4	SS	112.4 ± 9.4	-41.3 ± 6.0	SS	108.3 ± 8.2	-44.2 ± 6.1	SS	108.1 ± 9.7	-46.1 ± 7.8	SS
E20<br>Aml5/H12.5	122.0 ± 7.9	-32.1 ± 6.5	SS	121.6 ± 9.5	-33.3 ± 6.5	SS	120.3 ± 9.6	-34.2 ± 7.7	SS	114.2 ± 10.4	-39.6 ± 7.6	SS	112.6 ± 8.5	-39.9 ± 7.3	SS	113.8 ± 8.8	-40.4 ± 7.8	SS
E20<br>B5/H12.5	121.1 ± 8.3	-32.9 ± 7.1	SS	120.5 ± 9.7	-34.4 ± 6.7	SS	118.7 ± 9.9	-35.9 ± 8.0	SS	111.0 ± 10.3	-42.7 ± 7.6	SS	107.8 ± 8.0	-44.7 ± 6.9	SS	107.9 ± 9.4	-46.2 ± 8.6	SS
L100<br>Aml5/B5	121.9 ± 9.5	-32.2 ± 6.5	SS	121.3 ± 10.6	-33.6 ± 6.8	SS	119.5 ± 9.4	-35.0 ± 6.3	SS	113.9 ± 9.1	-39.8 ± 5.5	SS	110.7 ± 7.8	-41.8 ± 5.6	SS	111.3 ± 9.2	-42.9 ± 6.9	SS
L100<br>Aml5/H12.5	116.7 ± 7.9	-37.4 ± 6.8	SS	117.1 ± 9.6	-37.8 ± 6.8	SS	116.5 ± 9.7	-38.0 ± 7.9	SS	116.6 ± 10.5	-37.1 ± 7.7	SS	116.3 ± 8.7	-36.2 ± 7.4	SS	118.6 ± 8.9	-35.6 ± 7.6	SS
L100<br>B5/H12.5	118.7 ± 9.2	-35.4 ± 8.0	SS	118.2 ± 10.4	-36.6 ± 7.4	SS	116.7 ± 10.4	-37.9 ± 8.6	SS	112.6 ± 10.2	-41.1 ± 7.4	SS	110.3 ± 7.9	-42.2 ± 6.7	SS	111.3 ± 9.1	-42.9 ± 8.0	SS

Al300 = aliskiren 300 mg; **Aml5** = amlodipine 5 mg; **B5** = bisoprolol 5 mg; **E20** = enalapril 20 mg; **H12.5** = hydrochlorothiazide 12.5 mg; **L100** = losartan 100 mg; **SS** = statistically significant (*P* < 0.00001)

Table S3. *P*-values calculated using the Kolmogorov-Smirnov test for changes in systolic blood pressure in subpopulations (n = 100) with different ACE activity receiving the same regimens. Case 1: c_{ACE} = 7.0 h^{-1} (*LII*), case 2: c_{ACE} = 8.9 h^{-1} (*LID*), case 3: c_{ACE} = 10.8 h^{-1} (*LDD*), case 4: c_{ACE} = 42.3 h^{-1} (*HII*), case 5: c_{ACE} = 54.1 h^{-1} (*HID*), case 6: c_{ACE} = 65.9 h^{-1} (*HDD*). *P*-value for case *i vs*. case *j* is denoted P_{ij}.

Regimens	P_{12}	P_{13}	P_{23}	P_{14}	P_{15}	P_{16}	P_{24}	P_{25}	P_{26}	P_{34}	P_{35}	P_{36}	P_{45}	P_{46}	P_{56}
Al300	0.21055	0.01581	0.28093	0.00002	SS	SS	0.00232	0.00007	0.00386	0.11113	0.00232	0.07832	0.21055	0.69937	0.21055
E20	0.00079	SS	0.03663	SS	SS	SS	SS	SS	SS	SS	SS	SS	0.00079	0.00013	0.07832
L100	0.11113	0.00630	0.36672	SS	SS	SS	0.00045	SS	0.00007	0.02431	0.00025	0.01008	0.15454	0.69937	0.36672
Aml5	0.81275	0.46756	0.69937	0.11113	0.00136	0.00232	0.07832	0.00045	0.00386	0.36672	0.00386	0.03663	0.11113	0.15454	0.96707
B5	0.46756	0.07832	0.21055	0.07832	0.00232	0.07832	0.28093	0.05410	0.36672	0.96707	0.15454	0.81275	0.36672	0.69937	0.11113
H12.5	0.58062	0.69937	0.36672	0.46756	0.15454	0.05410	0.28093	0.01581	0.00232	0.28093	0.05410	0.01581	0.36672	0.36672	0.96707
Al300<br>Aml5	0.11113	0.01581	0.81275	0.00232	0.01581	0.01008	0.21055	0.46756	0.36672	0.28093	0.58062	0.90621	0.81275	0.69937	0.96707
Al300<br>B5	0.21055	0.00045	0.15454	SS	SS	SS	SS	SS	SS	0.00004	SS	SS	0.00630	0.02431	0.46756
Al300<br>H12.5	0.81275	0.07832	0.58062	0.81275	0.46756	0.07832	0.36672	0.11113	0.02431	0.46756	0.02431	0.00630	0.36672	0.15454	0.69937
E20<br>Aml5	0.00045	SS	0.05410	SS	SS	SS	SS	SS	SS	SS	SS	SS	0.11113	0.00232	0.15454
E20<br>B5	0.05410	SS	0.07832	SS	SS	SS	SS	SS	SS	SS	SS	SS	0.00232	0.00045	0.11113
E20<br>H12.5	0.36672	0.00386	0.07832	SS	SS	SS	SS	SS	SS	0.00004	SS	SS	0.69937	0.36672	0.81275
L100<br>Aml5	0.07832	0.00630	0.69937	0.00013	0.00013	0.00007	0.03663	0.03663	0.02431	0.07832	0.07832	0.28093	0.81275	0.90621	0.81275
L100<br>B5	0.21055	0.00136	0.15454	SS	SS	SS	SS	SS	SS	0.00004	SS	SS	0.00630	0.01008	0.36672
L100<br>H12.5	0.36672	0.15454	0.36672	0.46756	0.96707	0.58062	0.46756	0.21055	0.15454	0.58062	0.11113	0.07832	0.58062	0.28093	0.90621
Al300<br>Aml5/B5	0.07832	0.00232	0.28093	SS	SS	SS	SS	SS	SS	0.00004	SS	SS	0.11113	0.00386	0.15454
Al300<br>Aml5/H12.5	0.58062	0.58062	0.58062	0.58062	0.21055	0.11113	0.36672	0.05410	0.02431	0.21055	0.03663	0.02431	0.58062	0.15454	0.36672
Al300<br>B5/H12.5	0.21055	0.05410	0.28093	0.00004	SS	SS	0.01581	0.00079	0.00013	0.02431	0.00386	0.01008	0.46756	0.15454	0.69937
E20<br>Aml5/B5	0.01581	0.00002	0.21055	SS	SS	SS	SS	SS	SS	SS	SS	SS	0.01581	0.00045	0.07832
E20<br>Aml5/H12.5	0.15454	0.02431	0.21055	SS	SS	SS	SS	SS	SS	0.00079	0.00013	SS	0.90621	0.36672	0.58062
E20<br>B5/H12.5	0.07832	0.01581	0.15454	SS	SS	SS	SS	SS	SS	0.00002	SS	SS	0.21055	0.00232	0.21055
L100<br>Aml5/B5	0.07832	0.00232	0.28093	SS	SS	SS	SS	SS	SS	0.00002	SS	SS	0.11113	0.00386	0.15454
L100<br>Aml5/H12.5	0.46756	0.46756	0.58062	0.90621	0.28093	0.36672	0.58062	0.07832	0.11113	0.58062	0.15454	0.07832	0.69937	0.21055	0.46756
L100<br>B5/H12.5	0.21055	0.02431	0.15454	SS	SS	SS	0.00232	0.00013	0.00002	0.01581	0.00079	0.00232	0.46756	0.05410	0.69937

Al300 = aliskiren 300 mg; **Aml5** = amlodipine 5 mg; **B5** = bisoprolol 5 mg; **E20** = enalapril 20 mg; **H12.5** = hydrochlorothiazide 12.5 mg; **L100** = losartan 100 mg; **SS** = statistically significant (P < 0.00001)

Table S4. Simulated response of <u>diastolic blood pressure</u> to antihypertensive therapy in virtual hypertensive subpopulations (n = 100) with different ACE activity, including P-values (Kolmogorov-Smirnov test) for endpoint *vs*. baseline; data are presented as mean ± SD in mmHg

**Regimens**	***LII*** **(c_{ACE} = 7.0 h^{-1})**			***LID*** **(c_{ACE} = 8.9 h^{-1})**			***LDD*** **(c_{ACE} = 10.8 h^{-1})**			***HII*** **(c_{ACE} = 42.3 h^{-1})**			***HID*** **(c_{ACE} = 54.1 h^{-1})**			***HDD*** **(c_{ACE} = 65.9 h^{-1})**		
	**Value**	**Change**	***P***	**Value**	**Change**	***P***	**Value**	**Change**	***P***	**Value**	**Change**	***P***	**Value**	**Change**	***P***	**Value**	**Change**	***P***
Baseline	100.5 ± 5.6	–	–	101.0 ± 5.3	–	–	100.9 ± 5.1	–	–	100.1 ± 5.3	–	–	100.5 ± 5.8	–	–	100.5 ± 5.9	–	–
Al300	93.4 ± 5.4	-7.1 ± 0.7	SS	93.5 ± 5.1	-7.4 ± 0.9	SS	93.3 ± 4.7	-7.7 ± 0.9	SS	90.8 ± 5.1	-9.3 ± 0.9	SS	90.4 ± 5.4	-10.1 ± 1.1	SS	89.6 ± 5.8	-10.9 ± 1.2	SS
E20	95.1 ± 5.5	-5.4 ± 0.6	SS	94.8 ± 5.1	-6.2 ± 0.7	SS	94.1 ± 4.8	-6.8 ± 0.8	SS	89.1 ± 5.0	-11.0 ± 1.1	SS	88.2 ± 5.3	-12.3 ± 1.4	SS	87.0 ± 5.8	-13.5 ± 1.5	SS
L100	92.6 ± 5.4	-7.9 ± 0.8	SS	92.8 ± 5.1	-8.2 ± 0.9	SS	92.5 ± 4.7	-8.5 ± 0.9	SS	90.0 ± 5.1	-10.1 ± 1.0	SS	89.5 ± 5.4	-11.0 ± 1.2	SS	88.7 ± 5.8	-11.8 ± 1.3	SS
Aml5	92.1 ± 5.2	-8.4 ± 0.7	SS	92.6 ± 5.0	-8.3 ± 0.7	SS	92.7 ± 4.7	-8.3 ± 0.7	SS	92.2 ± 4.9	-7.9 ± 0.7	SS	92.6 ± 5.4	-7.9 ± 0.8	SS	92.6 ± 5.6	-7.9 ± 0.7	SS
B5	94.2 ± 5.3	-6.2 ± 0.9	SS	94.4 ± 5.0	-6.6 ± 1.0	SS	94.0 ± 4.6	-6.9 ± 1.1	SS	91.3 ± 5.0	-8.8 ± 1.1	SS	91.0 ± 5.3	-9.4 ± 1.2	SS	90.1 ± 5.7	-10.4 ± 1.6	SS
H12.5	92.0 ± 5.4	-8.4 ± 1.5	SS	92.7 ± 5.2	-8.3 ± 1.4	SS	92.7 ± 5.0	-8.3 ± 1.5	SS	92.6 ± 5.0	-7.5 ± 1.4	SS	93.5 ± 5.5	-7.0 ± 1.5	SS	93.5 ± 5.6	-7.0 ± 1.5	SS
Al300<br>Aml5	85.2 ± 5.2	-15.3 ± 1.0	SS	85.5 ± 4.9	-15.5 ± 1.2	SS	85.3 ± 4.5	-15.6 ± 1.1	SS	83.2 ± 4.8	-16.9 ± 1.1	SS	82.8 ± 5.1	-17.7 ± 1.3	SS	82.1 ± 5.7	-18.4 ± 1.3	SS
Al300<br>B5	86.4 ± 5.4	-14.1 ± 1.6	SS	87.0 ± 5.1	-14.0 ± 1.5	SS	86.9 ± 4.6	-14.0 ± 1.5	SS	85.2 ± 4.9	-14.9 ± 1.5	SS	84.5 ± 5.1	-15.9 ± 1.9	SS	83.6 ± 5.7	-16.9 ± 2.0	SS
Al300<br>H12.5	85.4 ± 5.4	-15.1 ± 1.7	SS	85.7 ± 5.0	-15.3 ± 1.5	SS	85.3 ± 4.8	-15.6 ± 1.7	SS	83.3 ± 4.9	-16.8 ± 1.7	SS	83.3 ± 5.2	-17.2 ± 1.8	SS	82.5 ± 5.7	-18.0 ± 1.9	SS
E20<br>Aml5	86.9 ± 5.2	-13.5 ± 0.9	SS	86.8 ± 4.9	-14.2 ± 1.0	SS	86.2 ± 4.5	-14.8 ± 1.0	SS	81.4 ± 4.8	-18.7 ± 1.4	SS	80.5 ± 5.1	-20.0 ± 1.6	SS	79.4 ± 5.8	-21.0 ± 1.6	SS
E20<br>B5	89.0 ± 5.3	-11.4 ± 1.3	SS	88.8 ± 5.0	-12.2 ± 1.3	SS	88.1 ± 4.5	-12.8 ± 1.4	SS	83.3 ± 4.9	-16.8 ± 1.6	SS	82.2 ± 5.1	-18.3 ± 2.1	SS	81.0 ± 5.8	-19.4 ± 2.2	SS
E20<br>H12.5	87.0 ± 5.4	-13.5 ± 1.7	SS	86.9 ± 5.0	-14.1 ± 1.5	SS	86.2 ± 4.8	-14.8 ± 1.7	SS	81.7 ± 4.9	-18.4 ± 1.9	SS	81.1 ± 5.1	-19.4 ± 2.0	SS	79.9 ± 5.7	-20.6 ± 2.1	SS
L100<br>Aml5	84.5 ± 5.2	-16.0 ± 1.0	SS	84.7 ± 4.9	-16.2 ± 1.2	SS	84.5 ± 4.5	-16.4 ± 1.2	SS	82.4 ± 4.8	-17.7 ± 1.2	SS	81.9 ± 5.1	-18.5 ± 1.4	SS	81.2 ± 5.7	-19.3 ± 1.4	SS
L100<br>B5	84.9 ± 5.4	-15.5 ± 1.7	SS	85.7 ± 5.1	-15.3 ± 1.6	SS	85.8 ± 4.6	-15.2 ± 1.6	SS	84.3 ± 4.9	-15.8 ± 1.5	SS	83.7 ± 5.1	-16.8 ± 2.0	SS	82.8 ± 5.7	-17.7 ± 2.1	SS
L100<br>H12.5	84.7 ± 5.4	-15.8 ± 1.8	SS	85.0 ± 5.0	-16.0 ± 1.6	SS	84.6 ± 4.8	-16.3 ± 1.8	SS	82.6 ± 4.9	-17.5 ± 1.8	SS	82.5 ± 5.2	-18.0 ± 1.9	SS	81.6 ± 5.7	-18.9 ± 2.0	SS
Al300<br>Aml5/B5	78.6 ± 5.2	-21.8 ± 1.5	SS	79.2 ± 4.9	-21.7 ± 1.6	SS	79.2 ± 4.5	-21.7 ± 1.7	SS	77.5 ± 4.8	-22.6 ± 1.9	SS	76.8 ± 5.0	-23.7 ± 2.2	SS	75.9 ± 5.7	-24.6 ± 2.2	SS
Al300<br>Aml5/H12.5	77.6 ± 5.4	-22.9 ± 2.1	SS	78.0 ± 5.0	-23.0 ± 1.9	SS	77.7 ± 4.9	-23.2 ± 2.2	SS	76.1 ± 4.9	-24.0 ± 2.1	SS	76.1 ± 5.2	-24.4 ± 2.2	SS	75.4 ± 5.7	-25.1 ± 2.1	SS
Al300<br>B5/H12.5	80.8 ± 5.4	-19.7 ± 2.0	SS	81.2 ± 5.0	-19.8 ± 2.0	SS	80.8 ± 4.8	-20.1 ± 2.3	SS	78.8 ± 5.0	-21.3 ± 2.4	SS	78.2 ± 5.0	-22.3 ± 2.6	SS	77.3 ± 5.7	-23.2 ± 2.7	SS
E20<br>Aml5/B5	81.2 ± 5.1	-19.2 ± 1.3	SS	81.0 ± 4.9	-20.0 ± 1.4	SS	80.4 ± 4.4	-20.6 ± 1.6	SS	75.6 ± 4.9	-24.5 ± 2.1	SS	74.3 ± 5.1	-26.2 ± 2.5	SS	73.1 ± 5.9	-27.3 ± 2.7	SS
E20<br>Aml5/H12.5	79.3 ± 5.3	-21.2 ± 2.0	SS	79.3 ± 5.0	-21.7 ± 1.8	SS	78.6 ± 4.9	-22.3 ± 2.1	SS	74.3 ± 5.0	-25.8 ± 2.4	SS	73.6 ± 5.2	-26.9 ± 2.4	SS	72.7 ± 5.8	-27.8 ± 2.5	SS
E20<br>B5/H12.5	82.7 ± 5.3	-17.7 ± 2.0	SS	82.5 ± 4.9	-18.4 ± 1.9	SS	81.7 ± 4.7	-19.2 ± 2.2	SS	77.1 ± 5.1	-23.0 ± 2.6	SS	76.0 ± 5.1	-24.5 ± 2.9	SS	74.8 ± 5.9	-25.7 ± 3.1	SS
L100<br>Aml5/B5	77.2 ± 5.3	-23.2 ± 1.6	SS	78.0 ± 5.0	-23.0 ± 1.7	SS	78.1 ± 4.5	-22.9 ± 1.8	SS	76.7 ± 4.8	-23.4 ± 2.0	SS	75.9 ± 5.0	-24.6 ± 2.3	SS	75.0 ± 5.7	-25.4 ± 2.4	SS
L100<br>Aml5/H12.5	76.8 ± 5.4	-23.6 ± 2.2	SS	77.2 ± 5.1	-23.7 ± 2.0	SS	76.9 ± 5.0	-24.0 ± 2.2	SS	75.2 ± 4.9	-24.9 ± 2.2	SS	75.1 ± 5.2	-25.3 ± 2.3	SS	74.5 ± 5.7	-26.0 ± 2.3	SS
L100<br>B5/H12.5	79.8 ± 5.4	-20.6 ± 2.0	SS	80.3 ± 5.0	-20.7 ± 2.0	SS	79.9 ± 4.8	-21.0 ± 2.4	SS	78.0 ± 5.0	-22.1 ± 2.5	SS	77.4 ± 5.0	-23.1 ± 2.7	SS	76.5 ± 5.7	-24.0 ± 2.8	SS

Al300 = aliskiren 300 mg; **Aml5** = amlodipine 5 mg; **B5** = bisoprolol 5 mg; **E20** = enalapril 20 mg; **H12.5** = hydrochlorothiazide 12.5 mg; **L100** = losartan 100 mg; **SS** = statistically significant (P < 0.00001)

Table S5. *P*-values calculated using the Kolmogorov-Smirnov test for changes in <u>diastolic blood pressure</u> in subpopulations (n = 100) with different ACE activity receiving the same regimens. Case 1: c_{ACE} = 7.0 h^{-1} (*LII*), case 2: c_{ACE} = 8.9 h^{-1} (*LID*), case 3: c_{ACE} = 10.8 h^{-1} (*LDD*), case 4: c_{ACE} = 42.3 h^{-1} (*HII*), case 5: c_{ACE} = 54.1 h^{-1} (*HID*), case 6: c_{ACE} = 65.9 h^{-1} (*HDD*). *P*-value for case *i* *vs*. case *j* is denoted P_{ij}.

Regimens	P_{12}	P_{13}	P_{23}	P_{14}	P_{15}	P_{16}	P_{24}	P_{25}	P_{26}	P_{34}	P_{35}	P_{36}	P_{45}	P_{46}	P_{56}
Al300	0.01581	0.00007	0.11113	SS	SS	SS	SS	SS	SS	SS	SS	SS	0.00045	SS	0.00025
E20	SS	SS	SS	SS	SS	SS	SS	SS	SS	SS	SS	SS	SS	SS	0.00002
L100	0.03663	0.00013	0.15454	SS	SS	SS	SS	SS	SS	SS	SS	SS	0.00025	SS	0.00079
Aml5	0.81275	0.36672	0.46756	SS	0.00007	SS	0.00013	0.00013	0.00002	0.00386	0.00630	0.00136	0.90621	0.81275	0.69937
B5	0.07832	0.00025	0.07832	SS	SS	SS	SS	SS	SS	SS	SS	SS	0.00013	SS	0.00630
H12.5	0.90621	0.90621	0.90621	0.00007	SS	SS	0.00025	SS	SS	0.00045	SS	SS	0.11113	0.11113	0.99376
Al300<br>Aml5	0.11113	0.02431	0.46756	SS	SS	SS	SS	SS	SS	SS	SS	SS	0.00232	SS	0.01581
Al300<br>B5	0.81275	0.99376	0.96707	0.00136	SS	SS	0.00079	SS	SS	0.00386	SS	SS	0.00386	SS	0.00630
Al300<br>H12.5	0.28093	0.05410	0.21055	SS	SS	SS	SS	SS	SS	0.00079	SS	SS	0.03663	0.00136	0.00630
E20<br>Aml5	SS	SS	0.00079	SS	SS	SS	SS	SS	SS	SS	SS	SS	SS	SS	0.00136
E20<br>B5	0.00136	SS	0.02431	SS	SS	SS	SS	SS	SS	SS	SS	SS	0.00025	SS	0.00025
E20<br>H12.5	0.00232	SS	0.00630	SS	SS	SS	SS	SS	SS	SS	SS	SS	0.00025	SS	0.00136
L100<br>Aml5	0.05410	0.02431	0.28093	SS	SS	SS	SS	SS	SS	SS	SS	SS	0.00136	SS	0.02431
L100<br>B5	0.69937	0.58062	0.69937	0.58062	0.00025	SS	0.11113	0.00025	SS	0.15454	SS	SS	0.00630	SS	0.00630
L100<br>H12.5	0.21055	0.05410	0.36672	SS	SS	SS	SS	SS	SS	0.00045	SS	SS	0.02431	0.00079	0.00630
Al300<br>Aml5/B5	0.90621	0.36672	0.46756	0.03663	SS	SS	0.02431	SS	SS	0.02431	SS	SS	0.00386	SS	0.03663
Al300<br>Aml5/H12.5	0.58062	0.15454	0.28093	0.01581	SS	SS	0.00232	SS	SS	0.15454	0.01581	0.00013	0.15454	0.01581	0.07832
Al300<br>B5/H12.5	0.28093	0.11113	0.58062	SS	SS	SS	0.00079	SS	SS	0.01008	SS	SS	0.07832	0.00025	0.02431
E20<br>Aml5/B5	0.00025	SS	0.05410	SS	SS	SS	SS	SS	SS	SS	SS	SS	0.00002	SS	0.02431
E20<br>Aml5/H12.5	0.02431	0.00045	0.01581	SS	SS	SS	SS	SS	SS	SS	SS	SS	0.00136	0.00004	0.03663
E20<br>B5/H12.5	0.01581	0.00002	0.11113	SS	SS	SS	SS	SS	SS	SS	SS	SS	0.00630	SS	0.01581
L100<br>Aml5/B5	0.58062	0.28093	0.69937	0.21055	0.00007	SS	0.15454	SS	SS	0.28093	SS	SS	0.00232	SS	0.07832
L100<br>Aml5/H12.5	0.58062	0.11113	0.28093	0.01008	SS	SS	0.00136	SS	SS	0.15454	0.00136	0.00002	0.05410	0.01581	0.07832
L100<br>B5/H12.5	0.46756	0.28093	0.46756	0.00007	SS	SS	0.00386	SS	SS	0.05410	SS	SS	0.05410	0.00045	0.02431

Al300 = aliskiren 300 mg; **Aml5** = amlodipine 5 mg; **B5** = bisoprolol 5 mg; **E20** = enalapril 20 mg; **H12.5** = hydrochlorothiazide 12.5 mg; **L100** = losartan 100 mg; **SS** = statistically significant (P < 0.00001)

Figure S10. Simulated change in heart rate from baseline to week 4 (mean ± SD, n = 100).

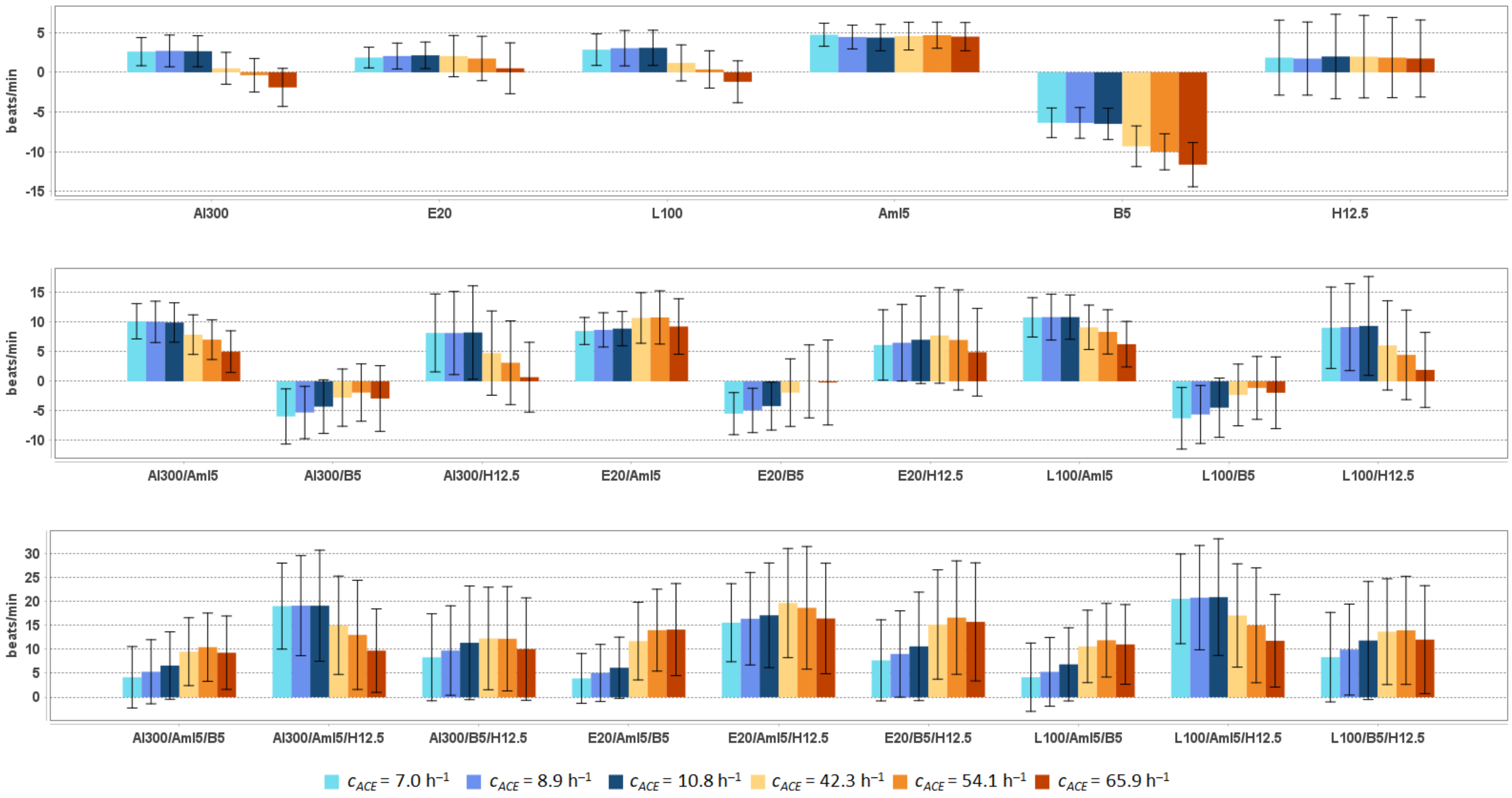

Al300 = aliskiren 300 mg; **Aml5** = amlodipine 5 mg; **B5** = bisoprolol 5 mg; **E20** = enalapril 20 mg; **H12.5** = hydrochlorothiazide 12.5 mg; **L100** = losartan 100 mg

Table S6. Simulated response of heart rate to antihypertensive therapy in virtual hypertensive subpopulations (n = 100) with different ACE activity, including P-values (Kolmogorov-Smirnov test) for endpoint *vs*. baseline; data are presented as mean ± SD in beats/min

**Regimens**	***LII*** **(c_{ACE} = 7.0 h^{-1})**			***LID*** **(c_{ACE} = 8.9 h^{-1})**			***LDD*** **(c_{ACE} = 10.8 h^{-1})**			***HII*** **(c_{ACE} = 42.3 h^{-1})**			***HID*** **(c_{ACE} = 54.1 h^{-1})**			***HDD*** **(c_{ACE} = 65.9 h^{-1})**		
	**Value**	**Change**	***P***	**Value**	**Change**	***P***	**Value**	**Change**	***P***	**Value**	**Change**	***P***	**Value**	**Change**	***P***	**Value**	**Change**	***P***
Baseline	77.2 ± 6.4	–	–	75.7 ± 7.6	–	–	75.2 ± 7.1	–	–	75.9 ± 7.1	–	–	76.6 ± 6.8	–	–	76.0 ± 7.4	–	–
Al300	79.8 ± 6.6	2.6 ± 1.8	0.05410	78.4 ± 8.1	2.7 ± 2.0	0.15454	77.9 ± 7.6	2.7 ± 2.0	0.15454	76.5 ± 7.7	0.5 ± 2.0	0.90621	76.2 ± 7.0	-0.4 ± 2.1	0.96707	74.1 ± 8.2	-1.9 ± 2.4	0.28093
E20	79.1 ± 6.5	1.9 ± 1.3	0.21055	77.7 ± 8.0	2.0 ± 1.6	0.28093	77.4 ± 7.5	2.1 ± 1.7	0.21055	78.0 ± 8.0	2.0 ± 2.6	0.36672	78.3 ± 7.2	1.7 ± 2.8	0.28093	76.5 ± 8.6	0.5 ± 3.2	0.69937
L100	80.1 ± 6.6	2.8 ± 2.0	0.02431	78.7 ± 8.2	3.0 ± 2.2	0.15454	78.3 ± 7.7	3.1 ± 2.2	0.11113	77.1 ± 7.8	1.2 ± 2.3	0.69937	77.0 ± 7.1	0.4 ± 2.3	0.90621	74.8 ± 8.3	-1.2 ± 2.6	0.46756
Aml5	82.0 ± 6.6	4.7 ± 1.5	0.00013	80.1 ± 7.8	4.4 ± 1.5	0.00136	79.6 ± 7.1	4.4 ± 1.7	0.00232	80.5 ± 7.6	4.6 ± 1.7	0.00630	81.3 ± 7.2	4.7 ± 1.6	0.00025	80.5 ± 7.8	4.5 ± 1.8	0.00136
B5	70.9 ± 6.5	-6.4 ± 1.9	SS	69.3 ± 7.9	-6.4 ± 1.9	0.00004	68.8 ± 7.6	-6.5 ± 2.0	SS	66.6 ± 7.4	-9.3 ± 2.6	SS	66.6 ± 6.9	-10.0 ± 2.3	SS	64.3 ± 8.1	-11.6 ± 2.8	SS
H12.5	79.1 ± 7.9	1.8 ± 4.7	0.21055	77.4 ± 8.8	1.7 ± 4.6	0.36672	77.2 ± 9.0	2.0 ± 5.3	0.21055	77.9 ± 8.8	2.0 ± 5.2	0.58062	78.4 ± 8.1	1.9 ± 5.0	0.28093	77.7 ± 9.4	1.7 ± 4.8	0.03663
Al300<br>Aml5	87.4 ± 6.9	10.1 ± 3.0	SS	85.7 ± 8.8	10.0 ± 3.5	SS	85.2 ± 8.0	9.9 ± 3.3	SS	83.8 ± 8.6	7.9 ± 3.3	SS	83.6 ± 7.6	7.0 ± 3.4	SS	81.0 ± 8.9	5.0 ± 3.5	0.00025
Al300<br>B5	71.3 ± 7.4	-5.9 ± 4.7	SS	70.4 ± 8.6	-5.3 ± 4.4	0.00386	71.0 ± 8.6	-4.3 ± 4.5	0.00136	73.2 ± 8.9	-2.8 ± 4.8	0.01581	74.7 ± 8.1	-1.9 ± 4.8	0.21055	73.1 ± 9.6	-2.9 ± 5.6	0.02431
Al300<br>H12.5	85.4 ± 9.0	8.2 ± 6.6	SS	83.8 ± 10.7	8.2 ± 7.0	SS	83.5 ± 11.0	8.2 ± 7.9	SS	80.7 ± 10.4	4.8 ± 7.1	0.05410	79.7 ± 9.3	3.1 ± 7.1	0.05410	76.7 ± 10.6	0.7 ± 5.9	0.21055
E20<br>Aml5	85.7 ± 6.7	8.5 ± 2.3	SS	84.4 ± 8.5	8.7 ± 2.9	SS	84.1 ± 7.8	8.9 ± 2.9	SS	86.7 ± 9.1	10.7 ± 4.3	SS	87.4 ± 8.0	10.8 ± 4.5	SS	85.2 ± 9.4	9.3 ± 4.7	SS
E20<br>B5	71.8 ± 6.9	-5.5 ± 3.6	0.00002	70.7 ± 8.4	-4.9 ± 3.7	0.00386	71.1 ± 8.4	-4.2 ± 4.1	0.00136	74.0 ± 9.4	-1.9 ± 5.7	0.15454	76.6 ± 8.9	-0.0 ± 6.2	0.46756	75.8 ± 10.5	-0.2 ± 7.2	0.21055
E20<br>H12.5	83.4 ± 8.6	6.1 ± 5.9	0.00002	82.2 ± 10.3	6.5 ± 6.5	0.00013	82.3 ± 10.7	7.0 ± 7.4	0.00007	83.7 ± 11.2	7.7 ± 8.1	0.00013	83.6 ± 10.2	7.0 ± 8.5	SS	80.9 ± 11.7	4.9 ± 7.4	0.00007
L100<br>Aml5	88.0 ± 7.0	10.8 ± 3.3	SS	86.5 ± 9.0	10.8 ± 3.9	SS	86.1 ± 8.2	10.8 ± 3.7	SS	85.1 ± 8.8	9.1 ± 3.7	SS	84.9 ± 7.7	8.3 ± 3.7	SS	82.2 ± 9.0	6.3 ± 3.9	0.00004
L100<br>B5	71.0 ± 7.8	-6.2 ± 5.2	SS	70.1 ± 8.8	-5.6 ± 4.9	0.00136	70.8 ± 8.9	-4.5 ± 5.0	0.00025	73.6 ± 9.1	-2.3 ± 5.2	0.03663	75.5 ± 8.3	-1.1 ± 5.3	0.28093	74.0 ± 9.9	-1.9 ± 6.1	0.07832
L100<br>H12.5	86.3 ± 9.2	9.0 ± 6.9	SS	84.8 ± 11.0	9.2 ± 7.4	SS	84.6 ± 11.4	9.4 ± 8.4	SS	82.0 ± 10.7	6.1 ± 7.5	0.00630	81.1 ± 9.6	4.5 ± 7.6	0.00630	77.9 ± 10.9	1.9 ± 6.3	0.01581
Al300<br>Aml5/B5	81.4 ± 8.6	4.1 ± 6.4	0.00045	81.0 ± 10.5	5.3 ± 6.7	0.00136	81.8 ± 10.3	6.6 ± 7.0	0.00007	85.4 ± 11.0	9.5 ± 7.1	SS	87.0 ± 9.5	10.4 ± 7.1	SS	85.2 ± 11.1	9.3 ± 7.7	SS
Al300<br>Aml5/H12.5	96.2 ± 10.8	19.0 ± 9.0	SS	94.8 ± 13.0	19.1 ± 10.4	SS	94.3 ± 13.7	19.1 ± 11.6	SS	90.9 ± 13.0	15.0 ± 10.3	SS	89.6 ± 12.6	13.0 ± 11.4	SS	85.7 ± 12.6	9.7 ± 8.7	SS
Al300<br>B5/H12.5	85.6 ± 10.8	8.3 ± 9.1	SS	85.4 ± 12.5	9.7 ± 9.3	SS	86.6 ± 14.3	11.3 ± 11.9	SS	88.2 ± 13.5	12.2 ± 10.7	SS	88.8 ± 11.9	12.2 ± 10.9	SS	86.0 ± 14.0	10.0 ± 10.7	SS
E20<br>Aml5/B5	81.1 ± 7.9	3.9 ± 5.2	0.00136	80.7 ± 10.1	5.0 ± 6.0	0.00386	81.4 ± 9.9	6.1 ± 6.4	0.00079	87.6 ± 11.7	11.7 ± 8.1	SS	90.6 ± 10.5	14.0 ± 8.6	SS	90.1 ± 12.2	14.1 ± 9.6	SS
E20<br>Aml5/H12.5	92.8 ± 10.2	15.5 ± 8.1	SS	92.0 ± 12.4	16.4 ± 9.7	SS	92.3 ± 13.1	17.1 ± 10.9	SS	95.6 ± 14.0	19.6 ± 11.4	SS	95.2 ± 13.6	18.6 ± 12.8	SS	92.4 ± 14.7	16.4 ± 11.5	SS
E20<br>B5/H12.5	84.9 ± 10.3	7.7 ± 8.5	SS	84.7 ± 12.3	9.0 ± 9.0	SS	85.8 ± 13.9	10.6 ± 11.3	SS	91.1 ± 14.1	15.2 ± 11.4	SS	93.2 ± 12.6	16.6 ± 11.8	SS	91.7 ± 15.1	15.7 ± 12.3	SS
L100<br>Aml5/B5	81.4 ± 9.2	4.2 ± 7.1	0.00025	81.0 ± 10.8	5.3 ± 7.2	0.00079	82.1 ± 10.7	6.8 ± 7.6	0.00007	86.5 ± 11.3	10.6 ± 7.6	SS	88.5 ± 9.9	11.9 ± 7.7	SS	87.0 ± 11.4	11.0 ± 8.3	SS
L100<br>Aml5/H12.5	97.8 ± 11.1	20.5 ± 9.4	SS	96.4 ± 13.3	20.8 ± 10.9	SS	96.1 ± 14.2	20.9 ± 12.2	SS	93.0 ± 13.4	17.0 ± 10.8	SS	91.6 ± 13.0	15.0 ± 12.0	SS	87.7 ± 13.3	11.8 ± 9.7	SS
L100<br>B5/H12.5	85.6 ± 11.0	8.3 ± 9.3	SS	85.6 ± 12.6	9.9 ± 9.5	SS	87.1 ± 14.6	11.8 ± 12.3	SS	89.6 ± 13.8	13.7 ± 11.0	SS	90.5 ± 12.2	13.9 ± 11.3	SS	88.0 ± 14.4	12.0 ± 11.3	SS

Al300 = aliskiren 300 mg; **Aml5** = amlodipine 5 mg; **B5** = bisoprolol 5 mg; **E20** = enalapril 20 mg; **H12.5** = hydrochlorothiazide 12.5 mg; **L100** = losartan 100 mg; **SS** = statistically significant (P < 0.00001)

Table S7. *P*-values calculated using the Kolmogorov-Smirnov test for changes in heart rate in subpopulations (n = 100) with different ACE activity receiving the same regimens. Case 1: c_{ACE} = 7.0 h^{-1} (*LII*), case 2: c_{ACE} = 8.9 h^{-1} (*LID*), case 3: c_{ACE} = 10.8 h^{-1} (*LDD*), case 4: c_{ACE} = 42.3 h^{-1} (*HII*), case 5: c_{ACE} = 54.1 h^{-1} (*HID*), case 6: c_{ACE} = 65.9 h^{-1} (*HDD*). *P*-value for case *i vs*. case *j* is denoted P_{ij}.

**Regimens**	P_{12}	P_{13}	P_{23}	P_{14}	P_{15}	P_{16}	P_{24}	P_{25}	P_{26}	P_{34}	P_{35}	P_{36}	P_{45}	P_{46}	P_{56}
Al300	0.36672	0.58062	0.36672	SS	SS	SS	SS	SS	SS	SS	SS	SS	0.02431	SS	0.00013
E20	0.15454	0.15454	0.46756	0.00630	0.00013	SS	0.11113	0.00630	SS	0.11113	0.01008	SS	0.46756	0.00386	0.02431
L100	0.36672	0.21055	0.69937	0.00004	SS	SS	0.00007	SS	SS	0.00013	SS	SS	0.05410	SS	0.00045
Aml5	0.69937	0.58062	0.99963	0.81275	0.90621	0.46756	0.69937	0.36672	0.46756	0.81275	0.36672	0.81275	0.69937	0.99376	0.46756
B5	0.96707	0.81275	0.81275	SS	SS	SS	SS	SS	SS	SS	SS	SS	0.02431	SS	0.00045
H12.5	0.58062	0.81275	0.58062	0.58062	0.90621	0.90621	0.11113	0.21055	0.36672	0.81275	0.58062	0.58062	0.90621	0.81275	0.99376
Al300<br>Aml5	0.69937	0.69937	0.81275	0.00007	SS	SS	0.00007	SS	SS	0.00232	SS	SS	0.11113	SS	0.00386
Al300<br>B5	0.69937	0.05410	0.21055	0.00025	SS	0.00013	0.00232	0.00013	0.00232	0.05410	0.03663	0.21055	0.69937	0.58062	0.28093
Al300<br>H12.5	0.81275	0.58062	0.36672	0.00079	SS	SS	0.00045	SS	SS	0.01008	0.00025	SS	0.11113	0.00025	0.15454
E20<br>Aml5	0.36672	0.05410	0.81275	SS	0.00007	0.00630	0.00045	0.00386	0.21055	0.00386	0.00386	0.28093	0.69937	0.07832	0.15454
E20<br>B5	0.81275	0.02431	0.28093	SS	SS	SS	0.00025	SS	SS	0.00136	0.00004	0.00025	0.11113	0.21055	0.36672
E20<br>H12.5	0.96707	0.28093	0.46756	0.07832	0.15454	0.02431	0.21055	0.28093	0.01008	0.96707	0.46756	0.07832	0.36672	0.05410	0.28093
L100<br>Aml5	0.69937	0.28093	0.90621	0.02431	SS	SS	0.01008	SS	SS	0.01008	0.00007	SS	0.15454	0.00013	0.00630
L100<br>B5	0.81275	0.07832	0.21055	0.00004	SS	SS	0.00025	SS	0.00013	0.01008	0.00630	0.03663	0.36672	0.69937	0.28093
L100<br>H12.5	0.96707	0.58062	0.58062	0.00386	SS	SS	0.00630	SS	SS	0.01581	0.00079	SS	0.15454	0.00136	0.11113
Al300<br>Aml5/B5	0.36672	0.03663	0.28093	SS	SS	SS	0.00079	0.00025	0.00045	0.03663	0.00386	0.05410	0.28093	0.58062	0.58062
Al300<br>Aml5/H12.5	0.69937	0.58062	0.81275	0.00386	SS	SS	0.01581	0.00002	SS	0.07832	0.00045	SS	0.21055	0.00079	0.07832
Al300<br>B5/H12.5	0.11113	0.01008	0.36672	0.01581	0.01008	0.15454	0.28093	0.36672	0.99376	0.28093	0.21055	0.46756	0.46756	0.36672	0.36672
E20<br>Aml5/B5	0.15454	0.01581	0.36672	SS	SS	SS	SS	SS	SS	SS	SS	SS	0.11113	0.28093	0.81275
E20<br>Aml5/H12.5	0.69937	0.15454	0.58062	0.00630	0.03663	0.81275	0.03663	0.05410	0.90621	0.36672	0.69937	0.81275	0.46756	0.07832	0.21055
E20<br>B5/H12.5	0.15454	0.00630	0.21055	SS	SS	SS	0.00045	0.00013	0.00004	0.00386	0.00025	0.01008	0.69937	0.58062	0.81275
L100<br>Aml5/B5	0.46756	0.05410	0.21055	SS	SS	SS	0.00013	SS	SS	0.00630	0.00013	0.00136	0.15454	0.69937	0.69937
L100<br>Aml5/H12.5	0.69937	0.46756	0.81275	0.01581	SS	SS	0.02431	0.00004	SS	0.15454	0.00079	SS	0.21055	0.00045	0.07832
L100<br>B5/H12.5	0.21055	0.00630	0.28093	0.00079	0.00232	0.00630	0.11113	0.07832	0.58062	0.21055	0.07832	0.81275	0.58062	0.69937	0.46756

Al300 = aliskiren 300 mg; **Aml5** = amlodipine 5 mg; **B5** = bisoprolol 5 mg; **E20** = enalapril 20 mg; **H12.5** = hydrochlorothiazide 12.5 mg; **L100** = losartan 100 mg; **SS** = statistically significant (P < 0.00001)

Figure S11. Simulated change in systemic arterial elasticity from baseline to week 4 (mean ± SD, n = 100)

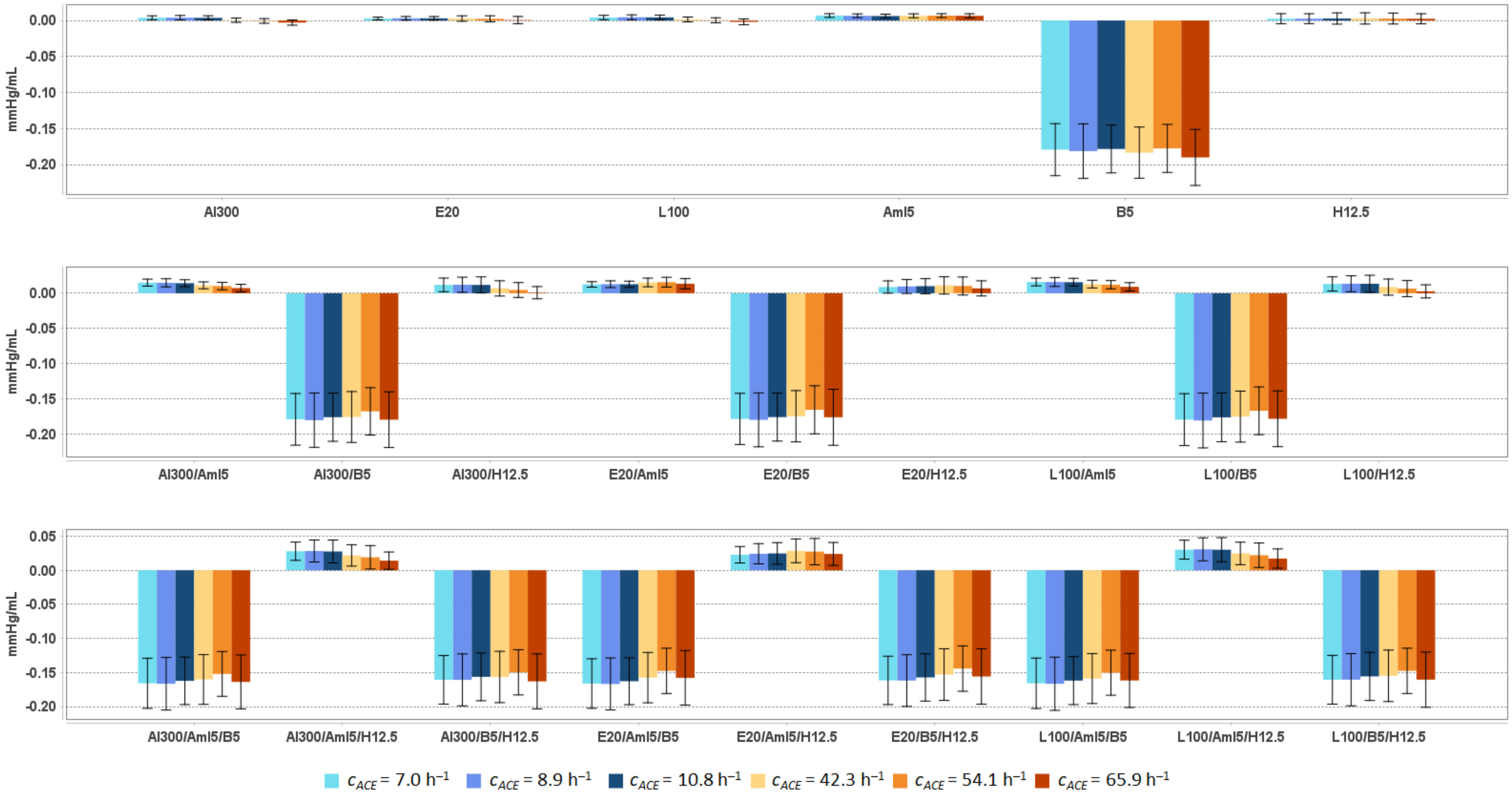

Al300 = aliskiren 300 mg; **Aml5** = amlodipine 5 mg; **B5** = bisoprolol 5 mg; **E20** = enalapril 20 mg; **H12.5** = hydrochlorothiazide 12.5 mg; **L100** = losartan 100 mg

Table S8. Simulated response of systemic arterial elasticity to antihypertensive therapy in virtual hypertensive subpopulations (n = 100) with different ACE activity, including P-values (Kolmogorov-Smirnov test) for endpoint *vs*. baseline; data are presented as mean ± SD in mmHg/mL

**Regimens**	***LII*** **(c_{ACE} = 7.0 h^{-1})**			***LID*** **(c_{ACE} = 8.9 h^{-1})**			***LDD*** **(c_{ACE} = 10.8 h^{-1})**			***HII*** **(c_{ACE} = 42.3 h^{-1})**			***HID*** **(c_{ACE} = 54.1 h^{-1})**			***HDD*** **(c_{ACE} = 65.9 h^{-1})**		
	**Value**	**Change**	***P***	**Value**	**Change**	***P***	**Value**	**Change**	***P***	**Value**	**Change**	***P***	**Value**	**Change**	***P***	**Value**	**Change**	***P***
Baseline	1.149 ± 0.240	–	–	1.163 ± 0.250	–	–	1.142 ± 0.220	–	–	1.153 ± 0.230	–	–	1.108 ± 0.220	–	–	1.177 ± 0.254	–	–
Al300	1.153 ± 0.240	0.004 ± 0.003	0.99963	1.167 ± 0.251	0.004 ± 0.003	1.00000	1.146 ± 0.220	0.004 ± 0.003	1.00000	1.154 ± 0.229	0.000 ± 0.003	1.00000	1.107 ± 0.221	-0.001 ± 0.003	1.00000	1.174 ± 0.254	-0.003 ± 0.004	1.00000
E20	1.152 ± 0.240	0.003 ± 0.002	1.00000	1.166 ± 0.251	0.003 ± 0.002	1.00000	1.145 ± 0.220	0.003 ± 0.002	1.00000	1.156 ± 0.229	0.003 ± 0.004	1.00000	1.110 ± 0.221	0.002 ± 0.004	1.00000	1.178 ± 0.254	0.001 ± 0.005	1.00000
L100	1.153 ± 0.240	0.004 ± 0.003	0.99963	1.167 ± 0.251	0.005 ± 0.003	1.00000	1.147 ± 0.220	0.004 ± 0.003	1.00000	1.155 ± 0.229	0.002 ± 0.003	1.00000	1.108 ± 0.220	0.000 ± 0.003	1.00000	1.175 ± 0.254	-0.002 ± 0.004	1.00000
Aml5	1.156 ± 0.240	0.007 ± 0.003	0.99376	1.169 ± 0.250	0.007 ± 0.003	1.00000	1.149 ± 0.220	0.006 ± 0.003	0.99963	1.160 ± 0.230	0.007 ± 0.003	1.00000	1.114 ± 0.221	0.007 ± 0.003	1.00000	1.184 ± 0.255	0.007 ± 0.003	0.99963
B5	0.970 ± 0.204	-0.179 ± 0.036	0.00002	0.981 ± 0.212	-0.181 ± 0.038	0.00002	0.964 ± 0.187	-0.178 ± 0.033	SS	0.970 ± 0.195	-0.183 ± 0.036	0.00004	0.930 ± 0.187	-0.178 ± 0.033	SS	0.987 ± 0.216	-0.190 ± 0.039	0.00002
H12.5	1.151 ± 0.241	0.003 ± 0.007	1.00000	1.165 ± 0.251	0.003 ± 0.007	1.00000	1.145 ± 0.221	0.003 ± 0.008	1.00000	1.156 ± 0.230	0.003 ± 0.008	1.00000	1.110 ± 0.221	0.003 ± 0.008	1.00000	1.180 ± 0.254	0.002 ± 0.007	1.00000
Al300 Aml5	1.164 ± 0.240	0.015 ± 0.005	0.90621	1.178 ± 0.251	0.015 ± 0.006	0.99376	1.157 ± 0.220	0.014 ± 0.005	0.96707	1.164 ± 0.229	0.011 ± 0.005	1.00000	1.118 ± 0.221	0.010 ± 0.005	0.99963	1.184 ± 0.254	0.007 ± 0.005	0.99963
Al300 B5	0.970 ± 0.204	-0.179 ± 0.037	0.00002	0.983 ± 0.212	-0.180 ± 0.038	0.00004	0.967 ± 0.186	-0.176 ± 0.034	SS	0.978 ± 0.195	-0.175 ± 0.036	0.00007	0.940 ± 0.187	-0.167 ± 0.034	0.00004	0.998 ± 0.216	-0.179 ± 0.039	0.00002
Al300 H12.5	1.161 ± 0.241	0.012 ± 0.010	0.90621	1.175 ± 0.252	0.012 ± 0.011	0.99963	1.154 ± 0.221	0.012 ± 0.011	0.96707	1.160 ± 0.229	0.007 ± 0.011	1.00000	1.112 ± 0.222	0.005 ± 0.011	0.99963	1.178 ± 0.254	0.001 ± 0.009	1.00000
E20 Aml5	1.161 ± 0.240	0.013 ± 0.004	0.96707	1.176 ± 0.251	0.013 ± 0.005	0.99376	1.155 ± 0.220	0.013 ± 0.004	0.96707	1.168 ± 0.229	0.015 ± 0.006	0.99376	1.123 ± 0.222	0.016 ± 0.007	0.99376	1.191 ± 0.254	0.013 ± 0.007	0.99376
E20 B5	0.971 ± 0.204	-0.178 ± 0.036	0.00002	0.983 ± 0.212	-0.179 ± 0.038	0.00004	0.967 ± 0.187	-0.176 ± 0.034	SS	0.979 ± 0.195	-0.174 ± 0.036	0.00007	0.942 ± 0.187	-0.165 ± 0.034	0.00004	1.001 ± 0.216	-0.176 ± 0.040	0.00004
E20 H12.5	1.158 ± 0.241	0.009 ± 0.009	0.99376	1.172 ± 0.251	0.010 ± 0.010	1.00000	1.152 ± 0.221	0.010 ± 0.011	0.99376	1.164 ± 0.229	0.011 ± 0.012	0.99963	1.118 ± 0.223	0.010 ± 0.013	0.99963	1.184 ± 0.254	0.007 ± 0.011	1.00000
L100 Aml5	1.165 ± 0.241	0.016 ± 0.005	0.90621	1.179 ± 0.251	0.016 ± 0.006	0.99376	1.158 ± 0.220	0.016 ± 0.006	0.96707	1.166 ± 0.229	0.013 ± 0.005	0.99376	1.120 ± 0.221	0.012 ± 0.006	0.99376	1.186 ± 0.254	0.009 ± 0.006	0.99963
L100 B5	0.970 ± 0.204	-0.179 ± 0.037	0.00002	0.982 ± 0.212	-0.180 ± 0.039	0.00004	0.966 ± 0.186	-0.176 ± 0.034	SS	0.978 ± 0.195	-0.175 ± 0.036	0.00007	0.941 ± 0.187	-0.167 ± 0.034	0.00004	0.999 ± 0.216	-0.178 ± 0.039	0.00004
L100 H12.5	1.162 ± 0.242	0.013 ± 0.010	0.90621	1.176 ± 0.252	0.013 ± 0.011	0.99963	1.156 ± 0.222	0.013 ± 0.012	0.96707	1.162 ± 0.229	0.009 ± 0.011	0.99963	1.114 ± 0.222	0.007 ± 0.011	0.99963	1.180 ± 0.254	0.003 ± 0.009	1.00000
Al300 Aml5/B5	0.983 ± 0.204	-0.166 ± 0.037	0.00004	0.996 ± 0.213	-0.167 ± 0.039	0.00025	0.980 ± 0.186	-0.162 ± 0.035	SS	0.993 ± 0.195	-0.160 ± 0.037	0.00079	0.956 ± 0.189	-0.152 ± 0.033	0.00025	1.013 ± 0.216	-0.164 ± 0.040	0.00004
Al300 Aml5/H12.5	1.177 ± 0.243	0.028 ± 0.013	0.58062	1.191 ± 0.253	0.028 ± 0.016	0.90621	1.170 ± 0.222	0.028 ± 0.017	0.58062	1.175 ± 0.230	0.022 ± 0.016	0.96707	1.127 ± 0.224	0.019 ± 0.017	0.90621	1.191 ± 0.255	0.014 ± 0.013	0.99376
Al300 B5/H12.5	0.988 ± 0.206	-0.161 ± 0.036	0.00007	1.002 ± 0.214	-0.161 ± 0.038	0.00025	0.986 ± 0.189	-0.156 ± 0.035	0.00013	0.997 ± 0.195	-0.157 ± 0.038	0.00079	0.958 ± 0.191	-0.150 ± 0.033	0.00045	1.014 ± 0.217	-0.163 ± 0.040	0.00004
E20 Aml5/B5	0.983 ± 0.204	-0.166 ± 0.036	0.00004	0.996 ± 0.213	-0.167 ± 0.038	0.00025	0.980 ± 0.187	-0.163 ± 0.035	SS	0.996 ± 0.195	-0.158 ± 0.037	0.00079	0.960 ± 0.189	-0.148 ± 0.033	0.00045	1.019 ± 0.217	-0.158 ± 0.040	0.00007
E20 Aml5/H12.5	1.172 ± 0.242	0.023 ± 0.012	0.69937	1.187 ± 0.253	0.024 ± 0.015	0.90621	1.167 ± 0.222	0.025 ± 0.016	0.58062	1.182 ± 0.231	0.029 ± 0.017	0.90621	1.135 ± 0.225	0.027 ± 0.019	0.81275	1.201 ± 0.256	0.024 ± 0.017	0.90621
E20 B5/H12.5	0.987 ± 0.206	-0.162 ± 0.035	0.00007	1.001 ± 0.215	-0.162 ± 0.038	0.00025	0.985 ± 0.189	-0.157 ± 0.035	0.00013	1.000 ± 0.196	-0.153 ± 0.038	0.00136	0.963 ± 0.191	-0.144 ± 0.033	0.00045	1.021 ± 0.217	-0.156 ± 0.041	0.00007
L100 Aml5/B5	0.983 ± 0.204	-0.166 ± 0.037	0.00007	0.996 ± 0.213	-0.167 ± 0.039	0.00013	0.980 ± 0.186	-0.162 ± 0.035	SS	0.994 ± 0.195	-0.159 ± 0.037	0.00079	0.957 ± 0.189	-0.150 ± 0.033	0.00045	1.016 ± 0.216	-0.162 ± 0.040	0.00007
L100 Aml5/H12.5	1.179 ± 0.243	0.030 ± 0.014	0.58062	1.194 ± 0.254	0.031 ± 0.017	0.90621	1.173 ± 0.223	0.030 ± 0.018	0.58062	1.178 ± 0.230	0.025 ± 0.017	0.96707	1.130 ± 0.224	0.022 ± 0.018	0.90621	1.194 ± 0.256	0.017 ± 0.014	0.99376
L100 B5/H12.5	0.988 ± 0.206	-0.161 ± 0.036	0.00007	1.002 ± 0.214	-0.161 ± 0.038	0.00025	0.987 ± 0.189	-0.156 ± 0.035	0.00013	0.998 ± 0.196	-0.155 ± 0.038	0.00136	0.960 ± 0.191	-0.148 ± 0.033	0.00045	1.017 ± 0.217	-0.161 ± 0.040	0.00007

Al300 = aliskiren 300 mg; **Aml5** = amlodipine 5 mg; **B5** = bisoprolol 5 mg; **E20** = enalapril 20 mg; **H12.5** = hydrochlorothiazide 12.5 mg; **L100** = losartan 100 mg; **SS** = statistically significant (P < 0.00001)

Table S9. *P*-values calculated using the Kolmogorov-Smirnov test for changes in systemic arterial elasticity in subpopulations (n = 100) with different ACE activity receiving the same regimens. Case 1: c_{ACE} = 7.0 h^{-1} (*LII*), case 2: c_{ACE} = 8.9 h^{-1} (*LID*), case 3: c_{ACE} = 10.8 h^{-1} (*LDD*), case 4: c_{ACE} = 42.3 h^{-1} (*HII*), case 5: c_{ACE} = 54.1 h^{-1} (*HID*), case 6: c_{ACE} = 65.9 h^{-1} (*HDD*). *P*-value for case *i vs*. case *j* is denoted P_{ij}.

**Regimens**	P_{12}	P_{13}	P_{23}	P_{14}	P_{15}	P_{16}	P_{24}	P_{25}	P_{26}	P_{34}	P_{35}	P_{36}	P_{45}	P_{46}	P_{56}
Al300	0.46756	0.58062	0.58062	SS	SS	SS	SS	SS	SS	SS	SS	SS	0.01581	SS	0.00013
E20	0.21055	0.15454	0.58062	0.00386	0.00025	SS	0.05410	0.00386	SS	0.05410	0.00386	SS	0.46756	0.00630	0.05410
L100	0.21055	0.46756	0.58062	0.00013	SS	SS	0.00004	SS	SS	SS	SS	SS	0.03663	SS	0.00079
Aml5	0.81275	0.28093	0.46756	0.81275	0.90621	0.58062	0.96707	0.90621	0.99376	0.36672	0.46756	0.46756	0.81275	0.99963	0.69937
B5	0.69937	0.69937	0.81275	0.69937	0.69937	0.07832	0.69937	0.36672	0.36672	0.36672	0.81275	0.07832	0.36672	0.46756	0.01581
H12.5	0.69937	0.96707	0.46756	0.69937	0.90621	0.69937	0.28093	0.21055	0.28093	0.81275	0.58062	0.46756	0.81275	0.90621	0.99376
Al300 Aml5	0.81275	0.58062	0.58062	0.00002	SS	SS	0.00025	SS	SS	0.00136	SS	SS	0.11113	0.00002	0.00386
Al300 B5	0.90621	0.69937	0.69937	0.90621	0.28093	0.96707	0.58062	0.05410	0.99963	0.90621	0.15454	0.69937	0.28093	0.81275	0.03663
Al300 H12.5	0.46756	0.46756	0.28093	0.00079	SS	SS	0.00025	SS	SS	0.01581	0.00025	SS	0.15454	0.00079	0.15454
E20 Aml5	0.46756	0.58062	0.96707	0.00045	0.00079	0.07832	0.01581	0.01008	0.36672	0.02431	0.00136	0.15454	0.58062	0.15454	0.07832
E20 B5	0.90621	0.69937	0.69937	0.69937	0.15454	0.58062	0.69937	0.02431	0.90621	0.96707	0.07832	0.96707	0.15454	0.90621	0.05410
E20 H12.5	0.96707	0.46756	0.58062	0.11113	0.15454	0.02431	0.28093	0.21055	0.01581	0.90621	0.46756	0.07832	0.36672	0.11113	0.15454
L100 Aml5	0.81275	0.90621	0.81275	0.00386	0.00002	SS	0.00630	0.00004	SS	0.01008	0.00007	SS	0.11113	0.00045	0.01581
L100 B5	0.90621	0.69937	0.69937	0.81275	0.21055	0.90621	0.58062	0.01581	0.96707	0.90621	0.07832	0.81275	0.21055	0.81275	0.05410
L100 H12.5	0.58062	0.46756	0.69937	0.00386	SS	SS	0.00630	SS	SS	0.01581	0.00079	SS	0.21055	0.00386	0.21055
Al300 Aml5/B5	0.90621	0.81275	0.81275	0.58062	0.11113	0.90621	0.46756	0.01008	0.90621	0.96707	0.07832	0.90621	0.21055	0.69937	0.02431
Al300 Aml5/H12.5	0.69937	0.46756	0.69937	0.00136	SS	SS	0.00386	0.00002	SS	0.07832	0.00079	SS	0.21055	0.00386	0.05410
Al300 B5/H12.5	0.99376	0.90621	0.69937	0.46756	0.11113	0.58062	0.46756	0.05410	0.90621	0.90621	0.21055	0.36672	0.36672	0.36672	0.02431
E20 Aml5/B5	0.90621	0.90621	0.69937	0.28093	0.01581	0.28093	0.21055	0.00136	0.46756	0.69937	0.00386	0.81275	0.11113	0.99376	0.03663
E20 Aml5/H12.5	0.46756	0.36672	0.58062	0.01581	0.03663	0.36672	0.11113	0.07832	0.90621	0.46756	0.69937	0.69937	0.58062	0.07832	0.11113
E20 B5/H12.5	0.96707	0.96707	0.69937	0.15454	0.00630	0.36672	0.11113	0.01008	0.58062	0.58062	0.03663	0.81275	0.36672	0.69937	0.02431
L100 Aml5/B5	0.90621	0.90621	0.81275	0.58062	0.03663	0.58062	0.46756	0.00630	0.90621	0.90621	0.03663	0.96707	0.21055	0.81275	0.02431
L100 Aml5/H12.5	0.69937	0.58062	0.69937	0.00630	0.00004	SS	0.03663	0.00013	SS	0.15454	0.00232	SS	0.28093	0.00386	0.05410
L100 B5/H12.5	0.99376	0.81275	0.69937	0.21055	0.07832	0.81275	0.46756	0.03663	0.96707	0.90621	0.21055	0.81275	0.36672	0.46756	0.03663

Al300 = aliskiren 300 mg; **Aml5** = amlodipine 5 mg; **B5** = bisoprolol 5 mg; **E20** = enalapril 20 mg; **H12.5** = hydrochlorothiazide 12.5 mg; **L100** = losartan 100 mg; **SS** = statistically significant (P < 0.00001)

Figure S12. Simulated change in systemic vascular resistance from baseline to week 4 (mean ± SD, n = 100)

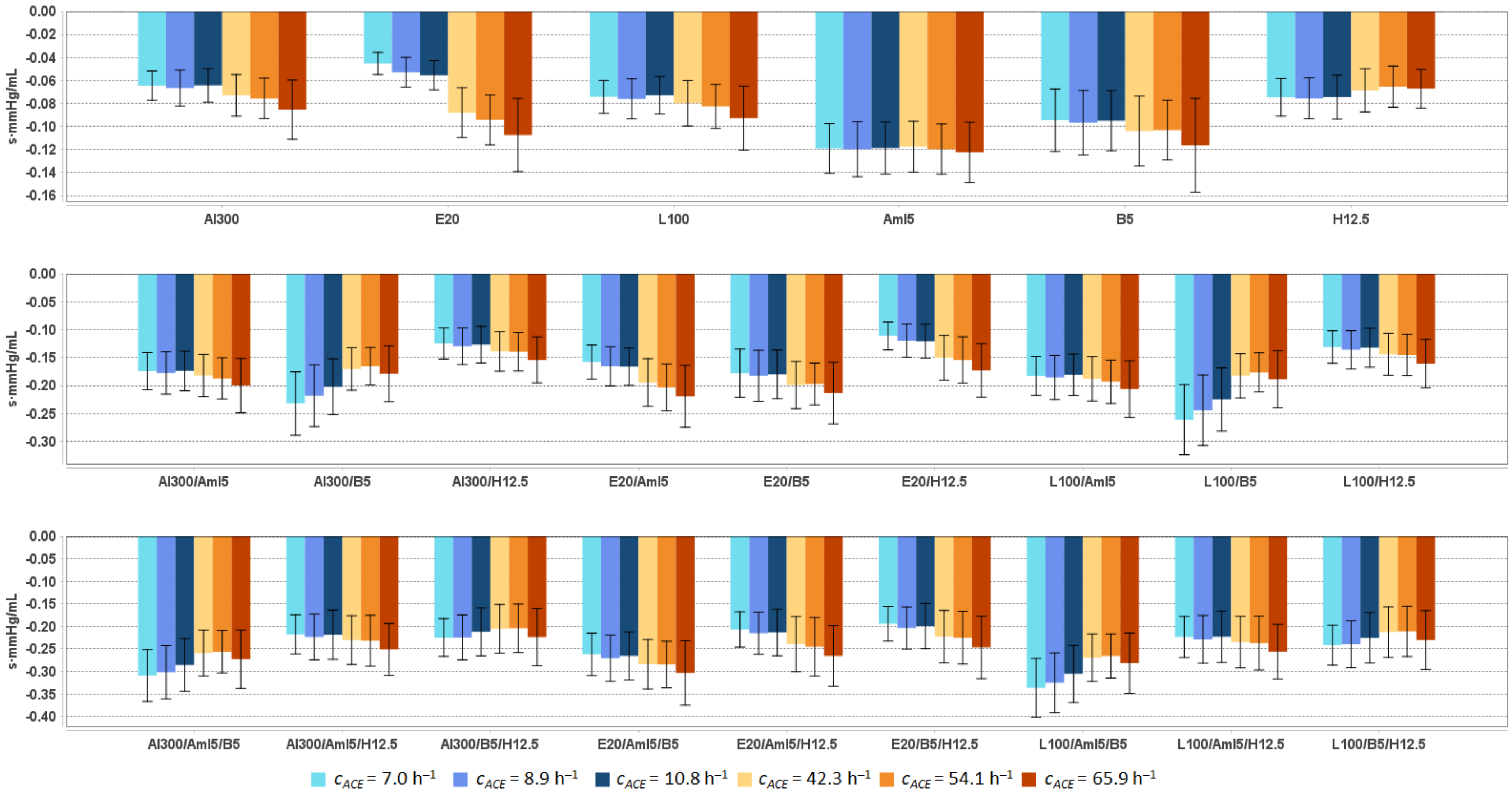

Al300 = aliskiren 300 mg; **Aml5** = amlodipine 5 mg; **B5** = bisoprolol 5 mg; **E20** = enalapril 20 mg; **H12.5** = hydrochlorothiazide 12.5 mg; **L100** = losartan 100 mg

Table S10. Simulated response of systemic vascular resistance to antihypertensive therapy in virtual hypertensive subpopulations (n = 100) with different ACE activity, including P-values (Kolmogorov-Smirnov test) for endpoint *vs*. baseline; data are presented as mean ± SD in s·mmHg/mL

**Regimens**	***LII*** **(c_{ACE} = 7.0 h^{-1})**			***LID*** **(c_{ACE} = 8.9 h^{-1})**			***LDD*** **(c_{ACE} = 10.8 h^{-1})**			***HII*** **(c_{ACE} = 42.3 h^{-1})**			***HID*** **(c_{ACE} = 54.1 h^{-1})**			***HDD*** **(c_{ACE} = 65.9 h^{-1})**		
	**Value**	**Change**	***P***	**Value**	**Change**	***P***	**Value**	**Change**	***P***	**Value**	**Change**	***P***	**Value**	**Change**	***P***	**Value**	**Change**	***P***
Baseline	1.39 ± 0.20	–	–	1.44 ± 0.22	–	–	1.42 ± 0.23	–	–	1.43 ± 0.22	–	–	1.41 ± 0.20	–	–	1.45 ± 0.25	–	–
Al300	1.33 ± 0.19	-0.06 ± 0.01	0.07832	1.37 ± 0.20	-0.07 ± 0.02	0.03663	1.36 ± 0.22	-0.06 ± 0.01	0.28093	1.36 ± 0.21	-0.07 ± 0.02	0.05410	1.33 ± 0.19	-0.08 ± 0.02	0.02431	1.37 ± 0.22	-0.09 ± 0.03	0.02431
E20	1.35 ± 0.19	-0.05 ± 0.01	0.36672	1.38 ± 0.21	-0.05 ± 0.01	0.11113	1.36 ± 0.22	-0.06 ± 0.01	0.28093	1.34 ± 0.20	-0.09 ± 0.02	0.02431	1.31 ± 0.18	-0.09 ± 0.02	0.01581	1.35 ± 0.22	-0.11 ± 0.03	0.00386
L100	1.32 ± 0.19	-0.07 ± 0.01	0.02431	1.36 ± 0.20	-0.08 ± 0.02	0.01581	1.35 ± 0.22	-0.07 ± 0.02	0.15454	1.35 ± 0.21	-0.08 ± 0.02	0.03663	1.32 ± 0.19	-0.08 ± 0.02	0.01581	1.36 ± 0.22	-0.09 ± 0.03	0.01008
Aml5	1.27 ± 0.18	-0.12 ± 0.02	0.00025	1.32 ± 0.20	-0.12 ± 0.02	0.00136	1.30 ± 0.21	-0.12 ± 0.02	0.00232	1.31 ± 0.21	-0.12 ± 0.02	0.00079	1.29 ± 0.18	-0.12 ± 0.02	0.00136	1.33 ± 0.22	-0.12 ± 0.03	0.00079
B5	1.30 ± 0.18	-0.09 ± 0.03	0.00630	1.34 ± 0.19	-0.10 ± 0.03	0.00630	1.33 ± 0.21	-0.09 ± 0.03	0.02431	1.33 ± 0.20	-0.10 ± 0.03	0.00232	1.30 ± 0.18	-0.10 ± 0.03	0.01008	1.34 ± 0.21	-0.12 ± 0.04	0.00136
H12.5	1.32 ± 0.19	-0.07 ± 0.02	0.02431	1.36 ± 0.20	-0.08 ± 0.02	0.02431	1.35 ± 0.21	-0.07 ± 0.02	0.11113	1.36 ± 0.21	-0.07 ± 0.02	0.11113	1.34 ± 0.19	-0.07 ± 0.02	0.05410	1.39 ± 0.23	-0.07 ± 0.02	0.15454
Al300<br>Aml5	1.22 ± 0.17	-0.17 ± 0.03	SS	1.26 ± 0.19	-0.18 ± 0.04	SS	1.25 ± 0.20	-0.17 ± 0.04	0.00002	1.25 ± 0.19	-0.18 ± 0.04	SS	1.22 ± 0.17	-0.19 ± 0.04	SS	1.25 ± 0.20	-0.20 ± 0.05	SS
Al300<br>B5	1.16 ± 0.16	-0.23 ± 0.06	SS	1.22 ± 0.18	-0.22 ± 0.06	SS	1.22 ± 0.19	-0.20 ± 0.05	SS	1.26 ± 0.19	-0.17 ± 0.04	SS	1.24 ± 0.17	-0.17 ± 0.03	SS	1.28 ± 0.20	-0.18 ± 0.05	SS
Al300<br>H12.5	1.27 ± 0.18	-0.12 ± 0.03	0.00004	1.31 ± 0.19	-0.13 ± 0.03	0.00025	1.29 ± 0.21	-0.13 ± 0.03	0.00232	1.29 ± 0.19	-0.14 ± 0.04	0.00002	1.27 ± 0.18	-0.14 ± 0.03	0.00025	1.30 ± 0.21	-0.15 ± 0.04	0.00004
E20<br>Aml5	1.24 ± 0.18	-0.16 ± 0.03	SS	1.27 ± 0.19	-0.17 ± 0.04	0.00002	1.25 ± 0.20	-0.17 ± 0.03	0.00004	1.24 ± 0.19	-0.19 ± 0.04	SS	1.20 ± 0.17	-0.20 ± 0.04	SS	1.24 ± 0.20	-0.22 ± 0.06	SS
E20<br>B5	1.22 ± 0.17	-0.18 ± 0.04	SS	1.25 ± 0.18	-0.18 ± 0.05	SS	1.24 ± 0.19	-0.18 ± 0.04	SS	1.23 ± 0.18	-0.20 ± 0.04	SS	1.21 ± 0.17	-0.20 ± 0.04	SS	1.24 ± 0.19	-0.21 ± 0.06	SS
E20<br>H12.5	1.28 ± 0.18	-0.11 ± 0.02	0.00136	1.32 ± 0.19	-0.12 ± 0.03	0.00079	1.30 ± 0.21	-0.12 ± 0.03	0.00386	1.28 ± 0.19	-0.15 ± 0.04	SS	1.25 ± 0.18	-0.15 ± 0.04	0.00002	1.28 ± 0.20	-0.17 ± 0.05	0.00002
L100<br>Aml5	1.21 ± 0.17	-0.18 ± 0.03	SS	1.25 ± 0.18	-0.19 ± 0.04	SS	1.24 ± 0.20	-0.18 ± 0.04	0.00002	1.24 ± 0.19	-0.19 ± 0.04	SS	1.21 ± 0.17	-0.19 ± 0.04	SS	1.25 ± 0.20	-0.21 ± 0.05	SS
L100<br>B5	1.13 ± 0.16	-0.26 ± 0.06	SS	1.19 ± 0.18	-0.24 ± 0.06	SS	1.20 ± 0.19	-0.22 ± 0.06	SS	1.25 ± 0.19	-0.18 ± 0.04	SS	1.23 ± 0.17	-0.18 ± 0.03	SS	1.27 ± 0.20	-0.19 ± 0.05	SS
L100<br>H12.5	1.26 ± 0.18	-0.13 ± 0.03	0.00002	1.30 ± 0.19	-0.14 ± 0.03	0.00025	1.29 ± 0.21	-0.13 ± 0.03	0.00136	1.29 ± 0.19	-0.14 ± 0.04	SS	1.26 ± 0.18	-0.15 ± 0.04	0.00013	1.29 ± 0.21	-0.16 ± 0.04	0.00004
Al300<br>Aml5/B5	1.08 ± 0.15	-0.31 ± 0.06	SS	1.13 ± 0.17	-0.30 ± 0.06	SS	1.13 ± 0.18	-0.29 ± 0.06	SS	1.17 ± 0.18	-0.26 ± 0.05	SS	1.15 ± 0.16	-0.26 ± 0.05	SS	1.18 ± 0.19	-0.27 ± 0.06	SS
Al300<br>Aml5/H12.5	1.18 ± 0.17	-0.22 ± 0.04	SS	1.21 ± 0.18	-0.22 ± 0.05	SS	1.20 ± 0.20	-0.22 ± 0.05	SS	1.20 ± 0.19	-0.23 ± 0.05	SS	1.17 ± 0.17	-0.23 ± 0.06	SS	1.20 ± 0.20	-0.25 ± 0.06	SS
Al300<br>B5/H12.5	1.17 ± 0.16	-0.22 ± 0.04	SS	1.21 ± 0.18	-0.22 ± 0.05	SS	1.21 ± 0.19	-0.21 ± 0.05	SS	1.23 ± 0.18	-0.21 ± 0.05	SS	1.20 ± 0.17	-0.20 ± 0.05	SS	1.23 ± 0.19	-0.22 ± 0.06	SS
E20<br>Aml5/B5	1.13 ± 0.16	-0.26 ± 0.05	SS	1.16 ± 0.17	-0.27 ± 0.05	SS	1.15 ± 0.18	-0.27 ± 0.05	SS	1.15 ± 0.17	-0.28 ± 0.05	SS	1.12 ± 0.16	-0.28 ± 0.05	SS	1.15 ± 0.18	-0.30 ± 0.07	SS
E20<br>Aml5/H12.5	1.19 ± 0.17	-0.21 ± 0.04	SS	1.22 ± 0.18	-0.22 ± 0.05	SS	1.21 ± 0.20	-0.21 ± 0.05	SS	1.19 ± 0.19	-0.24 ± 0.06	SS	1.16 ± 0.17	-0.25 ± 0.06	SS	1.19 ± 0.20	-0.27 ± 0.07	SS
E20<br>B5/H12.5	1.20 ± 0.17	-0.19 ± 0.04	SS	1.23 ± 0.18	-0.20 ± 0.05	SS	1.22 ± 0.19	-0.20 ± 0.05	SS	1.21 ± 0.18	-0.22 ± 0.06	SS	1.18 ± 0.17	-0.22 ± 0.06	SS	1.21 ± 0.19	-0.25 ± 0.07	SS
L100<br>Aml5/B5	1.06 ± 0.15	-0.34 ± 0.07	SS	1.11 ± 0.17	-0.33 ± 0.07	SS	1.11 ± 0.18	-0.31 ± 0.06	SS	1.16 ± 0.18	-0.27 ± 0.05	SS	1.14 ± 0.16	-0.27 ± 0.05	SS	1.17 ± 0.19	-0.28 ± 0.07	SS
L100<br>Aml5/H12.5	1.17 ± 0.17	-0.22 ± 0.05	SS	1.21 ± 0.18	-0.23 ± 0.05	SS	1.20 ± 0.20	-0.22 ± 0.06	SS	1.20 ± 0.19	-0.23 ± 0.06	SS	1.17 ± 0.17	-0.24 ± 0.06	SS	1.20 ± 0.20	-0.26 ± 0.06	SS
L100<br>B5/H12.5	1.15 ± 0.16	-0.24 ± 0.04	SS	1.20 ± 0.18	-0.24 ± 0.05	SS	1.20 ± 0.19	-0.23 ± 0.06	SS	1.22 ± 0.18	-0.21 ± 0.06	SS	1.19 ± 0.17	-0.21 ± 0.06	SS	1.22 ± 0.19	-0.23 ± 0.07	SS

Al300 = aliskiren 300 mg; **Aml5** = amlodipine 5 mg; **B5** = bisoprolol 5 mg; **E20** = enalapril 20 mg; **H12.5** = hydrochlorothiazide 12.5 mg; **L100** = losartan 100 mg; **SS** = statistically significant (P < 0.00001)

Table S11. *P*-values calculated using the Kolmogorov-Smirnov test for changes in <u>systemic vascular resistance</u> in subpopulations (n = 100) with different ACE activity receiving the same regimens. Case 1: c_{ACE} = 7.0 h^{-1} (*LII*), case 2: c_{ACE} = 8.9 h^{-1} (*LID*), case 3: c_{ACE} = 10.8 h^{-1} (*LDD*), case 4: c_{ACE} = 42.3 h^{-1} (*HII*), case 5: c_{ACE} = 54.1 h^{-1} (*HID*), case 6: c_{ACE} = 65.9 h^{-1} (*HDD*). *P*-value for case *i vs*. case *j* is denoted P_{ij}.

Regimens	P_{12}	P_{13}	P_{23}	P_{14}	P_{15}	P_{16}	P_{24}	P_{25}	P_{26}	P_{34}	P_{35}	P_{36}	P_{45}	P_{46}	P_{56}
Al300	0.21055	0.69937	0.46756	0.00079	0.00004	SS	0.03663	0.01581	SS	0.00630	0.00079	SS	0.69937	0.00136	0.01581
E20	0.00013	SS	0.36672	SS	SS	SS	SS	SS	SS	SS	SS	SS	0.15454	0.00007	0.01581
L100	0.46756	0.36672	0.58062	0.01581	0.00630	SS	0.28093	0.15454	0.00007	0.01581	0.00630	SS	0.58062	0.00232	0.01008
Aml5	0.81275	0.90621	0.99376	0.99376	0.69937	0.11113	0.90621	0.90621	0.58062	0.58062	0.90621	0.58062	0.36672	0.07832	0.21055
B5	0.58062	0.46756	0.96707	0.02431	0.01008	SS	0.07832	0.21055	0.00079	0.03663	0.07832	0.00079	0.96707	0.02431	0.07832
H12.5	0.69937	0.46756	0.90621	0.01008	0.00079	0.01581	0.01581	0.00630	0.03663	0.02431	0.00232	0.11113	0.28093	0.81275	0.36672
Al300<br>Aml5	0.90621	0.46756	0.81275	0.15454	0.01008	0.00025	0.46756	0.11113	0.00232	0.28093	0.11113	0.00079	0.28093	0.01581	0.05410
Al300<br>B5	0.07832	0.00079	0.15454	SS	SS	SS	SS	SS	0.00045	0.00013	0.00007	0.01581	0.58062	0.07832	0.05410
Al300<br>H12.5	0.21055	0.15454	0.46756	0.00386	0.01581	SS	0.07832	0.28093	0.00136	0.07832	0.05410	0.00002	0.90621	0.00630	0.03663
E20<br>Aml5	0.21055	0.05410	0.90621	SS	SS	SS	SS	SS	SS	0.00004	SS	SS	0.21055	0.00630	0.01581
E20<br>B5	0.15454	0.36672	0.99376	0.00002	0.00007	SS	0.01581	0.02431	0.00232	0.00386	0.00386	0.00013	0.90621	0.05410	0.03663
E20<br>H12.5	0.07832	0.00630	0.81275	SS	SS	SS	SS	SS	SS	SS	SS	SS	0.58062	0.00232	0.02431
L100<br>Aml5	0.90621	0.58062	0.81275	0.36672	0.03663	0.00045	0.81275	0.21055	0.00630	0.36672	0.15454	0.00079	0.36672	0.01581	0.03663
L100<br>B5	0.05410	0.00025	0.21055	SS	SS	SS	SS	SS	SS	SS	SS	0.00136	0.46756	0.21055	0.07832
L100<br>H12.5	0.21055	0.28093	0.46756	0.01581	0.02431	SS	0.15454	0.36672	0.00232	0.11113	0.05410	0.00004	0.90621	0.01008	0.02431
Al300<br>Aml5/B5	0.21055	0.00630	0.15454	SS	SS	SS	SS	SS	0.00386	0.00386	0.00630	0.07832	0.81275	0.03663	0.05410
Al300<br>Aml5/H12.5	0.28093	0.28093	0.69937	0.15454	0.07832	0.00013	0.28093	0.36672	0.03663	0.21055	0.36672	0.00232	0.69937	0.01581	0.11113
Al300<br>B5/H12.5	0.28093	0.11113	0.21055	0.01008	0.00386	0.02431	0.02431	0.02431	0.58062	0.28093	0.11113	0.21055	0.81275	0.01581	0.05410
E20<br>Aml5/B5	0.07832	0.58062	0.46756	0.00232	0.00386	0.00025	0.11113	0.36672	0.00232	0.07832	0.11113	0.00232	0.81275	0.02431	0.03663
E20<br>Aml5/H12.5	0.07832	0.03663	0.96707	SS	SS	SS	0.00386	0.00136	0.00002	0.01581	0.00232	SS	0.81275	0.00630	0.11113
E20<br>B5/H12.5	0.11113	0.28093	0.69937	0.00045	0.00025	SS	0.01581	0.05410	0.00013	0.02431	0.01581	0.00007	0.81275	0.02431	0.03663
L100<br>Aml5/B5	0.15454	0.00386	0.15454	SS	SS	SS	SS	SS	0.00045	0.00079	0.00079	0.01581	0.81275	0.05410	0.07832
L100<br>Aml5/H12.5	0.36672	0.28093	0.69937	0.15454	0.11113	0.00079	0.46756	0.58062	0.05410	0.36672	0.36672	0.00232	0.81275	0.01008	0.11113
L100<br>B5/H12.5	0.36672	0.07832	0.15454	0.00045	0.00025	0.00232	0.00386	0.00630	0.21055	0.07832	0.03663	0.36672	0.90621	0.03663	0.05410

Al300 = aliskiren 300 mg; **Aml5** = amlodipine 5 mg; **B5** = bisoprolol 5 mg; **E20** = enalapril 20 mg; **H12.5** = hydrochlorothiazide 12.5 mg; **L100** = losartan 100 mg; **SS** = statistically significant (P < 0.00001)

Figure S13. Simulated change in diastolic pulmonary arterial pressure from baseline to week 4 (mean ± SD, n = 100)

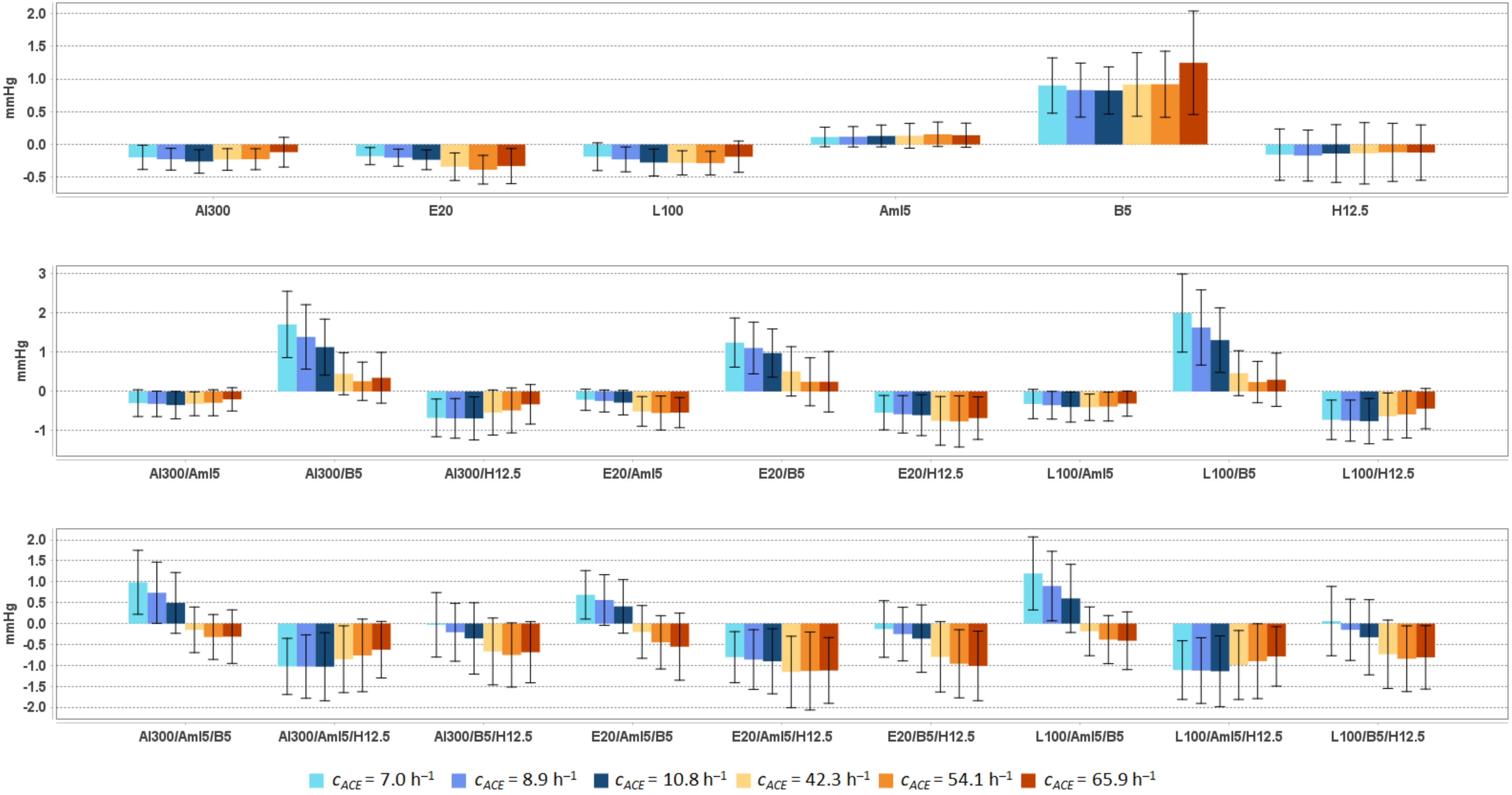

Al300 = aliskiren 300 mg; **Aml5** = amlodipine 5 mg; **B5** = bisoprolol 5 mg; **E20** = enalapril 20 mg; **H12.5** = hydrochlorothiazide 12.5 mg; **L100** = losartan 100 mg

Table S12. Simulated response of diastolic pulmonary arterial pressure to antihypertensive therapy in virtual hypertensive subpopulations (n = 100) with different ACE activity, including P-values (Kolmogorov-Smirnov test) for endpoint *vs*. baseline; data are presented as mean ± SD in mmHg

**Regimens**	***LII*** (**c_{ACE} = 7.0 h^{-1}**)			***LID*** (**c_{ACE} = 8.9 h^{-1}**)			***LDD*** (**c_{ACE} = 10.8 h^{-1}**)			***HII*** (**c_{ACE} = 42.3 h^{-1}**)			***HID*** (**c_{ACE} = 54.1 h^{-1}**)			***HDD*** (**c_{ACE} = 65.9 h^{-1}**)		
	**Value**	**Change**	***P***	**Value**	**Change**	***P***	**Value**	**Change**	***P***	**Value**	**Change**	***P***	**Value**	**Change**	***P***	**Value**	**Change**	***P***
Baseline	11.3 ± 0.6	–	–	11.3 ± 0.5	–	–	11.2 ± 0.7	–	–	11.2 ± 0.6	–	–	11.1 ± 0.7	–	–	11.3 ± 0.6	–	–
Al300	11.1 ± 0.6	-0.2 ± 0.2	0.01008	11.0 ± 0.5	-0.2 ± 0.2	0.00386	10.9 ± 0.7	-0.3 ± 0.2	0.00232	11.0 ± 0.6	-0.2 ± 0.2	0.00232	10.9 ± 0.7	-0.2 ± 0.2	0.01008	11.1 ± 0.7	-0.1 ± 0.2	0.15454
E20	11.1 ± 0.6	-0.2 ± 0.1	0.03663	11.1 ± 0.5	-0.2 ± 0.1	0.01581	11.0 ± 0.7	-0.2 ± 0.2	0.00630	10.9 ± 0.6	-0.3 ± 0.2	0.00004	10.7 ± 0.7	-0.4 ± 0.2	0.00013	10.9 ± 0.7	-0.3 ± 0.3	SS
L100	11.1 ± 0.6	-0.2 ± 0.2	0.01581	11.0 ± 0.5	-0.2 ± 0.2	0.00630	10.9 ± 0.7	-0.3 ± 0.2	0.00136	10.9 ± 0.6	-0.3 ± 0.2	0.00045	10.8 ± 0.7	-0.3 ± 0.2	0.00232	11.1 ± 0.7	-0.2 ± 0.2	0.00630
Aml5	11.4 ± 0.6	0.1 ± 0.1	0.21055	11.4 ± 0.5	0.1 ± 0.2	0.15454	11.3 ± 0.7	0.1 ± 0.2	0.05410	11.4 ± 0.6	0.1 ± 0.2	0.11113	11.3 ± 0.7	0.2 ± 0.2	0.11113	11.4 ± 0.6	0.1 ± 0.2	0.21055
B5	12.2 ± 0.8	0.9 ± 0.4	SS	12.1 ± 0.7	0.8 ± 0.4	SS	12.0 ± 0.9	0.8 ± 0.4	SS	12.1 ± 0.9	0.9 ± 0.5	SS	12.0 ± 0.9	0.9 ± 0.5	SS	12.5 ± 1.1	1.2 ± 0.8	SS
H12.5	11.2 ± 0.7	-0.2 ± 0.4	0.07832	11.1 ± 0.6	-0.2 ± 0.4	0.05410	11.1 ± 0.8	-0.1 ± 0.4	0.15454	11.1 ± 0.7	-0.1 ± 0.5	0.07832	11.0 ± 0.8	-0.1 ± 0.4	0.11113	11.1 ± 0.7	-0.1 ± 0.4	0.15454
Al300<br>Aml5	11.0 ± 0.6	-0.3 ± 0.3	0.00013	11.0 ± 0.6	-0.3 ± 0.3	0.00013	10.8 ± 0.7	-0.3 ± 0.3	0.00045	10.9 ± 0.6	-0.3 ± 0.3	0.00045	10.8 ± 0.7	-0.3 ± 0.3	0.00232	11.1 ± 0.7	-0.2 ± 0.3	0.00136
Al300<br>B5	13.0 ± 1.2	1.7 ± 0.8	SS	12.7 ± 1.1	1.4 ± 0.8	SS	12.3 ± 1.1	1.1 ± 0.7	SS	11.7 ± 0.9	0.5 ± 0.5	SS	11.4 ± 0.9	0.3 ± 0.5	0.00136	11.6 ± 1.0	0.3 ± 0.6	SS
Al300<br>H12.5	10.6 ± 0.7	-0.7 ± 0.5	SS	10.6 ± 0.7	-0.7 ± 0.5	SS	10.5 ± 0.8	-0.7 ± 0.5	SS	10.7 ± 0.8	-0.5 ± 0.6	SS	10.6 ± 0.9	-0.5 ± 0.6	SS	10.9 ± 0.8	-0.3 ± 0.5	0.00136
E20<br>Aml5	11.1 ± 0.6	-0.2 ± 0.3	0.00386	11.0 ± 0.5	-0.2 ± 0.3	0.00136	10.9 ± 0.7	-0.3 ± 0.3	0.00386	10.7 ± 0.7	-0.5 ± 0.4	SS	10.6 ± 0.7	-0.5 ± 0.4	SS	10.7 ± 0.7	-0.5 ± 0.4	SS
E20<br>B5	12.6 ± 1.0	1.2 ± 0.6	SS	12.4 ± 0.9	1.1 ± 0.7	SS	12.2 ± 1.1	1.0 ± 0.6	SS	11.7 ± 0.9	0.5 ± 0.6	SS	11.4 ± 1.0	0.2 ± 0.6	0.00079	11.5 ± 1.0	0.2 ± 0.8	0.00025
E20<br>H12.5	10.8 ± 0.7	-0.5 ± 0.4	SS	10.7 ± 0.6	-0.6 ± 0.5	SS	10.6 ± 0.8	-0.6 ± 0.5	SS	10.5 ± 0.8	-0.7 ± 0.6	SS	10.4 ± 0.9	-0.8 ± 0.7	SS	10.6 ± 0.8	-0.7 ± 0.5	SS
L100<br>Aml5	11.0 ± 0.7	-0.3 ± 0.4	0.00013	10.9 ± 0.6	-0.3 ± 0.4	0.00004	10.8 ± 0.7	-0.4 ± 0.4	0.00007	10.8 ± 0.6	-0.4 ± 0.3	0.00004	10.7 ± 0.7	-0.4 ± 0.4	0.00013	10.9 ± 0.7	-0.3 ± 0.3	0.00004
L100<br>B5	13.3 ± 1.3	2.0 ± 1.0	SS	12.9 ± 1.2	1.6 ± 1.0	SS	12.5 ± 1.2	1.3 ± 0.8	SS	11.7 ± 0.9	0.5 ± 0.6	SS	11.4 ± 0.9	0.2 ± 0.5	0.00079	11.6 ± 1.0	0.3 ± 0.7	0.00004
L100<br>H12.5	10.6 ± 0.7	-0.7 ± 0.5	SS	10.5 ± 0.7	-0.7 ± 0.5	SS	10.4 ± 0.8	-0.8 ± 0.6	SS	10.6 ± 0.8	-0.6 ± 0.6	SS	10.5 ± 0.9	-0.6 ± 0.6	SS	10.8 ± 0.8	-0.4 ± 0.5	SS
Al300<br>Aml5/B5	12.3 ± 1.1	1.0 ± 0.8	SS	12.0 ± 1.0	0.7 ± 0.7	SS	11.7 ± 1.1	0.5 ± 0.7	SS	11.1 ± 0.8	-0.1 ± 0.5	0.05410	10.8 ± 0.8	-0.3 ± 0.5	0.00045	10.9 ± 0.9	-0.3 ± 0.6	0.00079
Al300<br>Aml5/H12.5	10.3 ± 0.8	-1.0 ± 0.7	SS	10.2 ± 0.8	-1.0 ± 0.8	SS	10.2 ± 0.9	-1.0 ± 0.8	SS	10.4 ± 0.9	-0.9 ± 0.8	SS	10.4 ± 1.0	-0.8 ± 0.9	SS	10.6 ± 0.8	-0.6 ± 0.7	SS
Al300<br>B5/H12.5	11.3 ± 1.0	-0.0 ± 0.8	0.02431	11.1 ± 0.9	-0.2 ± 0.7	0.00630	10.8 ± 1.1	-0.4 ± 0.9	0.00025	10.6 ± 0.9	-0.7 ± 0.8	SS	10.4 ± 1.0	-0.8 ± 0.8	SS	10.6 ± 0.9	-0.7 ± 0.7	SS
E20<br>Aml5/B5	12.0 ± 0.9	0.7 ± 0.6	SS	11.8 ± 0.8	0.6 ± 0.6	SS	11.6 ± 1.0	0.4 ± 0.6	SS	11.0 ± 0.9	-0.2 ± 0.6	0.05410	10.7 ± 0.9	-0.4 ± 0.6	SS	10.7 ± 1.0	-0.6 ± 0.8	SS
E20<br>Aml5/H12.5	10.5 ± 0.8	-0.8 ± 0.6	SS	10.4 ± 0.8	-0.9 ± 0.7	SS	10.3 ± 0.9	-0.9 ± 0.8	SS	10.1 ± 0.9	-1.2 ± 0.9	SS	10.0 ± 1.1	-1.1 ± 0.9	SS	10.1 ± 0.9	-1.1 ± 0.8	SS
E20<br>B5/H12.5	11.2 ± 0.9	-0.1 ± 0.7	0.07832	11.0 ± 0.8	-0.3 ± 0.6	0.01008	10.8 ± 1.0	-0.4 ± 0.8	0.00013	10.4 ± 1.0	-0.8 ± 0.8	SS	10.2 ± 1.0	-1.0 ± 0.8	SS	10.2 ± 0.9	-1.0 ± 0.8	SS
L100<br>Aml5/B5	12.5 ± 1.2	1.2 ± 0.9	SS	12.2 ± 1.1	0.9 ± 0.8	SS	11.8 ± 1.1	0.6 ± 0.8	SS	11.0 ± 0.8	-0.2 ± 0.6	0.05410	10.7 ± 0.9	-0.4 ± 0.6	0.00002	10.8 ± 0.9	-0.4 ± 0.7	0.00007
L100<br>Aml5/H12.5	10.2 ± 0.9	-1.1 ± 0.7	SS	10.1 ± 0.9	-1.1 ± 0.8	SS	10.0 ± 1.0	-1.1 ± 0.8	SS	10.2 ± 0.9	-1.0 ± 0.8	SS	10.2 ± 1.0	-0.9 ± 0.9	SS	10.5 ± 0.8	-0.8 ± 0.7	SS
L100<br>B5/H12.5	11.4 ± 1.1	0.1 ± 0.8	0.00630	11.1 ± 0.9	-0.1 ± 0.7	0.01008	10.9 ± 1.1	-0.3 ± 0.9	0.00079	10.5 ± 0.9	-0.7 ± 0.8	SS	10.3 ± 1.0	-0.8 ± 0.8	SS	10.4 ± 0.9	-0.8 ± 0.8	SS

Al300 = aliskiren 300 mg; **Aml5** = amlodipine 5 mg; **B5** = bisoprolol 5 mg; **E20** = enalapril 20 mg; **H12.5** = hydrochlorothiazide 12.5 mg; **L100** = losartan 100 mg; **SS** = statistically significant (P < 0.00001)

Table S13. *P*-values calculated using the Kolmogorov-Smirnov test for changes in diastolic pulmonary arterial pressure in subpopulations (n = 100) with different ACE activity receiving the same regimens. Case 1: c_{ACE} = 7.0 h^{-1} (*LII*), case 2: c_{ACE} = 8.9 h^{-1} (*LID*), case 3: c_{ACE} = 10.8 h^{-1} (*LDD*), case 4: c_{ACE} = 42.3 h^{-1} (*HII*), case 5: c_{ACE} = 54.1 h^{-1} (*HID*), case 6: c_{ACE} = 65.9 h^{-1} (*HDD*). *P*-value for case *i vs*. case *j* is denoted P_{ij}.

Regimens	P_{12}	P_{13}	P_{23}	P_{14}	P_{15}	P_{16}	P_{24}	P_{25}	P_{26}	P_{34}	P_{35}	P_{36}	P_{45}	P_{46}	P_{56}
Al300	0.21055	0.00386	0.36672	0.03663	0.11113	0.03663	0.81275	0.81275	0.00386	0.69937	0.46756	0.00136	0.96707	0.00386	0.00630
E20	0.07832	0.00136	0.28093	SS	SS	SS	0.00004	SS	SS	0.00136	0.00013	0.00025	0.58062	0.58062	0.07832
L100	0.15454	0.00232	0.28093	0.00045	0.00025	0.21055	0.11113	0.11113	0.36672	0.96707	0.90621	0.07832	0.96707	0.02431	0.00630
Aml5	0.58062	0.58062	0.58062	0.15454	0.00386	0.03663	0.21055	0.03663	0.28093	0.36672	0.11113	0.36672	0.28093	0.81275	0.81275
B5	0.15454	0.46756	0.81275	0.99376	0.99376	0.00386	0.07832	0.03663	0.00013	0.36672	0.36672	0.00025	0.96707	0.01581	0.02431
H12.5	0.28093	0.90621	0.36672	0.96707	0.81275	0.58062	0.36672	0.07832	0.01581	0.96707	0.46756	0.21055	0.58062	0.46756	0.90621
Al300 Aml5	0.58062	0.36672	0.90621	0.69937	0.58062	0.28093	0.81275	0.11113	0.03663	0.96707	0.05410	0.03663	0.11113	0.02431	0.36672
Al300 B5	0.02431	0.00002	0.05410	SS	SS	SS	SS	SS	SS	SS	SS	SS	0.03663	0.28093	0.46756
Al300 H12.5	0.81275	0.58062	0.46756	0.05410	0.00025	SS	0.07832	0.00232	0.00007	0.15454	0.00013	0.00013	0.05410	0.02431	0.15454
E20 Aml5	0.28093	0.21055	0.36672	SS	SS	SS	0.00002	0.00002	SS	0.00004	0.00013	SS	0.96707	0.96707	0.90621
E20 B5	0.36672	0.01008	0.15454	SS	SS	SS	SS	SS	SS	0.00007	SS	SS	0.01581	0.02431	0.58062
E20 H12.5	0.99376	0.36672	0.58062	0.00386	0.05410	0.01581	0.01581	0.05410	0.07832	0.21055	0.11113	0.36672	0.46756	0.90621	0.90621
L100 Aml5	0.46756	0.36672	0.90621	0.07832	0.28093	0.81275	0.46756	0.69937	0.69937	0.81275	0.36672	0.58062	0.21055	0.36672	0.69937
L100 B5	0.02431	SS	0.03663	SS	SS	SS	SS	SS	SS	SS	SS	SS	0.03663	0.03663	0.58062
L100 H12.5	0.90621	0.36672	0.46756	0.15454	0.00232	0.00045	0.21055	0.03663	0.00136	0.28093	0.00386	0.00079	0.07832	0.03663	0.46756
Al300 Aml5/B5	0.02431	0.00045	0.11113	SS	SS	SS	SS	SS	SS	SS	SS	SS	0.03663	0.15454	0.58062
Al300 Aml5/H12.5	0.69937	0.69937	0.96707	0.03663	0.00013	0.00025	0.11113	0.01008	0.01581	0.05410	0.00232	0.00386	0.21055	0.15454	0.99376
Al300 B5/H12.5	0.36672	0.00630	0.07832	SS	SS	SS	0.00079	0.00004	0.00004	0.03663	0.00136	0.01581	0.69937	0.90621	0.58062
E20 Aml5/B5	0.11113	0.01581	0.36672	SS	SS	SS	SS	SS	SS	SS	SS	SS	0.03663	0.00386	0.28093
E20 Aml5/H12.5	0.81275	0.21055	0.69937	0.00232	0.03663	0.00079	0.03663	0.21055	0.01581	0.21055	0.58062	0.03663	0.58062	0.81275	0.46756
E20 B5/H12.5	0.36672	0.00630	0.05410	SS	SS	SS	SS	SS	SS	0.00386	0.00004	SS	0.46756	0.11113	0.21055
L100 Aml5/B5	0.03663	0.00007	0.05410	SS	SS	SS	SS	SS	SS	SS	SS	SS	0.02431	0.05410	0.28093
L100 Aml5/H12.5	0.81275	0.58062	0.96707	0.03663	0.00232	0.01581	0.15454	0.01581	0.05410	0.15454	0.00386	0.02431	0.21055	0.36672	0.96707
L100 B5/H12.5	0.15454	0.01008	0.03663	SS	SS	SS	0.00007	SS	SS	0.00386	0.00013	0.00136	0.58062	0.69937	0.46756

Al300 = aliskiren 300 mg; **Aml5** = amlodipine 5 mg; **B5** = bisoprolol 5 mg; **E20** = enalapril 20 mg; **H12.5** = hydrochlorothiazide 12.5 mg; **L100** = losartan 100 mg; **SS** = statistically significant (P < 0.00001)

Figure S14. Simulated change in systolic pulmonary arterial pressure from baseline to week 4 (mean ± SD, n = 100)

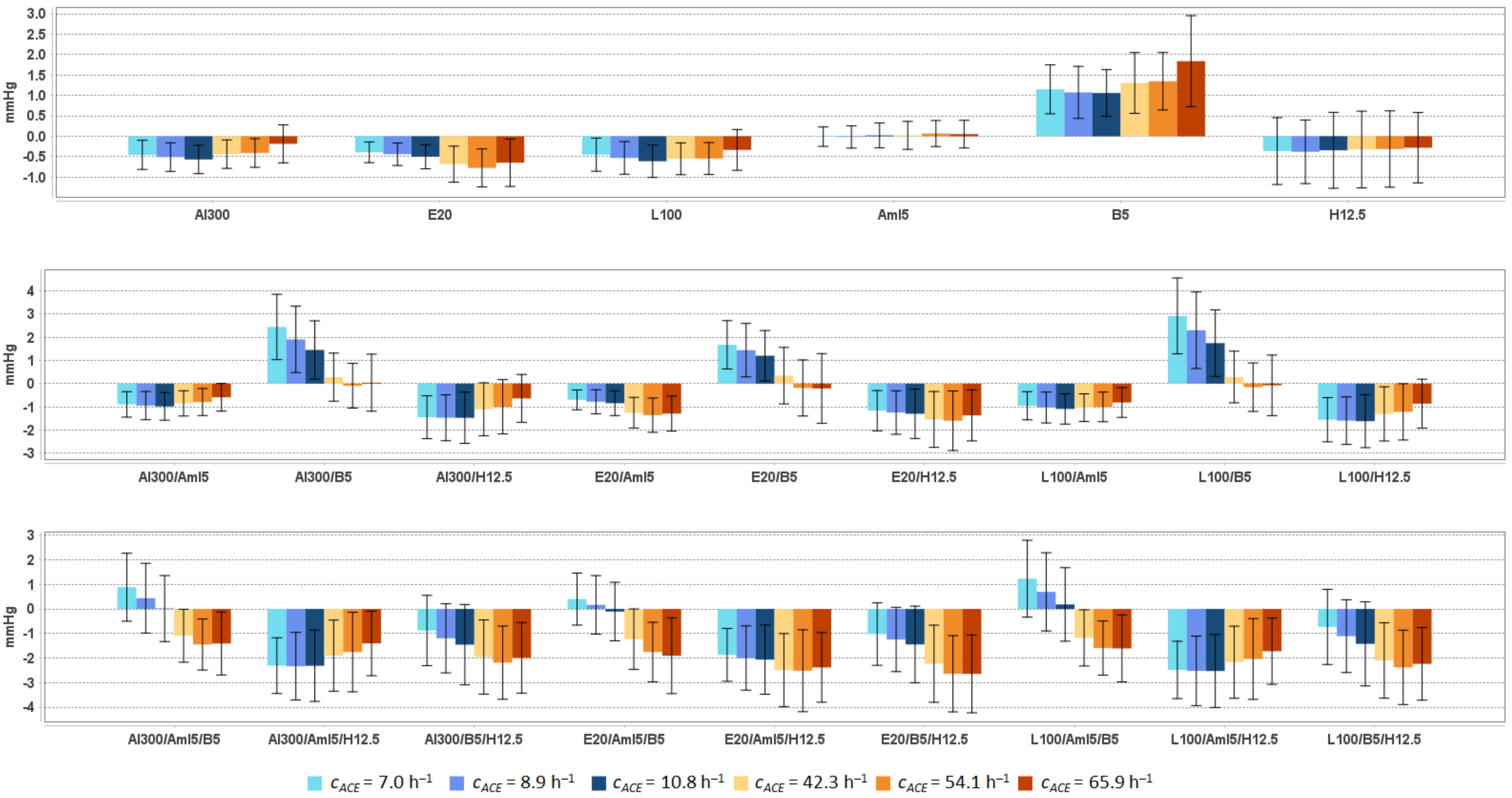

Al300 = aliskiren 300 mg; **Aml5** = amlodipine 5 mg; **B5** = bisoprolol 5 mg; **E20** = enalapril 20 mg; **H12.5** = hydrochlorothiazide 12.5 mg; **L100** = losartan 100 mg

Table S14. Simulated response of <u>systolic pulmonary arterial pressure</u> to antihypertensive therapy in virtual hypertensive subpopulations (n = 100) with different ACE activity, including P-values (Kolmogorov-Smirnov test) for endpoint *vs.* baseline; data are presented as mean ± SD in mmHg

Regimens	***LII*** **(c_{ACE} = 7.0 h^{-1})**			***LID*** **(c_{ACE} = 8.9 h^{-1})**			***LDD*** **(c_{ACE} = 10.8 h^{-1})**			***HII*** **(c_{ACE} = 42.3 h^{-1})**			***HID*** **(c_{ACE} = 54.1 h^{-1})**			***HDD*** **(c_{ACE} = 65.9 h^{-1})**		
	**Value**	**Change**	***P***	**Value**	**Change**	***P***	**Value**	**Change**	***P***	**Value**	**Change**	***P***	**Value**	**Change**	***P***	**Value**	**Change**	***P***
Baseline	17.8 ± 1.5	–	–	17.6 ± 1.3	–	–	17.5 ± 1.4	–	–	17.4 ± 1.4	–	–	17.7 ± 1.6	–	–	17.8 ± 1.5	–	–
Al300	17.3 ± 1.5	-0.5 ± 0.4	0.11113	17.1 ± 1.2	-0.5 ± 0.4	0.05410	17.0 ± 1.4	-0.6 ± 0.3	0.05410	16.9 ± 1.3	-0.4 ± 0.3	0.05410	17.3 ± 1.5	-0.4 ± 0.4	0.15454	17.6 ± 1.5	-0.2 ± 0.5	0.28093
E20	17.4 ± 1.4	-0.4 ± 0.3	0.21055	17.1 ± 1.2	-0.4 ± 0.3	0.11113	17.0 ± 1.4	-0.5 ± 0.3	0.11113	16.7 ± 1.3	-0.7 ± 0.4	0.00136	16.9 ± 1.5	-0.8 ± 0.5	0.01008	17.1 ± 1.4	-0.6 ± 0.6	0.00630
L100	17.3 ± 1.5	-0.5 ± 0.4	0.11113	17.0 ± 1.3	-0.5 ± 0.4	0.03663	16.9 ± 1.4	-0.6 ± 0.4	0.01581	16.8 ± 1.3	-0.6 ± 0.4	0.01008	17.2 ± 1.5	-0.5 ± 0.4	0.07832	17.5 ± 1.4	-0.3 ± 0.5	0.07832
Aml5	17.7 ± 1.5	-0.0 ± 0.2	0.99376	17.5 ± 1.3	-0.0 ± 0.3	0.99376	17.6 ± 1.5	0.0 ± 0.3	0.99963	17.4 ± 1.4	0.0 ± 0.3	0.96707	17.8 ± 1.6	0.1 ± 0.3	0.99963	17.8 ± 1.6	0.1 ± 0.3	0.99376
B5	18.9 ± 1.6	1.2 ± 0.6	SS	18.6 ± 1.5	1.1 ± 0.6	SS	18.6 ± 1.6	1.1 ± 0.6	0.00025	18.7 ± 1.5	1.3 ± 0.7	SS	19.1 ± 1.7	1.3 ± 0.7	0.00004	19.6 ± 1.8	1.8 ± 1.1	SS
H12.5	17.4 ± 1.7	-0.4 ± 0.8	0.21055	17.2 ± 1.3	-0.4 ± 0.8	0.21055	17.2 ± 1.6	-0.3 ± 0.9	0.21055	17.0 ± 1.6	-0.3 ± 0.9	0.15454	17.4 ± 1.7	-0.3 ± 0.9	0.36672	17.5 ± 1.7	-0.3 ± 0.9	0.46756
Al300<br>Aml5	16.9 ± 1.5	-0.9 ± 0.5	0.00136	16.6 ± 1.3	-0.9 ± 0.6	0.00007	16.6 ± 1.5	-1.0 ± 0.6	0.00013	16.5 ± 1.3	-0.8 ± 0.5	0.00025	16.9 ± 1.6	-0.8 ± 0.6	0.01008	17.2 ± 1.4	-0.6 ± 0.6	0.01581
Al300<br>B5	20.2 ± 2.1	2.4 ± 1.4	SS	19.5 ± 2.0	1.9 ± 1.4	SS	19.0 ± 2.0	1.5 ± 1.3	0.00004	17.6 ± 1.5	0.3 ± 1.0	0.46756	17.6 ± 1.7	-0.1 ± 1.0	0.90621	17.8 ± 1.7	0.0 ± 1.2	0.69937
Al300<br>H12.5	16.3 ± 1.7	-1.4 ± 0.9	SS	16.1 ± 1.4	-1.5 ± 1.0	SS	16.1 ± 1.6	-1.5 ± 1.1	SS	16.3 ± 1.6	-1.1 ± 1.1	SS	16.7 ± 1.7	-1.0 ± 1.2	0.00007	17.2 ± 1.7	-0.6 ± 1.0	0.05410
E20<br>Aml5	17.1 ± 1.5	-0.7 ± 0.4	0.01581	16.8 ± 1.2	-0.8 ± 0.5	0.00232	16.7 ± 1.5	-0.8 ± 0.5	0.00232	16.1 ± 1.4	-1.3 ± 0.7	SS	16.3 ± 1.6	-1.4 ± 0.7	0.00004	16.5 ± 1.4	-1.3 ± 0.8	0.00004
E20<br>B5	19.4 ± 1.9	1.7 ± 1.0	SS	19.0 ± 1.8	1.4 ± 1.2	SS	18.7 ± 1.9	1.2 ± 1.1	0.00025	17.7 ± 1.7	0.3 ± 1.2	0.21055	17.5 ± 1.8	-0.2 ± 1.2	0.46756	17.6 ± 1.8	-0.2 ± 1.5	0.36672
E20<br>H12.5	16.6 ± 1.6	-1.2 ± 0.9	0.00002	16.3 ± 1.3	-1.2 ± 0.9	SS	16.2 ± 1.6	-1.3 ± 1.1	0.00004	15.8 ± 1.6	-1.5 ± 1.2	SS	16.1 ± 1.8	-1.6 ± 1.3	SS	16.4 ± 1.7	-1.4 ± 1.1	0.00002
L100<br>Aml5	16.8 ± 1.5	-1.0 ± 0.6	0.00136	16.5 ± 1.3	-1.0 ± 0.7	0.00007	16.4 ± 1.5	-1.1 ± 0.7	0.00004	16.3 ± 1.4	-1.0 ± 0.6	SS	16.7 ± 1.6	-1.0 ± 0.6	0.00232	17.0 ± 1.4	-0.8 ± 0.6	0.00136
L100<br>B5	20.7 ± 2.3	2.9 ± 1.6	SS	19.9 ± 2.2	2.3 ± 1.6	SS	19.3 ± 2.1	1.7 ± 1.4	SS	17.7 ± 1.6	0.3 ± 1.1	0.28093	17.6 ± 1.7	-0.2 ± 1.0	0.69937	17.7 ± 1.7	-0.1 ± 1.3	0.46756
L100<br>H12.5	16.2 ± 1.7	-1.6 ± 1.0	SS	16.0 ± 1.4	-1.6 ± 1.0	SS	15.9 ± 1.7	-1.6 ± 1.1	SS	16.1 ± 1.6	-1.3 ± 1.2	SS	16.5 ± 1.8	-1.2 ± 1.2	0.00002	16.9 ± 1.7	-0.9 ± 1.1	0.01581
Al300<br>Aml5/B5	18.6 ± 2.1	0.9 ± 1.4	0.00045	18.0 ± 1.9	0.4 ± 1.4	0.07832	17.6 ± 2.0	0.0 ± 1.3	0.28093	16.3 ± 1.5	-1.1 ± 1.1	SS	16.3 ± 1.7	-1.4 ± 1.0	0.00002	16.4 ± 1.6	-1.4 ± 1.3	SS
Al300<br>Aml5/H12.5	15.4 ± 1.8	-2.3 ± 1.1	SS	15.2 ± 1.6	-2.3 ± 1.4	SS	15.2 ± 1.9	-2.3 ± 1.4	SS	15.5 ± 1.8	-1.9 ± 1.4	SS	15.9 ± 2.0	-1.8 ± 1.6	SS	16.4 ± 1.9	-1.4 ± 1.3	SS
Al300<br>B5/H12.5	16.9 ± 2.0	-0.9 ± 1.4	0.00079	16.4 ± 1.7	-1.2 ± 1.4	SS	16.1 ± 2.0	-1.5 ± 1.6	SS	15.4 ± 1.8	-2.0 ± 1.5	SS	15.5 ± 1.8	-2.2 ± 1.5	SS	15.8 ± 1.7	-2.0 ± 1.4	SS
E20<br>Aml5/B5	18.2 ± 1.8	0.4 ± 1.1	0.01581	17.7 ± 1.7	0.2 ± 1.2	0.46756	17.4 ± 1.9	-0.1 ± 1.2	0.28093	16.1 ± 1.6	-1.2 ± 1.2	SS	15.9 ± 1.8	-1.8 ± 1.2	SS	15.9 ± 1.8	-1.9 ± 1.5	SS
E20<br>Aml5/H12.5	15.9 ± 1.8	-1.9 ± 1.1	SS	15.6 ± 1.5	-2.0 ± 1.3	SS	15.5 ± 1.9	-2.1 ± 1.4	SS	14.9 ± 1.8	-2.5 ± 1.5	SS	15.2 ± 2.0	-2.5 ± 1.7	SS	15.4 ± 1.9	-2.4 ± 1.4	SS
E20<br>B5/H12.5	16.7 ± 1.9	-1.0 ± 1.3	0.00025	16.3 ± 1.6	-1.2 ± 1.3	SS	16.1 ± 2.0	-1.4 ± 1.6	SS	15.1 ± 1.8	-2.2 ± 1.6	SS	15.1 ± 1.8	-2.6 ± 1.6	SS	15.1 ± 1.8	-2.6 ± 1.6	SS
L100<br>Aml5/B5	19.0 ± 2.2	1.2 ± 1.6	SS	18.3 ± 2.1	0.7 ± 1.6	0.01581	17.7 ± 2.1	0.2 ± 1.5	0.36672	16.2 ± 1.6	-1.2 ± 1.1	SS	16.1 ± 1.7	-1.6 ± 1.1	SS	16.2 ± 1.7	-1.6 ± 1.4	SS
L100<br>Aml5/H12.5	15.3 ± 1.8	-2.5 ± 1.2	SS	15.0 ± 1.6	-2.5 ± 1.4	SS	15.0 ± 1.9	-2.5 ± 1.5	SS	15.2 ± 1.8	-2.2 ± 1.5	SS	15.7 ± 2.0	-2.0 ± 1.6	SS	16.1 ± 1.9	-1.7 ± 1.3	SS
L100<br>B5/H12.5	17.0 ± 2.1	-0.7 ± 1.5	0.00386	16.5 ± 1.8	-1.1 ± 1.5	SS	16.1 ± 2.1	-1.4 ± 1.7	SS	15.3 ± 1.8	-2.1 ± 1.5	SS	15.3 ± 1.8	-2.4 ± 1.5	SS	15.6 ± 1.8	-2.2 ± 1.5	SS

Al300 = aliskiren 300 mg; **Aml5** = amlodipine 5 mg; **B5** = bisoprolol 5 mg; **E20** = enalapril 20 mg; **H12.5** = hydrochlorothiazide 12.5 mg; **L100** = losartan 100 mg; **SS** = statistically significant (P < 0.00001)

Table S15. *P*-values calculated using the Kolmogorov-Smirnov test for changes in systolic pulmonary arterial pressure in subpopulations (n = 100) with different ACE activity receiving the same regimens. Case 1: c_{ACE} = 7.0 h^{-1} (*LII*), case 2: c_{ACE} = 8.9 h^{-1} (*LID*), case 3: c_{ACE} = 10.8 h^{-1} (*LDD*), case 4: c_{ACE} = 42.3 h^{-1} (*HII*), case 5: c_{ACE} = 54.1 h^{-1} (*HID*), case 6: c_{ACE} = 65.9 h^{-1} (*HDD*). *P*-value for case *i* *vs*. case *j* is denoted P_{ij}.

Regimens	P_{12}	P_{13}	P_{23}	P_{14}	P_{15}	P_{16}	P_{24}	P_{25}	P_{26}	P_{34}	P_{35}	P_{36}	P_{45}	P_{46}	P_{56}
Al300	0.36672	0.00630	0.11113	0.81275	0.58062	0.00013	0.58062	0.28093	0.00002	0.01581	0.00386	SS	0.90621	0.00136	0.00232
E20	0.21055	0.00136	0.07832	SS	SS	SS	0.00007	SS	0.00004	0.00386	0.00004	0.00232	0.58062	0.58062	0.15454
L100	0.28093	0.00386	0.11113	0.11113	0.11113	0.07832	0.81275	0.90621	0.00630	0.58062	0.36672	0.00079	0.99376	0.01008	0.00630
Aml5	0.58062	0.69937	0.69937	0.15454	0.00630	0.11113	0.21055	0.02431	0.15454	0.81275	0.15454	0.69937	0.28093	0.90621	0.69937
B5	0.28093	0.58062	0.69937	0.46756	0.28093	0.00079	0.01008	0.00630	SS	0.03663	0.01581	SS	0.90621	0.01008	0.00630
H12.5	0.58062	0.69937	0.28093	0.69937	0.81275	0.69937	0.36672	0.11113	0.05410	0.99376	0.46756	0.36672	0.28093	0.69937	0.99963
Al300<br>Aml5	0.58062	0.36672	0.96707	0.28093	0.21055	0.00386	0.15454	0.01581	0.00045	0.21055	0.00136	0.00079	0.11113	0.00386	0.11113
Al300<br>B5	0.01581	0.00004	0.02431	SS	SS	SS	SS	SS	SS	SS	SS	SS	0.05410	0.21055	0.21055
Al300<br>H12.5	0.90621	0.46756	0.36672	0.02431	0.00007	SS	0.05410	0.00136	SS	0.07832	0.00013	SS	0.07832	0.01008	0.36672
E20<br>Aml5	0.15454	0.11113	0.58062	SS	SS	SS	SS	SS	SS	0.00013	0.00007	SS	0.69937	0.90621	0.69937
E20<br>B5	0.36672	0.00232	0.01581	SS	SS	SS	SS	SS	SS	0.00007	SS	SS	0.02431	0.01008	0.69937
E20<br>H12.5	0.69937	0.21055	0.28093	0.00386	0.11113	0.05410	0.00232	0.21055	0.03663	0.15454	0.15454	0.69937	0.46756	0.58062	0.81275
L100<br>Aml5	0.58062	0.11113	0.81275	0.36672	0.81275	0.36672	0.81275	0.36672	0.07832	0.69937	0.03663	0.07832	0.28093	0.07832	0.21055
L100<br>B5	0.02431	SS	0.01581	SS	SS	SS	SS	SS	SS	SS	SS	SS	0.01581	0.07832	0.28093
L100<br>H12.5	0.90621	0.21055	0.36672	0.15454	0.00079	0.00002	0.11113	0.00630	0.00013	0.15454	0.00136	0.00013	0.07832	0.03663	0.28093
Al300<br>Aml5/B5	0.02431	0.00045	0.11113	SS	SS	SS	SS	SS	SS	SS	SS	SS	0.01581	0.15454	0.81275
Al300<br>Aml5/H12.5	0.58062	0.58062	0.96707	0.00386	0.00007	SS	0.07832	0.01008	0.00045	0.05410	0.00232	0.00045	0.28093	0.07832	0.69937
Al300<br>B5/H12.5	0.36672	0.00630	0.28093	0.00007	SS	SS	0.00630	0.00136	0.00025	0.11113	0.00386	0.03663	0.58062	0.90621	0.69937
E20<br>Aml5/B5	0.07832	0.01581	0.28093	SS	SS	SS	SS	SS	SS	0.00002	SS	SS	0.00630	0.02431	0.36672
E20<br>Aml5/H12.5	0.46756	0.21055	0.81275	0.00079	0.01008	0.00232	0.05410	0.28093	0.07832	0.21055	0.46756	0.15454	0.58062	0.58062	0.69937
E20<br>B5/H12.5	0.36672	0.00630	0.46756	SS	SS	SS	0.00013	SS	SS	0.01581	0.00013	0.00007	0.46756	0.11113	0.46756
L100<br>Aml5/B5	0.03663	0.00025	0.05410	SS	SS	SS	SS	SS	SS	SS	SS	SS	0.01581	0.07832	0.81275
L100<br>Aml5/H12.5	0.58062	0.58062	0.96707	0.00630	0.00045	0.00013	0.15454	0.02431	0.00630	0.07832	0.01008	0.00136	0.36672	0.11113	0.90621
L100<br>B5/H12.5	0.36672	0.00630	0.28093	SS	SS	SS	0.00079	0.00007	SS	0.03663	0.00136	0.00630	0.58062	0.58062	0.69937

Al300 = aliskiren 300 mg; **Aml5** = amlodipine 5 mg; **B5** = bisoprolol 5 mg; **E20** = enalapril 20 mg; **H12.5** = hydrochlorothiazide 12.5 mg; **L100** = losartan 100 mg; **SS** = statistically significant ($P < 0.00001$)

Figure S15. Simulated change in <u>stroke volume</u> from baseline to week 4 (mean ± SD, n = 100)

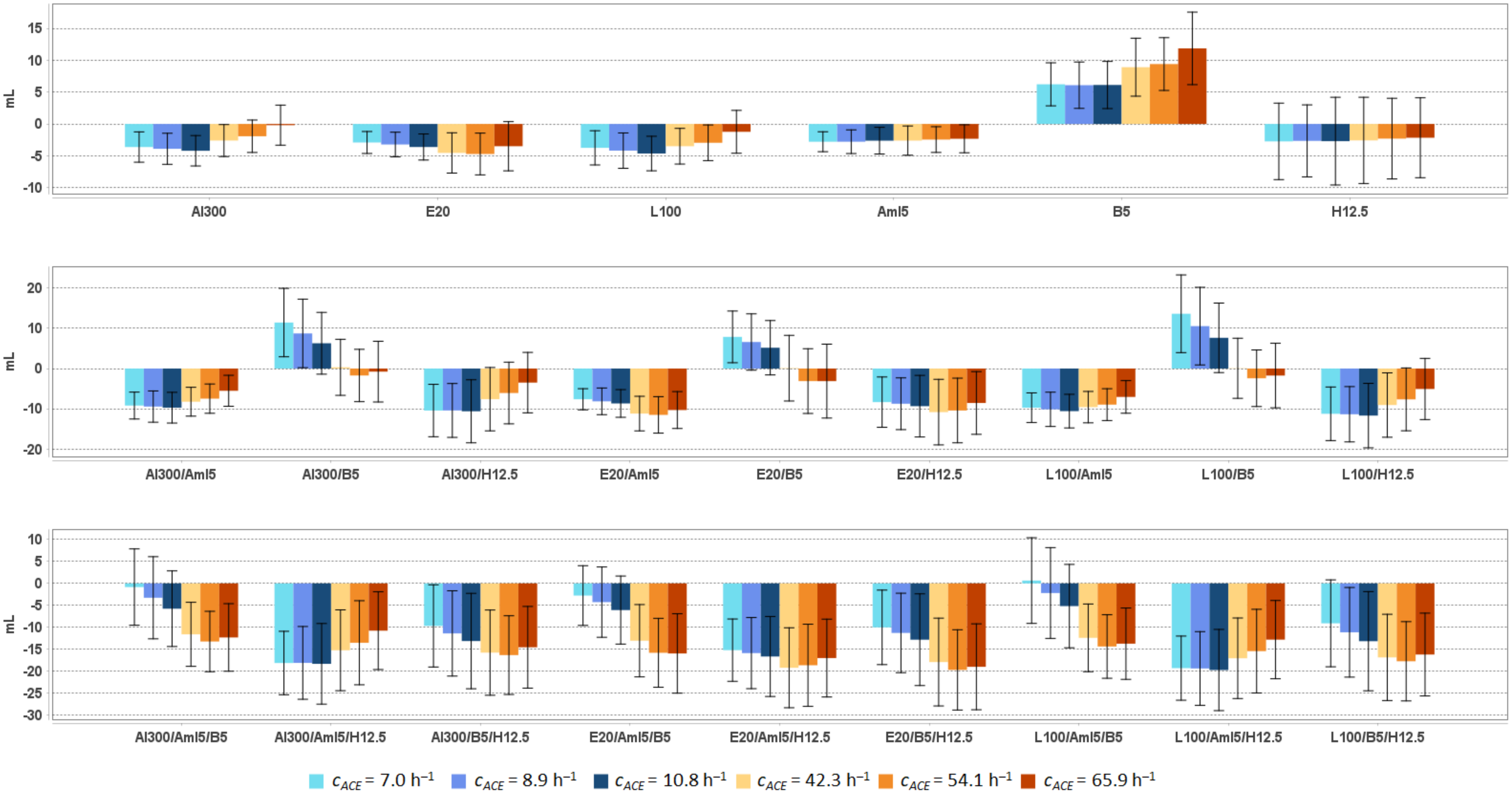

Al300 = aliskiren 300 mg; **Aml5** = amlodipine 5 mg; **B5** = bisoprolol 5 mg; **E20** = enalapril 20 mg; **H12.5** = hydrochlorothiazide 12.5 mg; **L100** = losartan 100 mg

Table S16. Simulated response of <u>stroke volume</u> to antihypertensive therapy in virtual hypertensive subpopulations (n = 100) with different ACE activity, including P-values (Kolmogorov-Smirnov test) for endpoint *vs*. baseline; data are presented as mean ± SD in mL

**Regimens**	***LII*** **(c_{ACE} = 7.0 h^{-1})**			***LID*** **(c_{ACE} = 8.9 h^{-1})**			***LDD*** **(c_{ACE} = 10.8 h^{-1})**			***HII*** **(c_{ACE} = 42.3 h^{-1})**			***HID*** **(c_{ACE} = 54.1 h^{-1})**			***HDD*** **(c_{ACE} = 65.9 h^{-1})**		
	**Value**	**Change**	***P***	**Value**	**Change**	***P***	**Value**	**Change**	***P***	**Value**	**Change**	***P***	**Value**	**Change**	***P***	**Value**	**Change**	***P***
Baseline	73.2 ± 6.9	–	–	72.5 ± 7.6	–	–	73.5 ± 7.8	–	–	72.8 ± 7.3	–	–	73.4 ± 7.9	–	–	71.8 ± 7.0	–	–
Al300	69.6 ± 7.0	-3.6 ± 2.4	0.00079	68.6 ± 8.0	-3.9 ± 2.4	0.02431	69.3 ± 8.0	-4.2 ± 2.4	0.01008	70.2 ± 7.1	-2.6 ± 2.5	0.02431	71.5 ± 8.1	-1.9 ± 2.5	0.11113	71.7 ± 7.0	-0.2 ± 3.1	0.99963
E20	70.3 ± 6.9	-2.9 ± 1.7	0.00630	69.3 ± 7.8	-3.2 ± 1.9	0.07832	69.9 ± 7.9	-3.6 ± 2.0	0.02431	68.2 ± 7.2	-4.5 ± 3.2	0.00013	68.7 ± 8.1	-4.7 ± 3.3	0.00045	68.4 ± 7.0	-3.5 ± 3.9	0.01581
L100	69.5 ± 7.2	-3.8 ± 2.7	0.00079	68.3 ± 8.1	-4.2 ± 2.8	0.01581	68.9 ± 8.0	-4.6 ± 2.7	0.00386	69.3 ± 7.1	-3.5 ± 2.8	0.00232	70.5 ± 8.1	-3.0 ± 2.8	0.01581	70.6 ± 7.0	-1.2 ± 3.4	0.46756
Aml5	70.4 ± 7.1	-2.8 ± 1.6	0.01581	69.7 ± 7.8	-2.8 ± 1.9	0.07832	70.9 ± 7.8	-2.6 ± 2.1	0.11113	70.2 ± 8.0	-2.6 ± 2.3	0.11113	71.0 ± 8.0	-2.5 ± 2.0	0.01008	69.5 ± 7.6	-2.3 ± 2.2	0.05410
B5	79.5 ± 7.6	6.2 ± 3.4	SS	78.6 ± 8.8	6.1 ± 3.6	0.00045	79.6 ± 8.9	6.1 ± 3.7	SS	81.7 ± 8.2	8.9 ± 4.6	SS	82.8 ± 9.8	9.4 ± 4.1	SS	83.7 ± 8.7	11.9 ± 5.7	SS
H12.5	70.5 ± 9.1	-2.7 ± 6.0	0.02431	69.9 ± 9.4	-2.7 ± 5.7	0.07832	70.8 ± 10.5	-2.7 ± 6.9	0.07832	70.2 ± 10.0	-2.6 ± 6.8	0.11113	71.1 ± 10.5	-2.3 ± 6.3	0.15454	69.7 ± 9.1	-2.2 ± 6.3	0.11113
Al300 Aml5	64.0 ± 7.2	-9.2 ± 3.3	SS	63.1 ± 8.3	-9.4 ± 3.9	SS	63.8 ± 8.1	-9.7 ± 3.8	SS	64.6 ± 7.7	-8.2 ± 3.6	SS	66.0 ± 8.2	-7.5 ± 3.6	SS	66.3 ± 7.5	-5.5 ± 3.9	0.00002
Al300 B5	84.7 ± 11.2	11.4 ± 8.5	SS	81.3 ± 11.8	8.7 ± 8.5	SS	79.8 ± 10.8	6.3 ± 7.7	SS	73.1 ± 9.1	0.3 ± 7.0	0.96707	71.7 ± 10.1	-1.7 ± 6.5	0.07832	71.1 ± 9.1	-0.8 ± 7.5	0.58062
Al300 H12.5	62.8 ± 9.0	-10.4 ± 6.5	SS	62.1 ± 9.9	-10.4 ± 6.7	SS	62.9 ± 10.8	-10.6 ± 7.8	SS	65.2 ± 10.3	-7.6 ± 7.9	SS	67.4 ± 11.2	-6.1 ± 7.7	0.00002	68.4 ± 9.6	-3.5 ± 7.5	0.01008
E20 Aml5	65.6 ± 7.0	-7.6 ± 2.6	SS	64.4 ± 8.1	-8.1 ± 3.3	SS	64.8 ± 8.0	-8.7 ± 3.4	SS	61.6 ± 7.8	-11.2 ± 4.3	SS	61.9 ± 8.2	-11.5 ± 4.5	SS	61.5 ± 7.4	-10.3 ± 4.6	SS
E20 B5	81.1 ± 9.6	7.9 ± 6.4	SS	79.1 ± 10.7	6.6 ± 7.0	0.00045	78.7 ± 10.3	5.2 ± 6.8	0.00004	72.9 ± 9.9	0.1 ± 8.2	0.81275	70.3 ± 11.1	-3.1 ± 8.0	0.00630	68.7 ± 10.3	-3.1 ± 9.2	0.02431
E20 H12.5	64.9 ± 8.9	-8.3 ± 6.3	SS	63.8 ± 9.7	-8.7 ± 6.4	SS	64.2 ± 10.7	-9.3 ± 7.7	SS	62.0 ± 10.3	-10.8 ± 8.1	SS	63.0 ± 11.1	-10.4 ± 8.0	SS	63.3 ± 9.4	-8.5 ± 7.8	SS
L100 Aml5	63.5 ± 7.4	-9.7 ± 3.7	SS	62.4 ± 8.5	-10.1 ± 4.3	SS	62.9 ± 8.3	-10.6 ± 4.2	SS	63.2 ± 7.7	-9.6 ± 3.9	SS	64.5 ± 8.2	-9.0 ± 4.0	SS	64.8 ± 7.5	-7.0 ± 4.1	SS
L100 B5	86.9 ± 12.1	13.6 ± 9.7	SS	83.1 ± 12.6	10.5 ± 9.6	SS	81.1 ± 11.4	7.6 ± 8.6	SS	72.9 ± 9.4	0.1 ± 7.5	0.81275	71.0 ± 10.4	-2.4 ± 7.0	0.05410	70.1 ± 9.4	-1.7 ± 8.0	0.28093
L100 H12.5	62.0 ± 9.1	-11.2 ± 6.7	SS	61.2 ± 10.0	-11.4 ± 6.9	SS	61.8 ± 10.9	-11.7 ± 8.0	SS	63.7 ± 10.3	-9.1 ± 8.0	SS	65.8 ± 11.2	-7.7 ± 7.8	SS	66.8 ± 9.5	-5.1 ± 7.6	0.00004
Al300 Aml5/B5	72.4 ± 11.1	-0.8 ± 8.7	0.07832	69.3 ± 12.3	-3.3 ± 9.3	0.00232	67.8 ± 11.2	-5.7 ± 8.6	0.00025	61.2 ± 9.2	-11.6 ± 7.3	SS	60.2 ± 9.7	-13.2 ± 6.9	SS	59.6 ± 9.0	-12.3 ± 7.7	SS
Al300 Aml5/H12.5	55.1 ± 9.5	-18.1 ± 7.2	SS	54.5 ± 10.9	-18.1 ± 8.3	SS	55.2 ± 11.5	-18.3 ± 9.2	SS	57.6 ± 11.6	-15.2 ± 9.2	SS	59.9 ± 12.2	-13.5 ± 9.6	SS	61.1 ± 11.0	-10.7 ± 8.8	SS
Al300 B5/H12.5	63.6 ± 11.5	-9.7 ± 9.4	SS	61.2 ± 12.3	-11.4 ± 9.7	SS	60.4 ± 13.0	-13.1 ± 10.8	SS	57.0 ± 11.2	-15.7 ± 9.7	SS	57.1 ± 11.3	-16.3 ± 9.0	SS	57.3 ± 10.0	-14.5 ± 9.3	SS
E20 Aml5/B5	70.5 ± 9.6	-2.8 ± 6.8	0.00386	68.3 ± 11.3	-4.3 ± 8.0	0.00045	67.4 ± 10.6	-6.1 ± 7.7	0.00013	59.8 ± 9.8	-13.0 ± 8.2	SS	57.7 ± 10.3	-15.8 ± 7.8	SS	55.9 ± 9.8	-15.9 ± 9.0	SS
E20 Aml5/H12.5	58.1 ± 9.5	-15.2 ± 7.1	SS	56.7 ± 10.8	-15.8 ± 8.1	SS	56.9 ± 11.6	-16.6 ± 9.1	SS	53.6 ± 11.3	-19.2 ± 9.1	SS	54.8 ± 11.6	-18.6 ± 9.3	SS	54.9 ± 10.7	-17.0 ± 8.8	SS
E20 B5/H12.5	63.2 ± 10.7	-10.0 ± 8.5	SS	61.3 ± 11.7	-11.3 ± 9.0	SS	60.7 ± 12.7	-12.8 ± 10.4	SS	54.9 ± 11.3	-17.9 ± 10.0	SS	53.8 ± 11.1	-19.7 ± 9.1	SS	52.9 ± 10.2	-19.0 ± 9.8	SS
L100 Aml5/B5	73.9 ± 11.9	0.6 ± 9.7	0.21055	70.3 ± 13.1	-2.2 ± 10.3	0.00232	68.3 ± 11.8	-5.2 ± 9.5	0.00079	60.4 ± 9.4	-12.4 ± 7.7	SS	59.1 ± 9.9	-14.3 ± 7.2	SS	58.1 ± 9.2	-13.7 ± 8.1	SS
L100 Aml5/H12.5	54.0 ± 9.5	-19.3 ± 7.3	SS	53.2 ± 11.0	-19.3 ± 8.4	SS	53.8 ± 11.5	-19.7 ± 9.2	SS	55.8 ± 11.4	-17.0 ± 9.2	SS	58.0 ± 12.0	-15.4 ± 9.5	SS	59.1 ± 11.0	-12.8 ± 8.9	SS
L100 B5/H12.5	64.2 ± 12.0	-9.1 ± 9.9	SS	61.4 ± 12.7	-11.1 ± 10.2	SS	60.4 ± 13.4	-13.1 ± 11.3	SS	56.0 ± 11.2	-16.8 ± 9.8	SS	55.7 ± 11.2	-17.7 ± 9.0	SS	55.7 ± 10.0	-16.2 ± 9.4	SS

Al300 = aliskiren 300 mg; **Aml5** = amlodipine 5 mg; **B5** = bisoprolol 5 mg; **E20** = enalapril 20 mg; **H12.5** = hydrochlorothiazide 12.5 mg; **L100** = losartan 100 mg; **SS** = statistically significant (P < 0.00001)

Table S17. *P*-values calculated using the Kolmogorov-Smirnov test for changes in stroke volume in subpopulations (n = 100) with different ACE activity receiving the same regimens. Case 1: c_{ACE} = 7.0 h^{-1} (*LII*), case 2: c_{ACE} = 8.9 h^{-1} (*LID*), case 3: c_{ACE} = 10.8 h^{-1} (*LDD*), case 4: c_{ACE} = 42.3 h^{-1} (*HII*), case 5: c_{ACE} = 54.1 h^{-1} (*HID*), case 6: c_{ACE} =65.9 h^{-1} (*HDD*). *P*-value for case *i* *vs*. case *j* is denoted P_{ij}.

Regimens	P_{12}	P_{13}	P_{23}	P_{14}	P_{15}	P_{16}	P_{24}	P_{25}	P_{26}	P_{34}	P_{35}	P_{36}	P_{45}	P_{46}	P_{56}
Al300	0.58062	0.00630	0.36672	0.05410	0.00045	SS	0.03663	0.00045	SS	0.00045	SS	SS	0.15454	0.00007	0.00079
E20	0.28093	0.00136	0.07832	SS	SS	0.00079	0.00013	0.00025	0.00386	0.00136	0.00232	0.03663	0.99376	0.21055	0.11113
L100	0.36672	0.00386	0.36672	0.69937	0.11113	SS	0.36672	0.03663	SS	0.02431	0.00013	SS	0.28093	0.00079	0.01581
Aml5	0.96707	0.21055	0.36672	0.21055	0.07832	0.11113	0.58062	0.15454	0.28093	0.96707	0.36672	0.46756	0.81275	0.69937	0.81275
B5	0.69937	0.81275	0.96707	0.00002	SS	SS	SS	SS	SS	SS	SS	SS	0.46756	0.00386	0.00386
H12.5	0.69937	0.58062	0.46756	0.36672	0.58062	0.81275	0.15454	0.15454	0.15454	0.90621	0.46756	0.46756	0.21055	0.69937	0.99376
Al300<br>Aml5	0.81275	0.36672	0.69937	0.28093	0.00386	SS	0.05410	0.00045	SS	0.02431	0.00045	SS	0.15454	0.00002	0.02431
Al300<br>B5	0.03663	0.00025	0.05410	SS	SS	SS	SS	SS	SS	0.00025	SS	SS	0.05410	0.21055	0.46756
Al300<br>H12.5	0.99376	0.58062	0.46756	0.01581	0.00013	SS	0.01581	0.00013	SS	0.07832	0.00013	SS	0.21055	0.00386	0.11113
E20<br>Aml5	0.28093	0.03663	0.36672	SS	SS	SS	SS	SS	0.00007	0.00025	0.00079	0.00630	0.90621	0.58062	0.46756
E20<br>B5	0.46756	0.01008	0.28093	SS	SS	SS	SS	SS	SS	0.00025	SS	SS	0.02431	0.03663	0.81275
E20<br>H12.5	0.81275	0.05410	0.46756	0.01581	0.05410	0.46756	0.05410	0.28093	0.81275	0.15454	0.58062	0.28093	0.58062	0.11113	0.58062
L100<br>Aml5	0.46756	0.15454	0.21055	0.69937	0.21055	0.00232	0.69937	0.02431	0.00004	0.11113	0.00386	SS	0.15454	0.00045	0.05410
L100<br>B5	0.03663	0.00013	0.05410	SS	SS	SS	SS	SS	SS	SS	SS	SS	0.03663	0.21055	0.58062
L100<br>H12.5	0.96707	0.46756	0.46756	0.11113	0.00079	SS	0.03663	0.00079	SS	0.11113	0.00136	SS	0.28093	0.00630	0.15454
Al300<br>Aml5/B5	0.05410	0.00136	0.28093	SS	SS	SS	SS	SS	SS	0.00013	SS	SS	0.05410	0.58062	0.69937
Al300<br>Aml5/H12.5	0.58062	0.58062	0.81275	0.01008	0.00013	SS	0.01008	0.00079	SS	0.11113	0.00386	SS	0.28093	0.00232	0.11113
Al300<br>B5/H12.5	0.46756	0.00630	0.11113	0.00136	0.00025	0.00045	0.01581	0.01581	0.03663	0.21055	0.01581	0.28093	0.81275	0.58062	0.58062
E20<br>Aml5/B5	0.11113	0.00630	0.28093	SS	SS	SS	SS	SS	SS	SS	SS	SS	0.03663	0.11113	0.58062
E20<br>Aml5/H12.5	0.46756	0.15454	0.69937	0.00232	0.00630	0.05410	0.01581	0.03663	0.46756	0.28093	0.58062	0.81275	0.81275	0.21055	0.46756
E20<br>B5/H12.5	0.58062	0.00630	0.11113	SS	SS	SS	0.00013	SS	SS	0.00232	0.00013	0.00045	0.58062	0.28093	0.69937
L100<br>Aml5/B5	0.05410	0.00079	0.15454	SS	SS	SS	SS	SS	SS	0.00002	SS	SS	0.05410	0.46756	0.90621
L100<br>Aml5/H12.5	0.58062	0.36672	0.69937	0.01581	0.00045	SS	0.05410	0.00386	0.00002	0.11113	0.01008	0.00002	0.36672	0.00630	0.21055
L100<br>B5/H12.5	0.36672	0.00630	0.21055	0.00004	SS	SS	0.01008	0.00232	0.00045	0.03663	0.00630	0.05410	0.69937	0.58062	0.58062

Al300 = aliskiren 300 mg; **Aml5** = amlodipine 5 mg; **B5** = bisoprolol 5 mg; **E20** = enalapril 20 mg; **H12.5** = hydrochlorothiazide 12.5 mg; **L100** = losartan 100 mg; **SS** = statistically significant (P < 0.00001)

Figure S16. Simulated change in ejection fraction from baseline to week 4 (mean ± SD, n = 100)

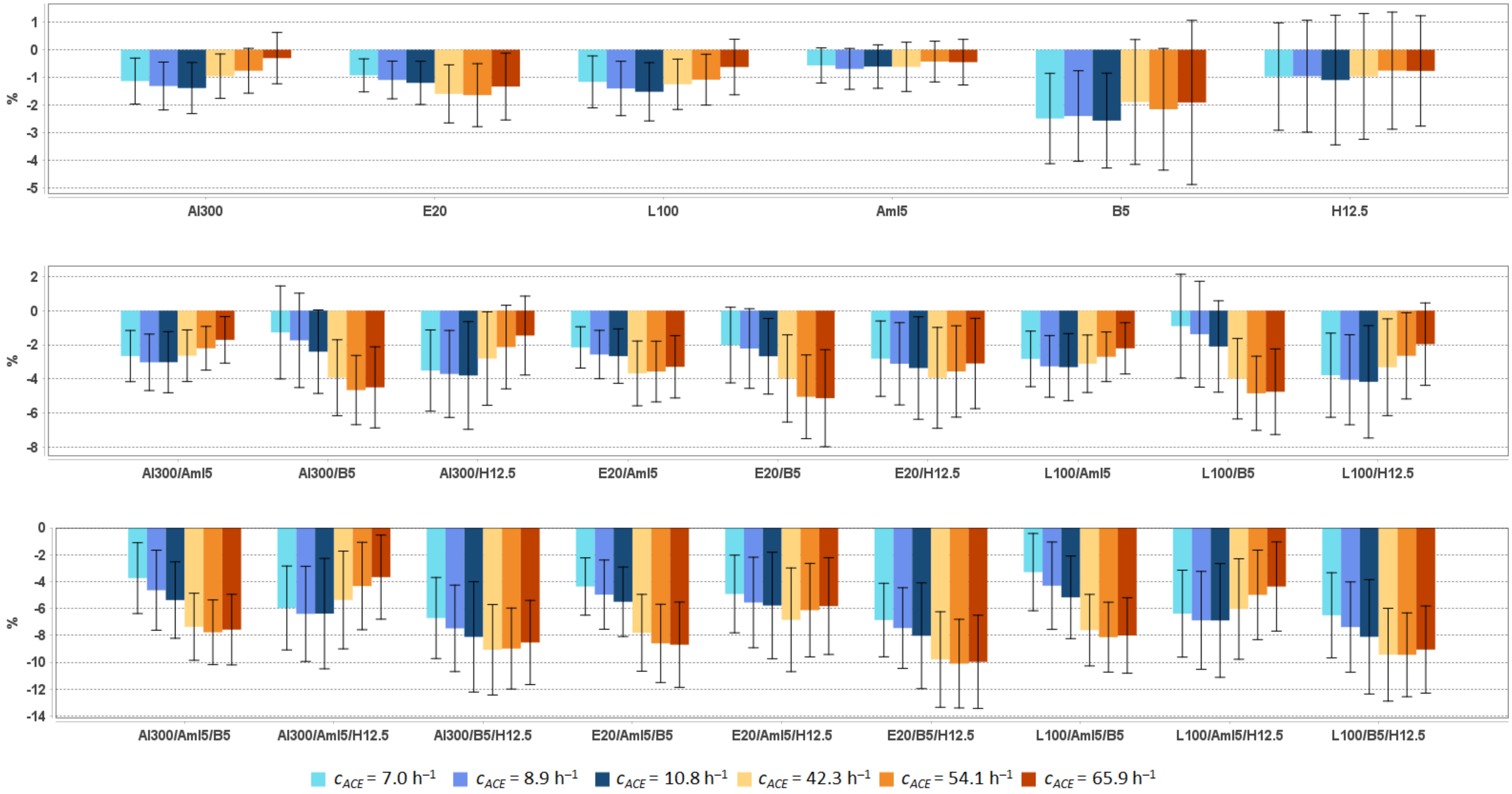

Al300 = aliskiren 300 mg; **Aml5** = amlodipine 5 mg; **B5** = bisoprolol 5 mg; **E20** = enalapril 20 mg; **H12.5** = hydrochlorothiazide 12.5 mg; **L100** = losartan 100 mg

Table S18. Simulated response of ejection fraction to antihypertensive therapy in virtual hypertensive subpopulations (n = 100) with different ACE activity, including P-values (Kolmogorov-Smirnov test) for endpoint *vs*. baseline; data are presented as mean ± SD in %

Regimens	***LII*** **(c_{ACE} = 7.0 h^{-1})**			***LID*** **(c_{ACE} = 8.9 h^{-1})**			***LDD*** **(c_{ACE} = 10.8 h^{-1})**			***HII*** **(c_{ACE} = 42.3 h^{-1})**			***HID*** **(c_{ACE} = 54.1 h^{-1})**			***HDD*** **(c_{ACE} = 65.9 h^{-1})**		
	**Value**	**Change**	***P***	**Value**	**Change**	***P***	**Value**	**Change**	***P***	**Value**	**Change**	***P***	**Value**	**Change**	***P***	**Value**	**Change**	***P***
Baseline	66.6 ± 5.6	–	–	66.4 ± 6.2	–	–	66.7 ± 6.5	–	–	66.3 ± 6.8	–	–	66.5 ± 6.2	–	–	65.5 ± 6.6	–	–
Al300	65.5 ± 5.7	-1.1 ± 0.8	0.28093	65.1 ± 6.2	-1.3 ± 0.9	0.46756	65.3 ± 6.5	-1.4 ± 0.9	0.28093	65.4 ± 6.8	-1.0 ± 0.8	0.90621	65.7 ± 6.3	-0.8 ± 0.8	0.90621	65.2 ± 6.6	-0.3 ± 0.9	0.99376
E20	65.7 ± 5.7	-0.9 ± 0.6	0.46756	65.3 ± 6.2	-1.1 ± 0.7	0.58062	65.5 ± 6.5	-1.2 ± 0.8	0.28093	64.7 ± 6.8	-1.6 ± 1.1	0.36672	64.8 ± 6.4	-1.6 ± 1.1	0.36672	64.2 ± 6.6	-1.3 ± 1.2	0.36672
L100	65.4 ± 5.8	-1.2 ± 0.9	0.28093	65.0 ± 6.2	-1.4 ± 1.0	0.28093	65.2 ± 6.5	-1.5 ± 1.1	0.21055	65.1 ± 6.8	-1.2 ± 0.9	0.69937	65.4 ± 6.4	-1.1 ± 0.9	0.81275	64.9 ± 6.6	-0.6 ± 1.0	0.90621
Aml5	66.0 ± 5.7	-0.6 ± 0.6	0.81275	65.7 ± 6.1	-0.7 ± 0.7	0.90621	66.1 ± 6.3	-0.6 ± 0.8	0.69937	65.7 ± 6.9	-0.6 ± 0.9	0.81275	66.0 ± 6.3	-0.4 ± 0.7	0.96707	65.1 ± 6.7	-0.4 ± 0.8	0.96707
B5	64.1 ± 5.4	-2.5 ± 1.6	0.05410	64.0 ± 6.2	-2.4 ± 1.6	0.02431	64.1 ± 6.5	-2.6 ± 1.7	0.07832	64.5 ± 6.8	-1.9 ± 2.3	0.28093	64.3 ± 5.9	-2.2 ± 2.2	0.21055	63.6 ± 6.4	-1.9 ± 3.0	0.28093
H12.5	65.6 ± 6.0	-1.0 ± 1.9	0.46756	65.4 ± 6.7	-1.0 ± 2.0	0.28093	65.6 ± 6.6	-1.1 ± 2.3	0.69937	65.4 ± 7.3	-1.0 ± 2.3	0.69937	65.7 ± 6.5	-0.8 ± 2.1	0.81275	64.8 ± 7.0	-0.8 ± 2.0	0.58062
Al300<br>Aml5	63.9 ± 6.0	-2.7 ± 1.5	0.00386	63.4 ± 6.3	-3.0 ± 1.7	0.00630	63.7 ± 6.5	-3.0 ± 1.8	0.00630	63.7 ± 6.9	-2.6 ± 1.5	0.05410	64.3 ± 6.5	-2.2 ± 1.3	0.15454	63.8 ± 6.7	-1.7 ± 1.4	0.15454
Al300<br>B5	65.3 ± 5.9	-1.3 ± 2.7	0.28093	64.6 ± 6.6	-1.7 ± 2.8	0.21055	64.3 ± 6.7	-2.4 ± 2.4	0.05410	62.4 ± 6.6	-3.9 ± 2.2	0.01581	61.8 ± 6.1	-4.6 ± 2.0	0.00025	61.0 ± 6.1	-4.5 ± 2.4	0.00045
Al300<br>H12.5	63.1 ± 6.2	-3.5 ± 2.4	0.00007	62.7 ± 6.9	-3.7 ± 2.5	0.00013	62.9 ± 6.8	-3.8 ± 3.1	0.01008	63.5 ± 7.3	-2.8 ± 2.7	0.05410	64.3 ± 6.7	-2.1 ± 2.4	0.21055	64.1 ± 7.0	-1.5 ± 2.3	0.11113
E20<br>Aml5	64.5 ± 5.8	-2.1 ± 1.2	0.02431	63.8 ± 6.3	-2.6 ± 1.4	0.02431	64.0 ± 6.4	-2.7 ± 1.6	0.01008	62.7 ± 7.0	-3.7 ± 1.9	0.00386	62.9 ± 6.7	-3.6 ± 1.8	0.00386	62.2 ± 6.7	-3.3 ± 1.8	0.01008
E20<br>B5	64.6 ± 5.7	-2.0 ± 2.2	0.21055	64.2 ± 6.4	-2.2 ± 2.3	0.05410	64.0 ± 6.6	-2.7 ± 2.2	0.03663	62.4 ± 6.7	-4.0 ± 2.6	0.01008	61.4 ± 6.3	-5.0 ± 2.4	0.00013	60.4 ± 6.3	-5.1 ± 2.8	0.00004
E20<br>H12.5	63.8 ± 6.1	-2.8 ± 2.2	0.00136	63.3 ± 6.8	-3.1 ± 2.4	0.00079	63.3 ± 6.8	-3.4 ± 3.0	0.01581	62.4 ± 7.4	-3.9 ± 3.0	0.00136	62.9 ± 6.9	-3.6 ± 2.7	0.00630	62.4 ± 7.1	-3.1 ± 2.6	0.00386
L100<br>Aml5	63.8 ± 6.0	-2.8 ± 1.6	0.00386	63.1 ± 6.4	-3.3 ± 1.8	0.00232	63.4 ± 6.5	-3.3 ± 2.0	0.00630	63.2 ± 6.9	-3.1 ± 1.7	0.02431	63.8 ± 6.6	-2.7 ± 1.5	0.05410	63.3 ± 6.7	-2.2 ± 1.5	0.11113
L100<br>B5	65.7 ± 6.1	-0.9 ± 3.0	0.69937	65.0 ± 6.8	-1.4 ± 3.1	0.36672	64.6 ± 6.8	-2.1 ± 2.7	0.07832	62.4 ± 6.7	-4.0 ± 2.4	0.01581	61.6 ± 6.2	-4.8 ± 2.2	0.00013	60.8 ± 6.2	-4.7 ± 2.5	0.00007
L100<br>H12.5	62.8 ± 6.3	-3.8 ± 2.5	0.00007	62.3 ± 6.9	-4.0 ± 2.6	0.00007	62.5 ± 6.9	-4.2 ± 3.3	0.00386	63.0 ± 7.4	-3.3 ± 2.8	0.01008	63.8 ± 6.8	-2.6 ± 2.5	0.05410	63.6 ± 7.0	-2.0 ± 2.4	0.05410
Al300<br>Aml5/B5	62.9 ± 6.1	-3.7 ± 2.6	0.00013	61.7 ± 6.5	-4.6 ± 3.0	SS	61.3 ± 6.6	-5.4 ± 2.8	0.00002	59.0 ± 6.6	-7.4 ± 2.5	SS	58.7 ± 6.5	-7.8 ± 2.4	SS	57.9 ± 6.3	-7.6 ± 2.6	SS
Al300<br>Aml5/H12.5	60.6 ± 6.7	-6.0 ± 3.1	SS	60.0 ± 7.3	-6.4 ± 3.5	SS	60.3 ± 7.1	-6.4 ± 4.1	SS	61.0 ± 7.8	-5.4 ± 3.6	0.00004	62.1 ± 7.2	-4.3 ± 3.2	0.00079	61.9 ± 7.4	-3.7 ± 3.1	0.00386
Al300<br>B5/H12.5	59.9 ± 6.3	-6.7 ± 3.0	SS	58.9 ± 6.8	-7.5 ± 3.2	SS	58.6 ± 6.9	-8.1 ± 4.1	SS	57.3 ± 7.1	-9.1 ± 3.4	SS	57.5 ± 6.6	-9.0 ± 3.0	SS	57.0 ± 6.6	-8.5 ± 3.1	SS
E20<br>Aml5/B5	62.2 ± 5.8	-4.4 ± 2.1	0.00007	61.4 ± 6.3	-5.0 ± 2.6	SS	61.2 ± 6.4	-5.5 ± 2.6	0.00002	58.5 ± 6.7	-7.8 ± 2.9	SS	57.9 ± 6.7	-8.6 ± 2.9	SS	56.8 ± 6.5	-8.7 ± 3.2	SS
E20<br>Aml5/H12.5	61.7 ± 6.5	-4.9 ± 2.9	SS	60.8 ± 7.2	-5.6 ± 3.4	SS	60.9 ± 7.0	-5.8 ± 4.0	0.00004	59.5 ± 7.9	-6.8 ± 3.9	SS	60.3 ± 7.4	-6.1 ± 3.5	SS	59.7 ± 7.7	-5.8 ± 3.6	SS
E20<br>B5/H12.5	59.7 ± 6.0	-6.9 ± 2.7	SS	58.9 ± 6.7	-7.5 ± 3.0	SS	58.7 ± 6.8	-8.0 ± 3.9	SS	56.6 ± 7.2	-9.8 ± 3.6	SS	56.4 ± 6.8	-10.1 ± 3.3	SS	55.6 ± 6.8	-10.0 ± 3.5	SS
L100<br>Aml5/B5	63.3 ± 6.2	-3.3 ± 2.9	0.00079	62.1 ± 6.6	-4.3 ± 3.2	SS	61.5 ± 6.7	-5.2 ± 3.1	0.00007	58.7 ± 6.7	-7.6 ± 2.7	SS	58.3 ± 6.6	-8.1 ± 2.6	SS	57.5 ± 6.3	-8.0 ± 2.8	SS
L100<br>Aml5/H12.5	60.2 ± 6.8	-6.4 ± 3.2	SS	59.5 ± 7.3	-6.9 ± 3.6	SS	59.8 ± 7.2	-6.9 ± 4.2	SS	60.3 ± 7.8	-6.0 ± 3.7	SS	61.5 ± 7.3	-5.0 ± 3.3	0.00013	61.2 ± 7.5	-4.4 ± 3.3	0.00045
L100<br>B5/H12.5	60.1 ± 6.4	-6.5 ± 3.2	SS	59.0 ± 6.9	-7.4 ± 3.4	SS	58.6 ± 7.0	-8.1 ± 4.3	SS	56.9 ± 7.1	-9.4 ± 3.4	SS	57.0 ± 6.7	-9.4 ± 3.1	SS	56.5 ± 6.7	-9.1 ± 3.2	SS

Al300 = aliskiren 300 mg; **Aml5** = amlodipine 5 mg; **B5** = bisoprolol 5 mg; **E20** = enalapril 20 mg; **H12.5** = hydrochlorothiazide 12.5 mg; **L100** = losartan 100 mg; **SS** = statistically significant (P < 0.00001)

Table S19. *P*-values calculated using the Kolmogorov-Smirnov test for changes in ejection fraction in subpopulations (n = 100) with different ACE activity receiving the same regimens. Case 1: c_{ACE} = 7.0 h^{-1} (*LII*), case 2: c_{ACE} = 8.9 h^{-1} (*LID*), case 3: c_{ACE} = 10.8 h^{-1} (*LDD*), case 4: c_{ACE} = 42.3 h^{-1} (*HII*), case 5: c_{ACE} = 54.1 h^{-1} (*HID*), case 6: c_{ACE} =65.9 h^{-1} (*HDD*). *P*-value for case *i vs*. case *j* is denoted P_{ij}.

Regimens	P_{12}	P_{13}	P_{23}	P_{14}	P_{15}	P_{16}	P_{24}	P_{25}	P_{26}	P_{34}	P_{35}	P_{36}	P_{45}	P_{46}	P_{56}
Al300	0.15454	0.05410	0.81275	0.28093	0.03663	SS	0.05410	0.00025	SS	0.07832	0.00004	SS	0.03663	0.00004	0.00630
E20	0.03663	0.01581	0.69937	SS	SS	0.00025	0.00045	0.00630	0.00232	0.00045	0.01008	0.03663	0.58062	0.21055	0.28093
L100	0.07832	0.03663	0.58062	0.15454	0.81275	0.00386	0.46756	0.02431	0.00045	0.28093	0.03663	0.00004	0.03663	0.00025	0.02431
Aml5	0.46756	0.69937	0.36672	0.58062	0.21055	0.07832	0.36672	0.07832	0.02431	0.99376	0.21055	0.21055	0.15454	0.28093	0.99376
B5	0.90621	0.96707	0.90621	0.15454	0.15454	0.01581	0.15454	0.28093	0.01581	0.03663	0.07832	0.00232	0.69937	0.36672	0.46756
H12.5	0.81275	0.90621	0.81275	0.21055	0.58062	0.69937	0.21055	0.03663	0.07832	0.69937	0.28093	0.28093	0.11113	0.36672	0.90621
Al300<br>Aml5	0.05410	0.11113	0.69937	0.90621	0.21055	0.00136	0.03663	0.00079	SS	0.15454	0.00630	0.00013	0.05410	0.00045	0.05410
Al300<br>B5	0.69937	0.03663	0.03663	SS	SS	SS	SS	SS	SS	0.00045	SS	SS	0.15454	0.28093	0.90621
Al300<br>H12.5	0.58062	0.58062	0.69937	0.07832	0.00079	SS	0.05410	0.00079	SS	0.21055	0.00045	SS	0.11113	0.00232	0.11113
E20<br>Aml5	0.03663	0.05410	0.81275	SS	SS	0.00002	0.00045	0.00630	0.02431	0.00007	0.00232	0.02431	0.58062	0.15454	0.46756
E20<br>B5	0.81275	0.05410	0.11113	SS	SS	SS	0.00002	SS	SS	0.00136	SS	SS	0.05410	0.01008	0.69937
E20<br>H12.5	0.36672	0.11113	0.46756	0.00232	0.03663	0.46756	0.03663	0.69937	0.90621	0.07832	0.36672	0.58062	0.21055	0.15454	0.46756
L100<br>Aml5	0.02431	0.07832	0.90621	0.28093	0.90621	0.03663	0.36672	0.01008	0.00045	0.69937	0.03663	0.00386	0.07832	0.00386	0.01581
L100<br>B5	0.58062	0.05410	0.03663	SS	SS	SS	SS	SS	SS	0.00004	SS	SS	0.07832	0.15454	0.90621
L100<br>H12.5	0.36672	0.46756	0.69937	0.21055	0.01008	SS	0.11113	0.00386	SS	0.58062	0.00386	0.00007	0.11113	0.00386	0.21055
Al300<br>Aml5/B5	0.01581	0.00025	0.28093	SS	SS	SS	SS	SS	SS	SS	SS	SS	0.36672	0.96707	0.69937
Al300<br>Aml5/H12.5	0.15454	0.69937	0.21055	0.28093	0.00136	0.00004	0.02431	0.00025	SS	0.28093	0.00386	SS	0.21055	0.01008	0.15454
Al300<br>B5/H12.5	0.02431	0.03663	0.28093	SS	SS	0.00007	0.01581	0.02431	0.15454	0.02431	0.01008	0.21055	0.69937	0.69937	0.69937
E20<br>Aml5/B5	0.03663	0.00079	0.21055	SS	SS	SS	SS	SS	SS	SS	SS	SS	0.21055	0.28093	0.58062
E20<br>Aml5/H12.5	0.15454	0.28093	0.90621	0.00079	0.00386	0.05410	0.05410	0.46756	0.69937	0.01581	0.11113	0.69937	0.36672	0.11113	0.69937
E20<br>B5/H12.5	0.05410	0.05410	0.28093	SS	SS	SS	0.00013	0.00004	SS	0.00079	0.00007	0.00025	0.69937	0.81275	0.58062
L100<br>Aml5/B5	0.05410	0.00045	0.28093	SS	SS	SS	SS	SS	SS	SS	SS	SS	0.28093	0.69937	0.69937
L100<br>Aml5/H12.5	0.15454	0.58062	0.36672	0.58062	0.01008	0.00013	0.07832	0.00045	0.00002	0.58062	0.01581	0.00013	0.15454	0.01008	0.21055
L100<br>B5/H12.5	0.01008	0.02431	0.15454	SS	SS	SS	0.00386	0.00232	0.01581	0.00136	0.00232	0.01008	0.46756	0.90621	0.58062

Al300 = aliskiren 300 mg; **Aml5** = amlodipine 5 mg; **B5** = bisoprolol 5 mg; **E20** = enalapril 20 mg; **H12.5** = hydrochlorothiazide 12.5 mg; **L100** = losartan 100 mg; **SS** = statistically significant (P < 0.00001)

Figure S17. Simulated change in <u>left ventricular end-diastolic pressure</u> from baseline to week 4 (mean ± SD, n = 100)

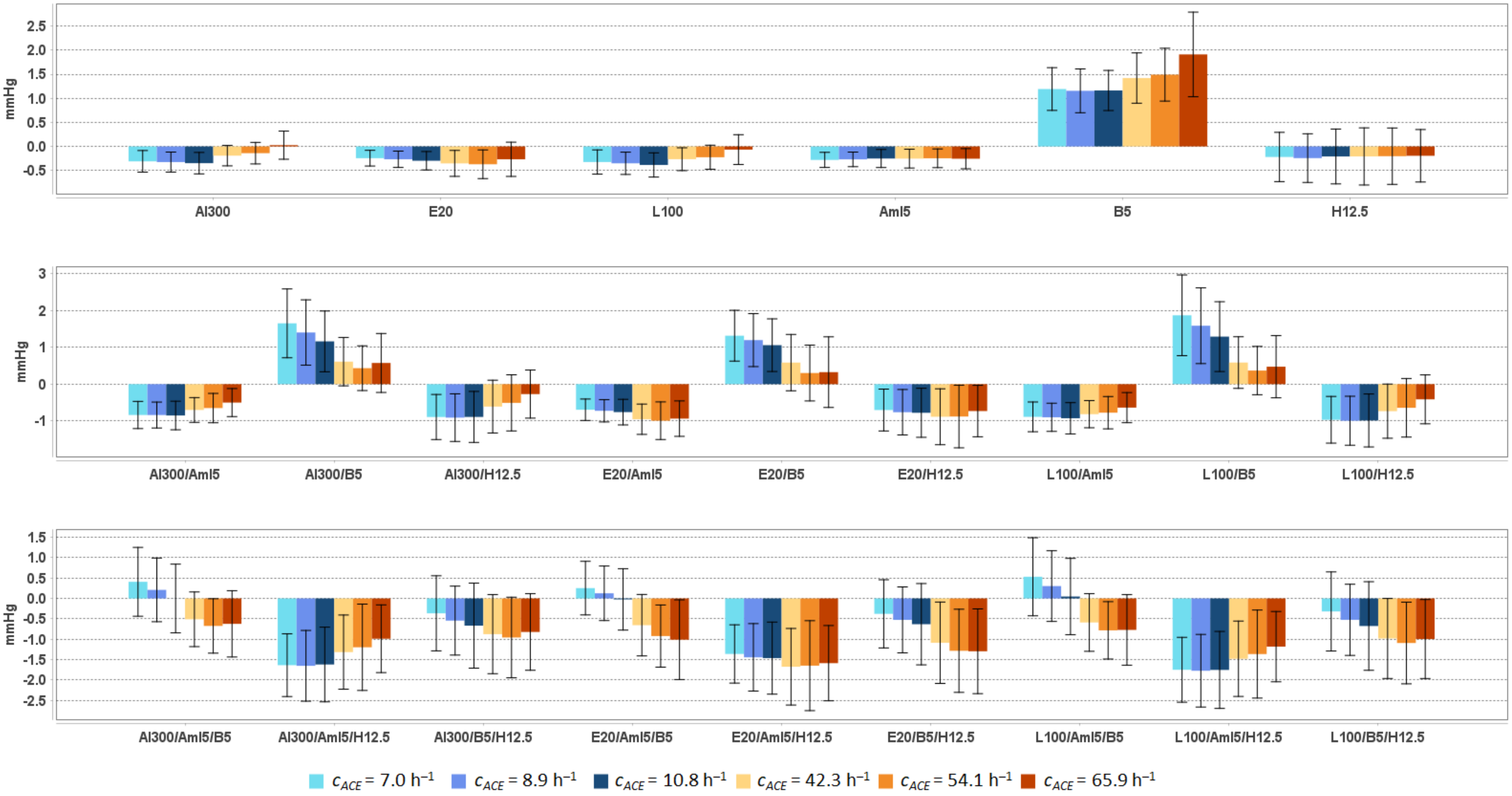

Al300 = aliskiren 300 mg; **Aml5** = amlodipine 5 mg; **B5** = bisoprolol 5 mg; **E20** = enalapril 20 mg; **H12.5** = hydrochlorothiazide 12.5 mg; **L100** = losartan 100 mg

Table S20. Simulated response of left ventricular end-diastolic pressure to antihypertensive therapy in virtual hypertensive subpopulations (n = 100) with different ACE activity, including P-values (Kolmogorov-Smirnov test) for endpoint *vs*. baseline; data are presented as mean ± SD in mmHg

Regimens	***LII* (c_{ACE} = 7.0 h^{-1})**			***LID* (c_{ACE} = 8.9 h^{-1})**			***LDD* (c_{ACE} = 10.8 h^{-1})**			***HII* (c_{ACE} = 42.3 h^{-1})**			***HID* (c_{ACE} = 54.1 h^{-1})**			***HDD* (c_{ACE} = 65.9 h^{-1})**		
	**Value**	**Change**	***P***	**Value**	**Change**	***P***	**Value**	**Change**	***P***	**Value**	**Change**	***P***	**Value**	**Change**	***P***	**Value**	**Change**	***P***
Baseline	8.1 ± 1.3	–	–	8.0 ± 1.2	–	–	8.1 ± 1.1	–	–	8.0 ± 1.0	–	–	8.0 ± 1.1	–	–	8.2 ± 1.0	–	–
Al300	7.7 ± 1.3	-0.3 ± 0.2	0.11113	7.7 ± 1.2	-0.3 ± 0.2	0.05410	7.7 ± 1.1	-0.3 ± 0.2	0.03663	7.8 ± 1.0	-0.2 ± 0.2	0.36672	7.9 ± 1.1	-0.1 ± 0.2	0.69937	8.2 ± 1.1	0.0 ± 0.3	0.36672
E20	7.8 ± 1.3	-0.2 ± 0.2	0.15454	7.7 ± 1.2	-0.3 ± 0.2	0.11113	7.8 ± 1.1	-0.3 ± 0.2	0.05410	7.7 ± 1.0	-0.4 ± 0.3	0.05410	7.6 ± 1.1	-0.4 ± 0.3	0.03663	7.9 ± 1.1	-0.3 ± 0.4	0.15454
L100	7.7 ± 1.3	-0.3 ± 0.3	0.05410	7.6 ± 1.2	-0.4 ± 0.2	0.05410	7.7 ± 1.1	-0.4 ± 0.3	0.01581	7.8 ± 1.0	-0.3 ± 0.2	0.15454	7.8 ± 1.1	-0.2 ± 0.2	0.21055	8.1 ± 1.1	-0.1 ± 0.3	0.90621
Aml5	7.8 ± 1.3	-0.3 ± 0.2	0.11113	7.7 ± 1.2	-0.3 ± 0.2	0.11113	7.8 ± 1.1	-0.3 ± 0.2	0.11113	7.8 ± 1.0	-0.3 ± 0.2	0.11113	7.7 ± 1.1	-0.2 ± 0.2	0.15454	8.0 ± 1.0	-0.3 ± 0.2	0.01581
B5	9.2 ± 1.6	1.2 ± 0.4	SS	9.1 ± 1.4	1.2 ± 0.5	SS	9.2 ± 1.3	1.2 ± 0.4	SS	9.5 ± 1.3	1.4 ± 0.5	SS	9.5 ± 1.5	1.5 ± 0.5	SS	10.1 ± 1.5	1.9 ± 0.9	SS
H12.5	7.8 ± 1.4	-0.2 ± 0.5	0.28093	7.7 ± 1.2	-0.2 ± 0.5	0.05410	7.8 ± 1.2	-0.2 ± 0.6	0.21055	7.8 ± 1.2	-0.2 ± 0.6	0.11113	7.8 ± 1.2	-0.2 ± 0.6	0.11113	8.0 ± 1.1	-0.2 ± 0.5	0.11113
Al300<br>Aml5	7.2 ± 1.2	-0.8 ± 0.4	SS	7.1 ± 1.1	-0.8 ± 0.4	SS	7.2 ± 1.0	-0.9 ± 0.4	SS	7.3 ± 0.9	-0.7 ± 0.3	SS	7.3 ± 1.1	-0.7 ± 0.4	0.00007	7.7 ± 1.0	-0.5 ± 0.4	0.00007
Al300<br>B5	9.7 ± 1.8	1.7 ± 0.9	SS	9.4 ± 1.7	1.4 ± 0.9	SS	9.2 ± 1.6	1.2 ± 0.8	SS	8.6 ± 1.3	0.6 ± 0.7	0.00013	8.4 ± 1.4	0.4 ± 0.6	0.07832	8.8 ± 1.4	0.6 ± 0.8	SS
Al300<br>H12.5	7.2 ± 1.3	-0.9 ± 0.6	SS	7.1 ± 1.1	-0.9 ± 0.6	SS	7.2 ± 1.1	-0.9 ± 0.7	SS	7.4 ± 1.2	-0.6 ± 0.7	0.00025	7.5 ± 1.2	-0.5 ± 0.8	0.00079	7.9 ± 1.2	-0.3 ± 0.7	0.07832
E20<br>Aml5	7.4 ± 1.2	-0.7 ± 0.3	SS	7.3 ± 1.1	-0.7 ± 0.3	SS	7.3 ± 1.0	-0.8 ± 0.3	SS	7.1 ± 0.9	-1.0 ± 0.4	SS	7.0 ± 1.1	-1.0 ± 0.5	SS	7.3 ± 1.0	-0.9 ± 0.5	SS
E20<br>B5	9.4 ± 1.7	1.3 ± 0.7	SS	9.2 ± 1.6	1.2 ± 0.7	SS	9.1 ± 1.5	1.1 ± 0.7	SS	8.6 ± 1.3	0.6 ± 0.8	0.00025	8.3 ± 1.4	0.3 ± 0.8	0.15454	8.5 ± 1.4	0.3 ± 1.0	0.00386
E20<br>H12.5	7.3 ± 1.3	-0.7 ± 0.6	0.00025	7.2 ± 1.1	-0.8 ± 0.6	SS	7.3 ± 1.2	-0.8 ± 0.7	SS	7.1 ± 1.2	-0.9 ± 0.8	SS	7.1 ± 1.2	-0.9 ± 0.9	SS	7.5 ± 1.2	-0.7 ± 0.7	0.00004
L100<br>Aml5	7.2 ± 1.2	-0.9 ± 0.4	SS	7.1 ± 1.1	-0.9 ± 0.4	SS	7.1 ± 1.0	-0.9 ± 0.4	SS	7.2 ± 0.9	-0.8 ± 0.4	SS	7.2 ± 1.1	-0.8 ± 0.4	SS	7.6 ± 1.0	-0.6 ± 0.4	0.00002
L100<br>B5	9.9 ± 1.9	1.9 ± 1.1	SS	9.6 ± 1.8	1.6 ± 1.0	SS	9.3 ± 1.7	1.3 ± 0.9	SS	8.6 ± 1.3	0.6 ± 0.7	0.00025	8.4 ± 1.4	0.4 ± 0.7	0.11113	8.7 ± 1.4	0.5 ± 0.8	0.00007
L100<br>H12.5	7.1 ± 1.3	-1.0 ± 0.6	SS	7.0 ± 1.1	-1.0 ± 0.7	SS	7.1 ± 1.1	-1.0 ± 0.7	SS	7.3 ± 1.2	-0.7 ± 0.7	0.00002	7.3 ± 1.2	-0.6 ± 0.8	0.00007	7.8 ± 1.2	-0.4 ± 0.7	0.03663
Al300<br>Aml5/B5	8.5 ± 1.6	0.4 ± 0.8	0.01581	8.2 ± 1.5	0.2 ± 0.8	0.21055	8.1 ± 1.4	-0.0 ± 0.8	0.46756	7.5 ± 1.1	-0.5 ± 0.7	0.00386	7.3 ± 1.2	-0.7 ± 0.7	0.00013	7.6 ± 1.2	-0.6 ± 0.8	0.00007
Al300<br>Aml5/H12.5	6.4 ± 1.2	-1.6 ± 0.8	SS	6.3 ± 1.1	-1.7 ± 0.9	SS	6.4 ± 1.2	-1.6 ± 0.9	SS	6.7 ± 1.2	-1.3 ± 0.9	SS	6.8 ± 1.3	-1.2 ± 1.1	SS	7.2 ± 1.2	-1.0 ± 0.8	SS
Al300<br>B5/H12.5	7.7 ± 1.5	-0.4 ± 0.9	0.07832	7.4 ± 1.3	-0.5 ± 0.8	0.00136	7.4 ± 1.4	-0.7 ± 1.0	0.00002	7.2 ± 1.3	-0.9 ± 1.0	SS	7.0 ± 1.3	-1.0 ± 1.0	SS	7.4 ± 1.3	-0.8 ± 0.9	SS
E20<br>Aml5/B5	8.3 ± 1.5	0.3 ± 0.7	0.11113	8.1 ± 1.4	0.1 ± 0.7	0.46756	8.0 ± 1.4	-0.0 ± 0.8	0.58062	7.4 ± 1.2	-0.7 ± 0.8	0.00013	7.1 ± 1.2	-0.9 ± 0.8	SS	7.2 ± 1.3	-1.0 ± 1.0	SS
E20<br>Aml5/H12.5	6.7 ± 1.2	-1.4 ± 0.7	SS	6.5 ± 1.1	-1.4 ± 0.8	SS	6.6 ± 1.2	-1.5 ± 0.9	SS	6.4 ± 1.2	-1.7 ± 0.9	SS	6.3 ± 1.3	-1.6 ± 1.1	SS	6.6 ± 1.2	-1.6 ± 0.9	SS
E20<br>B5/H12.5	7.7 ± 1.5	-0.4 ± 0.8	0.07832	7.5 ± 1.3	-0.5 ± 0.8	0.00232	7.4 ± 1.4	-0.6 ± 1.0	0.00013	6.9 ± 1.3	-1.1 ± 1.0	SS	6.7 ± 1.3	-1.3 ± 1.0	SS	6.9 ± 1.3	-1.3 ± 1.0	SS
L100<br>Aml5/B5	8.6 ± 1.7	0.5 ± 1.0	0.00232	8.3 ± 1.5	0.3 ± 0.9	0.07832	8.1 ± 1.5	0.0 ± 0.9	0.28093	7.4 ± 1.1	-0.6 ± 0.7	0.00079	7.2 ± 1.2	-0.8 ± 0.7	SS	7.4 ± 1.2	-0.8 ± 0.9	SS
L100<br>Aml5/H12.5	6.3 ± 1.2	-1.8 ± 0.8	SS	6.2 ± 1.1	-1.8 ± 0.9	SS	6.3 ± 1.2	-1.8 ± 0.9	SS	6.6 ± 1.2	-1.5 ± 0.9	SS	6.6 ± 1.3	-1.4 ± 1.1	SS	7.0 ± 1.2	-1.2 ± 0.9	SS
L100<br>B5/H12.5	7.7 ± 1.6	-0.3 ± 1.0	0.07832	7.5 ± 1.3	-0.5 ± 0.9	0.00232	7.4 ± 1.5	-0.7 ± 1.1	0.00004	7.1 ± 1.3	-1.0 ± 1.0	SS	6.9 ± 1.3	-1.1 ± 1.0	SS	7.2 ± 1.3	-1.0 ± 1.0	SS

Al300 = aliskiren 300 mg; **Aml5** = amlodipine 5 mg; **B5** = bisoprolol 5 mg; **E20** = enalapril 20 mg; **H12.5** = hydrochlorothiazide 12.5 mg; **L100** = losartan 100 mg; **SS** = statistically significant (P < 0.00001)

Table S21. *P*-values calculated using the Kolmogorov-Smirnov test for changes in left ventricular end-diastolic pressure in subpopulations (n = 100) with different ACE activity receiving the same regimens. Case 1: c_{ACE} = 7.0 h^{-1} (*LII*), case 2: c_{ACE} = 8.9 h^{-1} (*LID*), case 3: c_{ACE} = 10.8 h^{-1} (*LDD*), case 4: c_{ACE} = 42.3 h^{-1} (*HII*), case 5: c_{ACE} = 54.1 h^{-1} (*HID*), case 6: c_{ACE} = 65.9 h^{-1} (*HDD*). *P*-value for case *i* *vs*. case *j* is denoted P_{ij}.

**Regimens**	P_{12}	P_{13}	P_{23}	P_{14}	P_{15}	P_{16}	P_{24}	P_{25}	P_{26}	P_{34}	P_{35}	P_{36}	P_{45}	P_{46}	P_{56}
Al300	0.58062	0.15454	0.58062	0.01008	0.00004	SS	0.00232	SS	SS	0.00045	SS	SS	0.05410	0.00004	0.00136
E20	0.36672	0.02431	0.28093	0.00025	0.00025	0.00386	0.01008	0.00386	0.03663	0.05410	0.01008	0.02431	0.90621	0.15454	0.11113
L100	0.28093	0.11113	0.46756	0.28093	0.02431	SS	0.15454	0.00386	SS	0.02431	0.00025	SS	0.36672	0.00045	0.00630
Aml5	0.96707	0.46756	0.58062	0.69937	0.11113	0.28093	0.81275	0.15454	0.58062	0.81275	0.69937	0.90621	0.46756	0.90621	0.81275
B5	0.28093	0.58062	0.90621	0.01008	0.00079	SS	0.00232	0.00004	SS	0.01008	0.00045	SS	0.46756	0.00025	0.00136
H12.5	0.58062	0.81275	0.36672	0.69937	0.81275	0.90621	0.21055	0.15454	0.15454	0.81275	0.46756	0.46756	0.58062	0.81275	0.90621
Al300 Aml5	0.90621	0.96707	0.81275	0.07832	0.00004	SS	0.01581	SS	SS	0.05410	0.00002	SS	0.05410	0.00386	0.02431
Al300 B5	0.11113	0.00232	0.03663	SS	SS	SS	SS	SS	SS	0.00045	SS	SS	0.11113	0.28093	0.36672
Al300 H12.5	0.81275	0.58062	0.36672	0.01008	SS	SS	0.00232	SS	SS	0.07832	0.00013	SS	0.21055	0.00630	0.36672
E20 Aml5	0.58062	0.15454	0.69937	SS	0.00013	0.00004	0.00013	0.00386	0.00386	0.00045	0.01581	0.00386	0.58062	0.90621	0.90621
E20 B5	0.69937	0.01581	0.07832	SS	SS	SS	SS	SS	SS	0.00013	SS	SS	0.02431	0.05410	0.58062
E20 H12.5	0.90621	0.28093	0.36672	0.03663	0.21055	0.28093	0.07832	0.21055	0.46756	0.69937	0.69937	0.90621	0.46756	0.36672	0.90621
L100 Aml5	0.69937	0.81275	0.90621	0.90621	0.02431	0.00232	0.15454	0.00079	0.00007	0.21055	0.00136	0.00025	0.07832	0.01581	0.21055
L100 B5	0.07832	0.00079	0.05410	SS	SS	SS	SS	SS	SS	0.00007	SS	SS	0.03663	0.28093	0.36672
L100 H12.5	0.96707	0.36672	0.46756	0.02431	0.00004	SS	0.01008	0.00004	SS	0.11113	0.00045	SS	0.21055	0.02431	0.36672
Al300 Aml5/B5	0.07832	0.01581	0.21055	SS	SS	SS	SS	SS	SS	0.00079	SS	0.00002	0.15454	0.36672	0.58062
Al300 Aml5/H12.5	0.96707	0.46756	0.81275	0.00232	SS	SS	0.02431	0.00007	SS	0.03663	0.00045	0.00007	0.28093	0.02431	0.46756
Al300 B5/H12.5	0.21055	0.02431	0.21055	0.00079	0.00232	0.00136	0.07832	0.03663	0.03663	0.28093	0.03663	0.28093	0.81275	0.96707	0.69937
E20 Aml5/B5	0.21055	0.01008	0.36672	SS	SS	SS	SS	SS	SS	0.00002	SS	SS	0.11113	0.02431	0.46756
E20 Aml5/H12.5	0.96707	0.15454	0.58062	0.01008	0.05410	0.05410	0.05410	0.28093	0.15454	0.36672	0.69937	0.36672	0.81275	0.58062	0.69937
E20 B5/H12.5	0.46756	0.01581	0.15454	SS	SS	SS	0.00045	0.00004	SS	0.02431	0.00079	0.00079	0.58062	0.28093	0.58062
L100 Aml5/B5	0.05410	0.00232	0.21055	SS	SS	SS	SS	SS	SS	0.00007	SS	SS	0.15454	0.21055	0.58062
L100 Aml5/H12.5	0.96707	0.46756	0.81275	0.01581	0.00007	0.00004	0.05410	0.00013	0.00007	0.07832	0.00136	0.00079	0.28093	0.05410	0.90621
L100 B5/H12.5	0.11113	0.01581	0.21055	0.00002	0.00004	0.00007	0.01008	0.00630	0.00232	0.15454	0.01008	0.05410	0.69937	0.90621	0.81275

Al300 = aliskiren 300 mg; **Aml5** = amlodipine 5 mg; **B5** = bisoprolol 5 mg; **E20** = enalapril 20 mg; **H12.5** = hydrochlorothiazide 12.5 mg; **L100** = losartan 100 mg; **SS** = statistically significant ($P < 0.00001$)

Figure S18. Simulated change in left ventricular peak systolic pressure from baseline to week 4 (mean ± SD, n = 100)

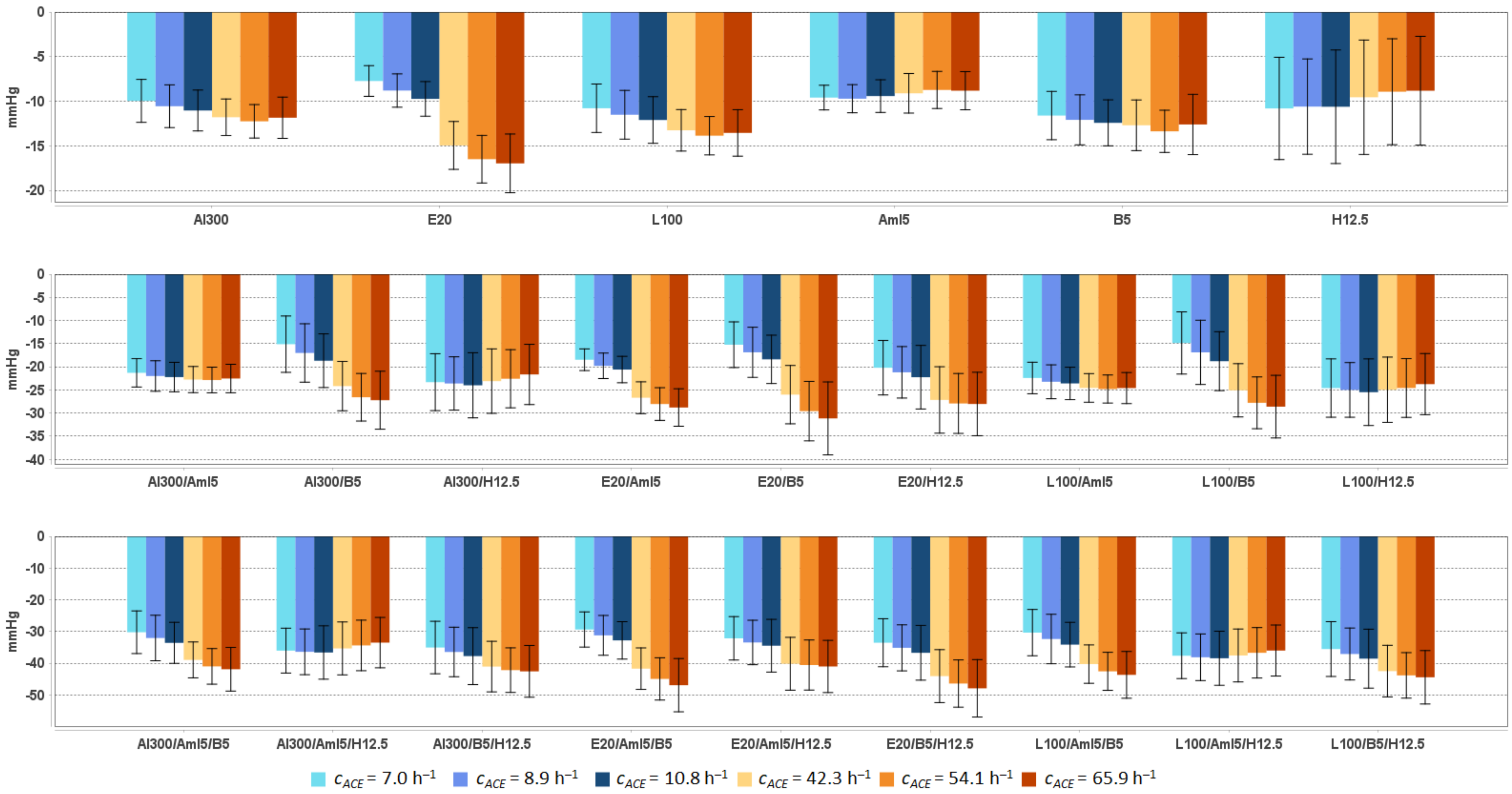

Al300 = aliskiren 300 mg; **Aml5** = amlodipine 5 mg; **B5** = bisoprolol 5 mg; **E20** = enalapril 20 mg; **H12.5** = hydrochlorothiazide 12.5 mg; **L100** = losartan 100 mg

Table S22. Simulated response of <u>left ventricular peak systolic pressure</u> to antihypertensive therapy in virtual hypertensive subpopulations (n = 100) with different ACE activity, including P-values (Kolmogorov-Smirnov test) for endpoint *vs*. baseline; data are presented as mean ± SD in mmHg

Regimens	***LII*** **(c_{ACE} = 7.0 h^{-1})**			***LID*** **(c_{ACE} = 8.9 h^{-1})**			***LDD*** **(c_{ACE} = 10.8 h^{-1})**			***HII*** **(c_{ACE} = 42.3 h^{-1})**			***HID*** **(c_{ACE} = 54.1 h^{-1})**			***HDD*** **(c_{ACE} = 65.9 h^{-1})**		
	**Value**	**Change**	***P***	**Value**	**Change**	***P***	**Value**	**Change**	***P***	**Value**	**Change**	***P***	**Value**	**Change**	***P***	**Value**	**Change**	***P***
Baseline	174.0 ± 6.6	–	–	174.3 ± 6.9	–	–	173.8 ± 6.4	–	–	172.8 ± 6.7	–	–	172.6 ± 6.8	–	–	173.4 ± 7.0	–	–
Al300	164.0 ± 6.6	-10.0 ± 2.4	SS	163.7 ± 7.3	-10.6 ± 2.4	SS	162.8 ± 6.9	-11.0 ± 2.3	SS	161.0 ± 7.1	-11.8 ± 2.0	SS	160.4 ± 7.1	-12.3 ± 1.9	SS	161.5 ± 7.3	-11.9 ± 2.3	SS
E20	166.3 ± 6.5	-7.7 ± 1.7	SS	165.5 ± 7.1	-8.8 ± 1.9	SS	164.1 ± 6.8	-9.7 ± 1.9	SS	157.9 ± 7.3	-15.0 ± 2.7	SS	156.1 ± 7.3	-16.5 ± 2.7	SS	156.4 ± 7.6	-17.0 ± 3.3	SS
L100	163.2 ± 6.7	-10.8 ± 2.7	SS	162.8 ± 7.4	-11.5 ± 2.7	SS	161.7 ± 7.0	-12.1 ± 2.6	SS	159.6 ± 7.2	-13.3 ± 2.3	SS	158.7 ± 7.1	-13.9 ± 2.2	SS	159.8 ± 7.4	-13.6 ± 2.6	SS
Aml5	164.4 ± 6.5	-9.6 ± 1.4	SS	164.6 ± 6.9	-9.7 ± 1.6	SS	164.4 ± 6.5	-9.4 ± 1.8	SS	163.7 ± 6.9	-9.1 ± 2.2	SS	163.9 ± 6.4	-8.7 ± 2.1	SS	164.6 ± 7.1	-8.8 ± 2.1	SS
B5	162.4 ± 6.5	-11.6 ± 2.7	SS	162.2 ± 7.2	-12.1 ± 2.8	SS	161.4 ± 6.7	-12.4 ± 2.6	SS	160.1 ± 7.4	-12.7 ± 2.8	SS	159.2 ± 7.0	-13.4 ± 2.4	SS	160.8 ± 7.6	-12.6 ± 3.4	SS
H12.5	163.2 ± 8.2	-10.8 ± 5.7	SS	163.7 ± 8.8	-10.6 ± 5.3	SS	163.2 ± 8.8	-10.6 ± 6.4	SS	163.3 ± 9.3	-9.6 ± 6.4	SS	163.7 ± 8.3	-8.9 ± 5.9	SS	164.6 ± 8.1	-8.8 ± 6.1	SS
Al300<br>Aml5	152.7 ± 6.8	-21.3 ± 3.1	SS	152.3 ± 7.7	-22.0 ± 3.3	SS	151.6 ± 7.4	-22.2 ± 3.2	SS	150.1 ± 7.4	-22.8 ± 2.8	SS	149.7 ± 7.0	-22.9 ± 2.8	SS	150.8 ± 7.6	-22.5 ± 3.1	SS
Al300<br>B5	158.9 ± 8.9	-15.1 ± 6.1	SS	157.3 ± 9.6	-17.0 ± 6.3	SS	155.1 ± 8.8	-18.7 ± 5.8	SS	148.7 ± 8.7	-24.2 ± 5.3	SS	146.0 ± 8.4	-26.6 ± 5.1	SS	146.2 ± 9.2	-27.2 ± 6.3	SS
Al300<br>H12.5	150.7 ± 8.2	-23.3 ± 6.1	SS	150.7 ± 9.1	-23.6 ± 5.8	SS	149.8 ± 9.5	-24.0 ± 7.0	SS	149.7 ± 9.9	-23.1 ± 7.0	SS	150.0 ± 8.7	-22.6 ± 6.3	SS	151.7 ± 8.6	-21.7 ± 6.5	SS
E20<br>Aml5	155.5 ± 6.6	-18.5 ± 2.3	SS	154.5 ± 7.4	-19.8 ± 2.8	SS	153.2 ± 7.2	-20.6 ± 2.9	SS	146.1 ± 7.6	-26.7 ± 3.4	SS	144.6 ± 7.4	-28.1 ± 3.5	SS	144.6 ± 8.0	-28.8 ± 4.1	SS
E20<br>B5	158.8 ± 7.9	-15.2 ± 4.9	SS	157.4 ± 8.8	-16.9 ± 5.4	SS	155.4 ± 8.4	-18.4 ± 5.2	SS	146.8 ± 9.5	-26.0 ± 6.3	SS	143.0 ± 9.3	-29.6 ± 6.4	SS	142.2 ± 10.4	-31.2 ± 7.9	SS
E20<br>H12.5	153.8 ± 8.1	-20.2 ± 5.9	SS	153.1 ± 9.0	-21.2 ± 5.6	SS	151.5 ± 9.3	-22.3 ± 6.9	SS	145.7 ± 10.0	-27.2 ± 7.2	SS	144.7 ± 8.8	-27.9 ± 6.5	SS	145.3 ± 8.8	-28.0 ± 6.9	SS
L100<br>Aml5	151.6 ± 7.0	-22.4 ± 3.4	SS	151.1 ± 7.9	-23.2 ± 3.7	SS	150.2 ± 7.6	-23.6 ± 3.5	SS	148.3 ± 7.5	-24.6 ± 3.1	SS	147.8 ± 7.1	-24.8 ± 3.0	SS	148.8 ± 7.7	-24.6 ± 3.4	SS
L100<br>B5	159.1 ± 9.4	-14.9 ± 6.7	SS	157.4 ± 10.1	-16.9 ± 6.9	SS	155.0 ± 9.3	-18.8 ± 6.4	SS	147.8 ± 9.0	-25.1 ± 5.8	SS	144.8 ± 8.7	-27.8 ± 5.6	SS	144.8 ± 9.6	-28.6 ± 6.8	SS
L100<br>H12.5	149.4 ± 8.3	-24.6 ± 6.3	SS	149.3 ± 9.2	-25.0 ± 5.9	SS	148.3 ± 9.6	-25.5 ± 7.2	SS	147.9 ± 9.9	-25.0 ± 7.1	SS	148.0 ± 8.7	-24.6 ± 6.3	SS	149.6 ± 8.6	-23.7 ± 6.6	SS
Al300<br>Aml5/B5	143.8 ± 9.3	-30.2 ± 6.7	SS	142.2 ± 10.4	-32.1 ± 7.2	SS	140.2 ± 9.5	-33.6 ± 6.5	SS	133.9 ± 8.8	-39.0 ± 5.7	SS	131.6 ± 8.5	-41.0 ± 5.6	SS	131.5 ± 9.6	-41.9 ± 6.9	SS
Al300<br>Aml5/H12.5	138.0 ± 8.8	-36.0 ± 7.1	SS	137.9 ± 10.2	-36.4 ± 7.2	SS	137.2 ± 10.7	-36.6 ± 8.4	SS	137.5 ± 10.8	-35.4 ± 8.3	SS	138.2 ± 9.7	-34.4 ± 8.0	SS	139.9 ± 9.7	-33.5 ± 7.9	SS
Al300<br>B5/H12.5	138.9 ± 9.6	-35.1 ± 8.3	SS	137.8 ± 10.6	-36.5 ± 7.8	SS	136.0 ± 11.0	-37.8 ± 9.0	SS	131.7 ± 10.2	-41.1 ± 8.0	SS	130.4 ± 8.8	-42.2 ± 7.0	SS	130.8 ± 9.6	-42.6 ± 8.2	SS
E20<br>Aml5/B5	144.6 ± 8.3	-29.4 ± 5.6	SS	143.1 ± 9.6	-31.2 ± 6.3	SS	141.0 ± 9.1	-32.8 ± 5.9	SS	131.1 ± 9.4	-41.7 ± 6.5	SS	127.6 ± 9.2	-45.0 ± 6.7	SS	126.4 ± 10.7	-46.9 ± 8.4	SS
E20<br>Aml5/H12.5	141.8 ± 8.7	-32.2 ± 6.8	SS	140.8 ± 10.1	-33.5 ± 7.0	SS	139.3 ± 10.6	-34.5 ± 8.3	SS	132.6 ± 10.6	-40.2 ± 8.3	SS	132.0 ± 9.6	-40.6 ± 7.9	SS	132.3 ± 9.8	-41.0 ± 8.2	SS
E20<br>B5/H12.5	140.4 ± 9.0	-33.6 ± 7.6	SS	139.1 ± 10.1	-35.2 ± 7.3	SS	137.1 ± 10.6	-36.7 ± 8.6	SS	128.7 ± 10.5	-44.1 ± 8.4	SS	126.2 ± 9.1	-46.4 ± 7.5	SS	125.4 ± 10.3	-47.9 ± 9.1	SS
L100<br>Aml5/B5	143.6 ± 9.8	-30.4 ± 7.3	SS	141.9 ± 10.9	-32.4 ± 7.8	SS	139.6 ± 10.0	-34.2 ± 7.0	SS	132.5 ± 9.1	-40.3 ± 6.1	SS	130.0 ± 8.7	-42.6 ± 6.0	SS	129.7 ± 9.9	-43.7 ± 7.4	SS
L100<br>Aml5/H12.5	136.3 ± 8.8	-37.7 ± 7.2	SS	136.1 ± 10.3	-38.1 ± 7.4	SS	135.3 ± 10.8	-38.5 ± 8.5	SS	135.3 ± 10.7	-37.6 ± 8.3	SS	135.9 ± 9.7	-36.7 ± 8.0	SS	137.4 ± 9.7	-36.0 ± 8.0	SS
L100<br>B5/H12.5	138.5 ± 10.0	-35.6 ± 8.6	SS	137.2 ± 10.9	-37.1 ± 8.2	SS	135.2 ± 11.3	-38.6 ± 9.3	SS	130.3 ± 10.3	-42.5 ± 8.1	SS	128.7 ± 8.9	-43.9 ± 7.2	SS	128.9 ± 9.8	-44.5 ± 8.4	SS

Al300 = aliskiren 300 mg; **Aml5** = amlodipine 5 mg; **B5** = bisoprolol 5 mg; **E20** = enalapril 20 mg; **H12.5** = hydrochlorothiazide 12.5 mg; **L100** = losartan 100 mg; **SS** = statistically significant (P < 0.00001)

Table S23. *P*-values calculated using the Kolmogorov-Smirnov test for changes in <u>left ventricular peak systolic pressure</u> in subpopulations (n = 100) with different ACE activity receiving the same regimens. Case 1: c_{ACE} = 7.0 h^{-1} (*LII*), case 2: c_{ACE} = 8.9 h^{-1} (*LID*), case 3: c_{ACE} = 10.8 h^{-1} (*LDD*), case 4: c_{ACE} = 42.3 h^{-1} (*HII*), case 5: c_{ACE} = 54.1 h^{-1} (*HID*), case 6: c_{ACE} = 65.9 h^{-1} (*HDD*). *P*-value for case *i* *vs*. case *j* is denoted P_{ij}.

**Regimens**	P_{12}	P_{13}	P_{23}	P_{14}	P_{15}	P_{16}	P_{24}	P_{25}	P_{26}	P_{34}	P_{35}	P_{36}	P_{45}	P_{46}	P_{56}
Al300	0.15454	0.01581	0.36672	SS	SS	SS	0.00079	SS	0.00045	0.05410	0.00079	0.02431	0.21055	0.69937	0.28093
E20	0.00079	SS	0.01581	SS	SS	SS	SS	SS	SS	SS	SS	SS	0.00079	0.00004	0.03663
L100	0.07832	0.00630	0.36672	SS	SS	SS	0.00007	SS	SS	0.01008	0.00002	0.00136	0.11113	0.46756	0.36672
Aml5	0.58062	0.69937	0.69937	0.15454	0.00045	0.01008	0.07832	0.00002	0.00045	0.36672	0.00045	0.01008	0.11113	0.21055	0.81275
B5	0.28093	0.11113	0.11113	0.03663	0.00045	0.00386	0.03663	0.02431	0.01581	0.69937	0.05410	0.46756	0.21055	0.46756	0.02431
H12.5	0.69937	0.69937	0.46756	0.46756	0.15454	0.07832	0.21055	0.01581	0.00232	0.28093	0.05410	0.02431	0.28093	0.36672	0.96707
Al300 Aml5	0.03663	0.00630	0.69937	0.00045	0.00079	0.00232	0.07832	0.36672	0.11113	0.36672	0.36672	0.69937	0.81275	0.90621	0.96707
Al300 B5	0.05410	0.00013	0.15454	SS	SS	SS	SS	SS	SS	SS	SS	SS	0.02431	0.00630	0.46756
Al300 H12.5	0.81275	0.15454	0.46756	0.81275	0.69937	0.11113	0.46756	0.15454	0.02431	0.58062	0.03663	0.01008	0.46756	0.28093	0.58062
E20 Aml5	0.00007	SS	0.02431	SS	SS	SS	SS	SS	SS	SS	SS	SS	0.07832	0.00079	0.15454
E20 B5	0.07832	SS	0.07832	SS	SS	SS	SS	SS	SS	SS	SS	SS	0.00386	0.00007	0.15454
E20 H12.5	0.36672	0.00386	0.07832	SS	SS	SS	SS	SS	SS	0.00002	SS	SS	0.81275	0.46756	0.90621
L100 Aml5	0.03663	0.00136	0.36672	SS	SS	0.00002	0.01581	0.00386	0.00386	0.07832	0.03663	0.11113	0.90621	0.81275	0.81275
L100 B5	0.05410	0.00007	0.11113	SS	SS	SS	SS	SS	SS	SS	SS	SS	0.02431	0.00232	0.36672
L100 H12.5	0.58062	0.11113	0.15454	0.28093	0.96707	0.81275	0.36672	0.28093	0.15454	0.81275	0.07832	0.07832	0.36672	0.46756	0.81275
Al300 Aml5/B5	0.05410	0.00136	0.28093	SS	SS	SS	SS	SS	SS	SS	SS	SS	0.07832	0.01581	0.28093
Al300 Aml5/H12.5	0.58062	0.58062	0.46756	0.46756	0.15454	0.11113	0.36672	0.03663	0.03663	0.21055	0.07832	0.03663	0.69937	0.15454	0.36672
Al300 B5/H12.5	0.21055	0.03663	0.28093	SS	SS	SS	0.00386	0.00007	0.00002	0.01581	0.00232	0.00630	0.58062	0.21055	0.81275
E20 Aml5/B5	0.00630	0.00007	0.15454	SS	SS	SS	SS	SS	SS	SS	SS	SS	0.00386	0.00025	0.07832
E20 Aml5/H12.5	0.21055	0.01008	0.21055	SS	SS	SS	SS	SS	SS	0.00045	0.00002	SS	0.46756	0.28093	0.36672
E20 B5/H12.5	0.15454	0.01008	0.21055	SS	SS	SS	SS	SS	SS	SS	SS	SS	0.15454	0.00386	0.21055
L100 Aml5/B5	0.05410	0.00136	0.28093	SS	SS	SS	SS	SS	SS	SS	SS	SS	0.03663	0.00630	0.28093
L100 Aml5/H12.5	0.46756	0.46756	0.36672	0.58062	0.28093	0.36672	0.36672	0.05410	0.11113	0.36672	0.15454	0.05410	0.69937	0.21055	0.46756
L100 B5/H12.5	0.21055	0.01581	0.15454	SS	SS	SS	0.00025	SS	SS	0.01008	0.00007	0.00136	0.46756	0.07832	0.69937

Al300 = aliskiren 300 mg; **Aml5** = amlodipine 5 mg; **B5** = bisoprolol 5 mg; **E20** = enalapril 20 mg; **H12.5** = hydrochlorothiazide 12.5 mg; **L100** = losartan 100 mg; **SS** = statistically significant (P < 0.00001)

Figure S19. Simulated change in left ventricular end-diastolic volume from baseline to week 4 (mean ± SD, n = 100)

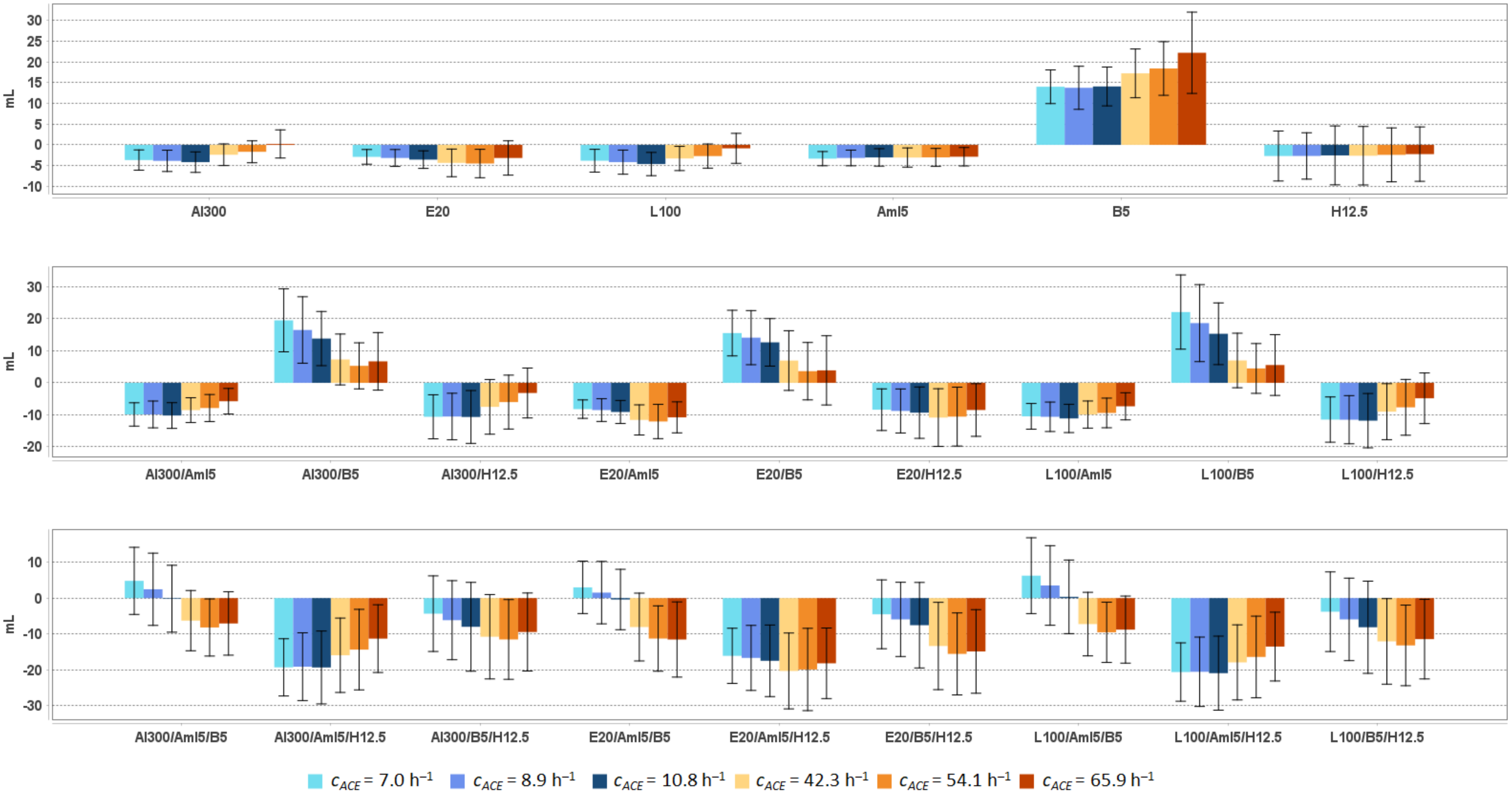

Al300 = aliskiren 300 mg; **Aml5** = amlodipine 5 mg; **B5** = bisoprolol 5 mg; **E20** = enalapril 20 mg; **H12.5** = hydrochlorothiazide 12.5 mg; **L100** = losartan 100 mg

Table S24. Simulated response of left ventricular end-diastolic volume to antihypertensive therapy in virtual hypertensive subpopulations (n = 100) with different ACE activity, including P-values (Kolmogorov-Smirnov test) for endpoint *vs*. baseline; data are presented as mean ± SD in mL

Regimens	***LII*** (c_{ACE} = 7.0 h^{-1})			***LID*** (c_{ACE} = 8.9 h^{-1})			***LDD*** (c_{ACE} = 10.8 h^{-1})			***HII*** (c_{ACE} = 42.3 h^{-1})			***HID*** (c_{ACE} = 54.1 h^{-1})			***HDD*** (c_{ACE} = 65.9 h^{-1})		
	**Value**	**Change**	***P***	**Value**	**Change**	***P***	**Value**	**Change**	***P***	**Value**	**Change**	***P***	**Value**	**Change**	***P***	**Value**	**Change**	***P***
Baseline	110.6 ± 12.8	–	–	110.0 ± 14.2	–	–	111.0 ± 14.0	–	–	110.5 ± 13.3	–	–	111.2 ± 13.5	–	–	110.5 ± 13.4	–	–
Al300	106.9 ± 12.6	-3.7 ± 2.4	0.15454	106.2 ± 14.1	-3.9 ± 2.6	0.15454	106.8 ± 13.8	-4.2 ± 2.5	0.01008	108.1 ± 13.1	-2.4 ± 2.6	0.15454	109.5 ± 13.7	-1.7 ± 2.6	0.58062	110.7 ± 13.6	0.2 ± 3.4	0.96707
E20	107.7 ± 12.6	-2.9 ± 1.8	0.28093	106.9 ± 14.1	-3.2 ± 2.0	0.36672	107.4 ± 13.8	-3.6 ± 2.1	0.03663	106.1 ± 13.0	-4.3 ± 3.3	0.01008	106.7 ± 13.5	-4.5 ± 3.4	0.01581	107.3 ± 13.6	-3.2 ± 4.1	0.11113
L100	106.7 ± 12.6	-3.8 ± 2.8	0.11113	105.8 ± 14.1	-4.2 ± 2.9	0.11113	106.3 ± 13.8	-4.6 ± 2.8	0.00630	107.2 ± 13.1	-3.3 ± 2.9	0.03663	108.5 ± 13.6	-2.7 ± 2.9	0.21055	109.6 ± 13.6	-0.8 ± 3.6	0.90621
Aml5	107.2 ± 12.6	-3.3 ± 1.7	0.03663	106.9 ± 13.9	-3.2 ± 1.9	0.36672	107.9 ± 13.8	-3.0 ± 2.1	0.05410	107.4 ± 13.2	-3.1 ± 2.3	0.11113	108.1 ± 13.5	-3.0 ± 2.2	0.02431	107.6 ± 13.6	-2.9 ± 2.2	0.15454
B5	124.6 ± 14.3	14.0 ± 4.1	SS	123.8 ± 16.9	13.8 ± 5.2	SS	125.1 ± 16.3	14.1 ± 4.7	SS	127.8 ± 15.8	17.3 ± 5.9	SS	129.6 ± 17.2	18.4 ± 6.5	SS	132.7 ± 18.2	22.2 ± 9.8	SS
H12.5	107.9 ± 14.3	-2.7 ± 6.0	0.15454	107.3 ± 14.5	-2.7 ± 5.6	0.58062	108.4 ± 15.9	-2.5 ± 7.1	0.07832	107.9 ± 14.3	-2.6 ± 7.1	0.28093	108.7 ± 15.6	-2.4 ± 6.5	0.21055	108.2 ± 14.3	-2.2 ± 6.6	0.46756
Al300<br>Aml5	100.7 ± 12.4	-9.9 ± 3.6	0.00004	100.1 ± 13.9	-9.9 ± 4.2	0.00025	100.8 ± 13.5	-10.2 ± 4.0	SS	102.0 ± 13.0	-8.5 ± 3.9	0.00007	103.3 ± 13.7	-7.9 ± 4.2	0.00045	104.7 ± 13.9	-5.7 ± 4.0	0.00630
Al300<br>B5	130.1 ± 16.6	19.5 ± 9.8	SS	126.5 ± 19.6	16.5 ± 10.4	SS	124.8 ± 17.1	13.8 ± 8.5	SS	117.8 ± 15.1	7.3 ± 7.9	0.00004	116.5 ± 15.4	5.3 ± 7.2	0.00630	117.2 ± 16.2	6.7 ± 9.0	0.00045
Al300<br>H12.5	100.0 ± 14.1	-10.6 ± 6.9	0.00002	99.5 ± 14.6	-10.5 ± 7.3	0.00013	100.3 ± 15.9	-10.7 ± 8.2	SS	103.0 ± 14.7	-7.5 ± 8.5	0.00232	105.1 ± 16.6	-6.0 ± 8.4	0.01008	107.3 ± 14.7	-3.2 ± 7.8	0.28093
E20<br>Aml5	102.4 ± 12.4	-8.2 ± 2.9	0.00025	101.5 ± 13.8	-8.5 ± 3.5	0.00232	101.9 ± 13.5	-9.1 ± 3.6	SS	98.9 ± 13.0	-11.6 ± 4.7	SS	99.1 ± 13.6	-12.1 ± 5.4	SS	99.7 ± 14.0	-10.8 ± 4.8	SS
E20<br>B5	126.1 ± 15.2	15.5 ± 7.1	SS	124.1 ± 18.3	14.1 ± 8.4	SS	123.6 ± 16.6	12.6 ± 7.4	SS	117.4 ± 15.6	6.9 ± 9.3	0.00007	114.8 ± 15.9	3.6 ± 9.0	0.03663	114.4 ± 17.2	3.9 ± 10.8	0.03663
E20<br>H12.5	102.2 ± 14.1	-8.4 ± 6.5	0.00079	101.2 ± 14.5	-8.8 ± 6.9	0.00136	101.6 ± 15.9	-9.3 ± 8.0	0.00013	99.6 ± 14.7	-10.8 ± 9.0	0.00004	100.6 ± 16.6	-10.6 ± 9.2	0.00013	102.0 ± 14.8	-8.5 ± 8.2	0.00045
L100<br>Aml5	100.1 ± 12.4	-10.5 ± 4.0	0.00002	99.4 ± 14.0	-10.6 ± 4.6	0.00013	99.8 ± 13.6	-11.1 ± 4.4	SS	100.6 ± 13.0	-9.9 ± 4.2	SS	101.8 ± 13.7	-9.4 ± 4.6	0.00004	103.1 ± 13.9	-7.3 ± 4.2	0.00079
L100<br>B5	132.7 ± 17.8	22.1 ± 11.6	SS	128.7 ± 20.8	18.6 ± 12.0	SS	126.3 ± 17.6	15.3 ± 9.6	SS	117.5 ± 15.3	7.0 ± 8.5	0.00004	115.6 ± 15.5	4.5 ± 7.8	0.01581	116.0 ± 16.4	5.6 ± 9.5	0.00630
L100<br>H12.5	99.1 ± 14.1	-11.5 ± 7.1	SS	98.5 ± 14.6	-11.5 ± 7.5	0.00002	99.1 ± 16.0	-11.8 ± 8.5	SS	101.5 ± 14.7	-9.0 ± 8.7	0.00025	103.5 ± 16.6	-7.7 ± 8.7	0.00386	105.7 ± 14.7	-4.8 ± 7.9	0.11113
Al300<br>Aml5/B5	115.4 ± 15.2	4.8 ± 9.3	0.00045	112.5 ± 17.9	2.5 ± 10.1	0.36672	110.8 ± 16.1	-0.2 ± 9.3	0.28093	104.2 ± 14.7	-6.3 ± 8.4	0.01581	103.0 ± 14.8	-8.2 ± 8.0	0.00045	103.4 ± 15.9	-7.1 ± 8.8	0.00232
Al300<br>Aml5/H12.5	91.3 ± 14.5	-19.3 ± 8.0	SS	90.9 ± 15.4	-19.1 ± 9.4	SS	91.6 ± 16.7	-19.3 ± 10.1	SS	94.6 ± 15.9	-15.9 ± 10.4	SS	96.8 ± 17.8	-14.3 ± 11.2	SS	99.2 ± 15.9	-11.3 ± 9.4	SS
Al300<br>B5/H12.5	106.2 ± 16.3	-4.3 ± 10.5	0.05410	103.9 ± 17.2	-6.1 ± 11.0	0.02431	103.0 ± 18.5	-8.0 ± 12.4	0.00079	99.7 ± 16.4	-10.8 ± 11.7	0.00004	99.6 ± 17.3	-11.5 ± 11.1	SS	101.0 ± 16.3	-9.4 ± 10.9	0.00079
E20<br>Aml5/B5	113.6 ± 14.3	3.0 ± 7.3	0.00079	111.5 ± 17.1	1.5 ± 8.7	0.36672	110.6 ± 15.8	-0.4 ± 8.4	0.36672	102.4 ± 15.0	-8.1 ± 9.4	0.00232	99.9 ± 15.1	-11.3 ± 9.1	SS	99.0 ± 16.7	-11.5 ± 10.5	SS
E20<br>Aml5/H12.5	94.5 ± 14.5	-16.1 ± 7.7	SS	93.4 ± 15.3	-16.7 ± 9.1	SS	93.5 ± 16.6	-17.5 ± 10.0	SS	90.2 ± 15.9	-20.3 ± 10.6	SS	91.3 ± 17.5	-19.9 ± 11.5	SS	92.3 ± 16.0	-18.2 ± 9.8	SS
E20<br>B5/H12.5	106.1 ± 15.8	-4.5 ± 9.6	0.07832	104.1 ± 16.8	-5.9 ± 10.3	0.05410	103.4 ± 18.2	-7.5 ± 11.9	0.00079	97.2 ± 16.5	-13.3 ± 12.2	SS	95.6 ± 17.1	-15.5 ± 11.4	SS	95.6 ± 16.7	-14.8 ± 11.6	SS
L100<br>Aml5/B5	116.8 ± 15.9	6.2 ± 10.6	0.00045	113.5 ± 18.6	3.5 ± 11.1	0.28093	111.3 ± 16.6	0.3 ± 10.2	0.21055	103.3 ± 14.8	-7.2 ± 8.8	0.00630	101.6 ± 14.9	-9.5 ± 8.4	0.00004	101.7 ± 16.2	-8.8 ± 9.3	0.00013
L100<br>Aml5/H12.5	90.0 ± 14.6	-20.6 ± 8.1	SS	89.5 ± 15.5	-20.5 ± 9.7	SS	90.1 ± 16.7	-20.9 ± 10.3	SS	92.6 ± 15.9	-17.9 ± 10.5	SS	94.8 ± 17.7	-16.4 ± 11.4	SS	97.0 ± 16.0	-13.5 ± 9.6	SS
L100<br>B5/H12.5	106.8 ± 16.7	-3.8 ± 11.1	0.11113	104.1 ± 17.6	-5.9 ± 11.5	0.01581	102.8 ± 18.7	-8.1 ± 12.8	0.00025	98.4 ± 16.4	-12.1 ± 11.9	SS	98.0 ± 17.2	-13.2 ± 11.2	SS	99.1 ± 16.4	-11.4 ± 11.1	0.00007

Al300 = aliskiren 300 mg; **Aml5** = amlodipine 5 mg; **B5** = bisoprolol 5 mg; **E20** = enalapril 20 mg; **H12.5** = hydrochlorothiazide 12.5 mg; **L100** = losartan 100 mg; **SS** = statistically significant (P < 0.00001)

Table S25. *P*-values calculated using the Kolmogorov-Smirnov test for changes in left ventricular end-diastolic volume in subpopulations (n = 100) with different ACE activity receiving the same regimens. Case 1: c_{ACE} = 7.0 h^{-1} (*LII*), case 2: c_{ACE} = 8.9 h^{-1} (*LID*), case 3: c_{ACE} = 10.8 h^{-1} (*LDD*), case 4: c_{ACE} = 42.3 h^{-1} (*HII*), case 5: c_{ACE} = 54.1 h^{-1} (*HID*), case 6: c_{ACE} = 65.9 h^{-1} (*HDD*). *P*-value for case *i vs*. case *j* is denoted P_{ij}.

Regimens	P_{12}	P_{13}	P_{23}	P_{14}	P_{15}	P_{16}	P_{24}	P_{25}	P_{26}	P_{34}	P_{35}	P_{36}	P_{45}	P_{46}	P_{56}
Al300	0.28093	0.03663	0.15454	0.01581	0.00004	SS	0.00386	0.00002	SS	0.00013	SS	SS	0.21055	0.00002	0.00136
E20	0.36672	0.00386	0.11113	0.00007	0.00013	0.00386	0.00045	0.00025	0.01008	0.00630	0.00232	0.02431	0.96707	0.15454	0.11113
L100	0.28093	0.01581	0.21055	0.28093	0.03663	SS	0.15454	0.01581	SS	0.01581	0.00013	SS	0.46756	0.00025	0.00630
Aml5	0.46756	0.15454	0.96707	0.58062	0.07832	0.11113	0.69937	0.46756	0.28093	0.96707	0.90621	0.90621	0.69937	0.90621	0.99963
B5	0.15454	0.81275	0.11113	0.00232	SS	SS	0.00007	SS	SS	0.00136	SS	SS	0.11113	0.00079	0.01008
H12.5	0.69937	0.58062	0.28093	0.69937	0.81275	0.81275	0.28093	0.15454	0.21055	0.99376	0.46756	0.36672	0.69937	0.90621	0.99963
Al300 Aml5	0.99376	0.58062	0.58062	0.15454	0.00079	SS	0.07832	0.00045	SS	0.07832	0.00025	SS	0.28093	0.00025	0.00630
Al300 B5	0.11113	0.00232	0.28093	SS	SS	SS	SS	SS	SS	0.00004	SS	0.00004	0.28093	0.58062	0.58062
Al300 H12.5	0.99376	0.46756	0.36672	0.01008	0.00002	SS	0.01008	0.00004	SS	0.05410	0.00007	SS	0.11113	0.00232	0.28093
E20 Aml5	0.46756	0.11113	0.21055	SS	SS	SS	SS	SS	SS	0.00136	0.00079	0.01008	0.90621	0.69937	0.36672
E20 B5	0.15454	0.01008	0.58062	SS	SS	SS	SS	SS	SS	0.00013	SS	SS	0.03663	0.07832	0.46756
E20 H12.5	0.90621	0.21055	0.36672	0.01581	0.11113	0.46756	0.02431	0.15454	0.81275	0.46756	0.69937	0.36672	0.58062	0.28093	0.69937
L100 Aml5	0.96707	0.36672	0.36672	0.69937	0.05410	0.00079	0.58062	0.00630	0.00004	0.28093	0.00232	SS	0.21055	0.00045	0.01581
L100 B5	0.11113	0.00232	0.28093	SS	SS	SS	SS	SS	SS	SS	SS	SS	0.11113	0.28093	0.58062
L100 H12.5	0.90621	0.58062	0.36672	0.03663	0.00025	SS	0.02431	0.00045	SS	0.21055	0.00136	SS	0.21055	0.00232	0.28093
Al300 Aml5/B5	0.07832	0.01008	0.28093	SS	SS	SS	SS	SS	SS	0.00079	SS	SS	0.15454	0.58062	0.81275
Al300 Aml5/H12.5	0.69937	0.46756	0.90621	0.00136	0.00004	SS	0.02431	0.00079	SS	0.07832	0.00079	SS	0.28093	0.00630	0.28093
Al300 B5/H12.5	0.58062	0.01581	0.21055	0.00136	0.00079	0.00136	0.03663	0.02431	0.05410	0.28093	0.03663	0.15454	0.90621	0.81275	0.69937
E20 Aml5/B5	0.21055	0.02431	0.28093	SS	SS	SS	SS	SS	SS	0.00004	SS	SS	0.07832	0.07832	0.58062
E20 Aml5/H12.5	0.69937	0.11113	0.69937	0.00232	0.01581	0.07832	0.00232	0.05410	0.46756	0.11113	0.58062	0.81275	0.69937	0.15454	0.58062
E20 B5/H12.5	0.36672	0.02431	0.11113	SS	SS	SS	0.00004	0.00002	SS	0.02431	0.00045	0.00136	0.58062	0.46756	0.81275
L100 Aml5/B5	0.11113	0.00630	0.15454	SS	SS	SS	SS	SS	SS	0.00004	SS	SS	0.15454	0.21055	0.90621
L100 Aml5/H12.5	0.81275	0.46756	0.81275	0.00630	0.00013	SS	0.07832	0.00232	0.00004	0.05410	0.00232	0.00004	0.36672	0.01581	0.36672
L100 B5/H12.5	0.46756	0.02431	0.15454	0.00004	0.00002	0.00004	0.00386	0.00386	0.00232	0.07832	0.01008	0.03663	0.81275	0.96707	0.81275

Al300 = aliskiren 300 mg; **Aml5** = amlodipine 5 mg; **B5** = bisoprolol 5 mg; **E20** = enalapril 20 mg; **H12.5** = hydrochlorothiazide 12.5 mg; **L100** = losartan 100 mg; **SS** = statistically significant (P < 0.00001)

Figure S20. Simulated change in left ventricular end-systolic volume from baseline to week 4 (mean ± SD, n = 100)

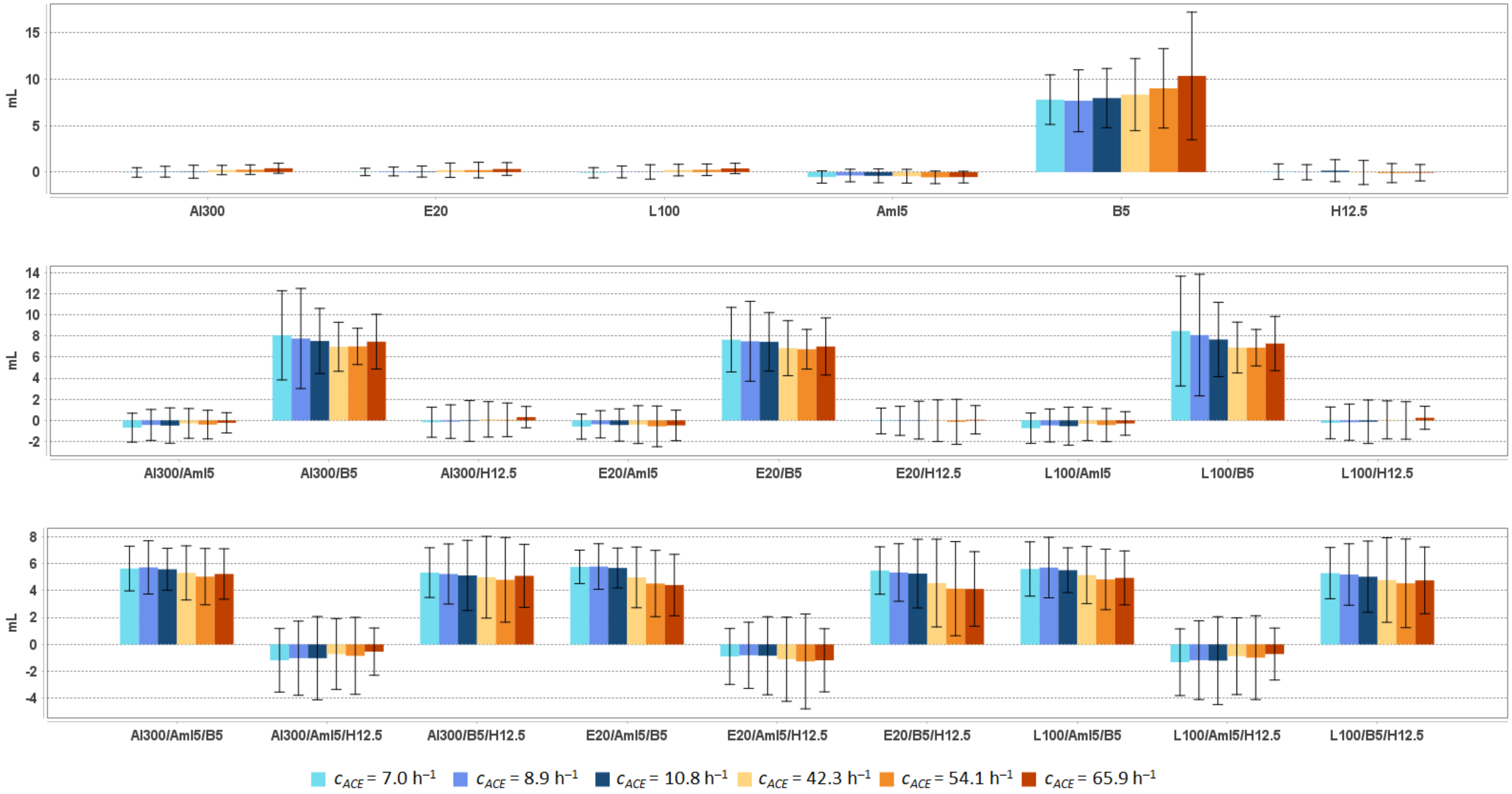

Al300 = aliskiren 300 mg; **Aml5** = amlodipine 5 mg; **B5** = bisoprolol 5 mg; **E20** = enalapril 20 mg; **H12.5** = hydrochlorothiazide 12.5 mg; **L100** = losartan 100 mg

Table S26. Simulated response of left ventricular end-systolic volume to antihypertensive therapy in virtual hypertensive subpopulations (n = 100) with different ACE activity, including P-values (Kolmogorov-Smirnov test) for endpoint *vs.* baseline; data are presented as mean ± SD in mL

Regimens	***LII*** (c_{ACE} = 7.0 h^{-1})			***LID*** (c_{ACE} = 8.9 h^{-1})			***LDD*** (c_{ACE} = 10.8 h^{-1})			***HII*** (c_{ACE} = 42.3 h^{-1})			***HID*** (c_{ACE} = 54.1 h^{-1})			***HDD*** (c_{ACE} = 65.9 h^{-1})		
	**Value**	**Change**	***P***	**Value**	**Change**	***P***	**Value**	**Change**	***P***	**Value**	**Change**	***P***	**Value**	**Change**	***P***	**Value**	**Change**	***P***
Baseline	37.3 ± 9.4	–	–	37.5 ± 10.2	–	–	37.5 ± 10.5	–	–	37.7 ± 10.9	–	–	37.7 ± 10.1	–	–	38.6 ± 10.7	–	–
Al300	37.3 ± 9.2	-0.0 ± 0.5	1.00000	37.5 ± 10.0	0.0 ± 0.6	1.00000	37.5 ± 10.3	0.0 ± 0.7	0.99963	37.9 ± 10.7	0.2 ± 0.5	0.99963	38.0 ± 10.1	0.3 ± 0.5	0.99963	39.0 ± 10.7	0.4 ± 0.5	0.99376
E20	37.4 ± 9.2	0.0 ± 0.4	0.99963	37.6 ± 10.1	0.1 ± 0.5	1.00000	37.5 ± 10.3	0.1 ± 0.6	0.99963	37.9 ± 10.5	0.2 ± 0.8	0.99963	37.9 ± 10.0	0.2 ± 0.8	0.99963	39.0 ± 10.6	0.3 ± 0.7	0.99963
L100	37.3 ± 9.2	-0.1 ± 0.6	1.00000	37.5 ± 10.0	0.0 ± 0.6	1.00000	37.5 ± 10.2	0.0 ± 0.8	0.99963	37.9 ± 10.6	0.2 ± 0.6	0.99963	38.0 ± 10.1	0.3 ± 0.6	0.99963	39.0 ± 10.7	0.4 ± 0.6	0.99376
Aml5	36.8 ± 9.1	-0.5 ± 0.7	0.99376	37.1 ± 9.9	-0.4 ± 0.7	0.99376	37.1 ± 10.1	-0.4 ± 0.7	0.99376	37.3 ± 10.5	-0.4 ± 0.7	0.96707	37.2 ± 9.9	-0.6 ± 0.7	0.99963	38.1 ± 10.5	-0.5 ± 0.6	0.96707
B5	45.1 ± 10.7	7.8 ± 2.7	SS	45.2 ± 12.4	7.7 ± 3.3	0.00136	45.4 ± 12.6	8.0 ± 3.2	0.00079	46.1 ± 13.1	8.3 ± 3.9	0.00025	46.7 ± 12.2	9.0 ± 4.3	SS	49.0 ± 14.0	10.4 ± 6.9	0.00004
H12.5	37.4 ± 9.4	0.0 ± 0.8	1.00000	37.5 ± 10.1	-0.0 ± 0.8	1.00000	37.6 ± 10.3	0.2 ± 1.2	0.99963	37.7 ± 10.6	-0.0 ± 1.3	0.99963	37.6 ± 10.1	-0.1 ± 1.0	1.00000	38.6 ± 10.6	-0.1 ± 0.9	1.00000
Al300<br>Aml5	36.7 ± 9.0	-0.7 ± 1.4	0.90621	37.1 ± 9.5	-0.4 ± 1.5	0.96707	37.0 ± 9.7	-0.5 ± 1.7	0.81275	37.4 ± 10.2	-0.3 ± 1.4	0.69937	37.3 ± 9.9	-0.4 ± 1.4	0.99963	38.4 ± 10.4	-0.2 ± 1.0	0.99963
Al300<br>B5	45.4 ± 11.4	8.1 ± 4.2	SS	45.3 ± 13.2	7.8 ± 4.7	0.00136	45.0 ± 12.3	7.5 ± 3.1	0.00079	44.7 ± 11.6	7.0 ± 2.3	0.00045	44.7 ± 10.4	7.0 ± 1.7	0.00025	46.1 ± 11.5	7.5 ± 2.6	0.00079
Al300<br>H12.5	37.2 ± 9.2	-0.2 ± 1.4	0.99963	37.4 ± 9.7	-0.1 ± 1.6	0.99963	37.4 ± 9.9	-0.0 ± 1.9	0.96707	37.8 ± 10.3	0.1 ± 1.7	0.99963	37.8 ± 10.2	0.1 ± 1.6	1.00000	38.9 ± 10.6	0.3 ± 1.0	0.99963
E20<br>Aml5	36.7 ± 9.0	-0.6 ± 1.2	0.90621	37.1 ± 9.6	-0.4 ± 1.3	0.96707	37.0 ± 9.8	-0.4 ± 1.5	0.90621	37.3 ± 10.0	-0.4 ± 1.8	0.58062	37.2 ± 9.8	-0.6 ± 1.9	0.96707	38.2 ± 10.3	-0.5 ± 1.4	0.96707
E20<br>B5	45.0 ± 10.8	7.6 ± 3.1	0.00004	45.0 ± 12.6	7.5 ± 3.8	0.00136	44.9 ± 12.1	7.4 ± 2.8	0.00079	44.6 ± 11.6	6.8 ± 2.6	0.00079	44.5 ± 10.2	6.7 ± 1.9	0.00045	45.6 ± 11.4	7.0 ± 2.7	0.00079
E20<br>H12.5	37.3 ± 9.3	-0.1 ± 1.2	1.00000	37.4 ± 9.8	-0.0 ± 1.4	0.99963	37.5 ± 9.9	0.0 ± 1.8	0.99376	37.7 ± 10.2	-0.0 ± 2.0	0.81275	37.6 ± 10.1	-0.1 ± 2.1	0.99376	38.7 ± 10.5	0.1 ± 1.3	1.00000
L100<br>Aml5	36.6 ± 9.0	-0.7 ± 1.4	0.90621	37.0 ± 9.5	-0.5 ± 1.6	0.90621	36.9 ± 9.7	-0.5 ± 1.8	0.81275	37.4 ± 10.1	-0.3 ± 1.6	0.69937	37.3 ± 9.8	-0.4 ± 1.6	0.99963	38.3 ± 10.4	-0.3 ± 1.1	0.96707
L100<br>B5	45.8 ± 11.9	8.5 ± 5.2	SS	45.6 ± 14.0	8.1 ± 5.8	0.00136	45.1 ± 12.5	7.7 ± 3.5	0.00079	44.6 ± 11.6	6.9 ± 2.4	0.00045	44.6 ± 10.3	6.9 ± 1.7	0.00045	45.9 ± 11.4	7.3 ± 2.6	0.00079
L100<br>H12.5	37.1 ± 9.2	-0.2 ± 1.5	0.99376	37.3 ± 9.6	-0.2 ± 1.7	0.99376	37.3 ± 9.8	-0.1 ± 2.1	0.90621	37.8 ± 10.3	0.1 ± 1.8	0.99376	37.7 ± 10.1	-0.0 ± 1.8	1.00000	38.9 ± 10.6	0.2 ± 1.1	1.00000
Al300<br>Aml5/B5	43.0 ± 9.6	5.6 ± 1.7	0.00025	43.2 ± 10.8	5.7 ± 2.0	0.01581	43.0 ± 10.5	5.6 ± 1.6	0.01008	43.0 ± 10.4	5.3 ± 2.0	0.00630	42.8 ± 9.8	5.0 ± 2.1	0.00630	43.9 ± 10.8	5.2 ± 1.9	0.00386
Al300<br>Aml5/H12.5	36.2 ± 9.2	-1.2 ± 2.4	0.81275	36.5 ± 9.2	-1.0 ± 2.8	0.69937	36.4 ± 9.6	-1.0 ± 3.1	0.69937	37.0 ± 10.0	-0.7 ± 2.6	0.46756	36.9 ± 10.2	-0.8 ± 2.9	0.90621	38.1 ± 10.4	-0.5 ± 1.8	0.96707
Al300<br>B5/H12.5	42.7 ± 9.6	5.3 ± 1.8	0.00079	42.7 ± 10.2	5.2 ± 2.2	0.02431	42.6 ± 10.3	5.1 ± 2.6	0.00630	42.7 ± 10.3	5.0 ± 3.0	0.01008	42.5 ± 10.2	4.8 ± 3.1	0.02431	43.7 ± 10.7	5.1 ± 2.3	0.01008
E20<br>Aml5/B5	43.1 ± 9.6	5.8 ± 1.2	0.00025	43.3 ± 10.7	5.8 ± 1.7	0.01581	43.1 ± 10.5	5.7 ± 1.5	0.01008	42.7 ± 10.2	5.0 ± 2.2	0.01008	42.2 ± 9.7	4.5 ± 2.5	0.01008	43.0 ± 10.8	4.4 ± 2.3	0.02431
E20<br>Aml5/H12.5	36.4 ± 9.2	-0.9 ± 2.1	0.81275	36.7 ± 9.3	-0.8 ± 2.5	0.81275	36.6 ± 9.6	-0.8 ± 2.9	0.69937	36.6 ± 9.8	-1.1 ± 3.1	0.21055	36.5 ± 10.2	-1.3 ± 3.5	0.81275	37.5 ± 10.3	-1.2 ± 2.3	0.69937
E20<br>B5/H12.5	42.8 ± 9.6	5.5 ± 1.8	0.00079	42.8 ± 10.1	5.3 ± 2.1	0.02431	42.7 ± 10.3	5.3 ± 2.5	0.00630	42.3 ± 10.2	4.6 ± 3.3	0.01581	41.9 ± 10.1	4.1 ± 3.5	0.03663	42.7 ± 10.7	4.1 ± 2.8	0.03663
L100<br>Aml5/B5	42.9 ± 9.7	5.6 ± 2.0	0.00045	43.2 ± 11.0	5.7 ± 2.2	0.01581	43.0 ± 10.5	5.5 ± 1.7	0.01581	42.9 ± 10.3	5.1 ± 2.1	0.00630	42.5 ± 9.7	4.8 ± 2.2	0.01008	43.6 ± 10.8	4.9 ± 2.0	0.00630
L100<br>Aml5/H12.5	36.0 ± 9.2	-1.3 ± 2.5	0.58062	36.3 ± 9.2	-1.2 ± 2.9	0.58062	36.3 ± 9.6	-1.2 ± 3.3	0.69937	36.8 ± 9.9	-0.9 ± 2.8	0.36672	36.7 ± 10.2	-1.0 ± 3.1	0.81275	37.9 ± 10.3	-0.7 ± 1.9	0.90621
L100<br>B5/H12.5	42.6 ± 9.6	5.3 ± 1.9	0.00079	42.7 ± 10.2	5.2 ± 2.3	0.02431	42.5 ± 10.3	5.0 ± 2.6	0.00630	42.5 ± 10.2	4.8 ± 3.1	0.01581	42.3 ± 10.2	4.5 ± 3.3	0.02431	43.4 ± 10.7	4.8 ± 2.5	0.01581

Al300 = aliskiren 300 mg; **Aml5** = amlodipine 5 mg; **B5** = bisoprolol 5 mg; **E20** = enalapril 20 mg; **H12.5** = hydrochlorothiazide 12.5 mg; **L100** = losartan 100 mg; **SS** = statistically significant (P < 0.00001)

Table S27. *P*-values calculated using the Kolmogorov-Smirnov test for changes in left ventricular end-systolic volume in subpopulations (n = 100) with different ACE activity receiving the same regimens. Case 1: c_{ACE} = 7.0 h^{-1} (*LII*), case 2: c_{ACE} = 8.9 h^{-1} (*LID*), case 3: c_{ACE} = 10.8 h^{-1} (*LDD*), case 4: c_{ACE} = 42.3 h^{-1} (*HII*), case 5: c_{ACE} = 54.1 h^{-1} (*HID*), case 6: c_{ACE} = 65.9 h^{-1} (*HDD*). *P*-value for case *i* *vs*. case *j* is denoted P_{ij}.

Regimens	P_{12}	P_{13}	P_{23}	P_{14}	P_{15}	P_{16}	P_{24}	P_{25}	P_{26}	P_{34}	P_{35}	P_{36}	P_{45}	P_{46}	P_{56}
Al300	0.21055	0.58062	0.46756	SS	0.00013	SS	0.02431	0.07832	SS	0.00079	0.00079	SS	0.96707	0.01008	0.00386
E20	0.28093	0.46756	0.46756	0.00007	0.00007	SS	0.01008	0.00136	0.00002	0.00232	0.00232	0.00004	0.69937	0.11113	0.28093
L100	0.15454	0.36672	0.58062	0.00002	0.00013	SS	0.01581	0.05410	SS	0.00045	0.00136	SS	0.96707	0.01581	0.03663
Aml5	0.02431	0.46756	0.03663	0.81275	0.69937	0.69937	0.21055	0.01581	0.00386	0.90621	0.28093	0.28093	0.46756	0.46756	0.90621
B5	0.11113	0.58062	0.03663	0.07832	0.11113	0.00025	0.07832	0.00079	0.00079	0.58062	0.21055	0.01581	0.21055	0.21055	0.28093
H12.5	0.96707	0.21055	0.21055	0.58062	0.36672	0.21055	0.46756	0.11113	0.11113	0.69937	0.21055	0.21055	0.81275	0.81275	0.99376
Al300<br>Aml5	0.15454	0.15454	0.46756	0.03663	0.05410	0.00025	0.58062	0.90621	0.07832	0.11113	0.15454	0.00232	0.69937	0.28093	0.36672
Al300<br>B5	0.03663	0.81275	0.28093	0.02431	0.03663	0.21055	0.21055	0.05410	0.01581	0.07832	0.15454	0.28093	0.28093	0.11113	0.81275
Al300<br>H12.5	0.36672	0.36672	0.69937	0.07832	0.05410	0.00045	0.69937	0.69937	0.01008	0.21055	0.21055	0.00136	0.81275	0.11113	0.28093
E20<br>Aml5	0.21055	0.11113	0.36672	0.07832	0.21055	0.58062	0.46756	0.69937	0.58062	0.58062	0.58062	0.58062	0.90621	0.46756	0.69937
E20<br>B5	0.02431	0.81275	0.36672	0.00386	0.05410	0.00630	0.36672	0.21055	0.46756	0.02431	0.05410	0.05410	0.58062	0.69937	0.90621
E20<br>H12.5	0.46756	0.28093	0.46756	0.15454	0.00630	0.21055	0.46756	0.15454	0.58062	0.81275	0.21055	0.69937	0.69937	0.58062	0.46756
L100<br>Aml5	0.15454	0.15454	0.36672	0.05410	0.03663	0.00136	0.69937	0.99376	0.15454	0.11113	0.28093	0.00630	0.90621	0.46756	0.58062
L100<br>B5	0.05410	0.69937	0.21055	0.00386	0.01581	0.03663	0.11113	0.03663	0.01008	0.05410	0.07832	0.28093	0.58062	0.15454	0.96707
L100<br>H12.5	0.36672	0.46756	0.81275	0.07832	0.02431	0.00232	0.58062	0.36672	0.02431	0.21055	0.11113	0.01581	0.69937	0.36672	0.46756
Al300<br>Aml5/B5	0.46756	0.15454	0.46756	0.69937	0.21055	0.36672	0.58062	0.07832	0.07832	0.81275	0.36672	0.21055	0.81275	0.46756	0.90621
Al300<br>Aml5/H12.5	0.36672	0.46756	0.96707	0.02431	0.00386	0.00386	0.58062	0.36672	0.03663	0.58062	0.21055	0.02431	0.46756	0.36672	0.36672
Al300<br>B5/H12.5	0.58062	0.15454	0.69937	0.05410	0.03663	0.21055	0.46756	0.15454	0.46756	0.46756	0.15454	0.58062	0.81275	0.58062	0.21055
E20<br>Aml5/B5	0.81275	0.15454	0.69937	0.01008	0.00002	SS	0.01581	0.00004	SS	0.28093	0.00386	0.00013	0.36672	0.01581	0.36672
E20<br>Aml5/H12.5	0.28093	0.46756	0.99376	0.28093	0.11113	0.36672	0.69937	0.81275	0.36672	0.96707	0.90621	0.69937	0.90621	0.58062	0.15454
E20<br>B5/H12.5	0.46756	0.21055	0.58062	0.00630	0.00013	0.00013	0.02431	0.01008	0.00079	0.21055	0.11113	0.00232	0.96707	0.02431	0.11113
L100<br>Aml5/B5	0.46756	0.15454	0.36672	0.46756	0.15454	0.28093	0.46756	0.02431	0.03663	0.58062	0.28093	0.07832	0.69937	0.28093	0.69937
L100<br>Aml5/H12.5	0.36672	0.69937	0.99376	0.03663	0.00386	0.03663	0.69937	0.28093	0.15454	0.58062	0.15454	0.05410	0.69937	0.46756	0.28093
L100<br>B5/H12.5	0.58062	0.15454	0.58062	0.03663	0.01581	0.05410	0.28093	0.07832	0.28093	0.81275	0.36672	0.58062	0.99376	0.36672	0.21055

Al300 = aliskiren 300 mg; **Aml5** = amlodipine 5 mg; **B5** = bisoprolol 5 mg; **E20** = enalapril 20 mg; **H12.5** = hydrochlorothiazide 12.5 mg; **L100** = losartan 100 mg; **SS** = statistically significant (P < 0.00001)

Figure S21. Simulated change in right ventricular end-diastolic pressure from baseline to week 4 (mean ± SD, n = 100)

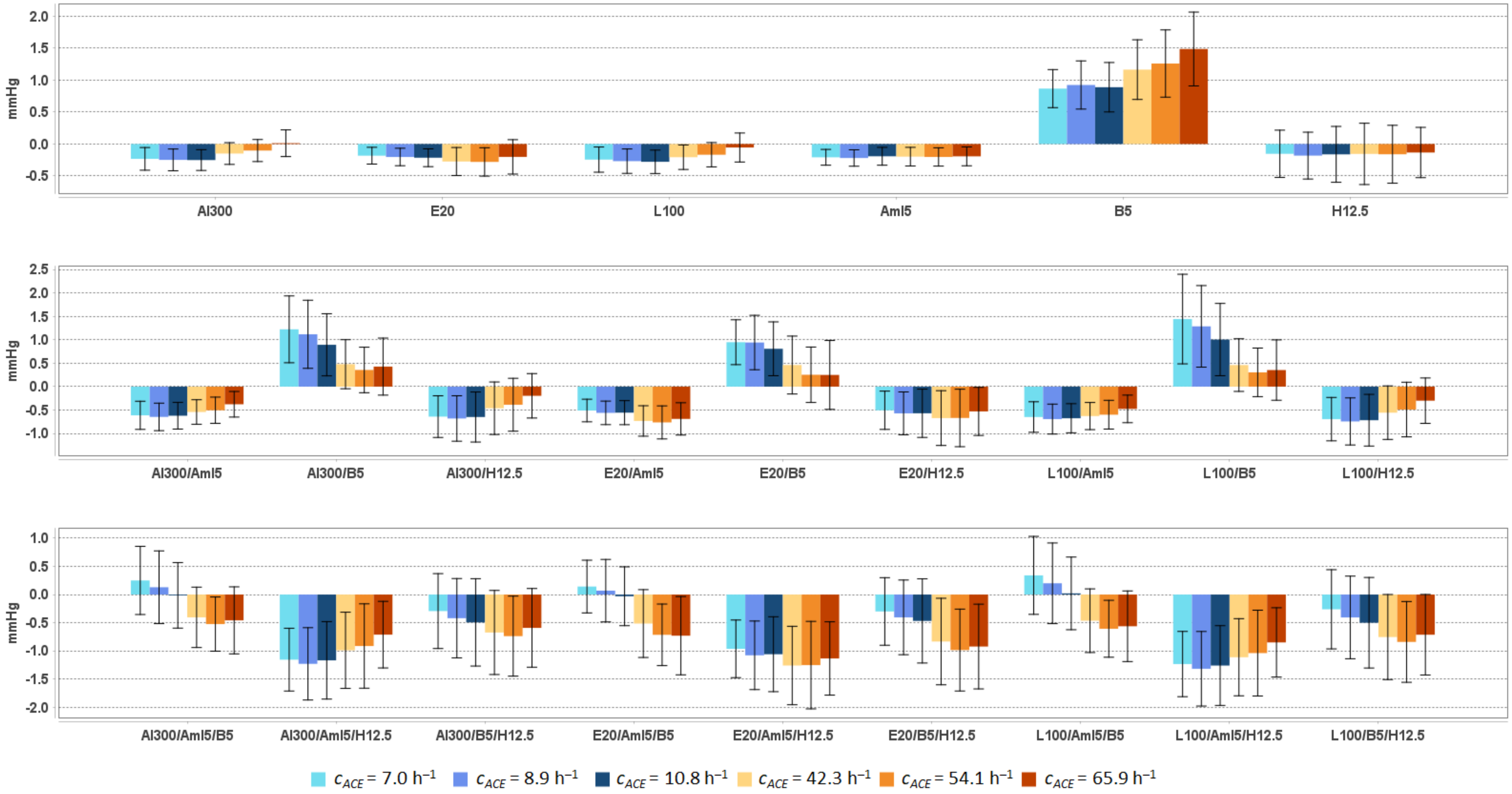

Al300 = aliskiren 300 mg; **Aml5** = amlodipine 5 mg; **B5** = bisoprolol 5 mg; **E20** = enalapril 20 mg; **H12.5** = hydrochlorothiazide 12.5 mg; **L100** = losartan 100 mg

Table S28. Simulated response of <u>right ventricular end-diastolic pressure</u> to antihypertensive therapy in virtual hypertensive subpopulations (n = 100) with different ACE activity, including P-values (Kolmogorov-Smirnov test) for endpoint *vs*. baseline; data are presented as mean ± SD in mmHg

Regimens	***LII*** **(c_{ACE} = 7.0 h^{-1})**			***LID*** **(c_{ACE} = 8.9 h^{-1})**			***LDD*** **(c_{ACE} = 10.8 h^{-1})**			***HII*** **(c_{ACE} = 42.3 h^{-1})**			***HID*** **(c_{ACE} = 54.1 h^{-1})**			***HDD*** **(c_{ACE} = 65.9 h^{-1})**		
	**Value**	**Change**	***P***	**Value**	**Change**	***P***	**Value**	**Change**	***P***	**Value**	**Change**	***P***	**Value**	**Change**	***P***	**Value**	**Change**	***P***
Baseline	5.4 ± 1.2	–	–	5.8 ± 1.1	–	–	5.2 ± 1.2	–	–	5.6 ± 1.1	–	–	5.6 ± 1.1	–	–	5.7 ± 1.0	–	–
Al300	5.2 ± 1.1	-0.2 ± 0.2	0.36672	5.5 ± 1.1	-0.2 ± 0.2	0.05410	5.0 ± 1.2	-0.3 ± 0.2	0.21055	5.5 ± 1.1	-0.1 ± 0.2	0.69937	5.5 ± 1.1	-0.1 ± 0.2	0.90621	5.7 ± 1.0	0.0 ± 0.2	1.00000
E20	5.2 ± 1.1	-0.2 ± 0.1	0.69937	5.6 ± 1.1	-0.2 ± 0.1	0.11113	5.0 ± 1.2	-0.2 ± 0.1	0.28093	5.4 ± 1.0	-0.3 ± 0.2	0.28093	5.3 ± 1.0	-0.3 ± 0.2	0.07832	5.5 ± 1.0	-0.2 ± 0.3	0.15454
L100	5.2 ± 1.1	-0.2 ± 0.2	0.28093	5.5 ± 1.1	-0.3 ± 0.2	0.03663	4.9 ± 1.2	-0.3 ± 0.2	0.15454	5.4 ± 1.1	-0.2 ± 0.2	0.46756	5.4 ± 1.1	-0.2 ± 0.2	0.58062	5.6 ± 1.0	-0.1 ± 0.2	0.90621
Aml5	5.2 ± 1.1	-0.2 ± 0.1	0.58062	5.5 ± 1.1	-0.2 ± 0.1	0.21055	5.0 ± 1.2	-0.2 ± 0.1	0.28093	5.4 ± 1.1	-0.2 ± 0.1	0.58062	5.4 ± 1.1	-0.2 ± 0.1	0.15454	5.5 ± 1.0	-0.2 ± 0.1	0.36672
B5	6.3 ± 1.3	0.9 ± 0.3	0.00013	6.7 ± 1.3	0.9 ± 0.4	SS	6.1 ± 1.5	0.9 ± 0.4	0.00007	6.8 ± 1.3	1.2 ± 0.5	SS	6.9 ± 1.4	1.3 ± 0.5	SS	7.2 ± 1.2	1.5 ± 0.6	SS
H12.5	5.2 ± 1.2	-0.2 ± 0.4	0.36672	5.6 ± 1.1	-0.2 ± 0.4	0.21055	5.1 ± 1.2	-0.2 ± 0.4	0.69937	5.5 ± 1.2	-0.2 ± 0.5	0.46756	5.4 ± 1.1	-0.2 ± 0.5	0.58062	5.6 ± 1.1	-0.1 ± 0.4	0.90621
Al300<br>Aml5	4.8 ± 1.0	-0.6 ± 0.3	0.00045	5.1 ± 1.0	-0.6 ± 0.3	0.00007	4.6 ± 1.1	-0.6 ± 0.3	0.00079	5.1 ± 1.0	-0.5 ± 0.3	0.00386	5.1 ± 1.0	-0.5 ± 0.3	0.00232	5.3 ± 1.0	-0.4 ± 0.3	0.02431
Al300<br>B5	6.6 ± 1.5	1.2 ± 0.7	SS	6.9 ± 1.5	1.1 ± 0.7	SS	6.1 ± 1.6	0.9 ± 0.7	0.00045	6.1 ± 1.2	0.5 ± 0.5	0.01581	5.9 ± 1.2	0.4 ± 0.5	0.00630	6.1 ± 1.2	0.4 ± 0.6	0.03663
Al300<br>H12.5	4.8 ± 1.1	-0.6 ± 0.4	0.00004	5.1 ± 1.0	-0.7 ± 0.5	0.00004	4.6 ± 1.2	-0.6 ± 0.5	0.00025	5.2 ± 1.2	-0.5 ± 0.6	0.00386	5.2 ± 1.1	-0.4 ± 0.6	0.07832	5.5 ± 1.1	-0.2 ± 0.5	0.46756
E20<br>Aml5	4.9 ± 1.1	-0.5 ± 0.2	0.00386	5.2 ± 1.0	-0.6 ± 0.3	0.00013	4.7 ± 1.1	-0.6 ± 0.3	0.00630	4.9 ± 1.0	-0.7 ± 0.3	0.00013	4.8 ± 1.0	-0.8 ± 0.4	SS	5.0 ± 1.0	-0.7 ± 0.3	0.00013
E20<br>B5	6.3 ± 1.4	0.9 ± 0.5	0.00025	6.7 ± 1.4	0.9 ± 0.6	SS	6.0 ± 1.6	0.8 ± 0.6	0.00079	6.1 ± 1.3	0.5 ± 0.6	0.01581	5.8 ± 1.3	0.3 ± 0.6	0.07832	5.9 ± 1.3	0.3 ± 0.7	0.11113
E20<br>H12.5	4.9 ± 1.1	-0.5 ± 0.4	0.00386	5.2 ± 1.0	-0.6 ± 0.5	0.00025	4.6 ± 1.2	-0.6 ± 0.5	0.00386	5.0 ± 1.1	-0.7 ± 0.6	0.00013	4.9 ± 1.1	-0.7 ± 0.6	0.00025	5.2 ± 1.1	-0.5 ± 0.5	0.00386
L100<br>Aml5	4.7 ± 1.0	-0.6 ± 0.3	0.00013	5.1 ± 1.0	-0.7 ± 0.3	0.00002	4.5 ± 1.1	-0.7 ± 0.3	0.00045	5.0 ± 1.0	-0.6 ± 0.3	0.00079	5.0 ± 1.0	-0.6 ± 0.3	0.00025	5.2 ± 1.0	-0.5 ± 0.3	0.01008
L100<br>B5	6.8 ± 1.7	1.4 ± 1.0	SS	7.1 ± 1.6	1.3 ± 0.9	SS	6.2 ± 1.7	1.0 ± 0.8	0.00007	6.1 ± 1.3	0.5 ± 0.6	0.01581	5.9 ± 1.2	0.3 ± 0.5	0.01581	6.0 ± 1.2	0.4 ± 0.6	0.07832
L100<br>H12.5	4.7 ± 1.1	-0.7 ± 0.5	0.00002	5.0 ± 1.0	-0.7 ± 0.5	SS	4.5 ± 1.2	-0.7 ± 0.6	0.00013	5.1 ± 1.2	-0.6 ± 0.6	0.00045	5.1 ± 1.1	-0.5 ± 0.6	0.01581	5.4 ± 1.1	-0.3 ± 0.5	0.07832
Al300<br>Aml5/B5	5.6 ± 1.3	0.3 ± 0.6	0.28093	5.9 ± 1.3	0.1 ± 0.6	0.21055	5.2 ± 1.4	-0.0 ± 0.6	0.69937	5.2 ± 1.1	-0.4 ± 0.5	0.01581	5.1 ± 1.1	-0.5 ± 0.5	0.00386	5.2 ± 1.1	-0.5 ± 0.6	0.01008
Al300<br>Aml5/H12.5	4.2 ± 1.0	-1.2 ± 0.6	SS	4.5 ± 1.0	-1.2 ± 0.6	SS	4.0 ± 1.2	-1.2 ± 0.7	SS	4.6 ± 1.2	-1.0 ± 0.7	SS	4.7 ± 1.1	-0.9 ± 0.7	SS	5.0 ± 1.1	-0.7 ± 0.6	0.00025
Al300<br>B5/H12.5	5.1 ± 1.2	-0.3 ± 0.7	0.05410	5.3 ± 1.2	-0.4 ± 0.7	0.00386	4.7 ± 1.4	-0.5 ± 0.8	0.00386	5.0 ± 1.2	-0.7 ± 0.7	0.00013	4.9 ± 1.1	-0.7 ± 0.7	0.00002	5.1 ± 1.2	-0.6 ± 0.7	0.00232
E20<br>Aml5/B5	5.5 ± 1.2	0.1 ± 0.5	0.90621	5.8 ± 1.2	0.1 ± 0.6	0.36672	5.2 ± 1.4	-0.0 ± 0.5	0.69937	5.1 ± 1.2	-0.5 ± 0.6	0.00079	4.9 ± 1.1	-0.7 ± 0.5	0.00013	5.0 ± 1.2	-0.7 ± 0.7	0.00007
E20<br>Aml5/H12.5	4.4 ± 1.1	-1.0 ± 0.5	SS	4.7 ± 1.0	-1.1 ± 0.6	SS	4.2 ± 1.2	-1.1 ± 0.7	SS	4.4 ± 1.1	-1.3 ± 0.7	SS	4.3 ± 1.1	-1.3 ± 0.8	SS	4.6 ± 1.1	-1.1 ± 0.7	SS
E20<br>B5/H12.5	5.1 ± 1.2	-0.3 ± 0.6	0.02431	5.4 ± 1.2	-0.4 ± 0.7	0.00630	4.7 ± 1.3	-0.5 ± 0.7	0.00386	4.8 ± 1.2	-0.8 ± 0.8	SS	4.6 ± 1.1	-1.0 ± 0.7	SS	4.8 ± 1.2	-0.9 ± 0.8	0.00002
L100<br>Aml5/B5	5.7 ± 1.4	0.3 ± 0.7	0.15454	6.0 ± 1.3	0.2 ± 0.7	0.07832	5.2 ± 1.5	0.0 ± 0.6	0.58062	5.2 ± 1.1	-0.5 ± 0.6	0.01581	5.0 ± 1.1	-0.6 ± 0.5	0.00079	5.1 ± 1.2	-0.6 ± 0.6	0.00232
L100<br>Aml5/H12.5	4.2 ± 1.0	-1.2 ± 0.6	SS	4.4 ± 1.0	-1.3 ± 0.7	SS	4.0 ± 1.1	-1.3 ± 0.7	SS	4.5 ± 1.1	-1.1 ± 0.7	SS	4.6 ± 1.1	-1.0 ± 0.8	SS	4.8 ± 1.1	-0.8 ± 0.6	SS
L100<br>B5/H12.5	5.1 ± 1.3	-0.3 ± 0.7	0.07832	5.4 ± 1.2	-0.4 ± 0.7	0.00630	4.7 ± 1.4	-0.5 ± 0.8	0.00386	4.9 ± 1.2	-0.8 ± 0.8	0.00007	4.8 ± 1.1	-0.8 ± 0.7	SS	5.0 ± 1.2	-0.7 ± 0.7	0.00045

Al300 = aliskiren 300 mg; **Aml5** = amlodipine 5 mg; **B5** = bisoprolol 5 mg; **E20** = enalapril 20 mg; **H12.5** = hydrochlorothiazide 12.5 mg; **L100** = losartan 100 mg; **SS** = statistically significant (P < 0.00001)

Table S29. *P*-values calculated using the Kolmogorov-Smirnov test for changes in right ventricular end-diastolic pressure in subpopulations (n = 100) with different ACE activity receiving the same regimens. Case 1: c_{ACE} = 7.0 h^{-1} (*LII*), case 2: c_{ACE} = 8.9 h^{-1} (*LID*), case 3: c_{ACE} = 10.8 h^{-1} (*LDD*), case 4: c_{ACE} = 42.3 h^{-1} (*HII*), case 5: c_{ACE} = 54.1 h^{-1} (*HID*), case 6: c_{ACE} = 65.9 h^{-1} (*HDD*). *P*-value for case *i vs*. case *j* is denoted P_{ij}.

Regimens	P_{12}	P_{13}	P_{23}	P_{14}	P_{15}	P_{16}	P_{24}	P_{25}	P_{26}	P_{34}	P_{35}	P_{36}	P_{45}	P_{46}	P_{56}
Al300	0.46756	0.05410	0.36672	0.02431	0.00004	SS	0.00386	SS	SS	0.00232	SS	SS	0.15454	SS	0.00386
E20	0.28093	0.02431	0.15454	0.00025	0.00136	0.01008	0.00630	0.00232	0.02431	0.01008	0.00630	0.03663	0.90621	0.15454	0.11113
L100	0.28093	0.01008	0.28093	0.28093	0.05410	SS	0.28093	0.00630	SS	0.01581	0.00004	SS	0.28093	0.00025	0.01008
Aml5	0.07832	0.58062	0.21055	0.28093	0.36672	0.36672	0.21055	0.28093	0.11113	0.28093	0.69937	0.81275	0.69937	0.81275	0.99376
B5	0.46756	0.81275	0.46756	0.00007	SS	SS	0.00079	0.00002	SS	0.00136	SS	SS	0.46756	0.00232	0.05410
H12.5	0.58062	0.81275	0.69937	0.28093	0.69937	0.81275	0.58062	0.07832	0.21055	0.96707	0.36672	0.46756	0.81275	0.36672	0.81275
Al300<br>Aml5	0.15454	0.58062	0.69937	0.46756	0.11113	0.00045	0.03663	0.00136	SS	0.15454	0.01008	0.00002	0.58062	0.00630	0.11113
Al300<br>B5	0.46756	0.01008	0.11113	SS	SS	SS	SS	SS	SS	0.00232	SS	0.00002	0.11113	0.36672	0.81275
Al300<br>H12.5	0.81275	0.28093	0.28093	0.01581	0.00002	SS	0.01008	SS	SS	0.15454	0.00136	SS	0.28093	0.00386	0.21055
E20<br>Aml5	0.05410	0.05410	0.81275	0.00002	SS	0.00004	0.00079	0.00232	0.01008	0.00386	0.00136	0.00630	0.99376	0.58062	0.69937
E20<br>B5	0.69937	0.02431	0.21055	SS	SS	SS	0.00002	SS	SS	0.00079	SS	SS	0.05410	0.07832	0.90621
E20<br>H12.5	0.46756	0.21055	0.46756	0.01008	0.11113	0.36672	0.21055	0.58062	0.81275	0.58062	0.69937	0.90621	0.46756	0.46756	0.46756
L100<br>Aml5	0.11113	0.46756	0.69937	0.96707	0.81275	0.01581	0.21055	0.02431	0.00013	0.81275	0.11113	0.00232	0.69937	0.02431	0.21055
L100<br>B5	0.36672	0.00630	0.07832	SS	SS	SS	SS	SS	SS	0.00045	SS	SS	0.05410	0.21055	0.81275
L100<br>H12.5	0.81275	0.21055	0.28093	0.03663	0.00013	SS	0.01581	0.00004	SS	0.36672	0.00630	0.00013	0.21055	0.01008	0.36672
Al300<br>Aml5/B5	0.07832	0.01008	0.36672	SS	SS	SS	SS	SS	SS	0.00136	SS	0.00002	0.11113	0.58062	0.58062
Al300<br>Aml5/H12.5	0.15454	0.36672	0.69937	0.05410	0.00136	SS	0.07832	0.00025	SS	0.36672	0.01008	0.00079	0.21055	0.02431	0.36672
Al300<br>B5/H12.5	0.15454	0.07832	0.36672	0.00136	0.00079	0.00232	0.11113	0.03663	0.28093	0.21055	0.05410	0.36672	0.81275	0.58062	0.36672
E20<br>Aml5/B5	0.15454	0.01581	0.58062	SS	SS	SS	SS	SS	SS	SS	SS	SS	0.05410	0.05410	0.58062
E20<br>Aml5/H12.5	0.03663	0.07832	0.81275	0.00013	0.00630	0.01581	0.21055	0.46756	0.28093	0.11113	0.58062	0.11113	0.69937	0.46756	0.81275
E20<br>B5/H12.5	0.58062	0.05410	0.46756	0.00002	SS	SS	0.00079	0.00002	SS	0.00386	0.00007	0.00079	0.69937	0.28093	0.58062
L100<br>Aml5/B5	0.11113	0.00630	0.28093	SS	SS	SS	SS	SS	SS	0.00007	SS	SS	0.07832	0.36672	0.81275
L100<br>Aml5/H12.5	0.21055	0.36672	0.69937	0.28093	0.00386	0.00013	0.11113	0.00136	0.00013	0.58062	0.01008	0.00386	0.36672	0.03663	0.46756
L100<br>B5/H12.5	0.07832	0.05410	0.15454	0.00004	SS	0.00004	0.01581	0.00232	0.00630	0.07832	0.01008	0.07832	0.69937	0.69937	0.58062

Al300 = aliskiren 300 mg; **Aml5** = amlodipine 5 mg; **B5** = bisoprolol 5 mg; **E20** = enalapril 20 mg; **H12.5** = hydrochlorothiazide 12.5 mg; **L100** = losartan 100 mg; **SS** = statistically significant ($P < 0.00001$)

Figure S22. Simulated change in right ventricular peak systolic pressure from baseline to week 4 (mean ± SD, n = 100)

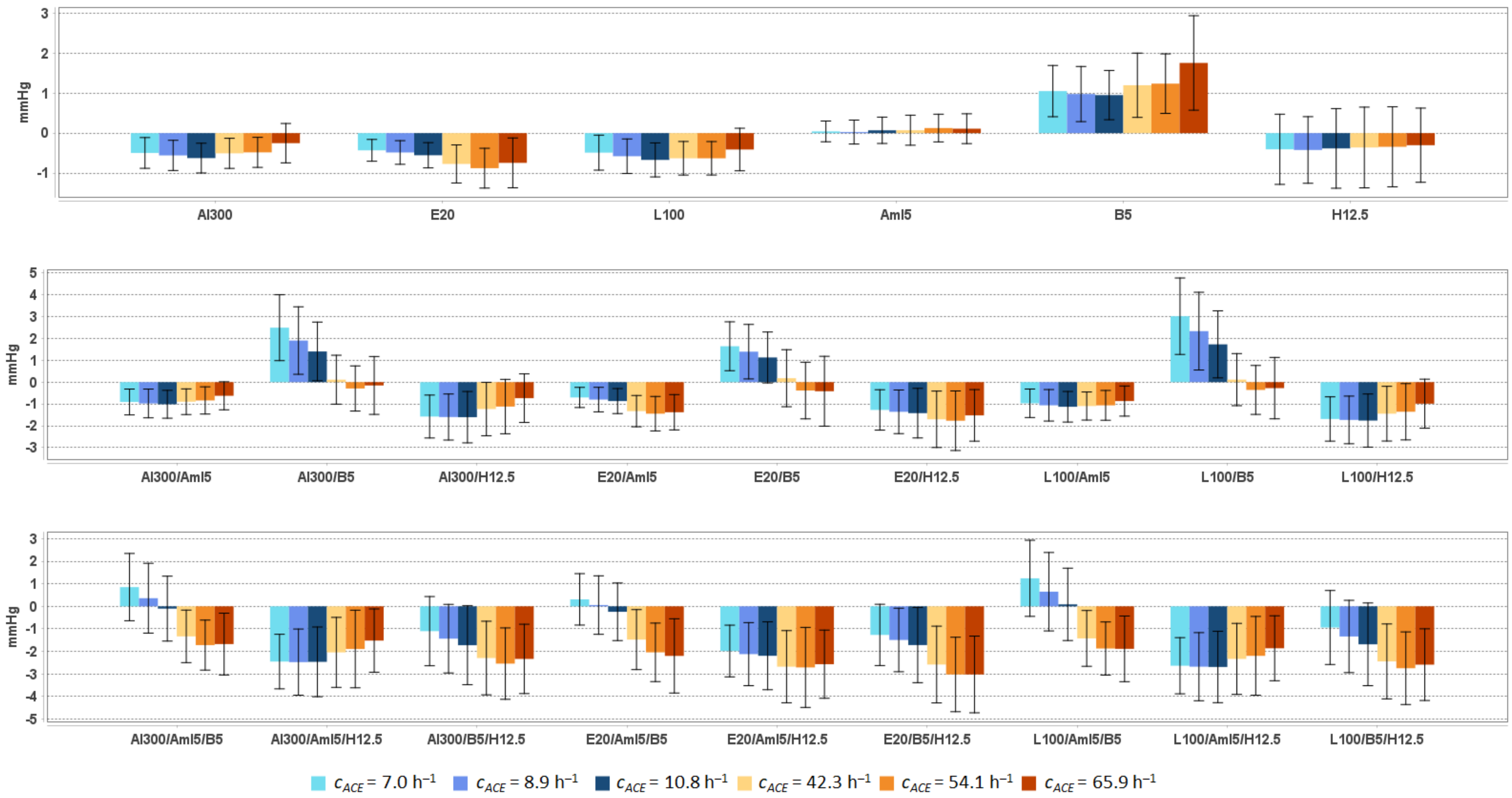

Al300 = aliskiren 300 mg; **Aml5** = amlodipine 5 mg; **B5** = bisoprolol 5 mg; **E20** = enalapril 20 mg; **H12.5** = hydrochlorothiazide 12.5 mg; **L100** = losartan 100 mg

Table S30. Simulated response of <u>right ventricular peak systolic pressure</u> to antihypertensive therapy in virtual hypertensive subpopulations (n = 100) with different ACE activity, including P-values (Kolmogorov-Smirnov test) for endpoint *vs*. baseline; data are presented as mean ± SD in mmHg

**Regimens**	***LII*** **(c_{ACE} = 7.0 h^{-1})**			***LID*** **(c_{ACE} = 8.9 h^{-1})**			***LDD*** **(c_{ACE} = 10.8 h^{-1})**			***HII*** **(c_{ACE} = 42.3 h^{-1})**			***HID*** **(c_{ACE} = 54.1 h^{-1})**			***HDD*** **(c_{ACE} = 65.9 h^{-1})**		
	**Value**	**Change**	***P***	**Value**	**Change**	***P***	**Value**	**Change**	***P***	**Value**	**Change**	***P***	**Value**	**Change**	***P***	**Value**	**Change**	***P***
Baseline	20.2 ± 1.6	–	–	19.9 ± 1.4	–	–	19.9 ± 1.6	–	–	19.7 ± 1.5	–	–	20.1 ± 1.6	–	–	20.1 ± 1.7	–	–
Al300	19.7 ± 1.6	-0.5 ± 0.4	0.15454	19.4 ± 1.4	-0.6 ± 0.4	0.02431	19.3 ± 1.5	-0.6 ± 0.4	0.05410	19.2 ± 1.4	-0.5 ± 0.4	0.05410	19.6 ± 1.6	-0.5 ± 0.4	0.15454	19.9 ± 1.6	-0.3 ± 0.5	0.36672
E20	19.8 ± 1.6	-0.4 ± 0.3	0.21055	19.4 ± 1.4	-0.5 ± 0.3	0.05410	19.4 ± 1.5	-0.6 ± 0.3	0.07832	19.0 ± 1.4	-0.8 ± 0.5	0.00232	19.2 ± 1.6	-0.9 ± 0.5	0.00630	19.4 ± 1.6	-0.7 ± 0.6	0.01581
L100	19.7 ± 1.6	-0.5 ± 0.4	0.15454	19.3 ± 1.4	-0.6 ± 0.4	0.02431	19.2 ± 1.5	-0.7 ± 0.4	0.02431	19.1 ± 1.4	-0.6 ± 0.4	0.01008	19.5 ± 1.6	-0.6 ± 0.4	0.07832	19.7 ± 1.6	-0.4 ± 0.5	0.36672
Aml5	20.2 ± 1.6	0.0 ± 0.3	0.99376	19.9 ± 1.4	0.0 ± 0.3	0.90621	20.0 ± 1.6	0.1 ± 0.3	0.99376	19.8 ± 1.6	0.1 ± 0.4	0.90621	20.2 ± 1.7	0.1 ± 0.3	0.90621	20.2 ± 1.7	0.1 ± 0.4	0.96707
B5	21.2 ± 1.7	1.0 ± 0.6	0.00013	20.9 ± 1.5	1.0 ± 0.7	0.00007	20.9 ± 1.7	1.0 ± 0.6	0.00079	20.9 ± 1.6	1.2 ± 0.8	SS	21.3 ± 1.8	1.2 ± 0.7	0.00013	21.9 ± 1.9	1.8 ± 1.2	SS
H12.5	19.8 ± 1.8	-0.4 ± 0.9	0.15454	19.5 ± 1.5	-0.4 ± 0.8	0.05410	19.5 ± 1.8	-0.4 ± 1.0	0.36672	19.4 ± 1.7	-0.4 ± 1.0	0.21055	19.8 ± 1.8	-0.3 ± 1.0	0.15454	19.8 ± 1.9	-0.3 ± 0.9	0.58062
Al300<br>Aml5	19.3 ± 1.7	-0.9 ± 0.6	0.00386	18.9 ± 1.4	-1.0 ± 0.7	0.00025	18.9 ± 1.6	-1.0 ± 0.6	0.00079	18.8 ± 1.4	-0.9 ± 0.6	0.00079	19.3 ± 1.7	-0.8 ± 0.6	0.00630	19.5 ± 1.6	-0.6 ± 0.6	0.07832
Al300<br>B5	22.7 ± 2.3	2.5 ± 1.5	SS	21.8 ± 2.1	1.9 ± 1.5	SS	21.3 ± 2.1	1.4 ± 1.3	0.00007	19.8 ± 1.6	0.1 ± 1.1	0.81275	19.8 ± 1.8	-0.3 ± 1.0	0.69937	20.0 ± 1.8	-0.2 ± 1.3	0.58062
Al300<br>H12.5	18.6 ± 1.8	-1.6 ± 1.0	SS	18.3 ± 1.5	-1.6 ± 1.1	SS	18.3 ± 1.8	-1.6 ± 1.2	SS	18.5 ± 1.7	-1.2 ± 1.2	0.00002	19.0 ± 1.9	-1.1 ± 1.2	0.00013	19.4 ± 1.8	-0.7 ± 1.1	0.01008
E20<br>Aml5	19.5 ± 1.6	-0.7 ± 0.5	0.02431	19.1 ± 1.4	-0.8 ± 0.6	0.00232	19.0 ± 1.6	-0.9 ± 0.6	0.00136	18.4 ± 1.5	-1.3 ± 0.7	SS	18.7 ± 1.7	-1.4 ± 0.8	0.00004	18.8 ± 1.6	-1.4 ± 0.8	SS
E20<br>B5	21.8 ± 2.0	1.6 ± 1.1	SS	21.3 ± 1.9	1.4 ± 1.2	SS	21.0 ± 2.0	1.1 ± 1.2	0.00136	19.9 ± 1.7	0.2 ± 1.3	0.36672	19.7 ± 1.9	-0.4 ± 1.3	0.28093	19.7 ± 2.0	-0.4 ± 1.6	0.28093
E20<br>H12.5	18.9 ± 1.8	-1.3 ± 0.9	0.00004	18.6 ± 1.5	-1.4 ± 1.0	SS	18.5 ± 1.8	-1.4 ± 1.1	SS	18.0 ± 1.7	-1.7 ± 1.3	SS	18.3 ± 1.9	-1.8 ± 1.4	SS	18.6 ± 1.8	-1.5 ± 1.2	SS
L100<br>Aml5	19.2 ± 1.7	-1.0 ± 0.7	0.00232	18.9 ± 1.4	-1.1 ± 0.7	0.00013	18.8 ± 1.6	-1.1 ± 0.7	0.00013	18.6 ± 1.5	-1.1 ± 0.6	0.00004	19.0 ± 1.7	-1.1 ± 0.7	0.00136	19.3 ± 1.6	-0.9 ± 0.7	0.00386
L100<br>B5	23.2 ± 2.5	3.0 ± 1.7	SS	22.2 ± 2.3	2.3 ± 1.8	SS	21.6 ± 2.2	1.7 ± 1.5	SS	19.8 ± 1.7	0.1 ± 1.2	0.58062	19.7 ± 1.8	-0.4 ± 1.1	0.46756	19.9 ± 1.9	-0.3 ± 1.4	0.36672
L100<br>H12.5	18.5 ± 1.8	-1.7 ± 1.0	SS	18.2 ± 1.5	-1.7 ± 1.1	SS	18.1 ± 1.8	-1.8 ± 1.2	SS	18.3 ± 1.7	-1.4 ± 1.3	SS	18.7 ± 1.9	-1.4 ± 1.3	SS	19.1 ± 1.8	-1.0 ± 1.1	0.00232
Al300<br>Aml5/B5	21.0 ± 2.2	0.9 ± 1.5	0.00045	20.3 ± 2.1	0.4 ± 1.5	0.07832	19.8 ± 2.1	-0.1 ± 1.4	0.21055	18.4 ± 1.6	-1.3 ± 1.2	SS	18.4 ± 1.8	-1.7 ± 1.1	SS	18.5 ± 1.8	-1.7 ± 1.4	SS
Al300<br>Aml5/H12.5	17.7 ± 2.0	-2.4 ± 1.2	SS	17.4 ± 1.8	-2.5 ± 1.5	SS	17.4 ± 2.1	-2.5 ± 1.5	SS	17.7 ± 1.9	-2.0 ± 1.6	SS	18.2 ± 2.2	-1.9 ± 1.7	SS	18.6 ± 2.0	-1.5 ± 1.4	SS
Al300<br>B5/H12.5	19.1 ± 2.2	-1.1 ± 1.5	0.00004	18.5 ± 1.8	-1.4 ± 1.5	SS	18.2 ± 2.2	-1.7 ± 1.8	SS	17.4 ± 1.9	-2.3 ± 1.6	SS	17.6 ± 2.0	-2.5 ± 1.6	SS	17.8 ± 1.9	-2.3 ± 1.5	SS
E20<br>Aml5/B5	20.5 ± 2.0	0.3 ± 1.1	0.11113	20.0 ± 1.9	0.1 ± 1.3	0.58062	19.7 ± 2.0	-0.2 ± 1.3	0.21055	18.3 ± 1.7	-1.5 ± 1.3	SS	18.1 ± 1.9	-2.0 ± 1.3	SS	17.9 ± 2.0	-2.2 ± 1.6	SS
E20<br>Aml5/H12.5	18.2 ± 1.9	-2.0 ± 1.1	SS	17.8 ± 1.8	-2.1 ± 1.4	SS	17.7 ± 2.0	-2.2 ± 1.5	SS	17.1 ± 1.9	-2.7 ± 1.6	SS	17.4 ± 2.2	-2.7 ± 1.8	SS	17.6 ± 2.1	-2.6 ± 1.5	SS
E20<br>B5/H12.5	18.9 ± 2.0	-1.3 ± 1.4	SS	18.4 ± 1.7	-1.5 ± 1.4	SS	18.2 ± 2.1	-1.7 ± 1.7	SS	17.1 ± 1.9	-2.6 ± 1.7	SS	17.1 ± 2.0	-3.0 ± 1.7	SS	17.1 ± 2.0	-3.0 ± 1.7	SS
L100<br>Aml5/B5	21.4 ± 2.4	1.2 ± 1.7	SS	20.6 ± 2.2	0.6 ± 1.7	0.00630	20.0 ± 2.2	0.1 ± 1.6	0.36672	18.3 ± 1.7	-1.4 ± 1.2	SS	18.2 ± 1.8	-1.9 ± 1.2	SS	18.2 ± 1.8	-1.9 ± 1.5	SS
L100<br>Aml5/H12.5	17.6 ± 2.0	-2.6 ± 1.2	SS	17.2 ± 1.8	-2.7 ± 1.5	SS	17.2 ± 2.1	-2.7 ± 1.6	SS	17.4 ± 1.9	-2.3 ± 1.6	SS	17.9 ± 2.2	-2.2 ± 1.7	SS	18.3 ± 2.1	-1.9 ± 1.4	SS
L100<br>B5/H12.5	19.3 ± 2.3	-0.9 ± 1.6	0.00025	18.6 ± 1.9	-1.3 ± 1.6	SS	18.2 ± 2.3	-1.7 ± 1.8	SS	17.3 ± 1.9	-2.4 ± 1.7	SS	17.3 ± 2.0	-2.7 ± 1.6	SS	17.5 ± 1.9	-2.6 ± 1.6	SS

Al300 = aliskiren 300 mg; **Aml5** = amlodipine 5 mg; **B5** = bisoprolol 5 mg; **E20** = enalapril 20 mg; **H12.5** = hydrochlorothiazide 12.5 mg; **L100** = losartan 100 mg; **SS** = statistically significant (P < 0.00001)

Table S31. *P*-values calculated using the Kolmogorov-Smirnov test for changes in right ventricular peak systolic pressure in subpopulations (n = 100) with different ACE activity receiving the same regimens. Case 1: $c_{ACE} = 7.0$ h^{-1} (*LII*), case 2: $c_{ACE} = 8.9$ h^{-1} (*LID*), case 3: $c_{ACE} = 10.8$ h^{-1} (*LDD*), case 4: $c_{ACE} = 42.3$ h^{-1} (*HII*), case 5: $c_{ACE} = 54.1$ h^{-1} (*HID*), case 6: $c_{ACE} = 65.9$ h^{-1} (*HDD*). *P*-value for case *i vs*. case *j* is denoted P_{ij}.

**Regimens**	P_{12}	P_{13}	P_{23}	P_{14}	P_{15}	P_{16}	P_{24}	P_{25}	P_{26}	P_{34}	P_{35}	P_{36}	P_{45}	P_{46}	P_{56}
Al300	0.36672	0.00386	0.11113	0.58062	0.96707	0.00045	0.81275	0.58062	0.00007	0.03663	0.01581	SS	0.96707	0.00232	0.00232
E20	0.28093	0.00045	0.07832	SS	SS	SS	0.00004	SS	SS	0.00136	0.00002	0.00079	0.69937	0.36672	0.07832
L100	0.36672	0.00232	0.15454	0.03663	0.05410	0.21055	0.69937	0.81275	0.01008	0.69937	0.69937	0.00386	0.99376	0.00630	0.01008
Aml5	0.46756	0.90621	0.69937	0.28093	0.02431	0.11113	0.15454	0.00630	0.11113	0.69937	0.15454	0.46756	0.21055	0.69937	0.81275
B5	0.28093	0.81275	0.46756	0.36672	0.36672	0.00045	0.02431	0.01008	SS	0.03663	0.03663	0.00002	0.90621	0.01008	0.00232
H12.5	0.58062	0.69937	0.28093	0.81275	0.81275	0.69937	0.28093	0.07832	0.05410	0.99376	0.46756	0.28093	0.28093	0.58062	0.99963
Al300 Aml5	0.69937	0.21055	0.96707	0.46756	0.36672	0.03663	0.15454	0.01581	0.00045	0.28093	0.00232	0.00079	0.07832	0.00630	0.15454
Al300 B5	0.02431	0.00002	0.01581	SS	SS	SS	SS	SS	SS	SS	SS	SS	0.01581	0.28093	0.21055
Al300 H12.5	0.90621	0.36672	0.36672	0.05410	0.00013	SS	0.05410	0.00232	SS	0.15454	0.00025	SS	0.07832	0.01581	0.28093
E20 Aml5	0.11113	0.07832	0.58062	SS	SS	SS	SS	SS	SS	0.00007	0.00007	SS	0.58062	0.90621	0.69937
E20 B5	0.36672	0.00079	0.01008	SS	SS	SS	SS	SS	SS	0.00007	SS	SS	0.01008	0.01008	0.58062
E20 H12.5	0.81275	0.11113	0.21055	0.00232	0.07832	0.02431	0.00630	0.21055	0.03663	0.15454	0.15454	0.58062	0.58062	0.58062	0.69937
L100 Aml5	0.36672	0.07832	0.69937	0.15454	0.46756	0.46756	0.90621	0.69937	0.21055	0.81275	0.15454	0.15454	0.36672	0.07832	0.21055
L100 B5	0.03663	SS	0.02431	SS	SS	SS	SS	SS	SS	SS	SS	SS	0.01581	0.05410	0.28093
L100 H12.5	0.90621	0.36672	0.46756	0.15454	0.00136	0.00004	0.15454	0.00630	0.00045	0.21055	0.00386	0.00045	0.07832	0.03663	0.28093
Al300 Aml5/B5	0.01581	0.00025	0.15454	SS	SS	SS	SS	SS	SS	SS	SS	SS	0.01581	0.15454	0.69937
Al300 Aml5/H12.5	0.46756	0.46756	0.96707	0.00386	0.00007	SS	0.11113	0.01008	0.00079	0.07832	0.00630	0.00136	0.21055	0.11113	0.58062
Al300 B5/H12.5	0.46756	0.00630	0.36672	SS	SS	SS	0.00386	0.00079	0.00045	0.07832	0.00386	0.02431	0.58062	0.90621	0.81275
E20 Aml5/B5	0.05410	0.01581	0.21055	SS	SS	SS	SS	SS	SS	SS	SS	SS	0.01008	0.00630	0.21055
E20 Aml5/H12.5	0.46756	0.28093	0.81275	0.00079	0.00386	0.00079	0.01581	0.15454	0.05410	0.15454	0.36672	0.11113	0.58062	0.58062	0.81275
E20 B5/H12.5	0.28093	0.00630	0.28093	SS	SS	SS	0.00002	SS	SS	0.01581	0.00025	0.00002	0.36672	0.21055	0.46756
L100 Aml5/B5	0.03663	0.00025	0.05410	SS	SS	SS	SS	SS	SS	SS	SS	SS	0.01008	0.11113	0.69937
L100 Aml5/H12.5	0.69937	0.81275	0.99376	0.01008	0.00136	0.00045	0.28093	0.03663	0.01008	0.15454	0.00630	0.00232	0.28093	0.15454	0.81275
L100 B5/H12.5	0.46756	0.00386	0.28093	SS	SS	SS	0.00079	0.00002	SS	0.02431	0.00136	0.00630	0.58062	0.58062	0.46756

Al300 = aliskiren 300 mg; **Aml5** = amlodipine 5 mg; **B5** = bisoprolol 5 mg; **E20** = enalapril 20 mg; **H12.5** = hydrochlorothiazide 12.5 mg; **L100** = losartan 100 mg; **SS** = statistically significant ($P < 0.00001$)

Figure S23. Simulated change in right ventricular end-diastolic volume from baseline to week 4 (mean ± SD, n = 100)

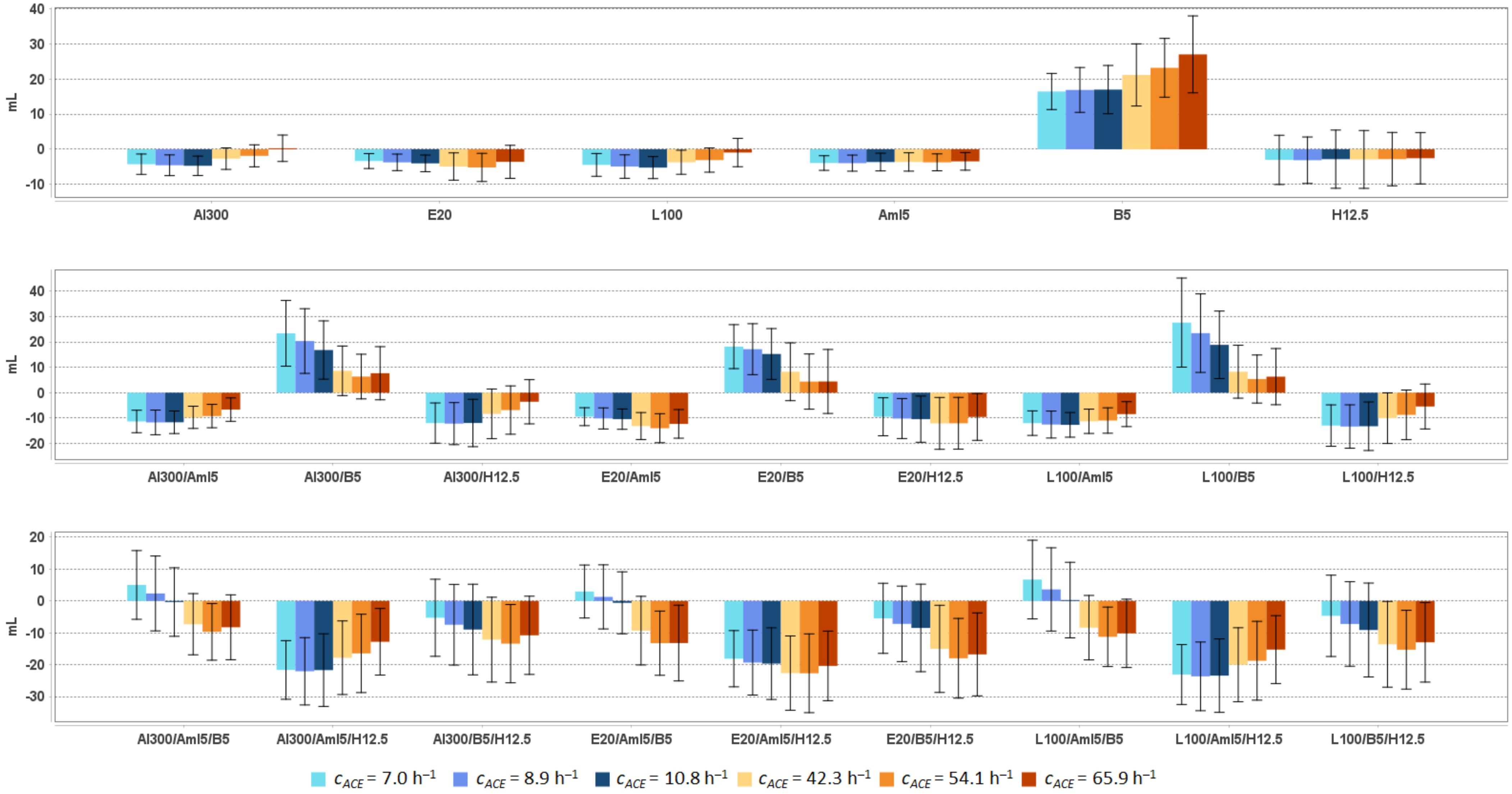

Al300 = aliskiren 300 mg; **Aml5** = amlodipine 5 mg; **B5** = bisoprolol 5 mg; **E20** = enalapril 20 mg; **H12.5** = hydrochlorothiazide 12.5 mg; **L100** = losartan 100 mg

Table S32. Simulated response of right ventricular end-diastolic volume to antihypertensive therapy in virtual hypertensive subpopulations (n = 100) with different ACE activity, including P-values (Kolmogorov-Smirnov test) for endpoint *vs*. baseline; data are presented as mean ± SD in mL

Regimens	***LII*** **(c_{ACE} = 7.0 h^{-1})**			***LID*** **(c_{ACE} = 8.9 h^{-1})**			***LDD*** **(c_{ACE} = 10.8 h^{-1})**			***HII*** **(c_{ACE} = 42.3 h^{-1})**			***HID*** **(c_{ACE} = 54.1 h^{-1})**			***HDD*** **(c_{ACE} = 65.9 h^{-1})**		
	**Value**	**Change**	***P***	**Value**	**Change**	***P***	**Value**	**Change**	***P***	**Value**	**Change**	***P***	**Value**	**Change**	***P***	**Value**	**Change**	***P***
Baseline	116.8 ± 14.7	–	–	118.1 ± 14.0	–	–	117.4 ± 14.9	–	–	117.2 ± 14.3	–	–	119.2 ± 12.4	–	–	117.1 ± 13.1	–	–
Al300	112.5 ± 14.1	-4.2 ± 2.9	0.01008	113.5 ± 13.8	-4.5 ± 3.0	0.00630	112.7 ± 14.8	-4.7 ± 2.8	0.00079	114.5 ± 14.1	-2.7 ± 3.1	0.07832	117.3 ± 12.6	-1.9 ± 3.1	0.36672	117.4 ± 13.2	0.3 ± 3.8	0.81275
E20	113.4 ± 14.2	-3.4 ± 2.1	0.03663	114.3 ± 13.8	-3.7 ± 2.4	0.01008	113.4 ± 14.8	-4.0 ± 2.4	0.00630	112.2 ± 14.0	-4.9 ± 3.9	0.00136	114.0 ± 12.5	-5.2 ± 4.0	0.00007	113.5 ± 13.2	-3.6 ± 4.7	0.01581
L100	112.3 ± 14.1	-4.4 ± 3.2	0.00630	113.1 ± 13.9	-4.9 ± 3.3	0.00630	112.2 ± 14.9	-5.2 ± 3.1	0.00045	113.5 ± 14.0	-3.7 ± 3.4	0.01581	116.1 ± 12.5	-3.1 ± 3.4	0.07832	116.2 ± 13.2	-0.9 ± 4.0	0.46756
Aml5	112.9 ± 14.3	-3.9 ± 2.1	0.03663	114.1 ± 13.6	-3.9 ± 2.3	0.00630	113.8 ± 14.6	-3.6 ± 2.5	0.01008	113.5 ± 14.2	-3.6 ± 2.6	0.01581	115.5 ± 12.7	-3.7 ± 2.4	0.00386	113.6 ± 13.3	-3.4 ± 2.5	0.01581
B5	133.2 ± 17.2	16.5 ± 5.1	SS	135.0 ± 17.5	16.9 ± 6.4	SS	134.4 ± 19.0	17.0 ± 6.9	SS	138.3 ± 18.8	21.2 ± 8.9	SS	142.4 ± 16.4	23.2 ± 8.4	SS	144.1 ± 18.2	27.1 ± 11.0	SS
H12.5	113.8 ± 16.1	-3.0 ± 7.0	0.07832	114.9 ± 14.4	-3.1 ± 6.6	0.03663	114.6 ± 17.5	-2.8 ± 8.3	0.03663	114.3 ± 15.6	-2.9 ± 8.2	0.03663	116.4 ± 15.2	-2.8 ± 7.6	0.11113	114.5 ± 14.1	-2.5 ± 7.3	0.11113
Al300<br>Aml5	105.4 ± 13.7	-11.3 ± 4.4	SS	106.3 ± 13.6	-11.7 ± 4.9	SS	105.8 ± 14.5	-11.7 ± 4.4	SS	107.4 ± 14.0	-9.7 ± 4.4	SS	110.0 ± 13.0	-9.2 ± 4.6	SS	110.4 ± 13.4	-6.7 ± 4.6	0.00079
Al300<br>B5	140.2 ± 20.8	23.4 ± 13.0	SS	138.4 ± 20.8	20.4 ± 12.8	SS	134.3 ± 20.7	16.8 ± 11.5	SS	125.8 ± 17.2	8.6 ± 9.8	0.00630	125.5 ± 14.7	6.4 ± 8.8	0.01008	124.8 ± 16.4	7.7 ± 10.5	0.00045
Al300<br>H12.5	104.8 ± 15.4	-12.0 ± 7.9	SS	105.9 ± 14.5	-12.2 ± 8.3	SS	105.5 ± 17.5	-12.0 ± 9.3	0.00002	108.8 ± 16.0	-8.3 ± 9.8	0.00045	112.3 ± 16.4	-6.9 ± 9.5	0.00136	113.5 ± 14.5	-3.6 ± 8.7	0.03663
E20<br>Aml5	107.3 ± 13.7	-9.5 ± 3.5	SS	107.9 ± 13.5	-10.2 ± 4.2	SS	107.0 ± 14.4	-10.4 ± 4.0	SS	104.0 ± 14.0	-13.2 ± 5.3	SS	105.2 ± 13.1	-14.0 ± 5.7	SS	104.8 ± 13.6	-12.3 ± 5.7	SS
E20<br>B5	134.9 ± 18.1	18.2 ± 8.7	SS	135.2 ± 18.9	17.2 ± 10.1	SS	132.7 ± 19.8	15.3 ± 10.0	SS	125.4 ± 18.0	8.3 ± 11.4	0.01008	123.6 ± 15.7	4.4 ± 10.9	0.01581	121.5 ± 17.7	4.4 ± 12.6	0.01581
E20<br>H12.5	107.3 ± 15.5	-9.5 ± 7.5	0.00007	107.9 ± 14.4	-10.2 ± 7.9	SS	107.0 ± 17.4	-10.4 ± 9.1	0.00002	105.0 ± 16.1	-12.1 ± 10.2	SS	107.1 ± 16.5	-12.1 ± 10.2	SS	107.5 ± 14.7	-9.6 ± 9.2	SS
L100<br>Aml5	104.8 ± 13.7	-12.0 ± 4.8	SS	105.5 ± 13.7	-12.6 ± 5.3	SS	104.7 ± 14.5	-12.7 ± 4.9	SS	105.9 ± 14.0	-11.3 ± 4.8	SS	108.2 ± 13.1	-11.0 ± 5.0	SS	108.6 ± 13.5	-8.5 ± 4.9	SS
L100<br>B5	144.4 ± 24.3	27.7 ± 17.6	SS	141.6 ± 22.9	23.5 ± 15.5	SS	136.3 ± 21.9	18.9 ± 13.3	SS	125.4 ± 17.5	8.3 ± 10.4	0.00630	124.6 ± 15.0	5.4 ± 9.5	0.01008	123.4 ± 16.7	6.3 ± 11.1	0.00136
L100<br>H12.5	103.8 ± 15.4	-13.0 ± 8.2	SS	104.7 ± 14.6	-13.3 ± 8.6	SS	104.2 ± 17.5	-13.2 ± 9.6	SS	107.1 ± 16.0	-10.0 ± 10.0	SS	110.4 ± 16.4	-8.7 ± 9.8	0.00002	111.6 ± 14.5	-5.4 ± 8.9	0.00079
Al300<br>Aml5/B5	121.8 ± 17.3	5.0 ± 10.8	0.03663	120.4 ± 18.2	2.4 ± 11.7	0.07832	117.1 ± 18.2	-0.3 ± 10.8	0.21055	109.9 ± 16.2	-7.3 ± 9.6	0.00025	109.5 ± 14.6	-9.7 ± 8.9	0.00002	108.8 ± 16.0	-8.2 ± 10.2	0.00004
Al300<br>Aml5/H12.5	95.1 ± 15.5	-21.6 ± 9.2	SS	96.0 ± 15.5	-22.1 ± 10.6	SS	95.7 ± 18.1	-21.7 ± 11.4	SS	99.4 ± 17.4	-17.8 ± 11.6	SS	102.7 ± 18.2	-16.4 ± 12.3	SS	104.3 ± 15.8	-12.8 ± 10.5	SS
Al300<br>B5/H12.5	111.5 ± 18.0	-5.3 ± 12.1	0.00136	110.6 ± 17.6	-7.5 ± 12.7	0.00136	108.5 ± 21.0	-9.0 ± 14.2	0.00136	105.0 ± 18.1	-12.1 ± 13.3	SS	105.8 ± 17.5	-13.4 ± 12.3	SS	106.3 ± 16.7	-10.8 ± 12.3	SS
E20<br>Aml5/B5	119.7 ± 16.1	3.0 ± 8.3	0.11113	119.3 ± 17.1	1.3 ± 10.1	0.15454	116.8 ± 17.7	-0.6 ± 9.7	0.28093	107.9 ± 16.6	-9.3 ± 10.8	SS	105.9 ± 15.0	-13.2 ± 10.1	SS	103.9 ± 17.0	-13.2 ± 11.9	SS
E20<br>Aml5/H12.5	98.7 ± 15.7	-18.1 ± 8.8	SS	98.7 ± 15.3	-19.3 ± 10.2	SS	97.8 ± 18.1	-19.7 ± 11.2	SS	94.5 ± 17.2	-22.6 ± 11.7	SS	96.5 ± 17.9	-22.7 ± 12.4	SS	96.7 ± 16.1	-20.4 ± 10.9	SS
E20<br>B5/H12.5	111.3 ± 17.4	-5.4 ± 11.0	0.00079	110.9 ± 17.0	-7.2 ± 11.8	0.00232	109.0 ± 20.7	-8.5 ± 13.7	0.00136	102.1 ± 18.2	-15.0 ± 13.7	SS	101.2 ± 17.3	-18.0 ± 12.5	SS	100.3 ± 17.1	-16.8 ± 13.0	SS
L100<br>Aml5/B5	123.5 ± 18.2	6.7 ± 12.3	0.01008	121.7 ± 19.0	3.6 ± 13.1	0.05410	117.7 ± 18.8	0.3 ± 11.8	0.15454	108.8 ± 16.4	-8.4 ± 10.1	0.00002	107.9 ± 14.7	-11.2 ± 9.3	SS	106.9 ± 16.3	-10.2 ± 10.7	SS
L100<br>Aml5/H12.5	93.7 ± 15.5	-23.1 ± 9.4	SS	94.4 ± 15.6	-23.6 ± 10.8	SS	94.0 ± 18.1	-23.4 ± 11.5	SS	97.2 ± 17.3	-20.0 ± 11.6	SS	100.4 ± 18.1	-18.8 ± 12.4	SS	101.8 ± 16.0	-15.3 ± 10.7	SS
L100<br>B5/H12.5	112.1 ± 18.4	-4.7 ± 12.8	0.00136	110.8 ± 18.0	-7.2 ± 13.3	0.00232	108.3 ± 21.3	-9.1 ± 14.7	0.00079	103.6 ± 18.1	-13.6 ± 13.5	SS	103.9 ± 17.4	-15.3 ± 12.4	SS	104.1 ± 16.8	-13.0 ± 12.5	SS

Al300 = aliskiren 300 mg; **Aml5** = amlodipine 5 mg; **B5** = bisoprolol 5 mg; **E20** = enalapril 20 mg; **H12.5** = hydrochlorothiazide 12.5 mg; **L100** = losartan 100 mg; **SS** = statistically significant (P < 0.00001)

Table S33. P-values calculated using the Kolmogorov-Smirnov test for changes in right ventricular end-diastolic volume in subpopulations (n = 100) with different ACE activity receiving the same regimens. Case 1: c_{ACE} = 7.0 h^{-1} (*LII*), case 2: c_{ACE} = 8.9 h^{-1} (*LID*), case 3: c_{ACE} = 10.8 h^{-1} (*LDD*), case 4: c_{ACE} = 42.3 h^{-1} (*HII*), case 5: c_{ACE} = 54.1 h^{-1} (*HID*), case 6: c_{ACE} = 65.9 h^{-1} (*HDD*). P-value for case i *vs*. case j is denoted P_{ij}.

Regimens	P_{12}	P_{13}	P_{23}	P_{14}	P_{15}	P_{16}	P_{24}	P_{25}	P_{26}	P_{34}	P_{35}	P_{36}	P_{45}	P_{46}	P_{56}
Al300	0.36672	0.02431	0.36672	0.01581	0.00002	SS	0.00232	SS	SS	0.00007	SS	SS	0.05410	SS	0.00630
E20	0.28093	0.00630	0.15454	0.00013	0.00025	0.00630	0.00136	0.00136	0.02431	0.00630	0.00136	0.02431	0.36672	0.21055	0.07832
L100	0.28093	0.01008	0.21055	0.21055	0.03663	SS	0.15454	0.01581	SS	0.00630	SS	SS	0.21055	0.00025	0.00630
Aml5	0.28093	0.28093	0.81275	0.58062	0.21055	0.15454	0.81275	0.90621	0.58062	0.81275	0.99376	0.36672	0.90621	0.81275	0.69937
B5	0.90621	0.69937	0.96707	0.00079	SS	SS	0.01581	SS	SS	0.00232	SS	SS	0.05410	0.00045	0.02431
H12.5	0.69937	0.58062	0.46756	0.69937	0.69937	0.81275	0.36672	0.15454	0.21055	0.90621	0.36672	0.58062	0.81275	0.81275	0.96707
Al300 Aml5	0.90621	0.81275	0.90621	0.21055	0.00232	SS	0.02431	0.00007	SS	0.02431	0.00045	SS	0.15454	0.00013	0.01581
Al300 B5	0.15454	0.00386	0.15454	SS	SS	SS	SS	SS	SS	0.00007	SS	0.00004	0.36672	0.69937	0.58062
Al300 H12.5	0.90621	0.58062	0.46756	0.02431	0.00007	SS	0.01008	0.00002	SS	0.05410	0.00045	SS	0.28093	0.00232	0.11113
E20 Aml5	0.58062	0.15454	0.46756	SS	SS	0.00004	SS	0.00002	0.00013	0.00136	0.00013	0.01581	0.69937	0.69937	0.21055
E20 B5	0.46756	0.02431	0.36672	SS	SS	SS	SS	SS	SS	0.00013	SS	SS	0.11113	0.11113	0.90621
E20 H12.5	0.69937	0.28093	0.36672	0.05410	0.07832	0.46756	0.15454	0.21055	0.81275	0.69937	0.46756	0.28093	0.90621	0.21055	0.46756
L100 Aml5	0.36672	0.46756	0.69937	0.69937	0.21055	0.00136	0.28093	0.01008	SS	0.11113	0.05410	SS	0.58062	0.00232	0.05410
L100 B5	0.21055	0.00232	0.11113	SS	SS	SS	SS	SS	SS	0.00002	SS	SS	0.21055	0.36672	0.69937
L100 H12.5	0.69937	0.81275	0.69937	0.05410	0.00025	SS	0.02431	0.00045	SS	0.11113	0.00386	SS	0.28093	0.00386	0.15454
Al300 Aml5/B5	0.07832	0.01008	0.21055	SS	SS	SS	SS	SS	SS	0.00079	SS	0.00002	0.11113	0.58062	0.69937
Al300 Aml5/H12.5	0.58062	0.58062	0.96707	0.01008	0.00025	SS	0.01008	0.00025	SS	0.05410	0.00232	SS	0.28093	0.05410	0.21055
Al300 B5/H12.5	0.58062	0.01581	0.21055	0.00630	0.00232	0.00232	0.11113	0.02431	0.11113	0.28093	0.05410	0.28093	0.81275	0.81275	0.58062
E20 Aml5/B5	0.15454	0.01581	0.36672	SS	SS	SS	SS	SS	SS	SS	SS	SS	0.05410	0.01581	0.36672
E20 Aml5/H12.5	0.28093	0.21055	0.96707	0.00630	0.00630	0.05410	0.05410	0.11113	0.58062	0.07832	0.28093	0.69937	0.69937	0.46756	0.58062
E20 B5/H12.5	0.28093	0.00630	0.36672	SS	SS	SS	0.00045	0.00002	SS	0.01008	0.00025	0.00232	0.58062	0.36672	0.69937
L100 Aml5/B5	0.07832	0.00386	0.21055	SS	SS	SS	SS	SS	SS	0.00007	SS	SS	0.07832	0.36672	0.81275
L100 Aml5/H12.5	0.58062	0.46756	0.99376	0.03663	0.00079	SS	0.02431	0.00079	SS	0.11113	0.00630	0.00007	0.36672	0.03663	0.36672
L100 B5/H12.5	0.15454	0.01581	0.28093	0.00013	0.00004	0.00013	0.01581	0.00232	0.01008	0.07832	0.01008	0.05410	0.69937	0.96707	0.46756

Al300 = aliskiren 300 mg; **Aml5** = amlodipine 5 mg; **B5** = bisoprolol 5 mg; **E20** = enalapril 20 mg; **H12.5** = hydrochlorothiazide 12.5 mg; **L100** = losartan 100 mg; **SS** = statistically significant ($P < 0.00001$)

Figure S24. Simulated change in right ventricular end-systolic volume from baseline to week 4 (mean ± SD, n = 100)

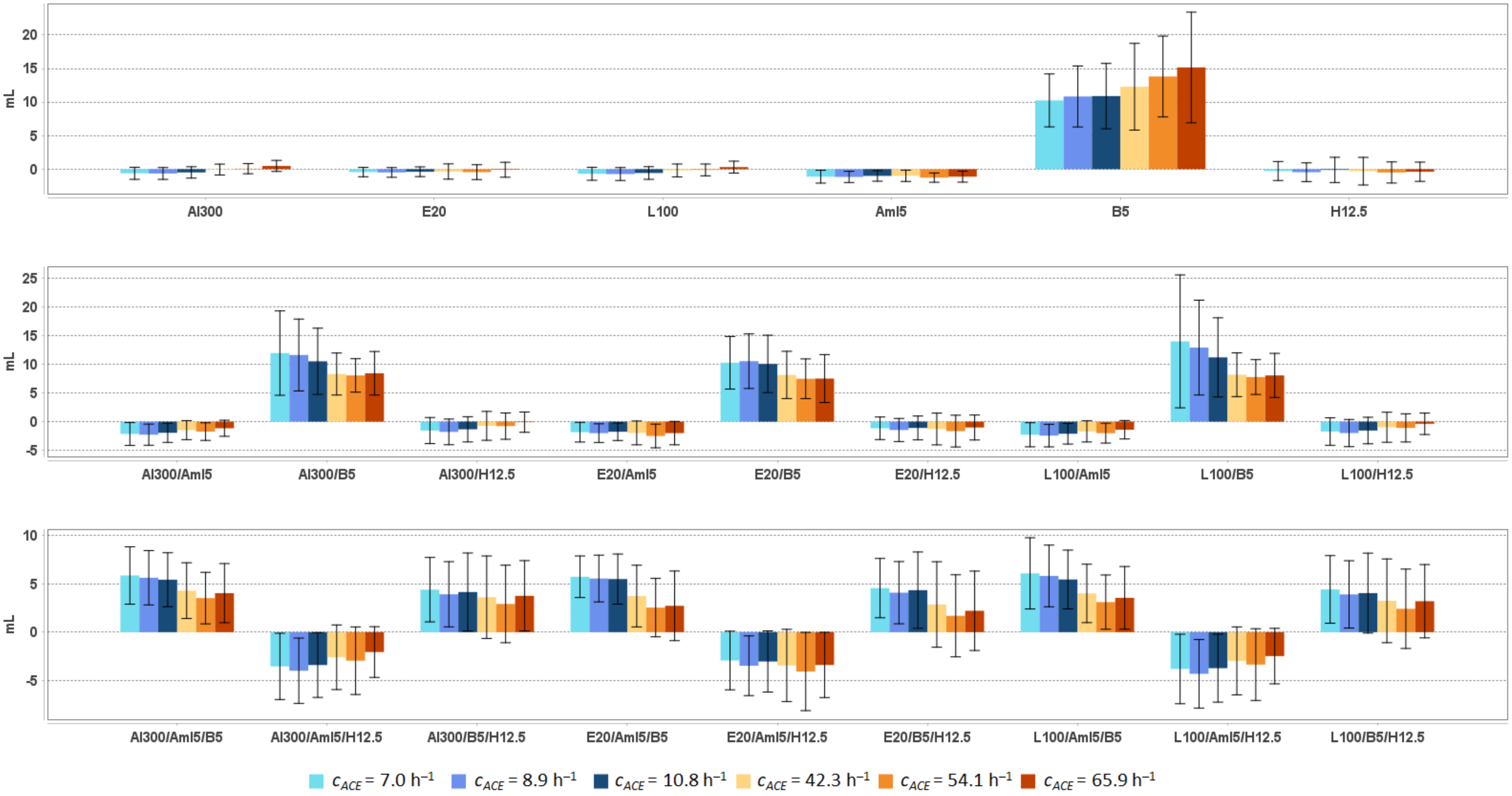

Al300 = aliskiren 300 mg; **Aml5** = amlodipine 5 mg; **B5** = bisoprolol 5 mg; **E20** = enalapril 20 mg; **H12.5** = hydrochlorothiazide 12.5 mg; **L100** = losartan 100 mg

Table S34. Simulated response of right ventricular end-systolic volume to antihypertensive therapy in virtual hypertensive subpopulations (n = 100) with different ACE activity, including P-values (Kolmogorov-Smirnov test) for endpoint *vs.* baseline; data are presented as mean ± SD in mL

Regimens	***LII*** **(c_{ACE} = 7.0 h^{-1})**			***LID*** **(c_{ACE} = 8.9 h^{-1})**			***LDD*** **(c_{ACE} = 10.8 h^{-1})**			***HII*** **(c_{ACE} = 42.3 h^{-1})**			***HID*** **(c_{ACE} = 54.1 h^{-1})**			***HDD*** **(c_{ACE} = 65.9 h^{-1})**		
	**Value**	**Change**	***P***	**Value**	**Change**	***P***	**Value**	**Change**	***P***	**Value**	**Change**	***P***	**Value**	**Change**	***P***	**Value**	**Change**	***P***
Baseline	43.5 ± 12.2	–	–	45.5 ± 10.5	–	–	43.9 ± 11.4	–	–	44.4 ± 11.5	–	–	45.7 ± 9.7	–	–	45.2 ± 11.4	–	–
Al300	42.9 ± 11.8	-0.6 ± 0.9	0.96707	44.9 ± 10.1	-0.6 ± 0.9	0.96707	43.4 ± 11.0	-0.5 ± 0.8	0.99376	44.3 ± 11.3	-0.1 ± 0.8	1.00000	45.8 ± 9.7	0.1 ± 0.8	0.90621	45.7 ± 11.6	0.5 ± 0.8	0.99963
E20	43.1 ± 11.9	-0.4 ± 0.7	0.99376	45.0 ± 10.2	-0.5 ± 0.7	0.96707	43.5 ± 11.1	-0.4 ± 0.7	0.99963	44.0 ± 11.1	-0.4 ± 1.1	0.99376	45.3 ± 9.6	-0.4 ± 1.1	0.96707	45.1 ± 11.3	-0.1 ± 1.1	0.99963
L100	42.8 ± 11.8	-0.7 ± 1.0	0.96707	44.8 ± 10.1	-0.7 ± 1.0	0.90621	43.3 ± 11.0	-0.6 ± 0.9	0.99376	44.2 ± 11.2	-0.2 ± 1.0	0.99963	45.6 ± 9.6	-0.1 ± 0.9	0.99376	45.5 ± 11.5	0.3 ± 0.9	0.99963
Aml5	42.4 ± 11.7	-1.1 ± 1.0	0.69937	44.4 ± 10.1	-1.2 ± 0.8	0.81275	42.9 ± 11.0	-1.0 ± 0.8	0.96707	43.4 ± 11.0	-1.0 ± 0.8	0.90621	44.5 ± 9.7	-1.3 ± 0.7	0.81275	44.1 ± 11.2	-1.1 ± 0.8	0.81275
B5	53.8 ± 14.8	10.2 ± 3.9	SS	56.4 ± 13.2	10.8 ± 4.5	SS	54.8 ± 14.5	10.9 ± 4.9	SS	56.6 ± 15.2	12.3 ± 6.5	SS	59.6 ± 11.9	13.8 ± 6.0	SS	60.4 ± 16.0	15.2 ± 8.2	SS
H12.5	43.3 ± 12.0	-0.3 ± 1.4	0.99963	45.1 ± 10.2	-0.5 ± 1.4	0.99376	43.8 ± 11.6	-0.1 ± 1.9	0.99376	44.1 ± 11.1	-0.3 ± 2.1	0.90621	45.2 ± 9.8	-0.5 ± 1.6	0.99376	44.9 ± 11.3	-0.4 ± 1.4	0.90621
Al300<br>Aml5	41.4 ± 11.2	-2.2 ± 2.0	0.36672	43.3 ± 9.6	-2.3 ± 1.9	0.28093	42.0 ± 10.5	-2.0 ± 1.7	0.46756	42.9 ± 10.7	-1.5 ± 1.7	0.36672	44.0 ± 9.7	-1.7 ± 1.5	0.36672	44.1 ± 11.1	-1.2 ± 1.4	0.69937
Al300<br>B5	55.5 ± 16.4	12.0 ± 7.4	SS	57.2 ± 14.1	11.7 ± 6.3	SS	54.5 ± 14.6	10.6 ± 5.8	SS	52.7 ± 12.7	8.3 ± 3.7	0.00013	53.8 ± 9.9	8.1 ± 2.9	SS	53.7 ± 12.7	8.5 ± 3.8	0.00013
Al300<br>H12.5	42.0 ± 11.4	-1.6 ± 2.3	0.46756	43.7 ± 9.7	-1.8 ± 2.2	0.58062	42.6 ± 11.0	-1.3 ± 2.2	0.81275	43.6 ± 10.8	-0.7 ± 2.5	0.81275	45.0 ± 10.0	-0.8 ± 2.3	0.81275	45.1 ± 11.3	-0.1 ± 1.8	0.90621
E20<br>Aml5	41.7 ± 11.3	-1.8 ± 1.7	0.36672	43.5 ± 9.7	-2.0 ± 1.6	0.36672	42.2 ± 10.6	-1.8 ± 1.5	0.46756	42.4 ± 10.5	-2.0 ± 2.1	0.15454	43.2 ± 9.6	-2.5 ± 2.1	0.28093	43.2 ± 10.9	-2.0 ± 2.0	0.28093
E20<br>B5	53.8 ± 14.9	10.3 ± 4.6	SS	56.1 ± 13.1	10.6 ± 4.8	SS	54.0 ± 14.1	10.1 ± 5.0	SS	52.5 ± 12.8	8.2 ± 4.1	0.00079	53.2 ± 9.8	7.5 ± 3.5	SS	52.8 ± 12.6	7.5 ± 4.2	0.00136
E20<br>H12.5	42.4 ± 11.6	-1.2 ± 2.0	0.69937	44.1 ± 9.8	-1.5 ± 2.0	0.58062	42.8 ± 11.1	-1.1 ± 2.1	0.90621	43.1 ± 10.6	-1.3 ± 2.8	0.36672	44.1 ± 10.0	-1.7 ± 2.8	0.46756	44.2 ± 11.1	-1.0 ± 2.2	0.69937
L100<br>Aml5	41.2 ± 11.1	-2.3 ± 2.1	0.28093	43.1 ± 9.6	-2.4 ± 2.0	0.21055	41.8 ± 10.5	-2.1 ± 1.8	0.46756	42.7 ± 10.6	-1.7 ± 1.9	0.15454	43.7 ± 9.7	-2.0 ± 1.7	0.36672	43.8 ± 11.0	-1.4 ± 1.6	0.58062
L100<br>B5	57.6 ± 19.2	14.0 ± 11.6	SS	58.5 ± 15.4	12.9 ± 8.3	SS	55.2 ± 15.3	11.2 ± 6.9	SS	52.6 ± 12.7	8.2 ± 3.8	0.00079	53.5 ± 9.8	7.8 ± 3.0	SS	53.3 ± 12.6	8.1 ± 3.9	0.00013
L100<br>H12.5	41.8 ± 11.4	-1.7 ± 2.4	0.36672	43.5 ± 9.6	-2.0 ± 2.4	0.36672	42.4 ± 11.0	-1.6 ± 2.3	0.69937	43.4 ± 10.7	-1.0 ± 2.6	0.69937	44.6 ± 10.0	-1.1 ± 2.5	0.69937	44.9 ± 11.2	-0.4 ± 1.9	0.81275
Al300<br>Aml5/B5	49.4 ± 12.7	5.9 ± 3.0	0.00079	51.2 ± 10.7	5.6 ± 2.8	0.01581	49.3 ± 11.7	5.4 ± 2.8	0.00136	48.7 ± 11.1	4.3 ± 2.9	0.05410	49.3 ± 9.6	3.5 ± 2.7	0.01008	49.3 ± 11.5	4.0 ± 3.1	0.11113
Al300<br>Aml5/H12.5	40.0 ± 10.9	-3.5 ± 3.4	0.03663	41.5 ± 9.3	-4.0 ± 3.4	0.02431	40.5 ± 10.7	-3.4 ± 3.3	0.07832	41.8 ± 10.6	-2.6 ± 3.3	0.05410	42.8 ± 10.4	-3.0 ± 3.5	0.28093	43.2 ± 10.9	-2.1 ± 2.6	0.28093
Al300<br>B5/H12.5	47.9 ± 12.1	4.4 ± 3.3	0.02431	49.5 ± 10.1	3.9 ± 3.4	0.05410	48.1 ± 12.0	4.1 ± 4.0	0.07832	48.0 ± 11.1	3.6 ± 4.3	0.15454	48.7 ± 10.3	2.9 ± 4.0	0.00630	49.0 ± 11.4	3.8 ± 3.6	0.07832
E20<br>Aml5/B5	49.3 ± 12.4	5.7 ± 2.2	0.00136	51.1 ± 10.6	5.5 ± 2.4	0.01581	49.4 ± 11.6	5.5 ± 2.6	0.00136	48.1 ± 11.0	3.7 ± 3.2	0.07832	48.3 ± 9.6	2.5 ± 3.0	0.05410	48.0 ± 11.5	2.7 ± 3.6	0.36672
E20<br>Aml5/H12.5	40.6 ± 11.1	-2.9 ± 3.0	0.21055	42.1 ± 9.4	-3.5 ± 3.1	0.07832	40.9 ± 10.7	-3.0 ± 3.2	0.11113	40.9 ± 10.4	-3.4 ± 3.7	0.02431	41.7 ± 10.4	-4.1 ± 4.0	0.15454	41.8 ± 10.8	-3.4 ± 3.4	0.07832
E20<br>B5/H12.5	48.1 ± 12.1	4.6 ± 3.1	0.01008	49.6 ± 10.1	4.1 ± 3.2	0.03663	48.3 ± 12.0	4.3 ± 3.9	0.05410	47.2 ± 11.0	2.9 ± 4.4	0.36672	47.4 ± 10.2	1.7 ± 4.2	0.05410	47.4 ± 11.4	2.2 ± 4.1	0.46756
L100<br>Aml5/B5	49.6 ± 13.0	6.1 ± 3.7	0.00136	51.3 ± 10.9	5.8 ± 3.2	0.01581	49.4 ± 11.7	5.4 ± 3.0	0.00136	48.4 ± 11.1	4.0 ± 3.0	0.05410	48.8 ± 9.6	3.1 ± 2.8	0.02431	48.8 ± 11.5	3.5 ± 3.2	0.15454
L100<br>Aml5/H12.5	39.7 ± 10.9	-3.8 ± 3.6	0.01581	41.2 ± 9.3	-4.3 ± 3.5	0.01581	40.2 ± 10.6	-3.7 ± 3.5	0.07832	41.4 ± 10.5	-3.0 ± 3.5	0.03663	42.4 ± 10.4	-3.4 ± 3.7	0.28093	42.8 ± 10.9	-2.5 ± 2.9	0.21055
L100<br>B5/H12.5	48.0 ± 12.2	4.4 ± 3.5	0.01581	49.4 ± 10.1	3.9 ± 3.5	0.05410	48.0 ± 12.0	4.0 ± 4.1	0.11113	47.6 ± 11.0	3.2 ± 4.3	0.28093	48.2 ± 10.3	2.4 ± 4.1	0.01581	48.4 ± 11.4	3.2 ± 3.8	0.21055

Al300 = aliskiren 300 mg; **Aml5** = amlodipine 5 mg; **B5** = bisoprolol 5 mg; **E20** = enalapril 20 mg; **H12.5** = hydrochlorothiazide 12.5 mg; **L100** = losartan 100 mg; **SS** = statistically significant (P < 0.00001)

Table S35. *P*-values calculated using the Kolmogorov-Smirnov test for changes in <u>right ventricular end-systolic volume</u> in subpopulations (n = 100) with different ACE activity receiving the same regimens. Case 1: c_{ACE} = 7.0 h^{-1} (*LII*), case 2: c_{ACE} = 8.9 h^{-1} (*LID*), case 3: c_{ACE} = 10.8 h^{-1} (*LDD*), case 4: c_{ACE} = 42.3 h^{-1} (*HII*), case 5: c_{ACE} = 54.1 h^{-1} (*HID*), case 6: c_{ACE} = 65.9 h^{-1} (*HDD*). *P*-value for case *i vs*. case *j* is denoted P_{ij}.

Regimens	P_{12}	P_{13}	P_{23}	P_{14}	P_{15}	P_{16}	P_{24}	P_{25}	P_{26}	P_{34}	P_{35}	P_{36}	P_{45}	P_{46}	P_{56}
Al300	0.90621	0.36672	0.46756	SS	SS	SS	SS	SS	SS	SS	SS	SS	0.15454	SS	0.01581
E20	0.81275	0.28093	0.46756	0.00002	0.00045	SS	0.00002	0.00045	SS	0.00232	0.02431	0.00002	0.46756	0.01581	0.02431
L100	0.69937	0.36672	0.36672	SS	SS	SS	SS	SS	SS	0.00002	0.00007	SS	0.58062	0.00079	0.02431
Aml5	0.96707	0.96707	0.81275	0.90621	0.07832	0.99376	0.58062	0.28093	0.99376	0.96707	0.11113	0.99376	0.01581	0.81275	0.11113
B5	0.58062	0.69937	0.81275	0.00630	0.00013	0.00004	0.01581	0.00386	0.00013	0.11113	0.00079	0.00013	0.07832	0.05410	0.69937
H12.5	0.28093	0.69937	0.21055	0.81275	0.28093	0.90621	0.15454	0.21055	0.21055	0.81275	0.90621	0.90621	0.81275	0.99963	0.46756
Al300<br>Aml5	0.81275	0.99376	0.46756	0.00232	0.15454	0.00004	0.01581	0.07832	0.00002	0.01581	0.11113	0.00004	0.46756	0.11113	0.00630
Al300<br>B5	0.99376	0.15454	0.21055	0.00025	0.00013	0.00079	0.00013	0.00013	0.00025	0.00386	0.02431	0.11113	0.46756	0.58062	0.90621
Al300<br>H12.5	0.69937	0.28093	0.46756	0.00045	0.00079	SS	0.00013	0.00007	SS	0.01581	0.01581	0.00004	0.58062	0.15454	0.05410
E20<br>Aml5	0.58062	0.90621	0.69937	0.15454	0.05410	0.21055	0.46756	0.15454	0.46756	0.15454	0.07832	0.21055	0.07832	0.96707	0.15454
E20<br>B5	0.99963	0.46756	0.36672	0.00013	SS	0.00002	0.00045	SS	0.00002	0.00136	0.00045	0.00232	0.58062	0.28093	0.99376
E20<br>H12.5	0.46756	0.69937	0.28093	0.21055	0.11113	0.11113	0.02431	0.15454	0.00630	0.81275	0.46756	0.58062	0.69937	0.69937	0.28093
L100<br>Aml5	0.69937	0.96707	0.46756	0.00630	0.36672	0.00079	0.03663	0.28093	0.00079	0.03663	0.15454	0.00136	0.36672	0.28093	0.02431
L100<br>B5	0.99376	0.11113	0.11113	SS	SS	SS	SS	SS	0.00002	0.00232	0.00232	0.02431	0.69937	0.69937	0.96707
L100<br>H12.5	0.46756	0.46756	0.46756	0.00045	0.00232	SS	0.00025	0.00045	SS	0.01008	0.03663	0.00045	0.96707	0.21055	0.07832
Al300<br>Aml5/B5	0.90621	0.58062	0.69937	0.01581	SS	0.00136	0.07832	0.00013	0.00630	0.11113	0.00045	0.02431	0.05410	0.58062	0.36672
Al300<br>Aml5/H12.5	0.46756	0.90621	0.69937	0.01008	0.03663	0.00013	0.01008	0.03663	0.00025	0.11113	0.36672	0.00630	0.58062	0.28093	0.28093
Al300<br>B5/H12.5	0.21055	0.46756	0.99376	0.21055	0.03663	0.28093	0.69937	0.15454	0.90621	0.69937	0.36672	0.81275	0.36672	0.90621	0.58062
E20<br>Aml5/B5	0.69937	0.36672	0.81275	0.00013	SS	SS	0.00386	SS	SS	0.00232	SS	SS	0.00630	0.02431	0.69937
E20<br>Aml5/H12.5	0.21055	0.36672	0.58062	0.15454	0.03663	0.21055	0.36672	0.58062	0.81275	0.46756	0.21055	0.90621	0.28093	0.81275	0.90621
E20<br>B5/H12.5	0.28093	0.36672	0.90621	0.01008	SS	0.00002	0.28093	0.00025	0.00630	0.28093	0.00079	0.00630	0.11113	0.46756	0.90621
L100<br>Aml5/B5	0.99376	0.58062	0.46756	0.02431	SS	0.00025	0.02431	SS	0.00045	0.07832	0.00004	0.00232	0.03663	0.28093	0.36672
L100<br>Aml5/H12.5	0.36672	0.81275	0.58062	0.03663	0.15454	0.00232	0.01581	0.05410	0.00136	0.21055	0.58062	0.01581	0.46756	0.36672	0.36672
L100<br>B5/H12.5	0.36672	0.46756	0.99376	0.15454	0.00136	0.02431	0.46756	0.07832	0.36672	0.69937	0.02431	0.36672	0.21055	0.96707	0.58062

Al300 = aliskiren 300 mg; **Aml5** = amlodipine 5 mg; **B5** = bisoprolol 5 mg; **E20** = enalapril 20 mg; **H12.5** = hydrochlorothiazide 12.5 mg; **L100** = losartan 100 mg; **SS** = statistically significant (P < 0.00001)

Figure S25. Simulated change in glomerular filtration rate from baseline to week 4 (mean ± SD, n = 100)

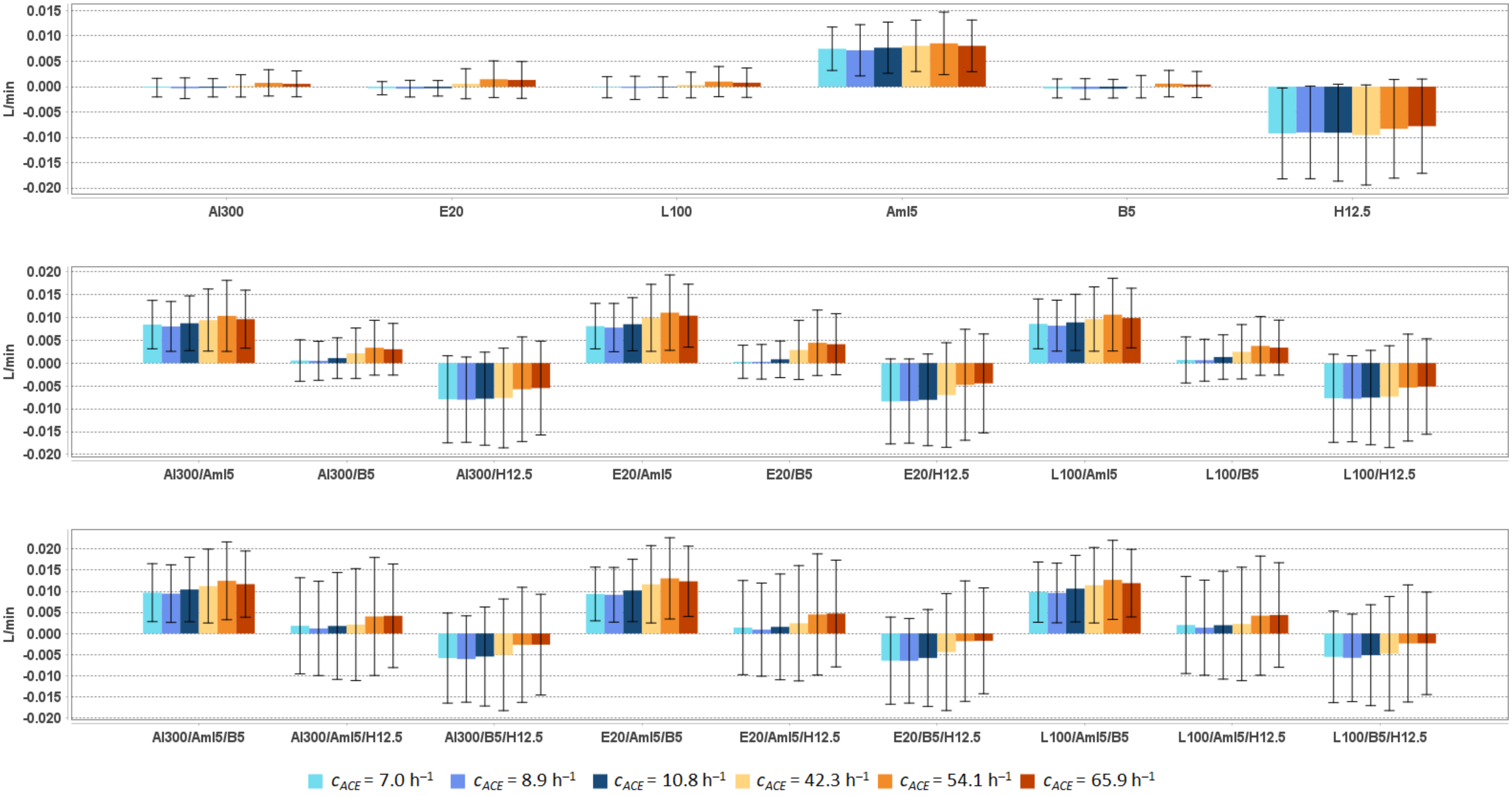

Al300 = aliskiren 300 mg; **Aml5** = amlodipine 5 mg; **B5** = bisoprolol 5 mg; **E20** = enalapril 20 mg; **H12.5** = hydrochlorothiazide 12.5 mg; **L100** = losartan 100 mg

Table S36. Simulated response of glomerular filtration rate to antihypertensive therapy in virtual hypertensive subpopulations (n = 100) with different ACE activity, including P-values (Kolmogorov-Smirnov test) for endpoint *vs*. baseline; data are presented as mean ± SD in L/min

Regimens	*LII* (c_{ACE} = 7.0 h^{-1})			*LID* (c_{ACE} = 8.9 h^{-1})			*LDD* (c_{ACE} = 10.8 h^{-1})			*HII* (c_{ACE} = 42.3 h^{-1})			*HID* (c_{ACE} = 54.1 h^{-1})			*HDD* (c_{ACE} = 65.9 h^{-1})		
	**Value**	**Change**	***P***	**Value**	**Change**	***P***	**Value**	**Change**	***P***	**Value**	**Change**	***P***	**Value**	**Change**	***P***	**Value**	**Change**	***P***
Baseline	0.0945 ± 0.0192	–	–	0.0948 ± 0.0197	–	–	0.0916 ± 0.0197	–	–	0.0922 ± 0.0186	–	–	0.0936 ± 0.0181	–	–	0.0950 ± 0.0196	–	–
Al300	0.0943 ± 0.0197	-0.0002 ± 0.0018	0.99963	0.0945 ± 0.0203	-0.0003 ± 0.0020	1.00000	0.0913 ± 0.0201	-0.0002 ± 0.0018	0.99963	0.0923 ± 0.0188	0.0002 ± 0.0022	0.99963	0.0943 ± 0.0181	0.0007 ± 0.0026	0.96707	0.0955 ± 0.0200	0.0006 ± 0.0026	0.99376
E20	0.0942 ± 0.0196	-0.0003 ± 0.0013	1.00000	0.0944 ± 0.0202	-0.0004 ± 0.0016	1.00000	0.0913 ± 0.0200	-0.0003 ± 0.0015	1.00000	0.0928 ± 0.0189	0.0006 ± 0.0030	0.96707	0.0950 ± 0.0181	0.0015 ± 0.0036	0.96707	0.0963 ± 0.0201	0.0013 ± 0.0036	0.90621
L100	0.0944 ± 0.0198	-0.0001 ± 0.0021	0.96707	0.0946 ± 0.0204	-0.0002 ± 0.0023	1.00000	0.0915 ± 0.0201	-0.0001 ± 0.0021	0.99963	0.0925 ± 0.0188	0.0003 ± 0.0025	0.99963	0.0946 ± 0.0181	0.0010 ± 0.0030	0.96707	0.0957 ± 0.0200	0.0008 ± 0.0029	0.99376
Aml5	0.1020 ± 0.0182	0.0075 ± 0.0043	0.01008	0.1020 ± 0.0185	0.0072 ± 0.0051	0.03663	0.0993 ± 0.0189	0.0077 ± 0.0050	0.00025	0.1002 ± 0.0178	0.0081 ± 0.0051	0.00136	0.1021 ± 0.0165	0.0085 ± 0.0062	0.00045	0.1030 ± 0.0181	0.0080 ± 0.0051	0.00630
B5	0.0941 ± 0.0198	-0.0004 ± 0.0019	0.99963	0.0944 ± 0.0204	-0.0005 ± 0.0020	0.99963	0.0912 ± 0.0201	-0.0004 ± 0.0018	0.99963	0.0922 ± 0.0188	-0.0000 ± 0.0022	1.00000	0.0942 ± 0.0181	0.0006 ± 0.0026	0.99376	0.0954 ± 0.0200	0.0004 ± 0.0026	0.99963
H12.5	0.0853 ± 0.0237	-0.0092 ± 0.0090	0.00136	0.0858 ± 0.0236	-0.0090 ± 0.0091	0.01008	0.0825 ± 0.0239	-0.0091 ± 0.0096	0.00386	0.0827 ± 0.0230	-0.0095 ± 0.0099	0.00232	0.0853 ± 0.0223	-0.0083 ± 0.0097	0.00630	0.0872 ± 0.0239	-0.0078 ± 0.0093	0.00630
Al300 Aml5	0.1029 ± 0.0186	0.0084 ± 0.0053	0.00630	0.1029 ± 0.0187	0.0080 ± 0.0054	0.00630	0.1003 ± 0.0192	0.0087 ± 0.0060	0.00025	0.1016 ± 0.0182	0.0094 ± 0.0068	0.00025	0.1039 ± 0.0166	0.0103 ± 0.0078	SS	0.1046 ± 0.0183	0.0096 ± 0.00630	0.00136
Al300 B5	0.0950 ± 0.0208	0.0006 ± 0.0045	0.81275	0.0953 ± 0.0210	0.0005 ± 0.0043	0.99376	0.0926 ± 0.0206	0.0011 ± 0.0044	0.58062	0.0943 ± 0.0193	0.0021 ± 0.0055	0.46756	0.0969 ± 0.0183	0.0034 ± 0.0060	0.36672	0.0980 ± 0.0204	0.0030 ± 0.0056	0.21055
Al300 H12.5	0.0866 ± 0.0242	-0.0079 ± 0.0095	0.00630	0.0868 ± 0.0241	-0.0080 ± 0.0093	0.02431	0.0838 ± 0.0245	-0.0078 ± 0.0102	0.01008	0.0846 ± 0.0234	-0.0076 ± 0.0109	0.03663	0.0879 ± 0.0227	-0.0057 ± 0.0114	0.03663	0.0895 ± 0.0243	-0.0055 ± 0.0103	0.03663
E20 Aml5	0.1026 ± 0.0185	0.0081 ± 0.0050	0.01008	0.1026 ± 0.0187	0.0078 ± 0.0053	0.01008	0.1001 ± 0.0192	0.0085 ± 0.0058	0.00025	0.1021 ± 0.0183	0.0099 ± 0.0073	0.00013	0.1046 ± 0.0167	0.0110 ± 0.0082	SS	0.1053 ± 0.0183	0.0104 ± 0.0069	0.00013
E20 B5	0.0948 ± 0.0204	0.0003 ± 0.0036	0.99376	0.0951 ± 0.0209	0.0003 ± 0.0038	0.99376	0.0924 ± 0.0205	0.0008 ± 0.0040	0.69937	0.0951 ± 0.0195	0.0029 ± 0.0065	0.28093	0.0980 ± 0.0184	0.0044 ± 0.0072	0.07832	0.0991 ± 0.0205	0.0041 ± 0.0067	0.03663
E20 H12.5	0.0861 ± 0.0240	-0.0084 ± 0.0093	0.00386	0.0865 ± 0.0240	-0.0083 ± 0.0092	0.02431	0.0835 ± 0.0244	-0.0080 ± 0.0100	0.01008	0.0852 ± 0.0237	-0.0070 ± 0.0114	0.05410	0.0888 ± 0.0229	-0.0047 ± 0.0121	0.05410	0.0905 ± 0.0246	-0.0044 ± 0.0108	0.07832
L100 Aml5	0.1030 ± 0.0186	0.0086 ± 0.0054	0.00232	0.1030 ± 0.0187	0.0082 ± 0.0056	0.00386	0.1005 ± 0.0193	0.0089 ± 0.0061	0.00025	0.1018 ± 0.0182	0.0096 ± 0.0070	0.00013	0.1042 ± 0.0166	0.0106 ± 0.0079	SS	0.1048 ± 0.0183	0.0098 ± 0.0065	0.00079
L100 B5	0.0952 ± 0.0210	0.0007 ± 0.0050	0.81275	0.0955 ± 0.0211	0.0006 ± 0.0046	0.99376	0.0929 ± 0.0207	0.0013 ± 0.0049	0.36672	0.0947 ± 0.0194	0.0025 ± 0.0059	0.36672	0.0973 ± 0.0184	0.0037 ± 0.0064	0.15454	0.0983 ± 0.0204	0.0034 ± 0.0060	0.07832
L100 H12.5	0.0868 ± 0.0243	-0.0077 ± 0.0096	0.00630	0.0870 ± 0.0242	-0.0078 ± 0.0094	0.03663	0.0840 ± 0.0246	-0.0075 ± 0.0103	0.01008	0.0848 ± 0.0235	-0.0073 ± 0.0111	0.03663	0.0882 ± 0.0228	-0.0054 ± 0.0117	0.03663	0.0898 ± 0.0244	-0.0051 ± 0.0104	0.07832
Al300 Aml5/B5	0.1042 ± 0.0192	0.0097 ± 0.0069	0.00025	0.1043 ± 0.0190	0.0094 ± 0.0068	0.00232	0.1020 ± 0.0195	0.0104 ± 0.0076	0.00025	0.1034 ± 0.0187	0.0112 ± 0.0088	SS	0.1060 ± 0.0167	0.0125 ± 0.0092	SS	0.1066 ± 0.0182	0.0117 ± 0.0078	SS
Al300 Aml5/H12.5	0.0963 ± 0.0237	0.0018 ± 0.0114	0.15454	0.0960 ± 0.0236	0.0012 ± 0.0112	0.15454	0.0933 ± 0.0249	0.0018 ± 0.0127	0.03663	0.0943 ± 0.0239	0.0021 ± 0.0133	0.11113	0.0976 ± 0.0225	0.0040 ± 0.0140	0.02431	0.0991 ± 0.0236	0.0042 ± 0.0123	0.01008
Al300 B5/H12.5	0.0886 ± 0.0249	-0.0058 ± 0.0107	0.02431	0.0888 ± 0.0248	-0.0061 ± 0.0102	0.15454	0.0861 ± 0.0254	-0.0055 ± 0.0117	0.01008	0.0871 ± 0.0245	-0.0051 ± 0.0132	0.05410	0.0909 ± 0.0235	-0.0027 ± 0.0136	0.21055	0.0923 ± 0.0250	-0.0026 ± 0.0119	0.21055
E20 Aml5/B5	0.1038 ± 0.0190	0.0094 ± 0.0064	0.00079	0.1040 ± 0.0189	0.0092 ± 0.0065	0.00232	0.1018 ± 0.0194	0.0102 ± 0.0074	0.00025	0.1038 ± 0.0188	0.0117 ± 0.0092	SS	0.1066 ± 0.0168	0.0131 ± 0.0096	SS	0.1073 ± 0.0182	0.0124 ± 0.0083	SS
E20 Aml5/H12.5	0.0959 ± 0.0236	0.0014 ± 0.0112	0.21055	0.0957 ± 0.0235	0.0009 ± 0.0110	0.21055	0.0931 ± 0.0248	0.0016 ± 0.0125	0.05410	0.0946 ± 0.0241	0.0024 ± 0.0137	0.07832	0.0981 ± 0.0226	0.0045 ± 0.0144	0.01008	0.0997 ± 0.0237	0.0047 ± 0.0126	0.00630
E20 B5/H12.5	0.0880 ± 0.0247	-0.0064 ± 0.0103	0.02431	0.0884 ± 0.0247	-0.0065 ± 0.0100	0.11113	0.0858 ± 0.0253	-0.0058 ± 0.0115	0.01008	0.0878 ± 0.0248	-0.0044 ± 0.0139	0.05410	0.0917 ± 0.0238	-0.0018 ± 0.0143	0.21055	0.0932 ± 0.0252	-0.0017 ± 0.0125	0.21055
L100 Aml5/B5	0.1043 ± 0.0193	0.0098 ± 0.0071	0.00025	0.1044 ± 0.0190	0.0096 ± 0.0071	0.00232	0.1022 ± 0.0195	0.0106 ± 0.0079	0.00025	0.1036 ± 0.0187	0.0114 ± 0.0089	SS	0.1063 ± 0.0167	0.0127 ± 0.0094	SS	0.1069 ± 0.0182	0.0119 ± 0.0080	SS
L100 Aml5/H12.5	0.0965 ± 0.0238	0.0020 ± 0.0115	0.15454	0.0962 ± 0.0236	0.0014 ± 0.0113	0.15454	0.0935 ± 0.0250	0.0020 ± 0.0128	0.02431	0.0944 ± 0.0240	0.0023 ± 0.0134	0.11113	0.0978 ± 0.0225	0.0042 ± 0.0141	0.01581	0.0993 ± 0.0236	0.0044 ± 0.0124	0.00630
L100 B5/H12.5	0.0889 ± 0.0251	-0.0055 ± 0.0108	0.02431	0.0891 ± 0.0249	-0.0058 ± 0.0104	0.15454	0.0864 ± 0.0255	-0.0051 ± 0.0120	0.01008	0.0874 ± 0.0247	-0.0048 ± 0.0135	0.05410	0.0912 ± 0.0236	-0.0024 ± 0.0139	0.21055	0.0926 ± 0.0251	-0.0023 ± 0.0121	0.21055

Al300 = aliskiren 300 mg; **Aml5** = amlodipine 5 mg; **B5** = bisoprolol 5 mg; **E20** = enalapril 20 mg; **H12.5** = hydrochlorothiazide 12.5 mg; **L100** = losartan 100 mg; **SS** = statistically significant (P < 0.00001)

Table S37. *P*-values calculated using the Kolmogorov-Smirnov test for changes in glomerular filtration rate in subpopulations (n = 100) with different ACE activity receiving the same regimens. Case 1: c_{ACE} = 7.0 h^{-1} (*LII*), case 2: c_{ACE} = 8.9 h^{-1} (*LID*), case 3: c_{ACE} = 10.8 h^{-1} (*LDD*), case 4: c_{ACE} = 42.3 h^{-1} (*HII*), case 5: c_{ACE} = 54.1 h^{-1} (*HID*), case 6: c_{ACE} = 65.9 h^{-1} (*HDD*). *P*-value for case *i vs*. case *j* is denoted P_{ij}.

**Regimens**	P_{12}	P_{13}	P_{23}	P_{14}	P_{15}	P_{16}	P_{24}	P_{25}	P_{26}	P_{34}	P_{35}	P_{36}	P_{45}	P_{46}	P_{56}
Al300	0.99376	0.58062	0.90621	0.58062	0.05410	0.07832	0.58062	0.01581	0.00630	0.28093	0.01581	0.01581	0.21055	0.36672	0.99376
E20	0.90621	0.90621	0.99963	0.00232	SS	SS	0.00386	SS	SS	0.00136	SS	SS	0.11113	0.28093	0.99376
L100	0.90621	0.46756	0.81275	0.46756	0.02431	0.05410	0.46756	0.00630	0.01008	0.28093	0.01008	0.02431	0.21055	0.36672	0.99963
Aml5	0.36672	0.81275	0.81275	0.81275	0.81275	0.46756	0.21055	0.15454	0.00630	0.46756	0.36672	0.05410	0.96707	0.36672	0.69937
B5	0.90621	0.69937	0.99963	0.58062	0.07832	0.05410	0.36672	0.05410	0.01008	0.28093	0.05410	0.02431	0.28093	0.11113	0.96707
H12.5	0.96707	0.46756	0.46756	0.69937	0.36672	0.21055	0.58062	0.36672	0.02431	0.81275	0.21055	0.36672	0.46756	0.15454	0.81275
Al300<br>Aml5	0.46756	0.69937	0.58062	0.81275	0.46756	0.36672	0.21055	0.07832	0.05410	0.28093	0.07832	0.07832	0.69937	0.81275	0.81275
Al300<br>B5	0.69937	0.58062	0.69937	0.21055	0.00630	0.01008	0.21055	0.00232	0.00630	0.58062	0.01581	0.03663	0.28093	0.28093	0.96707
Al300<br>H12.5	0.90621	0.58062	0.69937	0.90621	0.28093	0.11113	0.58062	0.21055	0.03663	0.69937	0.15454	0.15454	0.58062	0.28093	0.99376
E20<br>Aml5	0.46756	0.58062	0.69937	0.46756	0.07832	0.05410	0.03663	0.00136	0.00232	0.11113	0.00630	0.00630	0.69937	0.46756	0.81275
E20<br>B5	0.96707	0.58062	0.58062	0.00630	SS	0.00002	0.00630	SS	SS	0.05410	0.00013	0.00025	0.15454	0.15454	0.99376
E20<br>H12.5	0.90621	0.58062	0.46756	0.46756	0.07832	0.05410	0.28093	0.01581	0.00386	0.46756	0.03663	0.02431	0.46756	0.15454	0.96707
L100<br>Aml5	0.46756	0.69937	0.69937	0.81275	0.46756	0.46756	0.21055	0.05410	0.05410	0.36672	0.07832	0.11113	0.58062	0.81275	0.81275
L100<br>B5	0.46756	0.46756	0.58062	0.15454	0.00630	0.00630	0.15454	0.00136	0.00630	0.46756	0.03663	0.05410	0.28093	0.28093	0.96707
L100<br>H12.5	0.96707	0.58062	0.69937	0.96707	0.36672	0.07832	0.58062	0.15454	0.07832	0.69937	0.21055	0.15454	0.46756	0.21055	0.99376
Al300<br>Aml5/B5	0.58062	0.36672	0.58062	0.58062	0.15454	0.15454	0.28093	0.01581	0.02431	0.36672	0.15454	0.07832	0.28093	0.28093	0.96707
Al300<br>Aml5/H12.5	0.46756	0.36672	0.81275	0.90621	0.69937	0.46756	0.28093	0.07832	0.05410	0.69937	0.36672	0.21055	0.58062	0.36672	0.90621
Al300<br>B5/H12.5	0.69937	0.58062	0.81275	0.69937	0.36672	0.46756	0.28093	0.07832	0.05410	0.46756	0.11113	0.03663	0.36672	0.28093	0.96707
E20<br>Aml5/B5	0.69937	0.46756	0.69937	0.46756	0.03663	0.05410	0.07832	0.00386	0.01008	0.21055	0.01008	0.01008	0.21055	0.21055	0.99376
E20<br>Aml5/H12.5	0.36672	0.46756	0.81275	0.81275	0.36672	0.21055	0.15454	0.02431	0.01008	0.46756	0.28093	0.05410	0.69937	0.46756	0.81275
E20<br>B5/H12.5	0.96707	0.90621	0.81275	0.21055	0.05410	0.07832	0.05410	0.01008	0.00630	0.36672	0.02431	0.01581	0.36672	0.28093	0.81275
L100<br>Aml5/B5	0.69937	0.36672	0.36672	0.36672	0.11113	0.11113	0.28093	0.02431	0.02431	0.36672	0.15454	0.07832	0.28093	0.28093	0.96707
L100<br>Aml5/H12.5	0.46756	0.36672	0.81275	0.96707	0.58062	0.46756	0.28093	0.07832	0.03663	0.81275	0.36672	0.21055	0.69937	0.46756	0.81275
L100<br>B5/H12.5	0.58062	0.69937	0.81275	0.69937	0.28093	0.36672	0.28093	0.07832	0.05410	0.58062	0.07832	0.03663	0.36672	0.28093	0.90621

Al300 = aliskiren 300 mg; **Aml5** = amlodipine 5 mg; **B5** = bisoprolol 5 mg; **E20** = enalapril 20 mg; **H12.5** = hydrochlorothiazide 12.5 mg; **L100** = losartan 100 mg; **SS** = statistically significant (P < 0.00001)

Figure S26. Simulated change in renal blood flow from baseline to week 4 (mean ± SD, n = 100)

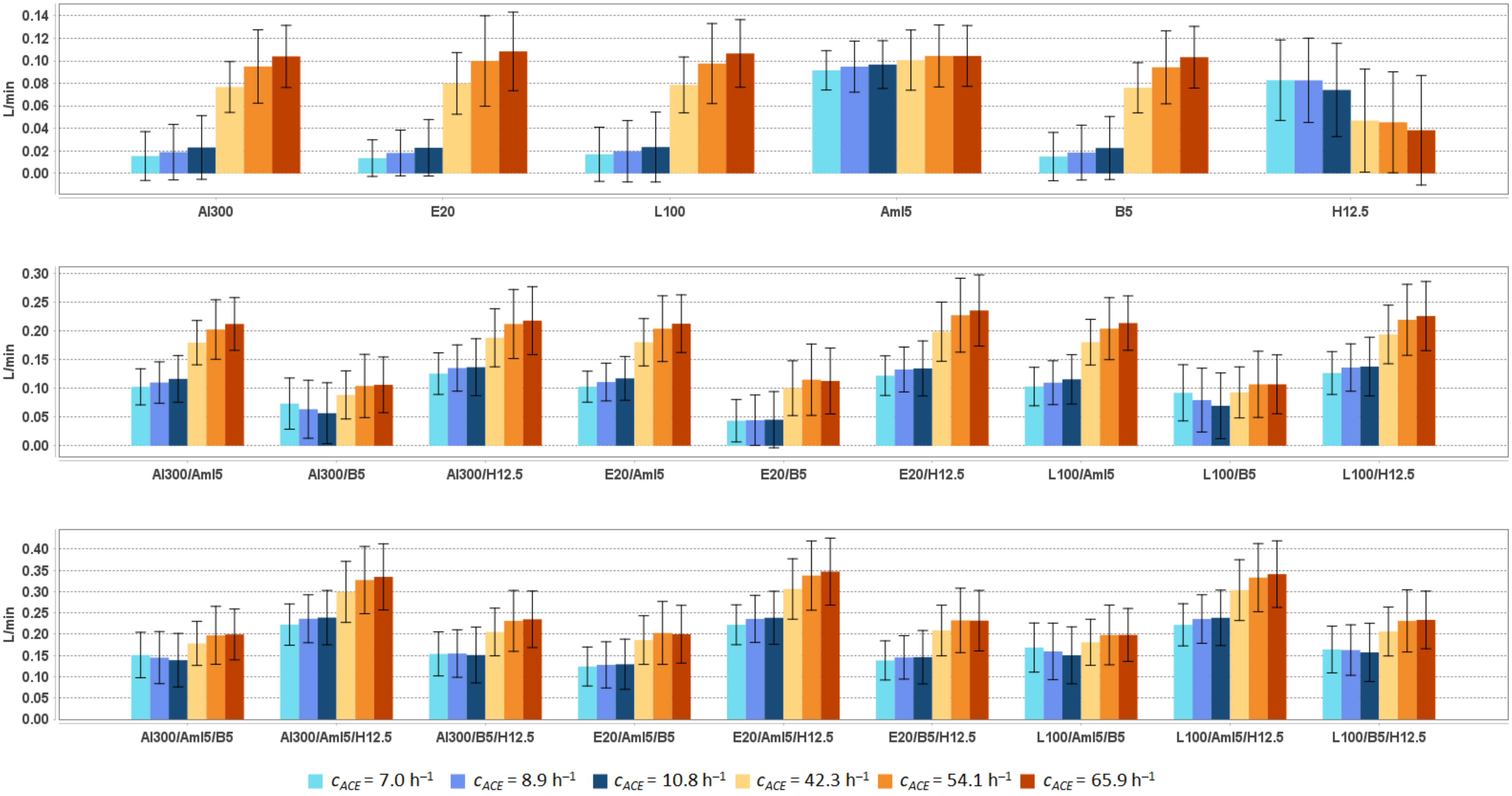

Al300 = aliskiren 300 mg; **Aml5** = amlodipine 5 mg; **B5** = bisoprolol 5 mg; **E20** = enalapril 20 mg; **H12.5** = hydrochlorothiazide 12.5 mg; **L100** = losartan 100 mg

Table S38. Simulated response of <u>renal blood flow</u> to antihypertensive therapy in virtual hypertensive subpopulations (n = 100) with different ACE activity, including P-values (Kolmogorov-Smirnov test) for endpoint *vs*. baseline; data are presented as mean ± SD in L/min

Regimens	*LII* (c_{ACE} = 7.0 h^{-1})			*LID* (c_{ACE} = 8.9 h^{-1})			*LDD* (c_{ACE} = 10.8 h^{-1})			*HII* (c_{ACE} = 42.3 h^{-1})			*HID* (c_{ACE} = 54.1 h^{-1})			*HDD* (c_{ACE} = 65.9 h^{-1})		
	Value	Change	*P*	Value	Change	*P*	Value	Change	*P*	Value	Change	*P*	Value	Change	*P*	Value	Change	*P*
Baseline	1.082 ± 0.147	–	–	1.108 ± 0.168	–	–	1.114 ± 0.161	–	–	1.106 ± 0.153	–	–	1.127 ± 0.152	–	–	1.142 ± 0.164	–	–
Al300	1.098 ± 0.147	0.015 ± 0.022	0.69937	1.127 ± 0.165	0.019 ± 0.025	0.81275	1.137 ± 0.162	0.023 ± 0.028	0.90621	1.183 ± 0.159	0.077 ± 0.023	0.00232	1.222 ± 0.165	0.095 ± 0.033	0.00232	1.246 ± 0.175	0.104 ± 0.028	0.00025
E20	1.096 ± 0.147	0.014 ± 0.016	0.81275	1.126 ± 0.166	0.018 ± 0.020	0.90621	1.137 ± 0.162	0.023 ± 0.025	0.90621	1.186 ± 0.159	0.080 ± 0.027	0.00079	1.227 ± 0.166	0.100 ± 0.040	0.00136	1.251 ± 0.175	0.108 ± 0.035	0.00025
L100	1.099 ± 0.147	0.017 ± 0.024	0.69937	1.128 ± 0.164	0.020 ± 0.027	0.81275	1.138 ± 0.162	0.023 ± 0.031	0.81275	1.185 ± 0.159	0.079 ± 0.025	0.00232	1.225 ± 0.165	0.098 ± 0.036	0.00136	1.249 ± 0.175	0.107 ± 0.030	0.00025
Aml5	1.174 ± 0.156	0.092 ± 0.017	0.00136	1.203 ± 0.181	0.095 ± 0.023	0.00232	1.211 ± 0.171	0.097 ± 0.021	0.00079	1.207 ± 0.166	0.101 ± 0.027	0.00079	1.232 ± 0.166	0.104 ± 0.028	0.00025	1.246 ± 0.177	0.104 ± 0.027	0.00007
B5	1.097 ± 0.147	0.015 ± 0.021	0.69937	1.127 ± 0.165	0.018 ± 0.024	0.81275	1.137 ± 0.162	0.023 ± 0.028	0.90621	1.183 ± 0.159	0.076 ± 0.022	0.00232	1.222 ± 0.165	0.094 ± 0.032	0.00232	1.245 ± 0.175	0.103 ± 0.027	0.00045
H12.5	1.165 ± 0.158	0.083 ± 0.036	0.00232	1.191 ± 0.185	0.083 ± 0.037	0.02431	1.188 ± 0.174	0.074 ± 0.041	0.01581	1.153 ± 0.171	0.047 ± 0.046	0.28093	1.173 ± 0.165	0.045 ± 0.045	0.21055	1.180 ± 0.179	0.038 ± 0.049	0.46756
Al300 Aml5	1.185 ± 0.156	0.102 ± 0.031	0.00004	1.218 ± 0.177	0.110 ± 0.036	0.00025	1.230 ± 0.172	0.116 ± 0.041	0.00013	1.286 ± 0.172	0.179 ± 0.039	SS	1.329 ± 0.179	0.202 ± 0.052	SS	1.354 ± 0.188	0.212 ± 0.046	SS
Al300 B5	1.156 ± 0.155	0.073 ± 0.045	0.00232	1.172 ± 0.168	0.063 ± 0.050	0.03663	1.171 ± 0.164	0.057 ± 0.053	0.11113	1.195 ± 0.158	0.088 ± 0.042	0.00045	1.231 ± 0.166	0.104 ± 0.055	0.00045	1.248 ± 0.174	0.106 ± 0.048	0.00013
Al300 H12.5	1.208 ± 0.159	0.125 ± 0.036	SS	1.243 ± 0.184	0.135 ± 0.040	0.00004	1.251 ± 0.180	0.136 ± 0.049	0.00002	1.294 ± 0.182	0.188 ± 0.050	SS	1.339 ± 0.185	0.212 ± 0.060	SS	1.360 ± 0.197	0.217 ± 0.059	SS
E20 Aml5	1.185 ± 0.156	0.103 ± 0.027	0.00004	1.219 ± 0.178	0.111 ± 0.033	0.00025	1.231 ± 0.172	0.117 ± 0.038	0.00007	1.286 ± 0.170	0.180 ± 0.041	SS	1.331 ± 0.179	0.203 ± 0.057	SS	1.354 ± 0.187	0.212 ± 0.050	SS
E20 B5	1.126 ± 0.151	0.044 ± 0.037	0.11113	1.153 ± 0.166	0.044 ± 0.044	0.15454	1.159 ± 0.163	0.045 ± 0.049	0.28093	1.207 ± 0.159	0.100 ± 0.048	0.00013	1.242 ± 0.167	0.115 ± 0.062	0.00013	1.255 ± 0.176	0.113 ± 0.057	0.00007
E20 H12.5	1.204 ± 0.159	0.122 ± 0.034	SS	1.241 ± 0.185	0.133 ± 0.039	0.00007	1.249 ± 0.180	0.134 ± 0.048	0.00002	1.305 ± 0.181	0.198 ± 0.051	SS	1.354 ± 0.186	0.227 ± 0.064	SS	1.377 ± 0.198	0.235 ± 0.062	SS
L100 Aml5	1.185 ± 0.156	0.103 ± 0.033	0.00002	1.218 ± 0.176	0.110 ± 0.038	0.00013	1.230 ± 0.172	0.115 ± 0.043	0.00013	1.286 ± 0.171	0.180 ± 0.040	SS	1.331 ± 0.179	0.204 ± 0.054	SS	1.355 ± 0.188	0.213 ± 0.047	SS
L100 B5	1.174 ± 0.157	0.092 ± 0.049	0.00013	1.188 ± 0.171	0.079 ± 0.055	0.01008	1.184 ± 0.165	0.069 ± 0.057	0.05410	1.199 ± 0.158	0.093 ± 0.044	0.00025	1.234 ± 0.166	0.107 ± 0.058	0.00045	1.249 ± 0.175	0.107 ± 0.051	0.00013
L100 H12.5	1.209 ± 0.159	0.126 ± 0.037	SS	1.244 ± 0.184	0.136 ± 0.041	0.00004	1.252 ± 0.180	0.138 ± 0.051	0.00002	1.300 ± 0.182	0.193 ± 0.051	SS	1.346 ± 0.185	0.219 ± 0.062	SS	1.368 ± 0.198	0.225 ± 0.060	SS
Al300 Aml5/B5	1.233 ± 0.162	0.151 ± 0.053	SS	1.253 ± 0.178	0.145 ± 0.061	SS	1.253 ± 0.173	0.139 ± 0.063	0.00007	1.285 ± 0.168	0.178 ± 0.052	SS	1.325 ± 0.177	0.197 ± 0.068	SS	1.341 ± 0.184	0.199 ± 0.060	SS
Al300 Aml5/H12.5	1.305 ± 0.170	0.223 ± 0.049	SS	1.344 ± 0.198	0.236 ± 0.056	SS	1.353 ± 0.191	0.239 ± 0.064	SS	1.406 ± 0.198	0.299 ± 0.072	SS	1.455 ± 0.199	0.327 ± 0.079	SS	1.477 ± 0.213	0.335 ± 0.078	SS
Al300 B5/H12.5	1.236 ± 0.162	0.154 ± 0.052	SS	1.263 ± 0.182	0.155 ± 0.056	SS	1.265 ± 0.179	0.151 ± 0.066	SS	1.312 ± 0.178	0.205 ± 0.056	SS	1.359 ± 0.185	0.231 ± 0.072	SS	1.377 ± 0.196	0.235 ± 0.066	SS
E20 Aml5/B5	1.206 ± 0.159	0.124 ± 0.046	SS	1.236 ± 0.176	0.128 ± 0.054	SS	1.243 ± 0.172	0.129 ± 0.059	0.00013	1.293 ± 0.169	0.186 ± 0.057	SS	1.330 ± 0.177	0.203 ± 0.074	SS	1.342 ± 0.185	0.200 ± 0.068	SS
E20 Aml5/H12.5	1.304 ± 0.170	0.222 ± 0.047	SS	1.344 ± 0.200	0.236 ± 0.055	SS	1.353 ± 0.192	0.239 ± 0.062	SS	1.413 ± 0.197	0.306 ± 0.071	SS	1.465 ± 0.200	0.338 ± 0.081	SS	1.489 ± 0.212	0.347 ± 0.079	SS
E20 B5/H12.5	1.221 ± 0.160	0.138 ± 0.046	SS	1.254 ± 0.181	0.145 ± 0.051	SS	1.260 ± 0.179	0.146 ± 0.063	SS	1.315 ± 0.178	0.209 ± 0.059	SS	1.360 ± 0.184	0.232 ± 0.076	SS	1.374 ± 0.195	0.232 ± 0.071	SS
L100 Aml5/B5	1.251 ± 0.165	0.169 ± 0.058	SS	1.268 ± 0.181	0.160 ± 0.066	SS	1.264 ± 0.174	0.150 ± 0.067	SS	1.287 ± 0.168	0.181 ± 0.054	SS	1.326 ± 0.177	0.198 ± 0.070	SS	1.340 ± 0.184	0.198 ± 0.062	SS
L100 Aml5/H12.5	1.304 ± 0.169	0.222 ± 0.050	SS	1.344 ± 0.197	0.236 ± 0.057	SS	1.353 ± 0.191	0.238 ± 0.065	SS	1.410 ± 0.198	0.304 ± 0.071	SS	1.460 ± 0.200	0.333 ± 0.080	SS	1.483 ± 0.213	0.341 ± 0.078	SS
L100 B5/H12.5	1.246 ± 0.164	0.164 ± 0.055	SS	1.271 ± 0.183	0.163 ± 0.059	SS	1.271 ± 0.180	0.157 ± 0.068	SS	1.313 ± 0.178	0.206 ± 0.057	SS	1.359 ± 0.185	0.231 ± 0.073	SS	1.376 ± 0.196	0.234 ± 0.068	SS

Al300 = aliskiren 300 mg; **Aml5** = amlodipine 5 mg; **B5** = bisoprolol 5 mg; **E20** = enalapril 20 mg; **H12.5** = hydrochlorothiazide 12.5 mg; **L100** = losartan 100 mg; **SS** = statistically significant (P < 0.00001)

Table S39. *P*-values calculated using the Kolmogorov-Smirnov test for changes in renal blood flow in subpopulations (n = 100) with different ACE activity receiving the same regimens. Case 1: c_{ACE} = 7.0 h^{-1} (*LII*), case 2: c_{ACE} = 8.9 h^{-1} (*LID*), case 3: c_{ACE} = 10.8 h^{-1} (*LDD*), case 4: c_{ACE} = 42.3 h^{-1} (*HII*), case 5: c_{ACE} = 54.1 h^{-1} (*HID*), case 6: c_{ACE} =65.9 h^{-1} (*HDD*). *P*-value for case *i* *vs*. case *j* is denoted P_{ij}.

Regimens	P_{12}	P_{13}	P_{23}	P_{14}	P_{15}	P_{16}	P_{24}	P_{25}	P_{26}	P_{34}	P_{35}	P_{36}	P_{45}	P_{46}	P_{56}
Al300	0.58062	0.03663	0.69937	SS	SS	SS	SS	SS	SS	SS	SS	SS	0.00079	SS	0.01581
E20	0.11113	0.00386	0.36672	SS	SS	SS	SS	SS	SS	SS	SS	SS	0.00232	0.00002	0.07832
L100	0.69937	0.15454	0.69937	SS	SS	SS	SS	SS	SS	SS	SS	SS	0.00136	SS	0.02431
Aml5	0.36672	0.46756	0.58062	0.05410	0.00232	0.00045	0.36672	0.05410	0.01581	0.90621	0.07832	0.07832	0.28093	0.36672	0.99376
B5	0.58062	0.03663	0.69937	SS	SS	SS	SS	SS	SS	SS	SS	SS	0.00045	SS	0.02431
H12.5	0.81275	0.07832	0.15454	SS	SS	SS	SS	SS	SS	0.00045	0.00136	0.00013	0.81275	0.46756	0.36672
Al300 Aml5	0.58062	0.07832	0.36672	SS	SS	SS	SS	SS	SS	SS	SS	SS	0.03663	0.00013	0.11113
Al300 B5	0.36672	0.21055	0.58062	0.21055	0.00630	0.00630	0.01008	0.00045	0.00007	0.00232	0.00002	SS	0.07832	0.21055	0.90621
Al300 H12.5	0.15454	0.11113	0.90621	SS	SS	SS	SS	SS	SS	SS	SS	SS	0.03663	0.00136	0.36672
E20 Aml5	0.36672	0.00630	0.21055	SS	SS	SS	SS	SS	SS	SS	SS	SS	0.02431	0.00232	0.28093
E20 B5	0.99376	0.58062	0.69937	SS	SS	SS	SS	SS	SS	SS	SS	SS	0.07832	0.21055	0.69937
E20 H12.5	0.07832	0.05410	0.96707	SS	SS	SS	SS	SS	SS	SS	SS	SS	0.05410	0.00025	0.21055
L100 Aml5	0.58062	0.15454	0.28093	SS	SS	SS	SS	SS	SS	SS	SS	SS	0.03663	0.00079	0.11113
L100 B5	0.07832	0.05410	0.58062	0.69937	0.15454	0.36672	0.28093	0.02431	0.02431	0.03663	0.01008	0.00232	0.07832	0.28093	0.99376
L100 H12.5	0.21055	0.21055	0.69937	SS	SS	SS	SS	SS	SS	SS	SS	SS	0.07832	0.00079	0.36672
Al300 Aml5/B5	0.58062	0.28093	0.81275	0.00630	0.00007	0.00013	0.00079	0.00002	SS	0.00025	SS	SS	0.21055	0.21055	0.46756
Al300 Aml5/H12.5	0.05410	0.03663	0.58062	SS	SS	SS	SS	SS	SS	SS	SS	SS	0.15454	0.00232	0.21055
Al300 B5/H12.5	0.90621	0.58062	0.81275	SS	SS	SS	SS	SS	SS	SS	SS	SS	0.11113	0.03663	0.81275
E20 Aml5/B5	0.90621	0.28093	0.90621	SS	SS	SS	SS	SS	SS	SS	SS	SS	0.28093	0.46756	0.21055
E20 Aml5/H12.5	0.03663	0.02431	0.69937	SS	SS	SS	SS	SS	SS	SS	SS	SS	0.11113	0.00079	0.28093
E20 B5/H12.5	0.90621	0.21055	0.46756	SS	SS	SS	SS	SS	SS	SS	SS	SS	0.28093	0.11113	0.96707
L100 Aml5/B5	0.46756	0.11113	0.81275	0.46756	0.03663	0.07832	0.07832	0.00232	0.00386	0.01581	0.00025	0.00007	0.28093	0.36672	0.81275
L100 Aml5/H12.5	0.07832	0.05410	0.69937	SS	SS	SS	SS	SS	SS	SS	SS	SS	0.11113	0.00079	0.21055
L100 B5/H12.5	0.81275	0.46756	0.58062	0.00045	SS	SS	SS	SS	SS	SS	SS	SS	0.11113	0.07832	0.90621

Al300 = aliskiren 300 mg; **Aml5** = amlodipine 5 mg; **B5** = bisoprolol 5 mg; **E20** = enalapril 20 mg; **H12.5** = hydrochlorothiazide 12.5 mg; **L100** = losartan 100 mg; **SS** = statistically significant (P < 0.00001)

Figure S27. Simulated change in <u>renal vascular resistance</u> from baseline to week 4 (mean ± SD, n = 100)

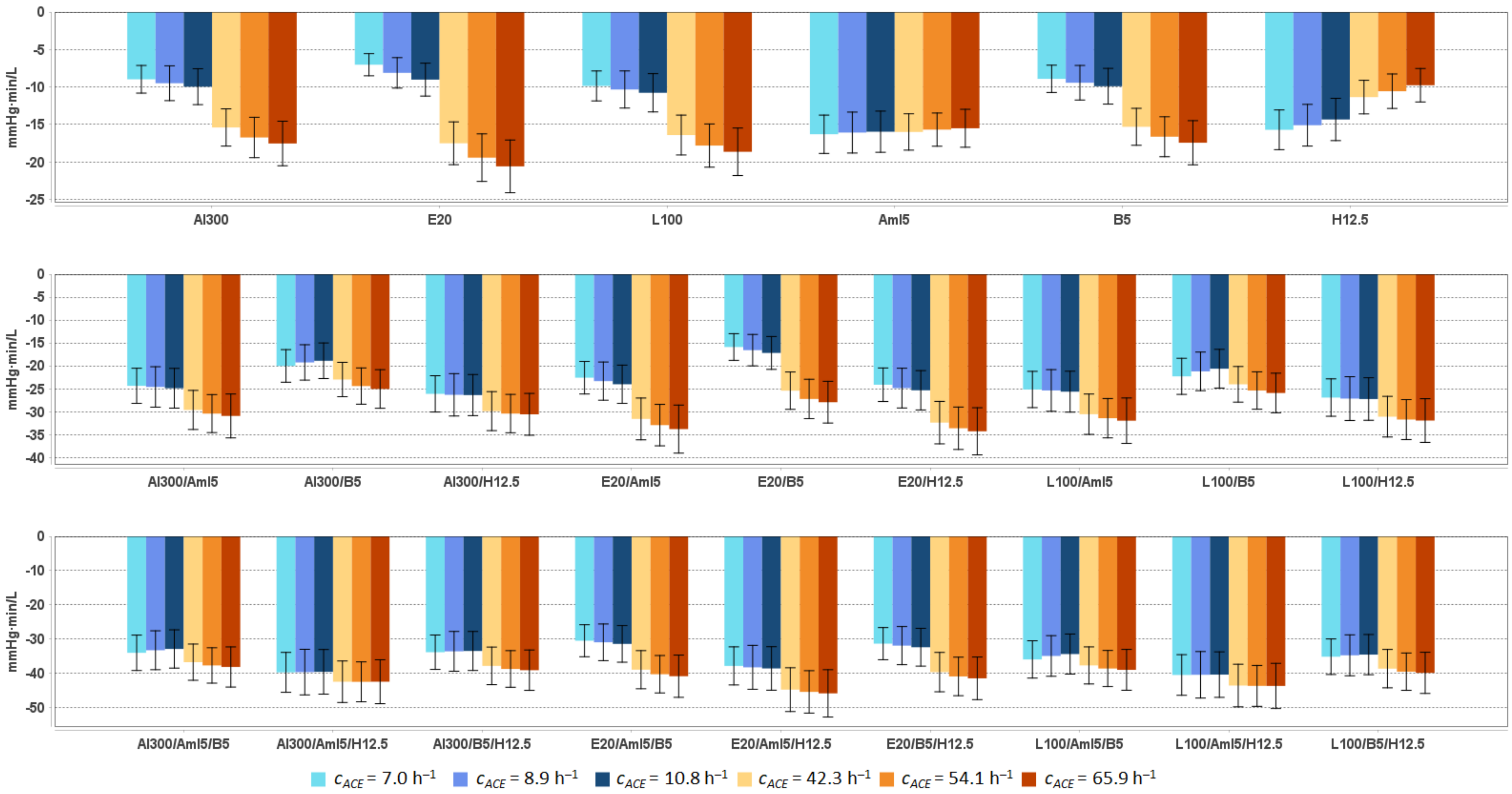

Al300 = aliskiren 300 mg; **Aml5** = amlodipine 5 mg; **B5** = bisoprolol 5 mg; **E20** = enalapril 20 mg; **H12.5** = hydrochlorothiazide 12.5 mg; **L100** = losartan 100 mg

Table S40. Simulated response of renal vascular resistance to antihypertensive therapy in virtual hypertensive subpopulations (n = 100) with different ACE activity, including P-values (Kolmogorov-Smirnov test) for endpoint *vs*. baseline; data are presented as mean ± SD in mmHg·min/L

Regimens	***LII*** **(c_{ACE} = 7.0 h^{-1})**			***LID*** **(c_{ACE} = 8.9 h^{-1})**			***LDD*** **(c_{ACE} = 10.8 h^{-1})**			***HII*** **(c_{ACE} = 42.3 h^{-1})**			***HID*** **(c_{ACE} = 54.1 h^{-1})**			***HDD*** **(c_{ACE} = 65.9 h^{-1})**		
	**Value**	**Change**	***P***	**Value**	**Change**	***P***	**Value**	**Change**	***P***	**Value**	**Change**	***P***	**Value**	**Change**	***P***	**Value**	**Change**	***P***
Baseline	107.4 ± 14.3	–	–	106.0 ± 15.9	–	–	105.1 ± 15.3	–	–	104.7 ± 13.7	–	–	102.7 ± 12.8	–	–	102.0 ± 13.9	–	–
Al300	98.4 ± 13.3	-9.0 ± 1.8	0.00079	96.5 ± 14.3	-9.5 ± 2.3	0.00025	95.1 ± 14.0	-10.0 ± 2.4	0.00232	89.3 ± 12.0	-15.4 ± 2.5	SS	85.9 ± 11.4	-16.7 ± 2.7	SS	84.5 ± 11.9	-17.5 ± 3.0	SS
E20	100.4 ± 13.5	-7.0 ± 1.5	0.00136	97.9 ± 14.5	-8.1 ± 2.0	0.00079	96.0 ± 14.1	-9.0 ± 2.2	0.00386	87.2 ± 11.8	-17.5 ± 2.9	SS	83.2 ± 11.3	-19.4 ± 3.2	SS	81.4 ± 11.6	-20.6 ± 3.5	SS
L100	97.5 ± 13.2	-9.9 ± 2.0	0.00025	95.6 ± 14.2	-10.3 ± 2.5	0.00007	94.3 ± 13.9	-10.8 ± 2.6	0.00025	88.3 ± 11.9	-16.4 ± 2.7	SS	84.9 ± 11.4	-17.8 ± 2.9	SS	83.4 ± 11.7	-18.7 ± 3.2	SS
Aml5	91.1 ± 11.9	-16.3 ± 2.6	SS	89.9 ± 13.4	-16.1 ± 2.7	SS	89.1 ± 12.7	-16.0 ± 2.7	SS	88.7 ± 11.5	-16.0 ± 2.4	SS	87.0 ± 10.9	-15.7 ± 2.2	SS	86.5 ± 11.6	-15.5 ± 2.5	SS
B5	98.5 ± 13.3	-8.9 ± 1.8	0.00079	96.5 ± 14.3	-9.4 ± 2.3	0.00025	95.2 ± 14.0	-9.9 ± 2.4	0.00232	89.4 ± 12.0	-15.3 ± 2.5	SS	86.0 ± 11.4	-16.6 ± 2.7	SS	84.6 ± 11.9	-17.4 ± 2.9	SS
H12.5	91.7 ± 12.0	-15.7 ± 2.7	SS	90.9 ± 13.4	-15.1 ± 2.8	SS	90.7 ± 12.9	-14.3 ± 2.8	SS	93.4 ± 12.1	-11.3 ± 2.2	SS	92.1 ± 11.3	-10.6 ± 2.3	0.00004	92.2 ± 12.7	-9.8 ± 2.2	0.00002
Al300<br>Aml5	83.1 ± 11.1	-24.4 ± 3.8	SS	81.4 ± 12.1	-24.6 ± 4.4	SS	80.2 ± 11.7	-24.9 ± 4.3	SS	75.1 ± 10.0	-29.6 ± 4.3	SS	72.3 ± 9.6	-30.4 ± 4.1	SS	71.1 ± 9.8	-30.9 ± 4.8	SS
Al300<br>B5	87.4 ± 12.0	-20.0 ± 3.6	SS	86.7 ± 13.1	-19.3 ± 3.9	SS	86.2 ± 12.7	-18.9 ± 3.9	SS	81.7 ± 11.4	-23.0 ± 3.7	SS	78.3 ± 11.0	-24.4 ± 4.0	SS	77.0 ± 11.2	-25.0 ± 4.2	SS
Al300<br>H12.5	81.3 ± 10.9	-26.1 ± 4.0	SS	79.7 ± 11.7	-26.3 ± 4.6	SS	78.7 ± 11.3	-26.4 ± 4.5	SS	74.8 ± 9.8	-29.9 ± 4.3	SS	72.3 ± 9.2	-30.4 ± 4.2	SS	71.4 ± 9.8	-30.6 ± 4.6	SS
E20<br>Aml5	84.8 ± 11.3	-22.6 ± 3.6	SS	82.7 ± 12.3	-23.3 ± 4.2	SS	81.0 ± 11.8	-24.0 ± 4.2	SS	73.1 ± 9.9	-31.6 ± 4.6	SS	69.8 ± 9.5	-32.9 ± 4.5	SS	68.2 ± 9.6	-33.8 ± 5.2	SS
E20<br>B5	91.5 ± 12.5	-15.9 ± 2.9	SS	89.4 ± 13.4	-16.6 ± 3.4	SS	87.9 ± 13.0	-17.2 ± 3.6	SS	79.3 ± 11.1	-25.4 ± 4.1	SS	75.5 ± 10.8	-27.2 ± 4.3	SS	74.1 ± 11.0	-27.9 ± 4.5	SS
E20<br>H12.5	83.3 ± 11.1	-24.1 ± 3.7	SS	81.1 ± 11.9	-24.9 ± 4.3	SS	79.7 ± 11.4	-25.3 ± 4.3	SS	72.3 ± 9.6	-32.4 ± 4.6	SS	69.1 ± 9.0	-33.6 ± 4.6	SS	67.7 ± 9.5	-34.3 ± 5.1	SS
L100<br>Aml5	82.3 ± 11.0	-25.2 ± 4.0	SS	80.6 ± 12.0	-25.4 ± 4.5	SS	79.4 ± 11.6	-25.6 ± 4.5	SS	74.2 ± 10.0	-30.6 ± 4.4	SS	71.3 ± 9.6	-31.4 ± 4.3	SS	70.1 ± 9.7	-32.0 ± 5.0	SS
L100<br>B5	85.1 ± 11.8	-22.3 ± 3.9	SS	84.8 ± 12.9	-21.2 ± 4.2	SS	84.4 ± 12.5	-20.6 ± 4.2	SS	80.7 ± 11.3	-24.0 ± 3.9	SS	77.3 ± 11.0	-25.4 ± 4.1	SS	76.1 ± 11.2	-25.9 ± 4.3	SS
L100<br>H12.5	80.5 ± 10.9	-26.9 ± 4.1	SS	78.8 ± 11.6	-27.1 ± 4.8	SS	77.8 ± 11.2	-27.2 ± 4.6	SS	73.6 ± 9.7	-31.1 ± 4.4	SS	71.0 ± 9.1	-31.7 ± 4.4	SS	70.1 ± 9.7	-31.9 ± 4.8	SS
Al300<br>Aml5/B5	73.5 ± 10.1	-33.9 ± 5.2	SS	72.8 ± 11.1	-33.2 ± 5.7	SS	72.2 ± 10.7	-32.8 ± 5.6	SS	68.0 ± 9.5	-36.7 ± 5.3	SS	65.0 ± 9.3	-37.6 ± 5.2	SS	63.9 ± 9.3	-38.1 ± 5.9	SS
Al300<br>Aml5/H12.5	67.8 ± 9.1	-39.6 ± 5.8	SS	66.4 ± 9.7	-39.6 ± 6.6	SS	65.6 ± 9.3	-39.5 ± 6.5	SS	62.3 ± 8.1	-42.4 ± 6.1	SS	60.3 ± 7.6	-42.4 ± 5.8	SS	59.6 ± 8.0	-42.4 ± 6.4	SS
Al300<br>B5/H12.5	73.6 ± 10.2	-33.8 ± 5.0	SS	72.5 ± 10.8	-33.5 ± 5.8	SS	71.6 ± 10.5	-33.4 ± 5.7	SS	67.0 ± 9.2	-37.8 ± 5.5	SS	64.0 ± 8.8	-38.7 ± 5.3	SS	63.0 ± 9.1	-39.0 ± 5.9	SS
E20<br>Aml5/B5	77.0 ± 10.5	-30.4 ± 4.7	SS	75.1 ± 11.3	-30.9 ± 5.3	SS	73.7 ± 10.9	-31.3 ± 5.3	SS	65.9 ± 9.3	-38.9 ± 5.6	SS	62.5 ± 9.1	-40.2 ± 5.5	SS	61.2 ± 9.1	-40.8 ± 6.2	SS
E20<br>Aml5/H12.5	69.6 ± 9.2	-37.8 ± 5.6	SS	67.8 ± 9.9	-38.2 ± 6.4	SS	66.6 ± 9.4	-38.5 ± 6.3	SS	60.0 ± 7.9	-44.7 ± 6.4	SS	57.3 ± 7.4	-45.3 ± 6.2	SS	56.2 ± 7.7	-45.8 ± 6.9	SS
E20<br>B5/H12.5	76.1 ± 10.5	-31.3 ± 4.7	SS	74.1 ± 11.0	-31.9 ± 5.6	SS	72.7 ± 10.7	-32.3 ± 5.5	SS	65.2 ± 9.0	-39.6 ± 5.7	SS	61.8 ± 8.6	-40.8 ± 5.6	SS	60.6 ± 8.9	-41.4 ± 6.2	SS
L100<br>Aml5/B5	71.5 ± 9.9	-35.9 ± 5.4	SS	71.1 ± 10.9	-34.9 ± 5.9	SS	70.7 ± 10.5	-34.3 ± 5.8	SS	67.1 ± 9.4	-37.6 ± 5.4	SS	64.1 ± 9.2	-38.5 ± 5.3	SS	63.1 ± 9.2	-38.9 ± 6.0	SS
L100<br>Aml5/H12.5	67.0 ± 9.0	-40.4 ± 5.9	SS	65.6 ± 9.6	-40.4 ± 6.8	SS	64.8 ± 9.2	-40.3 ± 6.6	SS	61.2 ± 8.0	-43.5 ± 6.2	SS	59.1 ± 7.5	-43.6 ± 6.0	SS	58.4 ± 7.9	-43.6 ± 6.6	SS
L100<br>B5/H12.5	72.3 ± 10.1	-35.1 ± 5.2	SS	71.3 ± 10.7	-34.7 ± 6.0	SS	70.6 ± 10.4	-34.5 ± 5.9	SS	66.2 ± 9.1	-38.6 ± 5.6	SS	63.2 ± 8.7	-39.5 ± 5.4	SS	62.2 ± 9.0	-39.8 ± 6.0	SS

Al300 = aliskiren 300 mg; **Aml5** = amlodipine 5 mg; **B5** = bisoprolol 5 mg; **E20** = enalapril 20 mg; **H12.5** = hydrochlorothiazide 12.5 mg; **L100** = losartan 100 mg; **SS** = statistically significant (P < 0.00001)

Table S41. *P*-values calculated using the Kolmogorov-Smirnov test for changes in renal vascular resistance in subpopulations (n = 100) with different ACE activity receiving the same regimens. Case 1: c_{ACE} = 7.0 h^{-1} (*LII*), case 2: c_{ACE} = 8.9 h^{-1} (*LID*), case 3: c_{ACE} = 10.8 h^{-1} (*LDD*), case 4: c_{ACE} = 42.3 h^{-1} (*HII*), case 5: c_{ACE} = 54.1 h^{-1} (*HID*), case 6: c_{ACE} = 65.9 h^{-1} (*HDD*). *P*-value for case *i vs*. case *j* is denoted P_{ij}.

Regimens	P_{12}	P_{13}	P_{23}	P_{14}	P_{15}	P_{16}	P_{24}	P_{25}	P_{26}	P_{34}	P_{35}	P_{36}	P_{45}	P_{46}	P_{56}
Al300	0.28093	0.00232	0.15454	SS	SS	SS	SS	SS	SS	SS	SS	SS	0.00136	0.00002	0.28093
E20	0.01008	SS	0.00630	SS	SS	SS	SS	SS	SS	SS	SS	SS	SS	SS	0.21055
L100	0.46756	0.00386	0.21055	SS	SS	SS	SS	SS	SS	SS	SS	SS	0.00136	0.00002	0.28093
Aml5	0.81275	0.11113	0.69937	0.69937	0.36672	0.11113	0.69937	0.46756	0.11113	0.36672	0.11113	0.28093	0.81275	0.28093	0.36672
B5	0.28093	0.00232	0.11113	SS	SS	SS	SS	SS	SS	SS	SS	SS	0.00079	0.00002	0.28093
H12.5	0.11113	0.00386	0.15454	SS	SS	SS	SS	SS	SS	SS	SS	SS	0.11113	0.00045	0.02431
Al300 Aml5	0.90621	0.69937	0.90621	SS	SS	SS	SS	SS	SS	SS	SS	SS	0.21055	0.15454	0.69937
Al300 B5	0.58062	0.01581	0.28093	SS	SS	SS	SS	SS	SS	SS	SS	SS	0.03663	0.00232	0.81275
Al300 H12.5	0.69937	0.96707	0.90621	SS	SS	SS	SS	SS	SS	SS	SS	SS	0.36672	0.81275	0.90621
E20 Aml5	0.46756	0.07832	0.46756	SS	SS	SS	SS	SS	SS	SS	SS	SS	0.03663	0.01008	0.58062
E20 B5	0.28093	0.03663	0.15454	SS	SS	SS	SS	SS	SS	SS	SS	SS	0.01581	0.00386	0.81275
E20 H12.5	0.36672	0.11113	0.81275	SS	SS	SS	SS	SS	SS	SS	SS	SS	0.11113	0.07832	0.69937
L100 Aml5	0.90621	0.81275	0.81275	SS	SS	SS	SS	SS	SS	SS	SS	SS	0.15454	0.11113	0.81275
L100 B5	0.46756	0.01581	0.28093	0.00025	SS	SS	SS	SS	SS	SS	SS	SS	0.03663	0.02431	0.81275
L100 H12.5	0.58062	0.90621	0.81275	SS	SS	SS	SS	SS	SS	SS	SS	SS	0.28093	0.58062	0.81275
Al300 Aml5/B5	0.69937	0.05410	0.46756	0.00079	0.00002	0.00002	0.00013	SS	SS	0.00002	SS	SS	0.36672	0.28093	0.81275
Al300 Aml5/H12.5	0.81275	0.46756	0.81275	0.00386	0.01581	0.02431	0.00136	0.00386	0.01008	0.00045	0.00045	0.00232	0.90621	0.90621	0.96707
Al300 B5/H12.5	0.81275	0.46756	0.96707	SS	SS	SS	SS	SS	SS	SS	SS	SS	0.21055	0.28093	0.90621
E20 Aml5/B5	0.81275	0.58062	0.96707	SS	SS	SS	SS	SS	SS	SS	SS	SS	0.21055	0.07832	0.81275
E20 Aml5/H12.5	0.69937	0.46756	0.90621	SS	SS	SS	SS	SS	SS	SS	SS	SS	0.46756	0.69937	0.96707
E20 B5/H12.5	0.46756	0.46756	0.90621	SS	SS	SS	SS	SS	SS	SS	SS	SS	0.15454	0.21055	0.90621
L100 Aml5/B5	0.69937	0.05410	0.46756	0.07832	0.00232	0.00136	0.00386	0.00025	0.00045	0.00007	SS	0.00002	0.46756	0.36672	0.81275
L100 Aml5/H12.5	0.81275	0.46756	0.69937	0.00232	0.01008	0.01581	0.00079	0.00232	0.00630	0.00013	0.00007	0.00136	0.81275	0.90621	0.96707
L100 B5/H12.5	0.81275	0.28093	0.90621	0.00013	SS	SS	0.00002	SS	SS	SS	SS	SS	0.15454	0.36672	0.90621

Al300 = aliskiren 300 mg; **Aml5** = amlodipine 5 mg; **B5** = bisoprolol 5 mg; **E20** = enalapril 20 mg; **H12.5** = hydrochlorothiazide 12.5 mg; **L100** = losartan 100 mg; **SS** = statistically significant (P < 0.00001)

Figure S28. Simulated change in afferent arteriolar resistance from baseline to week 4 (mean ± SD, *n* = 100)

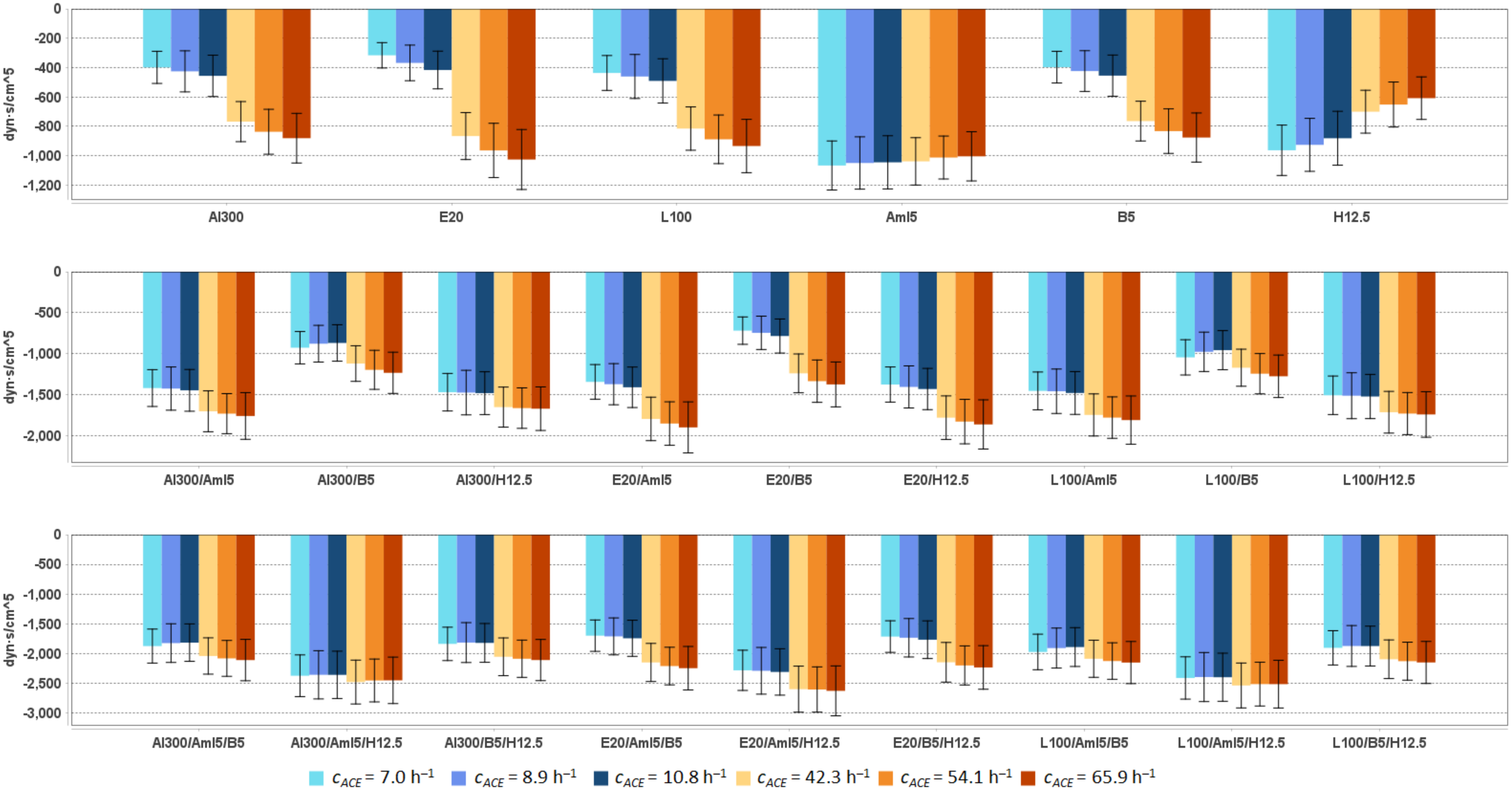

Al300 = aliskiren 300 mg; **Aml5** = amlodipine 5 mg; **B5** = bisoprolol 5 mg; **E20** = enalapril 20 mg; **H12.5** = hydrochlorothiazide 12.5 mg; **L100** = losartan 100 mg

Table S42. Simulated response of afferent arteriolar resistance to antihypertensive therapy in virtual hypertensive subpopulations (n = 100) with different ACE activity, including P-values (Kolmogorov-Smirnov test) for endpoint *vs*. baseline; data are presented as mean ± SD in dyn·s/cm^5

Regimens	*LII* (c_{ACE} = 7.0 h^{-1})			*LID* (c_{ACE} = 8.9 h^{-1})			*LDD* (c_{ACE} = 10.8 h^{-1})			*HII* (c_{ACE} = 42.3 h^{-1})			*HID* (c_{ACE} = 54.1 h^{-1})			*HDD* (c_{ACE} = 65.9 h^{-1})		
	**Value**	**Change**	***P***	**Value**	**Change**	***P***	**Value**	**Change**	***P***	**Value**	**Change**	***P***	**Value**	**Change**	***P***	**Value**	**Change**	***P***
Baseline	5011 ± 814	–	–	4936 ± 864	–	–	4886 ± 859	–	–	4773 ± 756	–	–	4613 ± 739	–	–	4586 ± 778	–	–
Al300	4613 ± 774	-398 ± 109	0.01581	4511 ± 792	-425 ± 140	0.00630	4430 ± 810	-456 ± 141	0.00630	4006 ± 687	-768 ± 136	SS	3776 ± 680	-837 ± 153	SS	3706 ± 678	-881 ± 169	SS
E20	4695 ± 780	-316 ± 86	0.05410	4569 ± 800	-368 ± 121	0.01008	4470 ± 814	-416 ± 128	0.01581	3907 ± 684	-866 ± 160	SS	3649 ± 680	-964 ± 185	SS	3561 ± 670	-1026 ± 204	SS
L100	4575 ± 771	-437 ± 119	0.01008	4476 ± 787	-461 ± 150	0.00232	4396 ± 807	-491 ± 151	0.00386	3958 ± 685	-815 ± 147	SS	3724 ± 680	-888 ± 165	SS	3652 ± 675	-934 ± 182	SS
Aml5	3945 ± 665	-1066 ± 167	SS	3887 ± 702	-1049 ± 178	SS	3841 ± 697	-1045 ± 181	SS	3735 ± 619	-1038 ± 161	SS	3601 ± 617	-1012 ± 146	SS	3582 ± 631	-1004 ± 168	SS
B5	4614 ± 774	-397 ± 108	0.01581	4513 ± 792	-424 ± 139	0.00630	4432 ± 810	-455 ± 140	0.00630	4009 ± 687	-764 ± 135	SS	3780 ± 679	-833 ± 152	SS	3710 ± 678	-876 ± 167	SS
H12.5	4049 ± 671	-963 ± 171	SS	4011 ± 710	-926 ± 180	SS	4005 ± 707	-881 ± 183	SS	4073 ± 653	-700 ± 146	SS	3961 ± 628	-652 ± 153	0.00002	3978 ± 696	-608 ± 145	SS
Al300 Aml5	3592 ± 639	-1420 ± 225	SS	3510 ± 649	-1426 ± 264	SS	3439 ± 662	-1448 ± 255	SS	3070 ± 563	-1703 ± 250	SS	2881 ± 566	-1732 ± 244	SS	2826 ± 549	-1760 ± 285	SS
Al300 B5	4084 ± 727	-927 ± 197	SS	4058 ± 738	-879 ± 224	SS	4017 ± 761	-869 ± 223	SS	3653 ± 678	-1121 ± 217	SS	3415 ± 681	-1197 ± 238	SS	3353 ± 666	-1234 ± 252	SS
Al300 H12.5	3541 ± 629	-1470 ± 229	SS	3462 ± 626	-1475 ± 272	SS	3404 ± 640	-1482 ± 261	SS	3121 ± 549	-1652 ± 244	SS	2948 ± 532	-1664 ± 247	SS	2915 ± 550	-1672 ± 266	SS
E20 Aml5	3667 ± 643	-1344 ± 211	SS	3564 ± 655	-1373 ± 251	SS	3476 ± 664	-1410 ± 247	SS	2977 ± 561	-1796 ± 265	SS	2760 ± 566	-1853 ± 265	SS	2687 ± 544	-1900 ± 311	SS
E20 B5	4293 ± 746	-719 ± 167	SS	4191 ± 752	-746 ± 203	SS	4102 ± 772	-785 ± 208	SS	3533 ± 672	-1240 ± 237	SS	3277 ± 678	-1336 ± 258	SS	3211 ± 663	-1375 ± 274	SS
E20 H12.5	3635 ± 633	-1377 ± 215	SS	3531 ± 634	-1406 ± 257	SS	3455 ± 643	-1431 ± 252	SS	2992 ± 542	-1781 ± 266	SS	2784 ± 526	-1829 ± 272	SS	2723 ± 535	-1864 ± 300	SS
L100 Aml5	3557 ± 638	-1454 ± 231	SS	3478 ± 646	-1459 ± 271	SS	3406 ± 659	-1480 ± 261	SS	3026 ± 562	-1748 ± 257	SS	2832 ± 566	-1780 ± 253	SS	2776 ± 546	-1811 ± 294	SS
L100 B5	3966 ± 717	-1045 ± 215	SS	3959 ± 729	-978 ± 240	SS	3929 ± 751	-957 ± 238	SS	3602 ± 676	-1172 ± 226	SS	3368 ± 680	-1244 ± 246	SS	3310 ± 665	-1276 ± 259	SS
L100 H12.5	3503 ± 628	-1508 ± 235	SS	3423 ± 622	-1513 ± 280	SS	3363 ± 637	-1523 ± 270	SS	3059 ± 545	-1715 ± 255	SS	2881 ± 529	-1731 ± 257	SS	2844 ± 544	-1742 ± 278	SS
Al300 Aml5/B5	3137 ± 611	-1875 ± 286	SS	3112 ± 613	-1825 ± 325	SS	3070 ± 627	-1816 ± 315	SS	2732 ± 557	-2041 ± 305	SS	2531 ± 568	-2081 ± 302	SS	2476 ± 543	-2110 ± 348	SS
Al300 Aml5/H12.5	2638 ± 516	-2373 ± 351	SS	2579 ± 503	-2358 ± 406	SS	2528 ± 510	-2358 ± 398	SS	2294 ± 435	-2480 ± 369	SS	2160 ± 425	-2453 ± 360	SS	2136 ± 428	-2451 ± 391	SS
Al300 B5/H12.5	3174 ± 615	-1838 ± 281	SS	3121 ± 597	-1816 ± 336	SS	3065 ± 617	-1821 ± 326	SS	2719 ± 537	-2054 ± 317	SS	2525 ± 526	-2087 ± 313	SS	2477 ± 527	-2109 ± 346	SS
E20 Aml5/B5	3312 ± 623	-1699 ± 264	SS	3225 ± 623	-1711 ± 311	SS	3143 ± 634	-1743 ± 304	SS	2623 ± 553	-2150 ± 320	SS	2402 ± 565	-2211 ± 316	SS	2340 ± 542	-2246 ± 366	SS
E20 Aml5/H12.5	2729 ± 518	-2282 ± 338	SS	2646 ± 509	-2290 ± 393	SS	2577 ± 512	-2310 ± 390	SS	2174 ± 429	-2599 ± 387	SS	2008 ± 418	-2605 ± 381	SS	1959 ± 417	-2628 ± 420	SS
E20 B5/H12.5	3297 ± 622	-1715 ± 266	SS	3202 ± 603	-1734 ± 323	SS	3119 ± 622	-1767 ± 317	SS	2626 ± 536	-2147 ± 333	SS	2412 ± 525	-2200 ± 329	SS	2352 ± 526	-2234 ± 367	SS
L100 Aml5/B5	3038 ± 604	-1973 ± 299	SS	3029 ± 606	-1908 ± 336	SS	2995 ± 619	-1891 ± 327	SS	2686 ± 555	-2088 ± 312	SS	2486 ± 567	-2126 ± 307	SS	2435 ± 543	-2152 ± 354	SS
L100 Aml5/H12.5	2601 ± 515	-2411 ± 356	SS	2541 ± 501	-2395 ± 413	SS	2488 ± 509	-2399 ± 405	SS	2236 ± 432	-2537 ± 377	SS	2099 ± 422	-2514 ± 368	SS	2072 ± 423	-2515 ± 401	SS
L100 B5/H12.5	3107 ± 610	-1904 ± 289	SS	3063 ± 593	-1873 ± 344	SS	3012 ± 613	-1874 ± 335	SS	2678 ± 537	-2096 ± 325	SS	2484 ± 526	-2129 ± 319	SS	2436 ± 526	-2150 ± 353	SS

Al300 = aliskiren 300 mg; **Aml5** = amlodipine 5 mg; **B5** = bisoprolol 5 mg; **E20** = enalapril 20 mg; **H12.5** = hydrochlorothiazide 12.5 mg; **L100** = losartan 100 mg; **SS** = statistically significant (P < 0.00001)

Table S43. P-values calculated using the Kolmogorov-Smirnov test for changes in afferent arteriolar resistance in subpopulations (n = 100) with different ACE activity receiving the same regimens. Case 1: c_{ACE} = 7.0 h^{-1} (*LII*), case 2: c_{ACE} = 8.9 h^{-1} (*LID*), case 3: c_{ACE} = 10.8 h^{-1} (*LDD*), case 4: c_{ACE} = 42.3 h^{-1} (*HII*), case 5: c_{ACE} = 54.1 h^{-1} (*HID*), case 6: c_{ACE} = 65.9 h^{-1} (*HDD*). P-value for case i *vs*. case j is denoted P_{ij}.

Regimens	P_{12}	P_{13}	P_{23}	P_{14}	P_{15}	P_{16}	P_{24}	P_{25}	P_{26}	P_{34}	P_{35}	P_{36}	P_{45}	P_{46}	P_{56}
Al300	0.07832	0.00079	0.21055	SS	SS	SS	SS	SS	SS	SS	SS	SS	0.00079	0.00004	0.28093
E20	0.01008	SS	0.01581	SS	SS	SS	SS	SS	SS	SS	SS	SS	0.00007	SS	0.11113
L100	0.15454	0.00386	0.21055	SS	SS	SS	SS	SS	SS	SS	SS	SS	0.00232	0.00004	0.28093
Aml5	0.69937	0.07832	0.58062	0.58062	0.28093	0.01008	0.46756	0.21055	0.11113	0.81275	0.15454	0.28093	0.58062	0.11113	0.46756
B5	0.11113	0.00136	0.28093	SS	SS	SS	SS	SS	SS	SS	SS	SS	0.00136	0.00007	0.21055
H12.5	0.21055	0.01008	0.15454	SS	SS	SS	SS	SS	SS	SS	SS	SS	0.07832	0.00079	0.05410
Al300<br>Aml5	0.81275	0.69937	0.81275	SS	SS	SS	SS	SS	SS	SS	SS	SS	0.69937	0.58062	0.81275
Al300<br>B5	0.46756	0.21055	0.96707	SS	SS	SS	SS	SS	SS	SS	SS	SS	0.01008	0.00136	0.69937
Al300<br>H12.5	0.81275	0.81275	0.99376	SS	SS	0.00004	0.00004	0.00004	0.00002	0.00004	0.00007	0.00004	0.96707	0.58062	0.58062
E20<br>Aml5	0.28093	0.11113	0.46756	SS	SS	SS	SS	SS	SS	SS	SS	SS	0.21055	0.21055	0.58062
E20<br>B5	0.46756	0.03663	0.46756	SS	SS	SS	SS	SS	SS	SS	SS	SS	0.00386	0.00079	0.69937
E20<br>H12.5	0.58062	0.28093	0.96707	SS	SS	SS	SS	SS	SS	SS	SS	SS	0.36672	0.46756	0.46756
L100<br>Aml5	0.81275	0.69937	0.81275	SS	SS	SS	SS	SS	SS	SS	SS	SS	0.69937	0.58062	0.81275
L100<br>B5	0.21055	0.05410	0.69937	0.00013	SS	SS	SS	SS	SS	SS	SS	SS	0.03663	0.00386	0.81275
L100<br>H12.5	0.81275	0.69937	0.99376	SS	SS	SS	SS	SS	SS	SS	0.00002	SS	0.81275	0.90621	0.69937
Al300<br>Aml5/B5	0.28093	0.21055	0.96707	0.00025	SS	0.00002	0.00004	SS	SS	0.00002	SS	SS	0.28093	0.46756	0.69937
Al300<br>Aml5/H12.5	0.58062	0.28093	0.90621	0.11113	0.28093	0.46756	0.05410	0.21055	0.21055	0.00630	0.07832	0.05410	0.69937	0.21055	0.69937
Al300<br>B5/H12.5	0.36672	0.46756	0.90621	0.00004	SS	0.00002	SS	SS	SS	SS	SS	SS	0.81275	0.81275	0.90621
E20<br>Aml5/B5	0.69937	0.36672	0.58062	SS	SS	SS	SS	SS	SS	SS	SS	SS	0.21055	0.46756	0.81275
E20<br>Aml5/H12.5	0.69937	0.58062	0.69937	SS	SS	SS	SS	0.00007	0.00002	SS	0.00002	SS	0.96707	0.99376	0.69937
E20<br>B5/H12.5	0.58062	0.58062	0.36672	SS	SS	SS	SS	SS	SS	SS	SS	SS	0.46756	0.58062	0.81275
L100<br>Aml5/B5	0.21055	0.15454	0.81275	0.00630	0.00079	0.00079	0.00136	0.00002	0.00013	0.00013	0.00002	0.00002	0.46756	0.46756	0.81275
L100<br>Aml5/H12.5	0.69937	0.28093	0.90621	0.07832	0.21055	0.36672	0.01581	0.11113	0.15454	0.00386	0.03663	0.03663	0.81275	0.36672	0.69937
L100<br>B5/H12.5	0.36672	0.28093	0.96707	0.00013	0.00002	0.00025	SS	0.00004	SS	SS	SS	SS	0.81275	0.81275	0.90621

Al300 = aliskiren 300 mg; **Aml5** = amlodipine 5 mg; **B5** = bisoprolol 5 mg; **E20** = enalapril 20 mg; **H12.5** = hydrochlorothiazide 12.5 mg; **L100** = losartan 100 mg; **SS** = statistically significant (P < 0.00001)

Figure S29. Simulated change in efferent arteriolar resistance from baseline to week 4 (mean ± SD, n = 100)

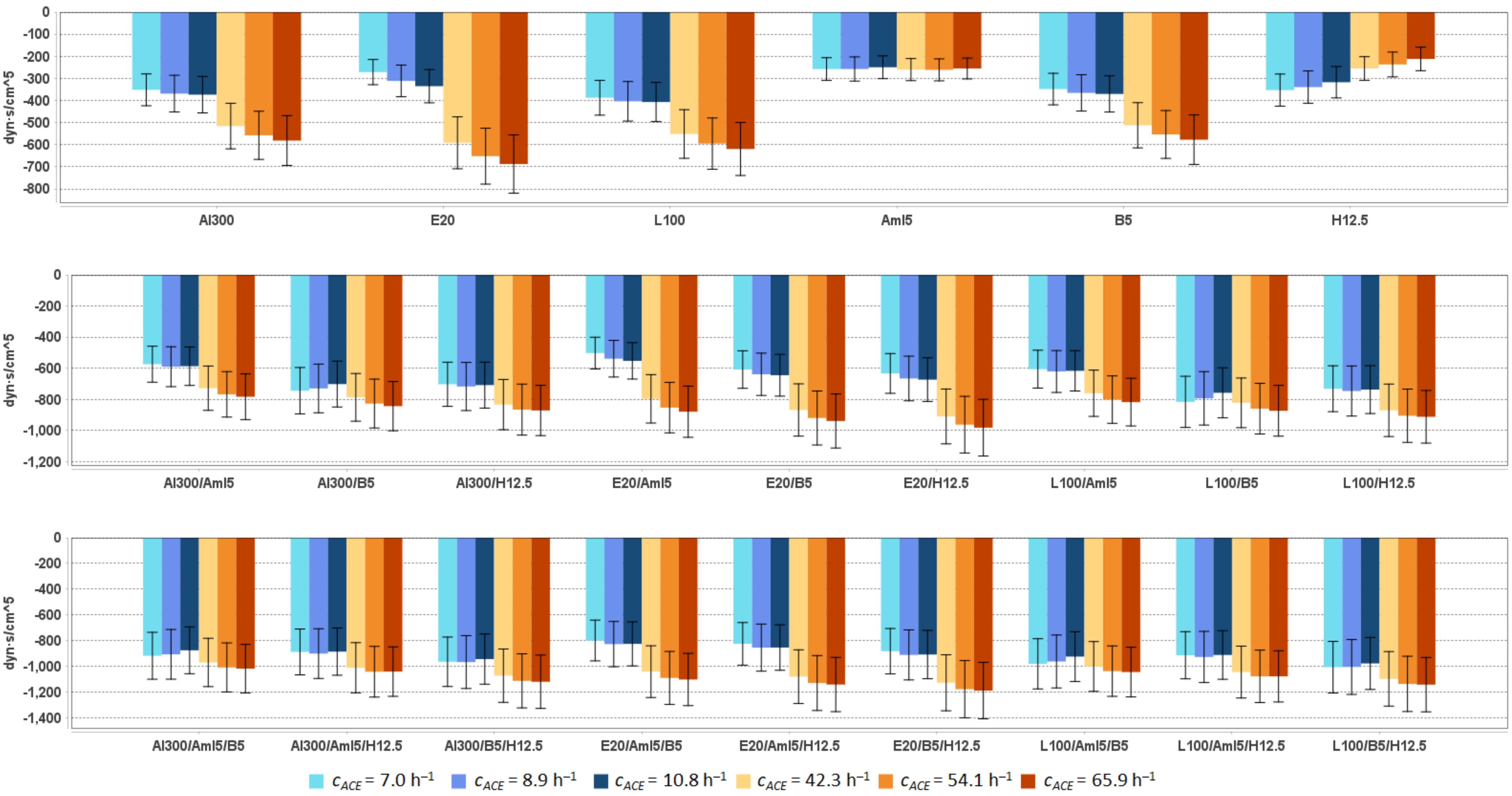

Al300 = aliskiren 300 mg; **Aml5** = amlodipine 5 mg; **B5** = bisoprolol 5 mg; **E20** = enalapril 20 mg; **H12.5** = hydrochlorothiazide 12.5 mg; **L100** = losartan 100 mg

Table S44. Simulated response of <u>efferent arteriolar resistance</u> to antihypertensive therapy in virtual hypertensive subpopulations (n = 100) with different ACE activity, including P-values (Kolmogorov-Smirnov test) for endpoint *vs*. baseline; data are presented as mean ± SD in dyn·s/cm^5

Regimens	***LII*** **(c_{ACE} = 7.0 h^{-1})**			***LID*** **(c_{ACE} = 8.9 h^{-1})**			***LDD*** **(c_{ACE} = 10.8 h^{-1})**			***HII*** **(c_{ACE} = 42.3 h^{-1})**			***HID*** **(c_{ACE} = 54.1 h^{-1})**			***HDD*** **(c_{ACE} = 65.9 h^{-1})**		
	**Value**	**Change**	***P***	**Value**	**Change**	***P***	**Value**	**Change**	***P***	**Value**	**Change**	***P***	**Value**	**Change**	***P***	**Value**	**Change**	***P***
Baseline	2485 ± 493	–	–	2489 ± 530	–	–	2419 ± 498	–	–	2559 ± 491	–	–	2581 ± 491	–	–	2528 ± 466	–	–
Al300	2133 ± 422	-352 ± 72	0.00013	2120 ± 450	-369 ± 83	0.00079	2045 ± 422	-374 ± 82	0.00025	2043 ± 392	-516 ± 103	SS	2023 ± 392	-557 ± 109	SS	1947 ± 364	-581 ± 113	SS
E20	2213 ± 438	-272 ± 57	0.00386	2178 ± 463	-311 ± 72	0.00630	2083 ± 430	-335 ± 75	0.00079	1967 ± 378	-591 ± 117	SS	1929 ± 375	-652 ± 126	SS	1841 ± 345	-687 ± 132	SS
L100	2097 ± 415	-388 ± 79	SS	2085 ± 443	-404 ± 90	0.00013	2012 ± 415	-407 ± 89	0.00007	2007 ± 385	-552 ± 110	SS	1986 ± 385	-595 ± 116	SS	1909 ± 357	-619 ± 120	SS
Aml5	2227 ± 441	-258 ± 51	0.00630	2232 ± 475	-257 ± 55	0.02431	2169 ± 446	-250 ± 51	0.01581	2299 ± 441	-260 ± 50	0.00232	2319 ± 441	-261 ± 50	0.00386	2273 ± 419	-255 ± 47	0.00232
B5	2137 ± 422	-348 ± 71	0.00013	2124 ± 451	-366 ± 82	0.00079	2048 ± 423	-370 ± 82	0.00025	2047 ± 393	-512 ± 102	SS	2027 ± 393	-554 ± 108	SS	1951 ± 365	-577 ± 112	SS
H12.5	2132 ± 423	-353 ± 73	0.00025	2150 ± 460	-339 ± 73	0.00232	2101 ± 432	-317 ± 71	0.00136	2304 ± 444	-255 ± 54	0.00232	2344 ± 444	-237 ± 56	0.01581	2316 ± 425	-212 ± 53	0.01581
Al300<br>Aml5	1911 ± 378	-574 ± 116	SS	1899 ± 403	-590 ± 128	SS	1832 ± 378	-587 ± 124	SS	1831 ± 351	-728 ± 142	SS	1813 ± 351	-768 ± 146	SS	1745 ± 326	-783 ± 147	SS
Al300<br>B5	1741 ± 349	-744 ± 149	SS	1759 ± 377	-730 ± 156	SS	1717 ± 352	-702 ± 147	SS	1772 ± 340	-787 ± 153	SS	1754 ± 340	-827 ± 157	SS	1685 ± 316	-843 ± 158	SS
Al300<br>H12.5	1782 ± 351	-703 ± 142	SS	1772 ± 377	-717 ± 155	SS	1710 ± 353	-708 ± 148	SS	1725 ± 331	-833 ± 161	SS	1715 ± 331	-866 ± 164	SS	1657 ± 308	-871 ± 161	SS
E20<br>Aml5	1982 ± 392	-502 ± 102	SS	1951 ± 414	-539 ± 118	SS	1866 ± 385	-552 ± 117	SS	1762 ± 338	-796 ± 156	SS	1728 ± 336	-853 ± 162	SS	1649 ± 309	-879 ± 165	SS
E20<br>B5	1877 ± 373	-608 ± 121	SS	1850 ± 395	-639 ± 136	SS	1774 ± 364	-645 ± 134	SS	1691 ± 324	-868 ± 168	SS	1661 ± 321	-920 ± 174	SS	1589 ± 297	-939 ± 174	SS
E20<br>H12.5	1852 ± 365	-633 ± 128	SS	1823 ± 388	-666 ± 143	SS	1746 ± 360	-673 ± 140	SS	1649 ± 316	-910 ± 176	SS	1618 ± 313	-962 ± 182	SS	1546 ± 289	-982 ± 182	SS
L100<br>Aml5	1879 ± 371	-606 ± 122	SS	1868 ± 397	-621 ± 135	SS	1802 ± 372	-616 ± 129	SS	1798 ± 345	-761 ± 149	SS	1779 ± 345	-802 ± 153	SS	1710 ± 320	-818 ± 153	SS
L100<br>B5	1669 ± 337	-816 ± 164	SS	1696 ± 366	-793 ± 172	SS	1661 ± 341	-758 ± 161	SS	1736 ± 333	-822 ± 160	SS	1721 ± 333	-860 ± 163	SS	1655 ± 310	-873 ± 163	SS
L100<br>H12.5	1753 ± 346	-732 ± 147	SS	1743 ± 371	-746 ± 161	SS	1681 ± 347	-737 ± 154	SS	1688 ± 324	-870 ± 169	SS	1676 ± 324	-905 ± 171	SS	1616 ± 301	-912 ± 169	SS
Al300<br>Aml5/B5	1566 ± 313	-919 ± 182	SS	1582 ± 339	-907 ± 193	SS	1542 ± 317	-876 ± 182	SS	1588 ± 304	-971 ± 188	SS	1571 ± 305	-1009 ± 191	SS	1509 ± 283	-1019 ± 189	SS
Al300<br>Aml5/H12.5	1596 ± 315	-889 ± 178	SS	1587 ± 337	-902 ± 193	SS	1532 ± 316	-886 ± 184	SS	1547 ± 297	-1012 ± 195	SS	1538 ± 296	-1043 ± 197	SS	1486 ± 276	-1042 ± 192	SS
Al300<br>B5/H12.5	1519 ± 301	-966 ± 192	SS	1521 ± 324	-968 ± 206	SS	1474 ± 303	-945 ± 195	SS	1485 ± 285	-1074 ± 208	SS	1467 ± 285	-1114 ± 211	SS	1407 ± 264	-1121 ± 208	SS
E20<br>Aml5/B5	1685 ± 335	-800 ± 159	SS	1661 ± 354	-828 ± 176	SS	1592 ± 327	-826 ± 171	SS	1516 ± 290	-1042 ± 201	SS	1489 ± 287	-1091 ± 206	SS	1425 ± 266	-1103 ± 203	SS
E20<br>Aml5/H12.5	1659 ± 327	-826 ± 166	SS	1634 ± 347	-855 ± 183	SS	1564 ± 322	-855 ± 177	SS	1477 ± 283	-1081 ± 209	SS	1450 ± 281	-1131 ± 214	SS	1385 ± 259	-1143 ± 211	SS
E20<br>B5/H12.5	1602 ± 316	-883 ± 176	SS	1577 ± 336	-912 ± 195	SS	1510 ± 310	-909 ± 188	SS	1430 ± 274	-1129 ± 218	SS	1402 ± 272	-1179 ± 223	SS	1338 ± 251	-1190 ± 220	SS
L100<br>Aml5/B5	1504 ± 303	-981 ± 196	SS	1526 ± 329	-963 ± 206	SS	1493 ± 307	-925 ± 194	SS	1557 ± 298	-1002 ± 194	SS	1542 ± 299	-1038 ± 196	SS	1483 ± 278	-1045 ± 193	SS
L100<br>Aml5/H12.5	1570 ± 310	-915 ± 183	SS	1561 ± 332	-928 ± 199	SS	1506 ± 311	-913 ± 189	SS	1513 ± 290	-1046 ± 202	SS	1502 ± 290	-1079 ± 204	SS	1449 ± 270	-1079 ± 199	SS
L100<br>B5/H12.5	1477 ± 293	-1008 ± 201	SS	1483 ± 317	-1006 ± 214	SS	1440 ± 295	-978 ± 202	SS	1460 ± 280	-1098 ± 212	SS	1443 ± 280	-1138 ± 215	SS	1384 ± 260	-1144 ± 211	SS

Al300 = aliskiren 300 mg; **Aml5** = amlodipine 5 mg; **B5** = bisoprolol 5 mg; **E20** = enalapril 20 mg; **H12.5** = hydrochlorothiazide 12.5 mg; **L100** = losartan 100 mg; **SS** = statistically significant (P < 0.00001)

Table S45. *P*-values calculated using the Kolmogorov-Smirnov test for changes in <u>efferent arteriolar resistance</u> in subpopulations (n = 100) with different ACE activity receiving the same regimens. Case 1: c_{ACE} = 7.0 h^{-1} (*LII*), case 2: c_{ACE} = 8.9 h^{-1} (*LID*), case 3: c_{ACE} = 10.8 h^{-1} (*LDD*), case 4: c_{ACE} = 42.3 h^{-1} (*HII*), case 5: c_{ACE} = 54.1 h^{-1} (*HID*), case 6: c_{ACE} = 65.9 h^{-1} (*HDD*). *P*-value for case *i vs*. case *j* is denoted P_{ij}.

Regimens	P_{12}	P_{13}	P_{23}	P_{14}	P_{15}	P_{16}	P_{24}	P_{25}	P_{26}	P_{34}	P_{35}	P_{36}	P_{45}	P_{46}	P_{56}
Al300	0.28093	0.36672	0.90621	SS	SS	SS	SS	SS	SS	SS	SS	SS	0.01581	0.00007	0.28093
E20	0.01008	SS	0.11113	SS	SS	SS	SS	SS	SS	SS	SS	SS	0.00386	SS	0.21055
L100	0.36672	0.58062	0.99376	SS	SS	SS	SS	SS	SS	SS	SS	SS	0.01581	0.00013	0.36672
Aml5	0.90621	0.28093	0.69937	0.90621	0.69937	0.69937	0.46756	0.36672	0.58062	0.07832	0.07832	0.11113	0.90621	0.69937	0.81275
B5	0.28093	0.28093	0.90621	SS	SS	SS	SS	SS	SS	SS	SS	SS	0.01581	0.00007	0.28093
H12.5	0.46756	0.01008	0.15454	SS	SS	SS	SS	SS	SS	SS	SS	SS	0.02431	SS	0.00386
Al300<br>Aml5	0.46756	0.90621	0.96707	SS	SS	SS	SS	SS	SS	SS	SS	SS	0.05410	0.01008	0.81275
Al300<br>B5	0.69937	0.05410	0.46756	0.15454	0.00136	0.00007	0.07832	0.00045	0.00079	0.00232	SS	SS	0.07832	0.01581	0.90621
Al300<br>H12.5	0.69937	0.90621	0.96707	SS	SS	SS	0.00025	SS	SS	0.00004	SS	SS	0.21055	0.07832	0.96707
E20<br>Aml5	0.11113	0.07832	0.96707	SS	SS	SS	SS	SS	SS	SS	SS	SS	0.01581	0.00025	0.69937
E20<br>B5	0.21055	0.21055	0.96707	SS	SS	SS	SS	SS	SS	SS	SS	SS	0.02431	0.00136	0.81275
E20<br>H12.5	0.21055	0.36672	0.96707	SS	SS	SS	SS	SS	SS	SS	SS	SS	0.03663	0.00232	0.90621
L100<br>Aml5	0.69937	0.90621	0.96707	SS	SS	SS	SS	SS	SS	SS	SS	SS	0.05410	0.01008	0.81275
L100<br>B5	0.46756	0.02431	0.46756	0.81275	0.07832	0.01008	0.46756	0.00630	0.01008	0.01581	0.00004	0.00013	0.07832	0.02431	0.90621
L100<br>H12.5	0.81275	0.90621	0.96707	SS	SS	SS	0.00025	SS	SS	0.00004	SS	SS	0.21055	0.07832	0.96707
Al300<br>Aml5/B5	0.69937	0.11113	0.58062	0.15454	0.00386	0.00025	0.05410	0.00136	0.00630	0.00386	SS	0.00004	0.15454	0.02431	0.96707
Al300<br>Aml5/H12.5	0.81275	0.81275	0.90621	0.00079	SS	SS	0.00232	0.00007	0.00045	0.00013	SS	0.00002	0.28093	0.28093	0.96707
Al300<br>B5/H12.5	0.90621	0.36672	0.69937	0.01008	0.00004	0.00002	0.00630	0.00025	0.00025	0.00045	SS	SS	0.21055	0.05410	0.96707
E20<br>Aml5/B5	0.36672	0.46756	0.96707	SS	SS	SS	SS	SS	SS	SS	SS	SS	0.05410	0.02431	0.99376
E20<br>Aml5/H12.5	0.46756	0.58062	0.96707	SS	SS	SS	SS	SS	SS	SS	SS	SS	0.07832	0.03663	0.99376
E20<br>B5/H12.5	0.46756	0.58062	0.96707	SS	SS	SS	SS	SS	SS	SS	SS	SS	0.11113	0.05410	0.99376
L100<br>Aml5/B5	0.69937	0.03663	0.36672	0.81275	0.05410	0.02431	0.21055	0.00630	0.02431	0.01581	0.00007	0.00079	0.15454	0.11113	0.96707
L100<br>Aml5/H12.5	0.81275	0.81275	0.90621	0.00079	SS	SS	0.00232	0.00007	0.00025	0.00013	SS	SS	0.28093	0.15454	0.99376
L100<br>B5/H12.5	0.81275	0.28093	0.69937	0.03663	0.00025	0.00007	0.01581	0.00045	0.00232	0.00045	SS	0.00007	0.21055	0.11113	0.99376

Al300 = aliskiren 300 mg; **Aml5** = amlodipine 5 mg; **B5** = bisoprolol 5 mg; **E20** = enalapril 20 mg; **H12.5** = hydrochlorothiazide 12.5 mg; **L100** = losartan 100 mg; **SS** = statistically significant (P < 0.00001)

Figure S30. Simulated change in glomerular hydrostatic pressure from baseline to week 4 (mean ± SD, n = 100)

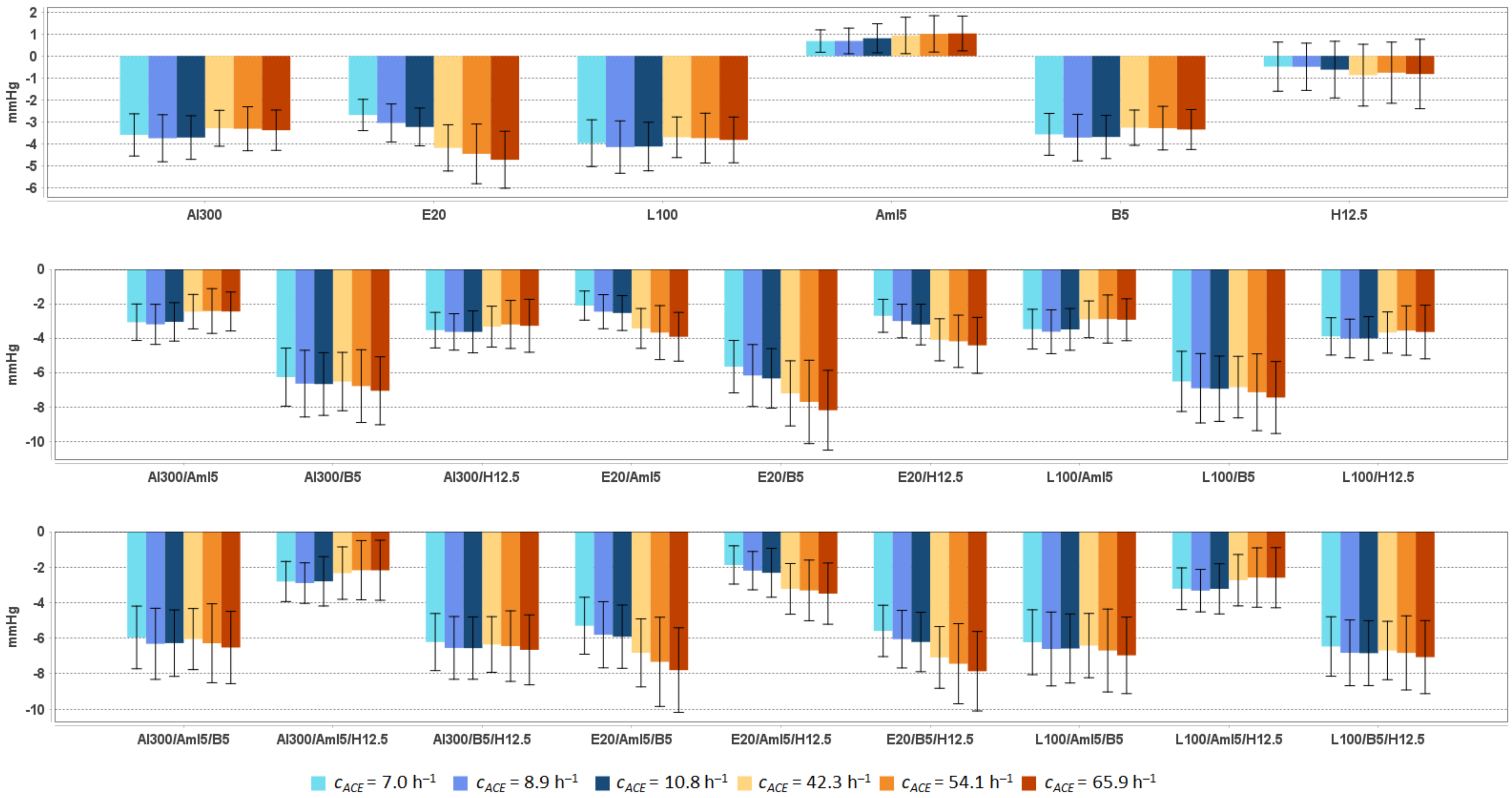

Al300 = aliskiren 300 mg; **Aml5** = amlodipine 5 mg; **B5** = bisoprolol 5 mg; **E20** = enalapril 20 mg; **H12.5** = hydrochlorothiazide 12.5 mg; **L100** = losartan 100 mg

Table S46. Simulated response of glomerular hydrostatic pressure to antihypertensive therapy in virtual hypertensive subpopulations (n = 100) with different ACE activity, including P-values (Kolmogorov-Smirnov test) for endpoint *vs*. baseline; data are presented as mean ± SD in mmHg

Regimens	***LII*** **(c_{ACE} = 7.0 h^{-1})**			***LID*** **(c_{ACE} = 8.9 h^{-1})**			***LDD*** **(c_{ACE} = 10.8 h^{-1})**			***HII*** **(c_{ACE} = 42.3 h^{-1})**			***HID*** **(c_{ACE} = 54.1 h^{-1})**			***HDD*** **(c_{ACE} = 65.9 h^{-1})**		
	**Value**	**Change**	***P***	**Value**	**Change**	***P***	**Value**	**Change**	***P***	**Value**	**Change**	***P***	**Value**	**Change**	***P***	**Value**	**Change**	***P***
Baseline	51.5 ± 2.8	–	–	51.9 ± 3.0	–	–	52.0 ± 3.0	–	–	52.9 ± 3.3	–	–	53.7 ± 4.0	–	–	54.0 ± 3.6	–	–
Al300	47.9 ± 2.4	-3.6 ± 1.0	SS	48.2 ± 2.4	-3.7 ± 1.1	SS	48.3 ± 2.6	-3.7 ± 1.0	SS	49.6 ± 2.9	-3.3 ± 0.8	SS	50.4 ± 3.4	-3.3 ± 1.0	SS	50.6 ± 3.2	-3.4 ± 0.9	SS
E20	48.8 ± 2.5	-2.7 ± 0.7	SS	48.9 ± 2.5	-3.1 ± 0.9	SS	48.8 ± 2.7	-3.2 ± 0.9	SS	48.7 ± 2.8	-4.2 ± 1.1	SS	49.2 ± 3.3	-4.5 ± 1.4	SS	49.3 ± 3.0	-4.7 ± 1.3	SS
L100	47.5 ± 2.4	-4.0 ± 1.1	SS	47.8 ± 2.4	-4.2 ± 1.2	SS	47.9 ± 2.6	-4.1 ± 1.1	SS	49.2 ± 2.9	-3.7 ± 0.9	SS	50.0 ± 3.4	-3.7 ± 1.1	SS	50.2 ± 3.1	-3.8 ± 1.0	SS
Aml5	52.2 ± 2.9	0.7 ± 0.5	0.07832	52.6 ± 3.1	0.7 ± 0.6	0.21055	52.8 ± 3.2	0.8 ± 0.7	0.28093	53.9 ± 3.4	0.9 ± 0.8	0.07832	54.7 ± 4.1	1.0 ± 0.8	0.15454	55.0 ± 3.8	1.0 ± 0.8	0.15454
B5	48.0 ± 2.4	-3.6 ± 1.0	SS	48.2 ± 2.4	-3.7 ± 1.1	SS	48.3 ± 2.6	-3.7 ± 1.0	SS	49.7 ± 2.9	-3.3 ± 0.8	SS	50.4 ± 3.4	-3.3 ± 1.0	SS	50.6 ± 3.2	-3.4 ± 0.9	SS
H12.5	51.0 ± 3.1	-0.5 ± 1.1	0.11113	51.4 ± 3.4	-0.5 ± 1.1	0.15454	51.4 ± 3.4	-0.6 ± 1.3	0.05410	52.0 ± 3.6	-0.9 ± 1.4	0.07832	52.9 ± 4.2	-0.8 ± 1.4	0.28093	53.2 ± 3.9	-0.8 ± 1.6	0.15454
Al300<br>Aml5	48.4 ± 2.5	-3.1 ± 1.1	SS	48.7 ± 2.5	-3.2 ± 1.2	SS	49.0 ± 2.8	-3.1 ± 1.1	SS	50.5 ± 3.0	-2.5 ± 1.0	SS	51.3 ± 3.6	-2.4 ± 1.3	0.00079	51.5 ± 3.3	-2.5 ± 1.1	0.00045
Al300<br>B5	45.3 ± 2.3	-6.3 ± 1.7	SS	45.3 ± 2.2	-6.6 ± 1.9	SS	45.3 ± 2.6	-6.7 ± 1.8	SS	46.4 ± 2.7	-6.5 ± 1.7	SS	46.9 ± 3.0	-6.8 ± 2.1	SS	46.9 ± 2.8	-7.1 ± 2.0	SS
Al300<br>H12.5	48.0 ± 2.6	-3.5 ± 1.0	SS	48.3 ± 2.7	-3.6 ± 1.1	SS	48.4 ± 2.9	-3.6 ± 1.2	SS	49.6 ± 3.2	-3.3 ± 1.2	SS	50.5 ± 3.7	-3.2 ± 1.4	SS	50.7 ± 3.5	-3.3 ± 1.5	SS
E20<br>Aml5	49.4 ± 2.6	-2.1 ± 0.8	SS	49.5 ± 2.6	-2.5 ± 1.0	SS	49.5 ± 2.9	-2.5 ± 1.0	SS	49.5 ± 2.9	-3.4 ± 1.2	SS	50.0 ± 3.4	-3.7 ± 1.6	SS	50.1 ± 3.1	-3.9 ± 1.4	SS
E20<br>B5	45.9 ± 2.3	-5.7 ± 1.5	SS	45.7 ± 2.2	-6.2 ± 1.8	SS	45.7 ± 2.5	-6.3 ± 1.7	SS	45.7 ± 2.7	-7.2 ± 1.9	SS	46.0 ± 2.9	-7.7 ± 2.4	SS	45.8 ± 2.8	-8.2 ± 2.3	SS
E20<br>H12.5	48.8 ± 2.7	-2.7 ± 1.0	SS	48.9 ± 2.8	-3.0 ± 1.0	SS	48.8 ± 3.0	-3.2 ± 1.2	SS	48.8 ± 3.1	-4.1 ± 1.2	SS	49.5 ± 3.6	-4.2 ± 1.5	SS	49.6 ± 3.4	-4.4 ± 1.6	SS
L100<br>Aml5	48.0 ± 2.4	-3.5 ± 1.2	SS	48.3 ± 2.4	-3.6 ± 1.3	SS	48.5 ± 2.8	-3.5 ± 1.2	SS	50.0 ± 3.0	-2.9 ± 1.1	SS	50.8 ± 3.6	-2.9 ± 1.4	0.00007	51.1 ± 3.3	-2.9 ± 1.2	SS
L100<br>B5	45.0 ± 2.3	-6.5 ± 1.8	SS	45.0 ± 2.2	-6.9 ± 2.0	SS	45.1 ± 2.6	-6.9 ± 1.9	SS	46.1 ± 2.7	-6.9 ± 1.8	SS	46.5 ± 2.9	-7.2 ± 2.2	SS	46.5 ± 2.8	-7.5 ± 2.1	SS
L100<br>H12.5	47.6 ± 2.5	-3.9 ± 1.1	SS	47.9 ± 2.6	-4.0 ± 1.1	SS	48.0 ± 2.9	-4.0 ± 1.3	SS	49.2 ± 3.1	-3.7 ± 1.2	SS	50.1 ± 3.7	-3.6 ± 1.4	SS	50.3 ± 3.5	-3.6 ± 1.6	SS
Al300<br>Aml5/B5	45.6 ± 2.3	-6.0 ± 1.8	SS	45.6 ± 2.2	-6.3 ± 2.0	SS	45.7 ± 2.6	-6.3 ± 1.9	SS	46.9 ± 2.7	-6.0 ± 1.7	SS	47.4 ± 3.1	-6.3 ± 2.2	SS	47.5 ± 2.9	-6.5 ± 2.0	SS
Al300<br>Aml5/H12.5	48.7 ± 2.7	-2.8 ± 1.1	SS	49.0 ± 2.8	-2.9 ± 1.1	SS	49.2 ± 3.1	-2.8 ± 1.4	SS	50.6 ± 3.3	-2.3 ± 1.5	SS	51.5 ± 4.0	-2.2 ± 1.7	0.00136	51.8 ± 3.7	-2.2 ± 1.7	0.00136
Al300<br>B5/H12.5	45.3 ± 2.3	-6.2 ± 1.6	SS	45.4 ± 2.3	-6.5 ± 1.8	SS	45.4 ± 2.7	-6.6 ± 1.8	SS	46.6 ± 2.8	-6.4 ± 1.6	SS	47.2 ± 3.2	-6.4 ± 2.0	SS	47.3 ± 3.1	-6.7 ± 2.0	SS
E20<br>Aml5/B5	46.2 ± 2.3	-5.3 ± 1.6	SS	46.1 ± 2.2	-5.8 ± 1.9	SS	46.1 ± 2.6	-5.9 ± 1.8	SS	46.1 ± 2.7	-6.8 ± 1.9	SS	46.4 ± 3.0	-7.3 ± 2.5	SS	46.2 ± 2.9	-7.8 ± 2.4	SS
E20<br>Aml5/H12.5	49.6 ± 2.8	-1.9 ± 1.1	SS	49.7 ± 2.9	-2.2 ± 1.1	SS	49.7 ± 3.2	-2.3 ± 1.4	SS	49.7 ± 3.2	-3.2 ± 1.4	SS	50.4 ± 3.7	-3.3 ± 1.7	SS	50.5 ± 3.5	-3.5 ± 1.7	SS
E20<br>B5/H12.5	45.9 ± 2.4	-5.6 ± 1.4	SS	45.9 ± 2.3	-6.1 ± 1.6	SS	45.8 ± 2.7	-6.2 ± 1.7	SS	45.8 ± 2.7	-7.1 ± 1.7	SS	46.3 ± 3.0	-7.4 ± 2.3	SS	46.1 ± 3.0	-7.9 ± 2.2	SS
L100<br>Aml5/B5	45.3 ± 2.3	-6.2 ± 1.8	SS	45.3 ± 2.2	-6.6 ± 2.1	SS	45.4 ± 2.6	-6.6 ± 1.9	SS	46.5 ± 2.7	-6.4 ± 1.8	SS	47.0 ± 3.0	-6.7 ± 2.3	SS	47.0 ± 2.9	-7.0 ± 2.2	SS
L100<br>Aml5/H12.5	48.3 ± 2.6	-3.2 ± 1.2	SS	48.6 ± 2.7	-3.3 ± 1.2	SS	48.8 ± 3.1	-3.2 ± 1.4	SS	50.2 ± 3.3	-2.7 ± 1.4	SS	51.1 ± 3.9	-2.6 ± 1.7	0.00007	51.4 ± 3.6	-2.6 ± 1.7	0.00025
L100<br>B5/H12.5	45.1 ± 2.3	-6.5 ± 1.7	SS	45.1 ± 2.3	-6.8 ± 1.9	SS	45.2 ± 2.7	-6.8 ± 1.8	SS	46.2 ± 2.7	-6.7 ± 1.6	SS	46.9 ± 3.1	-6.8 ± 2.1	SS	46.9 ± 3.0	-7.1 ± 2.1	SS

Al300 = aliskiren 300 mg; **Aml5** = amlodipine 5 mg; **B5** = bisoprolol 5 mg; **E20** = enalapril 20 mg; **H12.5** = hydrochlorothiazide 12.5 mg; **L100** = losartan 100 mg; **SS** = statistically significant (P < 0.00001)

Table S47. *P*-values calculated using the Kolmogorov-Smirnov test for changes in glomerular hydrostatic pressure in subpopulations (n = 100) with different ACE activity receiving the same regimens. Case 1: c_{ACE} = 7.0 h^{-1} (*LII*), case 2: c_{ACE} = 8.9 h^{-1} (*LID*), case 3: c_{ACE} = 10.8 h^{-1} (*LDD*), case 4: c_{ACE} = 42.3 h^{-1} (*HII*), case 5: c_{ACE} = 54.1 h^{-1} (*HID*), case 6: c_{ACE} = 65.9 h^{-1} (*HDD*). *P*-value for case *i vs*. case *j* is denoted P_{ij}.

Regimens	P_{12}	P_{13}	P_{23}	P_{14}	P_{15}	P_{16}	P_{24}	P_{25}	P_{26}	P_{34}	P_{35}	P_{36}	P_{45}	P_{46}	P_{56}
Al300	0.46756	0.69937	0.96707	0.15454	0.15454	0.36672	0.01581	0.07832	0.21055	0.01008	0.02431	0.11113	0.90621	0.46756	0.81275
E20	0.03663	0.00013	0.15454	SS	SS	SS	SS	SS	SS	SS	SS	SS	0.46756	0.01581	0.21055
L100	0.46756	0.58062	0.96707	0.36672	0.21055	0.46756	0.02431	0.21055	0.36672	0.03663	0.05410	0.28093	0.90621	0.46756	0.81275
Aml5	0.96707	0.36672	0.15454	0.21055	0.01008	0.00079	0.15454	0.00232	0.00079	0.58062	0.05410	0.00630	0.36672	0.11113	0.58062
B5	0.58062	0.69937	0.90621	0.15454	0.11113	0.28093	0.01581	0.07832	0.21055	0.01008	0.02431	0.11113	0.90621	0.46756	0.90621
H12.5	0.46756	0.01581	0.15454	0.01008	0.15454	0.15454	0.05410	0.28093	0.03663	0.36672	0.81275	0.58062	0.46756	0.11113	0.90621
Al300<br>Aml5	0.46756	0.90621	0.90621	0.00079	0.00013	0.00079	0.00004	0.00007	0.00045	0.00007	0.00013	0.00045	0.69937	0.90621	0.96707
Al300<br>B5	0.21055	0.21055	0.58062	0.46756	0.15454	0.01581	0.69937	0.69937	0.03663	0.58062	0.96707	0.21055	0.69937	0.11113	0.28093
Al300<br>H12.5	0.46756	0.46756	0.81275	0.28093	0.21055	0.01008	0.11113	0.05410	0.00136	0.15454	0.05410	0.00630	0.69937	0.15454	0.46756
E20<br>Aml5	0.01581	0.00079	0.46756	SS	SS	SS	SS	SS	SS	SS	SS	SS	0.28093	0.00232	0.02431
E20<br>B5	0.15454	0.02431	0.28093	SS	SS	SS	0.00025	0.00002	SS	0.01008	0.00079	SS	0.46756	0.01008	0.21055
E20<br>H12.5	0.00386	0.00045	0.36672	SS	SS	SS	SS	SS	SS	SS	SS	SS	0.46756	0.58062	0.46756
L100<br>Aml5	0.28093	0.90621	0.96707	0.00232	0.00136	0.01008	0.00025	0.00079	0.00386	0.00079	0.00079	0.00386	0.69937	0.81275	0.58062
L100<br>B5	0.21055	0.21055	0.58062	0.28093	0.07832	0.00386	0.58062	0.58062	0.03663	0.58062	0.99376	0.11113	0.58062	0.11113	0.28093
L100<br>H12.5	0.46756	0.58062	0.90621	0.58062	0.28093	0.01581	0.11113	0.11113	0.00232	0.15454	0.07832	0.02431	0.96707	0.21055	0.58062
Al300<br>Aml5/B5	0.28093	0.28093	0.99376	0.96707	0.69937	0.03663	0.58062	0.90621	0.15454	0.46756	0.90621	0.36672	0.58062	0.07832	0.28093
Al300<br>Aml5/H12.5	0.46756	0.58062	0.58062	0.05410	0.01581	0.00630	0.00136	0.00025	0.00025	0.05410	0.00630	0.00232	0.46756	0.21055	0.96707
Al300<br>B5/H12.5	0.46756	0.21055	0.90621	0.90621	0.46756	0.15454	0.69937	0.90621	0.81275	0.15454	0.90621	0.90621	0.81275	0.15454	0.90621
E20<br>Aml5/B5	0.05410	0.01008	0.81275	SS	SS	SS	0.00079	0.00013	SS	0.00386	0.00079	SS	0.36672	0.00630	0.11113
E20<br>Aml5/H12.5	0.00232	SS	0.28093	SS	SS	SS	SS	SS	SS	0.00007	SS	SS	0.81275	0.69937	0.81275
E20<br>B5/H12.5	0.21055	0.01008	0.69937	SS	SS	SS	0.00007	0.00002	SS	0.00386	0.00232	0.00004	0.36672	0.00630	0.46756
L100<br>Aml5/B5	0.28093	0.21055	0.90621	0.69937	0.46756	0.01581	0.58062	0.96707	0.07832	0.69937	0.96707	0.15454	0.58062	0.07832	0.21055
L100<br>Aml5/H12.5	0.36672	0.58062	0.46756	0.02431	0.02431	0.00630	0.00045	0.00079	0.00025	0.05410	0.01581	0.00136	0.90621	0.36672	0.96707
L100<br>B5/H12.5	0.36672	0.11113	0.90621	0.58062	0.28093	0.05410	0.58062	0.69937	0.36672	0.15454	0.81275	0.58062	0.58062	0.11113	0.81275

Al300 = aliskiren 300 mg; **Aml5** = amlodipine 5 mg; **B5** = bisoprolol 5 mg; **E20** = enalapril 20 mg; **H12.5** = hydrochlorothiazide 12.5 mg; **L100** = losartan 100 mg; **SS** = statistically significant (P < 0.00001)

Figure S31. Simulated change in plasma sodium from baseline to week 4 (mean ± SD, n = 100)

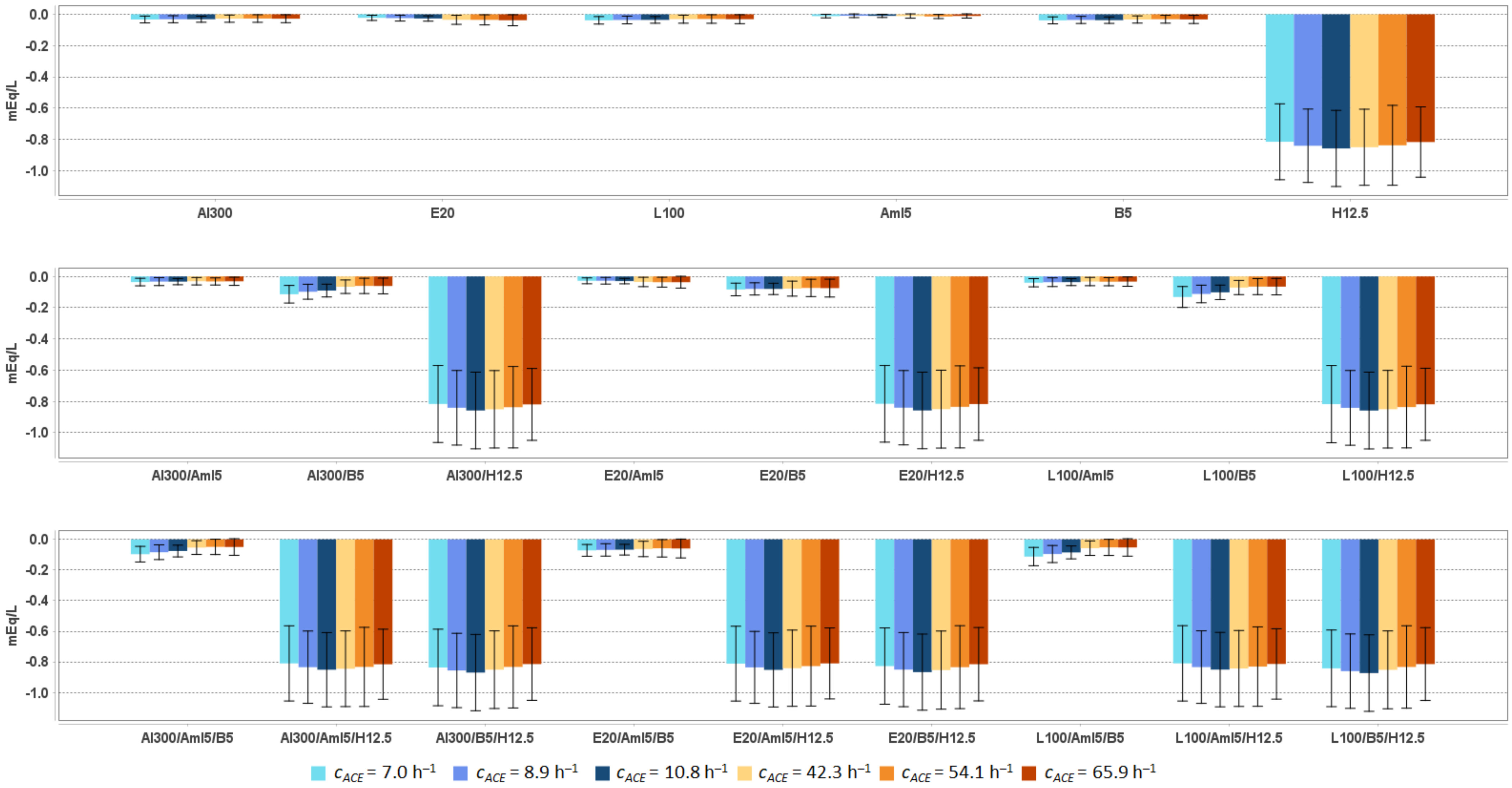

Al300 = aliskiren 300 mg; **Aml5** = amlodipine 5 mg; **B5** = bisoprolol 5 mg; **E20** = enalapril 20 mg; **H12.5** = hydrochlorothiazide 12.5 mg; **L100** = losartan 100 mg

Table S48. Simulated response of plasma sodium to antihypertensive therapy in virtual hypertensive subpopulations (n = 100) with different ACE activity, including P-values (Kolmogorov-Smirnov test) for endpoint *vs*. baseline; data are presented as mean ± SD in mEq/L

Regimens	***LII*** **(c_{ACE} = 7.0 h^{-1})**			***LID*** **(c_{ACE} = 8.9 h^{-1})**			***LDD*** **(c_{ACE} = 10.8 h^{-1})**			***HII*** **(c_{ACE} = 42.3 h^{-1})**			***HID*** **(c_{ACE} = 54.1 h^{-1})**			***HDD*** **(c_{ACE} = 65.9 h^{-1})**		
	**Value**	**Change**	***P***	**Value**	**Change**	***P***	**Value**	**Change**	***P***	**Value**	**Change**	***P***	**Value**	**Change**	***P***	**Value**	**Change**	***P***
Baseline	143.62 ± 1.29	–	–	143.65 ± 1.24	–	–	143.72 ± 1.27	–	–	143.47 ± 1.37	–	–	143.38 ± 1.34	–	–	143.45 ± 1.40	–	–
Al300	143.59 ± 1.28	-0.03 ± 0.02	0.99963	143.62 ± 1.24	-0.03 ± 0.02	1.00000	143.69 ± 1.27	-0.03 ± 0.02	0.99963	143.44 ± 1.37	-0.03 ± 0.02	1.00000	143.35 ± 1.34	-0.03 ± 0.02	0.99963	143.43 ± 1.40	-0.03 ± 0.03	1.00000
E20	143.60 ± 1.28	-0.02 ± 0.02	1.00000	143.62 ± 1.24	-0.02 ± 0.02	1.00000	143.69 ± 1.27	-0.03 ± 0.02	1.00000	143.43 ± 1.37	-0.04 ± 0.03	0.99963	143.34 ± 1.34	-0.04 ± 0.03	0.99376	143.42 ± 1.40	-0.04 ± 0.04	1.00000
L100	143.58 ± 1.28	-0.04 ± 0.02	0.99963	143.61 ± 1.24	-0.04 ± 0.02	0.99963	143.68 ± 1.27	-0.04 ± 0.02	0.99376	143.44 ± 1.37	-0.03 ± 0.03	0.99963	143.35 ± 1.34	-0.03 ± 0.03	0.99963	143.42 ± 1.40	-0.03 ± 0.03	1.00000
Aml5	143.61 ± 1.29	-0.01 ± 0.01	1.00000	143.64 ± 1.24	-0.01 ± 0.01	1.00000	143.71 ± 1.28	-0.01 ± 0.01	1.00000	143.46 ± 1.38	-0.01 ± 0.01	1.00000	143.36 ± 1.34	-0.01 ± 0.01	1.00000	143.44 ± 1.40	-0.01 ± 0.01	1.00000
B5	143.58 ± 1.28	-0.04 ± 0.02	0.99963	143.61 ± 1.24	-0.04 ± 0.02	1.00000	143.68 ± 1.27	-0.04 ± 0.02	0.99963	143.44 ± 1.37	-0.03 ± 0.02	0.99963	143.35 ± 1.34	-0.03 ± 0.03	0.99963	143.42 ± 1.40	-0.03 ± 0.03	1.00000
H12.5	142.81 ± 1.28	-0.82 ± 0.24	0.00013	142.81 ± 1.30	-0.84 ± 0.23	0.00025	142.86 ± 1.25	-0.86 ± 0.24	SS	142.62 ± 1.36	-0.85 ± 0.24	0.00045	142.54 ± 1.41	-0.84 ± 0.26	0.00232	142.64 ± 1.39	-0.82 ± 0.22	0.00025
Al300 Aml5	143.59 ± 1.28	-0.04 ± 0.02	1.00000	143.62 ± 1.25	-0.03 ± 0.03	1.00000	143.69 ± 1.27	-0.03 ± 0.02	0.99963	143.44 ± 1.37	-0.03 ± 0.02	1.00000	143.35 ± 1.34	-0.03 ± 0.02	1.00000	143.42 ± 1.40	-0.03 ± 0.03	1.00000
Al300 B5	143.51 ± 1.28	-0.11 ± 0.06	0.90621	143.55 ± 1.24	-0.10 ± 0.05	0.81275	143.63 ± 1.27	-0.09 ± 0.04	0.90621	143.40 ± 1.36	-0.07 ± 0.04	0.99376	143.32 ± 1.34	-0.06 ± 0.05	0.96707	143.39 ± 1.40	-0.06 ± 0.05	0.99963
Al300 H12.5	142.80 ± 1.27	-0.82 ± 0.25	0.00013	142.81 ± 1.30	-0.84 ± 0.24	0.00025	142.86 ± 1.25	-0.86 ± 0.25	SS	142.62 ± 1.36	-0.85 ± 0.25	0.00045	142.54 ± 1.41	-0.84 ± 0.26	0.00232	142.64 ± 1.39	-0.82 ± 0.23	0.00045
E20 Aml5	143.59 ± 1.28	-0.03 ± 0.02	1.00000	143.62 ± 1.24	-0.03 ± 0.02	1.00000	143.69 ± 1.27	-0.03 ± 0.02	0.99963	143.43 ± 1.37	-0.04 ± 0.03	0.99963	143.34 ± 1.34	-0.04 ± 0.03	0.99963	143.42 ± 1.40	-0.04 ± 0.04	1.00000
E20 B5	143.54 ± 1.28	-0.08 ± 0.04	0.96707	143.57 ± 1.24	-0.08 ± 0.04	0.81275	143.64 ± 1.27	-0.08 ± 0.04	0.96707	143.39 ± 1.36	-0.08 ± 0.05	0.96707	143.30 ± 1.34	-0.07 ± 0.06	0.96707	143.38 ± 1.40	-0.07 ± 0.06	0.99963
E20 H12.5	142.81 ± 1.27	-0.81 ± 0.25	0.00013	142.81 ± 1.30	-0.84 ± 0.24	0.00025	142.86 ± 1.25	-0.86 ± 0.24	SS	142.62 ± 1.36	-0.85 ± 0.25	0.00045	142.54 ± 1.41	-0.83 ± 0.26	0.00232	142.64 ± 1.39	-0.82 ± 0.23	0.00045
L100 Aml5	143.58 ± 1.28	-0.04 ± 0.03	0.99963	143.61 ± 1.25	-0.04 ± 0.03	1.00000	143.68 ± 1.27	-0.04 ± 0.02	0.99376	143.44 ± 1.37	-0.03 ± 0.03	1.00000	143.34 ± 1.34	-0.03 ± 0.03	1.00000	143.42 ± 1.40	-0.03 ± 0.03	1.00000
L100 B5	143.49 ± 1.28	-0.13 ± 0.07	0.81275	143.54 ± 1.24	-0.11 ± 0.06	0.81275	143.62 ± 1.26	-0.10 ± 0.05	0.90621	143.40 ± 1.36	-0.07 ± 0.05	0.99376	143.31 ± 1.34	-0.07 ± 0.05	0.96707	143.39 ± 1.40	-0.07 ± 0.05	0.99963
L100 H12.5	142.80 ± 1.27	-0.82 ± 0.25	0.00013	142.81 ± 1.30	-0.84 ± 0.24	0.00025	142.86 ± 1.25	-0.86 ± 0.25	SS	142.62 ± 1.36	-0.85 ± 0.25	0.00045	142.54 ± 1.41	-0.84 ± 0.26	0.00232	142.64 ± 1.39	-0.82 ± 0.23	0.00045
Al300 Aml5/B5	143.52 ± 1.28	-0.10 ± 0.05	0.96707	143.57 ± 1.25	-0.08 ± 0.05	0.81275	143.64 ± 1.27	-0.08 ± 0.04	0.96707	143.42 ± 1.36	-0.05 ± 0.05	0.99963	143.33 ± 1.34	-0.05 ± 0.05	0.99376	143.40 ± 1.40	-0.05 ± 0.05	0.99963
Al300 Aml5/H12.5	142.81 ± 1.28	-0.81 ± 0.25	0.00025	142.81 ± 1.31	-0.83 ± 0.24	0.00025	142.87 ± 1.25	-0.85 ± 0.24	SS	142.62 ± 1.36	-0.84 ± 0.25	0.00045	142.55 ± 1.41	-0.83 ± 0.26	0.00232	142.64 ± 1.39	-0.82 ± 0.23	0.00045
Al300 B5/H12.5	142.78 ± 1.27	-0.84 ± 0.25	0.00004	142.79 ± 1.31	-0.86 ± 0.24	0.00025	142.85 ± 1.25	-0.87 ± 0.25	SS	142.62 ± 1.36	-0.85 ± 0.25	0.00045	142.55 ± 1.41	-0.83 ± 0.27	0.00232	142.64 ± 1.40	-0.81 ± 0.24	0.00045
E20 Aml5/B5	143.55 ± 1.28	-0.07 ± 0.04	0.96707	143.58 ± 1.25	-0.07 ± 0.04	0.81275	143.65 ± 1.27	-0.07 ± 0.03	0.96707	143.41 ± 1.36	-0.06 ± 0.05	0.99376	143.32 ± 1.34	-0.06 ± 0.06	0.99376	143.39 ± 1.40	-0.06 ± 0.06	0.99963
E20 Aml5/H12.5	142.81 ± 1.28	-0.81 ± 0.24	0.00025	142.81 ± 1.31	-0.84 ± 0.23	0.00025	142.87 ± 1.25	-0.85 ± 0.24	SS	142.63 ± 1.36	-0.84 ± 0.25	0.00045	142.55 ± 1.41	-0.83 ± 0.26	0.00232	142.64 ± 1.40	-0.81 ± 0.23	0.00079
E20 B5/H12.5	142.79 ± 1.27	-0.83 ± 0.25	0.00007	142.80 ± 1.31	-0.85 ± 0.24	0.00025	142.85 ± 1.25	-0.87 ± 0.25	SS	142.62 ± 1.36	-0.85 ± 0.26	0.00045	142.54 ± 1.41	-0.83 ± 0.27	0.00232	142.64 ± 1.40	-0.82 ± 0.24	0.00045
L100 Aml5/B5	143.51 ± 1.28	-0.11 ± 0.06	0.90621	143.55 ± 1.25	-0.10 ± 0.06	0.81275	143.63 ± 1.27	-0.09 ± 0.04	0.96707	143.41 ± 1.36	-0.06 ± 0.05	0.99963	143.33 ± 1.34	-0.05 ± 0.05	0.99376	143.40 ± 1.40	-0.05 ± 0.06	0.99963
L100 Aml5/H12.5	142.81 ± 1.28	-0.81 ± 0.25	0.00025	142.81 ± 1.31	-0.83 ± 0.24	0.00025	142.87 ± 1.26	-0.85 ± 0.24	SS	142.63 ± 1.36	-0.84 ± 0.25	0.00045	142.55 ± 1.41	-0.83 ± 0.26	0.00232	142.64 ± 1.39	-0.81 ± 0.23	0.00045
L100 B5/H12.5	142.78 ± 1.28	-0.84 ± 0.25	0.00004	142.79 ± 1.31	-0.86 ± 0.24	0.00025	142.85 ± 1.25	-0.87 ± 0.25	SS	142.62 ± 1.36	-0.85 ± 0.25	0.00045	142.54 ± 1.41	-0.83 ± 0.27	0.00232	142.64 ± 1.40	-0.81 ± 0.24	0.00045

Al300 = aliskiren 300 mg; **Aml5** = amlodipine 5 mg; **B5** = bisoprolol 5 mg; **E20** = enalapril 20 mg; **H12.5** = hydrochlorothiazide 12.5 mg; **L100** = losartan 100 mg; **SS** = statistically significant (P < 0.00001)

Table S49. *P*-values calculated using the Kolmogorov-Smirnov test for changes in plasma sodium in subpopulations (n = 100) with different ACE activity receiving the same regimens. Case 1: c_{ACE} = 7.0 h^{-1} (*LII*), case 2: c_{ACE} = 8.9 h^{-1} (*LID*), case 3: c_{ACE} = 10.8 h^{-1} (*LDD*), case 4: c_{ACE} = 42.3 h^{-1} (*HII*), case 5: c_{ACE} = 54.1 h^{-1} (*HID*), case 6: c_{ACE} =65.9 h^{-1} (*HDD*). *P*-value for case *i* *vs*. case *j* is denoted P_{ij}.

Regimens	P_{12}	P_{13}	P_{23}	P_{14}	P_{15}	P_{16}	P_{24}	P_{25}	P_{26}	P_{34}	P_{35}	P_{36}	P_{45}	P_{46}	P_{56}
Al300	0.90621	0.58062	0.90621	0.11113	0.01581	0.21055	0.21055	0.07832	0.46756	0.07832	0.01581	0.28093	0.81275	0.69937	0.28093
E20	0.58062	0.28093	0.58062	0.00079	0.00007	0.00002	0.02431	0.00232	0.00025	0.03663	0.00232	0.00079	0.81275	0.69937	0.15454
L100	0.96707	0.46756	0.81275	0.05410	0.00386	0.15454	0.15454	0.05410	0.28093	0.05410	0.00630	0.28093	0.81275	0.69937	0.28093
Aml5	0.21055	0.69937	0.69937	0.46756	0.28093	0.58062	0.46756	0.07832	0.46756	0.58062	0.07832	0.90621	0.28093	0.96707	0.11113
B5	0.81275	0.81275	0.90621	0.21055	0.01581	0.15454	0.36672	0.15454	0.58062	0.21055	0.02431	0.11113	0.81275	0.81275	0.58062
H12.5	0.36672	0.15454	0.69937	0.46756	0.69937	0.81275	0.81275	0.99376	0.81275	0.90621	0.58062	0.28093	0.90621	0.28093	0.90621
Al300 Aml5	0.58062	0.69937	0.58062	0.15454	0.15454	0.28093	0.58062	0.69937	0.81275	0.21055	0.15454	0.81275	0.96707	0.69937	0.90621
Al300 B5	0.11113	0.11113	0.28093	SS	SS	SS	0.00025	SS	0.00013	0.00136	SS	0.00025	0.36672	0.96707	0.28093
Al300 H12.5	0.58062	0.11113	0.69937	0.36672	0.69937	0.81275	0.69937	0.96707	0.90621	0.81275	0.58062	0.36672	0.69937	0.46756	0.90621
E20 Aml5	0.90621	0.36672	0.36672	0.00386	0.00232	0.00045	0.03663	0.01581	0.00232	0.01008	0.01008	0.00136	0.96707	0.58062	0.69937
E20 B5	0.58062	0.90621	0.90621	0.11113	0.00386	0.05410	0.58062	0.03663	0.36672	0.21055	0.00232	0.07832	0.46756	0.90621	0.46756
E20 H12.5	0.58062	0.15454	0.69937	0.36672	0.69937	0.81275	0.81275	0.96707	0.81275	0.81275	0.58062	0.28093	0.69937	0.46756	0.81275
L100 Aml5	0.58062	0.58062	0.58062	0.02431	0.05410	0.21055	0.36672	0.21055	0.46756	0.07832	0.05410	0.46756	0.99376	0.58062	0.58062
L100 B5	0.07832	0.03663	0.21055	SS	SS	SS	0.00013	SS	SS	0.00025	SS	0.00004	0.28093	0.81275	0.36672
L100 H12.5	0.58062	0.11113	0.69937	0.36672	0.69937	0.81275	0.81275	0.96707	0.90621	0.81275	0.58062	0.28093	0.69937	0.46756	0.81275
Al300 Aml5/B5	0.05410	0.01008	0.36672	SS	SS	SS	0.00079	SS	0.00136	0.00013	SS	0.00025	0.58062	0.96707	0.28093
Al300 Aml5/H12.5	0.46756	0.07832	0.69937	0.36672	0.58062	0.81275	0.90621	0.90621	0.96707	0.81275	0.58062	0.36672	0.69937	0.58062	0.69937
Al300 B5/H12.5	0.69937	0.11113	0.46756	0.81275	0.96707	0.69937	0.96707	0.81275	0.36672	0.58062	0.36672	0.11113	0.69937	0.36672	0.81275
E20 Aml5/B5	0.58062	0.90621	0.90621	0.00630	0.00013	0.00630	0.07832	0.00386	0.11113	0.02431	0.00045	0.02431	0.46756	0.96707	0.36672
E20 Aml5/H12.5	0.36672	0.07832	0.69937	0.46756	0.69937	0.90621	0.90621	0.96707	0.81275	0.81275	0.58062	0.28093	0.58062	0.46756	0.81275
E20 B5/H12.5	0.58062	0.07832	0.58062	0.36672	0.90621	0.81275	0.81275	0.81275	0.58062	0.58062	0.36672	0.11113	0.69937	0.46756	0.90621
L100 Aml5/B5	0.03663	0.00386	0.46756	SS	SS	SS	0.00013	SS	0.00013	0.00007	SS	0.00013	0.46756	0.96707	0.36672
L100 Aml5/H12.5	0.46756	0.07832	0.69937	0.46756	0.58062	0.81275	0.90621	0.96707	0.90621	0.90621	0.58062	0.36672	0.69937	0.58062	0.81275
L100 B5/H12.5	0.81275	0.11113	0.36672	0.81275	0.96707	0.69937	0.96707	0.81275	0.36672	0.46756	0.28093	0.11113	0.69937	0.36672	0.81275

Al300 = aliskiren 300 mg; **Aml5** = amlodipine 5 mg; **B5** = bisoprolol 5 mg; **E20** = enalapril 20 mg; **H12.5** = hydrochlorothiazide 12.5 mg; **L100** = losartan 100 mg; **SS** = statistically significant (P < 0.00001)

Figure S32. Simulated change in <u>plasma renin activity</u> from baseline to week 4 (mean ± SD, *n* = 100)

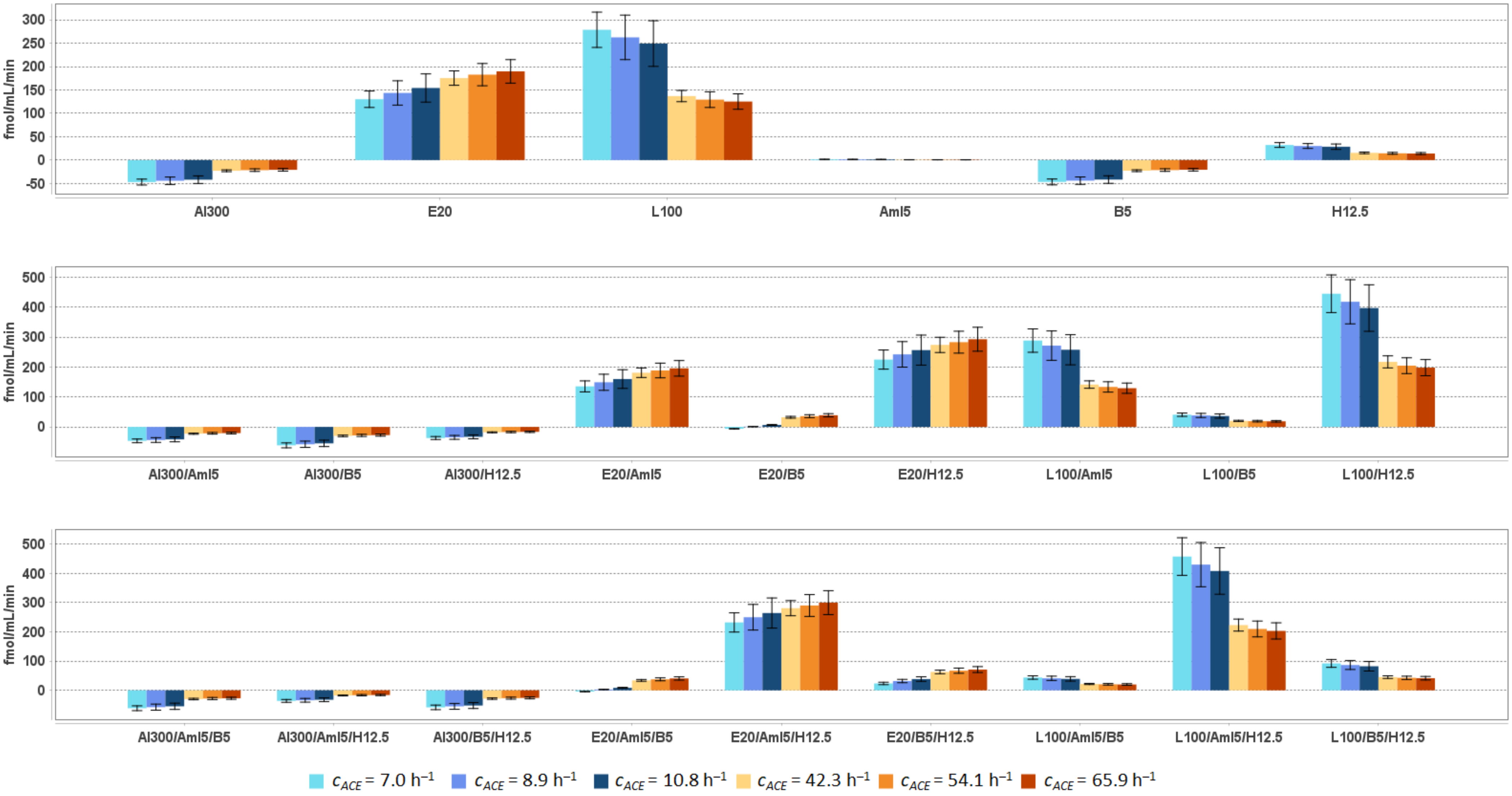

Al300 = aliskiren 300 mg; **Aml5** = amlodipine 5 mg; **B5** = bisoprolol 5 mg; **E20** = enalapril 20 mg; **H12.5** = hydrochlorothiazide 12.5 mg; **L100** = losartan 100 mg

Table S50. Simulated response of <u>plasma renin activity</u> to antihypertensive therapy in virtual hypertensive subpopulations (n = 100) with different ACE activity, including P-values (Kolmogorov-Smirnov test) for endpoint *vs*. baseline; data are presented as mean ± SD in fmol/mL/min

Regimens	***LII*** **(c_{ACE} = 7.0 h^{-1})**			***LID*** **(c_{ACE} = 8.9 h^{-1})**			***LDD*** **(c_{ACE} = 10.8 h^{-1})**			***HII*** **(c_{ACE} = 42.3 h^{-1})**			***HID*** **(c_{ACE} = 54.1 h^{-1})**			***HDD*** **(c_{ACE} = 65.9 h^{-1})**		
	**Value**	**Change**	***P***	**Value**	**Change**	***P***	**Value**	**Change**	***P***	**Value**	**Change**	***P***	**Value**	**Change**	***P***	**Value**	**Change**	***P***
Baseline	68.1 ± 9.1	–	–	64.1 ± 11.6	–	–	60.7 ± 11.8	–	–	33.2 ± 2.8	–	–	31.3 ± 4.0	–	–	30.3 ± 3.9	–	–
Al300	21.0 ± 2.8	-47.1 ± 6.3	SS	19.8 ± 3.6	-44.3 ± 8.0	SS	18.8 ± 3.7	-42.0 ± 8.2	SS	10.3 ± 0.9	-22.9 ± 1.9	SS	9.7 ± 1.3	-21.6 ± 2.8	SS	9.4 ± 1.2	-20.8 ± 2.7	SS
E20	198.3 ± 26.7	130.2 ± 17.6	SS	207.7 ± 37.6	143.7 ± 26.0	SS	215.0 ± 42.0	154.3 ± 30.2	SS	208.6 ± 18.2	175.4 ± 15.4	SS	214.1 ± 27.8	182.8 ± 23.8	SS	220.1 ± 29.1	189.8 ± 25.1	SS
L100	346.9 ± 46.9	278.8 ± 37.8	SS	326.6 ± 59.1	262.6 ± 47.6	SS	310.1 ± 60.7	249.3 ± 48.9	SS	170.2 ± 14.8	137.0 ± 12.0	SS	160.6 ± 20.8	129.4 ± 16.8	SS	155.5 ± 20.4	125.3 ± 16.5	SS
Aml5	69.6 ± 9.3	1.6 ± 0.3	0.69937	65.5 ± 11.8	1.4 ± 0.3	0.90621	62.1 ± 12.1	1.3 ± 0.3	0.58062	33.9 ± 2.9	0.7 ± 0.1	0.21055	32.0 ± 4.1	0.7 ± 0.1	0.11113	30.9 ± 4.0	0.6 ± 0.1	0.58062
B5	21.3 ± 2.9	-46.8 ± 6.3	SS	20.1 ± 3.6	-44.0 ± 7.9	SS	19.0 ± 3.7	-41.7 ± 8.1	SS	10.4 ± 0.9	-22.8 ± 1.9	SS	9.9 ± 1.3	-21.4 ± 2.7	SS	9.5 ± 1.3	-20.7 ± 2.7	SS
H12.5	100.3 ± 13.9	32.2 ± 5.1	SS	94.2 ± 16.5	30.1 ± 5.2	SS	89.3 ± 17.4	28.6 ± 5.7	SS	48.7 ± 4.4	15.5 ± 1.7	SS	45.8 ± 5.8	14.5 ± 2.0	SS	44.2 ± 5.9	14.0 ± 2.1	SS
Al300<br>Aml5	21.6 ± 2.9	-46.5 ± 6.2	SS	20.3 ± 3.7	-43.8 ± 7.9	SS	19.3 ± 3.8	-41.5 ± 8.1	SS	10.6 ± 0.9	-22.6 ± 1.9	SS	10.0 ± 1.3	-21.3 ± 2.7	SS	9.6 ± 1.3	-20.6 ± 2.7	SS
Al300<br>B5	6.6 ± 0.9	-61.5 ± 8.2	SS	6.2 ± 1.1	-57.9 ± 10.4	SS	5.9 ± 1.2	-54.9 ± 10.7	SS	3.2 ± 0.3	-30.0 ± 2.6	SS	3.1 ± 0.4	-28.2 ± 3.6	SS	3.0 ± 0.4	-27.3 ± 3.5	SS
Al300<br>H12.5	31.0 ± 4.3	-37.0 ± 4.9	SS	29.2 ± 5.2	-34.9 ± 6.5	SS	27.7 ± 5.4	-33.0 ± 6.5	SS	15.2 ± 1.4	-18.1 ± 1.5	SS	14.3 ± 1.8	-17.0 ± 2.2	SS	13.8 ± 1.9	-16.5 ± 2.1	SS
E20<br>Aml5	203.3 ± 27.4	135.3 ± 18.3	SS	213.0 ± 38.5	149.0 ± 26.9	SS	220.5 ± 43.0	159.8 ± 31.2	SS	214.1 ± 18.7	180.9 ± 15.9	SS	219.6 ± 28.4	188.3 ± 24.4	SS	225.7 ± 29.9	195.5 ± 25.9	SS
E20<br>B5	62.0 ± 8.4	-6.0 ± 0.9	0.00045	65.0 ± 11.7	0.9 ± 0.5	0.99376	67.3 ± 13.2	6.6 ± 1.4	0.01008	65.4 ± 5.8	32.2 ± 3.0	SS	67.2 ± 8.8	35.9 ± 4.8	SS	69.2 ± 9.3	38.9 ± 5.4	SS
E20<br>H12.5	292.7 ± 40.9	224.7 ± 31.9	SS	306.1 ± 54.0	242.1 ± 42.6	SS	317.1 ± 61.9	256.3 ± 50.1	SS	306.9 ± 28.1	273.7 ± 25.4	SS	314.1 ± 40.5	282.8 ± 36.6	SS	322.8 ± 43.8	292.6 ± 39.9	SS
L100<br>Aml5	356.2 ± 48.1	288.1 ± 39.0	SS	335.3 ± 60.6	271.2 ± 49.0	SS	318.2 ± 62.2	257.5 ± 50.3	SS	174.5 ± 15.2	141.3 ± 12.4	SS	164.7 ± 21.3	133.4 ± 17.3	SS	159.5 ± 21.0	129.2 ± 17.1	SS
L100<br>B5	108.8 ± 14.6	40.8 ± 5.5	SS	102.3 ± 18.4	38.3 ± 6.9	SS	97.2 ± 19.0	36.4 ± 7.2	SS	53.4 ± 4.7	20.2 ± 1.9	SS	50.5 ± 6.6	19.2 ± 2.6	SS	48.9 ± 6.5	18.7 ± 2.6	SS
L100<br>H12.5	512.4 ± 71.8	444.3 ± 62.8	SS	481.6 ± 85.2	417.6 ± 73.7	SS	457.4 ± 89.4	396.6 ± 77.6	SS	250.3 ± 22.9	217.1 ± 20.2	SS	235.6 ± 30.3	204.3 ± 26.4	SS	228.1 ± 30.8	197.9 ± 27.0	SS
Al300<br>Aml5/B5	6.8 ± 0.9	-61.3 ± 8.2	SS	6.4 ± 1.1	-57.7 ± 10.4	SS	6.0 ± 1.2	-54.7 ± 10.7	SS	3.3 ± 0.3	-29.9 ± 2.6	SS	3.1 ± 0.4	-28.2 ± 3.6	SS	3.0 ± 0.4	-27.2 ± 3.5	SS
Al300<br>Aml5/H12.5	31.8 ± 4.4	-36.2 ± 4.8	SS	29.9 ± 5.3	-34.1 ± 6.3	SS	28.4 ± 5.5	-32.4 ± 6.4	SS	15.5 ± 1.4	-17.7 ± 1.5	SS	14.6 ± 1.9	-16.7 ± 2.2	SS	14.1 ± 1.9	-16.1 ± 2.1	SS
Al300<br>B5/H12.5	9.7 ± 1.4	-58.4 ± 7.8	SS	9.1 ± 1.6	-54.9 ± 9.9	SS	8.7 ± 1.7	-52.1 ± 10.2	SS	4.7 ± 0.4	-28.5 ± 2.4	SS	4.5 ± 0.6	-26.8 ± 3.4	SS	4.3 ± 0.6	-25.9 ± 3.3	SS
E20<br>Aml5/B5	63.7 ± 8.6	-4.3 ± 0.7	0.00232	66.8 ± 12.0	2.7 ± 0.7	0.46756	69.1 ± 13.5	8.4 ± 1.8	0.00136	67.1 ± 5.9	33.9 ± 3.1	SS	68.9 ± 9.0	37.6 ± 5.0	SS	70.8 ± 9.5	40.6 ± 5.6	SS
E20<br>Aml5/H12.5	300.2 ± 41.9	232.1 ± 32.9	SS	313.9 ± 55.3	249.8 ± 43.8	SS	325.0 ± 63.2	264.2 ± 51.5	SS	314.0 ± 28.5	280.8 ± 25.8	SS	321.2 ± 41.3	289.9 ± 37.4	SS	330.1 ± 44.7	299.8 ± 40.9	SS
E20<br>B5/H12.5	91.5 ± 12.8	23.4 ± 4.1	SS	95.7 ± 17.0	31.7 ± 5.6	SS	99.2 ± 19.4	38.4 ± 7.8	SS	96.0 ± 8.8	62.8 ± 6.1	SS	98.4 ± 12.8	67.1 ± 8.8	SS	101.2 ± 13.9	71.0 ± 10.0	SS
L100<br>Aml5/B5	111.8 ± 14.9	43.7 ± 5.9	SS	105.1 ± 18.9	41.1 ± 7.3	SS	99.8 ± 19.5	39.0 ± 7.6	SS	54.8 ± 4.8	21.5 ± 2.0	SS	51.7 ± 6.7	20.4 ± 2.7	SS	50.1 ± 6.7	19.9 ± 2.8	SS
L100<br>Aml5/H12.5	525.4 ± 73.3	457.4 ± 64.3	SS	493.8 ± 87.2	429.8 ± 75.7	SS	468.8 ± 91.3	408.0 ± 79.6	SS	256.2 ± 23.3	223.0 ± 20.5	SS	241.1 ± 31.0	209.8 ± 27.0	SS	233.4 ± 31.6	203.1 ± 27.7	SS
L100<br>B5/H12.5	160.0 ± 22.4	92.0 ± 13.4	SS	150.5 ± 26.7	86.4 ± 15.2	SS	143.0 ± 28.0	82.2 ± 16.3	SS	78.4 ± 7.2	45.2 ± 4.5	SS	73.9 ± 9.6	42.6 ± 5.7	SS	71.7 ± 9.8	41.4 ± 6.0	SS

Al300 = aliskiren 300 mg; **Aml5** = amlodipine 5 mg; **B5** = bisoprolol 5 mg; **E20** = enalapril 20 mg; **H12.5** = hydrochlorothiazide 12.5 mg; **L100** = losartan 100 mg; **SS** = statistically significant (P < 0.00001)

Table S51. *P*-values calculated using the Kolmogorov-Smirnov test for changes in plasma renin activity in subpopulations (n = 100) with different ACE activity receiving the same regimens. Case 1: c_{ACE} = 7.0 h^{-1} (*LII*), case 2: c_{ACE} = 8.9 h^{-1} (*LID*), case 3: c_{ACE} = 10.8 h^{-1} (*LDD*), case 4: c_{ACE} = 42.3 h^{-1} (*HII*), case 5: c_{ACE} = 54.1 h^{-1} (*HID*), case 6: c_{ACE} = 65.9 h^{-1} (*HDD*). *P*-value for case *i vs*. case *j* is denoted P_{ij}.

Regimens	P_{12}	P_{13}	P_{23}	P_{14}	P_{15}	P_{16}	P_{24}	P_{25}	P_{26}	P_{34}	P_{35}	P_{36}	P_{45}	P_{46}	P_{56}
Al300	0.02431	0.00004	0.36672	SS	SS	SS	SS	SS	SS	SS	SS	SS	0.00025	SS	0.07832
E20	0.00004	SS	0.05410	SS	SS	SS	SS	SS	SS	SS	SS	SS	0.00002	SS	0.02431
L100	0.02431	0.00007	0.36672	SS	SS	SS	SS	SS	SS	SS	SS	SS	0.00025	SS	0.11113
Aml5	0.03663	SS	0.02431	SS	SS	SS	SS	SS	SS	SS	SS	SS	0.00007	SS	0.11113
B5	0.02431	0.00007	0.36672	SS	SS	SS	SS	SS	SS	SS	SS	SS	0.00025	SS	0.07832
H12.5	0.15454	0.00025	0.11113	SS	SS	SS	SS	SS	SS	SS	SS	SS	0.00045	SS	0.21055
Al300<br>Aml5	0.02431	0.00007	0.36672	SS	SS	SS	SS	SS	SS	SS	SS	SS	0.00025	SS	0.07832
Al300<br>B5	0.02431	0.00007	0.36672	SS	SS	SS	SS	SS	SS	SS	SS	SS	0.00025	SS	0.07832
Al300<br>H12.5	0.01008	0.00007	0.28093	SS	SS	SS	SS	SS	SS	SS	SS	SS	0.00025	SS	0.03663
E20<br>Aml5	0.00004	SS	0.05410	SS	SS	SS	SS	SS	SS	SS	SS	SS	0.00002	SS	0.02431
E20<br>B5	SS	SS	SS	SS	SS	SS	SS	SS	SS	SS	SS	SS	SS	SS	0.00045
E20<br>H12.5	0.00045	0.00002	0.07832	SS	SS	SS	SS	SS	SS	0.00025	0.00013	0.00004	0.00045	0.00004	0.11113
L100<br>Aml5	0.03663	0.00007	0.36672	SS	SS	SS	SS	SS	SS	SS	SS	SS	0.00025	SS	0.15454
L100<br>B5	0.01581	0.00002	0.28093	SS	SS	SS	SS	SS	SS	SS	SS	SS	0.00079	0.00045	0.28093
L100<br>H12.5	0.03663	0.00004	0.28093	SS	SS	SS	SS	SS	SS	SS	SS	SS	0.00045	SS	0.11113
Al300<br>Aml5/B5	0.02431	0.00007	0.36672	SS	SS	SS	SS	SS	SS	SS	SS	SS	0.00025	SS	0.07832
Al300<br>Aml5/H12.5	0.01008	0.00007	0.28093	SS	SS	SS	SS	SS	SS	SS	SS	SS	0.00025	0.00002	0.03663
Al300<br>B5/H12.5	0.02431	0.00004	0.36672	SS	SS	SS	SS	SS	SS	SS	SS	SS	0.00025	SS	0.05410
E20<br>Aml5/B5	SS	SS	SS	SS	SS	SS	SS	SS	SS	SS	SS	SS	SS	SS	0.00045
E20<br>Aml5/H12.5	0.00045	0.00007	0.07832	SS	SS	SS	SS	SS	SS	0.00025	0.00045	0.00013	0.00045	0.00004	0.11113
E20<br>B5/H12.5	SS	SS	SS	SS	SS	SS	SS	SS	SS	SS	SS	SS	0.00002	SS	0.01581
L100<br>Aml5/B5	0.01008	SS	0.28093	SS	SS	SS	SS	SS	SS	SS	SS	SS	0.00136	0.00013	0.36672
L100<br>Aml5/H12.5	0.02431	SS	0.21055	SS	SS	SS	SS	SS	SS	SS	SS	SS	0.00013	SS	0.11113
L100<br>B5/H12.5	0.05410	0.00004	0.21055	SS	SS	SS	SS	SS	SS	SS	SS	SS	0.00136	0.00004	0.36672

Al300 = aliskiren 300 mg; **Aml5** = amlodipine 5 mg; **B5** = bisoprolol 5 mg; **E20** = enalapril 20 mg; **H12.5** = hydrochlorothiazide 12.5 mg; **L100** = losartan 100 mg; **SS** = statistically significant (P < 0.00001)

Figure S33. Simulated change in plasma angiotensin I from baseline to week 4 (mean ± SD, n = 100)

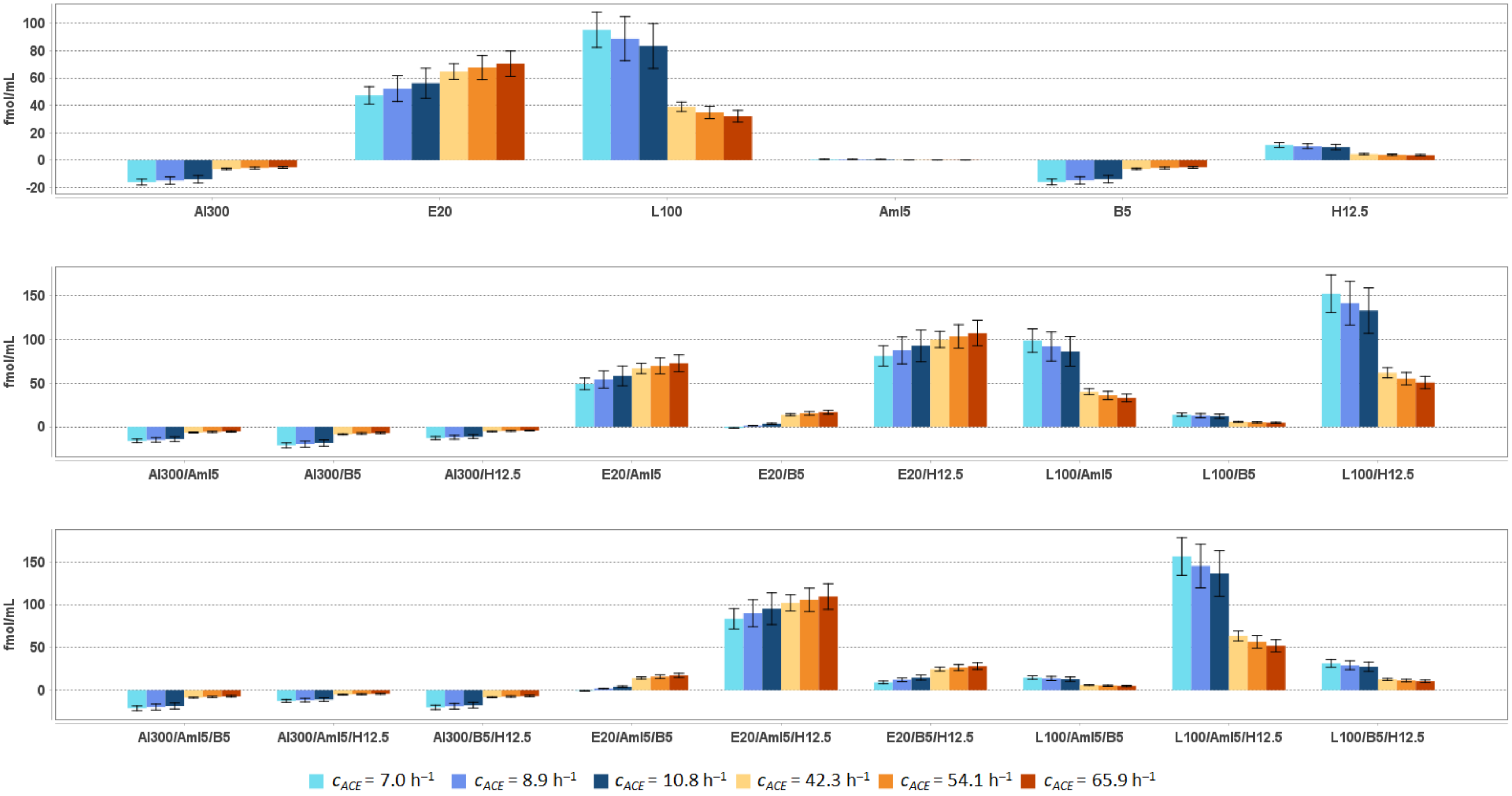

Al300 = aliskiren 300 mg; **Aml5** = amlodipine 5 mg; **B5** = bisoprolol 5 mg; **E20** = enalapril 20 mg; **H12.5** = hydrochlorothiazide 12.5 mg; **L100** = losartan 100 mg

Table S52. Simulated response of plasma angiotensin I to antihypertensive therapy in virtual hypertensive subpopulations (n = 100) with different ACE activity, including P-values (Kolmogorov-Smirnov test) for endpoint *vs*. baseline; data are presented as mean ± SD in fmol/mL

Regimens	*LII* (c_{ACE} = 7.0 h^{-1})			*LID* (c_{ACE} = 8.9 h^{-1})			*LDD* (c_{ACE} = 10.8 h^{-1})			*HII* (c_{ACE} = 42.3 h^{-1})			*HID* (c_{ACE} = 54.1 h^{-1})			*HDD* (c_{ACE} = 65.9 h^{-1})		
	**Value**	**Change**	***P***	**Value**	**Change**	***P***	**Value**	**Change**	***P***	**Value**	**Change**	***P***	**Value**	**Change**	***P***	**Value**	**Change**	***P***
Baseline	23.3 ± 3.1	–	–	21.7 ± 3.9	–	–	20.3 ± 4.0	–	–	9.4 ± 0.8	–	–	8.4 ± 1.1	–	–	7.7 ± 1.0	–	–
Al300	7.2 ± 1.0	-16.1 ± 2.1	SS	6.7 ± 1.2	-15.0 ± 2.7	SS	6.3 ± 1.2	-14.0 ± 2.7	SS	2.9 ± 0.3	-6.5 ± 0.6	SS	2.6 ± 0.3	-5.8 ± 0.7	SS	2.4 ± 0.3	-5.3 ± 0.7	SS
E20	70.6 ± 9.5	47.3 ± 6.4	SS	73.9 ± 13.4	52.3 ± 9.5	SS	76.5 ± 15.0	56.2 ± 11.0	SS	74.2 ± 6.5	64.7 ± 5.7	SS	76.1 ± 9.9	67.7 ± 8.8	SS	78.2 ± 10.3	70.5 ± 9.3	SS
L100	118.6 ± 16.0	95.3 ± 12.9	SS	110.4 ± 20.0	88.8 ± 16.1	SS	103.7 ± 20.3	83.4 ± 16.3	SS	48.4 ± 4.2	39.0 ± 3.4	SS	43.3 ± 5.6	34.9 ± 4.5	SS	39.8 ± 5.2	32.1 ± 4.2	SS
Aml5	23.8 ± 3.2	0.5 ± 0.1	0.69937	22.1 ± 4.0	0.5 ± 0.1	0.90621	20.8 ± 4.0	0.4 ± 0.1	0.58062	9.7 ± 0.8	0.2 ± 0.0	0.21055	8.6 ± 1.1	0.2 ± 0.0	0.11113	7.9 ± 1.0	0.2 ± 0.0	0.58062
B5	7.3 ± 1.0	-16.0 ± 2.1	SS	6.8 ± 1.2	-14.9 ± 2.7	SS	6.4 ± 1.2	-14.0 ± 2.7	SS	3.0 ± 0.3	-6.5 ± 0.6	SS	2.7 ± 0.3	-5.8 ± 0.7	SS	2.4 ± 0.3	-5.3 ± 0.7	SS
H12.5	34.3 ± 4.8	11.0 ± 1.7	SS	31.8 ± 5.6	10.2 ± 1.8	SS	29.9 ± 5.8	9.6 ± 1.9	SS	13.9 ± 1.2	4.4 ± 0.5	SS	12.3 ± 1.6	3.9 ± 0.5	SS	11.3 ± 1.5	3.6 ± 0.5	SS
Al300<br>Aml5	7.4 ± 1.0	-15.9 ± 2.1	SS	6.9 ± 1.2	-14.8 ± 2.7	SS	6.4 ± 1.3	-13.9 ± 2.7	SS	3.0 ± 0.3	-6.4 ± 0.5	SS	2.7 ± 0.3	-5.7 ± 0.7	SS	2.5 ± 0.3	-5.3 ± 0.7	SS
Al300<br>B5	2.3 ± 0.3	-21.0 ± 2.8	SS	2.1 ± 0.4	-19.6 ± 3.5	SS	2.0 ± 0.4	-18.4 ± 3.6	SS	0.9 ± 0.1	-8.5 ± 0.7	SS	0.8 ± 0.1	-7.6 ± 1.0	SS	0.8 ± 0.1	-7.0 ± 0.9	SS
Al300<br>H12.5	10.6 ± 1.5	-12.7 ± 1.7	SS	9.9 ± 1.7	-11.8 ± 2.2	SS	9.3 ± 1.8	-11.1 ± 2.2	SS	4.3 ± 0.4	-5.1 ± 0.4	SS	3.8 ± 0.5	-4.6 ± 0.6	SS	3.5 ± 0.5	-4.2 ± 0.5	SS
E20<br>Aml5	72.4 ± 9.8	49.1 ± 6.6	SS	75.8 ± 13.7	54.2 ± 9.8	SS	78.5 ± 15.3	58.1 ± 11.4	SS	76.1 ± 6.7	66.7 ± 5.8	SS	78.1 ± 10.1	69.6 ± 9.0	SS	80.2 ± 10.6	72.5 ± 9.6	SS
E20<br>B5	22.1 ± 3.0	-1.2 ± 0.2	0.05410	23.1 ± 4.2	1.5 ± 0.3	0.15454	24.0 ± 4.7	3.6 ± 0.7	0.00007	23.3 ± 2.1	13.8 ± 1.3	SS	23.9 ± 3.1	15.5 ± 2.0	SS	24.6 ± 3.3	16.8 ± 2.3	SS
E20<br>H12.5	104.2 ± 14.6	80.9 ± 11.5	SS	108.9 ± 19.2	87.3 ± 15.4	SS	112.8 ± 22.0	92.5 ± 18.1	SS	109.1 ± 10.0	99.7 ± 9.2	SS	111.7 ± 14.4	103.2 ± 13.3	SS	114.7 ± 15.6	107.0 ± 14.6	SS
L100<br>Aml5	121.7 ± 16.5	98.5 ± 13.3	SS	113.4 ± 20.5	91.7 ± 16.6	SS	106.5 ± 20.8	86.1 ± 16.8	SS	49.7 ± 4.3	40.2 ± 3.5	SS	44.4 ± 5.7	36.0 ± 4.7	SS	40.8 ± 5.4	33.1 ± 4.4	SS
L100<br>B5	37.2 ± 5.0	13.9 ± 1.9	SS	34.6 ± 6.2	12.9 ± 2.3	SS	32.5 ± 6.3	12.2 ± 2.4	SS	15.2 ± 1.3	5.7 ± 0.5	SS	13.6 ± 1.8	5.2 ± 0.7	SS	12.5 ± 1.7	4.8 ± 0.7	SS
L100<br>H12.5	175.1 ± 24.5	151.9 ± 21.5	SS	162.8 ± 28.8	141.2 ± 24.9	SS	153.0 ± 29.9	132.7 ± 26.0	SS	71.2 ± 6.5	61.8 ± 5.7	SS	63.5 ± 8.2	55.1 ± 7.1	SS	58.4 ± 7.9	50.6 ± 6.9	SS
Al300<br>Aml5/B5	2.3 ± 0.3	-21.0 ± 2.8	SS	2.2 ± 0.4	-19.5 ± 3.5	SS	2.0 ± 0.4	-18.3 ± 3.6	SS	0.9 ± 0.1	-8.5 ± 0.7	SS	0.8 ± 0.1	-7.6 ± 1.0	SS	0.8 ± 0.1	-7.0 ± 0.9	SS
Al300<br>Aml5/H12.5	10.9 ± 1.5	-12.4 ± 1.6	SS	10.1 ± 1.8	-11.5 ± 2.1	SS	9.5 ± 1.8	-10.8 ± 2.1	SS	4.4 ± 0.4	-5.0 ± 0.4	SS	3.9 ± 0.5	-4.5 ± 0.6	SS	3.6 ± 0.5	-4.1 ± 0.5	SS
Al300<br>B5/H12.5	3.3 ± 0.5	-20.0 ± 2.7	SS	3.1 ± 0.5	-18.6 ± 3.4	SS	2.9 ± 0.6	-17.4 ± 3.4	SS	1.4 ± 0.1	-8.1 ± 0.7	SS	1.2 ± 0.2	-7.2 ± 0.9	SS	1.1 ± 0.2	-6.6 ± 0.9	SS
E20<br>Aml5/B5	22.7 ± 3.1	-0.6 ± 0.2	0.46756	23.8 ± 4.3	2.1 ± 0.4	0.01581	24.6 ± 4.8	4.3 ± 0.9	SS	23.9 ± 2.1	14.4 ± 1.3	SS	24.5 ± 3.2	16.1 ± 2.1	SS	25.2 ± 3.4	17.4 ± 2.4	SS
E20<br>Aml5/H12.5	106.8 ± 14.9	83.6 ± 11.8	SS	111.7 ± 19.7	90.1 ± 15.8	SS	115.6 ± 22.5	95.3 ± 18.6	SS	111.7 ± 10.1	102.2 ± 9.4	SS	114.2 ± 14.7	105.8 ± 13.6	SS	117.3 ± 15.9	109.6 ± 14.9	SS
E20<br>B5/H12.5	32.6 ± 4.6	9.3 ± 1.6	SS	34.1 ± 6.0	12.4 ± 2.2	SS	35.3 ± 6.9	15.0 ± 3.0	SS	34.1 ± 3.1	24.7 ± 2.4	SS	35.0 ± 4.5	26.6 ± 3.5	SS	36.0 ± 4.9	28.2 ± 3.9	SS
L100<br>Aml5/B5	38.2 ± 5.1	14.9 ± 2.0	SS	35.5 ± 6.4	13.9 ± 2.5	SS	33.4 ± 6.5	13.1 ± 2.6	SS	15.6 ± 1.4	6.1 ± 0.6	SS	13.9 ± 1.8	5.5 ± 0.7	SS	12.8 ± 1.7	5.1 ± 0.7	SS
L100<br>Aml5/H12.5	179.6 ± 25.1	156.3 ± 22.0	SS	167.0 ± 29.5	145.3 ± 25.6	SS	156.8 ± 30.5	136.5 ± 26.6	SS	72.9 ± 6.6	63.4 ± 5.8	SS	65.0 ± 8.3	56.5 ± 7.3	SS	59.7 ± 8.1	52.0 ± 7.1	SS
L100<br>B5/H12.5	54.7 ± 7.6	31.4 ± 4.6	SS	50.9 ± 9.0	29.2 ± 5.2	SS	47.8 ± 9.4	27.5 ± 5.5	SS	22.3 ± 2.0	12.9 ± 1.3	SS	19.9 ± 2.6	11.5 ± 1.5	SS	18.3 ± 2.5	10.6 ± 1.5	SS

Al300 = aliskiren 300 mg; **Aml5** = amlodipine 5 mg; **B5** = bisoprolol 5 mg; **E20** = enalapril 20 mg; **H12.5** = hydrochlorothiazide 12.5 mg; **L100** = losartan 100 mg; **SS** = statistically significant (p < 0.00001)

Table S53. *P*-values calculated using the Kolmogorov-Smirnov test for changes in <u>plasma angiotensin I</u> in subpopulations (n = 100) with different ACE activity receiving the same regimens. Case 1: c_{ACE} = 7.0 h^{-1} (*LII*), case 2: c_{ACE} = 8.9 h^{-1} (*LID*), case 3: c_{ACE} = 10.8 h^{-1} (*LDD*), case 4: c_{ACE} = 42.3 h^{-1} (*HII*), case 5: c_{ACE} = 54.1 h^{-1} (*HID*), case 6: c_{ACE} = 65.9 h^{-1} (*HDD*). *P*-value for case *i vs*. case *j* is denoted P_{ij}.

**Regimens**	P_{12}	P_{13}	P_{23}	P_{14}	P_{15}	P_{16}	P_{24}	P_{25}	P_{26}	P_{34}	P_{35}	P_{36}	P_{45}	P_{46}	P_{56}
Al300	0.01008	SS	0.21055	SS	SS	SS	SS	SS	SS	SS	SS	SS	SS	SS	0.00004
E20	0.00002	SS	0.05410	SS	SS	SS	SS	SS	SS	SS	SS	SS	SS	SS	0.02431
L100	0.01581	SS	0.28093	SS	SS	SS	SS	SS	SS	SS	SS	SS	SS	SS	0.00007
Aml5	0.01581	SS	0.01008	SS	SS	SS	SS	SS	SS	SS	SS	SS	SS	SS	0.00136
B5	0.01008	SS	0.21055	SS	SS	SS	SS	SS	SS	SS	SS	SS	SS	SS	0.00004
H12.5	0.05410	0.00004	0.05410	SS	SS	SS	SS	SS	SS	SS	SS	SS	SS	SS	0.00232
Al300<br>Aml5	0.01008	SS	0.21055	SS	SS	SS	SS	SS	SS	SS	SS	SS	SS	SS	0.00004
Al300<br>B5	0.01008	SS	0.28093	SS	SS	SS	SS	SS	SS	SS	SS	SS	SS	SS	0.00004
Al300<br>H12.5	0.00232	SS	0.28093	SS	SS	SS	SS	SS	SS	SS	SS	SS	SS	SS	0.00007
E20<br>Aml5	0.00004	SS	0.05410	SS	SS	SS	SS	SS	SS	SS	SS	SS	0.00002	SS	0.02431
E20<br>B5	SS	SS	SS	SS	SS	SS	SS	SS	SS	SS	SS	SS	SS	SS	0.00025
E20<br>H12.5	0.00045	SS	0.07832	SS	SS	SS	SS	SS	SS	0.00007	0.00007	SS	0.00013	0.00002	0.07832
L100<br>Aml5	0.01581	SS	0.21055	SS	SS	SS	SS	SS	SS	SS	SS	SS	SS	SS	0.00007
L100<br>B5	0.00630	SS	0.21055	SS	SS	SS	SS	SS	SS	SS	SS	SS	SS	SS	0.00136
L100<br>H12.5	0.01008	SS	0.15454	SS	SS	SS	SS	SS	SS	SS	SS	SS	SS	SS	0.00136
Al300<br>Aml5/B5	0.01008	SS	0.28093	SS	SS	SS	SS	SS	SS	SS	SS	SS	SS	SS	0.00004
Al300<br>Aml5/H12.5	0.00232	SS	0.28093	SS	SS	SS	SS	SS	SS	SS	SS	SS	SS	SS	0.00007
Al300<br>B5/H12.5	0.01008	SS	0.21055	SS	SS	SS	SS	SS	SS	SS	SS	SS	SS	SS	0.00004
E20<br>Aml5/B5	SS	SS	SS	SS	SS	SS	SS	SS	SS	SS	SS	SS	SS	SS	0.00025
E20<br>Aml5/H12.5	0.00045	SS	0.07832	SS	SS	SS	SS	SS	SS	0.00013	0.00013	SS	0.00025	0.00004	0.07832
E20<br>B5/H12.5	SS	SS	SS	SS	SS	SS	SS	SS	SS	SS	SS	SS	SS	SS	0.01008
L100<br>Aml5/B5	0.00630	SS	0.11113	SS	SS	SS	SS	SS	SS	SS	SS	SS	SS	SS	0.00079
L100<br>Aml5/H12.5	0.01008	SS	0.11113	SS	SS	SS	SS	SS	SS	SS	SS	SS	SS	SS	0.00136
L100<br>B5/H12.5	0.01581	SS	0.07832	SS	SS	SS	SS	SS	SS	SS	SS	SS	SS	SS	0.01008

Al300 = aliskiren 300 mg; **Aml5** = amlodipine 5 mg; **B5** = bisoprolol 5 mg; **E20** = enalapril 20 mg; **H12.5** = hydrochlorothiazide 12.5 mg; **L100** = losartan 100 mg; **SS** = statistically significant (P < 0.00001)

Figure S34. Simulated change in plasma angiotensin II from baseline to week 4 (mean ± SD, n = 100)

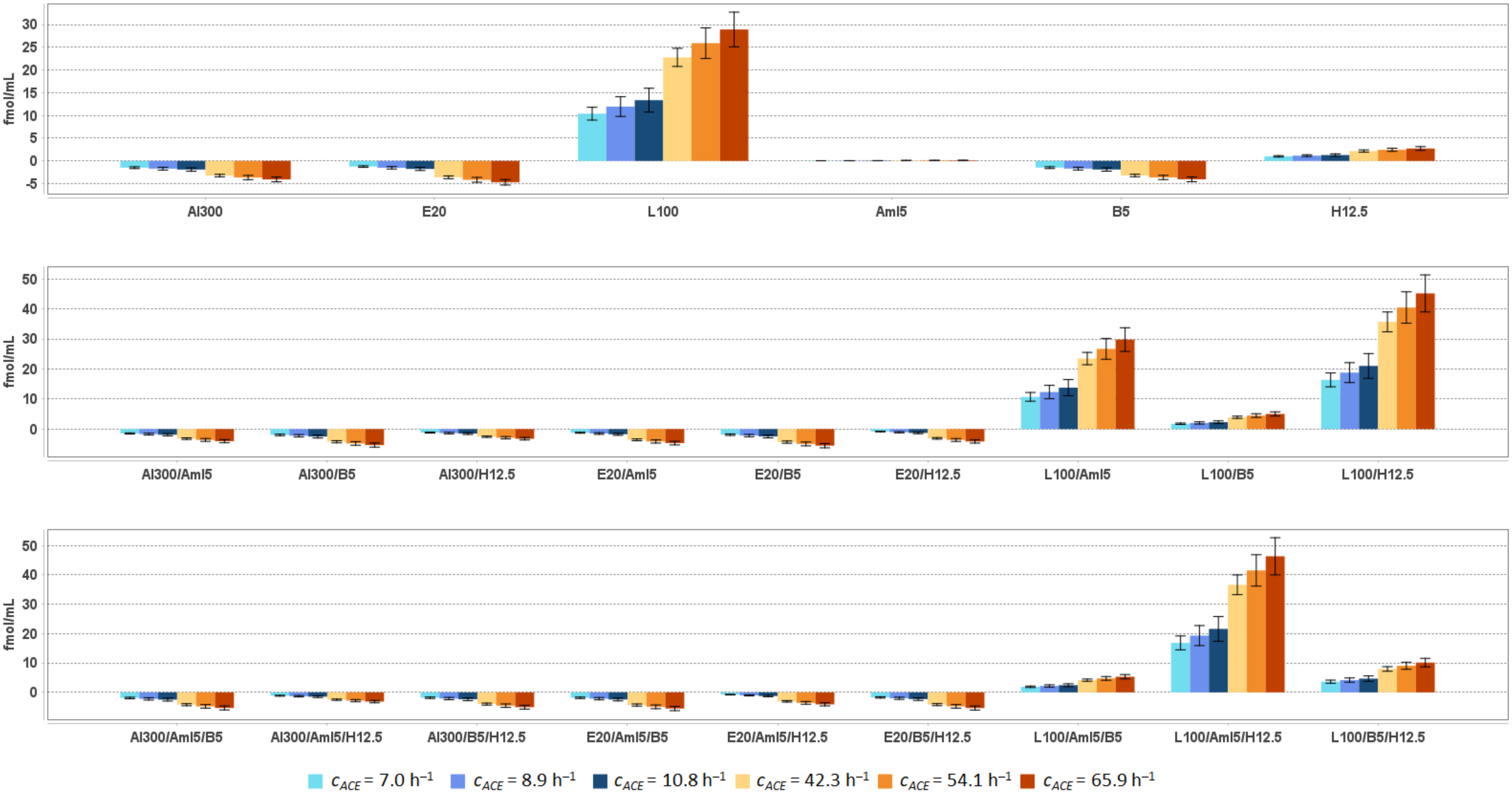

Al300 = aliskiren 300 mg; **Aml5** = amlodipine 5 mg; **B5** = bisoprolol 5 mg; **E20** = enalapril 20 mg; **H12.5** = hydrochlorothiazide 12.5 mg; **L100** = losartan 100 mg

Table S54. Simulated response of plasma angiotensin II to antihypertensive therapy in virtual hypertensive subpopulations (n = 100) with different ACE activity, including P-values (Kolmogorov-Smirnov test) for endpoint *vs*. baseline; data are presented as mean ± SD in fmol/mL

Regimens	***LII*** **(c_{ACE} = 7.0 h^{-1})**			***LID*** **(c_{ACE} = 8.9 h^{-1})**			***LDD*** **(c_{ACE} = 10.8 h^{-1})**			***HII*** **(c_{ACE} = 42.3 h^{-1})**			***HID*** **(c_{ACE} = 54.1 h^{-1})**			***HDD*** **(c_{ACE} = 65.9 h^{-1})**		
	**Value**	**Change**	***P***	**Value**	**Change**	***P***	**Value**	**Change**	***P***	**Value**	**Change**	***P***	**Value**	**Change**	***P***	**Value**	**Change**	***P***
Baseline	2.1 ± 0.3	–	–	2.5 ± 0.4	–	–	2.8 ± 0.5	–	–	4.7 ± 0.4	–	–	5.3 ± 0.7	–	–	5.9 ± 0.8	–	–
Al300	0.7 ± 0.1	-1.5 ± 0.2	SS	0.8 ± 0.1	-1.7 ± 0.3	SS	0.9 ± 0.2	-1.9 ± 0.4	SS	1.4 ± 0.1	-3.2 ± 0.3	SS	1.6 ± 0.2	-3.7 ± 0.5	SS	1.8 ± 0.2	-4.1 ± 0.5	SS
E20	0.9 ± 0.1	-1.2 ± 0.2	SS	1.0 ± 0.2	-1.5 ± 0.3	SS	1.0 ± 0.2	-1.8 ± 0.3	SS	1.1 ± 0.1	-3.6 ± 0.3	SS	1.1 ± 0.1	-4.2 ± 0.5	SS	1.2 ± 0.2	-4.7 ± 0.6	SS
L100	12.5 ± 1.7	10.4 ± 1.4	SS	14.4 ± 2.6	12.0 ± 2.2	SS	16.1 ± 3.2	13.4 ± 2.6	SS	27.5 ± 2.4	22.8 ± 2.0	SS	31.2 ± 4.0	25.9 ± 3.4	SS	34.8 ± 4.6	28.9 ± 3.8	SS
Aml5	2.2 ± 0.3	0.0 ± 0.0	0.69937	2.5 ± 0.5	0.1 ± 0.0	0.90621	2.8 ± 0.5	0.1 ± 0.0	0.58062	4.8 ± 0.4	0.1 ± 0.0	0.21055	5.4 ± 0.7	0.1 ± 0.0	0.11113	6.0 ± 0.8	0.1 ± 0.0	0.58062
B5	0.7 ± 0.1	-1.5 ± 0.2	SS	0.8 ± 0.1	-1.7 ± 0.3	SS	0.9 ± 0.2	-1.9 ± 0.4	SS	1.5 ± 0.1	-3.2 ± 0.3	SS	1.7 ± 0.2	-3.6 ± 0.5	SS	1.9 ± 0.2	-4.0 ± 0.5	SS
H12.5	3.2 ± 0.4	1.0 ± 0.2	SS	3.6 ± 0.6	1.2 ± 0.2	SS	4.1 ± 0.8	1.3 ± 0.3	SS	6.8 ± 0.6	2.2 ± 0.2	SS	7.8 ± 1.0	2.5 ± 0.3	SS	8.6 ± 1.2	2.7 ± 0.4	SS
Al300 Aml5	0.7 ± 0.1	-1.5 ± 0.2	SS	0.8 ± 0.1	-1.7 ± 0.3	SS	0.9 ± 0.2	-1.9 ± 0.4	SS	1.5 ± 0.1	-3.2 ± 0.3	SS	1.7 ± 0.2	-3.6 ± 0.5	SS	1.9 ± 0.2	-4.0 ± 0.5	SS
Al300 B5	0.2 ± 0.0	-1.9 ± 0.3	SS	0.2 ± 0.0	-2.2 ± 0.4	SS	0.3 ± 0.1	-2.5 ± 0.5	SS	0.5 ± 0.0	-4.2 ± 0.4	SS	0.5 ± 0.1	-4.8 ± 0.6	SS	0.6 ± 0.1	-5.3 ± 0.7	SS
Al300 H12.5	1.0 ± 0.1	-1.2 ± 0.2	SS	1.1 ± 0.2	-1.3 ± 0.2	SS	1.3 ± 0.2	-1.5 ± 0.3	SS	2.1 ± 0.2	-2.5 ± 0.2	SS	2.4 ± 0.3	-2.9 ± 0.4	SS	2.7 ± 0.4	-3.2 ± 0.4	SS
E20 Aml5	0.9 ± 0.1	-1.2 ± 0.2	SS	1.0 ± 0.2	-1.5 ± 0.3	SS	1.0 ± 0.2	-1.7 ± 0.3	SS	1.1 ± 0.1	-3.6 ± 0.3	SS	1.2 ± 0.2	-4.1 ± 0.5	SS	1.2 ± 0.2	-4.7 ± 0.6	SS
E20 B5	0.3 ± 0.0	-1.9 ± 0.2	SS	0.3 ± 0.1	-2.2 ± 0.4	SS	0.3 ± 0.1	-2.4 ± 0.5	SS	0.3 ± 0.0	-4.3 ± 0.4	SS	0.4 ± 0.0	-4.9 ± 0.6	SS	0.4 ± 0.1	-5.5 ± 0.7	SS
E20 H12.5	1.3 ± 0.2	-0.8 ± 0.1	SS	1.4 ± 0.2	-1.1 ± 0.2	SS	1.5 ± 0.3	-1.3 ± 0.3	SS	1.6 ± 0.1	-3.1 ± 0.3	SS	1.7 ± 0.2	-3.6 ± 0.5	SS	1.8 ± 0.2	-4.1 ± 0.5	SS
L100 Aml5	12.9 ± 1.7	10.7 ± 1.5	SS	14.8 ± 2.7	12.3 ± 2.2	SS	16.5 ± 3.2	13.8 ± 2.7	SS	28.2 ± 2.5	23.5 ± 2.1	SS	32.0 ± 4.1	26.7 ± 3.5	SS	35.7 ± 4.7	29.8 ± 3.9	SS
L100 B5	3.9 ± 0.5	1.8 ± 0.2	SS	4.5 ± 0.8	2.1 ± 0.4	SS	5.1 ± 1.0	2.3 ± 0.5	SS	8.6 ± 0.8	3.9 ± 0.4	SS	9.8 ± 1.3	4.5 ± 0.6	SS	11.0 ± 1.5	5.0 ± 0.7	SS
L100 H12.5	18.5 ± 2.6	16.4 ± 2.3	SS	21.3 ± 3.8	18.8 ± 3.3	SS	23.8 ± 4.6	21.0 ± 4.1	SS	40.4 ± 3.7	35.7 ± 3.3	SS	45.8 ± 5.9	40.5 ± 5.2	SS	51.1 ± 6.9	45.2 ± 6.2	SS
Al300 Aml5/B5	0.2 ± 0.0	-1.9 ± 0.3	SS	0.2 ± 0.0	-2.2 ± 0.4	SS	0.3 ± 0.1	-2.5 ± 0.5	SS	0.5 ± 0.0	-4.2 ± 0.4	SS	0.5 ± 0.1	-4.8 ± 0.6	SS	0.6 ± 0.1	-5.3 ± 0.7	SS
Al300 Aml5/H12.5	1.0 ± 0.1	-1.1 ± 0.2	SS	1.2 ± 0.2	-1.3 ± 0.2	SS	1.3 ± 0.3	-1.5 ± 0.3	SS	2.2 ± 0.2	-2.5 ± 0.2	SS	2.5 ± 0.3	-2.8 ± 0.4	SS	2.8 ± 0.4	-3.1 ± 0.4	SS
Al300 B5/H12.5	0.3 ± 0.0	-1.8 ± 0.2	SS	0.4 ± 0.1	-2.1 ± 0.4	SS	0.4 ± 0.1	-2.4 ± 0.5	SS	0.7 ± 0.1	-4.0 ± 0.3	SS	0.8 ± 0.1	-4.5 ± 0.6	SS	0.8 ± 0.1	-5.1 ± 0.7	SS
E20 Aml5/B5	0.3 ± 0.0	-1.9 ± 0.2	SS	0.3 ± 0.1	-2.2 ± 0.4	SS	0.3 ± 0.1	-2.4 ± 0.5	SS	0.3 ± 0.0	-4.3 ± 0.4	SS	0.4 ± 0.0	-4.9 ± 0.6	SS	0.4 ± 0.1	-5.5 ± 0.7	SS
E20 Aml5/H12.5	1.4 ± 0.2	-0.8 ± 0.1	SS	1.4 ± 0.3	-1.0 ± 0.2	SS	1.5 ± 0.3	-1.2 ± 0.2	SS	1.6 ± 0.1	-3.1 ± 0.3	SS	1.7 ± 0.2	-3.6 ± 0.5	SS	1.8 ± 0.2	-4.1 ± 0.5	SS
E20 B5/H12.5	0.4 ± 0.1	-1.7 ± 0.2	SS	0.4 ± 0.1	-2.0 ± 0.4	SS	0.5 ± 0.1	-2.3 ± 0.4	SS	0.5 ± 0.0	-4.2 ± 0.4	SS	0.5 ± 0.1	-4.8 ± 0.6	SS	0.6 ± 0.1	-5.3 ± 0.7	SS
L100 Aml5/B5	4.0 ± 0.5	1.9 ± 0.3	SS	4.6 ± 0.8	2.2 ± 0.4	SS	5.2 ± 1.0	2.4 ± 0.5	SS	8.8 ± 0.8	4.2 ± 0.4	SS	10.1 ± 1.3	4.8 ± 0.6	SS	11.2 ± 1.5	5.3 ± 0.7	SS
L100 Aml5/H12.5	19.0 ± 2.7	16.9 ± 2.4	SS	21.8 ± 3.8	19.3 ± 3.4	SS	24.4 ± 4.7	21.6 ± 4.2	SS	41.3 ± 3.8	36.7 ± 3.4	SS	46.8 ± 6.0	41.5 ± 5.3	SS	52.3 ± 7.1	46.4 ± 6.3	SS
L100 B5/H12.5	5.8 ± 0.8	3.6 ± 0.5	SS	6.6 ± 1.2	4.2 ± 0.7	SS	7.4 ± 1.5	4.7 ± 0.9	SS	12.6 ± 1.2	8.0 ± 0.8	SS	14.4 ± 1.9	9.1 ± 1.2	SS	16.0 ± 2.2	10.1 ± 1.4	SS

Al300 = aliskiren 300 mg; **Aml5** = amlodipine 5 mg; **B5** = bisoprolol 5 mg; **E20** = enalapril 20 mg; **H12.5** = hydrochlorothiazide 12.5 mg; **L100** = losartan 100 mg; **SS** = statistically significant (P < 0.00001)

Table S55. *P*-values calculated using the Kolmogorov-Smirnov test for changes in plasma angiotensin II in subpopulations (n = 100) with different ACE activity receiving the same regimens. Case 1: c_{ACE} = 7.0 h^{-1} (*LII*), case 2: c_{ACE} = 8.9 h^{-1} (*LID*), case 3: c_{ACE} = 10.8 h^{-1} (*LDD*), case 4: c_{ACE} = 42.3 h^{-1} (*HII*), case 5: c_{ACE} = 54.1 h^{-1} (*HID*), case 6: c_{ACE} = 65.9 h^{-1} (*HDD*). *P*-value for case *i vs*. case *j* is denoted P_{ij}.

**Regimens**	P_{12}	P_{13}	P_{23}	P_{14}	P_{15}	P_{16}	P_{24}	P_{25}	P_{26}	P_{34}	P_{35}	P_{36}	P_{45}	P_{46}	P_{56}
Al300	SS	SS	0.00136	SS	SS	SS	SS	SS	SS	SS	SS	SS	SS	SS	0.00002
E20	SS	SS	0.00013	SS	SS	SS	SS	SS	SS	SS	SS	SS	SS	SS	SS
L100	SS	SS	0.00386	SS	SS	SS	SS	SS	SS	SS	SS	SS	SS	SS	0.00004
Aml5	0.00025	SS	0.00136	SS	SS	SS	SS	SS	SS	SS	SS	SS	0.00025	SS	0.00232
B5	SS	SS	0.00136	SS	SS	SS	SS	SS	SS	SS	SS	SS	SS	SS	0.00002
H12.5	SS	SS	0.00630	SS	SS	SS	SS	SS	SS	SS	SS	SS	SS	SS	0.00079
Al300<br>Aml5	SS	SS	0.00136	SS	SS	SS	SS	SS	SS	SS	SS	SS	SS	SS	0.00002
Al300<br>B5	SS	SS	0.00136	SS	SS	SS	SS	SS	SS	SS	SS	SS	SS	SS	0.00002
Al300<br>H12.5	SS	SS	0.00386	SS	SS	SS	SS	SS	SS	SS	SS	SS	SS	SS	0.00007
E20<br>Aml5	SS	SS	0.00013	SS	SS	SS	SS	SS	SS	SS	SS	SS	SS	SS	SS
E20<br>B5	SS	SS	0.00079	SS	SS	SS	SS	SS	SS	SS	SS	SS	SS	SS	0.00002
E20<br>H12.5	SS	SS	SS	SS	SS	SS	SS	SS	SS	SS	SS	SS	SS	SS	SS
L100<br>Aml5	SS	SS	0.00386	SS	SS	SS	SS	SS	SS	SS	SS	SS	SS	SS	0.00004
L100<br>B5	SS	SS	0.00386	SS	SS	SS	SS	SS	SS	SS	SS	SS	SS	SS	SS
L100<br>H12.5	SS	SS	0.00386	SS	SS	SS	SS	SS	SS	SS	SS	SS	SS	SS	0.00002
Al300<br>Aml5/B5	SS	SS	0.00136	SS	SS	SS	SS	SS	SS	SS	SS	SS	SS	SS	0.00002
Al300<br>Aml5/H12.5	SS	SS	0.00630	SS	SS	SS	SS	SS	SS	SS	SS	SS	SS	SS	0.00007
Al300<br>B5/H12.5	SS	SS	0.00232	SS	SS	SS	SS	SS	SS	SS	SS	SS	SS	SS	0.00002
E20<br>Aml5/B5	SS	SS	0.00079	SS	SS	SS	SS	SS	SS	SS	SS	SS	SS	SS	0.00002
E20<br>Aml5/H12.5	SS	SS	SS	SS	SS	SS	SS	SS	SS	SS	SS	SS	SS	SS	SS
E20<br>B5/H12.5	SS	SS	0.00079	SS	SS	SS	SS	SS	SS	SS	SS	SS	SS	SS	0.00002
L100<br>Aml5/B5	SS	SS	0.00386	SS	SS	SS	SS	SS	SS	SS	SS	SS	SS	SS	SS
L100<br>Aml5/H12.5	SS	SS	0.00386	SS	SS	SS	SS	SS	SS	SS	SS	SS	SS	SS	0.00002
L100<br>B5/H12.5	SS	SS	0.00630	SS	SS	SS	SS	SS	SS	SS	SS	SS	SS	SS	0.00004

Al300 = aliskiren 300 mg; **Aml5** = amlodipine 5 mg; **B5** = bisoprolol 5 mg; **E20** = enalapril 20 mg; **H12.5** = hydrochlorothiazide 12.5 mg; **L100** = losartan 100 mg; **SS** = statistically significant ($P < 0.00001$)

Figure S35. Simulated change in plasma aldosterone from baseline to week 4 (mean ± SD, n = 100)

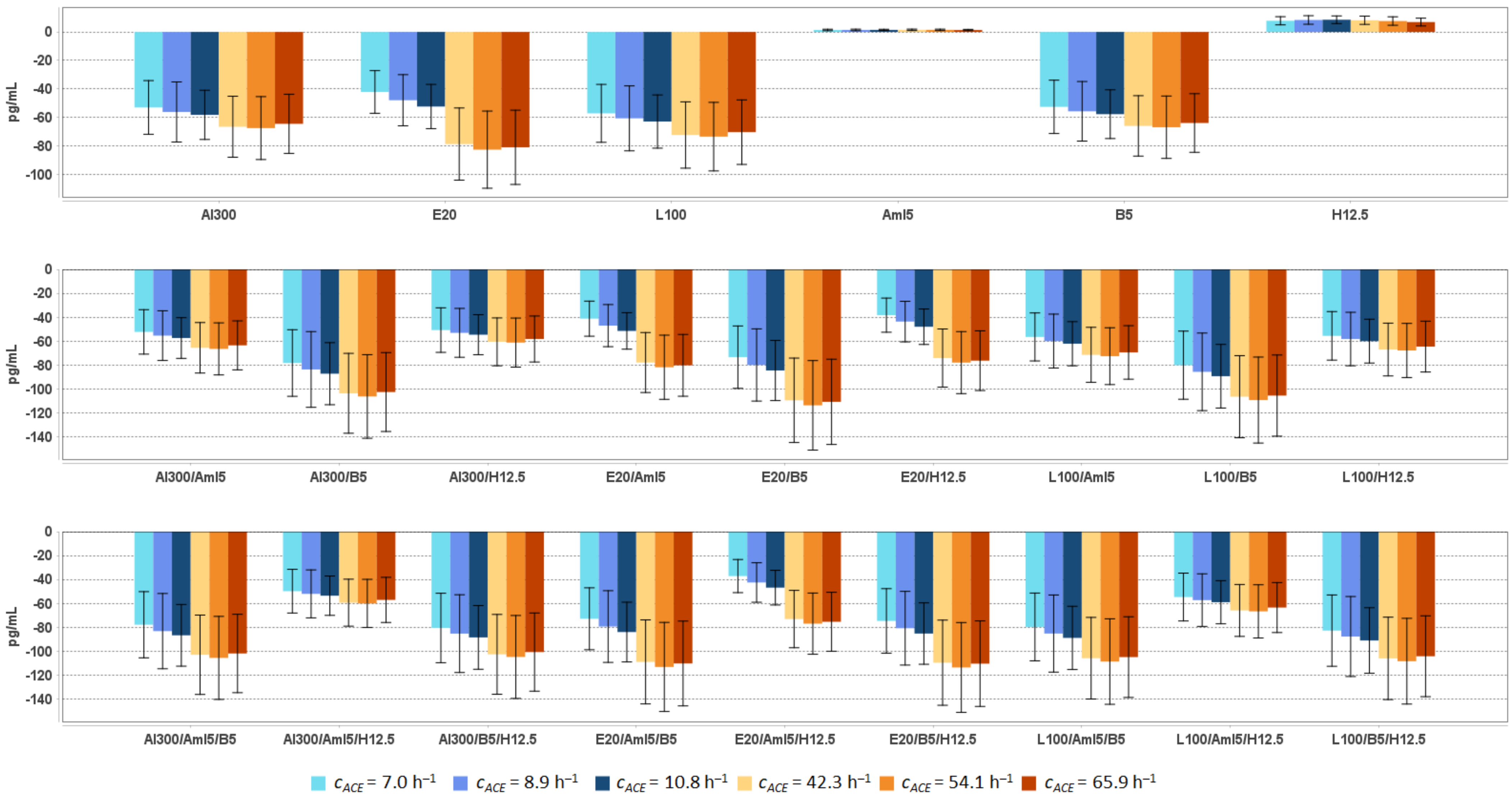

Al300 = aliskiren 300 mg; **Aml5** = amlodipine 5 mg; **B5** = bisoprolol 5 mg; **E20** = enalapril 20 mg; **H12.5** = hydrochlorothiazide 12.5 mg; **L100** = losartan 100 mg

Table S56. Simulated response of plasma aldosterone to antihypertensive therapy in virtual hypertensive subpopulations (n = 100) with different ACE activity, including P-values (Kolmogorov-Smirnov test) for endpoint *vs*. baseline; data are presented as mean ± SD in pg/mL

Regimens	***LII*** **(c_{ACE} = 7.0 h^{-1})**			***LID*** **(c_{ACE} = 8.9 h^{-1})**			***LDD*** **(c_{ACE} = 10.8 h^{-1})**			***HII*** **(c_{ACE} = 42.3 h^{-1})**			***HID*** **(c_{ACE} = 54.1 h^{-1})**			***HDD*** **(c_{ACE} = 65.9 h^{-1})**		
	**Value**	**Change**	***P***	**Value**	**Change**	***P***	**Value**	**Change**	***P***	**Value**	**Change**	***P***	**Value**	**Change**	***P***	**Value**	**Change**	***P***
Baseline	166 ± 59	–	–	172 ± 63	–	–	176 ± 52	–	–	193 ± 62	–	–	196 ± 64	–	–	188 ± 60	–	–
Al300	113 ± 40	-53 ± 19	SS	116 ± 42	-56 ± 21	SS	118 ± 35	-58 ± 17	SS	127 ± 41	-67 ± 21	SS	129 ± 42	-68 ± 22	SS	123 ± 40	-65 ± 21	SS
E20	124 ± 44	-42 ± 15	SS	124 ± 45	-48 ± 18	0.00013	124 ± 37	-52 ± 16	SS	114 ± 37	-79 ± 25	SS	113 ± 37	-83 ± 27	SS	107 ± 34	-81 ± 26	SS
L100	109 ± 39	-57 ± 20	SS	111 ± 41	-61 ± 23	SS	113 ± 34	-63 ± 19	SS	121 ± 39	-72 ± 23	SS	123 ± 40	-74 ± 24	SS	118 ± 38	-70 ± 23	SS
Aml5	167 ± 60	1 ± 1	1.00000	173 ± 64	1 ± 1	0.99963	177 ± 53	1 ± 0	0.99963	195 ± 63	1 ± 1	1.00000	198 ± 65	1 ± 1	0.99376	189 ± 61	1 ± 0	1.00000
B5	113 ± 41	-53 ± 19	SS	116 ± 42	-56 ± 21	SS	118 ± 35	-58 ± 17	SS	127 ± 41	-66 ± 21	SS	129 ± 42	-67 ± 22	SS	124 ± 40	-64 ± 21	SS
H12.5	174 ± 61	8 ± 3	0.81275	180 ± 65	8 ± 3	0.81275	185 ± 54	9 ± 3	0.69937	201 ± 64	8 ± 3	0.58062	204 ± 66	8 ± 3	0.81275	195 ± 62	7 ± 3	0.96707
Al300 Aml5	114 ± 41	-52 ± 19	SS	117 ± 43	-55 ± 21	SS	119 ± 35	-57 ± 17	SS	128 ± 41	-66 ± 21	SS	130 ± 42	-66 ± 22	SS	125 ± 40	-64 ± 20	SS
Al300 B5	88 ± 32	-78 ± 28	SS	88 ± 32	-84 ± 32	SS	89 ± 27	-87 ± 26	SS	90 ± 29	-104 ± 33	SS	90 ± 29	-106 ± 35	SS	86 ± 28	-103 ± 33	SS
Al300 H12.5	115 ± 41	-51 ± 19	SS	119 ± 43	-53 ± 20	0.00002	121 ± 35	-55 ± 17	SS	133 ± 42	-61 ± 20	SS	135 ± 44	-61 ± 21	SS	130 ± 41	-58 ± 19	SS
E20 Aml5	125 ± 45	-41 ± 15	0.00002	125 ± 46	-47 ± 18	0.00013	125 ± 37	-51 ± 15	SS	115 ± 37	-78 ± 25	SS	114 ± 37	-82 ± 27	SS	108 ± 35	-80 ± 26	SS
E20 B5	93 ± 33	-73 ± 26	SS	92 ± 33	-80 ± 30	SS	92 ± 27	-85 ± 25	SS	84 ± 27	-109 ± 35	SS	83 ± 27	-114 ± 37	SS	77 ± 25	-111 ± 36	SS
E20 H12.5	128 ± 45	-38 ± 14	0.00013	128 ± 46	-44 ± 17	0.00045	128 ± 37	-48 ± 15	SS	119 ± 38	-74 ± 24	SS	118 ± 38	-78 ± 26	SS	112 ± 36	-76 ± 25	SS
L100 Aml5	110 ± 39	-56 ± 20	SS	112 ± 41	-60 ± 23	SS	114 ± 34	-62 ± 18	SS	122 ± 39	-71 ± 23	SS	124 ± 40	-73 ± 24	SS	119 ± 38	-69 ± 22	SS
L100 B5	86 ± 31	-80 ± 28	SS	86 ± 31	-86 ± 32	SS	87 ± 26	-89 ± 27	SS	87 ± 28	-106 ± 34	SS	87 ± 28	-109 ± 36	SS	83 ± 27	-106 ± 34	SS
L100 H12.5	110 ± 39	-56 ± 20	SS	114 ± 41	-58 ± 22	SS	116 ± 34	-60 ± 18	SS	126 ± 40	-67 ± 22	SS	128 ± 42	-68 ± 23	SS	123 ± 39	-65 ± 21	SS
Al300 Aml5/B5	88 ± 32	-78 ± 28	SS	89 ± 32	-83 ± 31	SS	89 ± 27	-87 ± 26	SS	90 ± 29	-103 ± 33	SS	91 ± 30	-106 ± 35	SS	86 ± 28	-102 ± 33	SS
Al300 Aml5/H12.5	116 ± 41	-50 ± 18	SS	120 ± 43	-52 ± 20	0.00004	122 ± 36	-54 ± 16	SS	134 ± 43	-59 ± 20	SS	136 ± 44	-60 ± 20	SS	131 ± 42	-57 ± 19	SS
Al300 B5/H12.5	85 ± 30	-81 ± 29	SS	87 ± 31	-85 ± 33	SS	88 ± 26	-89 ± 27	SS	91 ± 29	-103 ± 33	SS	91 ± 30	-105 ± 35	SS	87 ± 28	-101 ± 33	SS
E20 Aml5/B5	93 ± 34	-73 ± 26	SS	93 ± 33	-79 ± 30	SS	92 ± 28	-84 ± 25	SS	84 ± 27	-109 ± 35	SS	83 ± 27	-113 ± 37	SS	78 ± 25	-110 ± 35	SS
E20 Aml5/H12.5	129 ± 45	-37 ± 14	0.00025	129 ± 47	-43 ± 17	0.00136	129 ± 38	-47 ± 15	SS	120 ± 38	-73 ± 24	SS	119 ± 39	-77 ± 26	SS	113 ± 36	-75 ± 25	SS
E20 B5/H12.5	91 ± 32	-75 ± 27	SS	91 ± 33	-81 ± 31	SS	91 ± 27	-85 ± 26	SS	84 ± 26	-110 ± 36	SS	83 ± 27	-114 ± 38	SS	78 ± 25	-110 ± 36	SS
L100 Aml5/B5	86 ± 31	-80 ± 28	SS	87 ± 31	-85 ± 32	SS	87 ± 26	-89 ± 26	SS	87 ± 28	-106 ± 34	SS	88 ± 29	-109 ± 36	SS	83 ± 27	-105 ± 34	SS
L100 Aml5/H12.5	111 ± 39	-55 ± 20	SS	115 ± 41	-57 ± 22	SS	117 ± 34	-59 ± 18	SS	127 ± 40	-66 ± 22	SS	130 ± 42	-67 ± 22	SS	125 ± 40	-64 ± 21	SS
L100 B5/H12.5	83 ± 30	-83 ± 30	SS	84 ± 30	-88 ± 33	SS	85 ± 25	-91 ± 27	SS	87 ± 28	-106 ± 34	SS	88 ± 28	-108 ± 36	SS	84 ± 27	-104 ± 34	SS

Al300 = aliskiren 300 mg; **Aml5** = amlodipine 5 mg; **B5** = bisoprolol 5 mg; **E20** = enalapril 20 mg; **H12.5** = hydrochlorothiazide 12.5 mg; **L100** = losartan 100 mg; **SS** = statistically significant (P < 0.00001)

Table S57. *P*-values calculated using the Kolmogorov-Smirnov test for changes in plasma aldosterone in subpopulations (n = 100) with different ACE activity receiving the same regimens. Case 1: c_{ACE} = 7.0 h^{-1} (*LII*), case 2: c_{ACE} = 8.9 h^{-1} (*LID*), case 3: c_{ACE} = 10.8 h^{-1} (*LDD*), case 4: c_{ACE} = 42.3 h^{-1} (*HII*), case 5: c_{ACE} = 54.1 h^{-1} (*HID*), case 6: c_{ACE} =65.9 h^{-1} (*HDD*). *P*-value for case *i vs*. case *j* is denoted P_{ij}.

Regimens	P_{12}	P_{13}	P_{23}	P_{14}	P_{15}	P_{16}	P_{24}	P_{25}	P_{26}	P_{34}	P_{35}	P_{36}	P_{45}	P_{46}	P_{56}
Al300	0.58062	0.02431	0.05410	0.00007	SS	0.00136	0.00232	0.00079	0.01581	0.00045	0.00013	0.02431	0.90621	0.46756	0.46756
E20	0.11113	0.00002	0.01008	SS	SS	SS	SS	SS	SS	SS	SS	SS	0.46756	0.81275	0.81275
L100	0.58062	0.02431	0.05410	0.00007	SS	0.00136	0.00136	0.00045	0.01581	0.00025	0.00007	0.02431	0.90621	0.46756	0.46756
Aml5	0.81275	0.58062	0.28093	0.15454	0.21055	0.81275	0.15454	0.28093	0.46756	0.58062	0.69937	0.69937	0.90621	0.11113	0.05410
B5	0.58062	0.02431	0.05410	0.00007	SS	0.00136	0.00232	0.00079	0.01581	0.00045	0.00013	0.02431	0.90621	0.46756	0.46756
H12.5	0.46756	0.15454	0.81275	0.90621	0.81275	0.11113	0.90621	0.21055	0.00386	0.46756	0.03663	0.00025	0.28093	0.01008	0.21055
Al300<br>Aml5	0.58062	0.02431	0.05410	0.00007	SS	0.00136	0.00232	0.00079	0.01581	0.00045	0.00013	0.02431	0.90621	0.46756	0.36672
Al300<br>B5	0.46756	0.01008	0.02431	SS	SS	0.00002	0.00025	0.00004	0.00232	0.00004	SS	0.00136	0.90621	0.69937	0.69937
Al300<br>H12.5	0.69937	0.05410	0.05410	0.00025	0.00013	0.03663	0.00630	0.00630	0.07832	0.00232	0.00630	0.15454	0.99376	0.46756	0.28093
E20<br>Aml5	0.11113	0.00002	0.01008	SS	SS	SS	SS	SS	SS	SS	SS	SS	0.46756	0.81275	0.81275
E20<br>B5	0.36672	0.00232	0.01581	SS	SS	SS	SS	SS	SS	SS	SS	SS	0.58062	0.96707	0.81275
E20<br>H12.5	0.15454	SS	0.00630	SS	SS	SS	SS	SS	SS	SS	SS	SS	0.36672	0.69937	0.69937
L100<br>Aml5	0.58062	0.02431	0.05410	0.00007	SS	0.00136	0.00136	0.00045	0.01581	0.00025	0.00007	0.02431	0.90621	0.46756	0.46756
L100<br>B5	0.46756	0.01008	0.02431	SS	SS	SS	0.00025	0.00004	0.00232	0.00004	SS	0.00079	0.90621	0.69937	0.69937
L100<br>H12.5	0.69937	0.05410	0.05410	0.00025	0.00007	0.02431	0.00630	0.00386	0.05410	0.00136	0.00232	0.15454	0.96707	0.46756	0.36672
Al300<br>Aml5/B5	0.46756	0.01008	0.02431	SS	SS	0.00002	0.00025	0.00004	0.00386	0.00004	SS	0.00136	0.90621	0.69937	0.69937
Al300<br>Aml5/H12.5	0.69937	0.05410	0.05410	0.00025	0.00013	0.03663	0.00630	0.00630	0.07832	0.00232	0.00386	0.15454	0.99376	0.46756	0.28093
Al300<br>B5/H12.5	0.69937	0.03663	0.05410	0.00004	SS	0.00045	0.00079	0.00025	0.01008	0.00025	0.00007	0.00630	0.90621	0.69937	0.46756
E20<br>Aml5/B5	0.36672	0.00232	0.01581	SS	SS	SS	SS	SS	SS	SS	SS	SS	0.58062	0.96707	0.81275
E20<br>Aml5/H12.5	0.15454	SS	0.00630	SS	SS	SS	SS	SS	SS	SS	SS	SS	0.36672	0.81275	0.69937
E20<br>B5/H12.5	0.36672	0.00630	0.03663	SS	SS	SS	0.00002	SS	SS	SS	SS	SS	0.81275	0.90621	0.81275
L100<br>Aml5/B5	0.46756	0.01008	0.02431	SS	SS	SS	0.00025	0.00004	0.00232	0.00004	SS	0.00079	0.90621	0.81275	0.69937
L100<br>Aml5/H12.5	0.69937	0.05410	0.05410	0.00025	0.00007	0.01581	0.00630	0.00386	0.05410	0.00136	0.00232	0.11113	0.96707	0.46756	0.36672
L100<br>B5/H12.5	0.58062	0.03663	0.05410	0.00004	SS	0.00045	0.00079	0.00025	0.01008	0.00013	0.00004	0.00630	0.90621	0.69937	0.58062

Al300 = aliskiren 300 mg; **Aml5** = amlodipine 5 mg; **B5** = bisoprolol 5 mg; **E20** = enalapril 20 mg; **H12.5** = hydrochlorothiazide 12.5 mg; **L100** = losartan 100 mg; **SS** = statistically significant (P < 0.00001)

Table S58. Pearson correlation coefficients of RAAS parameters with a decrease in diastolic blood pressure during simulated treatment of virtual subpopulations (n = 100) with aliskiren 300 mg (A300), amlodipine 5 mg (A5), bisoprolol 5 mg (B5), enalapril 20 mg (E20), HCTZ 12.5 mg (H12.5), losartan 100 mg (L100), and combinations of these drugs.

c_{ACE} (h^{-1})	A300	A5	B5	E20	H12.5	L100	A300 A5	A300 B5	A300 H12.5	E20 A5	E20 B5	E20 H12.5	L100 A5	L100 B5	L100 H12.5	A300 A5 B5	A300 A5 H12.5	A300 B5 H12.5	E20 A5 B5	E20 A5 H12.5	E20 B5 H12.5	L100 A5 B5	L100 A5 H12.5	L100 B5 H12.5
Plasma renin activity																								
**7.0**	0.25	-0.07	0.18	0.32	0.05	0.21	0.15	-0.35	0.20	0.16	-0.15	0.19	0.13	-0.43	0.20	-0.34	0.15	0.04	-0.14	0.14	0.12	-0.43	0.15	-0.01
**8.9**	0.42	-0.15	0.37	0.47	-0.27	0.38	0.25	-0.23	0.07	0.25	-0.03	0.05	0.24	-0.35	0.08	-0.14	0.00	0.00	0.02	-0.02	0.04	-0.26	0.01	-0.05
**10.8**	0.40	-0.13	0.45	0.44	-0.22	0.35	0.18	-0.13	0.04	0.20	0.00	0.04	0.15	-0.25	0.04	-0.15	-0.06	-0.02	-0.05	-0.06	0.01	-0.24	-0.05	-0.06
**42.3**	0.49	-0.08	0.41	0.45	0.01	0.47	0.31	0.35	0.29	0.29	0.30	0.30	0.31	0.33	0.29	0.28	0.19	0.29	0.24	0.19	0.28	0.26	0.19	0.29
**54.1**	0.68	-0.08	0.63	0.62	-0.21	0.66	0.51	0.48	0.29	0.48	0.42	0.32	0.50	0.46	0.31	0.41	0.19	0.30	0.36	0.21	0.30	0.39	0.20	0.30
**65.9**	0.71	-0.01	0.56	0.67	-0.11	0.70	0.65	0.53	0.43	0.61	0.46	0.46	0.64	0.51	0.44	0.49	0.40	0.43	0.43	0.42	0.42	0.47	0.42	0.43
Plasma angiotensin I																								
**7.0**	0.25	-0.07	0.18	0.32	0.05	0.21	0.15	-0.35	0.20	0.16	-0.15	0.19	0.13	-0.43	0.20	-0.34	0.15	0.04	-0.14	0.14	0.12	-0.43	0.15	-0.01
**8.9**	0.42	-0.15	0.37	0.47	-0.27	0.38	0.25	-0.23	0.07	0.25	-0.03	0.05	0.24	-0.35	0.08	-0.14	0.00	0.00	0.02	-0.02	0.04	-0.26	0.01	-0.05
**10.8**	0.40	-0.13	0.45	0.44	-0.22	0.35	0.18	-0.13	0.04	0.20	0.00	0.04	0.15	-0.25	0.04	-0.15	-0.06	-0.02	-0.05	-0.06	0.01	-0.24	-0.05	-0.06
**42.3**	0.49	-0.08	0.41	0.45	0.01	0.47	0.31	0.35	0.29	0.29	0.30	0.30	0.31	0.33	0.29	0.28	0.19	0.29	0.24	0.19	0.28	0.26	0.19	0.29
**54.1**	0.68	-0.08	0.63	0.62	-0.21	0.66	0.51	0.48	0.29	0.48	0.42	0.32	0.50	0.46	0.31	0.41	0.19	0.30	0.36	0.21	0.30	0.39	0.20	0.30
**65.9**	0.71	-0.01	0.56	0.67	-0.11	0.70	0.65	0.53	0.43	0.61	0.46	0.46	0.64	0.51	0.44	0.49	0.40	0.43	0.43	0.42	0.42	0.47	0.42	0.43
Plasma angiotensin II																								
**7.0**	0.25	-0.07	0.18	0.32	0.05	0.21	0.15	-0.35	0.20	0.16	-0.15	0.19	0.13	-0.43	0.20	-0.34	0.15	0.04	-0.14	0.14	0.12	-0.43	0.15	-0.01
**8.9**	0.42	-0.15	0.37	0.47	-0.27	0.38	0.25	-0.23	0.07	0.25	-0.03	0.05	0.24	-0.35	0.08	-0.14	0.00	0.00	0.02	-0.02	0.04	-0.26	0.01	-0.05
**10.8**	0.40	-0.13	0.45	0.44	-0.22	0.35	0.18	-0.13	0.04	0.20	0.00	0.04	0.15	-0.25	0.04	-0.15	-0.06	-0.02	-0.05	-0.06	0.01	-0.24	-0.05	-0.06
**42.3**	0.49	-0.08	0.41	0.45	0.01	0.47	0.31	0.35	0.29	0.29	0.30	0.30	0.31	0.33	0.29	0.28	0.19	0.29	0.24	0.19	0.28	0.26	0.19	0.29
**54.1**	0.68	-0.08	0.63	0.62	-0.21	0.66	0.51	0.48	0.29	0.48	0.42	0.32	0.50	0.46	0.31	0.41	0.19	0.30	0.36	0.21	0.30	0.39	0.20	0.30
**65.9**	0.71	-0.01	0.56	0.67	-0.11	0.70	0.65	0.53	0.43	0.61	0.46	0.46	0.64	0.51	0.44	0.49	0.40	0.43	0.43	0.42	0.42	0.47	0.42	0.43
Plasma aldosterone																								
**7.0**	-0.02	0.02	-0.12	-0.03	0.09	-0.01	0.03	-0.04	0.09	0.02	-0.07	0.09	0.03	-0.02	0.09	-0.03	0.10	0.06	-0.04	0.09	0.06	-0.02	0.10	0.06
**8.9**	-0.17	-0.06	-0.17	-0.15	0.05	-0.18	-0.11	-0.27	0.00	-0.10	-0.26	0.01	-0.12	-0.27	-0.01	-0.22	0.05	-0.08	-0.20	0.05	-0.06	-0.23	0.05	-0.09
**10.8**	-0.01	0.02	-0.01	-0.01	0.05	-0.02	0.01	-0.08	0.06	0.01	-0.07	0.06	0.00	-0.10	0.06	-0.05	0.05	0.03	-0.04	0.05	0.03	-0.06	0.05	0.02
**42.3**	0.04	-0.12	0.04	0.03	-0.07	0.03	-0.03	0.04	0.01	-0.02	0.03	0.01	-0.02	0.04	0.01	0.01	-0.03	0.04	0.01	-0.03	0.04	0.01	-0.03	0.04
**54.1**	0.08	0.02	0.03	0.09	-0.01	0.08	0.10	0.08	0.06	0.12	0.08	0.08	0.11	0.08	0.07	0.13	0.08	0.11	0.13	0.10	0.12	0.13	0.08	0.12
**65.9**	0.01	-0.05	0.03	0.00	0.05	0.00	-0.02	0.01	0.06	-0.02	0.01	0.05	-0.02	0.01	0.05	-0.02	0.05	0.04	-0.03	0.05	0.03	-0.02	0.05	0.04

Table S59. Pearson correlation coefficients of RAAS parameters with a decrease in systolic blood pressure during simulated treatment of virtual subpopulations (n = 100) with aliskiren 300 mg (A300), amlodipine 5 mg (A5), bisoprolol 5 mg (B5), enalapril 20 mg (E20), HCTZ 12.5 mg (H12.5), losartan 100 mg (L100), and combinations of these drugs.

c_{ACE} (h^{-1})	A300	A5	B5	E20	H12.5	L100	A300 A5	A300 B5	A300 H12.5	E20 A5	E20 B5	E20 H12.5	L100 A5	L100 B5	L100 H12.5	A300 A5 B5	A300 A5 H12.5	A300 B5 H12.5	E20 A5 B5	E20 A5 H12.5	E20 B5 H12.5	L100 A5 B5	L100 A5 H12.5	L100 B5 H12.5
Plasma renin activity																								
**7.0**	0.23	-0.05	0.20	0.19	0.15	0.24	0.15	0.27	0.20	0.10	0.28	0.18	0.17	0.25	0.21	0.22	0.16	0.26	0.23	0.14	0.25	0.22	0.17	0.26
**8.9**	0.24	-0.13	0.19	0.19	-0.26	0.26	0.13	0.37	-0.16	0.07	0.35	-0.20	0.16	0.37	-0.14	0.29	-0.16	0.06	0.27	-0.19	0.02	0.29	-0.13	0.08
**10.8**	0.17	-0.14	0.05	0.14	-0.11	0.20	0.04	0.28	-0.06	-0.01	0.27	-0.08	0.07	0.29	-0.04	0.20	-0.10	0.07	0.19	-0.11	0.05	0.21	-0.08	0.08
**42.3**	0.18	0.02	0.10	0.24	0.12	0.21	0.16	0.31	0.14	0.22	0.33	0.17	0.19	0.32	0.15	0.30	0.10	0.26	0.31	0.13	0.28	0.30	0.12	0.27
**54.1**	0.01	-0.11	-0.04	0.13	-0.11	0.06	-0.10	0.26	-0.16	0.01	0.29	-0.12	-0.05	0.27	-0.15	0.17	-0.19	0.02	0.21	-0.16	0.08	0.19	-0.18	0.04
**65.9**	-0.01	-0.07	-0.05	0.15	0.06	0.05	0.00	0.29	0.02	0.15	0.34	0.10	0.06	0.31	0.05	0.31	0.03	0.26	0.36	0.09	0.32	0.33	0.05	0.29
Plasma angiotensin I																								
**7.0**	0.23	-0.05	0.20	0.19	0.15	0.24	0.15	0.27	0.20	0.10	0.28	0.18	0.17	0.25	0.21	0.22	0.16	0.26	0.23	0.14	0.25	0.22	0.17	0.26
**8.9**	0.24	-0.13	0.19	0.19	-0.26	0.26	0.13	0.37	-0.16	0.07	0.35	-0.20	0.16	0.37	-0.14	0.29	-0.16	0.06	0.27	-0.19	0.02	0.29	-0.13	0.08
**10.8**	0.17	-0.14	0.05	0.14	-0.11	0.20	0.04	0.28	-0.06	-0.01	0.27	-0.08	0.07	0.29	-0.04	0.20	-0.10	0.07	0.19	-0.11	0.05	0.21	-0.08	0.08
**42.3**	0.18	0.02	0.10	0.24	0.12	0.21	0.16	0.31	0.14	0.22	0.33	0.17	0.19	0.32	0.15	0.30	0.10	0.26	0.31	0.13	0.28	0.30	0.12	0.27
**54.1**	0.01	-0.11	-0.04	0.13	-0.11	0.06	-0.10	0.26	-0.16	0.01	0.29	-0.12	-0.05	0.27	-0.15	0.17	-0.19	0.02	0.21	-0.16	0.08	0.19	-0.18	0.04
**65.9**	-0.01	-0.07	-0.05	0.15	0.06	0.05	0.00	0.29	0.02	0.15	0.34	0.10	0.06	0.31	0.05	0.31	0.03	0.26	0.36	0.09	0.32	0.33	0.05	0.29
Plasma angiotensin II																								
**7.0**	0.23	-0.05	0.20	0.19	0.15	0.24	0.15	0.27	0.20	0.10	0.28	0.18	0.17	0.25	0.21	0.22	0.16	0.26	0.23	0.14	0.25	0.22	0.17	0.26
**8.9**	0.24	-0.13	0.19	0.19	-0.26	0.26	0.13	0.37	-0.16	0.07	0.35	-0.20	0.16	0.37	-0.14	0.29	-0.16	0.06	0.27	-0.19	0.02	0.29	-0.13	0.08
**10.8**	0.17	-0.14	0.05	0.14	-0.11	0.20	0.04	0.28	-0.06	-0.01	0.27	-0.08	0.07	0.29	-0.04	0.20	-0.10	0.07	0.19	-0.11	0.05	0.21	-0.08	0.08
**42.3**	0.18	0.02	0.10	0.24	0.12	0.21	0.16	0.31	0.14	0.22	0.33	0.17	0.19	0.32	0.15	0.30	0.10	0.26	0.31	0.13	0.28	0.30	0.12	0.27
**54.1**	0.01	-0.11	-0.04	0.13	-0.11	0.06	-0.10	0.26	-0.16	0.01	0.29	-0.12	-0.05	0.27	-0.15	0.17	-0.19	0.02	0.21	-0.16	0.08	0.19	-0.18	0.04
**65.9**	-0.01	-0.07	-0.05	0.15	0.06	0.05	0.00	0.29	0.02	0.15	0.34	0.10	0.06	0.31	0.05	0.31	0.03	0.26	0.36	0.09	0.32	0.33	0.05	0.29
Plasma aldosterone																								
**7.0**	0.01	0.02	0.08	0.00	0.14	0.01	0.04	0.07	0.14	0.03	0.07	0.14	0.04	0.07	0.14	0.09	0.12	0.16	0.09	0.12	0.15	0.09	0.12	0.16
**8.9**	-0.13	0.05	-0.10	-0.14	0.17	-0.12	-0.03	-0.06	0.13	-0.03	-0.07	0.14	-0.03	-0.05	0.12	-0.03	0.14	0.08	-0.03	0.15	0.09	-0.02	0.13	0.07
**10.8**	-0.12	0.11	-0.08	-0.13	0.06	-0.11	-0.04	-0.02	0.02	-0.03	-0.04	0.03	-0.04	-0.01	0.02	-0.02	0.03	0.01	-0.03	0.04	0.01	-0.02	0.03	0.01
**42.3**	0.08	-0.07	0.05	0.11	-0.05	0.10	0.04	0.12	-0.01	0.07	0.12	0.01	0.06	0.12	0.00	0.11	-0.02	0.07	0.12	0.01	0.09	0.12	-0.01	0.08
**54.1**	0.01	-0.12	0.04	0.01	-0.07	0.01	-0.09	0.04	-0.07	-0.07	0.05	-0.07	-0.09	0.04	-0.07	-0.02	-0.08	-0.05	-0.01	-0.08	-0.04	-0.02	-0.08	-0.05
**65.9**	-0.04	-0.11	-0.05	-0.04	0.04	-0.04	-0.11	-0.04	0.03	-0.09	-0.04	0.03	-0.10	-0.04	0.03	-0.07	-0.01	0.02	-0.05	-0.02	0.02	-0.06	-0.01	0.02
