# Supplementary material for: Mathematical modelling of the influence of ACE I/D polymorphism on blood pressure and antihypertensive therapy
Source: Heliyon. 2024 Apr 23;10(9):e29988. doi: 10.1016/j.heliyon.2024.e29988 (PMC11068647; doi:10.1016/j.heliyon.2024.e29988)
Supplement: Multimedia component 2 [file mmc2.pdf]

Supplementary File 2 – Model Description

Mathematical modeling of the influence of *ACE I/D* polymorphism on blood pressure and antihypertensive therapy

Elena Kutumova*, Anna Kovaleva, Ruslan Sharipov, Galina Lifshits, Fedor Kolpakov

*** Corresponding Author:** elena.kutumova@biouml.org

Table S1. Mathematical functions of the model

Functions	Description
$mass_{elasticity}\left(e,m\right)=\frac{29.97\cdot e}{m^{0.75}}$	Allometric scaling of cardiac and vascular elasticity as a function of body mass *m* and the average normal elasticity *e*.
$mass_{volume}\left(v,m\right)=\frac{m}{70}\cdot v$	Allometric scaling relative to body mass *m*, considering the average human weight of 70 kg.
$mass_{conductivity}\left(c,m\right)=0.042\cdot m^{0.75}\cdot c$	Allometric scaling of cardiac and vascular conductivity as a function of body mass *m* and the average normal conductivity *c*.
$r_{plus}\left(a,b,x,x0\right)=\frac{1-e^{-a\cdot(x-x0)}}{1+b\cdot e^{-a\cdot(x-x0)}}$	Receptor activity function.
$r_{minus}\left(a,b,x,x_0\right)=1-r_{plus}\left(a,b,x,x_0\right)$	Receptor activity function.
$atrium_{pulse}\left(T,T_S,P_{AL},t\right)=\begin{cases}0, & t\le T-0.2\cdot\left(T-T_S\right)\\ 0.1\cdot P_{AL}\cdot\left\vert T-t\right\vert^{1/2}, & otherwise\end{cases}$	Function for calculating pulse waves of the left and right atrium depending on the duration of the cardiac cycle *T*, the actual duration of left or right ventricular systole T_S, systemic arterial pressure P_{AL}, and the current time of the cardiac cycle *t*.
$valve\left(P_{in},P_{out},R_{factor},YY\right)=\begin{cases}YY\cdot\left(P_{in}-P_{out}\right), & P_{in}\ge P_{out}\\ R_{factor}\cdot YY\cdot\left(P_{in}-P_{out}\right), & otherwise\end{cases}$	Function for calculating blood flow through heart valves taking into account inlet pressure P_{in}, outlet pressure P_{out}, conductivity *YY* and regurgitation coefficient R_{factor}. The second part of the formula describes the reverse flow through the valve (regurgitation, $0<R_{factor}<0.3$). Under normal conditions, $R_{factor}=0$.
$sigm\left(x\right)=\frac{1}{1+e^{-x}}$	Logistic function.
$\operatorname{sgn}\left(x,y\right)=\begin{cases}0, & x\le 0\ and\ y\le 0\\ y, & otherwise\end{cases}$	Function used to calculate oxygen debt.

Table S2. Model equations

№	Equations	Description	Modification[1]
**Cardiovascular module, systemic circulation**			
001	$\frac{dV_{AL}}{dt} = F_{HLAL} - F_{ALVL}$	Change in blood volume in the systemic arteries (V_{AL}) is the difference between incoming (F_{HLAL}) and outgoing (F_{ALVL}) blood flows.	–
002	$V_{AL}(0) = 0.13 \cdot V$	Starting value[2] of the blood volume in the systemic arteries is 13% of the total blood volume *V*.	–
003	$G_{AL} = mass_{elasticity}(G_{AL0} + A_9 \cdot H, m) \cdot (1 - B_{blocker\_st})$	G_{AL} – systemic arterial elasticity, G_{AL0} – basic elasticity, A_9 – systemic arterial tone, *H* – neurohumoral factor, *m* – body mass, $B_{blocker\_st}$ – effect of the β-blocker bisoprolol on arterial stiffness.	Reduction of arterial stiffness with bisoprolol (Asmar et al., 1991; Kahonen et al., 2000; Palmieri et al., 2004; Ong et al., 2011; Zhou et al., 2013; Eguchi et al., 2015) was added.
004	$\omega_{AL} = \omega_{AL\_nom} - mass_{volume}(A_8, m) \cdot H$	ω_{AL} – unstressed volume of the systemic arteries, ω_{AL_nom} – nominal unstressed volume, A_8 – sympathetic sensitivity of the systemic arteries, *m* – body mass, *H* – neurohumoral factor.	–
005	$\omega_{AL\_nom}(0) = k_{AL} \cdot V_{AL}$	Starting value of the nominal unstressed volume of the systemic arteries without nervous and hormonal influences is a fraction k_{AL} of V_{AL}.	–
006	$P_{AL} = G_{AL} \cdot (V_{AL} - \omega_{AL})$	P_{AL} – systemic arterial pressure, G_{AL} – systemic arterial elasticity, V_{AL} – systemic arterial volume, ω_{AL} – unstressed volume of the systemic arteries.	–
007	$Vis = 1.23 \cdot \left(1 - \frac{Hct}{99}\right)^{-n},\ n = 1.7 + 9.86 \cdot \exp(-0.0607 \cdot Hct)$	Blood viscosity *Vis* is calculated as a function of hematocrit *Hct* at a plasma viscosity of 1.23 cP.	–
008	$F_{ALVL} = \frac{(P_{AL} - P_{VL})}{\frac{Vis}{Vis_{norm}} \cdot R_{ALVL}}$	F_{ALVL} – blood flow through the systemic microvessels, R_{ALVL} – resistance of the systemic microvessels, P_{AL} – systemic arterial pressure, P_{VL} – systemic venous pressure, Vis/Vis_{norm} – normalized blood viscosity.	–
009	$R_{ALVL} = \frac{1}{Y_{ALVL}} \cdot \psi_{AT1\_ALVL} \cdot (1 - CCB_{sys}) \cdot (1 - Diuretic_{sys})$	R_{ALVL} – resistance of the systemic microvessels, ψ_{AT1_ALVL} – effect of angiotensin II bound to AT1 receptors in the vascular smooth muscle, CCB_{sys} – effect of the calcium channel blocker amlodipine, $Diuretic_{sys}$ – effect of the thiazide diuretic hydrochlorothiazide (HCTZ).	The vasodilatory effects of amlodipine and HCTZ were taken from the model extension (Kutumova et al., 2022)
010	$\psi_{AT1\_ALVL} = A_{AT1\_ALVL} + B_{AT1\_ALVL} \cdot AT1\_ANGII - \frac{C_{AT1\_ALVL}}{AT1\_ANGII}$	ψ_{AT1_ALVL} – effect of AT1-bound angiotensin II (*AT1_ANGII*) on R_{ALVL}. A_{AT1_ALVL}, B_{AT1_ALVL}, and C_{AT1_ALVL} are constant.	–

[1] Modification from the basic model by Kutumova et al. (2021)
[2] Formulas for calculating starting values are used to generate virtual patients and are not taken into account when considering the model in equilibrium.

011	$Y_{ALVL} = mass_{conductivity}\left(Y_{ALVL0} - A_3 \cdot H + A_4 \cdot DO_2, m\right)$	Y_{ALVL} – conductivity of the systemic microvessels, Y_{ALVL0} – basic conductivity, A_3 – sympathetic sensitivity of the systemic microvessels (dependence on nervous and hormonal influences), H – neurohumoral factor, A_4 – oxygen-deficient sensitivity of the systemic microvessels (dependence on oxygen debt DO_2), m – body mass.	–
012	$V_{VL} = V - V_{AL} - V_{AR} - V_{VR} - V_{HL} - V_{HR}$	Blood volume in the systemic veins V_{VL} is calculated by reducing the total circulating blood volume V by the volumes of all other parts of the cardiovascular system, including systemic arteries (V_{AL}), pulmonary arteries (V_{AR}), pulmonary veins (V_{VR}), left ventricle (V_{HL}) and right ventricle (V_{HR}).	–
013	$G_{VL} = mass_{elasticity}\left(G_{VL0} + A_{11} \cdot H, m\right)$	G_{VL} – systemic venous elasticity, G_{VL0} – basic elasticity, A_{11} – venous tone, H – neurohumoral factor, m – body mass.	–
014	$\omega_{VL}(0) = k_{VL} \cdot V_{VL}$	Starting value of the unstressed volume of the systemic veins, calculated as a fraction k_{VL} of V_{VL}.	–
015	$P_{VL} = P_0 + RA_{PULSE} + G_{VL} \cdot \left(V_{VL} - \omega_{VL}\right)$	P_{VL} – systemic venous pressure, P_0 – basic pressure, RA_{PULSE} – pulse wave of the right atrium, G_{VL} – systemic venous elasticity, V_{VL} – blood volume in the systemic veins, ω_{VL} – unstressed volume of the systemic veins.	–
016	$MAP = \frac{P_S + 2 \cdot P_D}{3}$	MAP – mean arterial pressure, P_S – systolic blood pressure, P_D – diastolic blood pressure.	–
017	$TPR = MAP/CO$	Total peripheral resistance TPR is the ratio of mean arterial pressure MAP to cardiac output CO.	–
018	$SVR = \frac{1}{Y_{HLAL}} + R_{ALVL} + \frac{1}{Y_{VLHR}}$	SVR – systemic vascular resistance, Y_{HLAL} – conductivity (reciprocal of resistance) of the aortic valve and systemic arteries, R_{ALVL} – resistance of the systemic microvessels, Y_{VLHR} – conductivity of the tricuspid valve and systemic veins.	–
**Cardiovascular module, pulmonary circulation**			
019	$\frac{dV_{AR}}{dt} = F_{HRAR} - F_{ARVR}$	Change in blood volume in the pulmonary arteries (V_{AR}) is the difference between incoming (F_{HRAR}) and outgoing (F_{ARVR}) blood flows.	–
020	$V_{AR}(0) = 0.035 \cdot V$	Starting value of the blood volume in the pulmonary arteries is 3.5% of the total blood volume V.	–
021	$G_{AR} = mass_{elasticity}\left(G_{AR0} + A_{19} \cdot H, m\right)$	G_{AR} – pulmonary arterial elasticity, G_{AR0} – basic elasticity, A_{19} – pulmonary arterial tone, H – neurohumoral factor, m – body mass.	–
022	$\omega_{AR} = \omega_{AR\_nom} - mass_{volume}\left(A_{18}, m\right) \cdot H$	ω_{AR} – unstressed volume of the pulmonary arteries, ω_{AR_nom} – nominal unstressed volume, A_{18} – sympathetic sensitivity of the pulmonary arteries, m – body mass, H – neurohumoral factor.	–
023	$\omega_{AR\_nom}(0) = k_{AR} \cdot V_{AR}$	Starting value of the nominal unstressed volume of the pulmonary arteries without nervous and hormonal influences is a fraction k_{AR} of V_{AR}.	–

024	$P_{AR} = G_{AR} \cdot (V_{AR} - \omega_{AR})$	P_{AR} – pulmonary arterial pressure, G_{AR} – pulmonary arterial elasticity, V_{AR} – blood volume in pulmonary arteries, ω_{AR} – unstressed volume of the pulmonary arteries.	–
025	$F_{ARVR} = \frac{Y_{ARVR}}{Vis/Vis_{norm}} \cdot (P_{AR} - P_{VR})$	F_{ARVR} – blood flow through the pulmonary microvessels, Y_{ARVR} – conductivity of the pulmonary microvessels, P_{AR} – pulmonary arterial pressure, P_{VR} – pulmonary venous pressure, Vis/Vis_{norm} – normalized blood viscosity.	–
026	$Y_{ARVR} = mass_{conductivity}(Y_{ARVR0} - A_{13} \cdot H + A_{14} \cdot DO_2, m)$	Y_{ARVR} – conductivity of the pulmonary microvessels, Y_{ARVR0} – basic conductivity, A_{13} – sympathetic sensitivity of the pulmonary microvessels (dependence on nervous and hormonal influences), H – neurohumoral factor, A_{14} – oxygen-deficient sensitivity of the pulmonary microvessels (dependence on oxygen debt DO_2), m – body mass.	–
027	$\frac{dV_{VR}}{dt} = F_{ARVR} - F_{VRHL}$	Change in blood volume in the pulmonary veins (V_{VR}) is the difference between incoming (F_{ARVR}) and outgoing (F_{VRHL}) blood flows.	–
028	$V_{VR}(0) = 0.065 \cdot V$	Starting value of the blood volume in the pulmonary veins is 6.5% of the total blood volume V.	–
029	$G_{VR} = mass_{elasticity}(G_{VR0} + A_{11} \cdot H, m)$	G_{VR} – pulmonary venous elasticity, G_{VR0} – basic elasticity, A_{11} – venous tone, H – neurohumoral factor, m – body mass.	–
030	$\omega_{VR}(0) = k_{VR} \cdot V_{VR}$	Starting value of the unstressed volume of the pulmonary veins, calculated as a fraction k_{VR} of V_{VR}.	–
031	$P_{VR} = P_0 + LA_{PULSE} + G_{VR} \cdot (V_{VR} - \omega_{VR})$	P_{VR} – pulmonary venous pressure, P_0 – basic pressure, LA_{PULSE} – pulse wave of the left atrium, G_{VR} – pulmonary venous elasticity, V_{VR} – blood volume in the pulmonary veins, ω_{VR} – unstressed volume of the pulmonary veins.	–
032	$PVR = \frac{1}{Y_{HRAR}} + \frac{1}{Y_{ARVR}} + \frac{1}{Y_{VRHL}}$	PVR – pulmonary vascular resistance, Y_{HRAR} – conductivity of the pulmonary valve and pulmonary arteries, Y_{ARVR} – conductivity of the pulmonary microvessels, Y_{VRHL} – conductivity of the mitral valve and pulmonary veins.	–
**Cardiovascular module, heart (LV – left ventricle, RV – right ventricle)**			
033	$\frac{dV_{HL}}{dt} = F_{VRHL} - F_{HLAL}$	Change in blood volume in the LV (V_{HL}) due to the difference between incoming (F_{VRHL}) and outgoing (F_{HLAL}) blood flows.	–
034	$V_{HL}(0) = 0.03 \cdot V$	Starting value of blood volume in the LV is 3% of the total blood volume V.	–
035	$\omega_{HL}(0) = k_{HL} \cdot V_{HL}$	Starting value of the unstressed LV volume is a fraction k_{HL} of V_{HL}.	–
036	$\frac{dV_{HR}}{dt} = F_{VLHR} - F_{HRAR}$	Change in blood volume in the RV (V_{HR}) due to the difference between incoming (F_{VLHR}) and outgoing (F_{HRAR}) blood flows.	–
037	$V_{HR}(0) = 0.03 \cdot V$	Starting value of blood volume in the RV is 3% of the total blood volume V.	–
038	$\omega_{HR}(0) = k_{HR} \cdot V_{HR}$	Starting value of the unstressed RV volume is a fraction k_{HR} of V_{HR}.	–

039	$K_L = \left(K_{L0} + 0.25 \cdot nH \cdot sigm\left(20 \cdot \left(K_{L0} - 0.4\right)\right) - -0.25 \cdot nH \cdot sigm\left(20 \cdot \left(K_{L0} - 0.7\right)\right)\right) \cdot \left(1 - B_{blocker\_in}\right)$	K_L – inotropic factor of the LV, K_{L0} – inotropic status of the LV, nH – sympathetic inotropic sensitivity of the myocardium, $B_{blocker\_in}$ – inotropic effect of the β-blocker bisoprolol.	The negative inotropic effect of bisoprolol (Bazroon and Alrashidi, 2022) was added.
040	$K_R = \left(K_{R0} + 0.25 \cdot nH \cdot sigm\left(20 \cdot \left(K_{R0} - 0.4\right)\right) - -0.25 \cdot nH \cdot sigm\left(20 \cdot \left(K_{R0} - 0.7\right)\right)\right) \cdot \left(1 - B_{blocker\_in}\right)$	K_R – inotropic factor of the RV, K_{R0} – inotropic status of the RV, nH – sympathetic inotropic sensitivity of the myocardium, $B_{blocker\_in}$ – inotropic effect of the β-blocker bisoprolol.	The negative inotropic effect of bisoprolol (Bazroon and Alrashidi, 2022) was added.
041	If: $Cycle_{Time} \geq Cycle_{Length}$<br>Then:<br>1. $Cycle_{Length} = 1/H$<br>2. $Cycle_{Time} = 0$<br>3. $V_{HL\_KD} = V_{HL}$<br>4. $V_{HR\_KD} = V_{HR}$<br>5. $SV = K_L \cdot SV_{\max} \cdot \left[sigm\left(0.03 \cdot \left(V_{HL} - FS_{threshold} - 80\right)\right) - -sigm\left(0.03 \cdot \left(V_{HL} - FS_{threshold} - 260\right)\right) \right]$<br>6. $V_{HL\_KS} = V_{HL\_KD} - SV$<br>7. $V_{HR\_KS} = V_{HR} - K_R \cdot SV_{\max} \cdot \left[sigm\left(0.03 \cdot \left(V_{HR} - -FS_{threshold} - 80\right)\right) - sigm\left(0.03 \cdot \left(V_{HR} - FS_{threshold} - 260\right)\right) \right]$<br>8. $Systole_{Length\_L\_Exp} = \frac{0.25}{H} + 0.2 \cdot \left(1 - K_L\right)$<br>9. $Systole_{Length\_R\_Exp} = \frac{0.25}{H} + 0.2 \cdot \left(1 - K_R\right)$<br>10. $Systole_{Length\_L} = \frac{0.25}{H} + 0.2 \cdot \left(1 - K_L\right)$<br>11. $Systole_{Length\_R} = \frac{0.25}{H} + 0.2 \cdot \left(1 - K_R\right)$<br>12. $Systole_L = 1$<br>13. $Systole_R = 1$<br>14. $P_D = P_{AL}$<br>15. $P_{AR\_D} = P_{AR}$<br>16. $P_{HL\_KD} = P_{HL\_D}$<br>17. $P_{HR\_KD} = P_{HR\_D}$	Transition "diastole – systole". A discrete event defined by an instantaneous change in the model parameters at the beginning of the cardiac cycle. The event is triggered when the current cycle time $Cycle_{Time}$ reaches the cycle length $Cycle_{Length}$.<br><br>H – neurohumoral factor, $SV_{\max}$ – theoretical maximum stroke volume, $FS_{threshold}$ – the Frank-Starling law threshold, P_{AL} – systemic arterial pressure, P_D – diastolic blood pressure, P_{AR} – pulmonary arterial pressure, P_{AR_D} – diastolic pulmonary arterial pressure.<br><br>V_{HL} – current LV volume, V_{HL_KD} – LV end-diastolic volume, SV – LV stroke volume, V_{HL_KS} – LV end-systolic volume, K_L – inotropic factor of the LV, $Systole_{Length\_L\_Exp}$ – nominal (expected) duration of the LV systole, $Systole_{Length\_L}$ – actual duration of the LV systole, $Systole_L$ – indicator of the actual LV systole, P_{HL_KD} – LV end-diastolic pressure, P_{HL_D} – LV diastolic pressure.<br><br>V_{HR} – current RV volume, V_{HR_KD} – RV end-diastolic volume, V_{HR_KS} – RV end-systolic volume, K_R – inotropic factor of the RV, $Systole_{Length\_R\_Exp}$ – nominal (expected) duration of the RV systole, $Systole_{Length\_R}$ – actual duration of the RV systole, $Systole_R$ – indicator of the actual RV systole, P_{HR_KD} – RV end-diastolic pressure, P_{HR_D} – RV diastolic pressure.	Equations fixing the values of P_{HL_KD} and P_{HR_KD} were added.

042	$FS_{threshold}(0) = mass_{volume}(FS_{threshold\,0}, m)$	Starting value of the Frank-Starling law threshold, $FS_{threshold\,0}$ – the average normal value of the threshold, *m* – body mass.	–
043	$SV_{\max}(0) = mass_{volume}(SV_{\max 0}, m)$	Starting value of the theoretical maximum stroke volume, $SV_{\max 0}$ – the average value of $SV_{\max}$, *m* – body mass.	–
044	If: $Systole = 1$<br>Then:<br>1. $F_{HLAL\_p} = F_{HLAL}$<br>2. $F_{HRAR\_p} = F_{HRAR}$	A discrete event is triggered after the transition "diastole – systole" (*Systole* = 1). At this point, blood flow through the aortic valve F_{HLAL} reaches its peak F_{HLAL_p}, and blood flow through the pulmonary valve F_{HRAR} reaches its peak F_{HRAR_p}.	–
045	If: $V_{HL} < V_{HL\_KS}$ and $Systole_L = 1$<br>Then:<br>1. $Systole_L = 0$<br>2. $Systole_{Length\_L} = Cycle_{Time}$<br>3. $P_S = P_{AL}$	Transition "LV systole – LV diastole". A discrete event is triggered if LV volume V_{HL} reaches LV end-systolic volume V_{HL_KS} and the LV is in systole. At this point, the LV switches to diastole ($Systole_L = 0$), and the actual duration of the LV systole $Systole_{Length\_L}$ and systolic blood pressure P_S are determined as the current values of cycle time $Cycle_{Time}$ and systemic arterial pressure P_{AL}, respectively.	–
046	If: $V_{HR} < V_{HR\_KS}$ and $Systole_R = 1$<br>Then:<br>1. $Systole_R = 0$<br>2. $Systole_{Length\_R} = Cycle_{Time}$<br>3. $P_{AR\_S} = P_{AR}$	Transition "RV systole – RV diastole". A discrete event is triggered if RV volume V_{HR} reaches RV end-systolic volume V_{HR_KS} and the RV is in systole. At this point, the RV switches to diastole ($Systole_R = 0$), and the actual duration of the RV systole $Systole_{Length\_R}$ and systolic pulmonary arterial pressure P_{AR_S} are determined as the current values of cycle time $Cycle_{Time}$ and pulmonary arterial pressure P_{AR}, respectively.	–
047	If: $P_{VR} > P_{HL}$<br>Then: $F_{VRHL\_ep} = F_{VRHL}$	Opening of the mitral valve. A discrete event that occurs as the LV enters diastole when pulmonary venous pressure P_{VR} becomes greater than LV pressure P_{HL}. In healthy people, the LV filling rate F_{VRHL} reaches its maximum value (early peak) F_{VRHL_ep} immediately after the valve opens.	–
048	If: $P_{VL} > P_{HR}$<br>Then: $F_{VLHR\_ep} = F_{VLHR}$	Opening of the tricuspid valve. A discrete event that occurs as the RV enters diastole when pressure in the systemic veins P_{VL} becomes greater than pressure in the RV P_{HR}. F_{VLHR_ep} – early peak of the RV filling.	–
049	$P_{HL\_D} = mass_{elasticity}(G_{HL}, m) \cdot (V_{HL} - \omega_{HL})$	P_{HL_D} – LV diastolic pressure, V_{HL} – LV volume, ω_{HL} – unstressed LV volume, G_{HL} – LV wall elasticity, *m* – body mass.	–
050	$P_{HR\_D} = mass_{elasticity}(G_{HR}, m) \cdot (V_{HR} - \omega_{HR})$	P_{HR_D} – RV diastolic pressure, V_{HR} – RV volume, ω_{HR} – unstressed RV volume, G_{HR} – RV wall elasticity, *m* – body mass.	–
051	$Systole_{L\_Exp} = \begin{cases} 0, & Cycle_{Time} \geq Systole_{Length\_L\_Exp} \\ 1, & otherwise \end{cases}$	Nominal LV systole indicator.	–

052	$Systole_{R\_Exp} = \begin{cases} 0, & Cycle_{Time} \geq Systole_{Length\_R\_Exp} \\ 1, & otherwise \end{cases}$	Nominal RV systole indicator.	–
053	$Systole = \begin{cases} 0, & Systole_L = 0 \text{ and } Systole_R = 0 \\ 1, & otherwise \end{cases}$	*Systole* is an indicator of the total actual systole. It is equal to 0 if the actual LV and RV systole indicators are simultaneously equal to 0, and 1, otherwise.	–
054	$DTS_L = Systole_L - Systole_{L\_Exp}$	LV systolic mismatch.	–
055	$DTS_R = Systole_R - Systole_{R\_Exp}$	RV systolic mismatch.	–
056	$\frac{dP_{HL\_S}}{dt} = A_5 \cdot DTS_L$	P_{HL_S} – LV systolic pressure, DTS_L – LV systolic mismatch, A_5 – sensitivity factor.	–
057	$\frac{dP_{HR\_S}}{dt} = A_{15} \cdot DTS_R$	P_{HR_S} – RV systolic pressure, DTS_R – RV systolic mismatch, A_{15} – sensitivity factor.	–
058	$P_{HL} = (Systole - Systole_L) \cdot P_{AL} + (1 - Systole) \cdot P_{HL\_D} + P_{HL\_S} \cdot Systole_L$	LV pressure P_{HL} is equal to LV systolic pressure P_{HL_S} if the LV is in systole; systemic arterial pressure P_{AL} if the LV is in diastole, and the RV is in systole; and LV diastolic pressure P_{HL_D} if both the LV and RV are in diastole.	–
059	$P_{HR} = Systole_R \cdot P_{HR\_S} + P_{AR} \cdot (Systole - Systole_R) + (1 - Systole) \cdot P_{HR\_D}$	RV pressure P_{HR} is equal to RV systolic pressure P_{HR_S} if the RV is in systole; pulmonary arterial pressure P_{AR} if the RV is in diastole, and the LV is in systole; and RV diastolic pressure P_{HR_D} if both the RV and LV are in diastole.	–
060	$Y_{VRHL} = mass_{conductivity}(Y_{VRHL0} + A_{16} \cdot P_{VR}, m)$	Y_{VRHL} – conductivity of the mitral valve and pulmonary veins, Y_{VRHL0} – basic conductivity, P_{VR} – pressure in the pulmonary vein and left atrium. A_{16} – constant, *m* – body mass.	–
061	$Y_{VLHR} = mass_{conductivity}(Y_{VLHR0} + P_{VL} \cdot A_6 + RO_{20} \cdot A_7 - P_{HR\_S} \cdot A_{10}, m)$	Y_{VLHR} – conductivity of the tricuspid valve and systemic veins, Y_{VLHR0} – basic conductivity, P_{VL} – pressure in the inferior vena cava and right atrium, RO_{20} – the average normal value of oxygen demand, P_{HR_S} – systolic pressure in the RV. A_6, A_7, A_{10} – constants. *m* – body mass.	–
062	$F_{VRHL} = valve(P_{VR}, P_{HL}, K_{VRHL}, Y_{VRHL})$	Blood flow through the mitral valve F_{VRHL} is described by the difference between pressure in the pulmonary vein and left atrium P_{VR} and LV pressure P_{HL}. Y_{VRHL} – conductivity of the mitral valve and pulmonary veins. K_{VRHL} – regurgitation coefficient.	–
063	$F_{HLAL} = valve(P_{HL}, P_{AL}, K_{HLAL}, mass_{conductivity}(Y_{HLAL}, m))$	Blood flow through the aortic valve F_{HLAL} is characterized by the difference between LV pressure P_{HL} and systemic arterial pressure P_{AL}. Y_{HLAL} – conductivity of the aortic valve and systemic arteries, allometrically scaled by body mass *m*. K_{HLAL} – regurgitation coefficient.	–

064	$F_{VLHR}=valve\left(P_{VL},P_{HR},K_{VLHR},Y_{VLHR}\right)$	Blood flow through the tricuspid valve F_{VLHR} is described by the difference between pressure in the inferior vena cava and right atrium P_{VL} and RV pressure P_{HR}. Y_{VLHR} – conductivity of the tricuspid valve and systemic veins. K_{VLHR} – regurgitation coefficient.	–
065	$F_{HRAR}=valve\left(P_{HR},P_{AR},K_{HRAR},\ mass_{conductivity}\left(Y_{HRAR},m\right)\right)$	Blood flow through the pulmonary valve F_{HRAR} is characterized by the difference between RV pressure P_{HR} and pulmonary arterial pressure P_{AR}. Y_{HRAR} – conductivity of the pulmonary valve and pulmonary arteries, allometrically scaled by body mass *m*. K_{HRAR} – regurgitation coefficient.	–
066	$LA_{PULSE}=atrium_{pulse}\left(Cycle_{Length},Systole_{Length\_L},P_{AL},Cycle_{Time}\right)$	Left atrium pulse wave depends on the cardiac cycle length $Cycle_{Length}$, the actual duration of the LV systole $Systole_{Length\_L}$, systemic arterial pressure P_{AL}, and the current cycle time $Cycle_{Time}$.	–
067	$RA_{PULSE}=atrium_{pulse}\left(Cycle_{Length},Systole_{Length\_R},P_{AL},Cycle_{Time}\right)$	Right atrium pulse wave depends on the cardiac cycle length $Cycle_{Length}$, the actual duration of the RV systole $Systole_{Length\_R}$, pulmonary arterial pressure P_{AL} and the current cycle time $Cycle_{Time}$.	–
068	If: $Cycle_{Time}>Cycle_{Length}-0.2\cdot\left(Cycle_{Length}-Systole_{Length\_L}\right)$<br>Then: $F_{VRHL\_ap}=F_{VRHL}$	A discrete event that determines the active peak F_{VRHL_ap} of blood flow through the mitral valve F_{VRHL}. This peak is reached by contraction of the left atrium at the time when LA_{PULSE} becomes positive.	–
069	If: $Cycle_{Time}>Cycle_{Length}-0.2\cdot\left(Cycle_{Length}-Systole_{Length\_R}\right)$<br>Then: $F_{VLHR\_ap}=F_{VLHR}$	A discrete event that defines the active peak F_{VLHR_ap} of blood flow through the tricuspid valve F_{VLHR}. This peak is reached by contraction of the right atrium at the time when RA_{PULSE} becomes positive.	–
070	$V_{HL\_KS}\left(0\right)=V_{HL\_KD}-K_L\cdot SV_{\max}\cdot\Big[sigm\Big(0.03\cdot\big(V_{HL\_KD}-$<br>$-FS_{threshold}-80\big)\Big)-sigm\Big(0.03\cdot\big(V_{HL\_KD}-FS_{threshold}-260\big)\Big)\Big];$<br>$Systole_{Length\_L\_Exp}\left(0\right)=0.25\cdot Cycle_{Length}+0.2\cdot\left(1-K_L\right);$<br>$Systole_{Length\_L}\left(0\right)=0.25\cdot Cycle_{Length}+0.2\cdot\left(1-K_L\right);$<br>$V_{HR\_KS}\left(0\right)=V_{HR\_KD}-K_R\cdot SV_{\max}\cdot\Big[sigm\Big(0.03\cdot\big(V_{HR\_KD}-$<br>$-FS_{threshold}-80\big)\Big)-sigm\Big(0.03\cdot\big(V_{HR\_KD}-FS_{threshold}-260\big)\Big)\Big];$<br>$Systole_{Length\_R\_Exp}\left(0\right)=0.25\cdot Cycle_{Length}+0.2\cdot\left(1-K_R\right);$<br>$Systole_{Length\_R}\left(0\right)=0.25\cdot Cycle_{Length}+0.2\cdot\left(1-K_R\right).$	Initialization of starting values. See Equations 041 for explanation.	–
071	$\frac{dCycle_{Time}}{dt}=1$	Linking the current time of the cardiac cycle $Cycle_{Time}$ to the model time.	–

072	$Heart_{Rate} = \frac{60}{Cycle_{Length}}$	The duration of the cardiac cycle $Cycle_{Length}$, by definition, is the time of one heartbeat. Heart rate $Heart_{Rate}$ is the number of heart beats per minute.	–
073	$CO = \frac{SV \cdot Heart_{Rate}}{1000}$	Cardiac output CO (L/min) is equal to stroke volume SV (mL) times heart rate $Heart_{Rate}$ (bpm).	–
074	$EF = \frac{SV}{V_{HL\_KD}} \cdot 100$	Ejection fraction EF is stroke volume SV divided by the LV end-diastolic volume V_{HL_KD} and multiplied by 100%.	–
**Cardiovascular module, tissue metabolism**			
075	$gO_2 = F_{ALVL} \cdot \min\left(AO_2 - VO_2, 1\right)$	Oxygen consumption gO_2 in tissues is equal to the product of blood flow through the tissues F_{ALVL} and the arteriovenous oxygen difference $AO_2 - VO_2$.	–
076	$AO_2 = \frac{He \cdot C_H \cdot SpO_2}{1000}$	Arterial oxygen content AO_2 is calculated as the product of the total amount of hemoglobin He (g/L), the oxygen capacity of hemoglobin C_H (mg/mL), and the arterial oxygen saturation SpO_2.	–
077	$\frac{dDO_2}{dt} = \operatorname{sgn}\left(DO_2, A_2 \cdot \left(RO_2 - gO_2\right)\right)$	The rate of change in oxygen debt DO_2 is proportional to the difference between oxygen demand RO_2 and oxygen consumption gO_2. A_2 is the functional status of the body.	–
078	$\frac{dVO_2}{dt} = A_1 \cdot \left(gO_2 - RO_2\right)$	The rate of change in venous oxygen content VO_2 is proportional to the difference between oxygen consumption gO_2 and oxygen demand RO_2. A_1 – total metabolic intensity.	–
079	$RO_2\left(0\right) = mass_{volume}\left(RO_{20}, m\right)$	Starting value of the oxygen demand, RO_{20} – the average normal value of RO_2, m – body mass.	–
**Cardiovascular module, neurohumoral control**			
080	$nB = \min\left(r_{minus}\left(0.07, 30, P_{AL}, 50 \cdot \psi_{AT1\_Baro}\right), 1\right)$	nB – baroreceptor activity, P_{AL} – systemic arterial pressure. $P_0 = 50$ mmHg is the baroreceptor sensitivity threshold, i.e., the lower bound for P_{AL} at which nB = 0. ψ_{AT1_Baro} determines the effect of AT1-bound angiotensin II on blood pressure without affecting heart rate (by changing the P_0 threshold).	–
081	$\psi_{AT1\_Baro} = sl_{baro} \cdot \frac{AT1\_ANGII}{AT1\_ANGII_{norm}} + 1 - sl_{baro}$	Linear function with a slope $0 < sl_{baro} < 1$, which defines the influence of the normalized level of AT1-bound angiotensin II $\left(AT1\_ANGII / AT1\_ANGII_{norm}\right)$ on the baroreceptor sensitivity threshold.	–
082	$nS = \min\left(r_{plus}\left(10, 50, st \cdot \psi_{AT1\_Stress}, 0\right), 1\right)$	nS – stress receptor activity. Parameter st is a stress factor that describes the level of steroid hormones in the blood (adrenaline, norepinephrine) and taking nominal values from 0 (absolute rest) to 1 (absolute stress). ψ_{AT1_Stress} determines the effect of AT1-bound angiotensin II on the norepinephrine release from the atria.	–

083	$\psi_{AT1\_Stress} = sl_{stress} \cdot \frac{AT1\_ANGII}{AT1\_ANGII_{norm}} + 1 - sl_{stress}$	Linear function with a slope $0 < sl_{stress} < 1$, which defines the influence of the normalized level of AT1-bound angiotensin II $(AT1\_ANGII / AT1\_ANGII_{norm})$ on the stress factor.	–
084	$nV = \min\left(r_{minus}\left(30, 30, VO_2, 0\right), 1\right)$	*nV* – respiratory receptor activity, VO_2 – venous oxygen content.	–
085	$nD = \min\left(r_{plus}\left(0.1, 10, DO_2, 0\right), 1\right)$	*nD* – fatigue receptor activity, DO_2 – oxygen debt.	–
086	$nSum = Heart_{Stress} \cdot nS \cdot \left(1 - B_{blocker}\right) \cdot \left(1 - Diuretic_{stress}\right) + + Heart_{Baro} \cdot nB + Heart_{Oxygen} \cdot nD + Heart_{VO2} \cdot nV + Heart_{Base}$	The activity of the cardiac center (*nSum*) is the sum of the activities of stress (*nS*), fatigue (*nD*), and respiratory receptors (*nV*), as well as baroreceptors (*nB*). $Heart_{Stress}$ – stress sensitivity, $Heart_{Baro}$ – baroreceptor sensitivity, $Heart_{Oxygen}$ – sensitivity to fatigue (a lower value corresponds to the greater exercise tolerance), $Heart_{VO2}$ – respiratory sensitivity, $Heart_{Base}$ – basic activity of the cardiac center, $B_{blocker}$ – negative chronotropic effect of the β-blocker bisoprolol, $Diuretic_{stress}$ – effect of the thiazide diuretic hydrochlorothiazide (HCTZ) on the pressor response of norepinephrine.	The effects of bisoprolol and HCTZ on stress receptor activity were taken from the model extension (Kutumova et al., 2022).
087	$nH = \begin{cases} 0, & H < 0 \\ \frac{1 - \exp(-3H)}{1 + 100 \cdot \exp(-3H)}, & otherwise \end{cases}$	Sigmoid function of sympathetic inotropic sensitivity of the myocardium depending on the neurohumoral factor *H*.	–
088	$\frac{dH}{dt} = A_{12} \cdot \left(3.2 \cdot \min\left(r_{plus}\left(2, 18, nSum, 0\right), 1\right) - H\right)$	Calculation of the neurohumoral factor *H*, considering the reactivity of the cardiac center A_{12}, which characterizes its adaptive ability.	–
**Renal module, nervous system**			
089	$RSNA = N_{rsna} \cdot \alpha_{map} \cdot \alpha_{rap}$	*RSNA* – renal sympathetic nerve activity, N_{rsna} – normalized *RSNA* value. α_{map} and α_{rap} represent the effects of mean arterial pressure and right atrial pressure on *RSNA*, respectively.	–
090	$\alpha_{map} = 0.5 + 1.1 \cdot \left(1 + \exp\left(\frac{MAP - 100}{15}\right)\right)^{-1}$	Effect of mean arterial pressure *MAP* on *RSNA*.	–
091	$\alpha_{rap} = 1 - 0.008 \cdot P_{ra}$	Effect of right atrial pressure P_{ra} on *RSNA*.	–
092	$P_{ra} = 0.2787 \cdot \exp\left(0.2281 \cdot CO\right)$	Normalized right atrial pressure P_{ra} is calculated using the Frank-Starling law as a function of cardiac output *CO*.	–
**Renal module, sodium transport and reabsorption along the nephron**			
093	$\Phi_{filsod} = GFR \cdot C_{sod}$	Φ_{filsod} – amount of sodium filtered from the glomerulus to the proximal tubule per minute (filtered sodium load), *GFR* – glomerular filtration rate, C_{sod} – serum sodium concentration.	–
094	$\eta_{pt\_sodreab} = \min\left(1, n_{\eta\_pt} \cdot \gamma_{filsod} \cdot \gamma_{at} \cdot \gamma_{rsna}\right)$	Fractional proximal sodium reabsorption is defined as the normalized value n_{η_pt} multiplied by the influence functions described below. Cannot exceed 100%.	–

095	$\gamma_{at} = 0.95 + \frac{0.12}{1 + \exp\left(2.6 - 1.8 \cdot \log AT1\_ANGII\right)}$	An increase in the level of AT1-bound angiotensin II (*AT1_ANGII*) leads to an increase in fractional proximal sodium reabsorption.	–
096	$\gamma_{filsod} = 0.8 + \frac{0.3}{1 + 138^{-1} \cdot \exp\left(\Phi_{filsod} - 14\right)}$	An increase in the filtered sodium load Φ_{filsod} leads to a decrease in fractional proximal sodium reabsorption.	–
097	$\gamma_{rsna} = 0.5 + \frac{0.7}{1 + 2.18^{-1} \cdot \exp\left(1 - RSNA\right)}$	Increased renal sympathetic nerve activity *RSNA* results in increased fractional proximal sodium reabsorption.	–
098	$\Phi_{pt\_sodreab} = \Phi_{filsod} \cdot \eta_{pt\_sodreab}$	$\Phi_{pt\_sodreab}$ – absolute proximal sodium reabsorption rate, Φ_{filsod} – filtered sodium load, $\eta_{pt\_sodreab}$ – fractional proximal sodium reabsorption.	–
099	$\Phi_{md\_sod} = \Phi_{filsod} - \Phi_{pt\_sodreab}$	Φ_{md_sod} – macula densa sodium flow rate, Φ_{filsod} – filtered sodium load, $\Phi_{pt\_sodreab}$ – absolute proximal sodium reabsorption rate.	–
100	$\eta_{dt\_sodreab} = \min\left(1, n_{\varepsilon\_dt} \cdot \psi_{al} \cdot \left(1 - Diuretic_{Inhibition}\right)\right)$	Fractional distal sodium reabsorption is defined as the normalized value n_{ε_dt} multiplied by the aldosterone influence function ψ_{al}. Cannot exceed 100%. $Diuretic_{Inhibition}$ – inhibition of distal tubular sodium reabsorption by the thiazide diuretic hydrochlorothiazide (HCTZ).	The effect of HCTZ on fractional distal sodium reabsorption was taken from the model extension (Kutumova et al., 2022).
101	$\Psi_{al} = 0.17 + 0.94 \cdot \left(1 + \exp\left(\frac{0.48 - 1.2 \cdot \log C_{al}}{0.88}\right)\right)^{-1}$	Effect of aldosterone concentration C_{al} on fractional distal sodium reabsorption.	–
102	$\Phi_{dt\_sodreab} = \Phi_{md\_sod} \cdot \eta_{dt\_sodreab}$	$\Phi_{dt\_sodreab}$ – absolute distal sodium reabsorption rate, Φ_{md_sod} – macula densa sodium flow rate, $\eta_{dt\_sodreab}$ – fractional distal sodium reabsorption.	–
103	$\Phi_{dt\_sod} = \Phi_{md\_sod} - \Phi_{dt\_sodreab}$	Φ_{dt_sod} – rate of the distal sodium outflow, Φ_{md_sod} – macula densa sodium flow rate, $\Phi_{dt\_sodreab}$ – absolute distal sodium reabsorption rate.	–
104	$\eta_{cd\_sodreab} = \min\left(1, n_{\eta\_cd} \cdot \lambda_{dt} \cdot \lambda_{anp}\right)$	Fractional collecting duct sodium reabsorption is defined as the normalized value n_{η_cd} multiplied by the influence functions described below. Cannot exceed 100%.	–
105	$\lambda_{dt} = 0.82 + \frac{0.39}{1 + \exp\left(0.5 \cdot \left(\Phi_{dt\_sod} - 1.6\right)\right)}$	Effect of distal sodium outflow rate Φ_{dt_sod} on fractional collecting duct sodium reabsorption.	–
106	$\lambda_{anp} = -\ 0.1 \cdot \frac{C_{anp}}{C_{anp\_norm}} + 1.1199$	Effect of normalized natriuretic peptide concentration C_{anp}/C_{anp_norm} on fractional collecting duct sodium reabsorption.	–
107	$\Phi_{cd\_sodreab} = \Phi_{dt\_sod} \cdot \eta_{cd\_sodreab}$	$\Phi_{cd\_sodreab}$ – absolute collecting duct sodium reabsorption rate, Φ_{dt_sod} – rate of the distal sodium outflow, $\eta_{cd\_sodreab}$ – fractional collecting duct sodium reabsorption.	–

108	$\Phi_{u\_sod} = \Phi_{dt\_sod} - \Phi_{cd\_sodreab}$	Φ_{u_sod} – urine sodium excretion rate, Φ_{dt_sod} – rate of the distal sodium outflow, $\Phi_{cd\_sodreab}$ – absolute collecting duct sodium reabsorption rate.	–
109	$\frac{dM_{sod}}{dt} = \Phi_{sodin} - \Phi_{u\_sod}$	The rate of formation of total exchangeable sodium (M_{sod}) is determined by the difference between the rate of sodium intake Φ_{sodin} and the rate of sodium excretion Φ_{u_sod}.	–
110	$C_{sod} = 1.03 \cdot \frac{(M_{sod} - 410)}{TBW} - 0.29 \cdot glucose + 78.44$	Serum sodium concentration C_{sod} as a function of total exchangeable sodium M_{sod}, total body water *TBW*, and plasma glucose (*glucose*).	–
**Renal module, renin-angiotensin-aldosterone system**			
111	$R_{sec} = \frac{\ln 2}{h_{renin}} \cdot PRC_{nom} \cdot \nu_{MD\_sod} \cdot \nu_{RSNA} \cdot \nu_{AT1\_ANGII} \cdot$ $\cdot (1 + Diuretic_{Stimulation}) \cdot (1 - B_{blocker\_rs})$	Renin secretion rate R_{sec} is defined by the nominal value of plasma renin concentration (PRC) PRC_{nom} multiplied by the influence functions described below. h_{renin} – half-life of PRC, $Diuretic_{Stimulation}$ – stimulation of renin secretion by the thiazide diuretic hydrochlorothiazide (HCTZ), $B_{blocker\_rs}$ – suppression of renin secretion by the β-blocker bisoprolol.	The effects of HCTZ and bisoprolol on renin secretion rate were taken from the model extension (Kutumova et al., 2022).
112	$\nu_{MD\_sod} = exp\left(-\tau_{MD\_renin} \cdot \left(\Phi_{md\_sod} - \Phi_{md\_sod\_0}\right)\right)$	Effect of macula densa sodium flow rate Φ_{md_sod} on the rate of renin secretion. $\Phi_{md\_sod\_0}$ – nominal value of Φ_{md_sod}, τ_{MD_renin} – constant.	–
113	$\nu_{RSNA} = 1.89 - \frac{2.056}{1.358 + \exp(RSNA - 0.8667)}$	Effect of renal sympathetic nerve activity *RSNA* on the rate of renin secretion.	–
114	$\nu_{AT1\_ANGII} = \left(\frac{AT1\_ANGII_{norm}}{AT1\_ANGII}\right)^{slope_{AT1\_PRC}}$	Effect of the normalized value of AT1-bound angiotensin II ($AT1\_ANGII_{norm} / AT1\_ANGII$) on the rate of renin secretion. $slope_{AT1\_PRC}$ – constant.	–
115	$\frac{dPRC}{dt} = R_{sec} - \frac{\ln 2}{h_{renin}} \cdot PRC$	Plasma renin concentration (*PRC*) is described by the rate of renin secretion R_{sec} and the rate of renin clearance, with a half-life of h_{renin}.	–
116	$PRC(0) = PRC_{nom}$	Starting value of *PRC* value is the nominal value PRC_{nom}.	–
117	$PRA = PRC \cdot X_{PRC\_PRA} \cdot (1 - DRI)$	*PRA* – plasma renin activity, *PRC* – plasma renin concentration, X_{PRC_PRA} – equilibrium ratio of *PRA* to *PRC*, *DRI* – direct renin inhibition by aliskiren.	The effect of aliskiren on *PRC* was taken from the model extension (Kutumova et al., 2022).
118	$\frac{dANGI}{dt} = PRA - (c_{ACE} \cdot (1 - ACEi) + c_{chym} + c_{nep}) \cdot ANGI -$ $- \frac{\ln 2}{h_{ANGI}} \cdot ANGI$	*ANGI* – plasma angiotensin I, *PRA* – plasma renin activity, c_{ACE} and c_{chym} – rates of conversion of *ANGI* to angiotensin II by ACE and chymase, c_{nep} – rate of conversion of *ANTI* to angiotensin-(1-7) by neprilisin, h_{ANGI} – half-life of *ANGI*, *ACEi* – inhibition of angiotensin-converting enzyme by enalapril.	The effect of enalapril on c_{ACE} was taken from the model extension (Kutumova et al., 2022).

119	$$\frac{dANGII}{dt}=\left(c_{ACE}\cdot(1-ACEi)+c_{chym}\right)\cdot ANGI-\left(c_{ACE2}+c_{ANGII\_ANGIV}+c_{AT1}\cdot(1-ARB)+c_{AT2}\right)\cdot ANGII-\frac{\ln 2}{h_{ANGII}}\cdot ANGII$$	*ANGII* – plasma angiotensin II, *ANGI* – plasma angiotensin I, c_{ACE} and c_{chym} – rates of conversion of *ANGI* to *ANGII* by ACE and chymase, c_{ACE2} – rate of conversion of *ANGII* to angiotensin-(1-7) by ACE2, c_{ANGII_ANGIV} – rate of conversion of *ANGII* to angiotensin IV, c_{AT1} and c_{AT2} – rates of *ANGII* binding to AT1 and AT2 receptors, h_{ANGII} – half-life of *ANGII*, $ACEi$ – inhibition of angiotensin-converting enzyme by enalapril, *ARB* – blocking angiotensin receptors with losartan.	The effects of enalapril on c_{ACE} and losartan on c_{AT1} were taken from the model extension (Kutumova et al., 2022).
120	$$\frac{dANG17}{dt}=c_{nep}\cdot ANGI+c_{ACE2}\cdot ANGII-\frac{\ln 2}{h_{ANG17}}\cdot ANG17$$	*ANG*17 – plasma angiotensin-(1-7), *ANGI* – plasma angiotensin I, *ANGII* – plasma angiotensin II, c_{nep} – rate of conversion of *ANTI* to *ANG*17 by neprilisin, c_{ACE2} – rate of conversion of *ANGII* to *ANG*17 by ACE2, h_{ANG17} – half-life of *ANG*17.	–
121	$$\frac{dANGIV}{dt}=c_{ANGII\_ANGIV}\cdot ANGII-\frac{\ln 2}{h_{ANGIV}}\cdot ANGIV$$	*ANGIV* – plasma angiotensin IV, *ANGII* – plasma angiotensin II, c_{ANGII_ANGIV} – rate of conversion of *ANGII* to *ANGIV*, h_{ANGIV} – half-life of *ANGIV*.	–
122	$$\frac{dAT1\_ANGII}{dt}=c_{AT1}\cdot(1-ARB)\cdot ANGII-\frac{\ln 2}{h_{AT1}}\cdot AT1\_ANGII$$	*AT1_ANGII* – concentration of AT1-bound angiotensin II, *ANGII* – plasma angiotensin II, c_{AT1} – rate of *ANGII* binding to AT1 receptors, h_{AT1} – half-life of *AT1_ANGII*, *ARB* – blocking angiotensin receptors with losartan.	The effect of losartan on c_{AT1} was taken from the model extension (Kutumova et al., 2022)
123	$$\frac{dAT2\_ANGII}{dt}=c_{AT2}\cdot ANGII-\frac{\ln 2}{h_{AT2}}\cdot AT2\_ANGII$$	*AT2_ANGII* – concentration of AT2-bound angiotensin II, *ANGII* – plasma angiotensin II, c_{AT2} – rate of *ANGII* binding to AT2 receptors, h_{AT2} – half-life of *AT2_ANGII*.	–
124	$N_{als}=\xi_{k\_sod}\cdot\xi_{map}\cdot\xi_{at}$	The normalized aldosterone secretion rate N_{als} is calculated as the product of the influence functions described below. ξ_{map} – effect of mean arterial pressure *MAP*. $\xi_{map}=1$ (no effect) if *MAP* is normal or above normal.	–
125	$\xi_{k\_sod}=2^{power},\quad power=C_K\cdot\left(1-Diuretic_{potassium}\right)-4.5$	Effect of potassium concentration (C_K) on the rate of aldosterone secretion. $Diuretic_{potassium}$ – reducing the level of potassium in the blood with the thiazide diuretic hydrochlorothiazide (HCTZ).	The effect of HCTZ on potassium was taken from the model extension (Kutumova et al., 2022).
126	$\xi_{at}=0.4+2.4\cdot\left(1+\exp\left(2.82-1.5\cdot\frac{\log AT1\_ANGII}{0.8}\right)\right)^{-1}$	Effect of AT1-bound angiotensin II (*AT1_ANGII*) on the rate of aldosterone secretion.	–
127	$\frac{dN_{al}}{dt}=\frac{N_{als}-N_{al}}{T_{al}}$	N_{al} – normalized aldosterone concentration, N_{als} – normalized aldosterone secretion rate, T_{al} – time constant.	–
128	$C_{al}=C_{al\_norm}\cdot N_{al}$	Plasma aldosterone C_{al} is equal to the product of the normalized aldosterone concentration N_{al} and its normal value C_{al_norm}.	–

**Renal module, hormonal system**			
129	$C_{anp} = C_{anp\_norm} \cdot \left(7.427 - \dfrac{6.554}{1+\exp\left(P_{ra} - 3.762\right)}\right)$	Natriuretic peptide concentration C_{anp} depending on pressure in the right atrium P_{ra} and the normal hormone level C_{anp_norm}.	–
130	$osmolality = 1.86 \cdot C_{sod} + glucose + urea \cdot \left(1 + Diuretic_{urea}\right) + 9$	*osmolality* – serum osmolality, C_{sod} – serum sodium concentration, *glucose* – plasma glucose, *urea* – plasma urea, $Diuretic_{urea}$ – increasing the level of urea in the blood with the thiazide diuretic hydrochlorothiazide (HCTZ).	The effect of HCTZ on urea was taken from the model extension (Kutumova et al., 2022).
131	$C_{adh} = \max\left(0, 0.23 \cdot \left(osmolality - 271\right)\right)$	Concentration of antidiuretic hormone C_{adh} depending on serum osmolality.	–
**Renal module, diuresis**			
132	$\Phi_{t\_wreab} = \eta_{pt\_sodreab} \cdot GFR + \left(1 - \eta_{pt\_sodreab}\right) \cdot GFR \cdot \mu_{adh}$	Φ_{t_wreab} – tubular water reabsorption rate, GFR – glomerular filtration rate, $\eta_{pt\_sodreab}$ – fractional proximal sodium reabsorption, μ_{adh} – effect of plasma antidiuretic hormone concentration.	–
133	$\mu_{adh} = \begin{cases} 0.0, & C_{adh} \le 0.765 \\ 0.383 \cdot C_{adh} - 0.293, & 0.765 < C_{adh} \le 3 \\ -0.0383 \cdot C_{adh}^2 + 0.364 \cdot C_{adh} + 0.109, & 3 < C_{adh} \le 5 \\ 0.0012 \cdot C_{adh} + 0.9653, & C_{adh} > 5 \end{cases}$	Effect of plasma antidiuretic hormone concentration C_{adh} on the rate of tubular water reabsorption.	–
134	$\Phi_u = GFR - \Phi_{t\_wreab}$	Φ_u – urine flow rate, GFR – glomerular filtration rate, Φ_{t_wreab} – tubular water reabsorption rate.	–
**Renal module, body fluids**			
135	$\Phi_{win} = \Phi_{win\_norm} \cdot \left(0.25 + 1.5 \cdot \left(1 + exp\left(2 - 2 \cdot \dfrac{C_{adh}}{C_{adh\_norm}}\right)\right)^{-1}\right)$	Φ_{win} – rate of water intake, Φ_{win_norm} – nominal value of water consumption rate, C_{adh}/C_{adh_norm} – normalized antidiuretic hormone concentration.	–
136	$\dfrac{dTBW}{dt} = \Phi_{win} - \Phi_u$	*TBW* – total body water, Φ_{win} – rate of water intake, Φ_u – urine flow rate.	–
137	$V = 111.5 \cdot TBW + 650$	Total blood volume *V* as a linear function of total body water *TBW*.	–
138	$V_{ecf} = 0.37 \cdot TBW + 2.7$	Extracellular fluid volume V_{ecf} as a linear function of total body water *TBW*.	–
**Renal module, glomerular filtration**			
139	$R_{aa} = R_{aa\_0} \cdot \beta_{rsna} \cdot \Sigma_{tgf} \cdot \Sigma_{myo} \cdot \Psi_{AT1\_aa} \cdot \left(1 - CCB_{aa}\right) \cdot \left(1 - Diuretic_{aa}\right)$	The resistance of a single afferent arteriole R_{aa} is equal to the nominal value R_{aa_0} multiplied by the influence functions described below. CCB_{aa} – effect of the calcium channel blocker amlodipine, $Diuretic_{aa}$ – effect of the thiazide diuretic hydrochlorothiazide (HCTZ).	The vasodilatory effects of amlodipine and HCTZ were taken from the model extension (Kutumova et al., 2022)

140	$R_{aa\_0} = 1.25E8 \cdot \frac{128 \cdot Vis \cdot L_{aa}}{\pi \cdot d_{aa}^4}$	The nominal value of the resistance of a single afferent arteriole R_{aa_0} can be determined by Poiseuille's law. d_{aa} and L_{aa} – diameter and length of the arteriole, *Vis* – blood viscosity. The coefficient 1.25E8 is used to convert units from cP · μm^{-3} to mmHg · min · L^{-1}.	–
141	$R_{preglom} = R_{preglom\_0} \cdot \beta_{rsna} \cdot \Sigma_{myo} \cdot \psi_{AT1\_preglom} \cdot \cdot (1 - CCB_{preglom}) \cdot (1 - Diuretic_{preglom})$	The resistance of interlobar, arcuate, and interlobular arteries $R_{preglom}$ is equal to the nominal value $R_{preglom\_0}$ multiplied by the influence functions described below. $CCB_{preglom}$ – effect of the calcium channel blocker amlodipine, $Diuretic_{preglom}$ – effect of the thiazide diuretic hydrochlorothiazide (HCTZ).	The vasodilatory effects of amlodipine and HCTZ were taken from the model extension (Kutumova et al., 2022)
142	$\beta_{rsna} = 1.5 \cdot (RSNA - 1) + 1$	Effect of renal sympathetic nerve activity *RSNA* on the resistance of single afferent arterioles (R_{aa}) and the resistance of interlobar, arcuate, and interlobular arteries ($R_{preglom}$).	–
143	$\frac{d\Sigma_{tgf}}{dt} = 0.3408 + 3.449 \cdot \left(3.88 + \exp\left(\frac{\Phi_{md\_sod} - 3.859}{-0.9617}\right)\right)^{-1} - \Sigma_{tgf}$	Σ_{tgf} – tubuloglomerular feedback signal, Φ_{md_sod} – macula densa sodium flow rate.	–
144	$\frac{d\Sigma_{myo}}{dt} = sl_{Pgh} \cdot \frac{P_{gh}}{P_{gh\_norm}} + 1 - sl_{Pgh} - \Sigma_{myo}$	Σ_{myo} – myogenic autoregulation signal, P_{gh}/P_{gh_norm} – the normalized value of glomerular hydrostatic pressure, sl_{Pgh} – constant.	–
145	$\Psi_{AT1\_aa} = A_{AT1\_aa} + B_{AT1\_aa} \cdot AT1\_ANGII - \frac{C_{AT1\_aa}}{AT1\_ANGII}$	Effect of AT1-bound angiotensin II (*AT1_ANGII*) on the resistance of a single afferent arteriole. A_{AT1_aa}, B_{AT1_aa}, and C_{AT1_aa} – constants.	–
146	$\Psi_{AT1\_preglom} = A_{AT1\_preglom} + B_{AT1\_preglom} \cdot AT1\_ANGII - \frac{C_{AT1\_preglom}}{AT1\_ANGII}$	Effect of AT1-bound angiotensin II (*AT1_ANGII*) on the resistance of interlobar, arcuate, and interlobular arteries. $A_{AT1\_preglom}$, $B_{AT1\_preglom}$, and $C_{AT1\_preglom}$ – constants.	–
147	$R_a = \frac{R_{aa}}{N_{nephrons}} + R_{preglom}$	R_a – resistance of afferent vessels, R_{aa} – resistance of a single afferent arteriole, $N_{nephrons}$ – number of nephrons in the kidneys, $R_{preglom}$ – resistance of interlobar, arcuate, and interlobular arteries.	–
148	$R_{a\_dyne} = 79.68 \cdot R_a$	Conversion units of R_a from mmHg · min · L^{-1} to dyn · s · cm^{-5}.	–
149	$R_{ea} = R_{ea\_0} \cdot \Psi_{AT1\_ea} \cdot (1 - CCB_{ea}) \cdot (1 - Diuretic_{ea})$	The resistance of a single efferent arteriole R_{ea} is equal to the nominal value R_{ea_0} multiplied by the effect function of AT1-bound angiotensin II (Ψ_{AT1_ea}). CCB_{ea} – effect of the calcium channel blocker amlodipine, $Diuretic_{ea}$ – effect of the thiazide diuretic hydrochlorothiazide (HCTZ).	The vasodilatory effects of amlodipine and HCTZ were taken from the model extension (Kutumova et al., 2022)
150	$R_{ea\_0} = 1.25E8 \cdot \frac{128 \cdot Vis \cdot L_{ea}}{\pi \cdot d_{ea}^4}$	The nominal value of the resistance of a single efferent arteriole R_{ea_0} can be determined by Poiseuille's law. d_{ea} and L_{ea} – diameter and length of the arteriole, *Vis* – blood viscosity. The coefficient 1.25E8 is used to convert units from cP · μm^{-3} to mmHg · min · L^{-1}.	–

151	$\Psi_{AT1\_ea} = A_{AT1\_ea} + B_{AT1\_ea} \cdot AT1\_ANGII - \dfrac{C_{AT1\_ea}}{AT1\_ANGII}$	Effect of AT1-bound angiotensin II (*AT1_ANGII*) on the resistance of a single efferent arteriole. A_{AT1_ea}, B_{AT1_ea}, and C_{AT1_ea} – constants.	–
152	$R_e = \dfrac{R_{ea}}{N_{nephrons}}$	R_e – resistance of all efferent arterioles, R_{ea} – resistance of a single efferent arteriole, $N_{nephrons}$ – number of nephrons in the kidneys.	–
153	$R_{e\_dyne} = 79.68 \cdot R_e$	Conversion units of R_e from mmHg · min · L^{-1} to dyn · s · cm^{-5}.	–
154	$RVR = R_a + \dfrac{RBF - GFR}{RBF} \cdot R_e + R_v$	*RVR* – renal vascular resistance, R_a – resistance of afferent vessels, R_e – resistance of all efferent arterioles, R_v – renal venous resistance, *GFR* – glomerular filtration rate, *RBF* – renal blood flow.	–
155	$RBF = \dfrac{MAP - P_v + K_{FG} \cdot R_e \cdot (MAP - P_B - P_{go})}{R_a + R_e + R_v + K_{FG} \cdot R_e \cdot R_a}$	*RBF* – renal blood flow, *MAP* – mean arterial pressure, P_v – renal venous pressure, P_B – hydrostatic pressure in Bowman's space, P_{go} – glomerular capillary oncotic pressure, K_{FG} – glomerular filtration coefficient, R_a – resistance of afferent vessels, R_e – resistance of all efferent arterioles, R_v – renal venous resistance.	–
156	$P_{gh} = MAP - RBF \cdot R_a$	Calculation of glomerular hydrostatic pressure P_{gh} using Ohm's law. *MAP* – mean arterial pressure, *RBF* – renal blood flow, R_a – resistance of afferent vessels.	–
157	$RPF = RBF \cdot (1 - 0.01 \cdot Hct)$	*RPF* – renal plasma flow, *RBF* – renal blood flow, *Hct* – hematocrit.	–
158	$FF = \dfrac{GFR}{RPF}$	Filtration fraction *FF* is the ratio of the glomerular filtration rate *GFR* to the renal plasma flow *RPF*.	–
159	$C_M = 0.1 \cdot \dfrac{TP}{FF} \cdot \ln\left(\dfrac{1}{1 - FF}\right)$	C_M – plasma protein mean concentration within the glomerular capillaries, *TP* – total protein, *FF* – filtration fraction.	–
160	$\dfrac{dP_{go}}{dt} = 5 \cdot (C_M - 2) - P_{go}$	P_{go} – glomerular capillary oncotic pressure, C_M – plasma protein mean concentration within the glomerular capillaries.	–
161	$K_{FG} = K_{FG\_0} \cdot \left(sl_{KFG} \cdot \dfrac{AT1\_ANGII_{norm}}{AT1\_ANGII} + 1 - sl_{KFG}\right)$	The glomerular filtration coefficient K_{FG} is calculated as the product of the normal value K_{FG_0} and a linear function (with slope $0 < sl_{KFG} < 1$) expressing the inverse relationship between K_{FG} and the normalized concentration of *AT1_ANGII*. The function is chosen so that the value $AT1\_ANGII = AT1\_ANGII_{norm}$ gives the result $K_{FG} = K_{FG\_0}$.	–
162	$GFR = K_{FG} \cdot (P_{gh} - P_B - P_{go})$	*GFR* – glomerular filtration rate, K_{FG} – glomerular filtration coefficient, P_{gh} – glomerular hydrostatic pressure, P_B – hydrostatic pressure in Bowman's space, P_{go} – glomerular capillary oncotic pressure.	–

Table S3. Model parameters with values for a normotensive person[3]

№	Notation	Description	Initial value	Primary source	Normal range[4]	Units
001	A_1	Total metabolic intensity	0.00076	Fitted	0.00032 – 0.00128 (Kutumova et al., 2021)	mL^{-1}
002	A_{10}	Constant for calculating the conductivity of the tricuspid valve and systemic veins	0.8	(Proshin and Solodyannikov, 2006)	–	$mL \cdot s^{-1} \cdot mmHg^{-2}$
003	A_{11}	Systemic and pulmonary venous tone	0.0325	(Proshin and Solodyannikov, 2006)	–	$mL \cdot mmHg^{-1}$
004	A_{12}	Reactivity of the cardiac center	0.19336	(Proshin and Solodyannikov, 2006)	–	s^{-1}
005	A_{13}	Sympathetic sensitivity of the pulmonary microvessels	0.65	(Proshin and Solodyannikov, 2006)	–	$mL \cdot mmHg^{-1}$
006	A_{14}	Sensitivity of pulmonary microvessels to oxygen debt	0.08265	(Proshin and Solodyannikov, 2006)	–	$s^{-1} \cdot mmHg^{-1}$
007	A_{15}	Sensitivity factor for calculating RV systolic pressure	22.0	(Proshin and Solodyannikov, 2006)	–	$s^{-1} \cdot mmHg^{-1}$
008	A_{16}	Constant for calculating the conductivity of the mitral valve and pulmonary veins	3.3	(Proshin and Solodyannikov, 2006)	–	$mL \cdot s^{-1} \cdot mmHg^{-2}$
009	A_{18}	Sympathetic sensitivity of the pulmonary arteries	40.0	(Proshin and Solodyannikov, 2006)	–	$mL \cdot s$
010	A_{19}	Pulmonary arterial tone	0.02	(Kutumova et al., 2021)	–	$mmHg \cdot s \cdot mL^{-1}$
011	A_2	Functional status of the body	0.3752	(Proshin and Solodyannikov, 2006)	–	–
012	A_3	Sympathetic sensitivity of the systemic microvessels	0.1	(Proshin and Solodyannikov, 2006)	–	$mL \cdot mmHg^{-1}$
013	A_4	Sensitivity of systemic microvessels to oxygen debt	0.031537	(Proshin and Solodyannikov, 2006)	–	$s^{-1} \cdot mmHg^{-1}$
014	A_5	Sensitivity factor for calculating LV systolic pressure	22.0	(Proshin and Solodyannikov, 2006)	–	$s \cdot mmHg^{-1}$
015	A_6	Constant for calculating the conductivity of the tricuspid valve and systemic veins	3.3	(Proshin and Solodyannikov, 2006)	–	$mL \cdot s^{-1} \cdot mmHg^{-2}$
016	A_7	Constant for calculating the conductivity of the tricuspid valve and systemic veins	4.0	(Proshin and Solodyannikov, 2006)	–	$mmHg^{-1}$
017	A_8	Sympathetic sensitivity of the systemic arteries	45.0	(Proshin and Solodyannikov, 2006)	–	$mL \cdot s$
018	A_9	Systemic arterial tone	0.07	(Proshin and Solodyannikov, 2006)	–	$mmHg \cdot s \cdot mL^{-1}$
019	A_{AT1_aa}	Constant for AT1-bound angiotensin II effect on afferent arteriole resistance	1.3754	(Kutumova et al., 2021)	–	–
020	A_{AT1_ALVL}	Constant for AT1-bound angiotensin II effect on blood flow through systemic microvessels	1.7323	(Kutumova et al., 2021)	–	–
021	A_{AT1_ea}	Constant for AT1-bound angiotensin II effect on efferent arteriole resistance	1.6601	(Kutumova et al., 2021)	–	–
022	$A_{AT1\_preglom}$	Constant for AT1-bound angiotensin II effect on resistance of interlobar, arcuate, and interlobular arteries	0.4464	(Kutumova et al., 2021)	–	–
023	AO_2	Arterial oxygen content	0.2	Calculated as $0.001 \cdot He \cdot C_H \cdot SpO_2$	0.145 – 0.244 (Hattori et al., 2004)	–
024	$AT1\_ANGII_{norm}$	Normal concentration of AT1-bound angiotensin II	16.63	(Hallow and Gebremichael , 2017)	–	$fmol \cdot mL^{-1}$

[3] All parameters of the model, except for the pharmacokinetic parameters of drugs, which are given in Table S5 below
[4] Data range or mean ± SD

025	B_{AT1_aa}	Constant for AT1-bound angiotensin II effect on afferent arteriole resistance	0.0549	(Kutumova et al., 2021)	–	–
026	B_{AT1_ALVL}	Constant for AT1-bound angiotensin II effect on blood flow through systemic microvessels	0.0032	(Kutumova et al., 2021)	–	–
027	B_{AT1_ea}	Constant for AT1-bound angiotensin II effect on efferent arteriole resistance	0.0383	(Kutumova et al., 2021)	–	–
028	$B_{AT1\_preglom}$	Constant for AT1-bound angiotensin II effect on resistance of interlobar, arcuate, and interlobular arteries	0.0661	(Kutumova et al., 2021)	–	–
029	c_{ACE}	Rate of conversion of angiotensin I to angiotensin II by ACE	0.90167	(Hallow et al., 2014)	–	min^{-1}
030	c_{ACE2}	Rate of conversion of angiotensin II to angiotensin-(1-7) by ACE2	0.04	(Hallow et al., 2014)	–	min^{-1}
031	C_{adh_norm}	Normal plasma concentration of antidiuretic hormone	4.97	Fitted	1.0 – 13.3 (Yarmohammadi et al., 2015)	$\text{pg}\cdot\text{mL}^{-1}$
032	C_{al_norm}	Normal plasma concentration of aldosterone	255.04	Fitted	70 – 300 (Fischbach, 2003)	$\text{pg}\cdot\text{mL}^{-1}$
033	c_{ANGII_ANGIV}	Rate of conversion of angiotensin II to angiotensin IV	0.39167	(Hallow et al., 2014)	–	min^{-1}
034	C_{anp_norm}	Normal plasma concentration of atrial natriuretic peptide	35.05	Fitted	7.4 – 152.0 (Cannone et al., 2018; Nozuki et al., 1986)	$\text{ng}\cdot\text{L}^{-1}$
035	c_{AT1}	Rate of angiotensin II binding to AT1 receptors	0.19667	(Hallow et al., 2014)	–	min^{-1}
036	C_{AT1_aa}	Constant for AT1-bound angiotensin II effect on afferent arteriole resistance	0.1437	(Kutumova et al., 2021)	–	–
037	C_{AT1_ALVL}	Constant for AT1-bound angiotensin II effect on blood flow through systemic microvessels	0.2170	(Kutumova et al., 2021)	–	–
038	C_{AT1_ea}	Constant for AT1-bound angiotensin II effect on efferent arteriole resistance	0.2441	(Kutumova et al., 2021)	–	–
039	$C_{AT1\_preglom}$	Constant for AT1-bound angiotensin II effect on resistance of interlobar, arcuate, and interlobular arteries	0.2133	(Kutumova et al., 2021)	–	–
040	c_{AT2}	Rate of angiotensin II binding to AT2 receptors	0.065	(Hallow et al., 2014)	–	min^{-1}
041	c_{chym}	Rate of conversion of angiotensin I to angiotensin II by chymase	0.01833	(Hallow et al., 2014)	–	min^{-1}
042	C_H	Oxygen capacity of hemoglobin	1.35	Fitted	1.32 – 1.39 (Dijkhuizen et al., 1977)	$\text{mL}\cdot\text{g}^{-1}$
043	C_K	Serum potassium concentration	3.88	Fitted	3.5 – 5.5 (Rastegar, 1990)	$\text{mEq}\cdot\text{L}^{-1}$
044	c_{nep}	Rate of conversion of angiotensin I to angiotensin-(1-7) by neprilysin	0.01833	(Hallow et al., 2014)	–	min^{-1}
045	d_{aa}	Afferent arteriolar diameter	19.45	Fitted	8.7 – 23.9 (Neal et al., 2018; Hill et al., 2006)	μm
046	d_{ea}	Efferent arteriolar diameter	17.55	Fitted	13.5 – 18.3 (Neal et al., 2018)	μm
047	$FS_{threshold}$	Frank-Starling law threshold	45.55	Calculated as $m/70 \cdot FS_{threshold0}$	–	mL
048	$FS_{threshold0}$	Average normal value of the Frank-Starling law threshold	39.85	Fitted	–	mL

049	G_{AL0}	Basic systemic arterial elasticity	0.76	Fitted	0.33 – 1.00 (Laskey et al., 1990)	mmHg·mL^{-1}
050	G_{AR0}	Basic pulmonary arterial elasticity	0.15	Fitted	0.08 – 0.26 (Thenappan et al., 2016)	mmHg·mL^{-1}
051	G_{HL}	LV wall elasticity	0.079	Fitted	0.01 – 0.43 (Zhang and Kovács, 2008)	mmHg·mL^{-1}
052	G_{HR}	RV wall elasticity	0.048	Fitted	0.01 – 0.43 (Zhang and Kovács, 2008)	mmHg·mL^{-1}
053	G_{VL0}	Basic elasticity of the systemic veins	0.028	Fitted	–	mmHg·mL^{-1}
054	G_{VR0}	Basic elasticity of the pulmonary veins	0.046	Fitted	–	mmHg·mL^{-1}
055	$glucose$	Plasma glucose	5.01	Fitted	3.9 – 6.1 (Dedov et al., 2017)	mmol·L^{-1}
056	h_{ANG17}	Half-life of angiotensin (1-7)	30.0	(Hallow et al., 2014)	19.2 – 51.6 (Rodgers et al., 2006)	min
057	h_{ANGI}	Half-life of angiotensin I	0.25	(Kutumova et al., 2021)	0.25 ± 0.08 (Admiraal et al., 1993)	min
058	h_{ANGII}	Half-life of angiotensin II	0.9	(Kutumova et al., 2021)	Men: 1.0; Women: 0.8 (Magness et al., 1994; Donato et al., 1972)	min
059	h_{ANGIV}	Half-life of angiotensin IV	0.5	(Hallow et al., 2014)	–	min
060	h_{AT1}	Half-life of AT1-bound angiotensin II	12.0	(Hallow et al., 2014)	12.0 (Inada et al., 1999)	min
061	h_{AT2}	Half-life of AT2-bound angiotensin II	12.0	(Hallow et al., 2014)	–	min
062	h_{renin}	Half-life of circulating renin	12.0	(Hallow et al., 2014)	10.0 – 15.0 (Skrabal, 1974)	min
063	Hct	Hematocrit	42.62	Fitted	Men: 40 – 54 Women: 36 – 48 (Billett, 1990)	%
064	He	Hemoglobin	154.3	Fitted	Men:140–180 Women:120–160 (Billett, 1990)	g·L^{-1}
065	$Heart_{Base}$	Basic activity of the cardiac center	0.17	Fitted	–	–
066	$Heart_{Baro}$	Baroreceptor sensitivity of the cardiac center	0.6	(Proshin and Solodyannikov, 2006)	–	–
067	$Heart_{Oxygen}$	Sensitivity of the cardiac center to fatigue	1.75	(Proshin and Solodyannikov, 2006)	–	–
068	$Heart_{Stress}$	Stress sensitivity of the cardiac center	1.5	(Proshin and Solodyannikov, 2006)	–	–
069	$Heart_{VO2}$	Respiratory sensitivity of the cardiac center	1.0	(Proshin and Solodyannikov, 2006)	–	–
070	k_{AL}	Ratio of unstressed volume to stressed volume of the systemic arteries	0.92	Fitted	0.7 – 1.0 (Magder, 2016)	–
071	k_{AR}	Ratio of unstressed volume to stressed volume of the pulmonary arteries	0.85	Fitted	0.7 – 1.0 (Magder, 2016)	–

072	K_{FG_0}	Normal glomerular filtration coefficient	0.0051	Fitted	0.0039–0.0162 (Hoang et al., 2003)	$L \cdot min^{-1} \cdot mmHg^{-1}$
073	k_{HL}	Ratio of unstressed volume to stressed volume of the left ventricle	0.13	Fitted	0.0 – 0.3 (Kutumova et al., 2021)	–
074	K_{HLAL}	Aortic valve regurgitation coefficient	0.0	(Proshin and Solodyannikov, 2006)	–	–
075	k_{HR}	Ratio of unstressed volume to stressed volume of the right ventricle	0.15	Fitted	0.0 – 0.3 (Kutumova et al., 2021)	–
076	K_{HRAR}	Pulmonary valve regurgitation coefficient	0.0	(Proshin and Solodyannikov, 2006)	–	–
077	k_{VL}	Ratio of unstressed volume to stressed volume of the systemic veins	1.0	Fitted	0.7 – 1.0 (Magder, 2016)	–
078	K_{VLHR}	Tricuspid valve regurgitation coefficient	0.0	(Proshin and Solodyannikov, 2006)	–	–
079	k_{VR}	Ratio of unstressed volume to stressed volume of the pulmonary veins	0.98	Fitted	0.7 – 1.0 (Magder, 2016)	–
080	K_{VRHL}	Mitral valve regurgitation coefficient	0.0	(Proshin and Solodyannikov, 2006)	–	–
081	K_{L0}	Inotropic status of the LV	0.56	Fitted	0.50 – 0.80 (Solodyannikov, 1994)	–
082	K_{R0}	Inotropic status of the RV	0.52	Fitted	0.50 – 0.80 (Solodyannikov, 1994)	–
083	L_{aa}	Afferent arteriolar length	120.8	Fitted	112.0 (Neal et al., 2018)	μm
084	L_{ea}	Efferent arteriolar length	127.5	Fitted	138.0 (Neal et al., 2018)	μm
085	m	Body mass	80.0	Fitted	–	kg
086	$N_{nephrons}$	Number of nephrons in the kidneys	2702905	Fitted	1.50E6 – 3.00E6 (Bertram et al., 2011)	–
087	N_{rsna}	Normalized renal sympathetic nerve activity	1.0	(Karaaslan et al., 2005)	–	–
088	n_{ε_dt}	Normal fractional distal sodium reabsorption	0.44	Fitted	–	–
089	n_{η_cd}	Normal fractional collecting duct sodium reabsorption	0.82	Fitted	–	–
090	n_{η_pt}	Normal fractional sodium reabsorption in the proximal tubule and the loop of Henle	0.85	Fitted	0.67 – 0.97 (Fliser et al., 1997; Bochud et al., 2009; Jin et al., 2009)	–
091	$n_{\varepsilon\_dt} + n_{\eta\_cd} - -n_{\eta\_cd} \cdot n_{\varepsilon\_dt}$	Normal fractional sodium reabsorption in the distal tubule and subsequent parts of the nephron	0.90	Fitted	0.78 – 0.98 (Fliser et al., 1997; Bochud et al., 2009; Jin et al., 2009)	–
092	P_0	Baroreceptor sensitivity threshold	2.0	(Proshin and Solodyannikov, 2006)	–	mmHg
093	P_B	Hydrostatic pressure in the Bowman's space	13.35	Fitted	10.0 – 15.0 (Digne-Malcolm et al., 2016)	mmHg
094	P_{gh_norm}	Normal glomerular hydrostatic pressure	56.84	Fitted	48.0 – 63.0 (Guberina et al., 2013)	mmHg

095	P_v	Renal venous pressure	6.0	(Digne-Malcolm et al., 2016)	–	mmHg
096	PRC_{nom}	Nominal value of plasma renin concentration	24.62	Fitted	3.42 – 69.4 (Perschel et al., 2004)	pg·mL^{-1}
097	$R_{preglom\_0}$	Nominal resistance of interlobar, arcuate, and interlobular arteries	10.08	Fitted	10.0 – 20.0 (Hallow and Gebremichael , 2017)	mmHg·min·L^{-1}
098	R_v	Renal venous resistance	18.10	Fitted	11.3 – 20.1 (Kutumova et al., 2021)	mmHg·min·L^{-1}
099	RO_2	Oxygen demand	6.26	Calculated as $m/70 \cdot RO_{20}$	–	mL·s^{-1}
100	RO_{20}	Average normal value of oxygen demand	5.48	Fitted	≈ 4.2 (Treacher and Leach, 1998)	mL·s^{-1}
101	sl_{baro}	Slope of the linear function of the effect of AT1-bound angiotensin II on baroreceptor activity	0.0499	(Kutumova et al., 2022)	–	–
102	sl_{KFG}	Slope of the linear function of the effect of AT1-bound angiotensin II on filtration coefficient	0.2015	(Kutumova et al., 2022)	–	–
103	sl_{Pgh}	Slope of the linear function of the effect of glomerular hydrostatic pressure on myogenic autoregulation signal	0.8294	(Kutumova et al., 2021)	–	–
104	sl_{stress}	Slope of the linear function of the effect of AT1-bound angiotensin II on stress receptor activity	0.2133	(Kutumova et al., 2022)	–	–
105	$slope_{AT1\_PRC}$	Constant for AT1-bound angiotensin II effect on renin secretion rate	1.2	(Hallow and Gebremichael , 2017)	–	–
106	SpO_2	Arterial oxygen saturation	0.96	Fitted	0.95 – 0.99 (Goldberg et al., 2017)	–
107	st	Stress factor, level of steroid hormones in the blood	0.25	(Proshin and Solodyannikov, 2006)	–	–
108	SV_{max}	Theoretical maximum stroke volume	228.57	Calculated as $m/70 \cdot SV_{max0}$	–	mL
109	SV_{max0}	Average value of theoretical maximum stroke volume	200.0	(Proshin and Solodyannikov, 2006)	–	mL
110	T_{al}	Time constant for calculating the aldosterone secretion	30.0	(Karaaslan et al., 2005)	–	min
111	TP	Total protein	75.72	Fitted	60.0 – 86.0 (Busher, 1990; Gardner and Scott, 1980)	g·L^{-1}
112	$urea$	Plasma urea concentration	2.02	Fitted	1.8 – 7.1 (Hosten, 1990)	mmol·L^{-1}
113	Vis	Blood viscosity	4.86	Calculated as $Vis = 1.23 \cdot \left(1 - \frac{Hct}{99}\right)^{-n}$, $n = 1.7 + 9.86 \cdot \exp(-0.0607 \cdot Hct)$	4.66 ± 0.72 (Furukawa et al., 2016)	cP
114	Vis_{norm}	Average normal blood viscosity	4.65	(Hund et al., 2017)	4.66 ± 0.72 (Furukawa et al., 2016)	cP
115	X_{PRC_PRA}	Equilibrium ratio of plasma renin activity to plasma renin concentration	0.87	Fitted	–	fmol·min^{-1}·pg^{-1}
116	Y_{ALVL0}	Basic conductivity of the systemic microvessels	1.363	(Kutumova et al., 2021)	–	mL·s^{-1}·mmHg^{-1}
117	Y_{ARVR0}	Basic conductivity of the pulmonary microvessels	13.89	Fitted	–	mL·s^{-1}·mmHg^{-1}

118	Y_{HLAL}	Conductivity of the aortic valve and systemic arteries	7.0	(Kutumova et al., 2021)	–	$mL \cdot s^{-1} \cdot mmHg^{-1}$
119	Y_{HRAR}	Conductivity of the pulmonary valve and pulmonary arteries	57.0	(Kutumova et al., 2021)	–	$mL \cdot s^{-1} \cdot mmHg^{-1}$
120	Y_{VLHR0}	Basic conductivity of the tricuspid valve and systemic veins	90.0	(Kutumova et al., 2021)	–	$mL \cdot s^{-1} \cdot mmHg^{-1}$
121	Y_{VRHL0}	Basic conductivity of the mitral valve and pulmonary veins	57.0	(Kutumova et al., 2021)	–	$mL \cdot s^{-1} \cdot mmHg^{-1}$
122	ξ_{map}	Effect of mean arterial pressure on aldosterone secretion	1.0	(Karaaslan et al., 2005)	–	–
123	τ_{MD_renin}	Constant to calculate the effect of macula densa sodium flow on renin secretion rate	0.0959	(Kutumova et al., 2021)	–	–
124	$\Phi_{md\_sod\_0}$	Nominal value of macula densa sodium flow rate	2.80	Fitted	–	$mEq \cdot min^{-1}$
125	Φ_{sodin}	Sodium intake	0.048	Fitted	0.035 – 0.174<br>(Luft et al., 1982)	$mEq \cdot min^{-1}$
126	Φ_{win_norm}	Normal value of water intake	0.0016	Fitted	0.0019 ± 0.0007<br>(Malisova et al., 2016)	$L \cdot min^{-1}$
127	ω_{AL_nom}	Nominal unstressed volume of the systemic arteries	551.2	$\approx k_{AL} \cdot V_{AL}(0)$	–	mL
128	ω_{AR_nom}	Nominal unstressed volume of the pulmonary arteries	136.7	$\approx k_{AR} \cdot V_{AR}(0)$	–	mL
129	ω_{HL}	Unstressed LV volume	18.1	$\approx k_{HL} \cdot V_{HL}(0)$	0.0 – 42.0<br>(Kutumova et al., 2021)	mL
130	ω_{HR}	Unstressed RV volume	20.6	$\approx k_{HR} \cdot V_{HR}(0)$	0.0 – 42.0<br>(Kutumova et al., 2021)	mL
131	ω_{VL}	Unstressed volume of the systemic veins	3260.3	$\approx k_{VL} \cdot V_{VL}(0)$	–	mL
132	ω_{VR}	Unstressed volume of the pulmonary veins	292.1	$\approx k_{VR} \cdot V_{VR}(0)$	–	mL

Table S4. Model variables with equilibrium values for a normotensive person

№	Variables	Description	Values	Normal range[5]	Units
001	$ANG17$	Plasma angiotensin-(1-7) concentration	14.792	14.1 – 31.7<br>(Ferrario et al., 1998)	$fmol \cdot mL^{-1}$
002	$ANGI$	Plasma angiotensin I concentration	7.860	2.8 – 28.5<br>(Lawrence et al., 1990;<br>Nussberger et al., 1992)	$fmol \cdot mL^{-1}$
003	$ANGII$	Plasma angiotensin II concentration	4.941	0.0 – 21.4<br>(Lawrence et al., 1990; Nussberger et al., 1992; Duggan et al., 1993)	$fmol \cdot mL^{-1}$
004	$ANGIV$	Plasma angiotensin IV concentration	1.396	–	$fmol \cdot mL^{-1}$
005	$AT1\_ANGII$	Concentration of AT1-bound angiotensin II	16.824	–	$fmol \cdot mL^{-1}$
006	$AT2\_ANGII$	Concentration of AT2-bound angiotensin II	5.561	–	$fmol \cdot mL^{-1}$
007	C_{adh}	Plasma concentration of antidiuretic hormone	2.785	1.0 – 13.3<br>(Yarmohammadi et al., 2015)	$pg \cdot mL^{-1}$
008	C_{al}	Plasma aldosterone concentration	215.385	70 – 300<br>(Fischbach, 2003)	$pg \cdot mL^{-1}$
009	C_{anp}	Plasma concentration of atrial natriuretic peptide	42.736	7.4 – 152.0<br>(Cannone et al., 2018;<br>Nozuki et al., 1986)	$ng \cdot L^{-1}$
010	C_M	Plasma protein mean concentration within the glomerular capillaries	8.035	–	$g \cdot L^{-1}$
011	C_{sod}	Serum sodium concentration	143.594	137 – 147<br>(Payne and Levell, 1968)	$mEq \cdot L^{-1}$
012	CO	Cardiac output	5.019	2.51 – 9.00<br>(Cattermole et al., 2017)	$L \cdot min^{-1}$
013	$Cycle_{Length}$	Cardiac cycle length (RR-interval)	0.812	Men: 0.624 – 1.284<br>Women: 0.600 – 1.213<br>(Mason et al., 2007)	s
014	$Cycle_{Time}$	Current cardiac cycle time	0.492	–	s
015	DO_2	Oxygen debt	6.054	–	mL
016	DTS_L	LV systolic mismatch	0.0	–	–
017	DTS_R	RV systolic mismatch	0.0	–	–
018	EF	Ejection fraction	55.199	50 – 80<br>(Pfisterer et al., 1985;<br>Saghiv and Sagiv, 2017)	%
019	F_{ALVL}	Blood flow through the systemic microvessels	84.176	–	$mL \cdot s^{-1}$
020	F_{ARVR}	Blood flow through the pulmonary microvessels	83.486	–	$mL \cdot s^{-1}$
021	F_{HLAL}	Blood flow through the aortic valve	0.0	–	$mL \cdot s^{-1}$
022	F_{HLAL_p}	Peak flow rate of F_{HLAL}	461.530	347.0 – 677.0<br>(Kyhl et al., 2013)	$mL \cdot s^{-1}$

[5] Data range or mean ± SD

023	F_{HRAR}	Blood flow through the pulmonary valve	0.0	–	$mL \cdot s^{-1}$
024	F_{HRAR_p}	Peak flow rate of F_{HRAR}	589.434	264.5 – 793.0 (Kyhl et al., 2013; Macedo et al., 2007)	$mL \cdot s^{-1}$
025	F_{VLHR}	Blood flow through the tricuspid valve	68.810	–	$mL \cdot s^{-1}$
026	F_{VLHR_ap}	Active peak of F_{VLHR}	433.925	29 – 770 (Maceira et al., 2006a)	$mL \cdot s^{-1}$
027	F_{VLHR_ep}	Early peak of F_{VLHR}	599.566	105 – 753 (Maceira et al., 2006a)	$mL \cdot s^{-1}$
028	F_{VRHL}	Blood flow through the mitral valve	67.323	–	$mL \cdot s^{-1}$
029	F_{VRHL_ap}	Active peak of F_{VRHL}	332.964	77 – 497 (Maceira et al., 2006b)	$mL \cdot s^{-1}$
030	F_{VRHL_ep}	Early peak of F_{VRHL}	798.271	178 – 1000 (Maceira et al., 2006b)	$mL \cdot s^{-1}$
031	FF	Filtration fraction	0.113	–	–
032	G_{AL}	Systemic arterial elasticity	0.949	0.33 – 1.00 (Laskey et al., 1990)	$mmHg \cdot mL^{-1}$
033	G_{AR}	Pulmonary arterial elasticity	0.194	0.08 – 0.26 (Thenappan et al., 2016)	$mmHg \cdot mL^{-1}$
034	G_{VL}	Systemic venous elasticity	0.075	–	$mmHg \cdot mL^{-1}$
035	G_{VR}	Pulmonary venous elasticity	0.096	–	$mmHg \cdot mL^{-1}$
036	GFR	Glomerular filtration rate	0.077	0.060 – 0.135 (Levin and Stevens, 2013; Cachat et al., 2015)	$L \cdot min^{-1}$
037	gO_2	Oxygen consumption	6.291	≈ 4.2 (Treacher and Leach, 1998)	$mL \cdot s^{-1}$
038	H	Neurohumoral factor	1.220	–	s^{-1}
039	$Heart_{Rate}$	Heart rate	73.890	60 – 100 (Ostchega et al., 2011)	$beats \cdot min^{-1}$
040	K_{FG}	Glomerular filtration coefficient	0.0051	0.0039–0.0162 (Hoang et al., 2003)	$L \cdot min^{-1} \cdot mmHg^{-1}$
041	K_L	Inotropic factor of the LV	0.621	–	–
042	K_R	Inotropic factor of the RV	0.583	–	–
043	LA_{PULSE}	Pulse wave of the left atrium	0.0	–	mmHg
044	M_{sod}	Total exchangeable sodium	2707.016	2040 – 3950 (Farber and Soberman, 1956)	mEq
045	MAP	Mean arterial pressure	93.799	70 – 105 (Doenyas-Barak et al., 2019)	mmHg
046	N_{al}	Normalized aldosterone concentration	0.845	–	–
047	N_{als}	Normalized aldosterone secretion rate	0.845	–	–
048	nB	Baroreceptor activity	0.457	–	–
049	nD	Fatigue receptor activity	0.070	–	–

050	nH	Sympathetic inotropic sensitivity of the myocardium	0.273	–	–
051	nS	Stress receptor activity	0.181	–	–
052	$nSum$	Activity of the cardiac center	1.265	–	–
053	nV	Respiratory receptor activity	0.426	–	–
054	$osmolality$	Blood osmolality	283.108	275 – 295 (Fogarty and Loughrey, 2016)	$mOsm \cdot kg^{-1}$
055	P_{AL}	Systemic arterial pressure	101.943	–	mmHg
056	P_{AR}	Pulmonary arterial pressure	14.016	–	mmHg
057	P_{AR_D}	Diastolic pulmonary arterial pressure	11.160	4 – 12 (Marini and Leatherman, 2005; Pagani et al., 1988)	mmHg
058	P_{AR_S}	Systolic pulmonary arterial pressure	18.358	15 – 30 (Marini and Leatherman, 2005; Pagani et al., 1988)	mmHg
059	P_D	Diastolic blood pressure	80.228	< 90 (Oparil et al., 2018)	mmHg
060	P_{gh}	Glomerular hydrostatic pressure	58.557	48.0 – 63.0 (Guberina et al., 2013)	mmHg
061	P_{go}	Glomerular capillary oncotic pressure	30.173	23.6 – 34.0 (Škrtić et al., 2015; Chagnac et al., 2000; Guasch et al., 1997)	mmHg
062	P_{HL}	LV pressure	7.587	–	mmHg
063	P_{HL_D}	LV diastolic pressure	7.587	3 – 12 (Pagani et al., 1988)	mmHg
064	P_{HL_S}	LV systolic pressure	138.914	100 – 140 (Pagani et al., 1988)	mmHg
065	P_{HR}	RV pressure	4.338	–	mmHg
066	P_{HR_D}	RV diastolic pressure	4.338	2 – 8 (Pagani et al., 1988)	mmHg
067	P_{HR_S}	RV systolic pressure	20.364	15 – 30 (Pagani et al., 1988)	mmHg
068	P_{ra}	Normalized right atrial pressure	0.876	–	–
069	P_S	Systolic blood pressure	120.942	< 140 (Oparil et al., 2018)	mmHg
070	P_{VL}	Systemic venous pressure	4.886	2 – 8 (Klingensmith et al., 2016)	mmHg
071	P_{VR}	Pulmonary venous pressure	8.297	3 – 20 (Kutumova et al., 2021)	mmHg
072	PRA	Plasma renin activity	29.170	15.0 – 31.7 (Valabhji et al., 2001)	$fmol \cdot mL^{-1} \cdot min^{-1}$
073	PRC	Plasma renin concentration	33.414	3.42 – 69.4 (Perschel et al., 2004)	$pg \cdot mL^{-1}$
074	PVR	Pulmonary vascular resistance	0.093	0.0151 – 0.1353 (Klingensmith et al., 2016; Stefanadis et al., 2001)	$s \cdot mmHg \cdot mL^{-1}$

075	R_a	Resistance of the afferent arterioles, and interlobar, arcuate, and interlobular arteries	29.827	–	$mmHg·min·mL^{-1}$
076	R_{a_dyne}	R_a in $dyn·s·cm^{-5}$	2376.597	753 – 6863 (Tsuda et al., 2018; Kutumova et al., 2021)	$dyn·s·cm^{-5}$
077	R_{aa}	Single afferent arteriole resistance	2.746E7	–	$mmHg·min·L^{-1}$
078	R_{aa_0}	Nominal single afferent arteriole resistance	2.089E7	–	$mmHg·min·L^{-1}$
079	R_e	Resistance of all efferent arterioles	28.212	–	$mmHg·min·mL^{-1}$
080	R_{e_dyne}	R_e in $dyn·s·cm^{-5}$	2247.921	1669 – 2843 (Kutumova et al., 2021)	$dyn·s·cm^{-5}$
081	R_{ea}	Single efferent arteriole resistance	7.625E7	–	$mmHg·min·L^{-1}$
082	R_{ea_0}	Nominal single efferent arteriole resistance	3.330E7	–	$mmHg·min·L^{-1}$
083	$R_{preglom}$	Resistance of interlobar, arcuate, and interlobular arteries	19.668	10.0 – 20.0 (Hallow and Gebremichael , 2017)	$mmHg·min·L^{-1}$
084	R_{sec}	Renin secretion rate	1.930	–	$pg·mL^{-1}·min^{-1}$
085	RA_{PULSE}	Pulse wave of the right atrium	0.0	–	mmHg
086	*RBF*	Renal blood flow	1.182	0.623 – 1.730 (Bax et al., 2005)	$L·min^{-1}$
087	*RPF*	Renal plasma flow	0.678	0.628 ± 0.162 (Škrtić et al., 2015)	$L·min^{-1}$
088	*RSNA*	Renal sympathetic nerve activity	1.154	–	–
089	*RVR*	Renal vascular resistance	74.308	55.1 – 83.6 (Kutumova et al., 2021)	$mmHg·min·L^{-1}$
090	*SV*	LV stroke volume	67.927	39.1 – 115.3 (Cattermole et al., 2017)	mL
091	*SVR*	Systemic vascular resistance (can be estimated by total peripheral resistance *TPR*)	1.253	0.5271 – 1.2048 (Klingensmith et al., 2016)	$s·mmHg·mL^{-1}$
092	*Systole*	Indicator of the total actual systole	0.0	–	–
093	$Systole_L$	Indicator of the actual LV systole	0.0	–	–
094	$Systole_{L\_Exp}$	Indicator of the nominal LV systole	0.0	–	–
095	$Systole_{Length\_L}$	Duration of the actual LV systole	0.278	–	s
096	$Systole_{Length\_L\_Exp}$	Duration of the nominal LV systole	0.278	–	s
097	$Systole_{Length\_R}$	Duration of the actual RV systole	0.286	–	s
098	$Systole_{Length\_R\_Exp}$	Duration of the nominal RV systole	0.286	–	s
099	$Systole_R$	Indicator of the actual RV systole	0.0	–	–
100	$Systole_{R\_Exp}$	Indicator of the nominal RV systole	0.0	–	–
101	*TBW*	Total body water	35.521	24.45 – 56.63 (Hoffer et al., 1969)	L
102	*TPR*	Total peripheral resistance	18.688	12.5 – 22.5 (Daly and Bondurant, 1962)	$mmHg·min·L^{-1}$
103	*V*	Total blood volume	4610.630	3061 – 6092 (Wennesland et al., 1959)	mL

104	V_{AL}	Systemic arterial blood volume	595.910	–	mL
105	V_{AR}	Pulmonary arterial blood volume	153.369	–	mL
106	V_{ecf}	Extracellular fluid volume	15.843	–	L
107	V_{HL}	LV blood volume	104.098	–	mL
108	V_{HL_KD}	LV end-diastolic volume	123.058	Men: 67 – 155; Women: 56 – 104 (Lang et al., 2006)	mL
109	V_{HL_KS}	LV end-systolic volume	55.130	Men: 22 – 58; Women: 19 – 49 (Lang et al., 2006)	mL
110	V_{HR}	RV blood volume	101.156	–	mL
111	V_{HR_KD}	RV end-diastolic volume	127.237	Men: 124 – 256; Women: 78 – 218 (Hudsmith et al., 2005)	mL
112	V_{HR_KS}	RV end-systolic volume	59.310	Men: 38 – 118; Women: 20 – 92 (Hudsmith et al., 2005)	mL
113	V_{VL}	Systemic venous blood volume	3298.698	~ 2000 – 3500 (Hall, 2011)	mL
114	V_{VR}	Pulmonary venous blood volume	357.400	–	mL
115	$V_{AR} + V_{VR}$	Pulmonary blood volume	510.769	~ 0.09·V – 0.10·V (Hall, 2011; Gazioglu and Yu, 1967)	mL
116	$V_{AR} + V_{VR} + V_{HR} + V_{HL}$	Cardiopulmonary blood volume	716.023	~ 0.153·V (Levinson et al., 1996)	mL
117	VO_2	Venous oxygen content	0.125	0.095 – 0.168 (Hattori et al., 2004)	–
118	Y_{ALVL}	Conductivity of the systemic microvessels	1.609	–	$mL \cdot s^{-1} \cdot mmHg^{-1}$
119	Y_{ARVR}	Conductivity of the pulmonary microvessels	15.273	–	$mL \cdot s^{-1} \cdot mmHg^{-1}$
120	Y_{VLHR}	Conductivity of the tricuspid valve and systemic veins	125.542	–	$mL \cdot s^{-1} \cdot mmHg^{-1}$
121	Y_{VRHL}	Conductivity of the mitral valve and pulmonary veins	94.801	–	$mL \cdot s^{-1} \cdot mmHg^{-1}$
122	α_{map}	Effect of mean arterial pressure on renal sympathetic nerve activity	1.162	–	–
123	α_{rap}	Effect of right atrial pressure on renal sympathetic nerve activity	0.993	–	–
124	β_{rsna}	Effect of renal sympathetic nerve activity on afferent arteriole resistance and resistance of interlobar, arcuate, and interlobular arteries	1.231	–	–
125	γ_{at}	Effect of AT1-bound angiotensin II on fractional sodium reabsorption in the proximal tubule and loop of Henle	0.998	–	–
126	γ_{filsod}	Effect of filtered sodium load on fractional sodium reabsorption in the proximal tubule and loop of Henle	1.100	–	–
127	γ_{rsna}	Effect of renal sympathetic nerve activity on fractional sodium reabsorption in the proximal tubule and loop of Henle	1.002	–	–
128	$\eta_{cd\_sodreab}$	Fractional collecting duct sodium reabsorption	0.876	–	–
129	$\eta_{dt\_sodreab}$	Fractional distal sodium reabsorption	0.460	–	–
130	$\eta_{pt\_sodreab}$	Fractional sodium reabsorption in the proximal tubule and loop of Henle	0.935	0.67 – 0.97 (Fliser et al., 1997; Bochud et al., 2009; Jin et al., 2009)	–

131	$\eta_{dt\_sodreab} + \eta_{cd\_sodreab} - -\eta_{dt\_sodreab} \cdot \eta_{cd\_sodreab}$	Fractional sodium reabsorption in the distal tubule and subsequent parts of the nephron	0.933	0.78 – 0.98 (Fliser et al., 1997; Bochud et al., 2009; Jin et al., 2009)	–
132	λ_{anp}	Effect of natriuretic peptide on fractional collecting duct sodium reabsorption	0.998	–	–
133	λ_{dt}	Effect of distal sodium outflow on fractional collecting duct sodium reabsorption	1.073	–	–
134	μ_{adh}	Effect of antidiuretic hormone concentration on tubular water reabsorption rate	0.774	–	–
135	ν_{AT1_ANGII}	Effect of AT1-bound angiotensin II on renin secretion rate	0.986	–	–
136	ν_{MD_sod}	Effect of macula densa sodium flow on renin secretion rate	1.222	–	–
137	ν_{RSNA}	Effect of renal sympathetic nerve activity on renin secretion rate	1.126	–	–
138	ξ_{at}	Effect of angiotensin hormone on aldosterone secretion rate	1.294	–	–
139	ξ_{k_sod}	Effect of potassium concentration on aldosterone secretion rate	0.653	–	–
140	Σ_{myo}	Myogenic autoregulation signal	1.025	–	–
141	Σ_{tgf}	Tubuloglomerular feedback signal	0.455	–	–
142	$\Phi_{cd\_sodreab}$	Absolute collecting duct sodium reabsorption rate	0.337	–	$mEq \cdot min^{-1}$
143	Φ_{dt_sod}	Distal sodium outflow	0.384	–	$mEq \cdot min^{-1}$
144	$\Phi_{dt\_sodreab}$	Absolute distal sodium reabsorption rate	0.327	–	$mEq \cdot min^{-1}$
145	Φ_{filsod}	Amount of sodium filtered from the glomerulus to the proximal tubule per minute (filtered sodium load)	11.002	10.0 – 23.0 (Natarajan et al., 2016)	$mEq \cdot min^{-1}$
146	Φ_{md_sod}	Macula densa sodium flow rate	0.712	–	$mEq \cdot min^{-1}$
147	$\Phi_{pt\_sodreab}$	Absolute proximal sodium reabsorption rate	10.290	–	$mEq \cdot min^{-1}$
148	Φ_{t_wreab}	Tubular water reabsorption rate	0.075	–	$L \cdot min^{-1}$
149	Φ_{u}	Urine flow rate	0.0011	0.0011 ± 0.0005 (Malisova et al., 2016)	$L \cdot min^{-1}$
150	Φ_{u_sod}	Urine sodium flow rate	0.048	0.097 ± 0.049 (Letcher et al., 1981)	$mEq \cdot min^{-1}$
151	Φ_{win}	Water intake	0.0011	0.0019 ± 0.0007 (Malisova et al., 2016)	$L \cdot min^{-1}$
152	ψ_{al}	Effect of aldosterone on fractional distal sodium reabsorption	1.047	–	–
153	ψ_{AT1_aa}	Effect of AT1-bound angiotensin II on afferent arteriole resistance	2.291	–	–
154	ψ_{AT1_ALVL}	Effect of AT1-bound angiotensin II on blood flow through the systemic microvessels	1.773	–	–
155	ψ_{AT1_Baro}	Effect of AT1-bound angiotensin II on baroreceptor activity	1.001	–	–
156	ψ_{AT1_ea}	Effect of AT1-bound angiotensin II on efferent arteriole resistance	2.290	–	–
157	$\psi_{AT1\_preglom}$	Effect of AT1-bound angiotensin II on resistance of interlobar, arcuate, interlobular arteries	1.546	–	–
158	ψ_{AT1_Stress}	Effect of AT1-bound angiotensin II on stress receptor activity	1.002	–	–
159	ω_{AL}	Unstressed volume of the systemic arteries	488.449	–	mL
160	ω_{AR}	Unstressed volume of the pulmonary arteries	80.947	–	mL

Table S5. Target variables of antihypertensive drugs in the model[6]

Therapy	Target variables			Drug effect		Value (E_v)	References
	Module	Symbol	Definition	Sign[7]	Parameter		
Aliskiren, 300 mg/day	Renal system	PRC	Plasma renin concentration	–	DRI	0.928	(Kutumova et al., 2022)
Amlodipine, 5 mg/day	Renal system	R_{aa}	Single afferent arteriole resistance	–	CCB_{aa}	0.413	(Kutumova et al., 2022)
		R_{ea}	Single efferent arteriole resistance	–	CCB_{ea}	0.107	
		$R_{preglom}$	Resistance of the interlobar, arcuate, and interlobular arteries	–	$CCB_{preglom}$	0.413	
	Cardiovascular system	R_{ALVL}	Resistance of the systemic microvessels	–	CCB_{sys}	0.107	
Bisoprolol, 5 mg/day	Cardiovascular system	nS	Activity of the stress receptors	–	$B_{blocker}$	0.467	Values were fitted to SBP, DBP, and HR response in clinical trials (Porthan et al., 2009).
		K_L, K_R	Inotropic factors of the left and right ventricles	–	$B_{blocker\_in}$	0.092	
		G_{AL}	Systemic arterial elasticity	–	$B_{blocker\_st}$	0.149	
	Renal system	R_{sec}	Renin secretion rate	–	$B_{blocker\_rs}$	0.926	
Enalapril, 20 mg/day	Renal system	c_{ACE}	Rate of conversion of angiotensin I to angiotensin II by ACE	–	$ACEi$	0.996	(Kutumova et al., 2022)
HCTZ, 12.5 mg/day	Renal system	$\eta_{dt\_sodreab}$	Fractional distal sodium reabsorption	–	$Diuretic_{Inhibition}$	0.292	Values were adjusted to better fit SBP and DBP response in clinical trials (MacKay et al., 1996), taking into account the following long-term dynamics:<br>- PRA increases by 45% (Villamil et al., 2007);<br>- HR, ECFV, GFR, and CO do not change significantly (van Brummelen et al., 1980; Shah et al., 1978; Scaglione et al., 1992; Scaglione et al., 1995; Duarte and Cooper-DeHoff, 2010; Rapoport and Soleimani, 2019; Leth, 1970; Digne-Malcolm et al., 2016).
		R_{sec}	Renin secretion rate	+	$Diuretic_{Stimulation}$	1.095	
		R_{aa}	Single afferent arteriole resistance	–	$Diuretic_{aa}$	0.358	
		R_{ea}	Single efferent arteriole resistance	–	$Diuretic_{ea}$	0.202	
		$R_{preglom}$	Resistance of the interlobar, arcuate, and interlobular arteries	–	$Diuretic_{preglom}$	0.419	
	Cardiovascular system	R_{ALVL}	Resistance of the systemic microvessels	–	$Diuretic_{sys}$	$\min\left(\frac{time}{1950000}\cdot 0.063, 0.063\right)$	
		nS	Activity of the stress receptors	–	$Diuretic_{stress}$	0.355	
	Renal system	C_K	Potassium level in the blood	–	$Diuretic_{potassium}$	0.030	(Kutumova et al., 2022)
		$urea$	Urea level in the blood	+	$Diuretic_{urea}$	0.100	
Losartan, 100 mg/day	Renal system	c_{AT1}	Rate of angiotensin II binding to the AT1 receptors	–	ARB	0.954	(Kutumova et al., 2022)

[6] ACE = angiotensin-converting enzyme; CO = cardiac output; DBP = diastolic blood pressure; ECFV = extracellular fluid volume; GFR = glomerular filtration rate; HCTZ = hydrochlorothiazide; HR = heart rate; PRA = plasma renin activity; SBP = systolic blood pressure.

[7] Stimulation: "+"; inhibition: "–".

Table S6. List of parameter ranges used to create virtual patients

№	Parameters	Notations	Ranges			Units
			Norm	Hypertension	References	
01	Total metabolic intensity	A_1	0.00032 – 0.00128		(Kutumova et al., 2021)	mL^{-1}
02	Sympathetic sensitivity of the systemic microvessels	A_3	0.1	0.1 – 0.6	(Proshin and Solodyannikov, 2006; Kutumova et al., 2021)	$mL\cdot mmHg^{-1}$
03	Systemic arterial tone	A_9	0.07	0.07 – 0.09	(Proshin and Solodyannikov, 2006; Kutumova et al., 2021)	$mmHg\cdot s\cdot mL^{-1}$
04	Normal concentration of antidiuretic hormone	C_{adh_norm}	1.0 – 13.3		(Yarmohammadi et al., 2015)	$pg\cdot mL^{-1}$
05	Normal concentration of aldosterone	C_{al_norm}	70 – 300		(Fischbach, 2003)	$pg\cdot mL^{-1}$
06	Normal concentration of natriuretic peptide	C_{anp_norm}	7.4 – 152.0		(Cannone et al., 2018; Nozuki et al., 1986)	$ng\cdot L^{-1}$
07	Oxygen capacity of hemoglobin	C_H	1.32 – 1.39		(Dijkhuizen et al., 1977)	$mL\cdot g^{-1}$
08	Serum potassium	C_K	3.5 – 5.5		(Rastegar, 1990)	$mEq\cdot L^{-1}$
09	Cardiac output (in the renal submodel)	CO	2.51 – 9.00		(Cattermole et al., 2017)	$L\cdot min^{-1}$
10	Afferent arteriolar diameter	d_{aa}	8.7 – 23.9		(Neal et al., 2018; Hill et al., 2006)	μm
11	Efferent arteriolar diameter	d_{ea}	12.2 – 20.1		(Kutumova et al., 2021)	μm
12	Average normal value of the Frank-Starling law threshold	$FS_{threshold0}$	0.0 – 40.0		(Kutumova et al., 2021)	mL
13	Basic elasticity of the systemic arteries	G_{AL0}	0.33 – 1.00	0.33 – 1.67	(Laskey et al., 1990; Haluska et al., 2010)	$mmHg\cdot mL^{-1}$
14	Basic elasticity of the pulmonary arteries	G_{AR0}	0.08 – 0.26		(Thenappan et al., 2016)	$mmHg\cdot mL^{-1}$
15	Left ventricular wall elasticity	G_{HL}	0.01 – 0.43	0.02 – 0.72	(Zhang and Kovács, 2008; Kutumova et al., 2021)	$mmHg\cdot mL^{-1}$
16	Right ventricular wall elasticity	G_{HR}	0.01 – 0.43	0.02 – 0.72	(Zhang and Kovács, 2008; Kutumova et al., 2021)	$mmHg\cdot mL^{-1}$
17	Basic elasticity of the systemic veins	G_{VL0}	0.01 – 0.05		(Kutumova et al., 2021)	$mmHg\cdot mL^{-1}$
18	Basic elasticity of the pulmonary veins	G_{VR0}	0.01 – 0.05		(Kutumova et al., 2021)	$mmHg\cdot mL^{-1}$
19	Plasma glucose	*glucose*	3.9 – 6.1		(Dedov et al., 2017)	$mmol\cdot L^{-1}$
20	Hematocrit	*Hct*	Men: 40 – 54<br>Women: 36 – 48		(Billett, 1990)	%
21	Hemoglobin	*He*	Men: 140 – 180<br>Women: 120 – 160		(Billett, 1990)	$g\cdot L^{-1}$
22	Basic activity of the cardiac center	$Heart_{Base}$	0.01 – 1.00		(Kutumova et al., 2021)	–
23	Ratio of unstressed volume to stressed volume of the systemic arteries	k_{AL}	0.7 – 1.0		(Magder, 2016)	–
24	Ratio of unstressed volume to stressed volume of the pulmonary arteries	k_{AR}	0.7 – 1.0		(Magder, 2016)	–
25	Ratio of unstressed volume to stressed volume of the systemic veins	k_{VL}	0.7 – 1.0		(Magder, 2016)	–
26	Ratio of unstressed volume to stressed volume of the pulmonary veins	k_{VR}	0.7 – 1.0		(Magder, 2016)	–
27	Ratio of unstressed volume to stressed volume of the left ventricle	k_{HL}	0.0 – 0.3		(Kutumova et al., 2021)	–
28	Ratio of unstressed volume to stressed volume of the right ventricle	k_{HR}	0.0 – 0.3		(Kutumova et al., 2021)	–

29	Normal glomerular filtration coefficient	K_{FG_0}	0.0039 – 0.0162		(Hoang et al., 2003)	L·min^{-1}·mmHg^{-1}
30	Inotropic status of the left ventricle	K_{L0}	0.5 – 0.8		(Solodyannikov, 1994)	–
31	Inotropic status of the right ventricle	K_{R0}	0.5 – 0.8		(Solodyannikov, 1994)	–
32	Afferent arteriolar length	L_{aa}	101 – 123		(Kutumova et al., 2021)	µm
33	Efferent arteriolar length	L_{ea}	124 – 152		(Kutumova et al., 2021)	µm
34	Initial value of the total exchangeable sodium	M_{sod}	2040 – 3950		(Farber and Soberman, 1956)	mEq
35	Number of nephrons in the kidneys	$N_{nephrons}$	1.50E6 – 3.00E6	0.80E6 – 2.75E6	(Bertram et al., 2011; Hoy et al., 2006)	–
36	Normal fractional distal sodium reabsorption	n_{ε_dt}	0.3 – 0.7		(Kutumova et al., 2021)	–
37	Normal fractional collecting duct sodium reabsorption	n_{η_cd}	0.6 – 1.0		(Kutumova et al., 2021)	–
38	Normal fractional proximal sodium reabsorption	n_{η_pt}	0.67 – 0.97		(Fliser et al., 1997; Bochud et al., 2009; Jin et al., 2009)	–
39	Hydrostatic pressure in the Bowman's space	P_B	10.0 – 15.0		(Digne-Malcolm et al., 2016)	mmHg
40	Normal value of the glomerular hydrostatic pressure	P_{gh_norm}	48.0 – 63.0		(Guberina et al., 2013)	mmHg
41	Initial value of the glomerular capillary oncotic pressure	P_{go}	23.6 – 34.0		(Škrtić et al., 2015; Chagnac et al., 2000; Guasch et al., 1997)	mmHg
42	Renal venous pressure	P_v	6.0	2.0 – 6.0	(Digne-Malcolm et al., 2016; Kutumova et al., 2021)	mmHg
43	Normal plasma renin concentration	PRC_{nom}	3.42 – 69.4		(Perschel et al., 2004)	pg·mL^{-1}
44	Nominal resistance of interlobar, arcuate, and interlobular arteries	$R_{preglom\_0}$	7.0 – 20.0	7.0 – 28.0	(Kutumova et al., 2021)	mmHg·min·l^{-1}
45	Renal venous resistance	R_v	11.3 – 20.1		(Kutumova et al., 2021)	mmHg·min·l^{-1}
46	Nominal body oxygen demand	RO_{20}	2.52 – 5.88		(Kutumova et al., 2021)	mL·s^{-1}
47	Arterial oxygen saturation	SpO_2	0.95 – 0.99	0.92 – 0.99	(Goldberg et al., 2017; Kutumova et al., 2021)	–
48	Total protein	TP	60.0 – 86.0		(Busher, 1990; Gardner and Scott, 1980)	g·L^{-1}
49	Plasma urea concentration	$urea$	1.8 – 7.1		(Hosten, 1990)	mmol·L^{-1}
50	Initial value of the total body water	TBW	$f(0.9 \cdot N_{BV}) – f(1.1 \cdot N_{BV})$[8]		(Kutumova et al., 2021)	L
51	Initial value of the venous oxygen content	VO_2	0.0855 – 0.1848		(Kutumova et al., 2021)	–
52	Equilibrium ratio of plasma renin activity to plasma renin concentration	X_{PRC_PRA}	0.61 – 1.42		(Kutumova et al., 2021)	fmol·min^{-1}·pg^{-1}
53	Basic conductivity of the systemic microvessels	Y_{ALVL0}	1.363	0.5 – 2.0	(Kutumova et al., 2021)	mL·s^{-1}·mmHg^{-1}
54	Basic conductivity of the pulmonary microvessels	Y_{ARVR0}	9.0 – 21.0		(Kutumova et al., 2021)	mL·s^{-1}·mmHg^{-1}
55	Nominal value of the macula densa sodium flow rate	$\Phi_{md\_sod\_0}$	1.0 – 4.0		(Kutumova et al., 2021)	mEq·min^{-1}
56	Sodium intake	Φ_{sodin}	0.0280 – 0.2088		(Kutumova et al., 2021)	mEq·min^{-1}
57	Normal value of water intake	Φ_{win_norm}	0.00096 – 0.00312		(Kutumova et al., 2021)	L·min^{-1}

[8] To calculate TBW, we used the formula $f(N_{BV}) = (N_{BV} – 650)/111.5$ (Moore, 1967), where N_{BV} is an estimate defined by Nadler et al. (1962). We considered $N_{BV} \pm 10\%$ as the range of total blood volume in normal humans.

Table S7. List of variable ranges used to create virtual patients

№	Variables	Notations/formulas	Ranges			Units
			Norm	Hypertension	References	
01	Arterial oxygen content	AO_2	0.145 – 0.244		(Hattori et al., 2004)	–
02	Plasma angiotensin (1-7)	$ANG17$	14.1 – 31.7	12.7 – 34.9	(Ferrario et al., 1998; Kutumova et al., 2021)	fmol·mL^{-1}
03	Plasma angiotensin I	$ANGI$	2.8 – 28.5		(Lawrence et al., 1990; Nussberger et al., 1992)	fmol·mL^{-1}
04	Plasma angiotensin II	$ANGII$	0.0 – 21.4		(Lawrence et al., 1990; Nussberger et al., 1992; Duggan et al., 1993)	fmol·mL^{-1}
05	Plasma antidiuretic hormone	C_{adh}	1.0 – 13.3		(Yarmohammadi et al., 2015)	pg·mL^{-1}
06	Plasma aldosterone	C_{al}	70 – 300		(Fischbach, 2003)	pg·mL^{-1}
07	Plasma natriuretic peptide	C_{anp}	7.4 – 152.0		(Cannone et al., 2018; Nozuki et al., 1986)	ng·L^{-1}
08	Plasma sodium	C_{sod}	137 – 147		(Payne and Levell, 1968)	mEq·L^{-1}
09	Cardiac output (in the heart submodel)	CO	2.51 – 9.00		(Cattermole et al., 2017)	L·min^{-1}
10	Ejection fraction	EF	50 – 80		(Pfisterer et al., 1985; Saghiv and Sagiv, 2017)	%
11	Blood flow in the systemic microvessels	F_{ALVL}	> 0.0		(Kutumova et al., 2021)	mL·s^{-1}
12	Blood flow in the pulmonary microvessels	F_{ARVR}	> 0.0		(Kutumova et al., 2021)	mL·s^{-1}
13	Peak rate of the transaortic flow	F_{HLAL_p}	347.0 – 677.0		(Kyhl et al., 2013)	mL·s^{-1}
14	Peak rate of the transpulmonary flow	F_{HRAR_p}	264.5 – 793.0		(Kyhl et al., 2013; Macedo et al., 2007)	mL·s^{-1}
15	Active peak filling rate of the right ventricle	F_{VLHR_ap}	Men: 23 – 947; Women: 54 – 680		(Maceira et al., 2006a)	mL·s^{-1}
16	Early peak filling rate of the right ventricle	F_{VLHR_ep}	Men: 8 – 814; Women: -17 – 701		(Maceira et al., 2006a)	mL·s^{-1}
17	Ratio of early to active peak filling rates of the right ventricle	F_{VLHR_ep}/F_{VLHR_ap}	Men: -0.5 – 2.5; Women: -0.4 – 2.5		(Maceira et al., 2006a)	–
18	Active peak filling rate of the left ventricle	F_{VRHL_ap}	Men: 99 – 647; Women: 58 – 508		(Maceira et al., 2006b)	mL·s^{-1}
19	Early peak filling rate of the left ventricle	F_{VRHL_ep}	Men: 21 – 1034; Women: -13 – 967		(Maceira et al., 2006b)	mL·s^{-1}
20	Ratio of early to active peak filling rates of the left ventricle	F_{VRHL_ep}/F_{VRHL_ap}	Men: 0.3 – 5.9; Women: 0.3 – 6.6		(Maceira et al., 2006b)	–
21	Systemic arterial elasticity	G_{AL}	0.33 – 1.00	0.33 – 1.67	(Laskey et al., 1990; Haluska et al., 2010)	mmHg·mL^{-1}
22	Pulmonary arterial elasticity	G_{AR}	0.08 – 0.26		(Thenappan et al., 2016)	mmHg·mL^{-1}
23	Glomerular filtration rate	GFR	0.060 – 0.135		(Levin and Stevens, 2013; Cachat et al., 2015)	L·min^{-1}
24	Total exchangeable sodium	M_{sod}	2040 – 3950		(Farber and Soberman, 1956)	mEq
25	Normal fractional sodium reabsorption in the distal tubule and subsequent parts of the nephron	$n_{\varepsilon\_dt} + n_{\eta\_cd} - n_{\eta\_cd} \cdot n_{\varepsilon\_dt}$	0.78 – 0.98		(Fliser et al., 1997; Bochud et al., 2009; Jin et al., 2009)	–
26	Plasma osmolality	$osmolality$	275 – 295		(Fogarty and Loughrey, 2016)	mOsmol·kg^{-1}
27	Diastolic pulmonary arterial pressure	P_{AR_D}	4.0 – 12.0		(Marini and Leatherman, 2005; Pagani et al., 1988)	mmHg

28	Systolic pulmonary arterial pressure	P_{AR_S}	15.0 – 30.0		(Marini and Leatherman, 2005; Pagani et al., 1988)	mmHg
29	Glomerular hydrostatic pressure	P_{gh}	48.0 – 63.0		(Guberina et al., 2013)	mmHg
30	Glomerular capillary oncotic pressure	P_{go}	23.6 – 34.0		(Škrtić et al., 2015; Chagnac et al., 2000; Guasch et al., 1997)	mmHg
31	Left ventricular diastolic pressure	P_{HL_D}	3.0 – 12.0	1.0 – 18.0	(Pagani et al., 1988; Kasner et al., 2007; Antony et al., 1993)	mmHg
32	Left ventricular systolic pressure	P_{HL_S}	100 – 140	100 – 186	(Pagani et al., 1988; Antony et al., 1993)	mmHg
33	Right ventricular diastolic pressure	P_{HR_D}	2.0 – 8.0	0.0 – 10.0	(Pagani et al., 1988; Ferlinz, 1980; Kasner et al., 2012)	mmHg
34	Right ventricular systolic pressure	P_{HR_S}	15.0 – 30.0		(Pagani et al., 1988)	mmHg
35	Systemic venous pressure	P_{VL}	2.0 –8.0	1.0 – 10.0	(Klingensmith et al., 2016; Ferlinz, 1980)	mmHg
36	Pulmonary venous pressure	P_{VR}	3.0 – 20.0		(Kutumova et al., 2021)	mmHg
37	Plasma renin concentration	PRC	3.42 – 69.4		(Perschel et al., 2004)	pg·mL^{-1}
38	Resistance of the afferent vessels	R_{a_dyne}	753 – 6863	3000 – 25000	(Tsuda et al., 2018; Gomez, 1951; Kutumova et al., 2021)	dyn·s·cm^{-5}
39	Resistance of the efferent arterioles	R_{e_dyne}	1350 – 3400		(Kutumova et al., 2021)	dyn·s·cm^{-5}
40	Resistance of the interlobar, arcuate, and interlobular arteries	$R_{preglom}$	7.0 – 20.0	7.0 – 28.0	(Kutumova et al., 2021)	mmHg·min·L^{-1}
41	Renal blood flow	RBF	0.623 – 1.730		(Bax et al., 2005)	L·min^{-1}
42	Renal vascular resistance	RVR	55.0 – 84.0	55.0 – 190.0	(Bauer et al., 1982; Kutumova et al., 2021)	mmHg·min·L^{-1}
43	Total blood volume	V	$0.9 \cdot N_{BV} – 1.1 \cdot N_{BV}$[9]		(Kutumova et al., 2021)	mL
44	Left ventricular end-diastolic volume	V_{HL_KD}	Men: 67 – 155; Women: 56 – 104		(Lang et al., 2006)	mL
45	Left ventricular end-systolic volume	V_{HL_KS}	Men: 22 – 58; Women: 19 – 49		(Lang et al., 2006)	mL
46	Right ventricular end-diastolic volume	V_{HR_KD}	Men: 124 – 256; Women: 78 – 218		(Hudsmith et al., 2005)	mL
47	Right ventricular end-systolic volume	V_{HR_KS}	Men: 38 – 118; Women: 20 – 92		(Hudsmith et al., 2005)	mL
48	Venous oxygen content	VO_2	0.0855 – 0.1848		(Kutumova et al., 2021)	–
49	Systemic vascular resistance	SVR	0.5271 – 1.2048	0.5271 – 1.9608	(Klingensmith et al., 2016; Prys-Roberts et al., 1971)	s·mmHg·mL^{-1}
50	Pulmonary vascular resistance	PVR	0.0151 – 0.1353		(Klingensmith et al., 2016; Stefanadis et al., 2001)	s·mmHg·mL^{-1}
51	Fractional proximal sodium reabsorption	$\eta_{pt\_sodreab}$	0.67 – 0.97		(Fliser et al., 1997; Bochud et al., 2009; Jin et al., 2009)	–
52	Fractional sodium reabsorption in the distal tubule and subsequent parts of the nephron	$\eta_{dt\_sodreab} + \eta_{cd\_sodreab} – \eta_{dt\_sodreab} \cdot \eta_{cd\_sodreab}$	0.78 – 0.98		(Fliser et al., 1997; Bochud et al., 2009; Jin et al., 2009)	–
53	Water intake	Φ_{win}	0.00096 – 0.00312		(Kutumova et al., 2021)	L·min^{-1}

[9] N_{BV} is an estimate defined by Nadler et al. (1962). We considered $N_{BV} \pm 10\%$ as the range of total blood volume in normal humans.

References

Admiraal, P.J., Danser, A.H., Jong, M.S., Pieterman, H., Derkx, F.H., Schalekamp, M.A. (1993). Regional angiotensin II production in essential hypertension and renal artery stenosis. Hypertension. 21(2), 173-184. doi: 10.1161/01.hyp.21.2.173

Antony, I., Nitenberg, A., Foult, J.M., Aptecar, E. (1993). Coronary vasodilator reserve in untreated and treated hypertensive patients with and without left ventricular hypertrophy. J Am Coll Cardiol. 22(2), 514-520. doi: 10.1016/0735-1097(93)90058-9

Asmar, R.G., Kerihuel, J.C., Girerd, X.J., Safar, M.E. (1991). Effect of bisoprolol on blood pressure and arterial hemodynamics in systemic hypertension. Am. J. Cardiol. 68(1), 61-64. doi: 10.1016/0002-9149(91)90711-s

Bauer, J.H., Brooks, C.S., Burch, R.N. (1982). Renal function and hemodynamic studies in low- and normal-renin essential hypertension. Arch Intern Med. 142(7), 1317-1323.

Bax, L., Bakker, C.J.G., Klein, W.M., Blanken, N., Beutler, J.J., Mali, W.P.T.R.M. (2005). Renal blood flow measurements with use of phase-contrast magnetic resonance imaging: normal values and reproducibility. J Vasc Interv Radiol. 16(6), 807-814. doi: 10.1097/01.RVI.0000161144.98350.28

Bazroon, A.A., Alrashidi, N.F. (2022). Bisoprolol. In: StatPearls [Internet]. Treasure Island (FL): StatPearls Publishing.

Bertram, J.F., Douglas-Denton, R.N., Diouf, B., Hughson, M.D., Hoy, W.E. (2011). Human nephron number: implications for health and disease. Pediatr Nephrol. 26(9), 1529-1533. doi: 10.1007/s00467-011-1843-8

Billett, H.H. (1990). Hemoglobin and hematocrit. In: Clinical methods: The history, physical, and laboratory examinations. 3rd edition. Editors: Walker, H.K., Hall, W.D., Hurst, J.W. Boston: Butterworths.

Bochud, M., Staessen, J.A., Maillard, M., Mazeko, M.J., Kuznetsova, T., Woodiwiss, A., Richart, T., Norton, G., Thijs, L., Elston, R., Burnier, M. (2009). Ethnic differences in proximal and distal tubular sodium reabsorption are heritable in black and white populations. J Hypertens. 27(3):606-612. doi: 10.1097/HJH.0b013e32832104b1

Busher, J.T. (1990). Serum albumin and globulin. In: Clinical methods: The history, physical, and laboratory examinations. 3rd edition. Editors: Walker, H.K., Hall, W.D., Hurst, J.W. Boston: Butterworths.

Cachat, F., Combescure, C., Cauderay, M., Girardin, E., Chehade, H. (2015). A systematic review of glomerular hyperfiltration assessment and definition in the medical literature. Clin J Am Soc Nephrol. 10(3), 382-389. doi: 10.2215/CJN.03080314

Cannone, V., Buglioni, A., Sangaralingham, S.J., Scott, C., Bailey, K.R., Rodeheffer, R., Redfield, M.M., Sarzani, R., Burnett, Jr. J.C. (2018). Aldosterone, hypertension, and antihypertensive therapy: insights from a general population. Mayo Clin Proc. 93(8), 980-990. doi: 10.1016/j.mayocp.2018.05.027

Cattermole, G.N., Leung, P.Y., Ho, G.Y., Lau, P.W., Chan, C.P., Chan, S.S., Smith, B.E., Graham, C.A., Rainer, T.H. (2017). The normal ranges of cardiovascular parameters measured using the ultrasonic cardiac output monito. Physiol Rep. 5(6), e13195. doi: 10.14814/phy2.13195

Chagnac, A., Weinstein, T., Korzets, A., Ramadan, E., Hirsch, J., Gafter, U. (2000). Glomerular hemodynamics in severe obesity. Am J Physiol Renal Physiol. 278(5), F817- F822. doi: 10.1152/ajprenal.2000.278.5.F817

Daly, W.J., Bondurant, S. (1962). The effects of oxygen breathing on heart rate, blood pressure, and cardiac index of normal men-resting, with reactive hyperaemia, and after atropine. J Clin Invest. 41(1), 126-132. doi: 10.1172/JCI104454

Dedov, I.I., Shestakova, M.V., Mayorov, A.Yu., editors. (2017). Algorithms of specialized medical care for diabetes mellitus patients. 8-th Edition. Moscow: UP Print, 112 p. (In Russ.)

Digne-Malcolm, H., Frise, M.C., Dorrington, K.L. (2016). How Do Antihypertensive Drugs Work? Insights from Studies of the Renal Regulation of Arterial Blood Pressure. Front. Physiol. 7, 320. doi: 10.3389/fphys.2016.00320

Dijkhuizen, P., Buursma, A., Fongers, T.M., Gerding, A.M., Oeseburg, B., Zijlstra, W.G. (1977). The oxygen binding capacity of human haemoglobin. Pflugers Arch. 369(3), 223-231. doi: 10.1007/BF00582188

Doenyas-Barak, K., de Abreu, M.H.F.G., Borges, L.E., Tavares Filho, H.A., Yunlin, F., Yurong, Z., Levin, N.W., Kaufman, A.M., Efrati, S., Pereg, D., Litovchik, I., Fuchs, S., Minha, S. (2019). Non-invasive hemodynamic profiling of patients undergoing hemodialysis – a multicenter observational cohort study. BMC Nephrol. 20(1), 347. doi: 10.1186/s12882-019-1542-4

Donato, L., Coli, A., Pasqualini, R., Duce, T. (1972). Metabolic clearance rate of radioiodinated angiotensin II in normal men. Am J Physiol. 223(5), 1250-1256. doi: 10.1152/ajplegacy.1972.223.5.1250

Duarte, J.D., Cooper-DeHoff, R.M. (2010). Mechanisms for blood pressure lowering and metabolic effects of thiazide and thiazide-like diuretics. Expert. Rev. Cardiovasc. Ther. 8(6), 793-802. doi: 10.1586/erc.10.27

Duggan, J., Nussberger, J., Kilfeather, S., O'Malley, K. (1993). Aging and human hormonal and pressor responsiveness to angiotensin II infusion with simultaneous measurement of exogenous and endogenous angiotensin II. Am. J. Hypertens. 6(8), 641-647. doi: 10.1093/ajh/6.8.641

Eguchi, K., Hoshide, S., Kario, K. (2015). Effects of celiprolol and bisoprolol on blood pressure, vascular stiffness, and baroreflex sensitivity. Am. J. Hypertens. 28(7), 858-867. doi: 10.1093/ajh/hpu245

Farber, S.J., Soberman, R.J. (1956). Total body water and total exchangeable sodium in edematous states due to cardiac, renal or hepatic disease. J Clin Invest. 35(7), 779-791. doi: 10.1172/JCI103330

Ferlinz, J. (1980). Right ventricular performance in essential hypertension. Circulation. 61(1), 156-162. doi: 10.1161/01.cir.61.1.156

Gardner, M.D., Scott, R. (1980). Age- and sex-related reference ranges for eight plasma constituents derived from randomly selected adults in a Scottish new town. J Clin Pathol. 33(4), 380-385. doi: 10.1136/jcp.33.4.380

Goldberg, S., Ollila, H.M., Lin, L., Sharifi, H., Rico, T., Andlauer, O., Aran, A., Bloomrosen, E., Faraco, J., Fang, H., Mignot, E. (2017). Analysis of hypoxic and hypercapnic ventilatory response in healthy volunteers. PLoS One. 12(1), e0168930. doi: 10.1371/journal.pone.0168930

Gomez D.M. Evaluation of renal resistances, with special reference to changes in essential hypertension. J Clin Invest. 30(10):1143-1155. doi: 10.1172/JCI102534

Guberina, H., Baumann, M., Bruck, H., Feldkamp, T., Nürnberger, J., Kribben, A., Philipp, T., Witzke, O., Sotiropoulos, G., Mitchell, A. (2013). Associations of smoking with alterations in renal hemodynamics may depend on sex--investigations in potential kidney donors. Kidney Blood Press Res. 37(6), 611-621. doi: 10.1159/000355741

Ferrario, C.M., Martell, N., Yunis, C., Flack, J.M., Chappell, M.C., Brosnihan, K.B., Dean, R.H., Fernandez, A., Novikov, S.V., Pinillas, C., Luque, M. (1998). Characterization of angiotensin-(1-7) in the urine of normal and essential hypertensive subjects. Am J Hypertens. 11(2), 137-146. doi: 10.1016/s0895-7061(97)00400-7

Fischbach, F.T. (2003). Manual of laboratory and diagnostic test, 7th ed. Philadelphia: Lippincott Williams and Wilkins.

Fliser, D., Franek, E., Joest, M., Block, S., Mutschler, E., Ritz, E. (1997). Renal function in the elderly: impact of hypertension and cardiac function. Kidney Int. 51(4):1196-1204. doi: 10.1038/ki.1997.163

Fogarty, J., Loughrey, C. (2016). Hyponatraemia in hospitalised adults: a guide for the junior doctor. Ulster Med J. 86(2):84-89

Furukawa, K., Abumiya, T., Sakai, K., Hirano, M., Osanai, T., Shichinohe, H., Nakayama, N., Kazumata, K., Aida, T., Houkin, K. (2016). Measurement of human blood viscosity by an electromagnetic spinning sphere viscometer. J Med Eng Technol. 40(6), 285-292. doi: 10.1080/03091902.2016.1181216

Gazioglu, K., Yu, P.N. (1967). Pulmonary blood volume and pulmonary capillary blood volume in valvular heart disease. Circulation. 35(4), 701-709. doi: 10.1161/01.cir.35.4.701

Guasch, A., Cua, M., You, W., Mitch, W.E. (1997). Sickle cell anemia causes a distinct pattern of glomerular dysfunction. Kidney Int. 51(3), 826-833. doi: 10.1038/ki.1997.116

Hall, J.E. (2011). Guyton and Hall textbook of medical physiology. 12th edition. Philadelphia: Saunders Elsevier, 1091 p.

Hallow, K.M., Lo, A., Beh, J., Rodrigo, M., Ermakov, S., Friedman, S., de Leon, H., Sarkar, A., Xiong, Y., Sarangapani, R., Schmidt, H., Webb, R., Kondic, A.G. (2014). A model-based approach to investigating the pathophysiological mechanisms of hypertension and response to antihypertensive therapies: Extending the Guyton model. Am. J. Physiol. Regul. Integr. Comp. Physiol. 306(9), R647-R662. doi: 10.1152/ajpregu.00039.2013

Hallow, K.M., Gebremichael, Y. (2017). A quantitative systems physiology model of renal function and blood pressure regulation: model description. CPT Pharmacometrics Syst Pharmacol. 6(6), 383-392. doi: 10.1002/psp4.12178

Haluska, B.A., Jeffriess, L., Brown, J., Carlier, S., Marwick, T.H. (2010). A comparison of methods for assessing total arterial compliance. J Hum Hypertens. 24(4), 254-262. doi: 10.1038/jhh.2009.92

Hattori, N., Bergsneider, M., Wu, H.M., Glenn, T.C., Vespa, P.M., Hovda, D.A., Phelps, M.E., Huang, S.C. (2004). Accuracy of a method using short inhalation of (15)O-O(2) for measuring cerebral oxygen extraction fraction with PET in healthy humans. J Nucl Med. 45, 765-770.

Hill, G.S., Heudes, D., Jacquot, C., Gauthier, E., Bariéty, J. (2006). Morphometric evidence for impairment of renal autoregulation in advanced essential hypertension. Kidney Int. 69(5), 823-831. doi: 10.1038/sj.ki.5000163

Hoang, K., Tan, J.C., Derby, G., Blouch, K.L., Masek, M., Ma, I., Lemley, K.V., Myers, B.D. (2003). Determinants of glomerular hypofiltration in aging humans. Kidney Int. 64(4), 1417-1424. doi: 10.1046/j.1523-1755.2003.00207.x

Hoffer, E.C., Meador, C.K., Simpson, D.C. (1969). Correlation of whole-body impedance with total body water volume. J Appl Physiol. 27(4):531-534. doi: 10.1152/jappl.1969.27.4.531

Hosten, A.O. (1990). BUN and Creatinine. In: Clinical methods: The history, physical, and laboratory examinations. 3rd edition. Editors: Walker, H.K., Hall, W.D., Hurst, J.W. Boston: Butterworths.

Hoy, W.E., Hughson, M.D., Singh, G.R., Douglas-Denton, R., Bertram, J.F. (2006). Reduced nephron number and glomerulomegaly in Australian Aborigines: a group at high risk for renal disease and hypertension. Kidney Int. 70(1), 104-110. doi: 10.1038/sj.ki.5000397

Hund, S.J., Kameneva, M.V., Antaki, J.F. (2017). A quasi-mechanistic mathematical representation for blood viscosity. Fluids. 2(1), 10. doi: 10.3390/fluids2010010

Hudsmith, L.E., Petersen, S.E., Francis, J.M., Robson, M.D., Neubauer, S. (2005). Normal human left and right ventricular and left atrial dimensions using steady state free precession magnetic resonance imaging. J Cardiovasc Magn Reson. 7(5), 775-782. doi: 10.1080/10976640500295516

Inada, Y., Ojima, M., Kanagawa, R., Misumi, Y., Nishikawa, K., Naka, T. (1999). Pharmacologic properties of candesartan cilexetil – possible mechanisms of long-acting antihypertensive action. J Hum Hypertens. 13 Suppl 1, S75-S80. doi: 10.1038/sj.jhh.1000749

Jin, Y., Kuznetsova, T., Maillard, M., Richart, T., Thijs, L., Bochud, M., Herregods, M.C., Burnier, M., Fagard, R., Staessen, J.A. (2009). Independent relations of left ventricular structure with the 24-hour urinary excretion of sodium and aldosterone. Hypertension. 54(3), 489-495. doi: 10.1161/HYPERTENSIONAHA.109.130492

Kahonen, M., Ylitalo, R., Koobi, T., Turjanmaa, V., Ylitalo, P. (2000). Influences of nonselective, beta(1)-selective and vasodilatory beta(1)-selective beta-blockers on arterial pulse wave velocity in normotensive subjects. Gen. Pharmacol. 35(4), 219-224. doi: 10.1016/s0306-3623(01)00109-4

Karaaslan, F., Denizhan, Y., Kayserilioglu, A., Ozcan Gulcur, H. (2005). Long term mathematical model involving renal sympathetic nerve activity, arterial pressure, and sodium excretion. Ann Biomed Eng. 33(11), 1607-1630. doi: 10.1007/s10439-005-5976-4

Kasner, M., Westermann, D., Steendijk, P., Gaub, R., Wilkenshoff, U., Weitmann, K., Hoffmann, W., Poller, W., Schultheiss, H.P., Pauschinger, M., Tschöpe, C. (2007). Utility of Doppler echocardiography and tissue Doppler imaging in the estimation of diastolic function in heart failure with normal ejection fraction: a comparative Doppler-conductance catheterization study. Circulation. 116(6), 637-647. doi: 10.1161/CIRCULATIONAHA.106.661983

Kasner, M., Westermann, D., Steendijk, P., Dröse, S., Poller, W., Schultheiss, H.P., Tschöpe, C. (2012). Left ventricular dysfunction induced by nonsevere idiopathic pulmonary arterial hypertension: a pressure-volume relationship study. Am J Respir Crit Care Med. 186(2):181-189. doi: 10.1164/rccm.201110-1860OC

Klingensmith, M.E., Vemuri, C., Fayanju, O.M., Robertson, J.O., Samson, P.P., Sanford, D.E., editors. (2016). The Washington manual of surgery. Seventh edition. St. Louis, Missouri: Wolters Kluwer, 984 p.

Kutumova, E., Kiselev, I., Sharipov, R., Lifshits, G., Kolpakov, F. (2021). Thoroughly calibrated modular agent-based model of the human cardiovascular and renal systems for blood pressure regulation in health and disease. Front. Physiol. 12, 746300. doi: 10.3389/fphys.2021.746300

Kutumova, E., Kiselev, I., Sharipov, R., Lifshits, G., Kolpakov, F. (2022). Mathematical modeling of antihypertensive therapy. Front. Physiol. 13, 1070115. doi: 10.3389/fphys.2022.1070115

Kyhl, K., Ahtarovski, K.A., Iversen, K., Thomsen, C., Vejlstrup, N., Engstrøm, T., Madsen, P.L. (2013). The decrease of cardiac chamber volumes and output during positive-pressure ventilation. Am J Physiol Heart Circ Physiol. 305(7), H1004- H1009. doi: 10.1152/ajpheart.00309.2013

Lang, R.M., Bierig, M., Devereux, R.B., Flachskampf, F.A., Foster, E., Pellikka, P.A., Picard, M.H., Roman, M.J., Seward, J., Shanewise, J., Solomon, S., Spencer, K.T., Sutton, M.St.J., Stewart, W. (2006). Recommendations for chamber quantification. Eur J Echocardiogr. 7(2), 79-108. doi: 10.1016/j.euje.2005.12.014

Laskey, W.K., Parker, H.G., Ferrari, V.A., Kussmaul, W.G., Noordergraaf, A. (1990). Estimation of total systemic arterial compliance in humans. J Appl Physiol (1985). 69(1), 112-119. doi: 10.1152/jappl.1990.69.1.112

Lawrence, A.C., Evin, G., Kladis, A., Campbell, D.J. (1990). An alternative strategy for the radioimmunoassay of angiotensin peptides using amino-terminal-directed antisera: measurement of eight angiotensin peptides in human plasma. J. Hypertens. 8(8), 715-724. doi: 10.1097/00004872-199008000-00005

Letcher, R.L., Chien, S., Pickering, T.G., Sealey, J.E., Laragh, J.H. (1981). Direct relationship between blood pressure and blood viscosity in normal and hypertensive subjects. Role of fibrinogen and concentration. Am J Med. 70(6), 1195-1202. doi: 10.1016/0002-9343(81)90827-5

Leth, A. (1970). Changes in plasma and extracellular fluid volumes in patients with essential hypertension during long-term treatment with hydrochlorothiazide. Circulation. 42, 479-485. doi: 10.1161/01.cir.42.3.479

Levin, A., Stevens, P.E. (2013). Summary of KDIGO 2012 CKD Guideline: behind the scenes, need for guidance, and a framework for moving forward. Kidney Int. 85(1), 49-61. doi: 10.1038/ki.2013.444

Levinson, G.E., Pacifico, A.D., Frank, F.M. (1996). Studies of cardiopulmonary blood volume. Measurement of total cardiopulmonary blood volume in normal human subjects at rest and during exercise. Circulation. 33(3), 347-356. doi: 10.1161/01.cir.33.3.347

Luft, F.C., Fineberg, N.S., Sloan, R.S. (1982). Estimating dietary sodium intake in individuals receiving a randomly fluctuating intake. Hypertension. 4(6), 805-808. doi: 10.1161/01.hyp.4.6.805

Macedo, R., Prakasa, K., Tichnell, C., Marcus, F., Calkins, H., Lima, J.A., Bluemke, D.A. (2007). Marked lipomatous infiltration of the right ventricle: MRI findings in relation to arrhythmogenic right ventricular dysplasia. AJR Am J Roentgenol. 188(5), W423- W427. doi: 10.2214/AJR.06.0161

Maceira, A.M., Prasad, S.K., Khan, M., Pennell, D.J. (2006a). Reference right ventricular systolic and diastolic function normalized to age, gender and body surface area from steady-state free precession cardiovascular magnetic resonance. Eur Heart J. 27(23), 2879-2888. doi: 10.1093/eurheartj/ehl336

Maceira, A.M., Prasad, S.K., Khan, M., Pennell, D.J. (2006b). Normalized left ventricular systolic and diastolic function by steady state free precession cardiovascular magnetic resonance. J Cardiovasc Magn Reson. 8(3), 417-426. doi: 10.1080/10976640600572889

MacKay, J.H., Arcuri, K.E., Goldberg, A.I., Snapinn, S.M., Sweet, C.S. (1996). Losartan and low-dose hydrochlorothiazide in patients with essential hypertension. A double-blind, placebo-controlled trial of concomitant administration compared with individual components. Arch. Intern. Med. 156(3), 278-285. doi:10.1001/archinte.1996.00440030072009

Magder, S. (2016). Volume and its relationship to cardiac output and venous return. Crit Care. 20(1), 271. doi: 10.1186/s13054-016-1438-7

Magness, R.R., Cox, K., Rosenfeld, C.R., Gant, N.F. (1994). Angiotensin II metabolic clearance rate and pressor responses in nonpregnant and pregnant women. Am J Obstet Gynecol. 171(3), 668-679. doi: 10.1016/0002-9378(94)90080-9

Malisova, O., Athanasatou, A., Pepa, A., Husemann, M., Domnik, K., Braun, H., Mora-Rodriguez, R., Ortega, J.F., Fernandez-Elias, V.E., Kapsokefalou, M. (2016). Water intake and hydration indices in healthy European adults: the European hydration research study (EHRS). Nutrients. 8(4), 204. doi: 10.3390/nu8040204

Marini, J.J., Leatherman, J.W. (2005). Pulmonary artery occlusion pressure: measurement, significance, and clinical uses. In: Functional hemodynamic monitoring. Update in intensive care and emergency medicine 42. Editors: Pinsky, M.R., Payen, D. Berlin, Heidelberg: Springer.

Mason, J.W., Ramseth, D.J., Chanter, D.O., Moon, T.E., Goodman, D.B., Mendzelevski, B. (2007). Electrocardiographic reference ranges derived from 79,743 ambulatory subjects. J Electrocardiol. 40(3), 228-234. doi: 10.1016/j.jelectrocard.2006.09.003

Moore, F.D. (1967). Body composition and its measurement in vivo. British Journal of Surgery. 54(13), 431-435. doi: 10.1002/bjs.1800541309

Nadler, S.B., Hidalgo, J.U., Bloch, T. (1962). Prediction of blood volume in normal human adults. Surgery. 51, 224-232

Natarajan, A.R., Eisner, G.M., Armando, I., Browning, S., Pezzullo, J.C., Rhee, L., Dajani, M., Carey, R.M., Jose, P.A. (2016). The renin-angiotensin and renal dopaminergic systems interact in normotensive humans. J Am Soc Nephrol. 27(1), 265-279. doi: 10.1681/ASN.2014100958

Neal, C.R., Arkill, K.P., Bell, J.S., Betteridge, K.B., Bates, D.O., Winlove, C.P., Salmon, A.H.J., Harper, S.J. (2018). Novel hemodynamic structures in the human glomerulus. Am J Physiol Renal Physiol. 315(5), F1370-F1384. doi: 10.1152/ajprenal.00566.2017

Nozuki, M., Mouri, T., Itoi, K., Takahashi, K., Totsune, K., Saito, T., Yoshinaga, K. (1986). Plasma concentrations of atrial natriuretic peptide in various diseases. Tohoku J Exp Med. 148(4), 439-447. doi: 10.1620/tjem.148.439

Nussberger, J., Brunner, D., Keller, I., Brunner, H.R. (1992). Measurement of converting enzyme activity by antibody-trapping of generated angiotensin II. Comparison with two other methods. Am. J. Hypertens. 5(6 Pt 1), 393-398. doi: 10.1093/ajh/5.6.393

Ong, K.T., Delerme, S., Pannier, B., Safar, M.E., Benetos, A., Laurent, S., Boutouyrie, P., investigators. (2011). Aortic stiffness is reduced beyond blood pressure lowering by short-term and long-term antihypertensive treatment: a meta-analysis of individual data in 294 patients. J. Hypertens. 29(6), 1034-1042. doi: 10.1097/HJH.0b013e328346a583

Oparil, S., Acelajado, M.C., Bakris, G.L., Berlowitz, D.R., Cífková, R., Dominiczak, A.F., Grassi, G., Jordan, J., Poulter, N.R., Rodgers, A., Whelton, P.K. (2018). Hypertension. Nat Rev Dis Primers. 4, 18014. doi: 10.1038/nrdp.2018.14

Ostchega, Y., Porter, K.S., Hughes, J., Dillon, C.F., Nwankwo, T. (2011). Resting pulse rate reference data for children, adolescents, and adults: United States, 1999-2008. Natl Health Stat Report. (41), 1-16.

Pagani, E.D., Alousi, A.A., Grant, A.M., Older, T.M., Dziuban, S.W.Jr., Allen, P.D. (1988). Changes in myofibrillar content and Mg-ATPase activity in ventricular tissues from patients with heart failure caused by coronary artery disease, cardiomyopathy, or mitral valve insufficiency. Circ Res. 63(2), 380-385. doi: 10.1161/01.res.63.2.380

Palmieri, E.A., Fazio, S., Palmieri, V., Lombardi, G., Biondi, B. (2004). Myocardial contractility and total arterial stiffness in patients with overt hyperthyroidism: acute effects of beta1-adrenergic blockade. Eur. J. Endocrinol. 150(6), 757-762. doi: 10.1530/eje.0.1500757

Payne, R.B., Levell, M.J. (1968). Redefinition of the normal range for serum sodium. Clin Chem. 14(2), 172-178.

Perschel, F.H., Schemer, R., Seiler, L., Reincke, M., Deinum, J., Maser-Gluth, C., Mechelhoff, D., Tauber, R., Diederich, S. (2004). Rapid screening test for primary hyperaldosteronism: ratio of plasma aldosterone to renin concentration determined by fully automated chemiluminescence immunoassays. Clin Chem. 50(9):1650-1655. doi: 10.1373/clinchem.2004.033159

Pfisterer, M.E., Battler, A., Zaret, B.L. (1985). Range of normal values for left and right ventricular ejection fraction at rest and during exercise assessed by radionuclide angiocardiography. Eur Heart J. 6(8), 647-655. doi: 10.1093/oxfordjournals.eurheartj.a061916

Porthan, K., Viitasalo, M., Hiltunen, T.P., Vaananen, H., Dabek, J., Suonsyrja, T., Hannila-Handelberg, T., Virolainen, J., Nieminen, M.S., Toivonen, L., Kontula, K., Oikarinen, L. (2009). Short-term electrophysiological effects of losartan, bisoprolol, amlodipine, and hydrochlorothiazide in hypertensive men. Ann. Med. 41(1), 29-37. doi: 10.1080/07853890802195211

Proshin, A.P., Solodyannikov, Yu.V. (2006). Mathematical modeling of blood circulation system and its practical application. Automation and Remote Control. 67(2), 329–341. doi: 10.1134/S000511790602010X

Rapoport, R.M., Soleimani, M. (2019). Mechanism of thiazide diuretic arterial pressure reduction: the search continues. Front. Pharmacol. 10, 815. doi: 10.3389/fphar.2019.00815

Rastegar, A. (1990). Serum Potassium. In: Clinical methods: The history, physical, and laboratory examinations. 3rd edition. Editors: Walker, H.K., Hall, W.D., Hurst, J.W. Boston: Butterworths.

Rodgers, K.E., Oliver, J., diZerega, G.S. (2006). Phase I/II dose escalation study of angiotensin 1-7 [A(1-7)] administered before and after chemotherapy in patients with newly diagnosed breast cancer. Cancer Chemother Pharmacol. 57(5), 559-568. doi: 10.1007/s00280-005-0078-4

Prys-Roberts, C., Meloche, R., Foëx, P. (1971). Studies of anaesthesia in relation to hypertension. I. Cardiovascular responses of treated and untreated patients. Br J Anaesth. 43(2):122-137. doi: 10.1093/bja/43.2.122

Saghiv, M., Sagiv, M. (2017). Response of left ventricular volumes and ejection fraction during different modes of exercise in health and CAD patients. International Journal of Clinical Cardiology. 1, 51–56.

Scaglione, R., Indovina, A., Parrinello, G., Lipari, R., Mulè, L.G., Ganguzza, A., Capuana, G., Stampino, C.G., Licata, G. (1992). Antihypertensive efficacy and effects of nitrendipine on cardiac and renal hemodynamics in mild to moderate hypertensive patients: randomized controlled trial versus hydrochlorothiazide. Cardiovasc. Drugs. Ther. 6, 141-146. doi: 10.1007/BF00054562

Scaglione, R., Ganguzza, A., Corrao, S., Costa, R., Paternà, S., Cannavo, M.G., Parrinello, G., Di Chiara, T., D'Aubert, M.D., Cottone, C., et al. (1995). Effects of cilazapril on renal haemodynamics and function in hypertensive patients: a randomised controlled study versus hydrochlorothiazide. Blood Press. 4, 363-368. doi: 10.3109/08037059509077622

Skrabal, F. (1974). Half-life of plasma renin activity in normal subjects and in malignant hypertension. Klin Wochenschr.52(24), 1173-1174. doi: 10.1007/BF01466736

Škrtić, M., Lytvyn, Y., Yang, G.K., Yip, P., Lai, V., Silverman, M., Cherney, D.Z. (2015). Glomerular haemodynamic profile of patients with Type 1 diabetes compared with healthy control subjects. Diabet Med. 32(7), 972-979. doi: 10.1111/dme.12717

Shah, S., Khatri, I., Freis, E.D. (1978). Mechanism of antihypertensive effect of thiazide diuretics. Am. Heart. J. 95(5), 611-618. doi: 10.1016/0002-8703(78)90303-4

Solodyannikov, Yu.V. (1994). Elements of mathematical modeling and identification of blood circulation system. Samara: Samara University, 316 p. (In Russ.)

Stefanadis, C., Manolis, A., Dernellis, J., Tsioufis, C., Tsiamis, E., Gavras, I., Gavras, H., Toutouzas, P. (2001). Acute effect of clonidine on left ventricular pressure-volume relation in hypertensive patients with diastolic heart dysfunction. J Hum Hypertens. 15(9), 635-642. doi: 10.1038/sj.jhh.1001243

Thenappan, T., Prins, K.W., Pritzker, M.R., Scandurra, J., Volmers, K., Weir, E.K. (2016). The critical role of pulmonary arterial compliance in pulmonary hypertension. Ann Am Thorac Soc. 13(2), 276-284. doi: 10.1513/AnnalsATS.201509-599FR

Treacher, D.F., Leach, R.M. (1998). Oxygen transport – 1. Basic principles. BMJ. 317(7168), 1302-1306. doi: 10.1136/bmj.317.7168.1302

Tsuda, A., Ishimura, E., Uedono, H., Ochi, A., Nakatani, S., Morioka, T., Mori, K., Uchida, J., Emoto, M., Nakatani, T., Inaba, M. (2018). Association of albuminuria with intraglomerular hydrostatic pressure and insulin resistance in subjects with impaired fasting glucose and/or impaired glucose tolerance. Diabetes Care. 41(11):2414-2420. doi: 10.2337/dc18-0718

Valabhji, J., Donovan, J., Kyd, P.A., Schachter, M., Elkeles, R.S. (2001). The relationship between active renin concentration and plasma renin activity in Type 1 diabetes. Diabet Med. 18(6), 451-458. doi: 10.1046/j.1464-5491.2001.00489.x

van Brummelen, P., Man in't Veld, A.J., Schalekamp, M.A. (1980). Hemodynamic changes during long-term thiazide treatment of essential hypertension in responders and nonresponders. Clin. Pharmacol. Ther. 27(3), 328-336. doi: 10.1038/clpt.1980.44

Villamil, A., Chrysant, S.G., Calhoun, D., Schober, B., Hsu, H., Matrisciano-Dimichino, L., Zhang, J. (2007). Renin inhibition with aliskiren provides additive antihypertensive efficacy when used in combination with hydrochlorothiazide. J. Hypertens. 25, 217-226. doi: 10.1097/HJH.0b013e3280103a6b

Wennesland, R., Brown, E., Hopper, J.Jr., Hodges, J.L.Jr., Guttentag, O.E., Scott, K.G., Tucker, I.N., Bradley, B. (1959). Red cell, plasma and blood volume in healthy men measured by radiochromium (Cr51) cell tagging and hematocrit: influence of age, somatotype and habits of physical activity on the variance after regression of volumes to height and weight combined. J Clin Invest. 38(7), 1065-1077. doi: 10.1172/JCI103883

Yarmohammadi, H., Erinjeri, J.P., Brown, K.T. (2015). Embolization of metastatic neuroendocrine tumor resulting in clinical manifestations of syndrome of inappropriate secretion of antidiuretic hormone. J Vasc Interv Radiol. 26(4), 533-537. doi: 10.1016/j.jvir.2014.11.032

Zhang, W., Kovács, S.J. (2008). The diastatic pressure-volume relationship is not the same as the enddiastolic pressure-volume relationship. Am J Physiol Heart Circ Physiol. 294(6), H2750- H2760. doi: 10.1152/ajpheart.00200.2008

Zhou, W.J., Wang, R.Y., Li, Y., Chen, D.R., Chen, E.Z., Zhu, D.L., Gao, P.J. (2013). A randomized controlled study on the effects of bisoprolol and atenolol on sympathetic nervous activity and central aortic pressure in patients with essential hypertension. PLoS One. 8(9), e72102. doi: 10.1371/journal.pone.0072102
